# Polyamino-Isoprenyl Derivatives as Antibiotic Adjuvants and Motility Inhibitors for *Bordetella bronchiseptica* Porcine Pulmonary Infection Treatment

**DOI:** 10.3389/fmicb.2019.01771

**Published:** 2019-08-13

**Authors:** Diane Borselli, Jean Michel Brunel, Olivier Gorgé, Jean Michel Bolla

**Affiliations:** INSERM, SSA, IRBA, MCT, Aix-Marseille University, Marseille, France

**Keywords:** antibiotic resistance, combination therapy, whole-cell screening, motility, virulence, *Bordetella bronchiseptica*

## Abstract

The spreading of multidrug-resistant bacteria and the lack of novel antibiotic molecules leave clinicians and veterinarians with very limited options to treat bacterial infections, especially those caused by Gram-negative pathogens. To reduce the selection of antibiotic resistance mechanisms and their transfer to human pathogens, veterinary pharmaceutical companies have dramatically decreased the number of antibiotics used. Among all the investigated alternate solutions, chemosensitizers, which decrease the amount of the used drugs, appear to be one of the most promising strategies. In this study, we reported that polyamino-isoprenyl derivatives can potentiate florfenicol activity against veterinary sensitive reference strains as well as clinical isolates. These molecules induce inner membrane depolarization and subsequently inhibit efflux pumps by collapsing the proton-motive force (PMF). Considering that *Bordetella bronchiseptica* rotor flagellum is highly PMF dependent and that flagellar motility represents an important factor involved in colonization, we monitored the swimming and swarming motilities of bacteria and showed a strong inhibition in the presence of the lead selected compound. Taken together, our results suggest that this class of molecules are able to increase treatment efficacy and decrease drug consumption.

## Introduction

In Europe, the overall sales of veterinary antimicrobial agents used in food-producing animals represented 7,680 t in 2016^1^, and the main use (around 60%) concerns the pig industry (Schwarz et al., 2001; Chauvin et al., 2002). The release of antibiotics in the environment by livestock production is one of the major contributing factors for the emergence of bacterial resistance in pathogens affecting both humans and animals (Jechalke et al., 2014; Wichmann et al., 2014; Bártíková et al., 2016; Albero et al., 2018). Thus, it is crucial to decrease the antibiotic consumption in the veterinary field and particularly in livestock without affecting animal health (Pruden et al., 2013; Wellington et al., 2013). Consequently, strategies have been proposed, and one of the most successful consists of a dual drug approach allowing an efficient enhancement of antibacterial activity while minimizing the drug concentration (Brooks and Brooks, 2014; Collignon et al., 2016).

The World Health Organization and the European Commission have published guidelines concerning the prudent use, in food production animals, of cephalosporin of third and fourth generation and fluoroquinolones, which are considered as “critically important” antimicrobial agents in human medicine (see footnote 1, McArthur et al., 2013; Briyne et al., 2014). According to the international recommendations, the antimicrobials that could be used to treat porcine respiratory tract infections are tetracyclines, phenicols, macrolides, fluoroquinolones, or a combination of trimethoprim and sulfonamides (Kadlec et al., 2004, 2007). It is noteworthy that respiratory diseases in swine such as pneumonia and atrophic rhinitis are caused by the Gram-negative coccobacillus *Bordetella bronchiseptica* that requires a tetracycline treatment even if resistance can occur (Prüller et al., 2015; Kadlec and Schwarz, 2018). Otherwise, florfenicol is a good alternative (Chauvin et al., 2002), because this antibiotic has a good distribution in the body and has few side effects. However, although florfenicol was only used in veterinary medicine, a cross-resistance by efflux with its non-fluorinated analog, chloramphenicol, has been evidenced (Kadlec et al., 2007).

In an ongoing project dedicated to reducing the florfenicol consumption from breeding pig holdings, we screened polyamino-isoprenyl compounds that were previously described (Borselli et al., 2016) for their ability to potentiate florfenicol activity. The objective of this study is to identify a compound with significant activity and to elucidate its mechanism of action on the membrane physiology and to evaluate its impact on the bacterial motility.

## Materials and Methods

### Bacterial Strains and Growth Conditions

The *B. bronchiseptica* strain used in this study comes from the Institut Pasteur collection, CIP 55.110. Ten isolates from pig husbandries in France were obtained from Labofarm (Loudéac France) (Table 1). The bacterial identification was carried out by matrix-assisted laser desorption ionization – time of flight (MALDI-TOF) Biotyper (Brüker, Billerica, Germany) with comparison with the manufacturer’s bacterial database. All the strains were grown either on Columbia agar at 35°C for 24–48 h or in Columbia broth at 35°C for 24 h.

**TABLE 1:** Susceptibilities to florfenicol (FF) and polyamine derivatives (Cpd1–Cpd7) of various *B. bronchiseptica* strains.

**Strains**	**Source**	**MIC (μg/ml)**		**MIC (μM)**					
			
		**FF**	**Cpd1**	**Cpd2**	**Cpd3**	**Cpd4**	**Cpd5**	**Cpd6**	**Cpd7**
CIP55.110	Pasteur Institute	4	187.5	50	50	50	>750	>750	375
42-F10	Labofarm	8	187.5	50	50	50	>750	>750	375
48-J7	Labofarm	4	187.5	50	100	50	>750	>750	375
77-C4	Labofarm	4	187.5	50	100	100	>750	>750	375
62-H4	Labofarm	4	187.5	50	50	50	>750	>750	375
73-B14	Labofarm	2	187.5	50	100	100	>750	>750	375
67-A11	Labofarm	4	187.5	50	100	50	>750	>750	375
78-E4	Labofarm	4	187.5	50	100	50	>750	>750	375
77-A12	Labofarm	4	187.5	50	100	50	>750	>750	375
SR11-24	Labofarm	2	187.5	50	50	50	>750	>750	375
SR11-14	Labofarm	32	187.5	50	50	50	>750	>750	375

### Antibiotics and Chemicals

The antibiotic florfenicol was supplied by the pharmaceutical company Virbac (Carros, France). The antibiotic imipenem was purchased from Sequoia Research Products (Pangbourne, United Kingdom). All the chemicals polymyxin B, polymyxin B nonapeptide (PMBN), phenylalanine-arginine beta- naphthylamide (PAβN), carbonylcyanide m-chlorophenylhy- drazone (CCCP), cetyl trimethylammonium bromide (CTAB), dimethyl sulfoxide (DMSO), 3,3′-dipropylthiacarbocyanine iodide [DiSC_3_(3)], hydroxyethyl piperazineethanesulfonic acid (HEPES), ethylene diamine tetraacetic acid (EDTA), and benzalkonium chloride were purchased from Sigma-Aldrich (St Quentin Fallavier, France). The polyamino-isoprenyl derivatives chemolibrary was described previously (Bolla et al., 2012).

### MIC Determination

Minimal inhibitory concentrations of antibiotics (MIC) were measured by the microdilution broth method in 96-well microtiter plates using the twofold dilution method and according to the NCCLS guidelines. The inoculum was adjusted at 5 × 10^5^ CFU/ml in a final volume of 200 μl. MIC was visually determined after 24-h incubation at 37°C. Assays were performed in three independent biological experiments.

### High Content Screening

The polyamino-isoprenyl derivatives chemical library was screened at 10 μM final concentration in the absence and in the presence of sub-inhibitory concentration of florfenicol (MIC/4) to identify only antibiotic adjuvants. We also added control molecules as polymyxin B, PAβN, CCCP, CTAB, and benzalkonium chloride at 10 μM. The screening was performed in 96-well microtiter plates in Columbia broth with a final volume of 200 μl and a DMSO final concentration of 2.5%. The bacterial inoculum was adjusted to 5 × 10^5^ CFU/ml. Microtiter plates were read at 600 nm on an Infinite M200 Pro plate reader (Tecan, Lyon, France) after 24-h incubation at 35°C. Assays were performed in three independent biological experiments.

### General Procedure for the Synthesis of Compounds for the Hit to Lead Procedure

The general synthetic pathway is illustrated for the preparation of compound 1.

A mixture of farnesal (345 mg, 2.27 mmol), titanium(IV) isopropoxide (645 mg, 2.27 mmol), and spermine (2.27 mmol) in absolute methanol (5 ml) was stirred at room temperature for 12 h. Sodium borohydride (172 mg, 4.5 mmol) was then added at 0°C, and the resulting mixture was stirred for an additional 2 h. The reaction was then quenched by adding water (1 ml). Stirring was maintained at room temperature for 20 min. After filtration over a pad of Celite washing with methanol and ethylacetate, the solvents were removed under vacuum and the crude amine was purified by flash chromatography on silica gel, using CH_2_Cl_2_/MeOH/NH_4_OH (7/3/1) as eluent, affording the expected coupling product 1 in 64% yield (see also Supplementary Material).

### Checkerboard Assay

Combinations of polyamino-isoprenyl derivatives with florfenicol were tested by using the previously described microdilution checkerboard method (Kadlec and Schwarz, 2018). Briefly, an inoculum (100 μl) of 5 × 10^5^ CFU/ml of *B. bronchiseptica* strain was added to 96-well microtiter plates containing a serial twofold dilution of antimicrobial agents in Columbia broth. After incubation of plates during 24 h at 35°C, we determined the MIC for each combination and calculated the fractional inhibitory concentration indexes (FICIs), allowing us to characterize the level of efficiency of the combination. The FICIs are calculated as follows: FICI = FIC A + FIC B, where FIC A = MIC antibacterial A in combination/MIC antibacterial A alone and FIC B = MIC antibacterial B in combination/MIC antibacterial B alone. The antibacterial combination was considered synergistic when the FICI was ≤ 0.5, indifferent when 0.5 < FICI ≤ 4, and antagonistic when FICI > 4 (Odds, 2003).

### Outer Membrane Permeabilization Assay

One hundred milliliters of Columbia broth was inoculated with an overnight culture of a *B. bronchiseptica* isolate producing β-lactamases (isolate 77-A12). Once the cultures reached the mid-logarithmic phase, imipenem was added (0.125 mg/L final concentration) for 2 h, and then cells were recovered by centrifugation (3,600 × *g* for 20 min at 20°C) and washed twice in 20 mM potassium phosphate buffer (pH 7.2) and 1 mM MgCl_2_ (PPB) supplemented with 30 μM of CCCP that allows the inactivation of active efflux (Nagano and Nikaido, 2009). After the second centrifugation, the cell suspension was adjusted to 0.5 OD 600 nm. One hundred microliters of the bacterial suspension was mixed with 50 μl of compound derivative dilutions already set up in microplates, to give final concentrations ranging from 1.56 to 200 μM. Then, 50 μl of nitrocefin was added to obtain a final concentration of 50 μg/ml. The absorbance at 490 nm was monitored to follow the nitrocefin hydrolysis using a Sunrise microplate reader (Tecan) over 2 h and 30 min. Experiments were performed in triplicate. For each compound, the efficacy of permeation was determined using the slope in the linear range, relative to the control slope obtained with 100 μM polymyxin-B.

### Real-Time Efflux Assay

The strain CIP55.110 was inoculated into 20 ml of Columbia broth and grown for 24 h at 35°C to reach the stationary phase. Cells were recovered by centrifugation (3,600 × *g* for 20 min at 20°C) and washed twice in PPB. The cell suspension was adjusted to 0.5 OD 600 nm and loaded with 1,2′ dinaphthylamine (TCI-Europe SA, Zwinjndretch, Belgium) 32 μM final concentration, supplemented with the proton dissipator CCCP 30 μM. After overnight incubation, cells were washed in PPB and the bacterial suspension was adjusted to 0.5 OD 600 nm. In a 96-well Greiner black microplate (Greiner, Courtaboeuf, France), 50 μl of the desired tested compounds were mixed with 100 μl of the cell suspension, yielding final concentration ranging from 12.5 to 200 μM. The dye transport was triggered after addition of 5 μl of Columbia broth and the fluorescence was monitored every 22 s for 30 min on an Infinite M200 microplate reader (Tecan) (excitation wavelength 370 nm and emission wavelength 420 nm). Polymyxin B is used as control in our experiments; this antibiotic is able to rapidly disrupt the proton-motive force (PMF) without interfering with fluorescence measurements. Assays were performed in three independent biological experiments.

### Inner Membrane Depolarization Assay

An overnight culture of bacterial strain CIP 55.110 was diluted 50-fold into 20 ml of Columbia broth. After reaching an OD 600 nm of 0.5, cells were recovered by centrifugation (3,600 × *g* for 20 min at 20°C) and incubated for 5 min at room temperature in 5 mM HEPES–10 mM EDTA (pH 7.0). They were then centrifuged (3,600 × *g* for 20 min at 20°C) and re-suspended in 5 mM HEPES (pH 7.0) supplemented with Columbia Broth 2.5% and adjusted to an OD 600 nm of 0.29. Fluorescence was monitored every 30 s (excitation wavelength 622 nm, emission wavelength 690 nm) after addition of 5 μl of a 160 μM solution of DiSC_3_(3) to 100 μl of the cell suspension to reach a final concentration of 7.6 μM. After a period of incubation (16.5 min) to allow the dye incorporation into the polarized membranes, 10 μl of compound dilutions was added. The difference in the relative fluorescence values (RFU) from the control containing only buffer and the control containing untreated bacteria in buffer is taken as the maximum level of depolarization. Assays were performed in three independent experiments.

### Motility Assay

Swimming motility was evaluated on tryptone-agar plates [tryptone (1% w/v), 0.5% NaCl (w/v), yeast extract 0.5% (w/v)] supplemented with agar 0.3% (w/v) (Murray and Kazmierczak, 2006). Swarming motility was evaluated on tryptone-agar plates supplemented with agar 0.5% (w/v) (Murray and Kazmierczak, 2006). For both assays, sterilized tryptone-agar was dispensed to Petri dishes containing either no compound or compound **1** (Cpd**1**) at 10 μM and left to dry at room temperature for 2 h. An inoculum obtained after dilution of an overnight culture was adjusted to an OD 600 nm of 1.1. The plates were inoculated with a sterile toothpick and incubated in a non-reverse position at 35°C during the indicated times (24, 48, or 72 h) and halo diameters corresponding to bacterial growth were measured and reported. Assays were performed in three independent biological experiments.

### Microscopy Live Tracking and Acquisition

A drop of each bacterial culture in exponential growth was deposited on a microscope slide and Cpd**1** was added where indicated and immediately observed. Acquisition was performed in resonant mode with the LEICA SP5 CLS by using a laser HeNe at 633 nm and a transmission PMT. The scans were recorded at a 512 × 512 resolution size and time laps over 200 frames. Their subsequent analysis was realized with the free software ImageJ using the MtrackJ plugging permitting to determine the average speed in micrometers per second of each bacterium followed. The results reported correspond to mean values obtained from 15 tracking measurements for each condition analyzed.

### Time-Kill Study

The effectiveness of Cpd**1** and florfenicol combination against the reference strain CIP 55.110 was determined by the time-kill assay. An overnight culture was diluted to 50-fold into 5 ml of Columbia agar and grown until the cell suspension reached the mid-logarithmic phase. The inoculum was then adjusted to 10^7^ CFU/ml in Columbia broth supplemented with either no addition, sub-inhibitory concentration of florfenicol (MIC/4), sub-inhibitory concentration of Cpd**1** (7.5 μM), or sub-inhibitory concentration of both. Each tube containing 1 ml was incubated at 35°C, and the bacterial count was determined after several times of incubation: 0, 1, 3, 6, 9, and 12 h by spreading the appropriate dilutions on Columbia agar plates. The plates were incubated overnight at 35°C before colony counting. The curves obtained are the result of two independent experiments.

### Libraries Construction for Bioinformatic Analysis

Libraries were prepared with NebNext kit (New England Biolabs, Ipswich, MA, United States) after Covaris ME220 Covaris Focused-ultrasonicator (Covaris, Woburn, MA, United States) shearing, according to manufacturer protocol, and sequenced on a NextSeq550 (Illumina, San Diego, CA, United States) with High Output 300 cycles V2 cartridge (ref. FC-404-2004). Antibiotic resistance profiles of the four strains were tested against CARD (22) and oneCodex, with confirmation of phenotypic resistance except for florfenicol.

## Results

### High Content Screening: Selection of Florfenicol Antibiotic Chemosensitizer

The MICs to florfenicol of 10 clinical strains and the reference one were determined, with values ranging from 2 to 32 mg/L showing different levels of resistance to this antibiotic (Table 1). A polyamino-isoprenyl chemical library containing 60 molecules has been tested at a 10 μM concentration in combination with florfenicol at sub-inhibitory concentration (MIC/4 = 1 mg/L) on the reference strain of *B. bronchiseptica* CIP 55.110 to identify compounds able to decrease florfenicol MIC below 1 mg/L. To avoid compounds with intrinsic activity, a control experiment was performed in the absence of florfenicol. We then identified compounds that could only kill bacteria in the presence of florfenicol. Among the tested compounds, only Cpd**1** led to an 85% inhibition of the bacterial growth under these experimental conditions.

### Efficacy of Compounds on Animal Isolates

**FIGURE 1:**
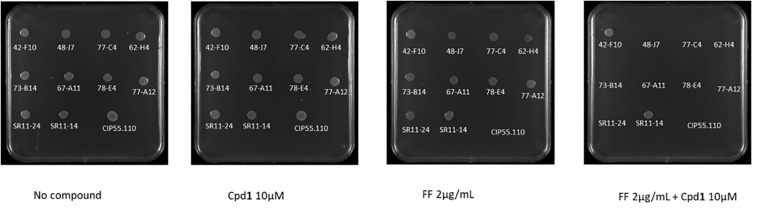
The ability of *B. bronchiseptica* reference strain CIP55,110 (5 μl of 5 × 10^5^ CFU were spotted on the plates) to grow under various experimental conditions was assessed in solid media containing (left to right) no compound, Cpd1 (compound 1) (10 μM), FF (Florfenicol) (2 μg/ml), and FF (2 μg/ml) + Cpd1 (10 μM).

To extend our study, we evaluated the efficacy of Cpd**1** on 10 animal isolates. This compound was able to restore the susceptibility of 8/10 isolates to florfenicol. Nevertheless, two of them were still resistant to florfenicol alone and in combination (Figure 1). It is noteworthy that, as already reported in literature (Corbiere-Priot and Duménil, 1996), a difference in MIC values was observed, depending on the method used for the evaluation; the MIC value being higher by using the microdilution broth method with respect to the one obtained by agar plate method.

### Hit to Lead Procedure

**FIGURE 2:**
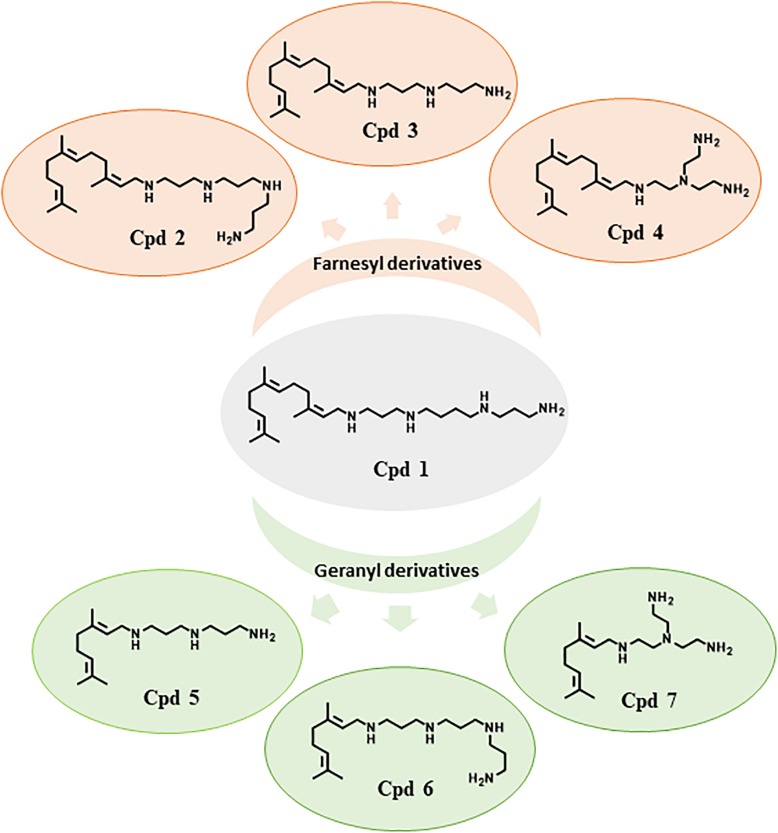
Structure of farnesyl and geranyl polyamine derivatives Cpd**1**–Cpd**7**.

In the perspective of improving the efficiency of Cpd**1**, several derivatives were synthetized (compounds **2–7**, Figure 2) as their corresponding water-soluble tartrate salts to increase their solubility in bacteria growth media and reduce solvent detrimental side effects.

Compounds **1–4** possess a common farnesyl moiety, whereas derivatives **5–7** possess a geranyl one. Moreover, each pair Cpd**2**/Cpd**6**, Cp**3**/Cpd**5**, and Cpd**4**/Cpd**7** have in common the same polyamine group (Figure 2). The MICs of these compounds were determined for each strain; no significant intrinsic activity was observed for any of these new compounds (Table 1).

### Checkerboard Assay

To determine both the minimal efficient combinations of the designed compounds and florfenicol that are necessary to inhibit the bacterial growth, we performed a checkerboard assay on animal isolates, with the strain CIP 55.110 being used as control. The results are summarized in Table 2. Cpd**1** exhibited a FIC index of about 0.5 that reflects the strong synergy between Cpd**1** and florfenicol used at MIC/2. We noticed that higher concentrations are necessary to observe a synergy using florfenicol at MIC/4. We also observed the same range of FIC index for the farnesyl compounds (Cpd**2**–Cpd**4**). On the other hand, we were unable to determine the FIC indexes for the geranyl ones (Cpd**5**–Cpd**7**) due to the high concentrations needed to inhibit the bacterial growth as mentioned by ND in Table 2.

**TABLE 2:** Fractional inhibitory concentration index obtained for each compound tested leading to a decrease of florfenicol concentration of 50 and 75%, respectively, under conditions A and B.

	**Cpd1**		**Cpd2**		**Cpd3**		**Cpd4**		**Cpd5**		**Cpd6**		**Cpd7**	
							
	**A**	**B**	**A**	**B**	**A**	**B**	**A**	**B**	**A**	**B**	**A**	**B**	**A**	**B**
CIP 55.110	0.50	0.29	0.56	0.75	0.56	0.50	0.63	0.38	0.53	ND	0.50	ND	0.53	ND
42-F10	0.51	ND	0.56	1.25	0.53	0.75	0.63	0.75	0.63	ND	0.57	ND	0.63	ND
48-J7	0.54	0.33	0.75	1.25	0.63	0.75	1.00	1.25	0.77	ND	0.63	ND	0.63	ND
77-C4	0.52	ND	0.56	0.75	0.53	1.25	0.63	1.25	0.57	ND	0.52	ND	0.57	ND
62-H4	0.54	ND	1.00	0.50	0.75	1.25	0.75	1.25	0.77	ND	ND	ND	ND	ND
73-B14	0.51	ND	0.63	0.75	0.53	0.75	0.63	1.25	ND	ND	0.63	ND	ND	ND
67-A11	0.50	0.26	0.53	0.50	0.52	0.38	0.53	0.50	0.63	ND	0.50	0.28	0.57	ND
78-E4	0.51	ND	0.75	0.25	0.52	0.75	0.53	0.50	0.51	0.38	0.50	0.38	0.57	ND
77-A12	0.50	0.26	0.53	0.50	0.52	0.50	0.53	0.50	0.52	0.32	0.52	0.32	0.57	ND
SR11-24	0.52	ND	0.75	0.75	0.53	1.25	0.56	0.75	0.63	ND	0.57	ND	0.57	ND
SR11-14	0.51	ND	0.63	0.75	0.56	0.75	1.00	0.75	0.63	ND	0.57	ND	0.57	ND

*Condition A, MIC/2; Condition B, MIC/4; ND, not determined.*

### Presence of an Active Efflux in *B. bronchiseptica*

**FIGURE 3:**
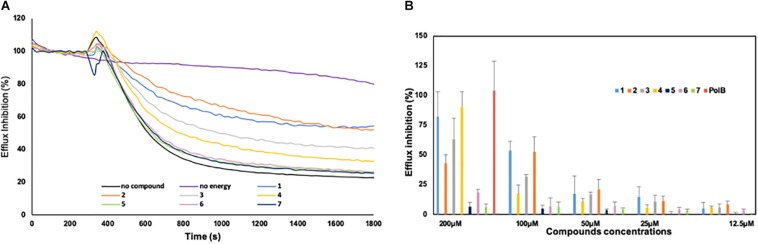
Inhibition of the efflux of the 1,2′ dinaphthylamine dye by derivatives 1–7. **(A)** Efflux was triggered after 200 s by the addition of culture media. The intensity of fluorescence emission for 1,2′ dinaphthylamine is given in relative fluorescence units (RFU). Each compound was tested at 100 μM concentration; as controls, an experiment without addition of compounds and culture media (purple curve) was also included. **(B)** Percentage of efflux inhibition obtained for each compound. Error bars represent standard deviations of three independent biological experiments.

For all the strains tested, we observed a MIC for florfenicol ranging from 2 to 4 μg/ml except for two strains 42-F10 and SR11-14 exhibiting a MIC over the resistance breakpoint of 8 μg/ml (White et al., 2000). We previously reported that efflux mechanisms are produced at a basal level in Gram-negative bacteria (Mamelli et al., 2007; Borselli et al., 2016) and can account for the observed low level of resistance that can be strongly induced when exposed to toxic compounds (Ghisalberti et al., 2005; Hay et al., 2013). To evaluate the presence of an active efflux in *B. bronchiseptica*, we measured the ability of the strain CIP55.110 to expel the 1,2′dNA, a fluorescent dye that is a substrate of efflux pumps (Bohnert et al., 2011). According to our previous report (Borselli et al., 2016), we first observed an accumulation of the dye after addition of CCCP at a 30 μM final concentration whereas no accumulation was observed in its absence. This suggested that de-energization of the cytoplasmic membrane by CCCP blocked dye releasing. Conversely, pre-loaded bacteria incubated with culture media led to a fluorescence decrease, suggesting an active efflux as shown in Figure 3 (black lane) where the strain CIP55.110 can expel about 75% of the pre-loaded dye. This result allowed us to compare the ability of the different compounds to inhibit this efflux.

### Action of the Derivatives on Dye Efflux

Some combinations between compounds **1–7** and florfenicol were found to restore susceptibility of the strains toward this antibiotic. This might suggest that the action of the compounds contributed to a better accumulation of florfenicol into the bacteria, increasing the intracellular concentration over the threshold of susceptibility.

To understand how the derivatives and especially the farnesyl ones could potentiate the antibiotic activity, we evaluated their ability to block the efflux activity in *B. bronchiseptica* (Figure 3). An example of the results obtained with compounds used at 100 μM is presented in Figure 3A. As shown on this graph, the strain CIP55.110 is able to expel the dye (black lane), and this transport is dependent of energy (violet lane) and compounds exhibit different levels of efflux inhibition. To better compare the efficiency of compounds, we performed a dose-response assay ranging from 200 to 12.5 μM; the results are summarized in Figure 3B. We observed that inhibition mainly concerns compounds 1–4 and that no inhibition was found in our conditions, below 25 μM. In addition, we observed a significant increase of inhibition, from 25 to 200 μM for compounds 1–4. At 100 μM, the dye efflux was inhibited by 50% by using Cpd**1** and Cpd**2**, whereas Cpd**3** and Cpd**4** led to a 30 and 17% efflux inhibition, respectively. Under similar experimental conditions, Cpd**5–7** did not present any significant effect.

**FIGURE 4:**
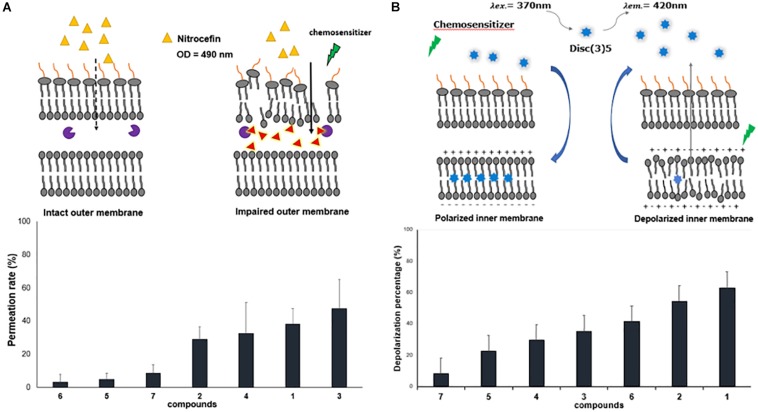
Effect of polyamine derivatives on membrane functions. **(A)** Outer membrane permeabilization by the different compounds at 100 μM. Nitrocefin hydrolysis was followed at 490 nm. **(B)** Inner membrane depolarization measured by the increase fluorescence of DisC_3_(5) in the presence of the different compounds at 100 μM. Error bars represent standard deviations of three independent biological experiments.

The other parameter that could modulate the antibiotic intracellular concentration is the outer membrane permeability. Thus, we evaluated the permeabilization activity of the polyamino-isoprenyl derivatives by using a previously reported nitrocefin hydrolysis method (15). We took advantage of the isolate 77-A12 that produces a β-lactamase (not shown). Interestingly, geranyl compounds (Cpd**5–**Cpd**7**) did not exhibit any permeabilization activity while farnesyl ones (Cpd**1**–Cpd**4**) increased the permeability of the outer membrane from 30 to 50%, considering that a maximum rate of permeation was obtained by using polymyxin B (100 μM) (Figure 4A). However, no influence of the amine structure was detected, with Cpd**4** (branched amine) presenting a similar activity with respect to the three other farnesyl compounds bearing a linear amine.

In Gram-negative bacteria, the PMF drives many of the efflux pumps and inner membrane transporters, and our compounds are able to inhibit efflux of the fluorescent dye used (Figure 3). Consequently, it is interesting to determine if the derivatives could alter the PMF across the inner membrane of *B. bronchiseptica*. As shown in Figure 4B, addition of polyamino-isoprenyl derivatives leads to an increase of the level of fluorescence that reflects the decrease of the PMF. In this case, Cpd**1** and Cpd**2** cause the most important depolarization at about 60% of the inner membrane. Surprisingly, derivatives Cpd**6** and Cpd**4** were also able to depolarize the inner membrane up to 40%. Nevertheless, we observe a slight effect for Cpd**5** and Cp**7**, which depolarized the inner membrane up to 20 and 10%, respectively. Molecules responsible for the most important depolarization remained the farnesyl ones, while the geranyl compounds displayed a lower rate of depolarization. In addition, the presence of a branched amine on the farnesyl moiety greatly influenced the activity (compare Cpd**1**/Cpd**4** and Cpd**2**/Cpd**4**) except for Cpd**3**, but one must consider that it is due to the presence of only three amino groups instead of Cpd**4**, as it is encountered for the other compounds. Thus, conversely to the results obtained from the outer membrane permeation assays, herein the activity depends on the isoprenyl moiety not only of the compound but also of the polyamine structure.

### Cpd1 and Motility Inhibition

As the derivatives can largely impair the PMF and particularly Cpd**1**, we were wondering if this compound could influence the flagella-driven motility that is highly dependent from the energy triggered by the PMF. We tested here the ability of Cpd**1** to inhibit the swimming and swarming of two strains of *B. bronchiseptica*, the CIP 55.110, which is susceptible to florfenicol, and the SR11-14, which is resistant both to florfenicol and to the combination florfenicol/Cpd**1**. Thus, we measured the diameter of the motility halo after 24 h of incubation at 25°C to assess the functioning of the flagella.

**FIGURE 5:**
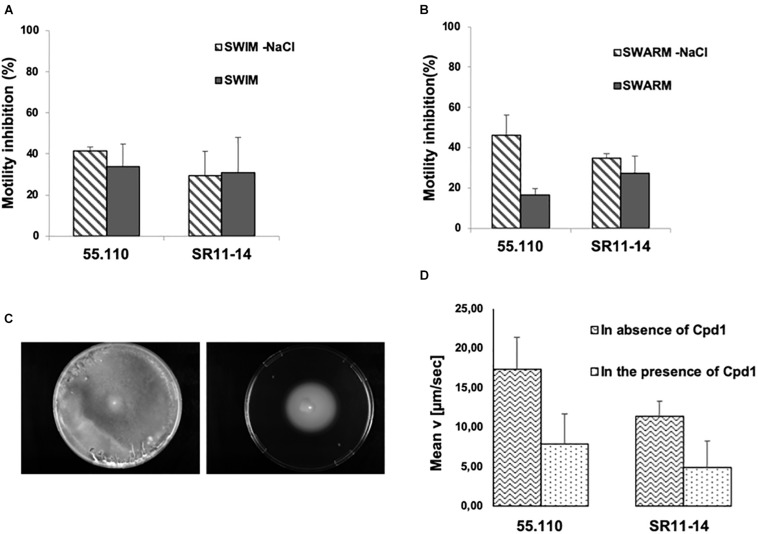
Effect of 10 μM of the hit compound on bacterial motility of a susceptible strain (CIP55.110) and a florfenicol resistant isolate (SR11-14) ± NaCl. Graph represents the percentage of inhibition of the motility halo in the presence of Cpd**1**. **(A)** Picture taken after 24 h incubation of the swimming plate. **(B)** Swarming motility, halo measured after 48 h incubation. **(C)** Picture taken after 24 h of incubation of swimming plate with NaCl inoculated with SR11-14, containing no compound (left) and 10 μM of Cpd**1** (right). In all cases, error bars represent standard deviations of three independent experiments. **(D)** Motility inhibition for CIP55.110 and SR11-14 in the presence or absence of Cpd**1**.

The motility of each strain was evaluated in the presence and absence of Cpd**1** and/or NaCl by considering that the flagella could use both the proton and the sodium gradient. We observed that Cpd**1** inhibited the swimming motility at about 40% for both strains (Figure 5A), whereas a slight effect was encountered toward the swarming motility ranging from 20 and 30% inhibition depending on the considered strain (Figure 5B). Nevertheless, it is noteworthy that the swarming inhibition by Cpd**1** is stronger in the absence of NaCl (up to 40%, Figure 5B). To further characterize the motility inhibition by Cpd**1**, we performed a real-time assay by monitoring the motility speed of bacteria after extemporaneous addition of the compound under the microscope. As shown in Figure 5C, a dramatic decrease of speed motility was observed for both strains. The observed inhibition of motility for both strains CIP55.110 and SR11-14 that correlated with the phenotype observed previously strongly suggests that Cpd**1** inhibits flagella motilities by disrupting the PMF (Figure 5D).

### Time Effect on Bacterial Growth

**FIGURE 6:**
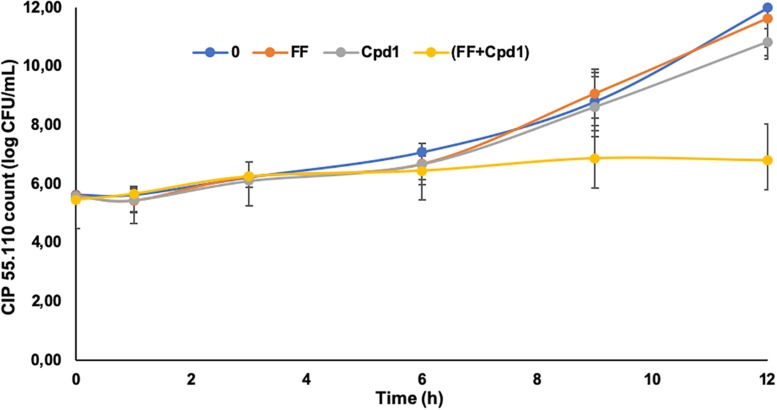
Time-kill curves of florfenicol (FF) (0.5 mg/L), Cpd**1** (7.5 μM), and the combination FF (0.5 mg/L) + Cpd**1** (7.5 μM) over a 12-h period against strain CIP55.110. Errors bars represent standard deviations of two independent biological experiments.

To further characterize the Cpd**1**/florfenicol combination, its *in vitro* pharmacodynamic (PD) parameters were evaluated using time-kill kinetics assays (Figure 6). Curves were obtained by plotting the number of CFU at every time point for the synergistic concentrations of the drugs.

As expected, the use of florfenicol at a sub-inhibitory concentration (MIC/4) had no effect on the cell growth such as the Cpd**1** used at a concentration of 7.5 μM, which corresponds to its MIC/32. Nevertheless, the combination of both totally inhibits the cell growth and, after 9 h of incubation, the reduction of bacterial cell count was around 2 Log compared to the bacteria incubated with florfenicol or Cpd**1** alone. After 12 h of incubation, the cell count was still reduced of 4 Log demonstrating a significant effect of the combination compared to the same antimicrobials administered alone (Figure 6). The combination florfenicol/Cpd**1** appeared to be bacteriostatic as florfenicol used alone.

### Florfenicol Resistance Mechanism Investigation

Two of the strains included in our study (42F10 and SR11-14) exhibited resistance to florfenicol (MIC = 8 and 32 μg/ml, respectively). Such resistance is not common but has already been described and the *floR* gene was identified in some isolates as responsible for this resistance (Kadlec et al., 2007; Kadlec and Schwarz, 2018). To check if *floR* could be associated with florfenicol resistance in these two strains, we designed primers to amplify the gene according to the sequence previously described in the study of Kadlec et al. (2007). No amplification was detected despite several attempts, suggesting that another mechanism of resistance was involved in these strains. Taken together, our data suggested that another mechanism was involved in florfenicol resistance in these two strains. We thus decided to determine the complete sequenced genomes of those two strains, as well as two other strains, our reference CIP55-110 and 77A12, which was resistant to β-lactams (SRA accession: PRJNA551886).

In order to be able to highlight a new gene of resistance or a *floR*-derived one, we mapped our two libraries made from resistant strains against reference strain *B. bronchiseptica* 253, by using BWA MeM software (version 0.7.16a-r1181) with a minimum seed length of 28; all other options were kept to standard. Owing to the fact that this strain does not have resistance to florfenicol, we selected reads that did not map to this strain. We reconstructed *de novo* the two other strains CIP55-110 and 77H12 with SPAdes version 3.9.1 (Bankevich et al., 2012) and we mapped the remaining reads to the obtained contigs with the same goal. We ended with 581 k-reads and 377 k-reads for SR11-14 and 42F10, respectively (Supplementary Material). We reconstructed *de novo* contigs with those remaining reads, keeping in mind the hypothesis that genes responsible for florfenicol resistance would be present in such contigs. We ended with 416 contigs and 331 contigs, respectively, that were mapped against all the florfenicol and/or chloramphenicol resistance associated genes published until now (634 sequences). Unfortunately, we did not find any match.

As a last attempt, considering that protein sequence is better preserved than nucleotide sequence, we performed a blastx of all our contigs against all the available florfenicol resistance-related protein sequences, but we were unsuccessful. Genes associated with resistance to antibiotics specifically detected in those two strains were found, indicating that our pipeline was able to detect genes present only in those strains and not in the reference and non-resistant ones (sul2 in SR11-14 and TEM-116 in 42F10). Interestingly, genes sharing homology with ABC transporters and OptrA (Tamang et al., 2017), which have been described to be associated with florfenicol resistance in *Enterococcus faecium*, have been detected co-localized on same contigs, which also exhibit plasmid associated features. Interestingly, OptrA was shown to afford cross-resistance to chloramphenicol and florfenicol in *E. faecium* (Wang et al., 2015; Bender et al., 2018; Na et al., 2019) that can be correlated with the cross-resistance to these two antibiotics in the two strains studied herein (see Supplementary Table 1).

## Discussion

The international and European authorities requested to use with parsimony antibiotics that can favor emergence of resistance in human and veterinary medicine. For the first time, an alternate way is studied by using adjuvant molecules that enhance florfenicol antibiotic activity. This strategy allowed us to decrease by four the MIC of the majority of strains tested here with small amounts of polyamino-isoprenyl derivatives, previously known to potentiate antibiotics by acting on membrane functions (Blanchet et al., 2016). Thus, even if florfenicol is only used to treat animal infections, it presents cross-resistance with chloramphenicol, and it appears important to prevent the spread to human pathogens.

Today, it is recognized that human health is directly related to animal health and the environment. The dramatic increase in antibiotic resistance and the scarcity of new drugs on the market in recent decades highlight the threat of moving to the past era of antibiotics, with a significant increase in infection mortality. In this context, taking into account the “One Health” problem becomes an urgent need. This must allow collaborative efforts between health actors, as well as breeders and those involved in environmental management.

This includes the involvement of a research at the interface between chemistry and microbiology, to develop new infection control strategies, by developing molecules that target specific new bacterial processes and/or new molecules that block/inhibit resistance mechanisms already under way. In recent decades, the efflux mechanisms have become increasingly important for human and animal health, and we propose to block the mechanisms of resistance by efflux of Gram-negative bacteria.

According to our previous work (Borselli et al., 2016), we evaluated enhancing activity of our polyamino-isoprenyl chemical library (used at a 10 μM concentration) in the presence of florfenicol at a sub-inhibitory concentration against *B. bronchiseptica*, CIP 55.110 reference strain. Thus, Cpd**1** was identified as an interesting chemosensitizer of *B. bronchiseptica* to florfenicol and evaluated against 10 animal isolates leading to the restoration of susceptibility for 8 of these 10 strains. Two isolates were resistant to the combination, and the mechanism of resistance involved remains unknown.

Subsequently, a pharmacomodulation study was performed to try to improve the encountered activity. In this context, six derivatives, three geranyl ones, and their corresponding farnesyl homologs were prepared and evaluated in a checkerboard assay. First, we noticed that Cpd**1** remained the better-tested compound, needing the lowest concentration to potentiate florfenicol activity. It is also noteworthy that all farnesyl compounds showed better efficiency than geranyl ones. As polyamines are well-known agents to disrupt membranes (Blanchet et al., 2016), we investigated the effect of the compounds on membrane physiology and stability. We investigated the permeability of the outer membrane, which plays a key role in antibiotic accumulation into Gram-negative bacteria, and as expected, all the tested polyamino-isoprenyl compounds increase the outer membrane permeability, with farnesyl compounds being better permeabilizers than geranyl ones.

The presence of an active efflux in *B. bronchiseptica* was demonstrated by using CCCP, allowing an efficient dye loading into the bacterial membranes. These results corroborate the genome sequence analysis from *B. pertussis* (a human and animal pathogen closely related to *B. bronchiseptica*), suggesting the presence of putative RND efflux pump coding sequences (Andrade et al., 2014). To our knowledge, it is also the first time that efflux was determined in *B. bronchiseptica*. Thereby, we evaluated the effect of Cpd**1** and its derivatives on the efflux activity of *B. bronchiseptica.* The molecules able to inhibit the dye efflux were the farnesyl ones, while the geranyl derivatives demonstrated no ability to block the efflux activity [Cpd**1** and Cpd**4** leading to 50% inhibition (used at 100 μM)]. Moreover, we observed a dose-response effect for each farnesyl compound as encountered in a previous work on *Pseudomonas* (Borselli et al., 2016). It also appeared that the farnesyl moiety was essential to obtain high activities. All these data led us to envision that these compounds could act against PMF, which is required for many cell processes, as efflux pump systems but also the flagella motors.

The flagella that potentially contribute to virulence could be considered as a new target for the development of motility inhibitors to circumvent the spread of bacterial infections (Malapaka et al., 2007). The effect of Cpd**1** was studied, and a significant inhibition on the flagella-dependent motilities was observed. Nevertheless, since *B. bronchiseptica* can also use the sodium gradient, we were unable to discriminate if Cpd**1** targets preferentially the proton or sodium gradient. We concluded that this effect is probably due to a general inhibition. On the other hand, we noticed that the inhibition of motility occurred in an independent way of the resistance level of the strain. Indeed, a resistant strain (SR11-14) and the reference strain CIP55.110 gave the same response to inhibition of motility after treatment with Cpd**1**. More interestingly, the study of the resistance mechanism of these strains by bioinformatic investigations showed the presence of a gene sharing homologies with the gene coding the ABC-F type ribosomal protection protein OptrA, known to confer florfenicol and chloramphenicol resistance in *E. faecium* and to use ATP as a source of energy (Wang et al., 2015; Bender et al., 2018; Na et al., 2019). This is consistent with our results demonstrating that Cpd**1** targets the PMF and correlates with the absence of efficacy toward the strains exhibiting this mechanism of resistance. In the strain SR 11-14, one might consider that OptrA and/or efflux pumps are overexpressed in this strain. We can hypothesize that florfenicol resistance could have been acquired through horizontal transfer from *Enterococcus*, and this hypothesis is strengthened by the fact that the contig containing resistance-associated genes showed high homology with plasmids found in such bacteria.

Finally, to better characterize the antibiotic/derivative combination, we did time-kill assays at the concentrations that have shown a synergistic effect. This florfenicol/Cpd**1** combination reduces the antibiotic efficient concentration four times and thus could allow drastically reducing the amount of the antibiotic used. Because Cpd**1** did not modify the bacteriostatic activity of florfenicol, but increased its efficiency, one may consider that Cpd**1** allows an increase of the intracellular concentration of the antibiotic (Vergalli et al., 2018). Experiments are in progress to evaluate this hypothesis and results will be reported in due course.

## Data Availability

All datasets generated for this study are included in the manuscript and/or the Supplementary Files.

## Author Contributions

All authors conceived the approach and reviewed the manuscript. DB carried out all the experiments related to bacteria. JBr carried out the synthesis, purification, and analyses of the compounds. OG performed the DNA sequencing and the sequence analyses. DB, JBr, and JBo wrote the main manuscript text. DB and JBr prepared the figures.

## Conflict of Interest Statement

The authors declare that the research was conducted in the absence of any commercial or financial relationships that could be construed as a potential conflict of interest.

## Footnotes

1 https://bi.ema.europa.eu/analyticsSOAP/saw.dll?PortalPages
