# Supplementary material for: Polyamino-Isoprenyl Derivatives as Antibiotic Adjuvants and Motility Inhibitors for Bordetella bronchiseptica Porcine Pulmonary Infection Treatment
Source: Front Microbiol. 2019 Aug 13;10:1771. doi: 10.3389/fmicb.2019.01771 (PMC6700233; doi:10.3389/fmicb.2019.01771)
Supplement: Supplementary file 1 [file Table_1.DOCX]

| **Strain** | **FFC*** | **CHL*** |
| --- | --- | --- |
| 55.110 | S | S |
| 77-A12 | S | S |
| 42-F10 | R | I |
| SR11-14 | R | R |

Susceptibility to phenicols of four strains selected from this study.

FFC : florfenicol; CHL Chloramphenicol

(*) Susceptible, Intermediate and Resistant were determined according to the NCCLS recommendation :

Clinical and Laboratory Standards Institute, *Performance Standards for Antimicrobial Disk and Dilution Susceptibility Tests for Bacteria Isolated From Animals—Third Edition: Approved Standard M31-A3*, **2008** Wayne, PA, USACLSI
