# Supplementary material for: Polyamino-Isoprenyl Derivatives as Antibiotic Adjuvants and Motility Inhibitors for Bordetella bronchiseptica Porcine Pulmonary Infection Treatment
Source: Front Microbiol. 2019 Aug 13;10:1771. doi: 10.3389/fmicb.2019.01771 (PMC6700233; doi:10.3389/fmicb.2019.01771)
Supplement: Supplementary file 2 [file Table_2.DOCX]

**Supplementary Material 1: Data Analysis of compounds 1-7**

**Compound 1**, 72% yield; Yellow solid; ^1^H NMR (MeOD, 250 MHz): *δ* = 5.05-4.93 (m, 3H), 2.93-2.57 (m, 14H), 2.19-1.92 (m, 10H), 1.63-1.87 (m, 23H). ^13^C (MeOD): *δ* = 142.24, 134.83, 131.09, 124.86, 124.17, 117.32, 47.90, 47.69, 47.64, 46.61, 44.01, 40.60, 39.83, 33.97, 31.20, 26.97, 26.30, 25.66, 25.11, 24.90, 17.62, 16.90, 15.93. C_25_H_50_N_4_ MS (ESI+) m/z 407.41 (100%, [M + H]^+^).

**Compound 2.** 49% yield; Yellow oil; ^1^H NMR (MeOD, 250 MHz): *δ* = 5.33-5.38 (m, 1H), 5.15-5.21 (m, 2H), 3.22-3.24 (m, 2H), 2.63-2.72 (m, 2H), 2.49-2.59 (m, 10H), 1.59-1.92 (m, 31 H) . ^13^C (MeOD): *δ* = 141.16, 135.71, 132.01, 124.30, 123.51, 120.56, 47.41, 46.32, 45.32, 39.71, 39.41, 33.25, 31.92, 27.35, 26.74, 24.12, 22.10, 18.68, 16.03. C_24_H_48_N_4_ MS (ESI+) m/z 393.39 (100%, [M + H]^+^).

**Compound 3.** 41% yield; Yellow oil; ^1^H NMR (MeOD, 250 MHz): *δ* = 5.34-5.38 (m, 1H), 5.16-5.21 (m, 2H), 3.32 (s, 1H), 3.22-3.26 (m, 2H), 2.63-2.69 (m, 2H), 2.49-2.55 (m, 6H), 2.02-2.06 (m, 8H), 1.59-2.00 (m, 19H) . ^13^C (MeOD): *δ* = 141.66, 135.71, 132.11, 124.30, 123.51, 120.51, 47.41, 46.31, 46.02, 45.21, 39.69, 39.41, 33.19, 31.89, 26.74, 24.62, 22.00, 18.78, 16.43. C_21_H_41_N_3_ MS (ESI+) m/z 336.33 (100%, [M + H]^+^).

**Compound 4.** 52% yield; Yellow oil; ^1^H NMR (MeOD, 250 MHz): *δ* = 5.34-5.38 (m, 1H), 5.16-5.20 (m, 2H), 3.34 (s, 1H), 3.22-3.26 (m, 2H), 2.39-2.50 (m, 12H), 2.02-2.04 (m, 8H), 1.52-1.79 (m, 16H) . ^13^C (MeOD): *δ* = 141.56, 136.01, 132.01, 124.32, 123.50, 120.41, 59.91, 58.02, 47.21, 45.31, 39.69, 38.82, 33.42, 26.64, 24.52, 21.95, 18.78, 16.41. C_21_H_42_N_4_ MS (ESI+) m/z 351.34 (100%, [M + H]^+^).

**Compound 5.** 62% yield; Yellow oil; ^1^H NMR (MeOD, 250 MHz): *δ* = 5.33-5.38 (m, 1H), 5.16-5.22 (m, 1H), 3.32 (s, 1H), 3.22-3.26 (m, 2H), 2.60-2.68 (m, 2H), 2.50-2.55 (m, 6H), 2.02-2.04 (m, 4H), 1.52-1.79 (m, 16H) . ^13^C (MeOD): *δ* = 141.58, 132.01, 123.52, 120.51, 47.41, 46.31, 46.01, 45.32, 39.43, 31.92, 26.44, 24.32, 18.78, 16.11. C_16_H_33_N_3_ MS (ESI+) m/z 268.27 (100%, [M + H]^+^).

**Compound 6.** 32% yield; Yellow oil; ^1^H NMR (MeOD, 250 MHz): *δ* = 5.34-5.38 (m, 1H), 5.17-5.22 (m, 1H), 3.29 (s, 1H), 3.22-3.24 (m, 2H), 2.60-2.67 (m, 2H), 2.53-2.55 (m, 10H), 2.01-2.04 (m, 4H), 1.49-1.79 (m, 19H) . ^13^C (MeOD): *δ* = 141.68, 132.00, 123.32, 120.11, 47.41, 46.32, 45.39, 45.32, 39.46, 31.92, 27.33, 26.44, 24.42, 18.63, 16.09. C_19_H_40_N_4_ MS (ESI+) m/z 325.33 (100%, [M + H]^+^).

**Compound 7.** 46% yield; Yellow oil; ^1^H NMR (MeOD, 250 MHz): *δ* = 5.33-5.38 (m, 1H), 5.17-5.22 (m, 1H), 3.32 (s, 1H), 3.22-3.23 (m, 2H), 2.39-2.60 (m, 12H), 1.98-2.01 (m, 4H), 1.52-1.79 (m, 13H) . ^13^C (MeOD): *δ* = 141.62, 132.00, 123.54, 120.51, 59.95, 56.03, 47.21, 45.19, 45.32, 39.45, 38.82, 26.44, 24.62, 18.63, 16.19. C_16_H_34_N_4_ MS (ESI+) m/z 283.28 (100%, [M + H]^+^).
