# Supplementary material for: Polyamino-Isoprenyl Derivatives as Antibiotic Adjuvants and Motility Inhibitors for Bordetella bronchiseptica Porcine Pulmonary Infection Treatment
Source: Front Microbiol. 2019 Aug 13;10:1771. doi: 10.3389/fmicb.2019.01771 (PMC6700233; doi:10.3389/fmicb.2019.01771)
Supplement: Supplementary file 3 [file Table_3.DOCX]

**Supplementary Material 2: DNA Sequence analysis of Strain CIP 55-110**

**BLASTX 2.6.0+**

[**Reference**](https://www.ncbi.nlm.nih.gov/entrez/query.fcgi?db=PubMed&cmd=R%0aetrieve&list_uids=9254694&dopt=Citation)**:**

Stephen F. Altschul, Thomas L. Madden, Alejandro A. Schäffer,

Jinghui Zhang, Zheng Zhang, Webb Miller, and David J. Lipman (1997),

"Gapped BLAST and PSI-BLAST: a new generation of protein database

search programs", Nucleic Acids Res. 25:3389-3402.

Database: BdD_Prot-name.fasta

702 sequences; 261,934 total letters

**Query=** NODE_1_length_12749_cov_22.1806

Length=12749

Score E

Sequences producing significant alignments: (Bits) Value

YP_002286765 streptomycin phosphotransferase (plasmid) [Pasteur... [80.9](file:///Users/JMBNew/Desktop/Articles%20en%20Cours/Article%20Diane/Bb_42-1_output-blast_named.html#BL_ORD_ID:608) 2e-17

> YP_002286765 streptomycin phosphotransferase (plasmid) [Pasteurella

multocida]

Length=267

Score = 80.9 bits (198), Expect = 2e-17, Method: Compositional matrix adjust.

Identities = 77/258 (30%), Positives = 115/258 (45%), Gaps = 23/258 (9%)

Frame = -1

Query 10847 WARDKVGQSGCAVYRLHSKSGGSDLFLKHGKDAFADDVTDEMVRLRWLAGH-ISVPSVVS 10671

W + G+SG V+R G F K + ++ E RL WL G ++ P V++

Sbjct 15 WLPVRGGESGDFVFR----RGDGHAFAKIAPASRRGELAGERDRLIWLKGRGVACPEVIN 70

Query 10670 FVRTPNQAWLLTTAIHGKTAYQVLKSDFGARLVVVDALAAFMRRLHAIPVSECSFNSDHA 10491

+ A L+ TAI G A + +D L ++ + +H++ V +C F +

Sbjct 71 WQEEQEGACLVITAIPGVPAADLSGADL---LKAWPSMGQQLGAVHSLSVDQCPFERRLS 127

Query 10490 CRLARARERIEAGVVDVDDFDKEREGWTAEQVWEAMHRLLPLA-----PDPVVTHGDFSL 10326

RA + + V+ D E + + + R LP+ D VV HGD +

Sbjct 128 RMFGRAVDVVSRNAVNPDFLPDEDKSTPQLDLLARVERELPVRLDQERTDMVVCHGDPCM 187

Query 10325 DNLLIVEGKVVGC---IDVGRAGIADRYQDLAVLWNCLEE--FEPSLQER----LVAQYG 10173

N + V+ K + C ID+GR G ADRY DLA++ EE P ER L G

Sbjct 188 PNFM-VDPKTLQCTGLIDLGRLGTADRYADLALMIANAEENWAAPDEAERAFAVLFNVLG 246

Query 10172 IADPDRRKLQFHLLLDEL 10119

I PDR +L F+L LD L

Sbjct 247 IEAPDRERLAFYLRLDPL 264

Lambda K H a alpha

0.318 0.134 0.401 0.792 4.96

Gapped

Lambda K H a alpha sigma

0.267 0.0410 0.140 1.90 42.6 43.6

Effective search space used: 817269088

**Query=** NODE_2_length_10442_cov_16.4818

Length=10442

***** No hits found *****

Lambda K H a alpha

0.318 0.134 0.401 0.792 4.96

Gapped

Lambda K H a alpha sigma

0.267 0.0410 0.140 1.90 42.6 43.6

Effective search space used: 671198228

**Query=** NODE_3_length_7187_cov_15.2603

Length=7187

***** No hits found *****

Lambda K H a alpha

0.318 0.134 0.401 0.792 4.96

Gapped

Lambda K H a alpha sigma

0.267 0.0410 0.140 1.90 42.6 43.6

Effective search space used: 459945536

**Query=** NODE_4_length_7004_cov_20.1219

Length=7004

***** No hits found *****

Lambda K H a alpha

0.318 0.134 0.401 0.792 4.96

Gapped

Lambda K H a alpha sigma

0.267 0.0410 0.140 1.90 42.6 43.6

Effective search space used: 447778720

**Query=** NODE_5_length_5230_cov_22.2998

Length=5230

Score E

Sequences producing significant alignments: (Bits) Value

AKL82310 tetracycline resistance protein, partial [Escherichia ... [248](file:///Users/JMBNew/Desktop/Articles%20en%20Cours/Article%20Diane/Bb_42-1_output-blast_named.html#BL_ORD_ID:200) 3e-77

AKL82311 tetracycline resistance protein, partial [Escherichia ... [246](file:///Users/JMBNew/Desktop/Articles%20en%20Cours/Article%20Diane/Bb_42-1_output-blast_named.html#BL_ORD_ID:201) 8e-77

YP_004799997 florfenicol export protein (plasmid) [Riemerella a... [43.9](file:///Users/JMBNew/Desktop/Articles%20en%20Cours/Article%20Diane/Bb_42-1_output-blast_named.html#BL_ORD_ID:617) 2e-05

WP_014043449 chloramphenicol/florfenicol efflux MFS transporter... [43.9](file:///Users/JMBNew/Desktop/Articles%20en%20Cours/Article%20Diane/Bb_42-1_output-blast_named.html#BL_ORD_ID:555) 2e-05

AEM66531 florfenicol export protein (plasmid) [Riemerella anati... [43.9](file:///Users/JMBNew/Desktop/Articles%20en%20Cours/Article%20Diane/Bb_42-1_output-blast_named.html#BL_ORD_ID:76) 2e-05

EYH62156 putative chloramphenicol and florfenicol resistance pr... [41.2](file:///Users/JMBNew/Desktop/Articles%20en%20Cours/Article%20Diane/Bb_42-1_output-blast_named.html#BL_ORD_ID:465) 1e-04

OAQ83863 florfenicol exporter [Purpureocillium lilacinum] [41.6](file:///Users/JMBNew/Desktop/Articles%20en%20Cours/Article%20Diane/Bb_42-1_output-blast_named.html#BL_ORD_ID:508) 1e-04

AEV96403 florfenicol/chloramphenicol resistance protein, partia... [40.0](file:///Users/JMBNew/Desktop/Articles%20en%20Cours/Article%20Diane/Bb_42-1_output-blast_named.html#BL_ORD_ID:92) 1e-04

ACJ65218 putative florfenicol exporter, partial [Vibrio cholerae] [40.0](file:///Users/JMBNew/Desktop/Articles%20en%20Cours/Article%20Diane/Bb_42-1_output-blast_named.html#BL_ORD_ID:50) 1e-04

XP_018179360 florfenicol exporter [Purpureocillium lilacinum] [41.2](file:///Users/JMBNew/Desktop/Articles%20en%20Cours/Article%20Diane/Bb_42-1_output-blast_named.html#BL_ORD_ID:594) 1e-04

OAQ90641 florfenicol exporter [Purpureocillium lilacinum] [41.2](file:///Users/JMBNew/Desktop/Articles%20en%20Cours/Article%20Diane/Bb_42-1_output-blast_named.html#BL_ORD_ID:509) 1e-04

AAS16363 florfenicol export protein [Escherichia coli] [40.8](file:///Users/JMBNew/Desktop/Articles%20en%20Cours/Article%20Diane/Bb_42-1_output-blast_named.html#BL_ORD_ID:15) 2e-04

YP_005221014 putative chloramphenicol and florfenicol resistanc... [40.8](file:///Users/JMBNew/Desktop/Articles%20en%20Cours/Article%20Diane/Bb_42-1_output-blast_named.html#BL_ORD_ID:625) 2e-04

CAM88409 putative Chloramphenicol and florfenicol resistance pr... [40.8](file:///Users/JMBNew/Desktop/Articles%20en%20Cours/Article%20Diane/Bb_42-1_output-blast_named.html#BL_ORD_ID:346) 2e-04

CAJ77032 chloramphenicol and florfenicol resistance protein [Ac... [40.8](file:///Users/JMBNew/Desktop/Articles%20en%20Cours/Article%20Diane/Bb_42-1_output-blast_named.html#BL_ORD_ID:339) 2e-04

ASR82671 chloramphenicol and florfenicol resistance protein (pl... [40.8](file:///Users/JMBNew/Desktop/Articles%20en%20Cours/Article%20Diane/Bb_42-1_output-blast_named.html#BL_ORD_ID:308) 2e-04

ASK37999 chloramphenicol and florfenicol resistance protein (pl... [40.8](file:///Users/JMBNew/Desktop/Articles%20en%20Cours/Article%20Diane/Bb_42-1_output-blast_named.html#BL_ORD_ID:307) 2e-04

ASJ23722 chloramphenicol and florfenicol resistance protein [La... [40.8](file:///Users/JMBNew/Desktop/Articles%20en%20Cours/Article%20Diane/Bb_42-1_output-blast_named.html#BL_ORD_ID:305) 2e-04

AGT26821 chloramphenicol and florfenicol resistance protein (Cm... [40.8](file:///Users/JMBNew/Desktop/Articles%20en%20Cours/Article%20Diane/Bb_42-1_output-blast_named.html#BL_ORD_ID:141) 2e-04

AEW92275 putative chloramphenicol and florfenicol resistance pr... [40.8](file:///Users/JMBNew/Desktop/Articles%20en%20Cours/Article%20Diane/Bb_42-1_output-blast_named.html#BL_ORD_ID:95) 2e-04

ABZ01840 CmlA9 chloramphenicol efflux protein [Salmonella enter... [40.8](file:///Users/JMBNew/Desktop/Articles%20en%20Cours/Article%20Diane/Bb_42-1_output-blast_named.html#BL_ORD_ID:41) 2e-04

floR_-_13918086_translation floR: florfenicol-chloramphenicol e... [40.4](file:///Users/JMBNew/Desktop/Articles%20en%20Cours/Article%20Diane/Bb_42-1_output-blast_named.html#BL_ORD_ID:670) 2e-04

YP_006961358 florfenicol-chloramphenicol exporter (plasmid) [Ma... [40.4](file:///Users/JMBNew/Desktop/Articles%20en%20Cours/Article%20Diane/Bb_42-1_output-blast_named.html#BL_ORD_ID:631) 2e-04

BAL04183 florfenicol-chloramphenicol exporter (plasmid) [Mannhe... [40.4](file:///Users/JMBNew/Desktop/Articles%20en%20Cours/Article%20Diane/Bb_42-1_output-blast_named.html#BL_ORD_ID:316) 2e-04

BAC79058 florfenicol exporter [Vibrio cholerae] [40.4](file:///Users/JMBNew/Desktop/Articles%20en%20Cours/Article%20Diane/Bb_42-1_output-blast_named.html#BL_ORD_ID:312) 2e-04

AAL59749 florfenicol exporter [Vibrio cholerae] [40.4](file:///Users/JMBNew/Desktop/Articles%20en%20Cours/Article%20Diane/Bb_42-1_output-blast_named.html#BL_ORD_ID:5) 2e-04

AAK64587 florfenicol exporter [Vibrio cholerae MO10] [40.4](file:///Users/JMBNew/Desktop/Articles%20en%20Cours/Article%20Diane/Bb_42-1_output-blast_named.html#BL_ORD_ID:2) 2e-04

AHG85140 Florfenicol export protein [Bibersteinia trehalosi USD... [40.4](file:///Users/JMBNew/Desktop/Articles%20en%20Cours/Article%20Diane/Bb_42-1_output-blast_named.html#BL_ORD_ID:151) 2e-04

AGH37387 Florfenicol export protein [Bibersteinia trehalosi USD... [40.4](file:///Users/JMBNew/Desktop/Articles%20en%20Cours/Article%20Diane/Bb_42-1_output-blast_named.html#BL_ORD_ID:125) 2e-04

AHG85541 Florfenicol export protein [Bibersteinia trehalosi USD... [40.4](file:///Users/JMBNew/Desktop/Articles%20en%20Cours/Article%20Diane/Bb_42-1_output-blast_named.html#BL_ORD_ID:152) 2e-04

AET15214 florfenicol / chloramphenicol efflux protein [Pasteure... [40.4](file:///Users/JMBNew/Desktop/Articles%20en%20Cours/Article%20Diane/Bb_42-1_output-blast_named.html#BL_ORD_ID:88) 2e-04

AKJ66278 FloR (plasmid) [[Haemophilus] parasuis] [40.4](file:///Users/JMBNew/Desktop/Articles%20en%20Cours/Article%20Diane/Bb_42-1_output-blast_named.html#BL_ORD_ID:194) 2e-04

floR_-_7886606_translation floR: FloR [40.4](file:///Users/JMBNew/Desktop/Articles%20en%20Cours/Article%20Diane/Bb_42-1_output-blast_named.html#BL_ORD_ID:656) 2e-04

YP_002894576 FloR (plasmid) [Salmonella enterica] [40.4](file:///Users/JMBNew/Desktop/Articles%20en%20Cours/Article%20Diane/Bb_42-1_output-blast_named.html#BL_ORD_ID:613) 2e-04

AF231986_1 florfenicol exporter (plasmid) [Escherichia coli] [40.4](file:///Users/JMBNew/Desktop/Articles%20en%20Cours/Article%20Diane/Bb_42-1_output-blast_named.html#BL_ORD_ID:103) 2e-04

ACQ77823 FloR (plasmid) [Salmonella enterica] [40.4](file:///Users/JMBNew/Desktop/Articles%20en%20Cours/Article%20Diane/Bb_42-1_output-blast_named.html#BL_ORD_ID:54) 2e-04

AAS16362 florfenicol export protein [Escherichia coli] [40.4](file:///Users/JMBNew/Desktop/Articles%20en%20Cours/Article%20Diane/Bb_42-1_output-blast_named.html#BL_ORD_ID:14) 2e-04

AF252855_1 florfenicol-resistance protein Flo [Escherichia coli] [40.4](file:///Users/JMBNew/Desktop/Articles%20en%20Cours/Article%20Diane/Bb_42-1_output-blast_named.html#BL_ORD_ID:104) 2e-04

AAW02923 florfenicol efflux protein (plasmid) [Escherichia coli] [40.4](file:///Users/JMBNew/Desktop/Articles%20en%20Cours/Article%20Diane/Bb_42-1_output-blast_named.html#BL_ORD_ID:21) 2e-04

FloR_-_24955634_translation FloR: florfenicol chloramphenicol r... [40.4](file:///Users/JMBNew/Desktop/Articles%20en%20Cours/Article%20Diane/Bb_42-1_output-blast_named.html#BL_ORD_ID:675) 2e-04

YP_008574689 florfenicol chloramphenicol resistance protein Flo... [40.4](file:///Users/JMBNew/Desktop/Articles%20en%20Cours/Article%20Diane/Bb_42-1_output-blast_named.html#BL_ORD_ID:636) 2e-04

CAD57670 florfenicol/chloramphenicol resistance protein (plasmi... [40.4](file:///Users/JMBNew/Desktop/Articles%20en%20Cours/Article%20Diane/Bb_42-1_output-blast_named.html#BL_ORD_ID:324) 2e-04

BAN84125 florfenicol chloramphenicol resistance protein FloR (p... [40.4](file:///Users/JMBNew/Desktop/Articles%20en%20Cours/Article%20Diane/Bb_42-1_output-blast_named.html#BL_ORD_ID:318) 2e-04

AAS00447 florfenicol export protein [Escherichia coli] [40.4](file:///Users/JMBNew/Desktop/Articles%20en%20Cours/Article%20Diane/Bb_42-1_output-blast_named.html#BL_ORD_ID:13) 2e-04

floR_-_11934756_translation floR: florfenicol/chloramphenicol r... [40.0](file:///Users/JMBNew/Desktop/Articles%20en%20Cours/Article%20Diane/Bb_42-1_output-blast_named.html#BL_ORD_ID:660) 2e-04

AIF79467 FloR (plasmid) [Escherichia coli] [40.0](file:///Users/JMBNew/Desktop/Articles%20en%20Cours/Article%20Diane/Bb_42-1_output-blast_named.html#BL_ORD_ID:164) 2e-04

SFV91173 Chloramphenicol and florfenicol resistance transporter... [40.0](file:///Users/JMBNew/Desktop/Articles%20en%20Cours/Article%20Diane/Bb_42-1_output-blast_named.html#BL_ORD_ID:535) 2e-04

ELX29704 florfenicol exporter [Salmonella enterica subsp. enter... [40.0](file:///Users/JMBNew/Desktop/Articles%20en%20Cours/Article%20Diane/Bb_42-1_output-blast_named.html#BL_ORD_ID:455) 2e-04

CCW76557 chloramphenicol and florfenicol resistance protein [Sa... [40.0](file:///Users/JMBNew/Desktop/Articles%20en%20Cours/Article%20Diane/Bb_42-1_output-blast_named.html#BL_ORD_ID:371) 2e-04

AQY10149 chloramphenicol and florfenicol resistance protein [Sa... [40.0](file:///Users/JMBNew/Desktop/Articles%20en%20Cours/Article%20Diane/Bb_42-1_output-blast_named.html#BL_ORD_ID:280) 2e-04

ANA09865 chloramphenicol and florfenicol resistance protein [Sa... [40.0](file:///Users/JMBNew/Desktop/Articles%20en%20Cours/Article%20Diane/Bb_42-1_output-blast_named.html#BL_ORD_ID:236) 2e-04

AMQ95871 FloR [Salmonella enterica subsp. enterica serovar Cerro] [40.0](file:///Users/JMBNew/Desktop/Articles%20en%20Cours/Article%20Diane/Bb_42-1_output-blast_named.html#BL_ORD_ID:233) 2e-04

AMP35663 FloR [Salmonella enterica] [40.0](file:///Users/JMBNew/Desktop/Articles%20en%20Cours/Article%20Diane/Bb_42-1_output-blast_named.html#BL_ORD_ID:228) 2e-04

AIW55480 chloramphenicol and florfenicol resistance protein [Pr... [40.0](file:///Users/JMBNew/Desktop/Articles%20en%20Cours/Article%20Diane/Bb_42-1_output-blast_named.html#BL_ORD_ID:180) 2e-04

AIW55431 chloramphenicol and florfenicol resistance protein [Pr... [40.0](file:///Users/JMBNew/Desktop/Articles%20en%20Cours/Article%20Diane/Bb_42-1_output-blast_named.html#BL_ORD_ID:179) 2e-04

AIU97987 chloramphenicol and florfenicol resistance protein [Sa... [40.0](file:///Users/JMBNew/Desktop/Articles%20en%20Cours/Article%20Diane/Bb_42-1_output-blast_named.html#BL_ORD_ID:178) 2e-04

AGK07102 chloramphenicol and florfenicol resistance protein [Pr... [40.0](file:///Users/JMBNew/Desktop/Articles%20en%20Cours/Article%20Diane/Bb_42-1_output-blast_named.html#BL_ORD_ID:129) 2e-04

AGK06972 chloramphenicol and florfenicol resistance protein [Pr... [40.0](file:///Users/JMBNew/Desktop/Articles%20en%20Cours/Article%20Diane/Bb_42-1_output-blast_named.html#BL_ORD_ID:128) 2e-04

AF261825_18 chloramphenicol and florfenicol resistance protein ... [40.0](file:///Users/JMBNew/Desktop/Articles%20en%20Cours/Article%20Diane/Bb_42-1_output-blast_named.html#BL_ORD_ID:105) 2e-04

AF118107_2 putative efflux protein Flor [Salmonella enterica su... [40.0](file:///Users/JMBNew/Desktop/Articles%20en%20Cours/Article%20Diane/Bb_42-1_output-blast_named.html#BL_ORD_ID:101) 2e-04

AMQ12815 FloR (plasmid) [Escherichia coli] [40.0](file:///Users/JMBNew/Desktop/Articles%20en%20Cours/Article%20Diane/Bb_42-1_output-blast_named.html#BL_ORD_ID:232) 2e-04

ABR22528 florfenicol resistance protein, partial [Vibrio tasman... [39.7](file:///Users/JMBNew/Desktop/Articles%20en%20Cours/Article%20Diane/Bb_42-1_output-blast_named.html#BL_ORD_ID:36) 2e-04

YP_002286768 phenicol exporter (plasmid) [Pasteurella multocida] [40.0](file:///Users/JMBNew/Desktop/Articles%20en%20Cours/Article%20Diane/Bb_42-1_output-blast_named.html#BL_ORD_ID:610) 2e-04

WP_012561099 MULTISPECIES: chloramphenicol/florfenicol efflux M... [40.0](file:///Users/JMBNew/Desktop/Articles%20en%20Cours/Article%20Diane/Bb_42-1_output-blast_named.html#BL_ORD_ID:552) 2e-04

CAQ77171 phenicol exporter (plasmid) [Pasteurella multocida] [40.0](file:///Users/JMBNew/Desktop/Articles%20en%20Cours/Article%20Diane/Bb_42-1_output-blast_named.html#BL_ORD_ID:348) 2e-04

floR_-_3362475_translation floR: florfenicol-chloramphenicol ex... [40.0](file:///Users/JMBNew/Desktop/Articles%20en%20Cours/Article%20Diane/Bb_42-1_output-blast_named.html#BL_ORD_ID:650) 2e-04

YP_232872 florfenicol-chloramphenicol exporter (plasmid) [Paste... [40.0](file:///Users/JMBNew/Desktop/Articles%20en%20Cours/Article%20Diane/Bb_42-1_output-blast_named.html#BL_ORD_ID:598) 2e-04

WP_011266118 chloramphenicol/florfenicol efflux MFS transporter... [40.0](file:///Users/JMBNew/Desktop/Articles%20en%20Cours/Article%20Diane/Bb_42-1_output-blast_named.html#BL_ORD_ID:548) 2e-04

CAI43272 florfenicol-chloramphenicol exporter (plasmid) [Pasteu... [40.0](file:///Users/JMBNew/Desktop/Articles%20en%20Cours/Article%20Diane/Bb_42-1_output-blast_named.html#BL_ORD_ID:329) 2e-04

floR_-_17434068_translation floR: Florfenicol/Chloramphenicol e... [40.0](file:///Users/JMBNew/Desktop/Articles%20en%20Cours/Article%20Diane/Bb_42-1_output-blast_named.html#BL_ORD_ID:673) 2e-04

floR_-_13917491_translation floR: FloR [40.0](file:///Users/JMBNew/Desktop/Articles%20en%20Cours/Article%20Diane/Bb_42-1_output-blast_named.html#BL_ORD_ID:669) 2e-04

floR_-_13911567_translation floR: florfenicol/ chloramphenicol ... [40.0](file:///Users/JMBNew/Desktop/Articles%20en%20Cours/Article%20Diane/Bb_42-1_output-blast_named.html#BL_ORD_ID:666) 2e-04

floR_-_13911262_translation floR: florfenicol/ chloramphenicol ... [40.0](file:///Users/JMBNew/Desktop/Articles%20en%20Cours/Article%20Diane/Bb_42-1_output-blast_named.html#BL_ORD_ID:664) 2e-04

floR_-_13905788_translation floR: florfenicol/chloramphenicol r... [40.0](file:///Users/JMBNew/Desktop/Articles%20en%20Cours/Article%20Diane/Bb_42-1_output-blast_named.html#BL_ORD_ID:663) 2e-04

floR_-_5741028_translation floR: florfenicol exporter [40.0](file:///Users/JMBNew/Desktop/Articles%20en%20Cours/Article%20Diane/Bb_42-1_output-blast_named.html#BL_ORD_ID:652) 2e-04

YP_008725150 Florfenicol/Chloramphenicol efflux protein (plasmi... [40.0](file:///Users/JMBNew/Desktop/Articles%20en%20Cours/Article%20Diane/Bb_42-1_output-blast_named.html#BL_ORD_ID:639) 2e-04

YP_008167024 FloR (plasmid) [Klebsiella pneumoniae] [40.0](file:///Users/JMBNew/Desktop/Articles%20en%20Cours/Article%20Diane/Bb_42-1_output-blast_named.html#BL_ORD_ID:635) 2e-04

YP_006960714 FloR (plasmid) [uncultured bacterium HHV216] [40.0](file:///Users/JMBNew/Desktop/Articles%20en%20Cours/Article%20Diane/Bb_42-1_output-blast_named.html#BL_ORD_ID:630) 2e-04

YP_006957001 FloR, chloramphenicol/ florfenicol resistance (pla... [40.0](file:///Users/JMBNew/Desktop/Articles%20en%20Cours/Article%20Diane/Bb_42-1_output-blast_named.html#BL_ORD_ID:628) 2e-04

YP_006956622 florfenicol/ chloramphenicol resistance protein (p... [40.0](file:///Users/JMBNew/Desktop/Articles%20en%20Cours/Article%20Diane/Bb_42-1_output-blast_named.html#BL_ORD_ID:627) 2e-04

YP_006956307 florfenicol/ chloramphenicol resistance protein (p... [40.0](file:///Users/JMBNew/Desktop/Articles%20en%20Cours/Article%20Diane/Bb_42-1_output-blast_named.html#BL_ORD_ID:626) 2e-04

YP_001552094 florfenicol exporter (plasmid) [Salmonella enteric... [40.0](file:///Users/JMBNew/Desktop/Articles%20en%20Cours/Article%20Diane/Bb_42-1_output-blast_named.html#BL_ORD_ID:606) 2e-04

SAU17560 putative chloramphenicol and florfenicol resistance pr... [40.0](file:///Users/JMBNew/Desktop/Articles%20en%20Cours/Article%20Diane/Bb_42-1_output-blast_named.html#BL_ORD_ID:520) 2e-04

CAX63193 florfenicol exporter [Acinetobacter baumannii] [40.0](file:///Users/JMBNew/Desktop/Articles%20en%20Cours/Article%20Diane/Bb_42-1_output-blast_named.html#BL_ORD_ID:349) 2e-04

BAF93177 florfenicol exporter (plasmid) [Salmonella enterica su... [40.0](file:///Users/JMBNew/Desktop/Articles%20en%20Cours/Article%20Diane/Bb_42-1_output-blast_named.html#BL_ORD_ID:315) 2e-04

ASI37824 FloR (plasmid) [Escherichia coli] [40.0](file:///Users/JMBNew/Desktop/Articles%20en%20Cours/Article%20Diane/Bb_42-1_output-blast_named.html#BL_ORD_ID:304) 2e-04

ASF80635 FloR (plasmid) [Escherichia coli] [40.0](file:///Users/JMBNew/Desktop/Articles%20en%20Cours/Article%20Diane/Bb_42-1_output-blast_named.html#BL_ORD_ID:302) 2e-04

ASF80457 FloR (plasmid) [Klebsiella pneumoniae] [40.0](file:///Users/JMBNew/Desktop/Articles%20en%20Cours/Article%20Diane/Bb_42-1_output-blast_named.html#BL_ORD_ID:301) 2e-04

ASF80271 FloR (plasmid) [Citrobacter freundii] [40.0](file:///Users/JMBNew/Desktop/Articles%20en%20Cours/Article%20Diane/Bb_42-1_output-blast_named.html#BL_ORD_ID:300) 2e-04

ASF80034 FloR (plasmid) [Citrobacter freundii] [40.0](file:///Users/JMBNew/Desktop/Articles%20en%20Cours/Article%20Diane/Bb_42-1_output-blast_named.html#BL_ORD_ID:299) 2e-04

ASF79798 FloR (plasmid) [Escherichia coli] [40.0](file:///Users/JMBNew/Desktop/Articles%20en%20Cours/Article%20Diane/Bb_42-1_output-blast_named.html#BL_ORD_ID:298) 2e-04

AMD83221 FloR (plasmid) [Klebsiella pneumoniae] [40.0](file:///Users/JMBNew/Desktop/Articles%20en%20Cours/Article%20Diane/Bb_42-1_output-blast_named.html#BL_ORD_ID:223) 2e-04

AMD83053 FloR (plasmid) [Klebsiella pneumoniae] [40.0](file:///Users/JMBNew/Desktop/Articles%20en%20Cours/Article%20Diane/Bb_42-1_output-blast_named.html#BL_ORD_ID:222) 2e-04

AKN19379 FloR (plasmid) [Salmonella enterica subsp. enterica se... [40.0](file:///Users/JMBNew/Desktop/Articles%20en%20Cours/Article%20Diane/Bb_42-1_output-blast_named.html#BL_ORD_ID:203) 2e-04

AIP92379 florfenicol exporter [Vibrio fluvialis] [40.0](file:///Users/JMBNew/Desktop/Articles%20en%20Cours/Article%20Diane/Bb_42-1_output-blast_named.html#BL_ORD_ID:174) 2e-04

AGZ05482 Florfenicol/Chloramphenicol efflux protein (plasmid) [... [40.0](file:///Users/JMBNew/Desktop/Articles%20en%20Cours/Article%20Diane/Bb_42-1_output-blast_named.html#BL_ORD_ID:144) 2e-04

AGO62602 FloR (plasmid) [Klebsiella pneumoniae] [40.0](file:///Users/JMBNew/Desktop/Articles%20en%20Cours/Article%20Diane/Bb_42-1_output-blast_named.html#BL_ORD_ID:135) 2e-04

AFG21521 florfenicol/ chloramphenicol resistance protein (plasm... [40.0](file:///Users/JMBNew/Desktop/Articles%20en%20Cours/Article%20Diane/Bb_42-1_output-blast_named.html#BL_ORD_ID:111) 2e-04

AFG21042 florfenicol/ chloramphenicol resistance protein (plasm... [40.0](file:///Users/JMBNew/Desktop/Articles%20en%20Cours/Article%20Diane/Bb_42-1_output-blast_named.html#BL_ORD_ID:110) 2e-04

AFG20876 FloR, chloramphenicol/ florfenicol resistance (plasmid... [40.0](file:///Users/JMBNew/Desktop/Articles%20en%20Cours/Article%20Diane/Bb_42-1_output-blast_named.html#BL_ORD_ID:109) 2e-04

AEX15914 florfenicol/chloramphenicol resistance protein (plasmi... [40.0](file:///Users/JMBNew/Desktop/Articles%20en%20Cours/Article%20Diane/Bb_42-1_output-blast_named.html#BL_ORD_ID:99) 2e-04

ACI02892 FloR (plasmid) [uncultured bacterium HHV216] [40.0](file:///Users/JMBNew/Desktop/Articles%20en%20Cours/Article%20Diane/Bb_42-1_output-blast_named.html#BL_ORD_ID:47) 2e-04

ABA56513 florfenicol export protein, partial [Salmonella enteri... [40.0](file:///Users/JMBNew/Desktop/Articles%20en%20Cours/Article%20Diane/Bb_42-1_output-blast_named.html#BL_ORD_ID:23) 3e-04

CEL26452 FloR (plasmid) [Escherichia coli] [40.0](file:///Users/JMBNew/Desktop/Articles%20en%20Cours/Article%20Diane/Bb_42-1_output-blast_named.html#BL_ORD_ID:399) 3e-04

CEL26214 FloR (plasmid) [Escherichia coli] [40.0](file:///Users/JMBNew/Desktop/Articles%20en%20Cours/Article%20Diane/Bb_42-1_output-blast_named.html#BL_ORD_ID:398) 3e-04

CEL26092 FloR (plasmid) [Escherichia coli] [40.0](file:///Users/JMBNew/Desktop/Articles%20en%20Cours/Article%20Diane/Bb_42-1_output-blast_named.html#BL_ORD_ID:397) 3e-04

ALS39188 Florfenicol resistance protein (plasmid) [Escherichia ... [40.0](file:///Users/JMBNew/Desktop/Articles%20en%20Cours/Article%20Diane/Bb_42-1_output-blast_named.html#BL_ORD_ID:221) 3e-04

ACK58048 florfenicol resistance protein, partial [Vibrio tasman... [39.7](file:///Users/JMBNew/Desktop/Articles%20en%20Cours/Article%20Diane/Bb_42-1_output-blast_named.html#BL_ORD_ID:51) 3e-04

CAB64207 chloramphenicol/florfenicol exporter [Salmonella enter... [40.0](file:///Users/JMBNew/Desktop/Articles%20en%20Cours/Article%20Diane/Bb_42-1_output-blast_named.html#BL_ORD_ID:322) 3e-04

ABP96837 florfenicol resistance protein, partial [Vibrio splend... [39.7](file:///Users/JMBNew/Desktop/Articles%20en%20Cours/Article%20Diane/Bb_42-1_output-blast_named.html#BL_ORD_ID:32) 3e-04

floR_-_7872488_translation floR: FloR [40.0](file:///Users/JMBNew/Desktop/Articles%20en%20Cours/Article%20Diane/Bb_42-1_output-blast_named.html#BL_ORD_ID:654) 3e-04

YP_002891083 FloR (plasmid) [Escherichia coli] [40.0](file:///Users/JMBNew/Desktop/Articles%20en%20Cours/Article%20Diane/Bb_42-1_output-blast_named.html#BL_ORD_ID:611) 3e-04

ACQ77650 FloR (plasmid) [Escherichia coli] [40.0](file:///Users/JMBNew/Desktop/Articles%20en%20Cours/Article%20Diane/Bb_42-1_output-blast_named.html#BL_ORD_ID:53) 3e-04

WP_063845119 chloramphenicol/florfenicol efflux MFS transporter... [40.0](file:///Users/JMBNew/Desktop/Articles%20en%20Cours/Article%20Diane/Bb_42-1_output-blast_named.html#BL_ORD_ID:570) 3e-04

CAL30184 Florfenicol/Chloramphenicol efflux protein [Bordetella... [40.0](file:///Users/JMBNew/Desktop/Articles%20en%20Cours/Article%20Diane/Bb_42-1_output-blast_named.html#BL_ORD_ID:341) 3e-04

AAC83804 chloramphenicol and florfenicol resistance protein, pa... [40.0](file:///Users/JMBNew/Desktop/Articles%20en%20Cours/Article%20Diane/Bb_42-1_output-blast_named.html#BL_ORD_ID:1) 3e-04

floR_-_18252523_translation floR: Florfenicol export protein [40.0](file:///Users/JMBNew/Desktop/Articles%20en%20Cours/Article%20Diane/Bb_42-1_output-blast_named.html#BL_ORD_ID:674) 3e-04

floR_-_7872612_translation floR: FloR [40.0](file:///Users/JMBNew/Desktop/Articles%20en%20Cours/Article%20Diane/Bb_42-1_output-blast_named.html#BL_ORD_ID:655) 3e-04

YP_008998279 Florfenicol export protein (plasmid) [Escherichia ... [40.0](file:///Users/JMBNew/Desktop/Articles%20en%20Cours/Article%20Diane/Bb_42-1_output-blast_named.html#BL_ORD_ID:640) 3e-04

YP_002894387 FloR (plasmid) [Escherichia coli] [40.0](file:///Users/JMBNew/Desktop/Articles%20en%20Cours/Article%20Diane/Bb_42-1_output-blast_named.html#BL_ORD_ID:612) 3e-04

SBZ20918 putative chloramphenicol and florfenicol resistance pr... [40.0](file:///Users/JMBNew/Desktop/Articles%20en%20Cours/Article%20Diane/Bb_42-1_output-blast_named.html#BL_ORD_ID:522) 3e-04

AHF23060 Florfenicol export protein (plasmid) [Escherichia coli... [40.0](file:///Users/JMBNew/Desktop/Articles%20en%20Cours/Article%20Diane/Bb_42-1_output-blast_named.html#BL_ORD_ID:150) 3e-04

ADE58508 FloR (plasmid) [Escherichia coli] [40.0](file:///Users/JMBNew/Desktop/Articles%20en%20Cours/Article%20Diane/Bb_42-1_output-blast_named.html#BL_ORD_ID:64) 3e-04

ACQ78004 FloR (plasmid) [Escherichia coli] [40.0](file:///Users/JMBNew/Desktop/Articles%20en%20Cours/Article%20Diane/Bb_42-1_output-blast_named.html#BL_ORD_ID:55) 3e-04

SBZ72551 putative chloramphenicol and florfenicol resistance pr... [40.0](file:///Users/JMBNew/Desktop/Articles%20en%20Cours/Article%20Diane/Bb_42-1_output-blast_named.html#BL_ORD_ID:524) 3e-04

AAT38901 putative efflux protein Flor, partial [Salmonella ente... [38.5](file:///Users/JMBNew/Desktop/Articles%20en%20Cours/Article%20Diane/Bb_42-1_output-blast_named.html#BL_ORD_ID:17) 3e-04

CAJ57806 florfenicol resistance protein, partial (plasmid) [Ste... [39.7](file:///Users/JMBNew/Desktop/Articles%20en%20Cours/Article%20Diane/Bb_42-1_output-blast_named.html#BL_ORD_ID:335) 3e-04

CAJ57802 florfenicol resistance protein, partial (plasmid) [Ste... [39.7](file:///Users/JMBNew/Desktop/Articles%20en%20Cours/Article%20Diane/Bb_42-1_output-blast_named.html#BL_ORD_ID:334) 3e-04

floR_-_3936963_translation floR: phenicol exporter [39.7](file:///Users/JMBNew/Desktop/Articles%20en%20Cours/Article%20Diane/Bb_42-1_output-blast_named.html#BL_ORD_ID:651) 3e-04

YP_512240 phenicol exporter (plasmid) [Bibersteinia trehalosi] [39.7](file:///Users/JMBNew/Desktop/Articles%20en%20Cours/Article%20Diane/Bb_42-1_output-blast_named.html#BL_ORD_ID:602) 3e-04

WP_011453054 MULTISPECIES: chloramphenicol/florfenicol efflux M... [39.7](file:///Users/JMBNew/Desktop/Articles%20en%20Cours/Article%20Diane/Bb_42-1_output-blast_named.html#BL_ORD_ID:549) 3e-04

CAJ65911 phenicol exporter (plasmid) [Bibersteinia trehalosi] [39.7](file:///Users/JMBNew/Desktop/Articles%20en%20Cours/Article%20Diane/Bb_42-1_output-blast_named.html#BL_ORD_ID:338) 3e-04

AMK38073 florfenicol exporter [Proteus mirabilis] [39.7](file:///Users/JMBNew/Desktop/Articles%20en%20Cours/Article%20Diane/Bb_42-1_output-blast_named.html#BL_ORD_ID:224) 3e-04

CCU69833 florfenicol-chloramphenicol exporter [Staphylococcus p... [39.7](file:///Users/JMBNew/Desktop/Articles%20en%20Cours/Article%20Diane/Bb_42-1_output-blast_named.html#BL_ORD_ID:370) 3e-04

ABO10438 florfenicol export protein [Escherichia coli] [39.7](file:///Users/JMBNew/Desktop/Articles%20en%20Cours/Article%20Diane/Bb_42-1_output-blast_named.html#BL_ORD_ID:27) 4e-04

floR_-_17035768_translation floR: florfenicol/ chloramphenicol ... [39.7](file:///Users/JMBNew/Desktop/Articles%20en%20Cours/Article%20Diane/Bb_42-1_output-blast_named.html#BL_ORD_ID:672) 4e-04

YP_008574993 florfenicol/ chloramphenicol export protein FloR (... [39.7](file:///Users/JMBNew/Desktop/Articles%20en%20Cours/Article%20Diane/Bb_42-1_output-blast_named.html#BL_ORD_ID:637) 4e-04

AGW01038 florfenicol/ chloramphenicol export protein FloR (plas... [39.7](file:///Users/JMBNew/Desktop/Articles%20en%20Cours/Article%20Diane/Bb_42-1_output-blast_named.html#BL_ORD_ID:142) 4e-04

ABO10437 florfenicol export protein [Escherichia coli] [39.7](file:///Users/JMBNew/Desktop/Articles%20en%20Cours/Article%20Diane/Bb_42-1_output-blast_named.html#BL_ORD_ID:26) 4e-04

SCA25268 putative chloramphenicol and florfenicol resistance pr... [39.7](file:///Users/JMBNew/Desktop/Articles%20en%20Cours/Article%20Diane/Bb_42-1_output-blast_named.html#BL_ORD_ID:528) 4e-04

SBZ44164 putative chloramphenicol and florfenicol resistance pr... [39.7](file:///Users/JMBNew/Desktop/Articles%20en%20Cours/Article%20Diane/Bb_42-1_output-blast_named.html#BL_ORD_ID:523) 4e-04

BAX18835 florfenicol/ chloramphenicol export protein FloR [Esch... [39.7](file:///Users/JMBNew/Desktop/Articles%20en%20Cours/Article%20Diane/Bb_42-1_output-blast_named.html#BL_ORD_ID:320) 4e-04

ASK37765 Florfenicol resistance protein (plasmid) [Escherichia ... [39.7](file:///Users/JMBNew/Desktop/Articles%20en%20Cours/Article%20Diane/Bb_42-1_output-blast_named.html#BL_ORD_ID:306) 4e-04

ASF20149 florfenicol exporter [Proteus mirabilis] [39.7](file:///Users/JMBNew/Desktop/Articles%20en%20Cours/Article%20Diane/Bb_42-1_output-blast_named.html#BL_ORD_ID:294) 4e-04

ANA09319 florfenicol export protein (plasmid) [Escherichia coli] [39.7](file:///Users/JMBNew/Desktop/Articles%20en%20Cours/Article%20Diane/Bb_42-1_output-blast_named.html#BL_ORD_ID:234) 4e-04

ALP69238 florfenicol exporter [Proteus mirabilis] [39.7](file:///Users/JMBNew/Desktop/Articles%20en%20Cours/Article%20Diane/Bb_42-1_output-blast_named.html#BL_ORD_ID:220) 4e-04

ABA64519 florfenicol export protein [Escherichia coli] [39.7](file:///Users/JMBNew/Desktop/Articles%20en%20Cours/Article%20Diane/Bb_42-1_output-blast_named.html#BL_ORD_ID:24) 4e-04

ABG36701 chloramphenicol and florfenicol resistance [Salmonella... [39.3](file:///Users/JMBNew/Desktop/Articles%20en%20Cours/Article%20Diane/Bb_42-1_output-blast_named.html#BL_ORD_ID:25) 5e-04

floR_-_11934200_translation floR: florfenicol/chloramphenicol r... [39.3](file:///Users/JMBNew/Desktop/Articles%20en%20Cours/Article%20Diane/Bb_42-1_output-blast_named.html#BL_ORD_ID:659) 5e-04

AJT60292 FloR cmlA family efflux protein (plasmid) [Escherichia... [39.3](file:///Users/JMBNew/Desktop/Articles%20en%20Cours/Article%20Diane/Bb_42-1_output-blast_named.html#BL_ORD_ID:189) 5e-04

AF332662_1 florfenicol export protein (plasmid) [Klebsiella pne... [39.3](file:///Users/JMBNew/Desktop/Articles%20en%20Cours/Article%20Diane/Bb_42-1_output-blast_named.html#BL_ORD_ID:106) 5e-04

YP_004810311 florfenicol export protein (plasmid) [Riemerella a... [38.9](file:///Users/JMBNew/Desktop/Articles%20en%20Cours/Article%20Diane/Bb_42-1_output-blast_named.html#BL_ORD_ID:620) 5e-04

WP_014053560 chloramphenicol/florfenicol efflux MFS transporter... [38.9](file:///Users/JMBNew/Desktop/Articles%20en%20Cours/Article%20Diane/Bb_42-1_output-blast_named.html#BL_ORD_ID:556) 5e-04

AEM66521 florfenicol export protein (plasmid) [Riemerella anati... [38.9](file:///Users/JMBNew/Desktop/Articles%20en%20Cours/Article%20Diane/Bb_42-1_output-blast_named.html#BL_ORD_ID:72) 5e-04

YP_001220605 florfenicol exporter (plasmid) [Aeromonas bestiarum] [38.9](file:///Users/JMBNew/Desktop/Articles%20en%20Cours/Article%20Diane/Bb_42-1_output-blast_named.html#BL_ORD_ID:603) 6e-04

DAA64637 TPA_inf: florfenicol exporter (plasmid) [Aeromonas bes... [38.9](file:///Users/JMBNew/Desktop/Articles%20en%20Cours/Article%20Diane/Bb_42-1_output-blast_named.html#BL_ORD_ID:416) 6e-04

ABQ41442 florfenicol exporter (plasmid) [Aeromonas bestiarum] [38.9](file:///Users/JMBNew/Desktop/Articles%20en%20Cours/Article%20Diane/Bb_42-1_output-blast_named.html#BL_ORD_ID:34) 6e-04

ARS43330 florfenicol-chloramphenicol exporter (plasmid) [Pasteu... [38.9](file:///Users/JMBNew/Desktop/Articles%20en%20Cours/Article%20Diane/Bb_42-1_output-blast_named.html#BL_ORD_ID:291) 6e-04

floR_-_13905643_translation floR: florfenicol/chloramphenicol r... [38.9](file:///Users/JMBNew/Desktop/Articles%20en%20Cours/Article%20Diane/Bb_42-1_output-blast_named.html#BL_ORD_ID:662) 7e-04

YP_007349558 florfenicol export (plasmid) [Klebsiella pneumoniae] [38.9](file:///Users/JMBNew/Desktop/Articles%20en%20Cours/Article%20Diane/Bb_42-1_output-blast_named.html#BL_ORD_ID:632) 7e-04

SCA37875 putative chloramphenicol and florfenicol resistance pr... [38.9](file:///Users/JMBNew/Desktop/Articles%20en%20Cours/Article%20Diane/Bb_42-1_output-blast_named.html#BL_ORD_ID:533) 7e-04

SCA36976 putative chloramphenicol and florfenicol resistance pr... [38.9](file:///Users/JMBNew/Desktop/Articles%20en%20Cours/Article%20Diane/Bb_42-1_output-blast_named.html#BL_ORD_ID:532) 7e-04

SCA29482 putative chloramphenicol and florfenicol resistance pr... [38.9](file:///Users/JMBNew/Desktop/Articles%20en%20Cours/Article%20Diane/Bb_42-1_output-blast_named.html#BL_ORD_ID:530) 7e-04

SCA27592 putative chloramphenicol and florfenicol resistance pr... [38.9](file:///Users/JMBNew/Desktop/Articles%20en%20Cours/Article%20Diane/Bb_42-1_output-blast_named.html#BL_ORD_ID:529) 7e-04

SCA25014 putative chloramphenicol and florfenicol resistance pr... [38.9](file:///Users/JMBNew/Desktop/Articles%20en%20Cours/Article%20Diane/Bb_42-1_output-blast_named.html#BL_ORD_ID:527) 7e-04

SCA21793 putative chloramphenicol and florfenicol resistance pr... [38.9](file:///Users/JMBNew/Desktop/Articles%20en%20Cours/Article%20Diane/Bb_42-1_output-blast_named.html#BL_ORD_ID:526) 7e-04

SCA10658 putative chloramphenicol and florfenicol resistance pr... [38.9](file:///Users/JMBNew/Desktop/Articles%20en%20Cours/Article%20Diane/Bb_42-1_output-blast_named.html#BL_ORD_ID:525) 7e-04

SBY74064 putative chloramphenicol and florfenicol resistance pr... [38.9](file:///Users/JMBNew/Desktop/Articles%20en%20Cours/Article%20Diane/Bb_42-1_output-blast_named.html#BL_ORD_ID:521) 7e-04

OSJ79165 florfenicol export [Salmonella enterica subsp. enteric... [38.9](file:///Users/JMBNew/Desktop/Articles%20en%20Cours/Article%20Diane/Bb_42-1_output-blast_named.html#BL_ORD_ID:519) 7e-04

OAF88709 Florfenicol export protein [Escherichia coli PCN079] [38.9](file:///Users/JMBNew/Desktop/Articles%20en%20Cours/Article%20Diane/Bb_42-1_output-blast_named.html#BL_ORD_ID:506) 7e-04

EKF76275 Florfenicol export protein [Klebsiella pneumoniae subs... [38.9](file:///Users/JMBNew/Desktop/Articles%20en%20Cours/Article%20Diane/Bb_42-1_output-blast_named.html#BL_ORD_ID:448) 7e-04

EJE85093 florfenicol exporter [Escherichia coli O111:H11 str. C... [38.9](file:///Users/JMBNew/Desktop/Articles%20en%20Cours/Article%20Diane/Bb_42-1_output-blast_named.html#BL_ORD_ID:442) 7e-04

CCN79991 florfenicol export (plasmid) [Klebsiella pneumoniae] [38.9](file:///Users/JMBNew/Desktop/Articles%20en%20Cours/Article%20Diane/Bb_42-1_output-blast_named.html#BL_ORD_ID:362) 7e-04

ASF20288 florfenicol exporter [Proteus mirabilis] [38.9](file:///Users/JMBNew/Desktop/Articles%20en%20Cours/Article%20Diane/Bb_42-1_output-blast_named.html#BL_ORD_ID:296) 7e-04

ASF20233 florfenicol exporter [Proteus mirabilis] [38.9](file:///Users/JMBNew/Desktop/Articles%20en%20Cours/Article%20Diane/Bb_42-1_output-blast_named.html#BL_ORD_ID:295) 7e-04

AQZ19169 FloR (plasmid) [[Haemophilus] parasuis] [38.9](file:///Users/JMBNew/Desktop/Articles%20en%20Cours/Article%20Diane/Bb_42-1_output-blast_named.html#BL_ORD_ID:281) 7e-04

ANA09614 florfenicol MFS transporter, FloR (plasmid) [Escherich... [38.9](file:///Users/JMBNew/Desktop/Articles%20en%20Cours/Article%20Diane/Bb_42-1_output-blast_named.html#BL_ORD_ID:235) 7e-04

AMP42434 florfenicol resistance protein [uncultured bacterium I... [38.9](file:///Users/JMBNew/Desktop/Articles%20en%20Cours/Article%20Diane/Bb_42-1_output-blast_named.html#BL_ORD_ID:231) 7e-04

AMP42401 florfenicol resistance protein [uncultured bacterium I... [38.9](file:///Users/JMBNew/Desktop/Articles%20en%20Cours/Article%20Diane/Bb_42-1_output-blast_named.html#BL_ORD_ID:230) 7e-04

ALN43627 FloR (plasmid) [Actinobacillus pleuropneumoniae] [38.9](file:///Users/JMBNew/Desktop/Articles%20en%20Cours/Article%20Diane/Bb_42-1_output-blast_named.html#BL_ORD_ID:219) 7e-04

AKM21207 FloR (plasmid) [Actinobacillus pleuropneumoniae] [38.9](file:///Users/JMBNew/Desktop/Articles%20en%20Cours/Article%20Diane/Bb_42-1_output-blast_named.html#BL_ORD_ID:202) 7e-04

AKG90161 floR (plasmid) [Salmonella enterica subsp. enterica se... [38.9](file:///Users/JMBNew/Desktop/Articles%20en%20Cours/Article%20Diane/Bb_42-1_output-blast_named.html#BL_ORD_ID:193) 7e-04

ACJ64205 florfenicol export protein (plasmid) [Edwardsiella ict... [38.9](file:///Users/JMBNew/Desktop/Articles%20en%20Cours/Article%20Diane/Bb_42-1_output-blast_named.html#BL_ORD_ID:49) 7e-04

ABO10439 florfenicol export protein [Escherichia coli] [38.9](file:///Users/JMBNew/Desktop/Articles%20en%20Cours/Article%20Diane/Bb_42-1_output-blast_named.html#BL_ORD_ID:28) 7e-04

ALJ52406 FloR (plasmid) [Escherichia coli] [38.5](file:///Users/JMBNew/Desktop/Articles%20en%20Cours/Article%20Diane/Bb_42-1_output-blast_named.html#BL_ORD_ID:218) 7e-04

CAJ30495 chloramphenicol/florfenicol exporter (plasmid) [Staphy... [38.5](file:///Users/JMBNew/Desktop/Articles%20en%20Cours/Article%20Diane/Bb_42-1_output-blast_named.html#BL_ORD_ID:332) 8e-04

CAG29651 florfenicol-chloramphenicol exporter [Staphylococcus l... [38.5](file:///Users/JMBNew/Desktop/Articles%20en%20Cours/Article%20Diane/Bb_42-1_output-blast_named.html#BL_ORD_ID:327) 8e-04

CAD70268 florfenicol-chloramphenicol exporter (plasmid) [Staphy... [38.5](file:///Users/JMBNew/Desktop/Articles%20en%20Cours/Article%20Diane/Bb_42-1_output-blast_named.html#BL_ORD_ID:325) 8e-04

AMN16506 FexA (plasmid) [Staphylococcus aureus subsp. aureus] [38.5](file:///Users/JMBNew/Desktop/Articles%20en%20Cours/Article%20Diane/Bb_42-1_output-blast_named.html#BL_ORD_ID:227) 8e-04

AGH12818 chloramphenicol/florfenicol exporter (plasmid) [Staphy... [38.5](file:///Users/JMBNew/Desktop/Articles%20en%20Cours/Article%20Diane/Bb_42-1_output-blast_named.html#BL_ORD_ID:124) 8e-04

WP_032491576 chloramphenicol/florfenicol efflux MFS transporter... [38.5](file:///Users/JMBNew/Desktop/Articles%20en%20Cours/Article%20Diane/Bb_42-1_output-blast_named.html#BL_ORD_ID:563) 8e-04

CAJ31068 chloramphenicol/florfenicol exporter (plasmid) [Staphy... [38.5](file:///Users/JMBNew/Desktop/Articles%20en%20Cours/Article%20Diane/Bb_42-1_output-blast_named.html#BL_ORD_ID:333) 8e-04

SCA35775 putative chloramphenicol and florfenicol resistance pr... [38.1](file:///Users/JMBNew/Desktop/Articles%20en%20Cours/Article%20Diane/Bb_42-1_output-blast_named.html#BL_ORD_ID:531) 0.001

BAX23716 florfenicol/ chloramphenicol export protein FloR [Esch... [38.1](file:///Users/JMBNew/Desktop/Articles%20en%20Cours/Article%20Diane/Bb_42-1_output-blast_named.html#BL_ORD_ID:321) 0.001

AIM49724 florfenicol exporter (plasmid) [Aeromonas salmonicida ... [38.1](file:///Users/JMBNew/Desktop/Articles%20en%20Cours/Article%20Diane/Bb_42-1_output-blast_named.html#BL_ORD_ID:173) 0.001

ARA90576 chloramphenicol/florfenicol exporter [Staphylococcus s... [37.7](file:///Users/JMBNew/Desktop/Articles%20en%20Cours/Article%20Diane/Bb_42-1_output-blast_named.html#BL_ORD_ID:287) 0.001

AQW34658 phenicol exporter protein [Staphylococcus sciuri] [37.7](file:///Users/JMBNew/Desktop/Articles%20en%20Cours/Article%20Diane/Bb_42-1_output-blast_named.html#BL_ORD_ID:255) 0.001

AMP42360 florfenicol efflux pump [uncultured bacterium IN-10] [37.7](file:///Users/JMBNew/Desktop/Articles%20en%20Cours/Article%20Diane/Bb_42-1_output-blast_named.html#BL_ORD_ID:229) 0.001

AIU94575 chloramphenicol and florfenicol resistance protein var... [37.7](file:///Users/JMBNew/Desktop/Articles%20en%20Cours/Article%20Diane/Bb_42-1_output-blast_named.html#BL_ORD_ID:177) 0.001

AQW34746 phenicol exporter protein [Staphylococcus sciuri] [37.4](file:///Users/JMBNew/Desktop/Articles%20en%20Cours/Article%20Diane/Bb_42-1_output-blast_named.html#BL_ORD_ID:279) 0.002

AQW34733 phenicol exporter protein [Staphylococcus sciuri] [37.4](file:///Users/JMBNew/Desktop/Articles%20en%20Cours/Article%20Diane/Bb_42-1_output-blast_named.html#BL_ORD_ID:275) 0.002

AQW34598 phenicol exporter protein [Staphylococcus sciuri] [37.4](file:///Users/JMBNew/Desktop/Articles%20en%20Cours/Article%20Diane/Bb_42-1_output-blast_named.html#BL_ORD_ID:244) 0.002

ATB51834 FloR (plasmid) [Actinobacillus indolicus] [36.2](file:///Users/JMBNew/Desktop/Articles%20en%20Cours/Article%20Diane/Bb_42-1_output-blast_named.html#BL_ORD_ID:310) 0.004

XP_002341611 florfenicol exporter, putative [Talaromyces stipit... [35.8](file:///Users/JMBNew/Desktop/Articles%20en%20Cours/Article%20Diane/Bb_42-1_output-blast_named.html#BL_ORD_ID:579) 0.006

EED24224 florfenicol exporter, putative [Talaromyces stipitatus... [35.8](file:///Users/JMBNew/Desktop/Articles%20en%20Cours/Article%20Diane/Bb_42-1_output-blast_named.html#BL_ORD_ID:425) 0.006

KTC83795 florfenicol efflux pump [Legionella brunensis] [35.0](file:///Users/JMBNew/Desktop/Articles%20en%20Cours/Article%20Diane/Bb_42-1_output-blast_named.html#BL_ORD_ID:485) 0.008

XP_018148689 florfenicol exporter [Pochonia chlamydosporia 170] [35.4](file:///Users/JMBNew/Desktop/Articles%20en%20Cours/Article%20Diane/Bb_42-1_output-blast_named.html#BL_ORD_ID:593) 0.009

OAQ72606 florfenicol exporter [Pochonia chlamydosporia 170] [35.4](file:///Users/JMBNew/Desktop/Articles%20en%20Cours/Article%20Diane/Bb_42-1_output-blast_named.html#BL_ORD_ID:507) 0.009

AQW34668 chloramphenicol/florfenicol exporter (plasmid) [Staphy... [35.0](file:///Users/JMBNew/Desktop/Articles%20en%20Cours/Article%20Diane/Bb_42-1_output-blast_named.html#BL_ORD_ID:260) 0.009

AQW34568 chloramphenicol/florfenicol exporter [Staphylococcus s... [35.0](file:///Users/JMBNew/Desktop/Articles%20en%20Cours/Article%20Diane/Bb_42-1_output-blast_named.html#BL_ORD_ID:239) 0.009

XP_002480947 florfenicol exporter, putative [Talaromyces stipit... [35.0](file:///Users/JMBNew/Desktop/Articles%20en%20Cours/Article%20Diane/Bb_42-1_output-blast_named.html#BL_ORD_ID:583) 0.010

EED20513 florfenicol exporter, putative [Talaromyces stipitatus... [35.0](file:///Users/JMBNew/Desktop/Articles%20en%20Cours/Article%20Diane/Bb_42-1_output-blast_named.html#BL_ORD_ID:424) 0.010

OCL08714 florfenicol exporter [Glonium stellatum] [35.0](file:///Users/JMBNew/Desktop/Articles%20en%20Cours/Article%20Diane/Bb_42-1_output-blast_named.html#BL_ORD_ID:510) 0.012

KTD21279 florfenicol efflux pump [Legionella londiniensis] [33.9](file:///Users/JMBNew/Desktop/Articles%20en%20Cours/Article%20Diane/Bb_42-1_output-blast_named.html#BL_ORD_ID:492) 0.023

ACV76847 major facilitator superfamily MFS_1 [Nakamurella multi... [33.9](file:///Users/JMBNew/Desktop/Articles%20en%20Cours/Article%20Diane/Bb_42-1_output-blast_named.html#BL_ORD_ID:60) 0.024

CRL95727 Bicyclomycin resistance protein (Sulfonamide resistanc... [33.5](file:///Users/JMBNew/Desktop/Articles%20en%20Cours/Article%20Diane/Bb_42-1_output-blast_named.html#BL_ORD_ID:409) 0.028

XP_002487874 florfenicol exporter, putative [Talaromyces stipit... [33.5](file:///Users/JMBNew/Desktop/Articles%20en%20Cours/Article%20Diane/Bb_42-1_output-blast_named.html#BL_ORD_ID:585) 0.038

EED12220 florfenicol exporter, putative [Talaromyces stipitatus... [33.5](file:///Users/JMBNew/Desktop/Articles%20en%20Cours/Article%20Diane/Bb_42-1_output-blast_named.html#BL_ORD_ID:422) 0.038

XP_006670772 florfenicol exporter, putative [Cordyceps militari... [33.1](file:///Users/JMBNew/Desktop/Articles%20en%20Cours/Article%20Diane/Bb_42-1_output-blast_named.html#BL_ORD_ID:588) 0.040

EGX91407 florfenicol exporter, putative [Cordyceps militaris CM01] [33.1](file:///Users/JMBNew/Desktop/Articles%20en%20Cours/Article%20Diane/Bb_42-1_output-blast_named.html#BL_ORD_ID:439) 0.040

ASF64231 Bcr/CflA family drug resistance efflux transporter [Sa... [33.1](file:///Users/JMBNew/Desktop/Articles%20en%20Cours/Article%20Diane/Bb_42-1_output-blast_named.html#BL_ORD_ID:297) 0.042

AEA08384 chloramphenicol transporter [Escherichia coli] [32.7](file:///Users/JMBNew/Desktop/Articles%20en%20Cours/Article%20Diane/Bb_42-1_output-blast_named.html#BL_ORD_ID:70) 0.045

H845_RS00265_-_25558887_translation chloramphenicol efflux pump [32.7](file:///Users/JMBNew/Desktop/Articles%20en%20Cours/Article%20Diane/Bb_42-1_output-blast_named.html#BL_ORD_ID:676) 0.047

AKU20099 chloramphenicol/florfenicol exporter [Enterococcus fae... [31.6](file:///Users/JMBNew/Desktop/Articles%20en%20Cours/Article%20Diane/Bb_42-1_output-blast_named.html#BL_ORD_ID:205) 0.12

AEV23046 florfenicol exporter protein,FexB (plasmid) [Enterococ... [31.6](file:///Users/JMBNew/Desktop/Articles%20en%20Cours/Article%20Diane/Bb_42-1_output-blast_named.html#BL_ORD_ID:91) 0.12

AEV23029 florfenicol exporter protein,FexB (plasmid) [Enterococ... [31.6](file:///Users/JMBNew/Desktop/Articles%20en%20Cours/Article%20Diane/Bb_42-1_output-blast_named.html#BL_ORD_ID:90) 0.12

ACI54526 drug resistance transporter, Bcr/CflA subfamily [Rhizo... [31.2](file:///Users/JMBNew/Desktop/Articles%20en%20Cours/Article%20Diane/Bb_42-1_output-blast_named.html#BL_ORD_ID:48) 0.16

AFH88776 florfenicol export protein, partial (plasmid) [Escheri... [30.8](file:///Users/JMBNew/Desktop/Articles%20en%20Cours/Article%20Diane/Bb_42-1_output-blast_named.html#BL_ORD_ID:113) 0.18

GAT30590 florfenicol exporter [Aspergillus luchuensis] [30.8](file:///Users/JMBNew/Desktop/Articles%20en%20Cours/Article%20Diane/Bb_42-1_output-blast_named.html#BL_ORD_ID:472) 0.19

GAA89875 florfenicol exporter [Aspergillus kawachii IFO 4308] [30.8](file:///Users/JMBNew/Desktop/Articles%20en%20Cours/Article%20Diane/Bb_42-1_output-blast_named.html#BL_ORD_ID:467) 0.19

GAQ45384 florfenicol exporter [Aspergillus niger] [30.8](file:///Users/JMBNew/Desktop/Articles%20en%20Cours/Article%20Diane/Bb_42-1_output-blast_named.html#BL_ORD_ID:471) 0.19

AAZ39943 membrane transport protein, partial [Rhizobium legumin... [30.0](file:///Users/JMBNew/Desktop/Articles%20en%20Cours/Article%20Diane/Bb_42-1_output-blast_named.html#BL_ORD_ID:22) 0.22

ORY09537 putative florfenicol exporter [Clohesyomyces aquaticus] [30.8](file:///Users/JMBNew/Desktop/Articles%20en%20Cours/Article%20Diane/Bb_42-1_output-blast_named.html#BL_ORD_ID:514) 0.23

CAL20780 putative multidrug resistance protein [Yersinia pestis... [30.0](file:///Users/JMBNew/Desktop/Articles%20en%20Cours/Article%20Diane/Bb_42-1_output-blast_named.html#BL_ORD_ID:340) 0.33

KTD48180 florfenicol efflux pump [Legionella quinlivanii] [30.0](file:///Users/JMBNew/Desktop/Articles%20en%20Cours/Article%20Diane/Bb_42-1_output-blast_named.html#BL_ORD_ID:497) 0.34

ACS55612 drug resistance transporter, Bcr/CflA subfamily [Rhizo... [30.0](file:///Users/JMBNew/Desktop/Articles%20en%20Cours/Article%20Diane/Bb_42-1_output-blast_named.html#BL_ORD_ID:56) 0.35

AFN07605 florfenicol-resistance protein, partial [Escherichia c... [28.9](file:///Users/JMBNew/Desktop/Articles%20en%20Cours/Article%20Diane/Bb_42-1_output-blast_named.html#BL_ORD_ID:116) 0.45

ACU36899 major facilitator superfamily MFS_1 [Actinosynnema mir... [29.6](file:///Users/JMBNew/Desktop/Articles%20en%20Cours/Article%20Diane/Bb_42-1_output-blast_named.html#BL_ORD_ID:57) 0.46

XP_022383730 florfenicol exporter [Aspergillus bombycis] [29.3](file:///Users/JMBNew/Desktop/Articles%20en%20Cours/Article%20Diane/Bb_42-1_output-blast_named.html#BL_ORD_ID:595) 0.64

OGM40013 florfenicol exporter [Aspergillus bombycis] [29.3](file:///Users/JMBNew/Desktop/Articles%20en%20Cours/Article%20Diane/Bb_42-1_output-blast_named.html#BL_ORD_ID:511) 0.64

EKU30462 putative chloramphenicol and florfenicol resistance pr... [29.3](file:///Users/JMBNew/Desktop/Articles%20en%20Cours/Article%20Diane/Bb_42-1_output-blast_named.html#BL_ORD_ID:449) 0.64

mdfA_-_945448_translation mdfA: multidrug efflux system protein [28.9](file:///Users/JMBNew/Desktop/Articles%20en%20Cours/Article%20Diane/Bb_42-1_output-blast_named.html#BL_ORD_ID:682) 0.80

> AKL82310 tetracycline resistance protein, partial [Escherichia

coli]

Length=167

Score = 248 bits (633), Expect = 3e-77, Method: Compositional matrix adjust.

Identities = 167/167 (100%), Positives = 167/167 (100%), Gaps = 0/167 (0%)

Frame = +1

Query 1375 LLALYALMQFACAPVLGALSDRFGRRPVLLVSLAGAAVDYAIMATAPFLWVLYigrivag 1554

LLALYALMQFACAPVLGALSDRFGRRPVLLVSLAGAAVDYAIMATAPFLWVLYIGRIVAG

Sbjct 1 LLALYALMQFACAPVLGALSDRFGRRPVLLVSLAGAAVDYAIMATAPFLWVLYIGRIVAG 60

Query 1555 itgatgavagayiaDITDGDERARHFGFMSACFGFGMVAGPVLGGLMGGFSPHapffaaa 1734

ITGATGAVAGAYIADITDGDERARHFGFMSACFGFGMVAGPVLGGLMGGFSPHAPFFAAA

Sbjct 61 ITGATGAVAGAYIADITDGDERARHFGFMSACFGFGMVAGPVLGGLMGGFSPHAPFFAAA 120

Query 1735 alnglnflTGCFLLPESHKGERRPLRREALNPLASFRWARGMTVVAA 1875

ALNGLNFLTGCFLLPESHKGERRPLRREALNPLASFRWARGMTVVAA

Sbjct 121 ALNGLNFLTGCFLLPESHKGERRPLRREALNPLASFRWARGMTVVAA 167

> AKL82311 tetracycline resistance protein, partial [Escherichia

coli]

Length=167

Score = 246 bits (629), Expect = 8e-77, Method: Compositional matrix adjust.

Identities = 166/167 (99%), Positives = 166/167 (99%), Gaps = 0/167 (0%)

Frame = +1

Query 1375 LLALYALMQFACAPVLGALSDRFGRRPVLLVSLAGAAVDYAIMATAPFLWVLYigrivag 1554

LLALYALMQFACAPVLGALSDRFGRRPVLLVSLAGAAVDYAIMAT PFLWVLYIGRIVAG

Sbjct 1 LLALYALMQFACAPVLGALSDRFGRRPVLLVSLAGAAVDYAIMATTPFLWVLYIGRIVAG 60

Query 1555 itgatgavagayiaDITDGDERARHFGFMSACFGFGMVAGPVLGGLMGGFSPHapffaaa 1734

ITGATGAVAGAYIADITDGDERARHFGFMSACFGFGMVAGPVLGGLMGGFSPHAPFFAAA

Sbjct 61 ITGATGAVAGAYIADITDGDERARHFGFMSACFGFGMVAGPVLGGLMGGFSPHAPFFAAA 120

Query 1735 alnglnflTGCFLLPESHKGERRPLRREALNPLASFRWARGMTVVAA 1875

ALNGLNFLTGCFLLPESHKGERRPLRREALNPLASFRWARGMTVVAA

Sbjct 121 ALNGLNFLTGCFLLPESHKGERRPLRREALNPLASFRWARGMTVVAA 167

> YP_004799997 florfenicol export protein (plasmid) [Riemerella

anatipestifer]

Length=407

Score = 43.9 bits (102), Expect = 2e-05, Method: Compositional matrix adjust.

Identities = 46/152 (30%), Positives = 72/152 (47%), Gaps = 14/152 (9%)

Frame = +1

Query 1261 ILSTVALDAVGIGL-IMPVLPGLLRDLVHSNDVTAHYGILLALYALMQFACAPVLGALSD 1437

IL+++A+D I L ++P +PG+L N + + L+LY +M + G LSD

Sbjct 27 ILASLAMD---IYLPVVPAMPGVL------NTTPSIIQLTLSLYMVMLGVGQVIFGPLSD 77

Query 1438 RFGRRPVLLV---SLAGAAVDYAIMATAPFLWVLYigrivagitgatgavagayiaDITD 1608

R GRRP+LLV + A++ A +TAP +V + G + A +

Sbjct 78 RVGRRPILLVGATAFVAASLGAACSSTAP-AFVAFRLLQAVGASAMMVATFATVRDVYAN 136

Query 1609 GDERARHFGFMSACFGFGMVAGPVLGGLMGGF 1704

E A +G S+ F GP+ G L+G F

Sbjct 137 RPEGAVIYGLFSSILAFVPALGPIAGALIGEF 168

> WP_014043449 chloramphenicol/florfenicol efflux MFS transporter

FloR [Riemerella anatipestifer]

Length=407

Score = 43.9 bits (102), Expect = 2e-05, Method: Compositional matrix adjust.

Identities = 46/152 (30%), Positives = 72/152 (47%), Gaps = 14/152 (9%)

Frame = +1

Query 1261 ILSTVALDAVGIGL-IMPVLPGLLRDLVHSNDVTAHYGILLALYALMQFACAPVLGALSD 1437

IL+++A+D I L ++P +PG+L N + + L+LY +M + G LSD

Sbjct 27 ILASLAMD---IYLPVVPAMPGVL------NTTPSIIQLTLSLYMVMLGVGQVIFGPLSD 77

Query 1438 RFGRRPVLLV---SLAGAAVDYAIMATAPFLWVLYigrivagitgatgavagayiaDITD 1608

R GRRP+LLV + A++ A +TAP +V + G + A +

Sbjct 78 RVGRRPILLVGATAFVAASLGAACSSTAP-AFVAFRLLQAVGASAMMVATFATVRDVYAN 136

Query 1609 GDERARHFGFMSACFGFGMVAGPVLGGLMGGF 1704

E A +G S+ F GP+ G L+G F

Sbjct 137 RPEGAVIYGLFSSILAFVPALGPIAGALIGEF 168

> AEM66531 florfenicol export protein (plasmid) [Riemerella anatipestifer]

Length=407

Score = 43.9 bits (102), Expect = 2e-05, Method: Compositional matrix adjust.

Identities = 46/152 (30%), Positives = 72/152 (47%), Gaps = 14/152 (9%)

Frame = +1

Query 1261 ILSTVALDAVGIGL-IMPVLPGLLRDLVHSNDVTAHYGILLALYALMQFACAPVLGALSD 1437

IL+++A+D I L ++P +PG+L N + + L+LY +M + G LSD

Sbjct 27 ILASLAMD---IYLPVVPAMPGVL------NTTPSIIQLTLSLYMVMLGVGQVIFGPLSD 77

Query 1438 RFGRRPVLLV---SLAGAAVDYAIMATAPFLWVLYigrivagitgatgavagayiaDITD 1608

R GRRP+LLV + A++ A +TAP +V + G + A +

Sbjct 78 RVGRRPILLVGATAFVAASLGAACSSTAP-AFVAFRLLQAVGASAMMVATFATVRDVYAN 136

Query 1609 GDERARHFGFMSACFGFGMVAGPVLGGLMGGF 1704

E A +G S+ F GP+ G L+G F

Sbjct 137 RPEGAVIYGLFSSILAFVPALGPIAGALIGEF 168

> EYH62156 putative chloramphenicol and florfenicol resistance

protein (CmlA), partial [Salmonella enterica subsp. enterica

serovar Heidelberg str. SARA 39]

Length=315

Score = 41.2 bits (95), Expect = 1e-04, Method: Compositional matrix adjust.

Identities = 45/152 (30%), Positives = 71/152 (47%), Gaps = 14/152 (9%)

Frame = +1

Query 1261 ILSTVALDAVGIGL-IMPVLPGLLRDLVHSNDVTAHYGILLALYALMQFACAPVLGALSD 1437

IL+++A+D I L ++P +PG+L N + + L+LY +M + G LSD

Sbjct 24 ILASLAMD---IYLPVVPAMPGVL------NTTPSIIQLTLSLYMVMLGVGQVIFGPLSD 74

Query 1438 RFGRRPVLLV---SLAGAAVDYAIMATAPFLWVLYigrivagitgatgavagayiaDITD 1608

R GRRP+LLV + A++ A +TA +V + G + A +

Sbjct 75 RVGRRPILLVGATAFVAASLGAACSSTA-LAFVAFRLVQAVGASAMLVATFATVRDVYAN 133

Query 1609 GDERARHFGFMSACFGFGMVAGPVLGGLMGGF 1704

E A +G S+ F GP+ G L+G F

Sbjct 134 RPEGAVIYGLFSSILAFVPALGPIAGALIGEF 165

> OAQ83863 florfenicol exporter [Purpureocillium lilacinum]

Length=539

Score = 41.6 bits (96), Expect = 1e-04, Method: Compositional matrix adjust.

Identities = 56/201 (28%), Positives = 86/201 (43%), Gaps = 20/201 (10%)

Frame = +1

Query 1144 TDKHNMSTNLSVIKNPRVQSDQRRLVRRPDVKPNRPLIVILSTVALDAVGIGLIMPVLPG 1323

+H++S N++ NP D R V RPL + S V I + L

Sbjct 26 NSQHDVS-NINRASNP--AEDDARKVETQTRDDERPLHSVFSPRIKAFVIIMTVFATLFS 82

Query 1324 LLRDLVHSNDVT----------AHYGILLALYALMQFACAPVL-GALSDRFGRRPVLLVS 1470

V+ +T + + +Y +MQ A AP+ G LSD+ GRRPV +++

Sbjct 83 PFSSFVYLPAITPIAEAYHRSLGELNLTVTVYQIMQ-AVAPLFFGDLSDQIGRRPVYMLT 141

Query 1471 LA---GAAVDYAIMATAPFLWVLYigrivagitgatgavagayiaDITDGDERARHFGFM 1641

A GA + A+ L VL + + + AT A+ A +AD+T ER + +

Sbjct 142 FAIYIGANIGLALQNNYAALMVL--RALQSTGSSATVAIGSAVVADLTTSAERGGYITAV 199

Query 1642 SACFGFGMVAGPVLGGLMGGF 1704

A F PVLGG++ F

Sbjct 200 QASVQFAPALAPVLGGILTQF 220

> AEV96403 florfenicol/chloramphenicol resistance protein, partial

[Vibrio cholerae non-O1/non-O139]

Length=209

Score = 40.0 bits (92), Expect = 1e-04, Method: Compositional matrix adjust.

Identities = 39/137 (28%), Positives = 60/137 (44%), Gaps = 10/137 (7%)

Frame = +1

Query 1303 IMPVLPGLLRDLVHSNDVTAHYGILLALYALMQFACAPVLGALSDRFGRRPVLL---VSL 1473

++P +PG+L N A + L+LY +M + G LSDR GRRP+LL +

Sbjct 4 VVPAMPGIL------NTTPAMIQLTLSLYMVMLGVGQVIFGPLSDRIGRRPILLAGATAF 57

Query 1474 AGAAVDYAIMATAPFLWVLYigrivagitgatgavagayiaDITDGDERARHFGFMSACF 1653

A++ A +TAP +V + G + A + E +G S+

Sbjct 58 VIASLGAAWSSTAP-AFVAFRLLQAVGASAMLVATFATVRDVYANRPEGVVIYGLFSSML 116

Query 1654 GFGMVAGPVLGGLMGGF 1704

F GP+ G L+G F

Sbjct 117 AFVPALGPIAGALIGEF 133

> ACJ65218 putative florfenicol exporter, partial [Vibrio cholerae]

Length=209

Score = 40.0 bits (92), Expect = 1e-04, Method: Compositional matrix adjust.

Identities = 39/137 (28%), Positives = 60/137 (44%), Gaps = 10/137 (7%)

Frame = +1

Query 1303 IMPVLPGLLRDLVHSNDVTAHYGILLALYALMQFACAPVLGALSDRFGRRPVLL---VSL 1473

++P +PG+L N A + L+LY +M + G LSDR GRRP+LL +

Sbjct 4 VVPAMPGIL------NTTPAMIQLTLSLYMVMLGVGQVIFGPLSDRIGRRPILLAGATAF 57

Query 1474 AGAAVDYAIMATAPFLWVLYigrivagitgatgavagayiaDITDGDERARHFGFMSACF 1653

A++ A +TAP +V + G + A + E +G S+

Sbjct 58 VIASLGAAWSSTAP-AFVAFRLLQAVGASAMLVATFATVRDVYANRPEGVVIYGLFSSML 116

Query 1654 GFGMVAGPVLGGLMGGF 1704

F GP+ G L+G F

Sbjct 117 AFVPALGPIAGALIGEF 133

> XP_018179360 florfenicol exporter [Purpureocillium lilacinum]

Length=564

Score = 41.2 bits (95), Expect = 1e-04, Method: Compositional matrix adjust.

Identities = 56/201 (28%), Positives = 86/201 (43%), Gaps = 20/201 (10%)

Frame = +1

Query 1144 TDKHNMSTNLSVIKNPRVQSDQRRLVRRPDVKPNRPLIVILSTVALDAVGIGLIMPVLPG 1323

+H++S N++ NP D R V RPL + S V I + L

Sbjct 26 NSQHDVS-NINRASNP--AEDDARKVETQTRDDERPLHSVFSPRIKAFVIIMTVFATLFS 82

Query 1324 LLRDLVHSNDVT----------AHYGILLALYALMQFACAPVL-GALSDRFGRRPVLLVS 1470

V+ +T + + +Y +MQ A AP+ G LSD+ GRRPV +++

Sbjct 83 PFSSFVYLPAITPIAEAYHRSLGELNLTVTVYQIMQ-AVAPLFFGDLSDQIGRRPVYMLT 141

Query 1471 LA---GAAVDYAIMATAPFLWVLYigrivagitgatgavagayiaDITDGDERARHFGFM 1641

A GA + A+ L VL + + + AT A+ A +AD+T ER + +

Sbjct 142 FAIYIGANIGLALQNNYAALMVL--RALQSTGSSATVAIGSAVVADLTTSAERGGYITAV 199

Query 1642 SACFGFGMVAGPVLGGLMGGF 1704

A F PVLGG++ F

Sbjct 200 QASVQFAPALAPVLGGILTQF 220

> OAQ90641 florfenicol exporter [Purpureocillium lilacinum]

Length=564

Score = 41.2 bits (95), Expect = 1e-04, Method: Compositional matrix adjust.

Identities = 56/201 (28%), Positives = 86/201 (43%), Gaps = 20/201 (10%)

Frame = +1

Query 1144 TDKHNMSTNLSVIKNPRVQSDQRRLVRRPDVKPNRPLIVILSTVALDAVGIGLIMPVLPG 1323

+H++S N++ NP D R V RPL + S V I + L

Sbjct 26 NSQHDVS-NINRASNP--AEDDARKVETQTRDDERPLHSVFSPRIKAFVIIMTVFATLFS 82

Query 1324 LLRDLVHSNDVT----------AHYGILLALYALMQFACAPVL-GALSDRFGRRPVLLVS 1470

V+ +T + + +Y +MQ A AP+ G LSD+ GRRPV +++

Sbjct 83 PFSSFVYLPAITPIAEAYHRSLGELNLTVTVYQIMQ-AVAPLFFGDLSDQIGRRPVYMLT 141

Query 1471 LA---GAAVDYAIMATAPFLWVLYigrivagitgatgavagayiaDITDGDERARHFGFM 1641

A GA + A+ L VL + + + AT A+ A +AD+T ER + +

Sbjct 142 FAIYIGANIGLALQNNYAALMVL--RALQSTGSSATVAIGSAVVADLTTSAERGGYITAV 199

Query 1642 SACFGFGMVAGPVLGGLMGGF 1704

A F PVLGG++ F

Sbjct 200 QASVQFAPALAPVLGGILTQF 220

> AAS16363 florfenicol export protein [Escherichia coli]

Length=404

Score = 40.8 bits (94), Expect = 2e-04, Method: Compositional matrix adjust.

Identities = 45/152 (30%), Positives = 70/152 (46%), Gaps = 14/152 (9%)

Frame = +1

Query 1261 ILSTVALDAVGIGL-IMPVLPGLLRDLVHSNDVTAHYGILLALYALMQFACAPVLGALSD 1437

IL+++A+D I L ++P +PG+L N A + L+LY +M + G LSD

Sbjct 24 ILASLAMD---IYLPVVPAMPGIL------NTTPAMIQLTLSLYVVMLGVGQVIFGPLSD 74

Query 1438 RFGRRPVLL---VSLAGAAVDYAIMATAPFLWVLYigrivagitgatgavagayiaDITD 1608

R GRRP+LL + A++ A +TAP +V + G + A +

Sbjct 75 RIGRRPILLAGATAFVIASLGAAWSSTAP-AFVAFRLLQAVGASAMLVATFATVRDVYAN 133

Query 1609 GDERARHFGFMSACFGFGMVAGPVLGGLMGGF 1704

E +G S+ F GP+ G L+G F

Sbjct 134 RPEGVVIYGLFSSILAFVPALGPIAGALIGEF 165

> YP_005221014 putative chloramphenicol and florfenicol resistance

protein (CmlA) (plasmid) [Klebsiella pneumoniae subsp. pneumoniae

HS11286]

Length=404

Score = 40.8 bits (94), Expect = 2e-04, Method: Compositional matrix adjust.

Identities = 45/152 (30%), Positives = 71/152 (47%), Gaps = 14/152 (9%)

Frame = +1

Query 1261 ILSTVALDAVGIGL-IMPVLPGLLRDLVHSNDVTAHYGILLALYALMQFACAPVLGALSD 1437

IL+++A+D I L ++P +PG+L N + + L+LY +M + G LSD

Sbjct 24 ILASLAMD---IYLPVVPAMPGVL------NTTPSIIQLTLSLYMVMLGVGQVIFGPLSD 74

Query 1438 RFGRRPVLLV---SLAGAAVDYAIMATAPFLWVLYigrivagitgatgavagayiaDITD 1608

R GRRP+LLV + A++ A +TA +V + G + A +

Sbjct 75 RVGRRPILLVGATAFVAASLGAACSSTA-LAFVAFRLVQAVGASAMLVATFATVRDVYAN 133

Query 1609 GDERARHFGFMSACFGFGMVAGPVLGGLMGGF 1704

E A +G S+ F GP+ G L+G F

Sbjct 134 RPEGAVIYGLFSSMLAFVPALGPIAGALIGEF 165

> CAM88409 putative Chloramphenicol and florfenicol resistance

protein (CmlA) [Acinetobacter baumannii AYE]

Length=404

Score = 40.8 bits (94), Expect = 2e-04, Method: Compositional matrix adjust.

Identities = 45/152 (30%), Positives = 71/152 (47%), Gaps = 14/152 (9%)

Frame = +1

Query 1261 ILSTVALDAVGIGL-IMPVLPGLLRDLVHSNDVTAHYGILLALYALMQFACAPVLGALSD 1437

IL+++A+D I L ++P +PG+L N + + L+LY +M + G LSD

Sbjct 24 ILASLAMD---IYLPVVPAMPGVL------NTTPSIIQLTLSLYMVMLGVGQVIFGPLSD 74

Query 1438 RFGRRPVLLV---SLAGAAVDYAIMATAPFLWVLYigrivagitgatgavagayiaDITD 1608

R GRRP+LLV + A++ A +TA +V + G + A +

Sbjct 75 RVGRRPILLVGATAFVAASLGAACSSTA-LAFVAFRLVQAVGASAMLVATFATVRDVYAN 133

Query 1609 GDERARHFGFMSACFGFGMVAGPVLGGLMGGF 1704

E A +G S+ F GP+ G L+G F

Sbjct 134 RPEGAVIYGLFSSMLAFVPALGPIAGALIGEF 165

> CAJ77032 chloramphenicol and florfenicol resistance protein [Acinetobacter

baumannii]

Length=404

Score = 40.8 bits (94), Expect = 2e-04, Method: Compositional matrix adjust.

Identities = 45/152 (30%), Positives = 71/152 (47%), Gaps = 14/152 (9%)

Frame = +1

Query 1261 ILSTVALDAVGIGL-IMPVLPGLLRDLVHSNDVTAHYGILLALYALMQFACAPVLGALSD 1437

IL+++A+D I L ++P +PG+L N + + L+LY +M + G LSD

Sbjct 24 ILASLAMD---IYLPVVPAMPGVL------NTTPSIIQLTLSLYMVMLGVGQVIFGPLSD 74

Query 1438 RFGRRPVLLV---SLAGAAVDYAIMATAPFLWVLYigrivagitgatgavagayiaDITD 1608

R GRRP+LLV + A++ A +TA +V + G + A +

Sbjct 75 RVGRRPILLVGATAFVAASLGAACSSTA-LAFVAFRLVQAVGASAMLVATFATVRDVYAN 133

Query 1609 GDERARHFGFMSACFGFGMVAGPVLGGLMGGF 1704

E A +G S+ F GP+ G L+G F

Sbjct 134 RPEGAVIYGLFSSMLAFVPALGPIAGALIGEF 165

> ASR82671 chloramphenicol and florfenicol resistance protein (plasmid)

[Klebsiella pneumoniae]

Length=404

Score = 40.8 bits (94), Expect = 2e-04, Method: Compositional matrix adjust.

Identities = 45/152 (30%), Positives = 71/152 (47%), Gaps = 14/152 (9%)

Frame = +1

Query 1261 ILSTVALDAVGIGL-IMPVLPGLLRDLVHSNDVTAHYGILLALYALMQFACAPVLGALSD 1437

IL+++A+D I L ++P +PG+L N + + L+LY +M + G LSD

Sbjct 24 ILASLAMD---IYLPVVPAMPGVL------NTTPSIIQLTLSLYMVMLGVGQVIFGPLSD 74

Query 1438 RFGRRPVLLV---SLAGAAVDYAIMATAPFLWVLYigrivagitgatgavagayiaDITD 1608

R GRRP+LLV + A++ A +TA +V + G + A +

Sbjct 75 RVGRRPILLVGATAFVAASLGAACSSTA-LAFVAFRLVQAVGASAMLVATFATVRDVYAN 133

Query 1609 GDERARHFGFMSACFGFGMVAGPVLGGLMGGF 1704

E A +G S+ F GP+ G L+G F

Sbjct 134 RPEGAVIYGLFSSMLAFVPALGPIAGALIGEF 165

> ASK37999 chloramphenicol and florfenicol resistance protein (plasmid)

[Klebsiella pneumoniae]

Length=404

Score = 40.8 bits (94), Expect = 2e-04, Method: Compositional matrix adjust.

Identities = 45/152 (30%), Positives = 71/152 (47%), Gaps = 14/152 (9%)

Frame = +1

Query 1261 ILSTVALDAVGIGL-IMPVLPGLLRDLVHSNDVTAHYGILLALYALMQFACAPVLGALSD 1437

IL+++A+D I L ++P +PG+L N + + L+LY +M + G LSD

Sbjct 24 ILASLAMD---IYLPVVPAMPGVL------NTTPSIIQLTLSLYMVMLGVGQVIFGPLSD 74

Query 1438 RFGRRPVLLV---SLAGAAVDYAIMATAPFLWVLYigrivagitgatgavagayiaDITD 1608

R GRRP+LLV + A++ A +TA +V + G + A +

Sbjct 75 RVGRRPILLVGATAFVAASLGAACSSTA-LAFVAFRLVQAVGASAMLVATFATVRDVYAN 133

Query 1609 GDERARHFGFMSACFGFGMVAGPVLGGLMGGF 1704

E A +G S+ F GP+ G L+G F

Sbjct 134 RPEGAVIYGLFSSMLAFVPALGPIAGALIGEF 165

> ASJ23722 chloramphenicol and florfenicol resistance protein [Laribacter

hongkongensis]

Length=404

Score = 40.8 bits (94), Expect = 2e-04, Method: Compositional matrix adjust.

Identities = 45/152 (30%), Positives = 71/152 (47%), Gaps = 14/152 (9%)

Frame = +1

Query 1261 ILSTVALDAVGIGL-IMPVLPGLLRDLVHSNDVTAHYGILLALYALMQFACAPVLGALSD 1437

IL+++A+D I L ++P +PG+L N + + L+LY +M + G LSD

Sbjct 24 ILASLAMD---IYLPVVPAMPGVL------NTTPSIIQLTLSLYMVMLGVGQVIFGPLSD 74

Query 1438 RFGRRPVLLV---SLAGAAVDYAIMATAPFLWVLYigrivagitgatgavagayiaDITD 1608

R GRRP+LLV + A++ A +TA +V + G + A +

Sbjct 75 RVGRRPILLVGATAFVAASLGAACSSTA-LAFVAFRLVQAVGASAMLVATFATVRDVYAN 133

Query 1609 GDERARHFGFMSACFGFGMVAGPVLGGLMGGF 1704

E A +G S+ F GP+ G L+G F

Sbjct 134 RPEGAVIYGLFSSMLAFVPALGPIAGALIGEF 165

> AGT26821 chloramphenicol and florfenicol resistance protein (CmlA)

(plasmid) [Klebsiella pneumoniae JM45]

Length=404

Score = 40.8 bits (94), Expect = 2e-04, Method: Compositional matrix adjust.

Identities = 45/152 (30%), Positives = 71/152 (47%), Gaps = 14/152 (9%)

Frame = +1

Query 1261 ILSTVALDAVGIGL-IMPVLPGLLRDLVHSNDVTAHYGILLALYALMQFACAPVLGALSD 1437

IL+++A+D I L ++P +PG+L N + + L+LY +M + G LSD

Sbjct 24 ILASLAMD---IYLPVVPAMPGVL------NTTPSIIQLTLSLYMVMLGVGQVIFGPLSD 74

Query 1438 RFGRRPVLLV---SLAGAAVDYAIMATAPFLWVLYigrivagitgatgavagayiaDITD 1608

R GRRP+LLV + A++ A +TA +V + G + A +

Sbjct 75 RVGRRPILLVGATAFVAASLGAACSSTA-LAFVAFRLVQAVGASAMLVATFATVRDVYAN 133

Query 1609 GDERARHFGFMSACFGFGMVAGPVLGGLMGGF 1704

E A +G S+ F GP+ G L+G F

Sbjct 134 RPEGAVIYGLFSSMLAFVPALGPIAGALIGEF 165

> AEW92275 putative chloramphenicol and florfenicol resistance

protein (CmlA) (plasmid) [Klebsiella pneumoniae subsp. pneumoniae

HS11286]

Length=404

Score = 40.8 bits (94), Expect = 2e-04, Method: Compositional matrix adjust.

Identities = 45/152 (30%), Positives = 71/152 (47%), Gaps = 14/152 (9%)

Frame = +1

Query 1261 ILSTVALDAVGIGL-IMPVLPGLLRDLVHSNDVTAHYGILLALYALMQFACAPVLGALSD 1437

IL+++A+D I L ++P +PG+L N + + L+LY +M + G LSD

Sbjct 24 ILASLAMD---IYLPVVPAMPGVL------NTTPSIIQLTLSLYMVMLGVGQVIFGPLSD 74

Query 1438 RFGRRPVLLV---SLAGAAVDYAIMATAPFLWVLYigrivagitgatgavagayiaDITD 1608

R GRRP+LLV + A++ A +TA +V + G + A +

Sbjct 75 RVGRRPILLVGATAFVAASLGAACSSTA-LAFVAFRLVQAVGASAMLVATFATVRDVYAN 133

Query 1609 GDERARHFGFMSACFGFGMVAGPVLGGLMGGF 1704

E A +G S+ F GP+ G L+G F

Sbjct 134 RPEGAVIYGLFSSMLAFVPALGPIAGALIGEF 165

> ABZ01840 CmlA9 chloramphenicol efflux protein [Salmonella enterica

subsp. enterica]

Length=404

Score = 40.8 bits (94), Expect = 2e-04, Method: Compositional matrix adjust.

Identities = 45/152 (30%), Positives = 71/152 (47%), Gaps = 14/152 (9%)

Frame = +1

Query 1261 ILSTVALDAVGIGL-IMPVLPGLLRDLVHSNDVTAHYGILLALYALMQFACAPVLGALSD 1437

IL+++A+D I L ++P +PG+L N + + L+LY +M + G LSD

Sbjct 24 ILASLAMD---IYLPVVPAMPGVL------NTTPSIIQLTLSLYMVMLGVGQVIFGPLSD 74

Query 1438 RFGRRPVLLV---SLAGAAVDYAIMATAPFLWVLYigrivagitgatgavagayiaDITD 1608

R GRRP+LLV + A++ A +TA +V + G + A +

Sbjct 75 RVGRRPILLVGATAFVAASLGAACSSTA-LAFVAFRLVQAVGASAMLVATFATVRDVYAN 133

Query 1609 GDERARHFGFMSACFGFGMVAGPVLGGLMGGF 1704

E A +G S+ F GP+ G L+G F

Sbjct 134 RPEGAVIYGLFSSMLAFVPALGPIAGALIGEF 165

> floR_-_13918086_translation floR: florfenicol-chloramphenicol

exporter

Length=404

Score = 40.4 bits (93), Expect = 2e-04, Method: Compositional matrix adjust.

Identities = 45/152 (30%), Positives = 70/152 (46%), Gaps = 14/152 (9%)

Frame = +1

Query 1261 ILSTVALDAVGIGL-IMPVLPGLLRDLVHSNDVTAHYGILLALYALMQFACAPVLGALSD 1437

IL+++A+D I L ++P +PG+L N A + L+LY +M + G LSD

Sbjct 24 ILASLAMD---IYLPVVPAMPGIL------NTTPAMIQLTLSLYMVMLGVGQVIFGPLSD 74

Query 1438 RFGRRPVLL---VSLAGAAVDYAIMATAPFLWVLYigrivagitgatgavagayiaDITD 1608

R GRRP+LL + A++ A +TAP +V + G + A +

Sbjct 75 RIGRRPILLAGATAFVIASLGAAWSSTAP-AFVAFRLLQAVGASAMLVATFATVRDVYAN 133

Query 1609 GDERARHFGFMSACFGFGMVAGPVLGGLMGGF 1704

E +G S+ F GP+ G L+G F

Sbjct 134 RPEGVVIYGLFSSVLAFVPALGPIAGALIGEF 165

> YP_006961358 florfenicol-chloramphenicol exporter (plasmid) [Mannheimia

haemolytica]

Length=404

Score = 40.4 bits (93), Expect = 2e-04, Method: Compositional matrix adjust.

Identities = 45/152 (30%), Positives = 70/152 (46%), Gaps = 14/152 (9%)

Frame = +1

Query 1261 ILSTVALDAVGIGL-IMPVLPGLLRDLVHSNDVTAHYGILLALYALMQFACAPVLGALSD 1437

IL+++A+D I L ++P +PG+L N A + L+LY +M + G LSD

Sbjct 24 ILASLAMD---IYLPVVPAMPGIL------NTTPAMIQLTLSLYMVMLGVGQVIFGPLSD 74

Query 1438 RFGRRPVLL---VSLAGAAVDYAIMATAPFLWVLYigrivagitgatgavagayiaDITD 1608

R GRRP+LL + A++ A +TAP +V + G + A +

Sbjct 75 RIGRRPILLAGATAFVIASLGAAWSSTAP-AFVAFRLLQAVGASAMLVATFATVRDVYAN 133

Query 1609 GDERARHFGFMSACFGFGMVAGPVLGGLMGGF 1704

E +G S+ F GP+ G L+G F

Sbjct 134 RPEGVVIYGLFSSVLAFVPALGPIAGALIGEF 165

> BAL04183 florfenicol-chloramphenicol exporter (plasmid) [Mannheimia

haemolytica]

Length=404

Score = 40.4 bits (93), Expect = 2e-04, Method: Compositional matrix adjust.

Identities = 45/152 (30%), Positives = 70/152 (46%), Gaps = 14/152 (9%)

Frame = +1

Query 1261 ILSTVALDAVGIGL-IMPVLPGLLRDLVHSNDVTAHYGILLALYALMQFACAPVLGALSD 1437

IL+++A+D I L ++P +PG+L N A + L+LY +M + G LSD

Sbjct 24 ILASLAMD---IYLPVVPAMPGIL------NTTPAMIQLTLSLYMVMLGVGQVIFGPLSD 74

Query 1438 RFGRRPVLL---VSLAGAAVDYAIMATAPFLWVLYigrivagitgatgavagayiaDITD 1608

R GRRP+LL + A++ A +TAP +V + G + A +

Sbjct 75 RIGRRPILLAGATAFVIASLGAAWSSTAP-AFVAFRLLQAVGASAMLVATFATVRDVYAN 133

Query 1609 GDERARHFGFMSACFGFGMVAGPVLGGLMGGF 1704

E +G S+ F GP+ G L+G F

Sbjct 134 RPEGVVIYGLFSSVLAFVPALGPIAGALIGEF 165

> BAC79058 florfenicol exporter [Vibrio cholerae]

Length=404

Score = 40.4 bits (93), Expect = 2e-04, Method: Compositional matrix adjust.

Identities = 45/152 (30%), Positives = 70/152 (46%), Gaps = 14/152 (9%)

Frame = +1

Query 1261 ILSTVALDAVGIGL-IMPVLPGLLRDLVHSNDVTAHYGILLALYALMQFACAPVLGALSD 1437

IL+++A+D I L ++P +PG+L N A + L+LY +M + G LSD

Sbjct 24 ILASLAMD---IYLPVVPAMPGIL------NTTPAMIQLTLSLYMVMLGVGQVIFGPLSD 74

Query 1438 RFGRRPVLL---VSLAGAAVDYAIMATAPFLWVLYigrivagitgatgavagayiaDITD 1608

R GRRP+LL + A++ A +TAP +V + G + A +

Sbjct 75 RIGRRPILLAGATAFVIASLGAAWSSTAP-AFVAFRLLQAVGASAMLVATFATVRDAYAN 133

Query 1609 GDERARHFGFMSACFGFGMVAGPVLGGLMGGF 1704

E +G S+ F GP+ G L+G F

Sbjct 134 RPEGVVIYGLFSSMLAFVPALGPIAGALIGEF 165

> AAL59749 florfenicol exporter [Vibrio cholerae]

Length=404

Score = 40.4 bits (93), Expect = 2e-04, Method: Compositional matrix adjust.

Identities = 45/152 (30%), Positives = 70/152 (46%), Gaps = 14/152 (9%)

Frame = +1

Query 1261 ILSTVALDAVGIGL-IMPVLPGLLRDLVHSNDVTAHYGILLALYALMQFACAPVLGALSD 1437

IL+++A+D I L ++P +PG+L N A + L+LY +M + G LSD

Sbjct 24 ILASLAMD---IYLPVVPAMPGIL------NTTPAMIQLTLSLYMVMLGVGQVIFGPLSD 74

Query 1438 RFGRRPVLL---VSLAGAAVDYAIMATAPFLWVLYigrivagitgatgavagayiaDITD 1608

R GRRP+LL + A++ A +TAP +V + G + A +

Sbjct 75 RIGRRPILLAGATAFVIASLGAAWSSTAP-AFVAFRLLQAVGASAMLVATFATVRDAYAN 133

Query 1609 GDERARHFGFMSACFGFGMVAGPVLGGLMGGF 1704

E +G S+ F GP+ G L+G F

Sbjct 134 RPEGVVIYGLFSSMLAFVPALGPIAGALIGEF 165

> AAK64587 florfenicol exporter [Vibrio cholerae MO10]

Length=404

Score = 40.4 bits (93), Expect = 2e-04, Method: Compositional matrix adjust.

Identities = 45/152 (30%), Positives = 70/152 (46%), Gaps = 14/152 (9%)

Frame = +1

Query 1261 ILSTVALDAVGIGL-IMPVLPGLLRDLVHSNDVTAHYGILLALYALMQFACAPVLGALSD 1437

IL+++A+D I L ++P +PG+L N A + L+LY +M + G LSD

Sbjct 24 ILASLAMD---IYLPVVPAMPGIL------NTTPAMIQLTLSLYMVMLGVGQVIFGPLSD 74

Query 1438 RFGRRPVLL---VSLAGAAVDYAIMATAPFLWVLYigrivagitgatgavagayiaDITD 1608

R GRRP+LL + A++ A +TAP +V + G + A +

Sbjct 75 RIGRRPILLAGATAFVIASLGAAWSSTAP-AFVAFRLLQAVGASAMLVATFATVRDAYAN 133

Query 1609 GDERARHFGFMSACFGFGMVAGPVLGGLMGGF 1704

E +G S+ F GP+ G L+G F

Sbjct 134 RPEGVVIYGLFSSMLAFVPALGPIAGALIGEF 165

> AHG85140 Florfenicol export protein [Bibersteinia trehalosi USDA-ARS-USMARC-189]

Length=404

Score = 40.4 bits (93), Expect = 2e-04, Method: Compositional matrix adjust.

Identities = 45/152 (30%), Positives = 70/152 (46%), Gaps = 14/152 (9%)

Frame = +1

Query 1261 ILSTVALDAVGIGL-IMPVLPGLLRDLVHSNDVTAHYGILLALYALMQFACAPVLGALSD 1437

IL+++A+D I L ++P +PG+L N A + L+LY +M + G LSD

Sbjct 24 ILASLAMD---IYLPVVPAMPGIL------NTTPAMIQLTLSLYMVMLGVGQVIFGPLSD 74

Query 1438 RFGRRPVLL---VSLAGAAVDYAIMATAPFLWVLYigrivagitgatgavagayiaDITD 1608

R GRRP+LL + A++ A +TAP +V + G + A +

Sbjct 75 RIGRRPILLAGATAFVIASLGAAWSSTAP-AFVAFRLLQAVGASAMLVATFATVRDVYAN 133

Query 1609 GDERARHFGFMSACFGFGMVAGPVLGGLMGGF 1704

E +G S+ F GP+ G L+G F

Sbjct 134 RPEGVVIYGLFSSVLAFVPALGPIAGALIGEF 165

> AGH37387 Florfenicol export protein [Bibersteinia trehalosi USDA-ARS-USMARC-192]

Length=404

Score = 40.4 bits (93), Expect = 2e-04, Method: Compositional matrix adjust.

Identities = 45/152 (30%), Positives = 70/152 (46%), Gaps = 14/152 (9%)

Frame = +1

Query 1261 ILSTVALDAVGIGL-IMPVLPGLLRDLVHSNDVTAHYGILLALYALMQFACAPVLGALSD 1437

IL+++A+D I L ++P +PG+L N A + L+LY +M + G LSD

Sbjct 24 ILASLAMD---IYLPVVPAMPGIL------NTTPAMIQLTLSLYMVMLGVGQVIFGPLSD 74

Query 1438 RFGRRPVLL---VSLAGAAVDYAIMATAPFLWVLYigrivagitgatgavagayiaDITD 1608

R GRRP+LL + A++ A +TAP +V + G + A +

Sbjct 75 RIGRRPILLAGATAFVIASLGAAWSSTAP-AFVAFRLLQAVGASAMLVATFATVRDVYAN 133

Query 1609 GDERARHFGFMSACFGFGMVAGPVLGGLMGGF 1704

E +G S+ F GP+ G L+G F

Sbjct 134 RPEGVVIYGLFSSVLAFVPALGPIAGALIGEF 165

> AHG85541 Florfenicol export protein [Bibersteinia trehalosi USDA-ARS-USMARC-190]

Length=404

Score = 40.4 bits (93), Expect = 2e-04, Method: Compositional matrix adjust.

Identities = 45/152 (30%), Positives = 70/152 (46%), Gaps = 14/152 (9%)

Frame = +1

Query 1261 ILSTVALDAVGIGL-IMPVLPGLLRDLVHSNDVTAHYGILLALYALMQFACAPVLGALSD 1437

IL+++A+D I L ++P +PG+L N A + L+LY +M + G LSD

Sbjct 24 ILASLAMD---IYLPVVPAMPGIL------NTTPAMIQLTLSLYMVMLGVGQVIFGPLSD 74

Query 1438 RFGRRPVLL---VSLAGAAVDYAIMATAPFLWVLYigrivagitgatgavagayiaDITD 1608

R GRRP+LL + A++ A +TAP +V + G + A +

Sbjct 75 RIGRRPILLAGATAFVIASLGAAWSSTAP-AFVAFRLLQAVGASAMLVATFATVRDVYAN 133

Query 1609 GDERARHFGFMSACFGFGMVAGPVLGGLMGGF 1704

E +G S+ F GP+ G L+G F

Sbjct 134 RPEGVVIYGLFSSVLAFVPALGPIAGALIGEF 165

> AET15214 florfenicol / chloramphenicol efflux protein [Pasteurella

multocida 36950]

Length=404

Score = 40.4 bits (93), Expect = 2e-04, Method: Compositional matrix adjust.

Identities = 45/152 (30%), Positives = 70/152 (46%), Gaps = 14/152 (9%)

Frame = +1

Query 1261 ILSTVALDAVGIGL-IMPVLPGLLRDLVHSNDVTAHYGILLALYALMQFACAPVLGALSD 1437

IL+++A+D I L ++P +PG+L N A + L+LY +M + G LSD

Sbjct 24 ILASLAMD---IYLPVVPAMPGIL------NTTPAMIQLTLSLYMVMLGVGQVIFGPLSD 74

Query 1438 RFGRRPVLL---VSLAGAAVDYAIMATAPFLWVLYigrivagitgatgavagayiaDITD 1608

R GRRP+LL + A++ A +TAP +V + G + A +

Sbjct 75 RIGRRPILLAGATAFVIASLGAAWSSTAP-AFVAFRLLQAVGASAMLVATFATVRDVYAN 133

Query 1609 GDERARHFGFMSACFGFGMVAGPVLGGLMGGF 1704

E +G S+ F GP+ G L+G F

Sbjct 134 RPEGVVIYGLFSSVLAFVPALGPIAGALIGEF 165

> AKJ66278 FloR (plasmid) [[Haemophilus] parasuis]

Length=404

Score = 40.4 bits (93), Expect = 2e-04, Method: Compositional matrix adjust.

Identities = 45/152 (30%), Positives = 71/152 (47%), Gaps = 14/152 (9%)

Frame = +1

Query 1261 ILSTVALDAVGIGL-IMPVLPGLLRDLVHSNDVTAHYGILLALYALMQFACAPVLGALSD 1437

IL+++A+D I L ++P +PG+L N + + L+LY +M + G LSD

Sbjct 24 ILASLAMD---IYLPVVPAMPGVL------NTTPSIIQLTLSLYMVMLGVGQVIFGPLSD 74

Query 1438 RFGRRPVLLV---SLAGAAVDYAIMATAPFLWVLYigrivagitgatgavagayiaDITD 1608

R GRRP+LLV + A++ A +TA +V + G + A +

Sbjct 75 RVGRRPILLVGATAFVAASLGAACSSTA-LAFVAFRLLQAVGASAMMVATFATVRDVYAN 133

Query 1609 GDERARHFGFMSACFGFGMVAGPVLGGLMGGF 1704

E A +G S+ F GP+ G L+G F

Sbjct 134 RPEGAVIYGLFSSMLAFVPALGPIAGALIGEF 165

> floR_-_7886606_translation floR: FloR

Length=404

Score = 40.4 bits (93), Expect = 2e-04, Method: Compositional matrix adjust.

Identities = 45/152 (30%), Positives = 70/152 (46%), Gaps = 14/152 (9%)

Frame = +1

Query 1261 ILSTVALDAVGIGL-IMPVLPGLLRDLVHSNDVTAHYGILLALYALMQFACAPVLGALSD 1437

IL+++A+D I L ++P +PG+L N A + L+LY +M + G LSD

Sbjct 24 ILASLAMD---IYLPVVPAMPGIL------NTTPAMIQLTLSLYMVMLGVGQVIFGPLSD 74

Query 1438 RFGRRPVLL---VSLAGAAVDYAIMATAPFLWVLYigrivagitgatgavagayiaDITD 1608

R GRRP+LL + A++ A +TAP +V + G + A +

Sbjct 75 RIGRRPILLAGATAFVIASLGAAWSSTAP-AFVAFRLLQAVGASAMLVATFATVRDVYAN 133

Query 1609 GDERARHFGFMSACFGFGMVAGPVLGGLMGGF 1704

E +G S+ F GP+ G L+G F

Sbjct 134 RPEGVVIYGLFSSVLAFVPALGPIAGALIGEF 165

> YP_002894576 FloR (plasmid) [Salmonella enterica]

Length=404

Score = 40.4 bits (93), Expect = 2e-04, Method: Compositional matrix adjust.

Identities = 45/152 (30%), Positives = 70/152 (46%), Gaps = 14/152 (9%)

Frame = +1

Query 1261 ILSTVALDAVGIGL-IMPVLPGLLRDLVHSNDVTAHYGILLALYALMQFACAPVLGALSD 1437

IL+++A+D I L ++P +PG+L N A + L+LY +M + G LSD

Sbjct 24 ILASLAMD---IYLPVVPAMPGIL------NTTPAMIQLTLSLYMVMLGVGQVIFGPLSD 74

Query 1438 RFGRRPVLL---VSLAGAAVDYAIMATAPFLWVLYigrivagitgatgavagayiaDITD 1608

R GRRP+LL + A++ A +TAP +V + G + A +

Sbjct 75 RIGRRPILLAGATAFVIASLGAAWSSTAP-AFVAFRLLQAVGASAMLVATFATVRDVYAN 133

Query 1609 GDERARHFGFMSACFGFGMVAGPVLGGLMGGF 1704

E +G S+ F GP+ G L+G F

Sbjct 134 RPEGVVIYGLFSSVLAFVPALGPIAGALIGEF 165

> AF231986_1 florfenicol exporter (plasmid) [Escherichia coli]

Length=404

Score = 40.4 bits (93), Expect = 2e-04, Method: Compositional matrix adjust.

Identities = 45/152 (30%), Positives = 70/152 (46%), Gaps = 14/152 (9%)

Frame = +1

Query 1261 ILSTVALDAVGIGL-IMPVLPGLLRDLVHSNDVTAHYGILLALYALMQFACAPVLGALSD 1437

IL+++A+D I L ++P +PG+L N A + L+LY +M + G LSD

Sbjct 24 ILASLAMD---IYLPVVPAMPGIL------NTTPAMIQLTLSLYMVMLGVGQVIFGPLSD 74

Query 1438 RFGRRPVLL---VSLAGAAVDYAIMATAPFLWVLYigrivagitgatgavagayiaDITD 1608

R GRRP+LL + A++ A +TAP +V + G + A +

Sbjct 75 RIGRRPILLAGATAFVIASLGAAWSSTAP-AFVAFRLLQAVGASAMLVATFATVRDVYAN 133

Query 1609 GDERARHFGFMSACFGFGMVAGPVLGGLMGGF 1704

E +G S+ F GP+ G L+G F

Sbjct 134 RPEGVVIYGLFSSVLAFVPALGPIAGALIGEF 165

> ACQ77823 FloR (plasmid) [Salmonella enterica]

Length=404

Score = 40.4 bits (93), Expect = 2e-04, Method: Compositional matrix adjust.

Identities = 45/152 (30%), Positives = 70/152 (46%), Gaps = 14/152 (9%)

Frame = +1

Query 1261 ILSTVALDAVGIGL-IMPVLPGLLRDLVHSNDVTAHYGILLALYALMQFACAPVLGALSD 1437

IL+++A+D I L ++P +PG+L N A + L+LY +M + G LSD

Sbjct 24 ILASLAMD---IYLPVVPAMPGIL------NTTPAMIQLTLSLYMVMLGVGQVIFGPLSD 74

Query 1438 RFGRRPVLL---VSLAGAAVDYAIMATAPFLWVLYigrivagitgatgavagayiaDITD 1608

R GRRP+LL + A++ A +TAP +V + G + A +

Sbjct 75 RIGRRPILLAGATAFVIASLGAAWSSTAP-AFVAFRLLQAVGASAMLVATFATVRDVYAN 133

Query 1609 GDERARHFGFMSACFGFGMVAGPVLGGLMGGF 1704

E +G S+ F GP+ G L+G F

Sbjct 134 RPEGVVIYGLFSSVLAFVPALGPIAGALIGEF 165

> AAS16362 florfenicol export protein [Escherichia coli]

Length=404

Score = 40.4 bits (93), Expect = 2e-04, Method: Compositional matrix adjust.

Identities = 45/152 (30%), Positives = 70/152 (46%), Gaps = 14/152 (9%)

Frame = +1

Query 1261 ILSTVALDAVGIGL-IMPVLPGLLRDLVHSNDVTAHYGILLALYALMQFACAPVLGALSD 1437

IL+++A+D I L ++P +PG+L N A + L+LY +M + G LSD

Sbjct 24 ILASLAMD---IYLPVVPAMPGIL------NTTPAMIQLTLSLYMVMLGVGQVIFGPLSD 74

Query 1438 RFGRRPVLL---VSLAGAAVDYAIMATAPFLWVLYigrivagitgatgavagayiaDITD 1608

R GRRP+LL + A++ A +TAP +V + G + A +

Sbjct 75 RIGRRPILLAGATAFVIASLGAAWSSTAP-AFVAFRLLQAVGASAMLVATFATVRDVYAN 133

Query 1609 GDERARHFGFMSACFGFGMVAGPVLGGLMGGF 1704

E +G S+ F GP+ G L+G F

Sbjct 134 RPEGVVIYGLFSSILAFVPALGPIAGALIGEF 165

> AF252855_1 florfenicol-resistance protein Flo [Escherichia coli]

Length=404

Score = 40.4 bits (93), Expect = 2e-04, Method: Compositional matrix adjust.

Identities = 45/152 (30%), Positives = 70/152 (46%), Gaps = 14/152 (9%)

Frame = +1

Query 1261 ILSTVALDAVGIGL-IMPVLPGLLRDLVHSNDVTAHYGILLALYALMQFACAPVLGALSD 1437

IL+++A+D I L ++P +PG+L N A + L+LY +M + G LSD

Sbjct 24 ILASLAMD---IYLPVVPAMPGIL------NTTPAMIQLTLSLYMVMLGVGQVIFGPLSD 74

Query 1438 RFGRRPVLL---VSLAGAAVDYAIMATAPFLWVLYigrivagitgatgavagayiaDITD 1608

R GRRP+LL + A++ A +TAP +V + G + A +

Sbjct 75 RIGRRPILLAGATAFVIASLGAAWSSTAP-AFVAFRLLQAVGASAMLVATFATVRDVYAN 133

Query 1609 GDERARHFGFMSACFGFGMVAGPVLGGLMGGF 1704

E +G S+ F GP+ G L+G F

Sbjct 134 RPEGVVIYGLFSSILAFVPALGPIAGALIGEF 165

> AAW02923 florfenicol efflux protein (plasmid) [Escherichia coli]

Length=404

Score = 40.4 bits (93), Expect = 2e-04, Method: Compositional matrix adjust.

Identities = 45/152 (30%), Positives = 70/152 (46%), Gaps = 14/152 (9%)

Frame = +1

Query 1261 ILSTVALDAVGIGL-IMPVLPGLLRDLVHSNDVTAHYGILLALYALMQFACAPVLGALSD 1437

IL+++A+D I L ++P +PG+L N A + L+LY +M + G LSD

Sbjct 24 ILASLAMD---IYLPVVPAMPGIL------NTTPAMIQLTLSLYMVMLGVGQVIFGPLSD 74

Query 1438 RFGRRPVLL---VSLAGAAVDYAIMATAPFLWVLYigrivagitgatgavagayiaDITD 1608

R GRRP+LL + A++ A +TAP +V + G + A +

Sbjct 75 RIGRRPILLAGATAFVIASLGAAWSSTAP-AFVAFRLLQAVGASAMLVATFATVRDVYAN 133

Query 1609 GDERARHFGFMSACFGFGMVAGPVLGGLMGGF 1704

E +G S+ F GP+ G L+G F

Sbjct 134 RPEGVVIYGLFSSILAFVPALGPIAGALIGEF 165

> FloR_-_24955634_translation FloR: florfenicol chloramphenicol

resistance protein FloR

Length=404

Score = 40.4 bits (93), Expect = 2e-04, Method: Compositional matrix adjust.

Identities = 45/152 (30%), Positives = 70/152 (46%), Gaps = 14/152 (9%)

Frame = +1

Query 1261 ILSTVALDAVGIGL-IMPVLPGLLRDLVHSNDVTAHYGILLALYALMQFACAPVLGALSD 1437

IL+++A+D I L ++P +PG+L N A + L+LY +M + G LSD

Sbjct 24 ILASLAMD---IYLPVVPAMPGIL------NTTPAMIQLTLSLYMVMLGVGQVIFGPLSD 74

Query 1438 RFGRRPVLL---VSLAGAAVDYAIMATAPFLWVLYigrivagitgatgavagayiaDITD 1608

R GRRP+LL + A++ A +TAP +V + G + A +

Sbjct 75 RIGRRPILLAGATAFVIASLGAAWSSTAP-AFVAFRLLQAVGASAMLVATFATVRDVYAN 133

Query 1609 GDERARHFGFMSACFGFGMVAGPVLGGLMGGF 1704

E +G S+ F GP+ G L+G F

Sbjct 134 RPEGVVIYGLFSSILAFVPALGPIAGALIGEF 165

> YP_008574689 florfenicol chloramphenicol resistance protein FloR

(plasmid) [Salmonella enterica subsp. enterica serovar Typhimurium]

Length=404

Score = 40.4 bits (93), Expect = 2e-04, Method: Compositional matrix adjust.

Identities = 45/152 (30%), Positives = 70/152 (46%), Gaps = 14/152 (9%)

Frame = +1

Query 1261 ILSTVALDAVGIGL-IMPVLPGLLRDLVHSNDVTAHYGILLALYALMQFACAPVLGALSD 1437

IL+++A+D I L ++P +PG+L N A + L+LY +M + G LSD

Sbjct 24 ILASLAMD---IYLPVVPAMPGIL------NTTPAMIQLTLSLYMVMLGVGQVIFGPLSD 74

Query 1438 RFGRRPVLL---VSLAGAAVDYAIMATAPFLWVLYigrivagitgatgavagayiaDITD 1608

R GRRP+LL + A++ A +TAP +V + G + A +

Sbjct 75 RIGRRPILLAGATAFVIASLGAAWSSTAP-AFVAFRLLQAVGASAMLVATFATVRDVYAN 133

Query 1609 GDERARHFGFMSACFGFGMVAGPVLGGLMGGF 1704

E +G S+ F GP+ G L+G F

Sbjct 134 RPEGVVIYGLFSSILAFVPALGPIAGALIGEF 165

> CAD57670 florfenicol/chloramphenicol resistance protein (plasmid)

[Escherichia coli]

Length=404

Score = 40.4 bits (93), Expect = 2e-04, Method: Compositional matrix adjust.

Identities = 45/152 (30%), Positives = 70/152 (46%), Gaps = 14/152 (9%)

Frame = +1

Query 1261 ILSTVALDAVGIGL-IMPVLPGLLRDLVHSNDVTAHYGILLALYALMQFACAPVLGALSD 1437

IL+++A+D I L ++P +PG+L N A + L+LY +M + G LSD

Sbjct 24 ILASLAMD---IYLPVVPAMPGIL------NTTPAMIQLTLSLYMVMLGVGQVIFGPLSD 74

Query 1438 RFGRRPVLL---VSLAGAAVDYAIMATAPFLWVLYigrivagitgatgavagayiaDITD 1608

R GRRP+LL + A++ A +TAP +V + G + A +

Sbjct 75 RIGRRPILLAGATAFVIASLGAAWSSTAP-AFVAFRLLQAVGASAMLVATFATVRDVYAN 133

Query 1609 GDERARHFGFMSACFGFGMVAGPVLGGLMGGF 1704

E +G S+ F GP+ G L+G F

Sbjct 134 RPEGVVIYGLFSSILAFVPALGPIAGALIGEF 165

> BAN84125 florfenicol chloramphenicol resistance protein FloR

(plasmid) [Salmonella enterica subsp. enterica serovar Typhimurium]

Length=404

Score = 40.4 bits (93), Expect = 2e-04, Method: Compositional matrix adjust.

Identities = 45/152 (30%), Positives = 70/152 (46%), Gaps = 14/152 (9%)

Frame = +1

Query 1261 ILSTVALDAVGIGL-IMPVLPGLLRDLVHSNDVTAHYGILLALYALMQFACAPVLGALSD 1437

IL+++A+D I L ++P +PG+L N A + L+LY +M + G LSD

Sbjct 24 ILASLAMD---IYLPVVPAMPGIL------NTTPAMIQLTLSLYMVMLGVGQVIFGPLSD 74

Query 1438 RFGRRPVLL---VSLAGAAVDYAIMATAPFLWVLYigrivagitgatgavagayiaDITD 1608

R GRRP+LL + A++ A +TAP +V + G + A +

Sbjct 75 RIGRRPILLAGATAFVIASLGAAWSSTAP-AFVAFRLLQAVGASAMLVATFATVRDVYAN 133

Query 1609 GDERARHFGFMSACFGFGMVAGPVLGGLMGGF 1704

E +G S+ F GP+ G L+G F

Sbjct 134 RPEGVVIYGLFSSILAFVPALGPIAGALIGEF 165

> AAS00447 florfenicol export protein [Escherichia coli]

Length=404

Score = 40.4 bits (93), Expect = 2e-04, Method: Compositional matrix adjust.

Identities = 45/152 (30%), Positives = 70/152 (46%), Gaps = 14/152 (9%)

Frame = +1

Query 1261 ILSTVALDAVGIGL-IMPVLPGLLRDLVHSNDVTAHYGILLALYALMQFACAPVLGALSD 1437

IL+++A+D I L ++P +PG+L N A + L+LY +M + G LSD

Sbjct 24 ILASLAMD---IYLPVVPAMPGIL------NTTPAMIQLTLSLYMVMLGVGQVIFGPLSD 74

Query 1438 RFGRRPVLL---VSLAGAAVDYAIMATAPFLWVLYigrivagitgatgavagayiaDITD 1608

R GRRP+LL + A++ A +TAP +V + G + A +

Sbjct 75 RIGRRPILLAGATAFVIASLGAAWSSTAP-AFVAFRLLQAVGASAMLVATFATVRDVYAN 133

Query 1609 GDERARHFGFMSACFGFGMVAGPVLGGLMGGF 1704

E +G S+ F GP+ G L+G F

Sbjct 134 RPEGVVIYGLFSSILAFVPALGPIAGALIGEF 165

> floR_-_11934756_translation floR: florfenicol/chloramphenicol

resistance protein FloR

Length=404

Score = 40.0 bits (92), Expect = 2e-04, Method: Compositional matrix adjust.

Identities = 45/152 (30%), Positives = 70/152 (46%), Gaps = 14/152 (9%)

Frame = +1

Query 1261 ILSTVALDAVGIGL-IMPVLPGLLRDLVHSNDVTAHYGILLALYALMQFACAPVLGALSD 1437

IL+++A+D I L ++P +PG+L N A + L+LY +M + G LSD

Sbjct 24 ILASLAMD---IYLPVVPAMPGIL------NTTPAMIQLTLSLYMVMLGVGQVIFGPLSD 74

Query 1438 RFGRRPVLL---VSLAGAAVDYAIMATAPFLWVLYigrivagitgatgavagayiaDITD 1608

R GRRP+LL + A++ A +TAP +V + G + A +

Sbjct 75 RIGRRPILLAGATAFVIASLGAAWSSTAP-AFVAFRLLQAVGASAMLVATFATVRDVYAN 133

Query 1609 GDERARHFGFMSACFGFGMVAGPVLGGLMGGF 1704

E +G S+ F GP+ G L+G F

Sbjct 134 RPEGVVIYGLFSSILAFVPALGPIAGALIGEF 165

> AIF79467 FloR (plasmid) [Escherichia coli]

Length=404

Score = 40.0 bits (92), Expect = 2e-04, Method: Compositional matrix adjust.

Identities = 45/152 (30%), Positives = 70/152 (46%), Gaps = 14/152 (9%)

Frame = +1

Query 1261 ILSTVALDAVGIGL-IMPVLPGLLRDLVHSNDVTAHYGILLALYALMQFACAPVLGALSD 1437

IL+++A+D I L ++P +PG+L N A + L+LY +M + G LSD

Sbjct 24 ILASLAMD---IYLPVVPAMPGIL------NTTPAMIQLTLSLYMVMLGVGQVIFGPLSD 74

Query 1438 RFGRRPVLL---VSLAGAAVDYAIMATAPFLWVLYigrivagitgatgavagayiaDITD 1608

R GRRP+LL + A++ A +TAP +V + G + A +

Sbjct 75 RIGRRPILLAGATAFVIASLGAAWSSTAP-AFVAFRLLQAVGASAMLVATFATVRDVYAN 133

Query 1609 GDERARHFGFMSACFGFGMVAGPVLGGLMGGF 1704

E +G S+ F GP+ G L+G F

Sbjct 134 RPEGVVIYGLFSSMLAFVPALGPIAGALIGEF 165

> SFV91173 Chloramphenicol and florfenicol resistance transporter

protein (Major facilitator superfamily) [Morganella morganii]

Length=404

Score = 40.0 bits (92), Expect = 2e-04, Method: Compositional matrix adjust.

Identities = 45/152 (30%), Positives = 70/152 (46%), Gaps = 14/152 (9%)

Frame = +1

Query 1261 ILSTVALDAVGIGL-IMPVLPGLLRDLVHSNDVTAHYGILLALYALMQFACAPVLGALSD 1437

IL+++A+D I L ++P +PG+L N A + L+LY +M + G LSD

Sbjct 24 ILASLAMD---IYLPVVPAMPGIL------NTTPAMIQLTLSLYMVMLGVGQVIFGPLSD 74

Query 1438 RFGRRPVLL---VSLAGAAVDYAIMATAPFLWVLYigrivagitgatgavagayiaDITD 1608

R GRRP+LL + A++ A +TAP +V + G + A +

Sbjct 75 RIGRRPILLAGATAFVIASLGAAWSSTAP-AFVAFRLLQAVGASAMLVATFATVRDVYAN 133

Query 1609 GDERARHFGFMSACFGFGMVAGPVLGGLMGGF 1704

E +G S+ F GP+ G L+G F

Sbjct 134 RPEGVVIYGLFSSMLAFVPALGPIAGALIGEF 165

> ELX29704 florfenicol exporter [Salmonella enterica subsp. enterica

serovar 4,[5],12:i:- str. 08-1739]

Length=404

Score = 40.0 bits (92), Expect = 2e-04, Method: Compositional matrix adjust.

Identities = 45/152 (30%), Positives = 70/152 (46%), Gaps = 14/152 (9%)

Frame = +1

Query 1261 ILSTVALDAVGIGL-IMPVLPGLLRDLVHSNDVTAHYGILLALYALMQFACAPVLGALSD 1437

IL+++A+D I L ++P +PG+L N A + L+LY +M + G LSD

Sbjct 24 ILASLAMD---IYLPVVPAMPGIL------NTTPAMIQLTLSLYMVMLGVGQVIFGPLSD 74

Query 1438 RFGRRPVLL---VSLAGAAVDYAIMATAPFLWVLYigrivagitgatgavagayiaDITD 1608

R GRRP+LL + A++ A +TAP +V + G + A +

Sbjct 75 RIGRRPILLAGATAFVIASLGAAWSSTAP-AFVAFRLLQAVGASAMLVATFATVRDVYAN 133

Query 1609 GDERARHFGFMSACFGFGMVAGPVLGGLMGGF 1704

E +G S+ F GP+ G L+G F

Sbjct 134 RPEGVVIYGLFSSMLAFVPALGPIAGALIGEF 165

> CCW76557 chloramphenicol and florfenicol resistance protein [Salmonella

enterica subsp. enterica serovar Typhimurium str.

DT104]

Length=404

Score = 40.0 bits (92), Expect = 2e-04, Method: Compositional matrix adjust.

Identities = 45/152 (30%), Positives = 70/152 (46%), Gaps = 14/152 (9%)

Frame = +1

Query 1261 ILSTVALDAVGIGL-IMPVLPGLLRDLVHSNDVTAHYGILLALYALMQFACAPVLGALSD 1437

IL+++A+D I L ++P +PG+L N A + L+LY +M + G LSD

Sbjct 24 ILASLAMD---IYLPVVPAMPGIL------NTTPAMIQLTLSLYMVMLGVGQVIFGPLSD 74

Query 1438 RFGRRPVLL---VSLAGAAVDYAIMATAPFLWVLYigrivagitgatgavagayiaDITD 1608

R GRRP+LL + A++ A +TAP +V + G + A +

Sbjct 75 RIGRRPILLAGATAFVIASLGAAWSSTAP-AFVAFRLLQAVGASAMLVATFATVRDVYAN 133

Query 1609 GDERARHFGFMSACFGFGMVAGPVLGGLMGGF 1704

E +G S+ F GP+ G L+G F

Sbjct 134 RPEGVVIYGLFSSMLAFVPALGPIAGALIGEF 165

> AQY10149 chloramphenicol and florfenicol resistance protein [Salmonella

enterica]

Length=404

Score = 40.0 bits (92), Expect = 2e-04, Method: Compositional matrix adjust.

Identities = 45/152 (30%), Positives = 70/152 (46%), Gaps = 14/152 (9%)

Frame = +1

Query 1261 ILSTVALDAVGIGL-IMPVLPGLLRDLVHSNDVTAHYGILLALYALMQFACAPVLGALSD 1437

IL+++A+D I L ++P +PG+L N A + L+LY +M + G LSD

Sbjct 24 ILASLAMD---IYLPVVPAMPGIL------NTTPAMIQLTLSLYMVMLGVGQVIFGPLSD 74

Query 1438 RFGRRPVLL---VSLAGAAVDYAIMATAPFLWVLYigrivagitgatgavagayiaDITD 1608

R GRRP+LL + A++ A +TAP +V + G + A +

Sbjct 75 RIGRRPILLAGATAFVIASLGAAWSSTAP-AFVAFRLLQAVGASAMLVATFATVRDVYAN 133

Query 1609 GDERARHFGFMSACFGFGMVAGPVLGGLMGGF 1704

E +G S+ F GP+ G L+G F

Sbjct 134 RPEGVVIYGLFSSMLAFVPALGPIAGALIGEF 165

> ANA09865 chloramphenicol and florfenicol resistance protein [Salmonella

enterica subsp. enterica]

Length=404

Score = 40.0 bits (92), Expect = 2e-04, Method: Compositional matrix adjust.

Identities = 45/152 (30%), Positives = 70/152 (46%), Gaps = 14/152 (9%)

Frame = +1

Query 1261 ILSTVALDAVGIGL-IMPVLPGLLRDLVHSNDVTAHYGILLALYALMQFACAPVLGALSD 1437

IL+++A+D I L ++P +PG+L N A + L+LY +M + G LSD

Sbjct 24 ILASLAMD---IYLPVVPAMPGIL------NTTPAMIQLTLSLYMVMLGVGQVIFGPLSD 74

Query 1438 RFGRRPVLL---VSLAGAAVDYAIMATAPFLWVLYigrivagitgatgavagayiaDITD 1608

R GRRP+LL + A++ A +TAP +V + G + A +

Sbjct 75 RIGRRPILLAGATAFVIASLGAAWSSTAP-AFVAFRLLQAVGASAMLVATFATVRDVYAN 133

Query 1609 GDERARHFGFMSACFGFGMVAGPVLGGLMGGF 1704

E +G S+ F GP+ G L+G F

Sbjct 134 RPEGVVIYGLFSSMLAFVPALGPIAGALIGEF 165

> AMQ95871 FloR [Salmonella enterica subsp. enterica serovar Cerro]

Length=404

Score = 40.0 bits (92), Expect = 2e-04, Method: Compositional matrix adjust.

Identities = 45/152 (30%), Positives = 70/152 (46%), Gaps = 14/152 (9%)

Frame = +1

Query 1261 ILSTVALDAVGIGL-IMPVLPGLLRDLVHSNDVTAHYGILLALYALMQFACAPVLGALSD 1437

IL+++A+D I L ++P +PG+L N A + L+LY +M + G LSD

Sbjct 24 ILASLAMD---IYLPVVPAMPGIL------NTTPAMIQLTLSLYMVMLGVGQVIFGPLSD 74

Query 1438 RFGRRPVLL---VSLAGAAVDYAIMATAPFLWVLYigrivagitgatgavagayiaDITD 1608

R GRRP+LL + A++ A +TAP +V + G + A +

Sbjct 75 RIGRRPILLAGATAFVIASLGAAWSSTAP-AFVAFRLLQAVGASAMLVATFATVRDVYAN 133

Query 1609 GDERARHFGFMSACFGFGMVAGPVLGGLMGGF 1704

E +G S+ F GP+ G L+G F

Sbjct 134 RPEGVVIYGLFSSMLAFVPALGPIAGALIGEF 165

> AMP35663 FloR [Salmonella enterica]

Length=404

Score = 40.0 bits (92), Expect = 2e-04, Method: Compositional matrix adjust.

Identities = 45/152 (30%), Positives = 70/152 (46%), Gaps = 14/152 (9%)

Frame = +1

Query 1261 ILSTVALDAVGIGL-IMPVLPGLLRDLVHSNDVTAHYGILLALYALMQFACAPVLGALSD 1437

IL+++A+D I L ++P +PG+L N A + L+LY +M + G LSD

Sbjct 24 ILASLAMD---IYLPVVPAMPGIL------NTTPAMIQLTLSLYMVMLGVGQVIFGPLSD 74

Query 1438 RFGRRPVLL---VSLAGAAVDYAIMATAPFLWVLYigrivagitgatgavagayiaDITD 1608

R GRRP+LL + A++ A +TAP +V + G + A +

Sbjct 75 RIGRRPILLAGATAFVIASLGAAWSSTAP-AFVAFRLLQAVGASAMLVATFATVRDVYAN 133

Query 1609 GDERARHFGFMSACFGFGMVAGPVLGGLMGGF 1704

E +G S+ F GP+ G L+G F

Sbjct 134 RPEGVVIYGLFSSMLAFVPALGPIAGALIGEF 165

> AIW55480 chloramphenicol and florfenicol resistance protein [Proteus

mirabilis]

Length=404

Score = 40.0 bits (92), Expect = 2e-04, Method: Compositional matrix adjust.

Identities = 45/152 (30%), Positives = 70/152 (46%), Gaps = 14/152 (9%)

Frame = +1

Query 1261 ILSTVALDAVGIGL-IMPVLPGLLRDLVHSNDVTAHYGILLALYALMQFACAPVLGALSD 1437

IL+++A+D I L ++P +PG+L N A + L+LY +M + G LSD

Sbjct 24 ILASLAMD---IYLPVVPAMPGIL------NTTPAMIQLTLSLYMVMLGVGQVIFGPLSD 74

Query 1438 RFGRRPVLL---VSLAGAAVDYAIMATAPFLWVLYigrivagitgatgavagayiaDITD 1608

R GRRP+LL + A++ A +TAP +V + G + A +

Sbjct 75 RIGRRPILLAGATAFVIASLGAAWSSTAP-AFVAFRLLQAVGASAMLVATFATVRDVYAN 133

Query 1609 GDERARHFGFMSACFGFGMVAGPVLGGLMGGF 1704

E +G S+ F GP+ G L+G F

Sbjct 134 RPEGVVIYGLFSSMLAFVPALGPIAGALIGEF 165

> AIW55431 chloramphenicol and florfenicol resistance protein [Proteus

mirabilis]

Length=404

Score = 40.0 bits (92), Expect = 2e-04, Method: Compositional matrix adjust.

Identities = 45/152 (30%), Positives = 70/152 (46%), Gaps = 14/152 (9%)

Frame = +1

Query 1261 ILSTVALDAVGIGL-IMPVLPGLLRDLVHSNDVTAHYGILLALYALMQFACAPVLGALSD 1437

IL+++A+D I L ++P +PG+L N A + L+LY +M + G LSD

Sbjct 24 ILASLAMD---IYLPVVPAMPGIL------NTTPAMIQLTLSLYMVMLGVGQVIFGPLSD 74

Query 1438 RFGRRPVLL---VSLAGAAVDYAIMATAPFLWVLYigrivagitgatgavagayiaDITD 1608

R GRRP+LL + A++ A +TAP +V + G + A +

Sbjct 75 RIGRRPILLAGATAFVIASLGAAWSSTAP-AFVAFRLLQAVGASAMLVATFATVRDVYAN 133

Query 1609 GDERARHFGFMSACFGFGMVAGPVLGGLMGGF 1704

E +G S+ F GP+ G L+G F

Sbjct 134 RPEGVVIYGLFSSMLAFVPALGPIAGALIGEF 165

> AIU97987 chloramphenicol and florfenicol resistance protein [Salmonella

enterica subsp. enterica]

Length=404

Score = 40.0 bits (92), Expect = 2e-04, Method: Compositional matrix adjust.

Identities = 45/152 (30%), Positives = 70/152 (46%), Gaps = 14/152 (9%)

Frame = +1

Query 1261 ILSTVALDAVGIGL-IMPVLPGLLRDLVHSNDVTAHYGILLALYALMQFACAPVLGALSD 1437

IL+++A+D I L ++P +PG+L N A + L+LY +M + G LSD

Sbjct 24 ILASLAMD---IYLPVVPAMPGIL------NTTPAMIQLTLSLYMVMLGVGQVIFGPLSD 74

Query 1438 RFGRRPVLL---VSLAGAAVDYAIMATAPFLWVLYigrivagitgatgavagayiaDITD 1608

R GRRP+LL + A++ A +TAP +V + G + A +

Sbjct 75 RIGRRPILLAGATAFVIASLGAAWSSTAP-AFVAFRLLQAVGASAMLVATFATVRDVYAN 133

Query 1609 GDERARHFGFMSACFGFGMVAGPVLGGLMGGF 1704

E +G S+ F GP+ G L+G F

Sbjct 134 RPEGVVIYGLFSSMLAFVPALGPIAGALIGEF 165

> AGK07102 chloramphenicol and florfenicol resistance protein [Proteus

mirabilis]

Length=404

Score = 40.0 bits (92), Expect = 2e-04, Method: Compositional matrix adjust.

Identities = 45/152 (30%), Positives = 70/152 (46%), Gaps = 14/152 (9%)

Frame = +1

Query 1261 ILSTVALDAVGIGL-IMPVLPGLLRDLVHSNDVTAHYGILLALYALMQFACAPVLGALSD 1437

IL+++A+D I L ++P +PG+L N A + L+LY +M + G LSD

Sbjct 24 ILASLAMD---IYLPVVPAMPGIL------NTTPAMIQLTLSLYMVMLGVGQVIFGPLSD 74

Query 1438 RFGRRPVLL---VSLAGAAVDYAIMATAPFLWVLYigrivagitgatgavagayiaDITD 1608

R GRRP+LL + A++ A +TAP +V + G + A +

Sbjct 75 RIGRRPILLAGATAFVIASLGAAWSSTAP-AFVAFRLLQAVGASAMLVATFATVRDVYAN 133

Query 1609 GDERARHFGFMSACFGFGMVAGPVLGGLMGGF 1704

E +G S+ F GP+ G L+G F

Sbjct 134 RPEGVVIYGLFSSMLAFVPALGPIAGALIGEF 165

> AGK06972 chloramphenicol and florfenicol resistance protein [Proteus

mirabilis]

Length=404

Score = 40.0 bits (92), Expect = 2e-04, Method: Compositional matrix adjust.

Identities = 45/152 (30%), Positives = 70/152 (46%), Gaps = 14/152 (9%)

Frame = +1

Query 1261 ILSTVALDAVGIGL-IMPVLPGLLRDLVHSNDVTAHYGILLALYALMQFACAPVLGALSD 1437

IL+++A+D I L ++P +PG+L N A + L+LY +M + G LSD

Sbjct 24 ILASLAMD---IYLPVVPAMPGIL------NTTPAMIQLTLSLYMVMLGVGQVIFGPLSD 74

Query 1438 RFGRRPVLL---VSLAGAAVDYAIMATAPFLWVLYigrivagitgatgavagayiaDITD 1608

R GRRP+LL + A++ A +TAP +V + G + A +

Sbjct 75 RIGRRPILLAGATAFVIASLGAAWSSTAP-AFVAFRLLQAVGASAMLVATFATVRDVYAN 133

Query 1609 GDERARHFGFMSACFGFGMVAGPVLGGLMGGF 1704

E +G S+ F GP+ G L+G F

Sbjct 134 RPEGVVIYGLFSSMLAFVPALGPIAGALIGEF 165

> AF261825_18 chloramphenicol and florfenicol resistance protein

[Salmonella enterica subsp. enterica serovar Typhimurium]

Length=404

Score = 40.0 bits (92), Expect = 2e-04, Method: Compositional matrix adjust.

Identities = 45/152 (30%), Positives = 70/152 (46%), Gaps = 14/152 (9%)

Frame = +1

Query 1261 ILSTVALDAVGIGL-IMPVLPGLLRDLVHSNDVTAHYGILLALYALMQFACAPVLGALSD 1437

IL+++A+D I L ++P +PG+L N A + L+LY +M + G LSD

Sbjct 24 ILASLAMD---IYLPVVPAMPGIL------NTTPAMIQLTLSLYMVMLGVGQVIFGPLSD 74

Query 1438 RFGRRPVLL---VSLAGAAVDYAIMATAPFLWVLYigrivagitgatgavagayiaDITD 1608

R GRRP+LL + A++ A +TAP +V + G + A +

Sbjct 75 RIGRRPILLAGATAFVIASLGAAWSSTAP-AFVAFRLLQAVGASAMLVATFATVRDVYAN 133

Query 1609 GDERARHFGFMSACFGFGMVAGPVLGGLMGGF 1704

E +G S+ F GP+ G L+G F

Sbjct 134 RPEGVVIYGLFSSMLAFVPALGPIAGALIGEF 165

> AF118107_2 putative efflux protein Flor [Salmonella enterica

subsp. enterica serovar Typhimurium str. DT104]

Length=404

Score = 40.0 bits (92), Expect = 2e-04, Method: Compositional matrix adjust.

Identities = 45/152 (30%), Positives = 70/152 (46%), Gaps = 14/152 (9%)

Frame = +1

Query 1261 ILSTVALDAVGIGL-IMPVLPGLLRDLVHSNDVTAHYGILLALYALMQFACAPVLGALSD 1437

IL+++A+D I L ++P +PG+L N A + L+LY +M + G LSD

Sbjct 24 ILASLAMD---IYLPVVPAMPGIL------NTTPAMIQLTLSLYMVMLGVGQVIFGPLSD 74

Query 1438 RFGRRPVLL---VSLAGAAVDYAIMATAPFLWVLYigrivagitgatgavagayiaDITD 1608

R GRRP+LL + A++ A +TAP +V + G + A +

Sbjct 75 RIGRRPILLAGATAFVIASLGAAWSSTAP-AFVAFRLLQAVGASAMLVATFATVRDVYAN 133

Query 1609 GDERARHFGFMSACFGFGMVAGPVLGGLMGGF 1704

E +G S+ F GP+ G L+G F

Sbjct 134 RPEGVVIYGLFSSMLAFVPALGPIAGALIGEF 165

> AMQ12815 FloR (plasmid) [Escherichia coli]

Length=404

Score = 40.0 bits (92), Expect = 2e-04, Method: Compositional matrix adjust.

Identities = 44/151 (29%), Positives = 68/151 (45%), Gaps = 12/151 (8%)

Frame = +1

Query 1261 ILSTVALDAVGIGLIMPVLPGLLRDLVHSNDVTAHYGILLALYALMQFACAPVLGALSDR 1440

IL+++A+D L +PV+P + L N A + L+LY +M + G LSDR

Sbjct 24 ILASLAMD-----LYLPVVPAMPGIL---NTTPAMIQLTLSLYMVMLGVGQVIFGPLSDR 75

Query 1441 FGRRPVLL---VSLAGAAVDYAIMATAPFLWVLYigrivagitgatgavagayiaDITDG 1611

GRRP+LL + A++ A +TAP +V + G + A +

Sbjct 76 IGRRPILLAGATAFVIASLGAAWSSTAP-AFVAFRLLQAVGASAMLVATFATVRDVYANR 134

Query 1612 DERARHFGFMSACFGFGMVAGPVLGGLMGGF 1704

E +G S+ F GP+ G L+G F

Sbjct 135 PEGVVIYGLFSSILAFVPALGPIAGALIGEF 165

> ABR22528 florfenicol resistance protein, partial [Vibrio tasmaniensis]

Length=283

Score = 39.7 bits (91), Expect = 2e-04, Method: Compositional matrix adjust.

Identities = 39/137 (28%), Positives = 60/137 (44%), Gaps = 10/137 (7%)

Frame = +1

Query 1303 IMPVLPGLLRDLVHSNDVTAHYGILLALYALMQFACAPVLGALSDRFGRRPVLL---VSL 1473

++P +PG+L N A + L+LY +M + G LSDR GRRP+LL +

Sbjct 9 VVPAMPGIL------NTTPAMIQLTLSLYMVMLGVGQVIFGPLSDRIGRRPILLAGATAF 62

Query 1474 AGAAVDYAIMATAPFLWVLYigrivagitgatgavagayiaDITDGDERARHFGFMSACF 1653

A++ A +TAP +V + G + A + E +G S+

Sbjct 63 VIASLGAAWSSTAP-AFVAFRLLQAVGASAMLVATFATVRDVYANRPEGVVIYGLFSSML 121

Query 1654 GFGMVAGPVLGGLMGGF 1704

F GP+ G L+G F

Sbjct 122 AFVPALGPIAGALIGEF 138

> YP_002286768 phenicol exporter (plasmid) [Pasteurella multocida]

Length=404

Score = 40.0 bits (92), Expect = 2e-04, Method: Compositional matrix adjust.

Identities = 45/152 (30%), Positives = 70/152 (46%), Gaps = 14/152 (9%)

Frame = +1

Query 1261 ILSTVALDAVGIGL-IMPVLPGLLRDLVHSNDVTAHYGILLALYALMQFACAPVLGALSD 1437

IL+++A+D I L ++P +PG+L N A + L+LY +M + G LSD

Sbjct 24 ILASLAMD---IYLPVVPAMPGIL------NTTPAMIQLTLSLYMVMLGVGQVIFGPLSD 74

Query 1438 RFGRRPVLL---VSLAGAAVDYAIMATAPFLWVLYigrivagitgatgavagayiaDITD 1608

R GRRP+LL + A++ A +TAP +V + G + A +

Sbjct 75 RIGRRPILLAGATAFVIASLGAAWSSTAP-AFVAFRLLQAVGASAMLVATFATVRDVYAN 133

Query 1609 GDERARHFGFMSACFGFGMVAGPVLGGLMGGF 1704

E +G S+ F GP+ G L+G F

Sbjct 134 RPEGVVIYGLFSSMLAFVPALGPIAGALIGEF 165

> WP_012561099 MULTISPECIES: chloramphenicol/florfenicol efflux

MFS transporter FloR [Gammaproteobacteria]

Length=404

Score = 40.0 bits (92), Expect = 2e-04, Method: Compositional matrix adjust.

Identities = 45/152 (30%), Positives = 70/152 (46%), Gaps = 14/152 (9%)

Frame = +1

Query 1261 ILSTVALDAVGIGL-IMPVLPGLLRDLVHSNDVTAHYGILLALYALMQFACAPVLGALSD 1437

IL+++A+D I L ++P +PG+L N A + L+LY +M + G LSD

Sbjct 24 ILASLAMD---IYLPVVPAMPGIL------NTTPAMIQLTLSLYMVMLGVGQVIFGPLSD 74

Query 1438 RFGRRPVLL---VSLAGAAVDYAIMATAPFLWVLYigrivagitgatgavagayiaDITD 1608

R GRRP+LL + A++ A +TAP +V + G + A +

Sbjct 75 RIGRRPILLAGATAFVIASLGAAWSSTAP-AFVAFRLLQAVGASAMLVATFATVRDVYAN 133

Query 1609 GDERARHFGFMSACFGFGMVAGPVLGGLMGGF 1704

E +G S+ F GP+ G L+G F

Sbjct 134 RPEGVVIYGLFSSMLAFVPALGPIAGALIGEF 165

> CAQ77171 phenicol exporter (plasmid) [Pasteurella multocida]

Length=404

Score = 40.0 bits (92), Expect = 2e-04, Method: Compositional matrix adjust.

Identities = 45/152 (30%), Positives = 70/152 (46%), Gaps = 14/152 (9%)

Frame = +1

Query 1261 ILSTVALDAVGIGL-IMPVLPGLLRDLVHSNDVTAHYGILLALYALMQFACAPVLGALSD 1437

IL+++A+D I L ++P +PG+L N A + L+LY +M + G LSD

Sbjct 24 ILASLAMD---IYLPVVPAMPGIL------NTTPAMIQLTLSLYMVMLGVGQVIFGPLSD 74

Query 1438 RFGRRPVLL---VSLAGAAVDYAIMATAPFLWVLYigrivagitgatgavagayiaDITD 1608

R GRRP+LL + A++ A +TAP +V + G + A +

Sbjct 75 RIGRRPILLAGATAFVIASLGAAWSSTAP-AFVAFRLLQAVGASAMLVATFATVRDVYAN 133

Query 1609 GDERARHFGFMSACFGFGMVAGPVLGGLMGGF 1704

E +G S+ F GP+ G L+G F

Sbjct 134 RPEGVVIYGLFSSMLAFVPALGPIAGALIGEF 165

> floR_-_3362475_translation floR: florfenicol-chloramphenicol

exporter

Length=404

Score = 40.0 bits (92), Expect = 2e-04, Method: Compositional matrix adjust.

Identities = 45/152 (30%), Positives = 70/152 (46%), Gaps = 14/152 (9%)

Frame = +1

Query 1261 ILSTVALDAVGIGL-IMPVLPGLLRDLVHSNDVTAHYGILLALYALMQFACAPVLGALSD 1437

IL+++A+D I L ++P +PG+L N A + L+LY +M + G LSD

Sbjct 24 ILASLAMD---IYLPVVPAMPGIL------NTTPAMIQLTLSLYMVMLGVGQVIFGPLSD 74

Query 1438 RFGRRPVLL---VSLAGAAVDYAIMATAPFLWVLYigrivagitgatgavagayiaDITD 1608

R GRRP+LL + A++ A +TAP +V + G + A +

Sbjct 75 RIGRRPILLAGATAFVIASLGAAWSSTAP-AFVAFRLLQAVGASAMLVATFATVRDVYAN 133

Query 1609 GDERARHFGFMSACFGFGMVAGPVLGGLMGGF 1704

E +G S+ F GP+ G L+G F

Sbjct 134 RPEGVVIYGLFSSMLAFVPALGPIAGALIGEF 165

> YP_232872 florfenicol-chloramphenicol exporter (plasmid) [Pasteurella

multocida]

Length=404

Score = 40.0 bits (92), Expect = 2e-04, Method: Compositional matrix adjust.

Identities = 45/152 (30%), Positives = 70/152 (46%), Gaps = 14/152 (9%)

Frame = +1

Query 1261 ILSTVALDAVGIGL-IMPVLPGLLRDLVHSNDVTAHYGILLALYALMQFACAPVLGALSD 1437

IL+++A+D I L ++P +PG+L N A + L+LY +M + G LSD

Sbjct 24 ILASLAMD---IYLPVVPAMPGIL------NTTPAMIQLTLSLYMVMLGVGQVIFGPLSD 74

Query 1438 RFGRRPVLL---VSLAGAAVDYAIMATAPFLWVLYigrivagitgatgavagayiaDITD 1608

R GRRP+LL + A++ A +TAP +V + G + A +

Sbjct 75 RIGRRPILLAGATAFVIASLGAAWSSTAP-AFVAFRLLQAVGASAMLVATFATVRDVYAN 133

Query 1609 GDERARHFGFMSACFGFGMVAGPVLGGLMGGF 1704

E +G S+ F GP+ G L+G F

Sbjct 134 RPEGVVIYGLFSSMLAFVPALGPIAGALIGEF 165

> WP_011266118 chloramphenicol/florfenicol efflux MFS transporter

FloR [Pasteurella multocida]

Length=404

Score = 40.0 bits (92), Expect = 2e-04, Method: Compositional matrix adjust.

Identities = 45/152 (30%), Positives = 70/152 (46%), Gaps = 14/152 (9%)

Frame = +1

Query 1261 ILSTVALDAVGIGL-IMPVLPGLLRDLVHSNDVTAHYGILLALYALMQFACAPVLGALSD 1437

IL+++A+D I L ++P +PG+L N A + L+LY +M + G LSD

Sbjct 24 ILASLAMD---IYLPVVPAMPGIL------NTTPAMIQLTLSLYMVMLGVGQVIFGPLSD 74

Query 1438 RFGRRPVLL---VSLAGAAVDYAIMATAPFLWVLYigrivagitgatgavagayiaDITD 1608

R GRRP+LL + A++ A +TAP +V + G + A +

Sbjct 75 RIGRRPILLAGATAFVIASLGAAWSSTAP-AFVAFRLLQAVGASAMLVATFATVRDVYAN 133

Query 1609 GDERARHFGFMSACFGFGMVAGPVLGGLMGGF 1704

E +G S+ F GP+ G L+G F

Sbjct 134 RPEGVVIYGLFSSMLAFVPALGPIAGALIGEF 165

> CAI43272 florfenicol-chloramphenicol exporter (plasmid) [Pasteurella

multocida]

Length=404

Score = 40.0 bits (92), Expect = 2e-04, Method: Compositional matrix adjust.

Identities = 45/152 (30%), Positives = 70/152 (46%), Gaps = 14/152 (9%)

Frame = +1

Query 1261 ILSTVALDAVGIGL-IMPVLPGLLRDLVHSNDVTAHYGILLALYALMQFACAPVLGALSD 1437

IL+++A+D I L ++P +PG+L N A + L+LY +M + G LSD

Sbjct 24 ILASLAMD---IYLPVVPAMPGIL------NTTPAMIQLTLSLYMVMLGVGQVIFGPLSD 74

Query 1438 RFGRRPVLL---VSLAGAAVDYAIMATAPFLWVLYigrivagitgatgavagayiaDITD 1608

R GRRP+LL + A++ A +TAP +V + G + A +

Sbjct 75 RIGRRPILLAGATAFVIASLGAAWSSTAP-AFVAFRLLQAVGASAMLVATFATVRDVYAN 133

Query 1609 GDERARHFGFMSACFGFGMVAGPVLGGLMGGF 1704

E +G S+ F GP+ G L+G F

Sbjct 134 RPEGVVIYGLFSSMLAFVPALGPIAGALIGEF 165

> floR_-_17434068_translation floR: Florfenicol/Chloramphenicol

efflux protein

Length=404

Score = 40.0 bits (92), Expect = 2e-04, Method: Compositional matrix adjust.

Identities = 45/152 (30%), Positives = 70/152 (46%), Gaps = 14/152 (9%)

Frame = +1

Query 1261 ILSTVALDAVGIGL-IMPVLPGLLRDLVHSNDVTAHYGILLALYALMQFACAPVLGALSD 1437

IL+++A+D I L ++P +PG+L N A + L+LY +M + G LSD

Sbjct 24 ILASLAMD---IYLPVVPAMPGIL------NTTPAMIQLTLSLYMVMLGVGQVIFGPLSD 74

Query 1438 RFGRRPVLL---VSLAGAAVDYAIMATAPFLWVLYigrivagitgatgavagayiaDITD 1608

R GRRP+LL + A++ A +TAP +V + G + A +

Sbjct 75 RIGRRPILLAGATAFVIASLGAAWSSTAP-AFVAFRLLQAVGASAMLVATFATVRDVYAN 133

Query 1609 GDERARHFGFMSACFGFGMVAGPVLGGLMGGF 1704

E +G S+ F GP+ G L+G F

Sbjct 134 RPEGVVIYGLFSSMLAFVPALGPIAGALIGEF 165

> floR_-_13917491_translation floR: FloR

Length=404

Score = 40.0 bits (92), Expect = 2e-04, Method: Compositional matrix adjust.

Identities = 45/152 (30%), Positives = 70/152 (46%), Gaps = 14/152 (9%)

Frame = +1

Query 1261 ILSTVALDAVGIGL-IMPVLPGLLRDLVHSNDVTAHYGILLALYALMQFACAPVLGALSD 1437

IL+++A+D I L ++P +PG+L N A + L+LY +M + G LSD

Sbjct 24 ILASLAMD---IYLPVVPAMPGIL------NTTPAMIQLTLSLYMVMLGVGQVIFGPLSD 74

Query 1438 RFGRRPVLL---VSLAGAAVDYAIMATAPFLWVLYigrivagitgatgavagayiaDITD 1608

R GRRP+LL + A++ A +TAP +V + G + A +

Sbjct 75 RIGRRPILLAGATAFVIASLGAAWSSTAP-AFVAFRLLQAVGASAMLVATFATVRDVYAN 133

Query 1609 GDERARHFGFMSACFGFGMVAGPVLGGLMGGF 1704

E +G S+ F GP+ G L+G F

Sbjct 134 RPEGVVIYGLFSSMLAFVPALGPIAGALIGEF 165

> floR_-_13911567_translation floR: florfenicol/ chloramphenicol

resistance protein

Length=404

Score = 40.0 bits (92), Expect = 2e-04, Method: Compositional matrix adjust.

Identities = 45/152 (30%), Positives = 70/152 (46%), Gaps = 14/152 (9%)

Frame = +1

Query 1261 ILSTVALDAVGIGL-IMPVLPGLLRDLVHSNDVTAHYGILLALYALMQFACAPVLGALSD 1437

IL+++A+D I L ++P +PG+L N A + L+LY +M + G LSD

Sbjct 24 ILASLAMD---IYLPVVPAMPGIL------NTTPAMIQLTLSLYMVMLGVGQVIFGPLSD 74

Query 1438 RFGRRPVLL---VSLAGAAVDYAIMATAPFLWVLYigrivagitgatgavagayiaDITD 1608

R GRRP+LL + A++ A +TAP +V + G + A +

Sbjct 75 RIGRRPILLAGATAFVIASLGAAWSSTAP-AFVAFRLLQAVGASAMLVATFATVRDVYAN 133

Query 1609 GDERARHFGFMSACFGFGMVAGPVLGGLMGGF 1704

E +G S+ F GP+ G L+G F

Sbjct 134 RPEGVVIYGLFSSMLAFVPALGPIAGALIGEF 165

> floR_-_13911262_translation floR: florfenicol/ chloramphenicol

resistance protein

Length=404

Score = 40.0 bits (92), Expect = 2e-04, Method: Compositional matrix adjust.

Identities = 45/152 (30%), Positives = 70/152 (46%), Gaps = 14/152 (9%)

Frame = +1

Query 1261 ILSTVALDAVGIGL-IMPVLPGLLRDLVHSNDVTAHYGILLALYALMQFACAPVLGALSD 1437

IL+++A+D I L ++P +PG+L N A + L+LY +M + G LSD

Sbjct 24 ILASLAMD---IYLPVVPAMPGIL------NTTPAMIQLTLSLYMVMLGVGQVIFGPLSD 74

Query 1438 RFGRRPVLL---VSLAGAAVDYAIMATAPFLWVLYigrivagitgatgavagayiaDITD 1608

R GRRP+LL + A++ A +TAP +V + G + A +

Sbjct 75 RIGRRPILLAGATAFVIASLGAAWSSTAP-AFVAFRLLQAVGASAMLVATFATVRDVYAN 133

Query 1609 GDERARHFGFMSACFGFGMVAGPVLGGLMGGF 1704

E +G S+ F GP+ G L+G F

Sbjct 134 RPEGVVIYGLFSSMLAFVPALGPIAGALIGEF 165

> floR_-_13905788_translation floR: florfenicol/chloramphenicol

resistance protein FloR

Length=404

Score = 40.0 bits (92), Expect = 2e-04, Method: Compositional matrix adjust.

Identities = 45/152 (30%), Positives = 70/152 (46%), Gaps = 14/152 (9%)

Frame = +1

Query 1261 ILSTVALDAVGIGL-IMPVLPGLLRDLVHSNDVTAHYGILLALYALMQFACAPVLGALSD 1437

IL+++A+D I L ++P +PG+L N A + L+LY +M + G LSD

Sbjct 24 ILASLAMD---IYLPVVPAMPGIL------NTTPAMIQLTLSLYMVMLGVGQVIFGPLSD 74

Query 1438 RFGRRPVLL---VSLAGAAVDYAIMATAPFLWVLYigrivagitgatgavagayiaDITD 1608

R GRRP+LL + A++ A +TAP +V + G + A +

Sbjct 75 RIGRRPILLAGATAFVIASLGAAWSSTAP-AFVAFRLLQAVGASAMLVATFATVRDVYAN 133

Query 1609 GDERARHFGFMSACFGFGMVAGPVLGGLMGGF 1704

E +G S+ F GP+ G L+G F

Sbjct 134 RPEGVVIYGLFSSMLAFVPALGPIAGALIGEF 165

> floR_-_5741028_translation floR: florfenicol exporter

Length=404

Score = 40.0 bits (92), Expect = 2e-04, Method: Compositional matrix adjust.

Identities = 45/152 (30%), Positives = 70/152 (46%), Gaps = 14/152 (9%)

Frame = +1

Query 1261 ILSTVALDAVGIGL-IMPVLPGLLRDLVHSNDVTAHYGILLALYALMQFACAPVLGALSD 1437

IL+++A+D I L ++P +PG+L N A + L+LY +M + G LSD

Sbjct 24 ILASLAMD---IYLPVVPAMPGIL------NTTPAMIQLTLSLYMVMLGVGQVIFGPLSD 74

Query 1438 RFGRRPVLL---VSLAGAAVDYAIMATAPFLWVLYigrivagitgatgavagayiaDITD 1608

R GRRP+LL + A++ A +TAP +V + G + A +

Sbjct 75 RIGRRPILLAGATAFVIASLGAAWSSTAP-AFVAFRLLQAVGASAMLVATFATVRDVYAN 133

Query 1609 GDERARHFGFMSACFGFGMVAGPVLGGLMGGF 1704

E +G S+ F GP+ G L+G F

Sbjct 134 RPEGVVIYGLFSSMLAFVPALGPIAGALIGEF 165

> YP_008725150 Florfenicol/Chloramphenicol efflux protein (plasmid)

[Klebsiella pneumoniae]

Length=404

Score = 40.0 bits (92), Expect = 2e-04, Method: Compositional matrix adjust.

Identities = 45/152 (30%), Positives = 70/152 (46%), Gaps = 14/152 (9%)

Frame = +1

Query 1261 ILSTVALDAVGIGL-IMPVLPGLLRDLVHSNDVTAHYGILLALYALMQFACAPVLGALSD 1437

IL+++A+D I L ++P +PG+L N A + L+LY +M + G LSD

Sbjct 24 ILASLAMD---IYLPVVPAMPGIL------NTTPAMIQLTLSLYMVMLGVGQVIFGPLSD 74

Query 1438 RFGRRPVLL---VSLAGAAVDYAIMATAPFLWVLYigrivagitgatgavagayiaDITD 1608

R GRRP+LL + A++ A +TAP +V + G + A +

Sbjct 75 RIGRRPILLAGATAFVIASLGAAWSSTAP-AFVAFRLLQAVGASAMLVATFATVRDVYAN 133

Query 1609 GDERARHFGFMSACFGFGMVAGPVLGGLMGGF 1704

E +G S+ F GP+ G L+G F

Sbjct 134 RPEGVVIYGLFSSMLAFVPALGPIAGALIGEF 165

> YP_008167024 FloR (plasmid) [Klebsiella pneumoniae]

Length=404

Score = 40.0 bits (92), Expect = 2e-04, Method: Compositional matrix adjust.

Identities = 45/152 (30%), Positives = 70/152 (46%), Gaps = 14/152 (9%)

Frame = +1

Query 1261 ILSTVALDAVGIGL-IMPVLPGLLRDLVHSNDVTAHYGILLALYALMQFACAPVLGALSD 1437

IL+++A+D I L ++P +PG+L N A + L+LY +M + G LSD

Sbjct 24 ILASLAMD---IYLPVVPAMPGIL------NTTPAMIQLTLSLYMVMLGVGQVIFGPLSD 74

Query 1438 RFGRRPVLL---VSLAGAAVDYAIMATAPFLWVLYigrivagitgatgavagayiaDITD 1608

R GRRP+LL + A++ A +TAP +V + G + A +

Sbjct 75 RIGRRPILLAGATAFVIASLGAAWSSTAP-AFVAFRLLQAVGASAMLVATFATVRDVYAN 133

Query 1609 GDERARHFGFMSACFGFGMVAGPVLGGLMGGF 1704

E +G S+ F GP+ G L+G F

Sbjct 134 RPEGVVIYGLFSSMLAFVPALGPIAGALIGEF 165

> YP_006960714 FloR (plasmid) [uncultured bacterium HHV216]

Length=404

Score = 40.0 bits (92), Expect = 2e-04, Method: Compositional matrix adjust.

Identities = 45/152 (30%), Positives = 70/152 (46%), Gaps = 14/152 (9%)

Frame = +1

Query 1261 ILSTVALDAVGIGL-IMPVLPGLLRDLVHSNDVTAHYGILLALYALMQFACAPVLGALSD 1437

IL+++A+D I L ++P +PG+L N A + L+LY +M + G LSD

Sbjct 24 ILASLAMD---IYLPVVPAMPGIL------NTTPAMIQLTLSLYMVMLGVGQVIFGPLSD 74

Query 1438 RFGRRPVLL---VSLAGAAVDYAIMATAPFLWVLYigrivagitgatgavagayiaDITD 1608

R GRRP+LL + A++ A +TAP +V + G + A +

Sbjct 75 RIGRRPILLAGATAFVIASLGAAWSSTAP-AFVAFRLLQAVGASAMLVATFATVRDVYAN 133

Query 1609 GDERARHFGFMSACFGFGMVAGPVLGGLMGGF 1704

E +G S+ F GP+ G L+G F

Sbjct 134 RPEGVVIYGLFSSMLAFVPALGPIAGALIGEF 165

> YP_006957001 FloR, chloramphenicol/ florfenicol resistance (plasmid)

[Salmonella enterica subsp. enterica serovar Heidelberg]

Length=404

Score = 40.0 bits (92), Expect = 2e-04, Method: Compositional matrix adjust.

Identities = 45/152 (30%), Positives = 70/152 (46%), Gaps = 14/152 (9%)

Frame = +1

Query 1261 ILSTVALDAVGIGL-IMPVLPGLLRDLVHSNDVTAHYGILLALYALMQFACAPVLGALSD 1437

IL+++A+D I L ++P +PG+L N A + L+LY +M + G LSD

Sbjct 24 ILASLAMD---IYLPVVPAMPGIL------NTTPAMIQLTLSLYMVMLGVGQVIFGPLSD 74

Query 1438 RFGRRPVLL---VSLAGAAVDYAIMATAPFLWVLYigrivagitgatgavagayiaDITD 1608

R GRRP+LL + A++ A +TAP +V + G + A +

Sbjct 75 RIGRRPILLAGATAFVIASLGAAWSSTAP-AFVAFRLLQAVGASAMLVATFATVRDVYAN 133

Query 1609 GDERARHFGFMSACFGFGMVAGPVLGGLMGGF 1704

E +G S+ F GP+ G L+G F

Sbjct 134 RPEGVVIYGLFSSMLAFVPALGPIAGALIGEF 165

> YP_006956622 florfenicol/ chloramphenicol resistance protein

(plasmid) [Salmonella enterica subsp. enterica serovar Heidelberg]

Length=404

Score = 40.0 bits (92), Expect = 2e-04, Method: Compositional matrix adjust.

Identities = 45/152 (30%), Positives = 70/152 (46%), Gaps = 14/152 (9%)

Frame = +1

Query 1261 ILSTVALDAVGIGL-IMPVLPGLLRDLVHSNDVTAHYGILLALYALMQFACAPVLGALSD 1437

IL+++A+D I L ++P +PG+L N A + L+LY +M + G LSD

Sbjct 24 ILASLAMD---IYLPVVPAMPGIL------NTTPAMIQLTLSLYMVMLGVGQVIFGPLSD 74

Query 1438 RFGRRPVLL---VSLAGAAVDYAIMATAPFLWVLYigrivagitgatgavagayiaDITD 1608

R GRRP+LL + A++ A +TAP +V + G + A +

Sbjct 75 RIGRRPILLAGATAFVIASLGAAWSSTAP-AFVAFRLLQAVGASAMLVATFATVRDVYAN 133

Query 1609 GDERARHFGFMSACFGFGMVAGPVLGGLMGGF 1704

E +G S+ F GP+ G L+G F

Sbjct 134 RPEGVVIYGLFSSMLAFVPALGPIAGALIGEF 165

> YP_006956307 florfenicol/ chloramphenicol resistance protein

(plasmid) [Salmonella enterica subsp. enterica serovar Heidelberg]

Length=404

Score = 40.0 bits (92), Expect = 2e-04, Method: Compositional matrix adjust.

Identities = 45/152 (30%), Positives = 70/152 (46%), Gaps = 14/152 (9%)

Frame = +1

Query 1261 ILSTVALDAVGIGL-IMPVLPGLLRDLVHSNDVTAHYGILLALYALMQFACAPVLGALSD 1437

IL+++A+D I L ++P +PG+L N A + L+LY +M + G LSD

Sbjct 24 ILASLAMD---IYLPVVPAMPGIL------NTTPAMIQLTLSLYMVMLGVGQVIFGPLSD 74

Query 1438 RFGRRPVLL---VSLAGAAVDYAIMATAPFLWVLYigrivagitgatgavagayiaDITD 1608

R GRRP+LL + A++ A +TAP +V + G + A +

Sbjct 75 RIGRRPILLAGATAFVIASLGAAWSSTAP-AFVAFRLLQAVGASAMLVATFATVRDVYAN 133

Query 1609 GDERARHFGFMSACFGFGMVAGPVLGGLMGGF 1704

E +G S+ F GP+ G L+G F

Sbjct 134 RPEGVVIYGLFSSMLAFVPALGPIAGALIGEF 165

> YP_001552094 florfenicol exporter (plasmid) [Salmonella enterica

subsp. enterica serovar Dublin]

Length=404

Score = 40.0 bits (92), Expect = 2e-04, Method: Compositional matrix adjust.

Identities = 45/152 (30%), Positives = 70/152 (46%), Gaps = 14/152 (9%)

Frame = +1

Query 1261 ILSTVALDAVGIGL-IMPVLPGLLRDLVHSNDVTAHYGILLALYALMQFACAPVLGALSD 1437

IL+++A+D I L ++P +PG+L N A + L+LY +M + G LSD

Sbjct 24 ILASLAMD---IYLPVVPAMPGIL------NTTPAMIQLTLSLYMVMLGVGQVIFGPLSD 74

Query 1438 RFGRRPVLL---VSLAGAAVDYAIMATAPFLWVLYigrivagitgatgavagayiaDITD 1608

R GRRP+LL + A++ A +TAP +V + G + A +

Sbjct 75 RIGRRPILLAGATAFVIASLGAAWSSTAP-AFVAFRLLQAVGASAMLVATFATVRDVYAN 133

Query 1609 GDERARHFGFMSACFGFGMVAGPVLGGLMGGF 1704

E +G S+ F GP+ G L+G F

Sbjct 134 RPEGVVIYGLFSSMLAFVPALGPIAGALIGEF 165

> SAU17560 putative chloramphenicol and florfenicol resistance

protein (CmlA) [Klebsiella pneumoniae]

Length=404

Score = 40.0 bits (92), Expect = 2e-04, Method: Compositional matrix adjust.

Identities = 45/152 (30%), Positives = 70/152 (46%), Gaps = 14/152 (9%)

Frame = +1

Query 1261 ILSTVALDAVGIGL-IMPVLPGLLRDLVHSNDVTAHYGILLALYALMQFACAPVLGALSD 1437

IL+++A+D I L ++P +PG+L N A + L+LY +M + G LSD

Sbjct 24 ILASLAMD---IYLPVVPAMPGIL------NTTPAMIQLTLSLYMVMLGVGQVIFGPLSD 74

Query 1438 RFGRRPVLL---VSLAGAAVDYAIMATAPFLWVLYigrivagitgatgavagayiaDITD 1608

R GRRP+LL + A++ A +TAP +V + G + A +

Sbjct 75 RIGRRPILLAGATAFVIASLGAAWSSTAP-AFVAFRLLQAVGASAMLVATFATVRDVYAN 133

Query 1609 GDERARHFGFMSACFGFGMVAGPVLGGLMGGF 1704

E +G S+ F GP+ G L+G F

Sbjct 134 RPEGVVIYGLFSSMLAFVPALGPIAGALIGEF 165

> CAX63193 florfenicol exporter [Acinetobacter baumannii]

Length=404

Score = 40.0 bits (92), Expect = 2e-04, Method: Compositional matrix adjust.

Identities = 45/152 (30%), Positives = 70/152 (46%), Gaps = 14/152 (9%)

Frame = +1

Query 1261 ILSTVALDAVGIGL-IMPVLPGLLRDLVHSNDVTAHYGILLALYALMQFACAPVLGALSD 1437

IL+++A+D I L ++P +PG+L N A + L+LY +M + G LSD

Sbjct 24 ILASLAMD---IYLPVVPAMPGIL------NTTPAMIQLTLSLYMVMLGVGQVIFGPLSD 74

Query 1438 RFGRRPVLL---VSLAGAAVDYAIMATAPFLWVLYigrivagitgatgavagayiaDITD 1608

R GRRP+LL + A++ A +TAP +V + G + A +

Sbjct 75 RIGRRPILLAGATAFVIASLGAAWSSTAP-AFVAFRLLQAVGASAMLVATFATVRDVYAN 133

Query 1609 GDERARHFGFMSACFGFGMVAGPVLGGLMGGF 1704

E +G S+ F GP+ G L+G F

Sbjct 134 RPEGVVIYGLFSSMLAFVPALGPIAGALIGEF 165

> BAF93177 florfenicol exporter (plasmid) [Salmonella enterica

subsp. enterica serovar Dublin]

Length=404

Score = 40.0 bits (92), Expect = 2e-04, Method: Compositional matrix adjust.

Identities = 45/152 (30%), Positives = 70/152 (46%), Gaps = 14/152 (9%)

Frame = +1

Query 1261 ILSTVALDAVGIGL-IMPVLPGLLRDLVHSNDVTAHYGILLALYALMQFACAPVLGALSD 1437

IL+++A+D I L ++P +PG+L N A + L+LY +M + G LSD

Sbjct 24 ILASLAMD---IYLPVVPAMPGIL------NTTPAMIQLTLSLYMVMLGVGQVIFGPLSD 74

Query 1438 RFGRRPVLL---VSLAGAAVDYAIMATAPFLWVLYigrivagitgatgavagayiaDITD 1608

R GRRP+LL + A++ A +TAP +V + G + A +

Sbjct 75 RIGRRPILLAGATAFVIASLGAAWSSTAP-AFVAFRLLQAVGASAMLVATFATVRDVYAN 133

Query 1609 GDERARHFGFMSACFGFGMVAGPVLGGLMGGF 1704

E +G S+ F GP+ G L+G F

Sbjct 134 RPEGVVIYGLFSSMLAFVPALGPIAGALIGEF 165

> ASI37824 FloR (plasmid) [Escherichia coli]

Length=404

Score = 40.0 bits (92), Expect = 2e-04, Method: Compositional matrix adjust.

Identities = 45/152 (30%), Positives = 70/152 (46%), Gaps = 14/152 (9%)

Frame = +1

Query 1261 ILSTVALDAVGIGL-IMPVLPGLLRDLVHSNDVTAHYGILLALYALMQFACAPVLGALSD 1437

IL+++A+D I L ++P +PG+L N A + L+LY +M + G LSD

Sbjct 24 ILASLAMD---IYLPVVPAMPGIL------NTTPAMIQLTLSLYMVMLGVGQVIFGPLSD 74

Query 1438 RFGRRPVLL---VSLAGAAVDYAIMATAPFLWVLYigrivagitgatgavagayiaDITD 1608

R GRRP+LL + A++ A +TAP +V + G + A +

Sbjct 75 RIGRRPILLAGATAFVIASLGAAWSSTAP-AFVAFRLLQAVGASAMLVATFATVRDVYAN 133

Query 1609 GDERARHFGFMSACFGFGMVAGPVLGGLMGGF 1704

E +G S+ F GP+ G L+G F

Sbjct 134 RPEGVVIYGLFSSMLAFVPALGPIAGALIGEF 165

> ASF80635 FloR (plasmid) [Escherichia coli]

Length=404

Score = 40.0 bits (92), Expect = 2e-04, Method: Compositional matrix adjust.

Identities = 45/152 (30%), Positives = 70/152 (46%), Gaps = 14/152 (9%)

Frame = +1

Query 1261 ILSTVALDAVGIGL-IMPVLPGLLRDLVHSNDVTAHYGILLALYALMQFACAPVLGALSD 1437

IL+++A+D I L ++P +PG+L N A + L+LY +M + G LSD

Sbjct 24 ILASLAMD---IYLPVVPAMPGIL------NTTPAMIQLTLSLYMVMLGVGQVIFGPLSD 74

Query 1438 RFGRRPVLL---VSLAGAAVDYAIMATAPFLWVLYigrivagitgatgavagayiaDITD 1608

R GRRP+LL + A++ A +TAP +V + G + A +

Sbjct 75 RIGRRPILLAGATAFVIASLGAAWSSTAP-AFVAFRLLQAVGASAMLVATFATVRDVYAN 133

Query 1609 GDERARHFGFMSACFGFGMVAGPVLGGLMGGF 1704

E +G S+ F GP+ G L+G F

Sbjct 134 RPEGVVIYGLFSSMLAFVPALGPIAGALIGEF 165

> ASF80457 FloR (plasmid) [Klebsiella pneumoniae]

Length=404

Score = 40.0 bits (92), Expect = 2e-04, Method: Compositional matrix adjust.

Identities = 45/152 (30%), Positives = 70/152 (46%), Gaps = 14/152 (9%)

Frame = +1

Query 1261 ILSTVALDAVGIGL-IMPVLPGLLRDLVHSNDVTAHYGILLALYALMQFACAPVLGALSD 1437

IL+++A+D I L ++P +PG+L N A + L+LY +M + G LSD

Sbjct 24 ILASLAMD---IYLPVVPAMPGIL------NTTPAMIQLTLSLYMVMLGVGQVIFGPLSD 74

Query 1438 RFGRRPVLL---VSLAGAAVDYAIMATAPFLWVLYigrivagitgatgavagayiaDITD 1608

R GRRP+LL + A++ A +TAP +V + G + A +

Sbjct 75 RIGRRPILLAGATAFVIASLGAAWSSTAP-AFVAFRLLQAVGASAMLVATFATVRDVYAN 133

Query 1609 GDERARHFGFMSACFGFGMVAGPVLGGLMGGF 1704

E +G S+ F GP+ G L+G F

Sbjct 134 RPEGVVIYGLFSSMLAFVPALGPIAGALIGEF 165

> ASF80271 FloR (plasmid) [Citrobacter freundii]

Length=404

Score = 40.0 bits (92), Expect = 2e-04, Method: Compositional matrix adjust.

Identities = 45/152 (30%), Positives = 70/152 (46%), Gaps = 14/152 (9%)

Frame = +1

Query 1261 ILSTVALDAVGIGL-IMPVLPGLLRDLVHSNDVTAHYGILLALYALMQFACAPVLGALSD 1437

IL+++A+D I L ++P +PG+L N A + L+LY +M + G LSD

Sbjct 24 ILASLAMD---IYLPVVPAMPGIL------NTTPAMIQLTLSLYMVMLGVGQVIFGPLSD 74

Query 1438 RFGRRPVLL---VSLAGAAVDYAIMATAPFLWVLYigrivagitgatgavagayiaDITD 1608

R GRRP+LL + A++ A +TAP +V + G + A +

Sbjct 75 RIGRRPILLAGATAFVIASLGAAWSSTAP-AFVAFRLLQAVGASAMLVATFATVRDVYAN 133

Query 1609 GDERARHFGFMSACFGFGMVAGPVLGGLMGGF 1704

E +G S+ F GP+ G L+G F

Sbjct 134 RPEGVVIYGLFSSMLAFVPALGPIAGALIGEF 165

> ASF80034 FloR (plasmid) [Citrobacter freundii]

Length=404

Score = 40.0 bits (92), Expect = 2e-04, Method: Compositional matrix adjust.

Identities = 45/152 (30%), Positives = 70/152 (46%), Gaps = 14/152 (9%)

Frame = +1

Query 1261 ILSTVALDAVGIGL-IMPVLPGLLRDLVHSNDVTAHYGILLALYALMQFACAPVLGALSD 1437

IL+++A+D I L ++P +PG+L N A + L+LY +M + G LSD

Sbjct 24 ILASLAMD---IYLPVVPAMPGIL------NTTPAMIQLTLSLYMVMLGVGQVIFGPLSD 74

Query 1438 RFGRRPVLL---VSLAGAAVDYAIMATAPFLWVLYigrivagitgatgavagayiaDITD 1608

R GRRP+LL + A++ A +TAP +V + G + A +

Sbjct 75 RIGRRPILLAGATAFVIASLGAAWSSTAP-AFVAFRLLQAVGASAMLVATFATVRDVYAN 133

Query 1609 GDERARHFGFMSACFGFGMVAGPVLGGLMGGF 1704

E +G S+ F GP+ G L+G F

Sbjct 134 RPEGVVIYGLFSSMLAFVPALGPIAGALIGEF 165

> ASF79798 FloR (plasmid) [Escherichia coli]

Length=404

Score = 40.0 bits (92), Expect = 2e-04, Method: Compositional matrix adjust.

Identities = 45/152 (30%), Positives = 70/152 (46%), Gaps = 14/152 (9%)

Frame = +1

Query 1261 ILSTVALDAVGIGL-IMPVLPGLLRDLVHSNDVTAHYGILLALYALMQFACAPVLGALSD 1437

IL+++A+D I L ++P +PG+L N A + L+LY +M + G LSD

Sbjct 24 ILASLAMD---IYLPVVPAMPGIL------NTTPAMIQLTLSLYMVMLGVGQVIFGPLSD 74

Query 1438 RFGRRPVLL---VSLAGAAVDYAIMATAPFLWVLYigrivagitgatgavagayiaDITD 1608

R GRRP+LL + A++ A +TAP +V + G + A +

Sbjct 75 RIGRRPILLAGATAFVIASLGAAWSSTAP-AFVAFRLLQAVGASAMLVATFATVRDVYAN 133

Query 1609 GDERARHFGFMSACFGFGMVAGPVLGGLMGGF 1704

E +G S+ F GP+ G L+G F

Sbjct 134 RPEGVVIYGLFSSMLAFVPALGPIAGALIGEF 165

> AMD83221 FloR (plasmid) [Klebsiella pneumoniae]

Length=404

Score = 40.0 bits (92), Expect = 2e-04, Method: Compositional matrix adjust.

Identities = 45/152 (30%), Positives = 70/152 (46%), Gaps = 14/152 (9%)

Frame = +1

Query 1261 ILSTVALDAVGIGL-IMPVLPGLLRDLVHSNDVTAHYGILLALYALMQFACAPVLGALSD 1437

IL+++A+D I L ++P +PG+L N A + L+LY +M + G LSD

Sbjct 24 ILASLAMD---IYLPVVPAMPGIL------NTTPAMIQLTLSLYMVMLGVGQVIFGPLSD 74

Query 1438 RFGRRPVLL---VSLAGAAVDYAIMATAPFLWVLYigrivagitgatgavagayiaDITD 1608

R GRRP+LL + A++ A +TAP +V + G + A +

Sbjct 75 RIGRRPILLAGATAFVIASLGAAWSSTAP-AFVAFRLLQAVGASAMLVATFATVRDVYAN 133

Query 1609 GDERARHFGFMSACFGFGMVAGPVLGGLMGGF 1704

E +G S+ F GP+ G L+G F

Sbjct 134 RPEGVVIYGLFSSMLAFVPALGPIAGALIGEF 165

> AMD83053 FloR (plasmid) [Klebsiella pneumoniae]

Length=404

Score = 40.0 bits (92), Expect = 2e-04, Method: Compositional matrix adjust.

Identities = 45/152 (30%), Positives = 70/152 (46%), Gaps = 14/152 (9%)

Frame = +1

Query 1261 ILSTVALDAVGIGL-IMPVLPGLLRDLVHSNDVTAHYGILLALYALMQFACAPVLGALSD 1437

IL+++A+D I L ++P +PG+L N A + L+LY +M + G LSD

Sbjct 24 ILASLAMD---IYLPVVPAMPGIL------NTTPAMIQLTLSLYMVMLGVGQVIFGPLSD 74

Query 1438 RFGRRPVLL---VSLAGAAVDYAIMATAPFLWVLYigrivagitgatgavagayiaDITD 1608

R GRRP+LL + A++ A +TAP +V + G + A +

Sbjct 75 RIGRRPILLAGATAFVIASLGAAWSSTAP-AFVAFRLLQAVGASAMLVATFATVRDVYAN 133

Query 1609 GDERARHFGFMSACFGFGMVAGPVLGGLMGGF 1704

E +G S+ F GP+ G L+G F

Sbjct 134 RPEGVVIYGLFSSMLAFVPALGPIAGALIGEF 165

> AKN19379 FloR (plasmid) [Salmonella enterica subsp. enterica

serovar Corvallis]

Length=404

Score = 40.0 bits (92), Expect = 2e-04, Method: Compositional matrix adjust.

Identities = 45/152 (30%), Positives = 70/152 (46%), Gaps = 14/152 (9%)

Frame = +1

Query 1261 ILSTVALDAVGIGL-IMPVLPGLLRDLVHSNDVTAHYGILLALYALMQFACAPVLGALSD 1437

IL+++A+D I L ++P +PG+L N A + L+LY +M + G LSD

Sbjct 24 ILASLAMD---IYLPVVPAMPGIL------NTTPAMIQLTLSLYMVMLGVGQVIFGPLSD 74

Query 1438 RFGRRPVLL---VSLAGAAVDYAIMATAPFLWVLYigrivagitgatgavagayiaDITD 1608

R GRRP+LL + A++ A +TAP +V + G + A +

Sbjct 75 RIGRRPILLAGATAFVIASLGAAWSSTAP-AFVAFRLLQAVGASAMLVATFATVRDVYAN 133

Query 1609 GDERARHFGFMSACFGFGMVAGPVLGGLMGGF 1704

E +G S+ F GP+ G L+G F

Sbjct 134 RPEGVVIYGLFSSMLAFVPALGPIAGALIGEF 165

> AIP92379 florfenicol exporter [Vibrio fluvialis]

Length=404

Score = 40.0 bits (92), Expect = 2e-04, Method: Compositional matrix adjust.

Identities = 45/152 (30%), Positives = 70/152 (46%), Gaps = 14/152 (9%)

Frame = +1

Query 1261 ILSTVALDAVGIGL-IMPVLPGLLRDLVHSNDVTAHYGILLALYALMQFACAPVLGALSD 1437

IL+++A+D I L ++P +PG+L N A + L+LY +M + G LSD

Sbjct 24 ILASLAMD---IYLPVVPAMPGIL------NTTPAMIQLTLSLYMVMLGVGQVIFGPLSD 74

Query 1438 RFGRRPVLL---VSLAGAAVDYAIMATAPFLWVLYigrivagitgatgavagayiaDITD 1608

R GRRP+LL + A++ A +TAP +V + G + A +

Sbjct 75 RIGRRPILLAGATAFVIASLGAAWSSTAP-AFVAFRLLQAVGASAMLVATFATVRDVYAN 133

Query 1609 GDERARHFGFMSACFGFGMVAGPVLGGLMGGF 1704

E +G S+ F GP+ G L+G F

Sbjct 134 RPEGVVIYGLFSSMLAFVPALGPIAGALIGEF 165

> AGZ05482 Florfenicol/Chloramphenicol efflux protein (plasmid)

[Klebsiella pneumoniae]

Length=404

Score = 40.0 bits (92), Expect = 2e-04, Method: Compositional matrix adjust.

Identities = 45/152 (30%), Positives = 70/152 (46%), Gaps = 14/152 (9%)

Frame = +1

Query 1261 ILSTVALDAVGIGL-IMPVLPGLLRDLVHSNDVTAHYGILLALYALMQFACAPVLGALSD 1437

IL+++A+D I L ++P +PG+L N A + L+LY +M + G LSD

Sbjct 24 ILASLAMD---IYLPVVPAMPGIL------NTTPAMIQLTLSLYMVMLGVGQVIFGPLSD 74

Query 1438 RFGRRPVLL---VSLAGAAVDYAIMATAPFLWVLYigrivagitgatgavagayiaDITD 1608

R GRRP+LL + A++ A +TAP +V + G + A +

Sbjct 75 RIGRRPILLAGATAFVIASLGAAWSSTAP-AFVAFRLLQAVGASAMLVATFATVRDVYAN 133

Query 1609 GDERARHFGFMSACFGFGMVAGPVLGGLMGGF 1704

E +G S+ F GP+ G L+G F

Sbjct 134 RPEGVVIYGLFSSMLAFVPALGPIAGALIGEF 165

> AGO62602 FloR (plasmid) [Klebsiella pneumoniae]

Length=404

Score = 40.0 bits (92), Expect = 2e-04, Method: Compositional matrix adjust.

Identities = 45/152 (30%), Positives = 70/152 (46%), Gaps = 14/152 (9%)

Frame = +1

Query 1261 ILSTVALDAVGIGL-IMPVLPGLLRDLVHSNDVTAHYGILLALYALMQFACAPVLGALSD 1437

IL+++A+D I L ++P +PG+L N A + L+LY +M + G LSD

Sbjct 24 ILASLAMD---IYLPVVPAMPGIL------NTTPAMIQLTLSLYMVMLGVGQVIFGPLSD 74

Query 1438 RFGRRPVLL---VSLAGAAVDYAIMATAPFLWVLYigrivagitgatgavagayiaDITD 1608

R GRRP+LL + A++ A +TAP +V + G + A +

Sbjct 75 RIGRRPILLAGATAFVIASLGAAWSSTAP-AFVAFRLLQAVGASAMLVATFATVRDVYAN 133

Query 1609 GDERARHFGFMSACFGFGMVAGPVLGGLMGGF 1704

E +G S+ F GP+ G L+G F

Sbjct 134 RPEGVVIYGLFSSMLAFVPALGPIAGALIGEF 165

> AFG21521 florfenicol/ chloramphenicol resistance protein (plasmid)

[Salmonella enterica subsp. enterica serovar Heidelberg]

Length=404

Score = 40.0 bits (92), Expect = 2e-04, Method: Compositional matrix adjust.

Identities = 45/152 (30%), Positives = 70/152 (46%), Gaps = 14/152 (9%)

Frame = +1

Query 1261 ILSTVALDAVGIGL-IMPVLPGLLRDLVHSNDVTAHYGILLALYALMQFACAPVLGALSD 1437

IL+++A+D I L ++P +PG+L N A + L+LY +M + G LSD

Sbjct 24 ILASLAMD---IYLPVVPAMPGIL------NTTPAMIQLTLSLYMVMLGVGQVIFGPLSD 74

Query 1438 RFGRRPVLL---VSLAGAAVDYAIMATAPFLWVLYigrivagitgatgavagayiaDITD 1608

R GRRP+LL + A++ A +TAP +V + G + A +

Sbjct 75 RIGRRPILLAGATAFVIASLGAAWSSTAP-AFVAFRLLQAVGASAMLVATFATVRDVYAN 133

Query 1609 GDERARHFGFMSACFGFGMVAGPVLGGLMGGF 1704

E +G S+ F GP+ G L+G F

Sbjct 134 RPEGVVIYGLFSSMLAFVPALGPIAGALIGEF 165

> AFG21042 florfenicol/ chloramphenicol resistance protein (plasmid)

[Salmonella enterica subsp. enterica serovar Heidelberg]

Length=404

Score = 40.0 bits (92), Expect = 2e-04, Method: Compositional matrix adjust.

Identities = 45/152 (30%), Positives = 70/152 (46%), Gaps = 14/152 (9%)

Frame = +1

Query 1261 ILSTVALDAVGIGL-IMPVLPGLLRDLVHSNDVTAHYGILLALYALMQFACAPVLGALSD 1437

IL+++A+D I L ++P +PG+L N A + L+LY +M + G LSD

Sbjct 24 ILASLAMD---IYLPVVPAMPGIL------NTTPAMIQLTLSLYMVMLGVGQVIFGPLSD 74

Query 1438 RFGRRPVLL---VSLAGAAVDYAIMATAPFLWVLYigrivagitgatgavagayiaDITD 1608

R GRRP+LL + A++ A +TAP +V + G + A +

Sbjct 75 RIGRRPILLAGATAFVIASLGAAWSSTAP-AFVAFRLLQAVGASAMLVATFATVRDVYAN 133

Query 1609 GDERARHFGFMSACFGFGMVAGPVLGGLMGGF 1704

E +G S+ F GP+ G L+G F

Sbjct 134 RPEGVVIYGLFSSMLAFVPALGPIAGALIGEF 165

> AFG20876 FloR, chloramphenicol/ florfenicol resistance (plasmid)

[Salmonella enterica subsp. enterica serovar Heidelberg]

Length=404

Score = 40.0 bits (92), Expect = 2e-04, Method: Compositional matrix adjust.

Identities = 45/152 (30%), Positives = 70/152 (46%), Gaps = 14/152 (9%)

Frame = +1

Query 1261 ILSTVALDAVGIGL-IMPVLPGLLRDLVHSNDVTAHYGILLALYALMQFACAPVLGALSD 1437

IL+++A+D I L ++P +PG+L N A + L+LY +M + G LSD

Sbjct 24 ILASLAMD---IYLPVVPAMPGIL------NTTPAMIQLTLSLYMVMLGVGQVIFGPLSD 74

Query 1438 RFGRRPVLL---VSLAGAAVDYAIMATAPFLWVLYigrivagitgatgavagayiaDITD 1608

R GRRP+LL + A++ A +TAP +V + G + A +

Sbjct 75 RIGRRPILLAGATAFVIASLGAAWSSTAP-AFVAFRLLQAVGASAMLVATFATVRDVYAN 133

Query 1609 GDERARHFGFMSACFGFGMVAGPVLGGLMGGF 1704

E +G S+ F GP+ G L+G F

Sbjct 134 RPEGVVIYGLFSSMLAFVPALGPIAGALIGEF 165

> AEX15914 florfenicol/chloramphenicol resistance protein (plasmid)

[Acinetobacter baumannii]

Length=404

Score = 40.0 bits (92), Expect = 2e-04, Method: Compositional matrix adjust.

Identities = 45/152 (30%), Positives = 70/152 (46%), Gaps = 14/152 (9%)

Frame = +1

Query 1261 ILSTVALDAVGIGL-IMPVLPGLLRDLVHSNDVTAHYGILLALYALMQFACAPVLGALSD 1437

IL+++A+D I L ++P +PG+L N A + L+LY +M + G LSD

Sbjct 24 ILASLAMD---IYLPVVPAMPGIL------NTTPAMIQLTLSLYMVMLGVGQVIFGPLSD 74

Query 1438 RFGRRPVLL---VSLAGAAVDYAIMATAPFLWVLYigrivagitgatgavagayiaDITD 1608

R GRRP+LL + A++ A +TAP +V + G + A +

Sbjct 75 RIGRRPILLAGATAFVIASLGAAWSSTAP-AFVAFRLLQAVGASAMLVATFATVRDVYAN 133

Query 1609 GDERARHFGFMSACFGFGMVAGPVLGGLMGGF 1704

E +G S+ F GP+ G L+G F

Sbjct 134 RPEGVVIYGLFSSMLAFVPALGPIAGALIGEF 165

> ACI02892 FloR (plasmid) [uncultured bacterium HHV216]

Length=404

Score = 40.0 bits (92), Expect = 2e-04, Method: Compositional matrix adjust.

Identities = 45/152 (30%), Positives = 70/152 (46%), Gaps = 14/152 (9%)

Frame = +1

Query 1261 ILSTVALDAVGIGL-IMPVLPGLLRDLVHSNDVTAHYGILLALYALMQFACAPVLGALSD 1437

IL+++A+D I L ++P +PG+L N A + L+LY +M + G LSD

Sbjct 24 ILASLAMD---IYLPVVPAMPGIL------NTTPAMIQLTLSLYMVMLGVGQVIFGPLSD 74

Query 1438 RFGRRPVLL---VSLAGAAVDYAIMATAPFLWVLYigrivagitgatgavagayiaDITD 1608

R GRRP+LL + A++ A +TAP +V + G + A +

Sbjct 75 RIGRRPILLAGATAFVIASLGAAWSSTAP-AFVAFRLLQAVGASAMLVATFATVRDVYAN 133

Query 1609 GDERARHFGFMSACFGFGMVAGPVLGGLMGGF 1704

E +G S+ F GP+ G L+G F

Sbjct 134 RPEGVVIYGLFSSMLAFVPALGPIAGALIGEF 165

> ABA56513 florfenicol export protein, partial [Salmonella enterica

subsp. enterica serovar Typhimurium]

Length=404

Score = 40.0 bits (92), Expect = 3e-04, Method: Compositional matrix adjust.

Identities = 45/152 (30%), Positives = 70/152 (46%), Gaps = 14/152 (9%)

Frame = +1

Query 1261 ILSTVALDAVGIGL-IMPVLPGLLRDLVHSNDVTAHYGILLALYALMQFACAPVLGALSD 1437

IL+++A+D I L ++P +PG+L N A + L+LY +M + G LSD

Sbjct 24 ILASLAMD---IYLPVVPAMPGIL------NTTPAMIQLTLSLYMVMLGVGQVIFGPLSD 74

Query 1438 RFGRRPVLL---VSLAGAAVDYAIMATAPFLWVLYigrivagitgatgavagayiaDITD 1608

R GRRP+LL + A++ A +TAP +V + G + A +

Sbjct 75 RIGRRPILLAGATAFVIASLGAAWSSTAP-AFVAFRLLQAVGASAMLVATFATVRDVYAN 133

Query 1609 GDERARHFGFMSACFGFGMVAGPVLGGLMGGF 1704

E +G S+ F GP+ G L+G F

Sbjct 134 RPEGVVIYGLFSSMLAFVPALGPIAGALIGEF 165

> CEL26452 FloR (plasmid) [Escherichia coli]

Length=404

Score = 40.0 bits (92), Expect = 3e-04, Method: Compositional matrix adjust.

Identities = 45/152 (30%), Positives = 70/152 (46%), Gaps = 14/152 (9%)

Frame = +1

Query 1261 ILSTVALDAVGIGL-IMPVLPGLLRDLVHSNDVTAHYGILLALYALMQFACAPVLGALSD 1437

IL+++A+D I L ++P +PG+L N A + L+LY +M + G LSD

Sbjct 24 ILASLAMD---IYLPVVPAMPGIL------NTTPAMIQLTLSLYMVMLGVGQVIFGPLSD 74

Query 1438 RFGRRPVLL---VSLAGAAVDYAIMATAPFLWVLYigrivagitgatgavagayiaDITD 1608

R GRRP+LL + A++ A +TAP +V + G + A +

Sbjct 75 RIGRRPILLAGATAFVIASLGAAWSSTAP-AFVAFRLLQAVGASAMLVATFATVRDVYAN 133

Query 1609 GDERARHFGFMSACFGFGMVAGPVLGGLMGGF 1704

E +G S+ F GP+ G L+G F

Sbjct 134 RPEGVVIYGLFSSMLAFVPALGPIAGALIGEF 165

> CEL26214 FloR (plasmid) [Escherichia coli]

Length=404

Score = 40.0 bits (92), Expect = 3e-04, Method: Compositional matrix adjust.

Identities = 45/152 (30%), Positives = 70/152 (46%), Gaps = 14/152 (9%)

Frame = +1

Query 1261 ILSTVALDAVGIGL-IMPVLPGLLRDLVHSNDVTAHYGILLALYALMQFACAPVLGALSD 1437

IL+++A+D I L ++P +PG+L N A + L+LY +M + G LSD

Sbjct 24 ILASLAMD---IYLPVVPAMPGIL------NTTPAMIQLTLSLYMVMLGVGQVIFGPLSD 74

Query 1438 RFGRRPVLL---VSLAGAAVDYAIMATAPFLWVLYigrivagitgatgavagayiaDITD 1608

R GRRP+LL + A++ A +TAP +V + G + A +

Sbjct 75 RIGRRPILLAGATAFVIASLGAAWSSTAP-AFVAFRLLQAVGASAMLVATFATVRDVYAN 133

Query 1609 GDERARHFGFMSACFGFGMVAGPVLGGLMGGF 1704

E +G S+ F GP+ G L+G F

Sbjct 134 RPEGVVIYGLFSSMLAFVPALGPIAGALIGEF 165

> CEL26092 FloR (plasmid) [Escherichia coli]

Length=404

Score = 40.0 bits (92), Expect = 3e-04, Method: Compositional matrix adjust.

Identities = 45/152 (30%), Positives = 70/152 (46%), Gaps = 14/152 (9%)

Frame = +1

Query 1261 ILSTVALDAVGIGL-IMPVLPGLLRDLVHSNDVTAHYGILLALYALMQFACAPVLGALSD 1437

IL+++A+D I L ++P +PG+L N A + L+LY +M + G LSD

Sbjct 24 ILASLAMD---IYLPVVPAMPGIL------NTTPAMIQLTLSLYMVMLGVGQVIFGPLSD 74

Query 1438 RFGRRPVLL---VSLAGAAVDYAIMATAPFLWVLYigrivagitgatgavagayiaDITD 1608

R GRRP+LL + A++ A +TAP +V + G + A +

Sbjct 75 RIGRRPILLAGATAFVIASLGAAWSSTAP-AFVAFRLLQAVGASAMLVATFATVRDVYAN 133

Query 1609 GDERARHFGFMSACFGFGMVAGPVLGGLMGGF 1704

E +G S+ F GP+ G L+G F

Sbjct 134 RPEGVVIYGLFSSMLAFVPALGPIAGALIGEF 165

> ALS39188 Florfenicol resistance protein (plasmid) [Escherichia

coli]

Length=404

Score = 40.0 bits (92), Expect = 3e-04, Method: Compositional matrix adjust.

Identities = 45/152 (30%), Positives = 70/152 (46%), Gaps = 14/152 (9%)

Frame = +1

Query 1261 ILSTVALDAVGIGL-IMPVLPGLLRDLVHSNDVTAHYGILLALYALMQFACAPVLGALSD 1437

IL+++A+D I L ++P +PG+L N A + L+LY +M + G LSD

Sbjct 24 ILASLAMD---IYLPVVPAMPGIL------NTTPAMIQLTLSLYMVMLGVGQVIFGPLSD 74

Query 1438 RFGRRPVLL---VSLAGAAVDYAIMATAPFLWVLYigrivagitgatgavagayiaDITD 1608

R GRRP+LL + A++ A +TAP +V + G + A +

Sbjct 75 RIGRRPILLAGATAFVIASLGAAWSSTAP-AFVAFRLLQAVGASAMLVATFATVRDVYAN 133

Query 1609 GDERARHFGFMSACFGFGMVAGPVLGGLMGGF 1704

E +G S+ F GP+ G L+G F

Sbjct 134 RPEGVVIYGLFSSMLAFVPALGPIAGALIGEF 165

> ACK58048 florfenicol resistance protein, partial [Vibrio tasmaniensis]

Length=279

Score = 39.7 bits (91), Expect = 3e-04, Method: Compositional matrix adjust.

Identities = 39/137 (28%), Positives = 60/137 (44%), Gaps = 10/137 (7%)

Frame = +1

Query 1303 IMPVLPGLLRDLVHSNDVTAHYGILLALYALMQFACAPVLGALSDRFGRRPVLL---VSL 1473

++P +PG+L N A + L+LY +M + G LSDR GRRP+LL +

Sbjct 5 VVPAMPGIL------NTTPAMIQLTLSLYMVMLGVGQVIFGPLSDRIGRRPILLAGATAF 58

Query 1474 AGAAVDYAIMATAPFLWVLYigrivagitgatgavagayiaDITDGDERARHFGFMSACF 1653

A++ A +TAP +V + G + A + E +G S+

Sbjct 59 VIASLGAAWSSTAP-AFVAFRLLQAVGASAMLVATFATVRDVYANRPEGVVIYGLFSSML 117

Query 1654 GFGMVAGPVLGGLMGGF 1704

F GP+ G L+G F

Sbjct 118 AFVPALGPIAGALIGEF 134

> CAB64207 chloramphenicol/florfenicol exporter [Salmonella enterica

subsp. enterica serovar Typhimurium]

Length=404

Score = 40.0 bits (92), Expect = 3e-04, Method: Compositional matrix adjust.

Identities = 45/152 (30%), Positives = 70/152 (46%), Gaps = 14/152 (9%)

Frame = +1

Query 1261 ILSTVALDAVGIGL-IMPVLPGLLRDLVHSNDVTAHYGILLALYALMQFACAPVLGALSD 1437

IL+++A+D I L ++P +PG+L N A + L+LY +M + G LSD

Sbjct 24 ILASLAMD---IYLPVVPAMPGIL------NTTPAMIQLTLSLYMVMLGVGQVIFGPLSD 74

Query 1438 RFGRRPVLL---VSLAGAAVDYAIMATAPFLWVLYigrivagitgatgavagayiaDITD 1608

R GRRP+LL + A++ A +TAP +V + G + A +

Sbjct 75 RIGRRPILLAGATAFVIASLGAAWSSTAP-AFVAFRLLQAVGASAMLVATFATVRDVYAN 133

Query 1609 GDERARHFGFMSACFGFGMVAGPVLGGLMGGF 1704

E +G S+ F GP+ G L+G F

Sbjct 134 RPEGVVIYGLFSSMLAFVPALGPIAGALIGEF 165

> ABP96837 florfenicol resistance protein, partial [Vibrio splendidus]

Length=283

Score = 39.7 bits (91), Expect = 3e-04, Method: Compositional matrix adjust.

Identities = 39/137 (28%), Positives = 60/137 (44%), Gaps = 10/137 (7%)

Frame = +1

Query 1303 IMPVLPGLLRDLVHSNDVTAHYGILLALYALMQFACAPVLGALSDRFGRRPVLL---VSL 1473

++P +PG+L N A + L+LY +M + G LSDR GRRP+LL +

Sbjct 7 VVPAMPGIL------NTTPAMIQLTLSLYMVMLGVGQVIFGPLSDRIGRRPILLAGATAF 60

Query 1474 AGAAVDYAIMATAPFLWVLYigrivagitgatgavagayiaDITDGDERARHFGFMSACF 1653

A++ A +TAP +V + G + A + E +G S+

Sbjct 61 VIASLGAAWSSTAP-AFVAFRLLQAVGASAMLVATFATVRDVYANRPEGVVIYGLFSSML 119

Query 1654 GFGMVAGPVLGGLMGGF 1704

F GP+ G L+G F

Sbjct 120 AFVPALGPIAGALIGEF 136

> floR_-_7872488_translation floR: FloR

Length=404

Score = 40.0 bits (92), Expect = 3e-04, Method: Compositional matrix adjust.

Identities = 45/152 (30%), Positives = 70/152 (46%), Gaps = 14/152 (9%)

Frame = +1

Query 1261 ILSTVALDAVGIGL-IMPVLPGLLRDLVHSNDVTAHYGILLALYALMQFACAPVLGALSD 1437

IL+++A+D I L ++P +PG+L N A + L+LY +M + G LSD

Sbjct 24 ILASLAMD---IYLPVVPAMPGIL------NTTPAMIQLTLSLYMVMLGVGQVIFGPLSD 74

Query 1438 RFGRRPVLL---VSLAGAAVDYAIMATAPFLWVLYigrivagitgatgavagayiaDITD 1608

R GRRP+LL + A++ A +TAP +V + G + A +

Sbjct 75 RIGRRPILLAGATAFVIASLGAAWSSTAP-AFVAFRLLQAVGASAMLVATFATVRDVYAN 133

Query 1609 GDERARHFGFMSACFGFGMVAGPVLGGLMGGF 1704

E +G S+ F GP+ G L+G F

Sbjct 134 RPEGVVIYGLFSSMLAFVPALGPIAGALIGEF 165

> YP_002891083 FloR (plasmid) [Escherichia coli]

Length=404

Score = 40.0 bits (92), Expect = 3e-04, Method: Compositional matrix adjust.

Identities = 45/152 (30%), Positives = 70/152 (46%), Gaps = 14/152 (9%)

Frame = +1

Query 1261 ILSTVALDAVGIGL-IMPVLPGLLRDLVHSNDVTAHYGILLALYALMQFACAPVLGALSD 1437

IL+++A+D I L ++P +PG+L N A + L+LY +M + G LSD

Sbjct 24 ILASLAMD---IYLPVVPAMPGIL------NTTPAMIQLTLSLYMVMLGVGQVIFGPLSD 74

Query 1438 RFGRRPVLL---VSLAGAAVDYAIMATAPFLWVLYigrivagitgatgavagayiaDITD 1608

R GRRP+LL + A++ A +TAP +V + G + A +

Sbjct 75 RIGRRPILLAGATAFVIASLGAAWSSTAP-AFVAFRLLQAVGASAMLVATFATVRDVYAN 133

Query 1609 GDERARHFGFMSACFGFGMVAGPVLGGLMGGF 1704

E +G S+ F GP+ G L+G F

Sbjct 134 RPEGVVIYGLFSSMLAFVPALGPIAGALIGEF 165

> ACQ77650 FloR (plasmid) [Escherichia coli]

Length=404

Score = 40.0 bits (92), Expect = 3e-04, Method: Compositional matrix adjust.

Identities = 45/152 (30%), Positives = 70/152 (46%), Gaps = 14/152 (9%)

Frame = +1

Query 1261 ILSTVALDAVGIGL-IMPVLPGLLRDLVHSNDVTAHYGILLALYALMQFACAPVLGALSD 1437

IL+++A+D I L ++P +PG+L N A + L+LY +M + G LSD

Sbjct 24 ILASLAMD---IYLPVVPAMPGIL------NTTPAMIQLTLSLYMVMLGVGQVIFGPLSD 74

Query 1438 RFGRRPVLL---VSLAGAAVDYAIMATAPFLWVLYigrivagitgatgavagayiaDITD 1608

R GRRP+LL + A++ A +TAP +V + G + A +

Sbjct 75 RIGRRPILLAGATAFVIASLGAAWSSTAP-AFVAFRLLQAVGASAMLVATFATVRDVYAN 133

Query 1609 GDERARHFGFMSACFGFGMVAGPVLGGLMGGF 1704

E +G S+ F GP+ G L+G F

Sbjct 134 RPEGVVIYGLFSSMLAFVPALGPIAGALIGEF 165

> WP_063845119 chloramphenicol/florfenicol efflux MFS transporter

FloR [Bordetella bronchiseptica]

Length=404

Score = 40.0 bits (92), Expect = 3e-04, Method: Compositional matrix adjust.

Identities = 45/152 (30%), Positives = 70/152 (46%), Gaps = 14/152 (9%)

Frame = +1

Query 1261 ILSTVALDAVGIGL-IMPVLPGLLRDLVHSNDVTAHYGILLALYALMQFACAPVLGALSD 1437

IL+++A+D I L ++P +PG+L N A + L+LY +M + G LSD

Sbjct 24 ILASLAMD---IYLPVVPAMPGIL------NTTPAMIQLTLSLYMVMLGVGQVIFGPLSD 74

Query 1438 RFGRRPVLL---VSLAGAAVDYAIMATAPFLWVLYigrivagitgatgavagayiaDITD 1608

R GRRP+LL + A++ A +TAP +V + G + A +

Sbjct 75 RIGRRPILLAGATAFVIASLGAAWSSTAP-AFVAFRLLQAVGASAMLVATFATVRDVYAN 133

Query 1609 GDERARHFGFMSACFGFGMVAGPVLGGLMGGF 1704

E +G S+ F GP+ G L+G F

Sbjct 134 RPEGVVIYGLFSSMLAFVPALGPIAGALIGEF 165

> CAL30184 Florfenicol/Chloramphenicol efflux protein [Bordetella

bronchiseptica]

Length=404

Score = 40.0 bits (92), Expect = 3e-04, Method: Compositional matrix adjust.

Identities = 45/152 (30%), Positives = 70/152 (46%), Gaps = 14/152 (9%)

Frame = +1

Query 1261 ILSTVALDAVGIGL-IMPVLPGLLRDLVHSNDVTAHYGILLALYALMQFACAPVLGALSD 1437

IL+++A+D I L ++P +PG+L N A + L+LY +M + G LSD

Sbjct 24 ILASLAMD---IYLPVVPAMPGIL------NTTPAMIQLTLSLYMVMLGVGQVIFGPLSD 74

Query 1438 RFGRRPVLL---VSLAGAAVDYAIMATAPFLWVLYigrivagitgatgavagayiaDITD 1608

R GRRP+LL + A++ A +TAP +V + G + A +

Sbjct 75 RIGRRPILLAGATAFVIASLGAAWSSTAP-AFVAFRLLQAVGASAMLVATFATVRDVYAN 133

Query 1609 GDERARHFGFMSACFGFGMVAGPVLGGLMGGF 1704

E +G S+ F GP+ G L+G F

Sbjct 134 RPEGVVIYGLFSSMLAFVPALGPIAGALIGEF 165

> AAC83804 chloramphenicol and florfenicol resistance protein,

partial [Salmonella enterica subsp. enterica serovar Typhimurium]

Length=399

Score = 40.0 bits (92), Expect = 3e-04, Method: Compositional matrix adjust.

Identities = 45/152 (30%), Positives = 70/152 (46%), Gaps = 14/152 (9%)

Frame = +1

Query 1261 ILSTVALDAVGIGL-IMPVLPGLLRDLVHSNDVTAHYGILLALYALMQFACAPVLGALSD 1437

IL+++A+D I L ++P +PG+L N A + L+LY +M + G LSD

Sbjct 24 ILASLAMD---IYLPVVPAMPGIL------NTTPAMIQLTLSLYMVMLGVGQVIFGPLSD 74

Query 1438 RFGRRPVLL---VSLAGAAVDYAIMATAPFLWVLYigrivagitgatgavagayiaDITD 1608

R GRRP+LL + A++ A +TAP +V + G + A +

Sbjct 75 RIGRRPILLAGATAFVIASLGAAWSSTAP-AFVAFRLLQAVGASAMLVATFATVRDVYAN 133

Query 1609 GDERARHFGFMSACFGFGMVAGPVLGGLMGGF 1704

E +G S+ F GP+ G L+G F

Sbjct 134 RPEGVVIYGLFSSMLAFVPALGPIAGALIGEF 165

> floR_-_18252523_translation floR: Florfenicol export protein

Length=404

Score = 40.0 bits (92), Expect = 3e-04, Method: Compositional matrix adjust.

Identities = 45/152 (30%), Positives = 70/152 (46%), Gaps = 14/152 (9%)

Frame = +1

Query 1261 ILSTVALDAVGIGL-IMPVLPGLLRDLVHSNDVTAHYGILLALYALMQFACAPVLGALSD 1437

IL+++A+D I L ++P +PG+L N A + L+LY +M + G LSD

Sbjct 24 ILASLAMD---IYLPVVPAMPGIL------NTTPAMIQLTLSLYMVMLGVGQVIFGPLSD 74

Query 1438 RFGRRPVLL---VSLAGAAVDYAIMATAPFLWVLYigrivagitgatgavagayiaDITD 1608

R GRRP+LL + A++ A +TAP +V + G + A +

Sbjct 75 RIGRRPILLAGATAFVIASLGAAWSSTAP-AFVAFRLLQAVGASAMLVATFATVRDVYAN 133

Query 1609 GDERARHFGFMSACFGFGMVAGPVLGGLMGGF 1704

E +G S+ F GP+ G L+G F

Sbjct 134 RPEGVVIYGLFSSMLAFVPALGPIAGALIGEF 165

> floR_-_7872612_translation floR: FloR

Length=404

Score = 40.0 bits (92), Expect = 3e-04, Method: Compositional matrix adjust.

Identities = 45/152 (30%), Positives = 70/152 (46%), Gaps = 14/152 (9%)

Frame = +1

Query 1261 ILSTVALDAVGIGL-IMPVLPGLLRDLVHSNDVTAHYGILLALYALMQFACAPVLGALSD 1437

IL+++A+D I L ++P +PG+L N A + L+LY +M + G LSD

Sbjct 24 ILASLAMD---IYLPVVPAMPGIL------NTTPAMIQLTLSLYMVMLGVGQVIFGPLSD 74

Query 1438 RFGRRPVLL---VSLAGAAVDYAIMATAPFLWVLYigrivagitgatgavagayiaDITD 1608

R GRRP+LL + A++ A +TAP +V + G + A +

Sbjct 75 RIGRRPILLAGATAFVIASLGAAWSSTAP-AFVAFRLLQAVGASAMLVATFATVRDVYAN 133

Query 1609 GDERARHFGFMSACFGFGMVAGPVLGGLMGGF 1704

E +G S+ F GP+ G L+G F

Sbjct 134 RPEGVVIYGLFSSMLAFVPALGPIAGALIGEF 165

> YP_008998279 Florfenicol export protein (plasmid) [Escherichia

coli ACN001]

Length=404

Score = 40.0 bits (92), Expect = 3e-04, Method: Compositional matrix adjust.

Identities = 45/152 (30%), Positives = 70/152 (46%), Gaps = 14/152 (9%)

Frame = +1

Query 1261 ILSTVALDAVGIGL-IMPVLPGLLRDLVHSNDVTAHYGILLALYALMQFACAPVLGALSD 1437

IL+++A+D I L ++P +PG+L N A + L+LY +M + G LSD

Sbjct 24 ILASLAMD---IYLPVVPAMPGIL------NTTPAMIQLTLSLYMVMLGVGQVIFGPLSD 74

Query 1438 RFGRRPVLL---VSLAGAAVDYAIMATAPFLWVLYigrivagitgatgavagayiaDITD 1608

R GRRP+LL + A++ A +TAP +V + G + A +

Sbjct 75 RIGRRPILLAGATAFVIASLGAAWSSTAP-AFVAFRLLQAVGASAMLVATFATVRDVYAN 133

Query 1609 GDERARHFGFMSACFGFGMVAGPVLGGLMGGF 1704

E +G S+ F GP+ G L+G F

Sbjct 134 RPEGVVIYGLFSSMLAFVPALGPIAGALIGEF 165

> YP_002894387 FloR (plasmid) [Escherichia coli]

Length=404

Score = 40.0 bits (92), Expect = 3e-04, Method: Compositional matrix adjust.

Identities = 45/152 (30%), Positives = 70/152 (46%), Gaps = 14/152 (9%)

Frame = +1

Query 1261 ILSTVALDAVGIGL-IMPVLPGLLRDLVHSNDVTAHYGILLALYALMQFACAPVLGALSD 1437

IL+++A+D I L ++P +PG+L N A + L+LY +M + G LSD

Sbjct 24 ILASLAMD---IYLPVVPAMPGIL------NTTPAMIQLTLSLYMVMLGVGQVIFGPLSD 74

Query 1438 RFGRRPVLL---VSLAGAAVDYAIMATAPFLWVLYigrivagitgatgavagayiaDITD 1608

R GRRP+LL + A++ A +TAP +V + G + A +

Sbjct 75 RIGRRPILLAGATAFVIASLGAAWSSTAP-AFVAFRLLQAVGASAMLVATFATVRDVYAN 133

Query 1609 GDERARHFGFMSACFGFGMVAGPVLGGLMGGF 1704

E +G S+ F GP+ G L+G F

Sbjct 134 RPEGVVIYGLFSSMLAFVPALGPIAGALIGEF 165

> SBZ20918 putative chloramphenicol and florfenicol resistance

protein (CmlA) [Klebsiella pneumoniae]

Length=404

Score = 40.0 bits (92), Expect = 3e-04, Method: Compositional matrix adjust.

Identities = 45/152 (30%), Positives = 70/152 (46%), Gaps = 14/152 (9%)

Frame = +1

Query 1261 ILSTVALDAVGIGL-IMPVLPGLLRDLVHSNDVTAHYGILLALYALMQFACAPVLGALSD 1437

IL+++A+D I L ++P +PG+L N A + L+LY +M + G LSD

Sbjct 24 ILASLAMD---IYLPVVPAMPGIL------NTTPAMIQLTLSLYMVMLGVGQVIFGPLSD 74

Query 1438 RFGRRPVLL---VSLAGAAVDYAIMATAPFLWVLYigrivagitgatgavagayiaDITD 1608

R GRRP+LL + A++ A +TAP +V + G + A +

Sbjct 75 RIGRRPILLAGATAFVIASLGAAWSSTAP-AFVAFRLLQAVGASAMLVATFATVRDVYAN 133

Query 1609 GDERARHFGFMSACFGFGMVAGPVLGGLMGGF 1704

E +G S+ F GP+ G L+G F

Sbjct 134 RPEGVVIYGLFSSMLAFVPALGPIAGALIGEF 165

> AHF23060 Florfenicol export protein (plasmid) [Escherichia coli

ACN001]

Length=404

Score = 40.0 bits (92), Expect = 3e-04, Method: Compositional matrix adjust.

Identities = 45/152 (30%), Positives = 70/152 (46%), Gaps = 14/152 (9%)

Frame = +1

Query 1261 ILSTVALDAVGIGL-IMPVLPGLLRDLVHSNDVTAHYGILLALYALMQFACAPVLGALSD 1437

IL+++A+D I L ++P +PG+L N A + L+LY +M + G LSD

Sbjct 24 ILASLAMD---IYLPVVPAMPGIL------NTTPAMIQLTLSLYMVMLGVGQVIFGPLSD 74

Query 1438 RFGRRPVLL---VSLAGAAVDYAIMATAPFLWVLYigrivagitgatgavagayiaDITD 1608

R GRRP+LL + A++ A +TAP +V + G + A +

Sbjct 75 RIGRRPILLAGATAFVIASLGAAWSSTAP-AFVAFRLLQAVGASAMLVATFATVRDVYAN 133

Query 1609 GDERARHFGFMSACFGFGMVAGPVLGGLMGGF 1704

E +G S+ F GP+ G L+G F

Sbjct 134 RPEGVVIYGLFSSMLAFVPALGPIAGALIGEF 165

> ADE58508 FloR (plasmid) [Escherichia coli]

Length=404

Score = 40.0 bits (92), Expect = 3e-04, Method: Compositional matrix adjust.

Identities = 45/152 (30%), Positives = 70/152 (46%), Gaps = 14/152 (9%)

Frame = +1

Query 1261 ILSTVALDAVGIGL-IMPVLPGLLRDLVHSNDVTAHYGILLALYALMQFACAPVLGALSD 1437

IL+++A+D I L ++P +PG+L N A + L+LY +M + G LSD

Sbjct 24 ILASLAMD---IYLPVVPAMPGIL------NTTPAMIQLTLSLYMVMLGVGQVIFGPLSD 74

Query 1438 RFGRRPVLL---VSLAGAAVDYAIMATAPFLWVLYigrivagitgatgavagayiaDITD 1608

R GRRP+LL + A++ A +TAP +V + G + A +

Sbjct 75 RIGRRPILLAGATAFVIASLGAAWSSTAP-AFVAFRLLQAVGASAMLVATFATVRDVYAN 133

Query 1609 GDERARHFGFMSACFGFGMVAGPVLGGLMGGF 1704

E +G S+ F GP+ G L+G F

Sbjct 134 RPEGVVIYGLFSSMLAFVPALGPIAGALIGEF 165

> ACQ78004 FloR (plasmid) [Escherichia coli]

Length=404

Score = 40.0 bits (92), Expect = 3e-04, Method: Compositional matrix adjust.

Identities = 45/152 (30%), Positives = 70/152 (46%), Gaps = 14/152 (9%)

Frame = +1

Query 1261 ILSTVALDAVGIGL-IMPVLPGLLRDLVHSNDVTAHYGILLALYALMQFACAPVLGALSD 1437

IL+++A+D I L ++P +PG+L N A + L+LY +M + G LSD

Sbjct 24 ILASLAMD---IYLPVVPAMPGIL------NTTPAMIQLTLSLYMVMLGVGQVIFGPLSD 74

Query 1438 RFGRRPVLL---VSLAGAAVDYAIMATAPFLWVLYigrivagitgatgavagayiaDITD 1608

R GRRP+LL + A++ A +TAP +V + G + A +

Sbjct 75 RIGRRPILLAGATAFVIASLGAAWSSTAP-AFVAFRLLQAVGASAMLVATFATVRDVYAN 133

Query 1609 GDERARHFGFMSACFGFGMVAGPVLGGLMGGF 1704

E +G S+ F GP+ G L+G F

Sbjct 134 RPEGVVIYGLFSSMLAFVPALGPIAGALIGEF 165

> SBZ72551 putative chloramphenicol and florfenicol resistance

protein (CmlA) [Klebsiella pneumoniae]

Length=404

Score = 40.0 bits (92), Expect = 3e-04, Method: Compositional matrix adjust.

Identities = 45/152 (30%), Positives = 70/152 (46%), Gaps = 14/152 (9%)

Frame = +1

Query 1261 ILSTVALDAVGIGL-IMPVLPGLLRDLVHSNDVTAHYGILLALYALMQFACAPVLGALSD 1437

IL+++A+D I L ++P +PG+L N A + L+LY +M + G LSD

Sbjct 24 ILASLAMD---IYLPVVPAMPGIL------NTTPAMIQLTLSLYMVMLGVGQVIFGPLSD 74

Query 1438 RFGRRPVLL---VSLAGAAVDYAIMATAPFLWVLYigrivagitgatgavagayiaDITD 1608

R GRRP+LL + A++ A +TAP +V + G + A +

Sbjct 75 RIGRRPILLAGATAFVIASLGAAWSSTAP-AFVAFRLLQAVGASAMLVATFATVRDVYAN 133

Query 1609 GDERARHFGFMSACFGFGMVAGPVLGGLMGGF 1704

E +G S+ F GP+ G L+G F

Sbjct 134 RPEGVVIYGLFSSMLAFVPALGPIAGALIGEF 165

> AAT38901 putative efflux protein Flor, partial [Salmonella enterica

subsp. enterica serovar Pullorum]

Length=173

Score = 38.5 bits (88), Expect = 3e-04, Method: Compositional matrix adjust.

Identities = 45/152 (30%), Positives = 70/152 (46%), Gaps = 14/152 (9%)

Frame = +1

Query 1261 ILSTVALDAVGIGL-IMPVLPGLLRDLVHSNDVTAHYGILLALYALMQFACAPVLGALSD 1437

IL+++A+D I L ++P +PG+L N A + L+LY +M + G LSD

Sbjct 12 ILASLAMD---IYLPVVPAMPGIL------NTTPAMIQLTLSLYMVMLGVGQVIFGPLSD 62

Query 1438 RFGRRPVLL---VSLAGAAVDYAIMATAPFLWVLYigrivagitgatgavagayiaDITD 1608

R GRRP+LL + A++ A +TAP +V + G + A +

Sbjct 63 RIGRRPILLAGATAFVIASLGAAWSSTAP-AFVAFRLLQAVGASAMLVATFATVRDVYAN 121

Query 1609 GDERARHFGFMSACFGFGMVAGPVLGGLMGGF 1704

E +G S+ F GP+ G L+G F

Sbjct 122 RPEGVVIYGLFSSMLAFVPALGPIAGVLIGEF 153

> CAJ57806 florfenicol resistance protein, partial (plasmid) [Stenotrophomonas

maltophilia]

Length=382

Score = 39.7 bits (91), Expect = 3e-04, Method: Compositional matrix adjust.

Identities = 45/152 (30%), Positives = 70/152 (46%), Gaps = 14/152 (9%)

Frame = +1

Query 1261 ILSTVALDAVGIGL-IMPVLPGLLRDLVHSNDVTAHYGILLALYALMQFACAPVLGALSD 1437

IL+++A+D I L ++P +PG+L N A + L+LY +M + G LSD

Sbjct 2 ILASLAMD---IYLPVVPAMPGIL------NTTPAMIQLTLSLYMVMLGVGQVIFGPLSD 52

Query 1438 RFGRRPVLL---VSLAGAAVDYAIMATAPFLWVLYigrivagitgatgavagayiaDITD 1608

R GRRP+LL + A++ A +TAP +V + G + A +

Sbjct 53 RIGRRPILLAGATAFVIASLGAAWSSTAP-AFVAFRLLQAVGASAMLVATFATVRDVYAN 111

Query 1609 GDERARHFGFMSACFGFGMVAGPVLGGLMGGF 1704

E +G S+ F GP+ G L+G F

Sbjct 112 RPEGVVIYGLFSSVLAFVPALGPIAGALIGEF 143

> CAJ57802 florfenicol resistance protein, partial (plasmid) [Stenotrophomonas

maltophilia]

Length=382

Score = 39.7 bits (91), Expect = 3e-04, Method: Compositional matrix adjust.

Identities = 45/152 (30%), Positives = 70/152 (46%), Gaps = 14/152 (9%)

Frame = +1

Query 1261 ILSTVALDAVGIGL-IMPVLPGLLRDLVHSNDVTAHYGILLALYALMQFACAPVLGALSD 1437

IL+++A+D I L ++P +PG+L N A + L+LY +M + G LSD

Sbjct 2 ILASLAMD---IYLPVVPAMPGIL------NTTPAMIQLTLSLYMVMLGVGQVIFGPLSD 52

Query 1438 RFGRRPVLL---VSLAGAAVDYAIMATAPFLWVLYigrivagitgatgavagayiaDITD 1608

R GRRP+LL + A++ A +TAP +V + G + A +

Sbjct 53 RIGRRPILLAGATAFVIASLGAAWSSTAP-AFVAFRLLQAVGASAMLVATFATVRDVYAN 111

Query 1609 GDERARHFGFMSACFGFGMVAGPVLGGLMGGF 1704

E +G S+ F GP+ G L+G F

Sbjct 112 RPEGVVIYGLFSSVLAFVPALGPIAGALIGEF 143

> floR_-_3936963_translation floR: phenicol exporter

Length=404

Score = 39.7 bits (91), Expect = 3e-04, Method: Compositional matrix adjust.

Identities = 39/137 (28%), Positives = 60/137 (44%), Gaps = 10/137 (7%)

Frame = +1

Query 1303 IMPVLPGLLRDLVHSNDVTAHYGILLALYALMQFACAPVLGALSDRFGRRPVLL---VSL 1473

++P +PG+L N A + L+LY +M + G LSDR GRRP+LL +

Sbjct 36 VVPAMPGIL------NTTPAMIQLTLSLYMVMLGVGQVIFGPLSDRIGRRPILLAGATAF 89

Query 1474 AGAAVDYAIMATAPFLWVLYigrivagitgatgavagayiaDITDGDERARHFGFMSACF 1653

A++ A +TAP +V + G + A + E +G S+

Sbjct 90 VIASLGAAWSSTAP-AFVAFRLLQAVGASAMLVATFATVRDVYANRPEGVVIYGLFSSIL 148

Query 1654 GFGMVAGPVLGGLMGGF 1704

F GP+ G L+G F

Sbjct 149 AFVPALGPIAGALIGEF 165

> YP_512240 phenicol exporter (plasmid) [Bibersteinia trehalosi]

Length=404

Score = 39.7 bits (91), Expect = 3e-04, Method: Compositional matrix adjust.

Identities = 39/137 (28%), Positives = 60/137 (44%), Gaps = 10/137 (7%)

Frame = +1

Query 1303 IMPVLPGLLRDLVHSNDVTAHYGILLALYALMQFACAPVLGALSDRFGRRPVLL---VSL 1473

++P +PG+L N A + L+LY +M + G LSDR GRRP+LL +

Sbjct 36 VVPAMPGIL------NTTPAMIQLTLSLYMVMLGVGQVIFGPLSDRIGRRPILLAGATAF 89

Query 1474 AGAAVDYAIMATAPFLWVLYigrivagitgatgavagayiaDITDGDERARHFGFMSACF 1653

A++ A +TAP +V + G + A + E +G S+

Sbjct 90 VIASLGAAWSSTAP-AFVAFRLLQAVGASAMLVATFATVRDVYANRPEGVVIYGLFSSIL 148

Query 1654 GFGMVAGPVLGGLMGGF 1704

F GP+ G L+G F

Sbjct 149 AFVPALGPIAGALIGEF 165

> WP_011453054 MULTISPECIES: chloramphenicol/florfenicol efflux

MFS transporter FloR [Gammaproteobacteria]

Length=404

Score = 39.7 bits (91), Expect = 3e-04, Method: Compositional matrix adjust.

Identities = 39/137 (28%), Positives = 60/137 (44%), Gaps = 10/137 (7%)

Frame = +1

Query 1303 IMPVLPGLLRDLVHSNDVTAHYGILLALYALMQFACAPVLGALSDRFGRRPVLL---VSL 1473

++P +PG+L N A + L+LY +M + G LSDR GRRP+LL +

Sbjct 36 VVPAMPGIL------NTTPAMIQLTLSLYMVMLGVGQVIFGPLSDRIGRRPILLAGATAF 89

Query 1474 AGAAVDYAIMATAPFLWVLYigrivagitgatgavagayiaDITDGDERARHFGFMSACF 1653

A++ A +TAP +V + G + A + E +G S+

Sbjct 90 VIASLGAAWSSTAP-AFVAFRLLQAVGASAMLVATFATVRDVYANRPEGVVIYGLFSSIL 148

Query 1654 GFGMVAGPVLGGLMGGF 1704

F GP+ G L+G F

Sbjct 149 AFVPALGPIAGALIGEF 165

> CAJ65911 phenicol exporter (plasmid) [Bibersteinia trehalosi]

Length=404

Score = 39.7 bits (91), Expect = 3e-04, Method: Compositional matrix adjust.

Identities = 39/137 (28%), Positives = 60/137 (44%), Gaps = 10/137 (7%)

Frame = +1

Query 1303 IMPVLPGLLRDLVHSNDVTAHYGILLALYALMQFACAPVLGALSDRFGRRPVLL---VSL 1473

++P +PG+L N A + L+LY +M + G LSDR GRRP+LL +

Sbjct 36 VVPAMPGIL------NTTPAMIQLTLSLYMVMLGVGQVIFGPLSDRIGRRPILLAGATAF 89

Query 1474 AGAAVDYAIMATAPFLWVLYigrivagitgatgavagayiaDITDGDERARHFGFMSACF 1653

A++ A +TAP +V + G + A + E +G S+

Sbjct 90 VIASLGAAWSSTAP-AFVAFRLLQAVGASAMLVATFATVRDVYANRPEGVVIYGLFSSIL 148

Query 1654 GFGMVAGPVLGGLMGGF 1704

F GP+ G L+G F

Sbjct 149 AFVPALGPIAGALIGEF 165

> AMK38073 florfenicol exporter [Proteus mirabilis]

Length=389

Score = 39.7 bits (91), Expect = 3e-04, Method: Compositional matrix adjust.

Identities = 45/152 (30%), Positives = 70/152 (46%), Gaps = 14/152 (9%)

Frame = +1

Query 1261 ILSTVALDAVGIGL-IMPVLPGLLRDLVHSNDVTAHYGILLALYALMQFACAPVLGALSD 1437

IL+++A+D I L ++P +PG+L N A + L+LY +M + G LSD

Sbjct 9 ILASLAMD---IYLPVVPAMPGIL------NTTPAMIQLTLSLYMVMLGVGQVIFGPLSD 59

Query 1438 RFGRRPVLL---VSLAGAAVDYAIMATAPFLWVLYigrivagitgatgavagayiaDITD 1608

R GRRP+LL + A++ A +TAP +V + G + A +

Sbjct 60 RIGRRPILLAGATAFVIASLGAAWSSTAP-AFVAFRLLQAVGASAMLVATFATVRDVYAN 118

Query 1609 GDERARHFGFMSACFGFGMVAGPVLGGLMGGF 1704

E +G S+ F GP+ G L+G F

Sbjct 119 RPEGVVIYGLFSSMLAFVPALGPIAGALIGEF 150

> CCU69833 florfenicol-chloramphenicol exporter [Staphylococcus

pseudintermedius]

Length=475

Score = 39.7 bits (91), Expect = 3e-04, Method: Compositional matrix adjust.

Identities = 45/177 (25%), Positives = 86/177 (49%), Gaps = 13/177 (7%)

Frame = +1

Query 1192 RVQSDQRRLVRRPDVKPNRPLIVILS-TVALDAVGIGLIMPVLPGLLRDLVHSND----V 1356

+ S + +++ R L+++LS +V + A+ + L+ PVLP + +DL S +

Sbjct 2 KKDSKSKEMIQSEKRGSTRLLMMVLSLSVLVAAITVDLVNPVLPLISKDLEASKSQVSWI 61

Query 1357 TAHYGILLALYALMQFACAPVLGALSDRFGRRPVLLVSLAGAAVDYAIMATAPFLWVLYi 1536

+ I+LA+ P+ G +SD F R + + ++ A + A AP L +L +

Sbjct 62 VSGIAIVLAI-------GVPIYGRISDFFELRKLYIFAIMILASGSLLCAIAPNLPLLVL 114

Query 1537 grivagitgatg-avagayiaDITDGDERARHFGFMSACFGFGMVAGPVLGGLMGGF 1704

GR+V G + ++ I+ + +R G ++ G G AGP+ GG++G +

Sbjct 115 GRMVQGAGMSAIPVLSVIAISKVFPQGKRGGALGIIAGSIGVGTAAGPIFGGVVGQY 171

> ABO10438 florfenicol export protein [Escherichia coli]

Length=404

Score = 39.7 bits (91), Expect = 4e-04, Method: Compositional matrix adjust.

Identities = 45/152 (30%), Positives = 70/152 (46%), Gaps = 14/152 (9%)

Frame = +1

Query 1261 ILSTVALDAVGIGL-IMPVLPGLLRDLVHSNDVTAHYGILLALYALMQFACAPVLGALSD 1437

IL+++A+D I L ++P +PG+L N A + L+LY +M + G LSD

Sbjct 24 ILASLAMD---IYLPVVPAMPGIL------NTTPAMIQLTLSLYMVMLGVGQVIFGPLSD 74

Query 1438 RFGRRPVLL---VSLAGAAVDYAIMATAPFLWVLYigrivagitgatgavagayiaDITD 1608

R GRRP+LL + A++ A +TAP +V + G + A +

Sbjct 75 RIGRRPILLAGATAFVIASLGAAWSSTAP-AFVAFRLLQAVGASAMLVATFATVRDVYAN 133

Query 1609 GDERARHFGFMSACFGFGMVAGPVLGGLMGGF 1704

E +G S+ F GP+ G L+G F

Sbjct 134 RPEGVVIYGLFSSMLAFVPALGPIAGTLIGEF 165

> floR_-_17035768_translation floR: florfenicol/ chloramphenicol

export protein FloR

Length=404

Score = 39.7 bits (91), Expect = 4e-04, Method: Compositional matrix adjust.

Identities = 45/152 (30%), Positives = 70/152 (46%), Gaps = 14/152 (9%)

Frame = +1

Query 1261 ILSTVALDAVGIGL-IMPVLPGLLRDLVHSNDVTAHYGILLALYALMQFACAPVLGALSD 1437

IL+++A+D I L ++P +PG+L N A + L+LY +M + G LSD

Sbjct 24 ILASLAMD---IYLPVVPAMPGIL------NTTPAMIQLTLSLYMVMLGVGQVIFGPLSD 74

Query 1438 RFGRRPVLL---VSLAGAAVDYAIMATAPFLWVLYigrivagitgatgavagayiaDITD 1608

R GRRP+LL + A++ A +TAP +V + G + A +

Sbjct 75 RIGRRPILLAGATAFVIASLGAAWSSTAP-AFVAFRLLQAVGASAMLVATFATVRDVYAN 133

Query 1609 GDERARHFGFMSACFGFGMVAGPVLGGLMGGF 1704

E +G S+ F GP+ G L+G F

Sbjct 134 RPEGVVIYGLFSSMLAFVPALGPIAGTLIGEF 165

> YP_008574993 florfenicol/ chloramphenicol export protein FloR

(plasmid) [Escherichia coli]

Length=404

Score = 39.7 bits (91), Expect = 4e-04, Method: Compositional matrix adjust.

Identities = 45/152 (30%), Positives = 70/152 (46%), Gaps = 14/152 (9%)

Frame = +1

Query 1261 ILSTVALDAVGIGL-IMPVLPGLLRDLVHSNDVTAHYGILLALYALMQFACAPVLGALSD 1437

IL+++A+D I L ++P +PG+L N A + L+LY +M + G LSD

Sbjct 24 ILASLAMD---IYLPVVPAMPGIL------NTTPAMIQLTLSLYMVMLGVGQVIFGPLSD 74

Query 1438 RFGRRPVLL---VSLAGAAVDYAIMATAPFLWVLYigrivagitgatgavagayiaDITD 1608

R GRRP+LL + A++ A +TAP +V + G + A +

Sbjct 75 RIGRRPILLAGATAFVIASLGAAWSSTAP-AFVAFRLLQAVGASAMLVATFATVRDVYAN 133

Query 1609 GDERARHFGFMSACFGFGMVAGPVLGGLMGGF 1704

E +G S+ F GP+ G L+G F

Sbjct 134 RPEGVVIYGLFSSMLAFVPALGPIAGTLIGEF 165

> AGW01038 florfenicol/ chloramphenicol export protein FloR (plasmid)

[Escherichia coli]

Length=404

Score = 39.7 bits (91), Expect = 4e-04, Method: Compositional matrix adjust.

Identities = 45/152 (30%), Positives = 70/152 (46%), Gaps = 14/152 (9%)

Frame = +1

Query 1261 ILSTVALDAVGIGL-IMPVLPGLLRDLVHSNDVTAHYGILLALYALMQFACAPVLGALSD 1437

IL+++A+D I L ++P +PG+L N A + L+LY +M + G LSD

Sbjct 24 ILASLAMD---IYLPVVPAMPGIL------NTTPAMIQLTLSLYMVMLGVGQVIFGPLSD 74

Query 1438 RFGRRPVLL---VSLAGAAVDYAIMATAPFLWVLYigrivagitgatgavagayiaDITD 1608

R GRRP+LL + A++ A +TAP +V + G + A +

Sbjct 75 RIGRRPILLAGATAFVIASLGAAWSSTAP-AFVAFRLLQAVGASAMLVATFATVRDVYAN 133

Query 1609 GDERARHFGFMSACFGFGMVAGPVLGGLMGGF 1704

E +G S+ F GP+ G L+G F

Sbjct 134 RPEGVVIYGLFSSMLAFVPALGPIAGTLIGEF 165

> ABO10437 florfenicol export protein [Escherichia coli]

Length=404

Score = 39.7 bits (91), Expect = 4e-04, Method: Compositional matrix adjust.

Identities = 45/152 (30%), Positives = 70/152 (46%), Gaps = 14/152 (9%)

Frame = +1

Query 1261 ILSTVALDAVGIGL-IMPVLPGLLRDLVHSNDVTAHYGILLALYALMQFACAPVLGALSD 1437

IL+++A+D I L ++P +PG+L N A + L+LY +M + G LSD

Sbjct 24 ILASLAMD---IYLPVVPAMPGIL------NTTPAMIQLTLSLYMVMLGVGQVIFGPLSD 74

Query 1438 RFGRRPVLL---VSLAGAAVDYAIMATAPFLWVLYigrivagitgatgavagayiaDITD 1608

R GRRP+LL + A++ A +TAP +V + G + A +

Sbjct 75 RIGRRPILLAGATAFVIASLGAAWSSTAP-AFVAFRLLQAVGASAMLVATFATVRDVYAN 133

Query 1609 GDERARHFGFMSACFGFGMVAGPVLGGLMGGF 1704

E +G S+ F GP+ G L+G F

Sbjct 134 RPEGVVIYGLFSSMLAFVPALGPIAGTLIGEF 165

> SCA25268 putative chloramphenicol and florfenicol resistance

protein (CmlA) [Klebsiella pneumoniae]

Length=404

Score = 39.7 bits (91), Expect = 4e-04, Method: Compositional matrix adjust.

Identities = 45/152 (30%), Positives = 70/152 (46%), Gaps = 14/152 (9%)

Frame = +1

Query 1261 ILSTVALDAVGIGL-IMPVLPGLLRDLVHSNDVTAHYGILLALYALMQFACAPVLGALSD 1437

IL+++A+D I L ++P +PG+L N A + L+LY +M + G LSD

Sbjct 24 ILASLAMD---IYLPVVPAMPGIL------NTTPAMIQLTLSLYMVMLGVGQVIFGPLSD 74

Query 1438 RFGRRPVLL---VSLAGAAVDYAIMATAPFLWVLYigrivagitgatgavagayiaDITD 1608

R GRRP+LL + A++ A +TAP +V + G + A +

Sbjct 75 RIGRRPILLAGATAFVIASLGAAWSSTAP-AFVAFRLLQAVGASAMLVATFATVRDVYAN 133

Query 1609 GDERARHFGFMSACFGFGMVAGPVLGGLMGGF 1704

E +G S+ F GP+ G L+G F

Sbjct 134 RPEGVVIYGLFSSMLAFVPALGPIAGTLIGEF 165

> SBZ44164 putative chloramphenicol and florfenicol resistance

protein (CmlA) [Klebsiella pneumoniae]

Length=404

Score = 39.7 bits (91), Expect = 4e-04, Method: Compositional matrix adjust.

Identities = 45/152 (30%), Positives = 70/152 (46%), Gaps = 14/152 (9%)

Frame = +1

Query 1261 ILSTVALDAVGIGL-IMPVLPGLLRDLVHSNDVTAHYGILLALYALMQFACAPVLGALSD 1437

IL+++A+D I L ++P +PG+L N A + L+LY +M + G LSD

Sbjct 24 ILASLAMD---IYLPVVPAMPGIL------NTTPAMIQLTLSLYMVMLGVGQVIFGPLSD 74

Query 1438 RFGRRPVLL---VSLAGAAVDYAIMATAPFLWVLYigrivagitgatgavagayiaDITD 1608

R GRRP+LL + A++ A +TAP +V + G + A +

Sbjct 75 RIGRRPILLAGATAFVIASLGAAWSSTAP-AFVAFRLLQAVGASAMLVATFATVRDVYAN 133

Query 1609 GDERARHFGFMSACFGFGMVAGPVLGGLMGGF 1704

E +G S+ F GP+ G L+G F

Sbjct 134 RPEGVVIYGLFSSMLAFVPALGPIAGTLIGEF 165

> BAX18835 florfenicol/ chloramphenicol export protein FloR [Escherichia

coli]

Length=404

Score = 39.7 bits (91), Expect = 4e-04, Method: Compositional matrix adjust.

Identities = 45/152 (30%), Positives = 70/152 (46%), Gaps = 14/152 (9%)

Frame = +1

Query 1261 ILSTVALDAVGIGL-IMPVLPGLLRDLVHSNDVTAHYGILLALYALMQFACAPVLGALSD 1437

IL+++A+D I L ++P +PG+L N A + L+LY +M + G LSD

Sbjct 24 ILASLAMD---IYLPVVPAMPGIL------NTTPAMIQLTLSLYMVMLGVGQVIFGPLSD 74

Query 1438 RFGRRPVLL---VSLAGAAVDYAIMATAPFLWVLYigrivagitgatgavagayiaDITD 1608

R GRRP+LL + A++ A +TAP +V + G + A +

Sbjct 75 RIGRRPILLAGATAFVIASLGAAWSSTAP-AFVAFRLLQAVGASAMLVATFATVRDVYAN 133

Query 1609 GDERARHFGFMSACFGFGMVAGPVLGGLMGGF 1704

E +G S+ F GP+ G L+G F

Sbjct 134 RPEGVVIYGLFSSMLAFVPALGPIAGTLIGEF 165

> ASK37765 Florfenicol resistance protein (plasmid) [Escherichia

coli]

Length=404

Score = 39.7 bits (91), Expect = 4e-04, Method: Compositional matrix adjust.

Identities = 45/152 (30%), Positives = 70/152 (46%), Gaps = 14/152 (9%)

Frame = +1

Query 1261 ILSTVALDAVGIGL-IMPVLPGLLRDLVHSNDVTAHYGILLALYALMQFACAPVLGALSD 1437

IL+++A+D I L ++P +PG+L N A + L+LY +M + G LSD

Sbjct 24 ILASLAMD---IYLPVVPAMPGIL------NTTPAMIQLTLSLYMVMLGVGQVIFGPLSD 74

Query 1438 RFGRRPVLL---VSLAGAAVDYAIMATAPFLWVLYigrivagitgatgavagayiaDITD 1608

R GRRP+LL + A++ A +TAP +V + G + A +

Sbjct 75 RIGRRPILLAGATAFVIASLGAAWSSTAP-AFVAFRLLQAVGASAMLVATFATVRDVYAN 133

Query 1609 GDERARHFGFMSACFGFGMVAGPVLGGLMGGF 1704

E +G S+ F GP+ G L+G F

Sbjct 134 RPEGVVIYGLFSSMLAFVPALGPIAGTLIGEF 165

> ASF20149 florfenicol exporter [Proteus mirabilis]

Length=404

Score = 39.7 bits (91), Expect = 4e-04, Method: Compositional matrix adjust.

Identities = 45/152 (30%), Positives = 70/152 (46%), Gaps = 14/152 (9%)

Frame = +1

Query 1261 ILSTVALDAVGIGL-IMPVLPGLLRDLVHSNDVTAHYGILLALYALMQFACAPVLGALSD 1437

IL+++A+D I L ++P +PG+L N A + L+LY +M + G LSD

Sbjct 24 ILASLAMD---IYLPVVPAMPGIL------NTTPAMIQLTLSLYMVMLGVGQVIFGPLSD 74

Query 1438 RFGRRPVLL---VSLAGAAVDYAIMATAPFLWVLYigrivagitgatgavagayiaDITD 1608

R GRRP+LL + A++ A +TAP +V + G + A +

Sbjct 75 RIGRRPILLAGATAFVIASLGAAWSSTAP-AFVAFRLLQAVGASAMLVATFATVRDVYAN 133

Query 1609 GDERARHFGFMSACFGFGMVAGPVLGGLMGGF 1704

E +G S+ F GP+ G L+G F

Sbjct 134 RPEGVVIYGLFSSMLAFVPALGPIAGTLIGEF 165

> ANA09319 florfenicol export protein (plasmid) [Escherichia coli]

Length=404

Score = 39.7 bits (91), Expect = 4e-04, Method: Compositional matrix adjust.

Identities = 45/152 (30%), Positives = 70/152 (46%), Gaps = 14/152 (9%)

Frame = +1

Query 1261 ILSTVALDAVGIGL-IMPVLPGLLRDLVHSNDVTAHYGILLALYALMQFACAPVLGALSD 1437

IL+++A+D I L ++P +PG+L N A + L+LY +M + G LSD

Sbjct 24 ILASLAMD---IYLPVVPAMPGIL------NTTPAMIQLTLSLYMVMLGVGQVIFGPLSD 74

Query 1438 RFGRRPVLL---VSLAGAAVDYAIMATAPFLWVLYigrivagitgatgavagayiaDITD 1608

R GRRP+LL + A++ A +TAP +V + G + A +

Sbjct 75 RIGRRPILLAGATAFVIASLGAAWSSTAP-AFVAFRLLQAVGASAMLVATFATVRDVYAN 133

Query 1609 GDERARHFGFMSACFGFGMVAGPVLGGLMGGF 1704

E +G S+ F GP+ G L+G F

Sbjct 134 RPEGVVIYGLFSSMLAFVPALGPIAGTLIGEF 165

> ALP69238 florfenicol exporter [Proteus mirabilis]

Length=404

Score = 39.7 bits (91), Expect = 4e-04, Method: Compositional matrix adjust.

Identities = 45/152 (30%), Positives = 70/152 (46%), Gaps = 14/152 (9%)

Frame = +1

Query 1261 ILSTVALDAVGIGL-IMPVLPGLLRDLVHSNDVTAHYGILLALYALMQFACAPVLGALSD 1437

IL+++A+D I L ++P +PG+L N A + L+LY +M + G LSD

Sbjct 24 ILASLAMD---IYLPVVPAMPGIL------NTTPAMIQLTLSLYMVMLGVGQVIFGPLSD 74

Query 1438 RFGRRPVLL---VSLAGAAVDYAIMATAPFLWVLYigrivagitgatgavagayiaDITD 1608

R GRRP+LL + A++ A +TAP +V + G + A +

Sbjct 75 RIGRRPILLAGATAFVIASLGAAWSSTAP-AFVAFRLLQAVGASAMLVATFATVRDVYAN 133

Query 1609 GDERARHFGFMSACFGFGMVAGPVLGGLMGGF 1704

E +G S+ F GP+ G L+G F

Sbjct 134 RPEGVVIYGLFSSMLAFVPALGPIAGTLIGEF 165

> ABA64519 florfenicol export protein [Escherichia coli]

Length=404

Score = 39.7 bits (91), Expect = 4e-04, Method: Compositional matrix adjust.

Identities = 45/152 (30%), Positives = 70/152 (46%), Gaps = 14/152 (9%)

Frame = +1

Query 1261 ILSTVALDAVGIGL-IMPVLPGLLRDLVHSNDVTAHYGILLALYALMQFACAPVLGALSD 1437

IL+++A+D I L ++P +PG+L N A + L+LY +M + G LSD

Sbjct 24 ILASLAMD---IYLPVVPAMPGIL------NTTPAMIQLTLSLYMVMLGVGQVIFGPLSD 74

Query 1438 RFGRRPVLL---VSLAGAAVDYAIMATAPFLWVLYigrivagitgatgavagayiaDITD 1608

R GRRP+LL + A++ A +TAP +V + G + A +

Sbjct 75 RIGRRPILLAGATAFVIASLGAAWSSTAP-AFVAFRLLQAVGASAMLVATFATVRDVYAN 133

Query 1609 GDERARHFGFMSACFGFGMVAGPVLGGLMGGF 1704

E +G S+ F GP+ G L+G F

Sbjct 134 RPEGVVIYGLFSSMLAFVPALGPIAGTLIGEF 165

> ABG36701 chloramphenicol and florfenicol resistance [Salmonella

enterica subsp. enterica serovar Newport]

Length=404

Score = 39.3 bits (90), Expect = 5e-04, Method: Compositional matrix adjust.

Identities = 45/152 (30%), Positives = 69/152 (45%), Gaps = 14/152 (9%)

Frame = +1

Query 1261 ILSTVALDAVGIGL-IMPVLPGLLRDLVHSNDVTAHYGILLALYALMQFACAPVLGALSD 1437

IL+++A+D I L ++P +PG+L N A + L+LY +M + G LSD

Sbjct 24 ILASLAMD---IYLPVVPAMPGIL------NTTPAMIQLTLSLYMVMLGVGQVIFGPLSD 74

Query 1438 RFGRRPVLL---VSLAGAAVDYAIMATAPFLWVLYigrivagitgatgavagayiaDITD 1608

R GRRP+LL + A++ A TAP +V + G + A +

Sbjct 75 RIGRRPILLAGATAFVIASLGAAWSPTAP-AFVAFRLLQAVGASAMLVATFATVRDVYAN 133

Query 1609 GDERARHFGFMSACFGFGMVAGPVLGGLMGGF 1704

E +G S+ F GP+ G L+G F

Sbjct 134 RPEGVVIYGLFSSMLAFVPALGPIAGALIGEF 165

> floR_-_11934200_translation floR: florfenicol/chloramphenicol

resistance protein

Length=404

Score = 39.3 bits (90), Expect = 5e-04, Method: Compositional matrix adjust.

Identities = 45/152 (30%), Positives = 69/152 (45%), Gaps = 14/152 (9%)

Frame = +1

Query 1261 ILSTVALDAVGIGL-IMPVLPGLLRDLVHSNDVTAHYGILLALYALMQFACAPVLGALSD 1437

IL+++A+D I L ++P +PG+L N A + L+LY +M + G LSD

Sbjct 24 ILASLAMD---IYLPVVPAMPGIL------NTTPAMIQLTLSLYMVMLGVGQVIFGPLSD 74

Query 1438 RFGRRPVLL---VSLAGAAVDYAIMATAPFLWVLYigrivagitgatgavagayiaDITD 1608

R GRRP+LL + A++ A +TAP +V + G + A

Sbjct 75 RIGRRPILLAGATAFVIASLGAAWSSTAP-AFVAFRLLQAVGASAMLVATFATVRDVYAS 133

Query 1609 GDERARHFGFMSACFGFGMVAGPVLGGLMGGF 1704

E +G S+ F GP+ G L+G F

Sbjct 134 RPEGVVIYGLFSSMLAFVPALGPIAGALIGEF 165

> AJT60292 FloR cmlA family efflux protein (plasmid) [Escherichia

coli]

Length=404

Score = 39.3 bits (90), Expect = 5e-04, Method: Compositional matrix adjust.

Identities = 45/152 (30%), Positives = 69/152 (45%), Gaps = 14/152 (9%)

Frame = +1

Query 1261 ILSTVALDAVGIGL-IMPVLPGLLRDLVHSNDVTAHYGILLALYALMQFACAPVLGALSD 1437

IL+++A+D I L ++P +PG+L N A + L+LY +M + G LSD

Sbjct 24 ILASLAMD---IYLPVVPAMPGIL------NTTPAMIQLTLSLYMVMLGVGQVIFGPLSD 74

Query 1438 RFGRRPVLL---VSLAGAAVDYAIMATAPFLWVLYigrivagitgatgavagayiaDITD 1608

R GRRP+LL + A++ A +TAP +V + G + A

Sbjct 75 RIGRRPILLAGATAFVIASLGAAWSSTAP-AFVAFRLLQAVGASAMLVATFATVRDVYAS 133

Query 1609 GDERARHFGFMSACFGFGMVAGPVLGGLMGGF 1704

E +G S+ F GP+ G L+G F

Sbjct 134 RPEGVVIYGLFSSMLAFVPALGPIAGALIGEF 165

> AF332662_1 florfenicol export protein (plasmid) [Klebsiella pneumoniae]

Length=404

Score = 39.3 bits (90), Expect = 5e-04, Method: Compositional matrix adjust.

Identities = 45/152 (30%), Positives = 69/152 (45%), Gaps = 14/152 (9%)

Frame = +1

Query 1261 ILSTVALDAVGIGL-IMPVLPGLLRDLVHSNDVTAHYGILLALYALMQFACAPVLGALSD 1437

IL+++A+D I L ++P +PG+L N A + L+LY +M + G LSD

Sbjct 24 ILASLAMD---IYLPVVPAMPGIL------NTTPAMIQLTLSLYMVMLGVGQVIFGPLSD 74

Query 1438 RFGRRPVLL---VSLAGAAVDYAIMATAPFLWVLYigrivagitgatgavagayiaDITD 1608

R GRRP+LL + A++ A +TAP +V + G + A

Sbjct 75 RIGRRPILLAGATAFVIASLGAAWSSTAP-AFVAFRLLQAVGASAMLVATFATVRDVYAS 133

Query 1609 GDERARHFGFMSACFGFGMVAGPVLGGLMGGF 1704

E +G S+ F GP+ G L+G F

Sbjct 134 RPEGVVIYGLFSSMLAFVPALGPIAGALIGEF 165

> YP_004810311 florfenicol export protein (plasmid) [Riemerella

anatipestifer]

Length=407

Score = 38.9 bits (89), Expect = 5e-04, Method: Compositional matrix adjust.

Identities = 45/152 (30%), Positives = 70/152 (46%), Gaps = 14/152 (9%)

Frame = +1

Query 1261 ILSTVALDAVGIGL-IMPVLPGLLRDLVHSNDVTAHYGILLALYALMQFACAPVLGALSD 1437

IL+++A+D I L ++P +PG+L N A + L+LY +M + G LSD

Sbjct 27 ILASLAMD---IYLPVVPAMPGIL------NTTPAMIQLTLSLYMVMLGVGQVIFGPLSD 77

Query 1438 RFGRRPVLL---VSLAGAAVDYAIMATAPFLWVLYigrivagitgatgavagayiaDITD 1608

R GRRP+LL + A++ A +TAP +V + G + A +

Sbjct 78 RIGRRPILLAGATAFVIASLGAAWSSTAP-AFVAFRLLQAVGASAMLVATFATVRDVYAN 136

Query 1609 GDERARHFGFMSACFGFGMVAGPVLGGLMGGF 1704

E +G S+ F GP+ G L+G F

Sbjct 137 RPEGVVIYGLFSSMLAFVPALGPIAGVLIGEF 168

> WP_014053560 chloramphenicol/florfenicol efflux MFS transporter

FloR [Riemerella anatipestifer]

Length=407

Score = 38.9 bits (89), Expect = 5e-04, Method: Compositional matrix adjust.

Identities = 45/152 (30%), Positives = 70/152 (46%), Gaps = 14/152 (9%)

Frame = +1

Query 1261 ILSTVALDAVGIGL-IMPVLPGLLRDLVHSNDVTAHYGILLALYALMQFACAPVLGALSD 1437

IL+++A+D I L ++P +PG+L N A + L+LY +M + G LSD

Sbjct 27 ILASLAMD---IYLPVVPAMPGIL------NTTPAMIQLTLSLYMVMLGVGQVIFGPLSD 77

Query 1438 RFGRRPVLL---VSLAGAAVDYAIMATAPFLWVLYigrivagitgatgavagayiaDITD 1608

R GRRP+LL + A++ A +TAP +V + G + A +

Sbjct 78 RIGRRPILLAGATAFVIASLGAAWSSTAP-AFVAFRLLQAVGASAMLVATFATVRDVYAN 136

Query 1609 GDERARHFGFMSACFGFGMVAGPVLGGLMGGF 1704

E +G S+ F GP+ G L+G F

Sbjct 137 RPEGVVIYGLFSSMLAFVPALGPIAGVLIGEF 168

> AEM66521 florfenicol export protein (plasmid) [Riemerella anatipestifer]

Length=407

Score = 38.9 bits (89), Expect = 5e-04, Method: Compositional matrix adjust.

Identities = 45/152 (30%), Positives = 70/152 (46%), Gaps = 14/152 (9%)

Frame = +1

Query 1261 ILSTVALDAVGIGL-IMPVLPGLLRDLVHSNDVTAHYGILLALYALMQFACAPVLGALSD 1437

IL+++A+D I L ++P +PG+L N A + L+LY +M + G LSD

Sbjct 27 ILASLAMD---IYLPVVPAMPGIL------NTTPAMIQLTLSLYMVMLGVGQVIFGPLSD 77

Query 1438 RFGRRPVLL---VSLAGAAVDYAIMATAPFLWVLYigrivagitgatgavagayiaDITD 1608

R GRRP+LL + A++ A +TAP +V + G + A +

Sbjct 78 RIGRRPILLAGATAFVIASLGAAWSSTAP-AFVAFRLLQAVGASAMLVATFATVRDVYAN 136

Query 1609 GDERARHFGFMSACFGFGMVAGPVLGGLMGGF 1704

E +G S+ F GP+ G L+G F

Sbjct 137 RPEGVVIYGLFSSMLAFVPALGPIAGVLIGEF 168

> YP_001220605 florfenicol exporter (plasmid) [Aeromonas bestiarum]

Length=404

Score = 38.9 bits (89), Expect = 6e-04, Method: Compositional matrix adjust.

Identities = 45/152 (30%), Positives = 70/152 (46%), Gaps = 14/152 (9%)

Frame = +1

Query 1261 ILSTVALDAVGIGL-IMPVLPGLLRDLVHSNDVTAHYGILLALYALMQFACAPVLGALSD 1437

IL+++A+D I L ++P +PG+L N A + L+LY +M + G LSD

Sbjct 24 ILASLAMD---IYLPVVPAMPGIL------NTTPAMIQLTLSLYMVMLGVGQVIFGPLSD 74

Query 1438 RFGRRPVLL---VSLAGAAVDYAIMATAPFLWVLYigrivagitgatgavagayiaDITD 1608

R GRRP+LL + A++ A +TAP +V + G + A +

Sbjct 75 RIGRRPILLAGATAFVIASLGAAWSSTAP-AFVAFRLLQAVGASAMLVATFATVRDVYAN 133

Query 1609 GDERARHFGFMSACFGFGMVAGPVLGGLMGGF 1704

E +G S+ F GP+ G L+G F

Sbjct 134 RPEGVVIYGLFSSMLAFVPALGPIAGVLIGEF 165

> DAA64637 TPA_inf: florfenicol exporter (plasmid) [Aeromonas bestiarum]

Length=404

Score = 38.9 bits (89), Expect = 6e-04, Method: Compositional matrix adjust.

Identities = 45/152 (30%), Positives = 70/152 (46%), Gaps = 14/152 (9%)

Frame = +1

Query 1261 ILSTVALDAVGIGL-IMPVLPGLLRDLVHSNDVTAHYGILLALYALMQFACAPVLGALSD 1437

IL+++A+D I L ++P +PG+L N A + L+LY +M + G LSD

Sbjct 24 ILASLAMD---IYLPVVPAMPGIL------NTTPAMIQLTLSLYMVMLGVGQVIFGPLSD 74

Query 1438 RFGRRPVLL---VSLAGAAVDYAIMATAPFLWVLYigrivagitgatgavagayiaDITD 1608

R GRRP+LL + A++ A +TAP +V + G + A +

Sbjct 75 RIGRRPILLAGATAFVIASLGAAWSSTAP-AFVAFRLLQAVGASAMLVATFATVRDVYAN 133

Query 1609 GDERARHFGFMSACFGFGMVAGPVLGGLMGGF 1704

E +G S+ F GP+ G L+G F

Sbjct 134 RPEGVVIYGLFSSMLAFVPALGPIAGVLIGEF 165

> ABQ41442 florfenicol exporter (plasmid) [Aeromonas bestiarum]

Length=404

Score = 38.9 bits (89), Expect = 6e-04, Method: Compositional matrix adjust.

Identities = 45/152 (30%), Positives = 70/152 (46%), Gaps = 14/152 (9%)

Frame = +1

Query 1261 ILSTVALDAVGIGL-IMPVLPGLLRDLVHSNDVTAHYGILLALYALMQFACAPVLGALSD 1437

IL+++A+D I L ++P +PG+L N A + L+LY +M + G LSD

Sbjct 24 ILASLAMD---IYLPVVPAMPGIL------NTTPAMIQLTLSLYMVMLGVGQVIFGPLSD 74

Query 1438 RFGRRPVLL---VSLAGAAVDYAIMATAPFLWVLYigrivagitgatgavagayiaDITD 1608

R GRRP+LL + A++ A +TAP +V + G + A +

Sbjct 75 RIGRRPILLAGATAFVIASLGAAWSSTAP-AFVAFRLLQAVGASAMLVATFATVRDVYAN 133

Query 1609 GDERARHFGFMSACFGFGMVAGPVLGGLMGGF 1704

E +G S+ F GP+ G L+G F

Sbjct 134 RPEGVVIYGLFSSMLAFVPALGPIAGVLIGEF 165

> ARS43330 florfenicol-chloramphenicol exporter (plasmid) [Pasteurella

multocida]

Length=404

Score = 38.9 bits (89), Expect = 6e-04, Method: Compositional matrix adjust.

Identities = 45/152 (30%), Positives = 70/152 (46%), Gaps = 14/152 (9%)

Frame = +1

Query 1261 ILSTVALDAVGIGL-IMPVLPGLLRDLVHSNDVTAHYGILLALYALMQFACAPVLGALSD 1437

IL+++A+D I L ++P +PG+L N A + L+LY +M + G LSD

Sbjct 24 ILASLAMD---IYLPVVPAMPGIL------NTTPAMIQLTLSLYMVMLGVGQVIFGPLSD 74

Query 1438 RFGRRPVLL---VSLAGAAVDYAIMATAPFLWVLYigrivagitgatgavagayiaDITD 1608

R GRRP+LL + A++ A +TAP +V + G + A +

Sbjct 75 RIGRRPILLAGATAFVIASLGAAWSSTAP-AFVAFRLLQAVGASAMLVATFATVRDVYAN 133

Query 1609 GDERARHFGFMSACFGFGMVAGPVLGGLMGGF 1704

E +G S+ F GP+ G L+G F

Sbjct 134 RPEGVVIYGLFSSMLAFVPALGPIAGVLIGEF 165

> floR_-_13905643_translation floR: florfenicol/chloramphenicol

resistance protein FloR

Length=404

Score = 38.9 bits (89), Expect = 7e-04, Method: Compositional matrix adjust.

Identities = 45/152 (30%), Positives = 70/152 (46%), Gaps = 14/152 (9%)

Frame = +1

Query 1261 ILSTVALDAVGIGL-IMPVLPGLLRDLVHSNDVTAHYGILLALYALMQFACAPVLGALSD 1437

IL+++A+D I L ++P +PG+L N A + L+LY +M + G LSD

Sbjct 24 ILASLAMD---IYLPVVPAMPGIL------NTTPAMIQLTLSLYMVMLGVGQVIFGPLSD 74

Query 1438 RFGRRPVLL---VSLAGAAVDYAIMATAPFLWVLYigrivagitgatgavagayiaDITD 1608

R GRRP+LL + A++ A +TAP +V + G + A +

Sbjct 75 RIGRRPILLAGATAFVIASLGAAWSSTAP-AFVAFRLLQAVGASAMLVATFATVRDVYAN 133

Query 1609 GDERARHFGFMSACFGFGMVAGPVLGGLMGGF 1704

E +G S+ F GP+ G L+G F

Sbjct 134 RPEGVVIYGLFSSMLAFVPALGPIAGVLIGEF 165

> YP_007349558 florfenicol export (plasmid) [Klebsiella pneumoniae]

Length=404

Score = 38.9 bits (89), Expect = 7e-04, Method: Compositional matrix adjust.

Identities = 45/152 (30%), Positives = 70/152 (46%), Gaps = 14/152 (9%)

Frame = +1

Query 1261 ILSTVALDAVGIGL-IMPVLPGLLRDLVHSNDVTAHYGILLALYALMQFACAPVLGALSD 1437

IL+++A+D I L ++P +PG+L N A + L+LY +M + G LSD

Sbjct 24 ILASLAMD---IYLPVVPAMPGIL------NTTPAMIQLTLSLYMVMLGVGQVIFGPLSD 74

Query 1438 RFGRRPVLL---VSLAGAAVDYAIMATAPFLWVLYigrivagitgatgavagayiaDITD 1608

R GRRP+LL + A++ A +TAP +V + G + A +

Sbjct 75 RIGRRPILLAGATAFVIASLGAAWSSTAP-AFVAFRLLQAVGASAMLVATFATVRDVYAN 133

Query 1609 GDERARHFGFMSACFGFGMVAGPVLGGLMGGF 1704

E +G S+ F GP+ G L+G F

Sbjct 134 RPEGVVIYGLFSSMLAFVPALGPIAGVLIGEF 165

> SCA37875 putative chloramphenicol and florfenicol resistance

protein (CmlA) [Klebsiella pneumoniae]

Length=404

Score = 38.9 bits (89), Expect = 7e-04, Method: Compositional matrix adjust.

Identities = 45/152 (30%), Positives = 70/152 (46%), Gaps = 14/152 (9%)

Frame = +1

Query 1261 ILSTVALDAVGIGL-IMPVLPGLLRDLVHSNDVTAHYGILLALYALMQFACAPVLGALSD 1437

IL+++A+D I L ++P +PG+L N A + L+LY +M + G LSD

Sbjct 24 ILASLAMD---IYLPVVPAMPGIL------NTTPAMIQLTLSLYMVMLGVGQVIFGPLSD 74

Query 1438 RFGRRPVLL---VSLAGAAVDYAIMATAPFLWVLYigrivagitgatgavagayiaDITD 1608

R GRRP+LL + A++ A +TAP +V + G + A +

Sbjct 75 RIGRRPILLAGATAFVIASLGAAWSSTAP-AFVAFRLLQAVGASAMLVATFATVRDVYAN 133

Query 1609 GDERARHFGFMSACFGFGMVAGPVLGGLMGGF 1704

E +G S+ F GP+ G L+G F

Sbjct 134 RPEGVVIYGLFSSMLAFVPALGPIAGVLIGEF 165

> SCA36976 putative chloramphenicol and florfenicol resistance

protein (CmlA) [Klebsiella pneumoniae]

Length=404

Score = 38.9 bits (89), Expect = 7e-04, Method: Compositional matrix adjust.

Identities = 45/152 (30%), Positives = 70/152 (46%), Gaps = 14/152 (9%)

Frame = +1

Query 1261 ILSTVALDAVGIGL-IMPVLPGLLRDLVHSNDVTAHYGILLALYALMQFACAPVLGALSD 1437

IL+++A+D I L ++P +PG+L N A + L+LY +M + G LSD

Sbjct 24 ILASLAMD---IYLPVVPAMPGIL------NTTPAMIQLTLSLYMVMLGVGQVIFGPLSD 74

Query 1438 RFGRRPVLL---VSLAGAAVDYAIMATAPFLWVLYigrivagitgatgavagayiaDITD 1608

R GRRP+LL + A++ A +TAP +V + G + A +

Sbjct 75 RIGRRPILLAGATAFVIASLGAAWSSTAP-AFVAFRLLQAVGASAMLVATFATVRDVYAN 133

Query 1609 GDERARHFGFMSACFGFGMVAGPVLGGLMGGF 1704

E +G S+ F GP+ G L+G F

Sbjct 134 RPEGVVIYGLFSSMLAFVPALGPIAGVLIGEF 165

> SCA29482 putative chloramphenicol and florfenicol resistance

protein (CmlA) [Klebsiella pneumoniae]

Length=404

Score = 38.9 bits (89), Expect = 7e-04, Method: Compositional matrix adjust.

Identities = 45/152 (30%), Positives = 70/152 (46%), Gaps = 14/152 (9%)

Frame = +1

Query 1261 ILSTVALDAVGIGL-IMPVLPGLLRDLVHSNDVTAHYGILLALYALMQFACAPVLGALSD 1437

IL+++A+D I L ++P +PG+L N A + L+LY +M + G LSD

Sbjct 24 ILASLAMD---IYLPVVPAMPGIL------NTTPAMIQLTLSLYMVMLGVGQVIFGPLSD 74

Query 1438 RFGRRPVLL---VSLAGAAVDYAIMATAPFLWVLYigrivagitgatgavagayiaDITD 1608

R GRRP+LL + A++ A +TAP +V + G + A +

Sbjct 75 RIGRRPILLAGATAFVIASLGAAWSSTAP-AFVAFRLLQAVGASAMLVATFATVRDVYAN 133

Query 1609 GDERARHFGFMSACFGFGMVAGPVLGGLMGGF 1704

E +G S+ F GP+ G L+G F

Sbjct 134 RPEGVVIYGLFSSMLAFVPALGPIAGVLIGEF 165

> SCA27592 putative chloramphenicol and florfenicol resistance

protein (CmlA) [Klebsiella pneumoniae]

Length=404

Score = 38.9 bits (89), Expect = 7e-04, Method: Compositional matrix adjust.

Identities = 45/152 (30%), Positives = 70/152 (46%), Gaps = 14/152 (9%)

Frame = +1

Query 1261 ILSTVALDAVGIGL-IMPVLPGLLRDLVHSNDVTAHYGILLALYALMQFACAPVLGALSD 1437

IL+++A+D I L ++P +PG+L N A + L+LY +M + G LSD

Sbjct 24 ILASLAMD---IYLPVVPAMPGIL------NTTPAMIQLTLSLYMVMLGVGQVIFGPLSD 74

Query 1438 RFGRRPVLL---VSLAGAAVDYAIMATAPFLWVLYigrivagitgatgavagayiaDITD 1608

R GRRP+LL + A++ A +TAP +V + G + A +

Sbjct 75 RIGRRPILLAGATAFVIASLGAAWSSTAP-AFVAFRLLQAVGASAMLVATFATVRDVYAN 133

Query 1609 GDERARHFGFMSACFGFGMVAGPVLGGLMGGF 1704

E +G S+ F GP+ G L+G F

Sbjct 134 RPEGVVIYGLFSSMLAFVPALGPIAGVLIGEF 165

> SCA25014 putative chloramphenicol and florfenicol resistance

protein (CmlA) [Klebsiella pneumoniae]

Length=404

Score = 38.9 bits (89), Expect = 7e-04, Method: Compositional matrix adjust.

Identities = 45/152 (30%), Positives = 70/152 (46%), Gaps = 14/152 (9%)

Frame = +1

Query 1261 ILSTVALDAVGIGL-IMPVLPGLLRDLVHSNDVTAHYGILLALYALMQFACAPVLGALSD 1437

IL+++A+D I L ++P +PG+L N A + L+LY +M + G LSD

Sbjct 24 ILASLAMD---IYLPVVPAMPGIL------NTTPAMIQLTLSLYMVMLGVGQVIFGPLSD 74

Query 1438 RFGRRPVLL---VSLAGAAVDYAIMATAPFLWVLYigrivagitgatgavagayiaDITD 1608

R GRRP+LL + A++ A +TAP +V + G + A +

Sbjct 75 RIGRRPILLAGATAFVIASLGAAWSSTAP-AFVAFRLLQAVGASAMLVATFATVRDVYAN 133

Query 1609 GDERARHFGFMSACFGFGMVAGPVLGGLMGGF 1704

E +G S+ F GP+ G L+G F

Sbjct 134 RPEGVVIYGLFSSMLAFVPALGPIAGVLIGEF 165

> SCA21793 putative chloramphenicol and florfenicol resistance

protein (CmlA) [Klebsiella pneumoniae]

Length=404

Score = 38.9 bits (89), Expect = 7e-04, Method: Compositional matrix adjust.

Identities = 45/152 (30%), Positives = 70/152 (46%), Gaps = 14/152 (9%)

Frame = +1

Query 1261 ILSTVALDAVGIGL-IMPVLPGLLRDLVHSNDVTAHYGILLALYALMQFACAPVLGALSD 1437

IL+++A+D I L ++P +PG+L N A + L+LY +M + G LSD

Sbjct 24 ILASLAMD---IYLPVVPAMPGIL------NTTPAMIQLTLSLYMVMLGVGQVIFGPLSD 74

Query 1438 RFGRRPVLL---VSLAGAAVDYAIMATAPFLWVLYigrivagitgatgavagayiaDITD 1608

R GRRP+LL + A++ A +TAP +V + G + A +

Sbjct 75 RIGRRPILLAGATAFVIASLGAAWSSTAP-AFVAFRLLQAVGASAMLVATFATVRDVYAN 133

Query 1609 GDERARHFGFMSACFGFGMVAGPVLGGLMGGF 1704

E +G S+ F GP+ G L+G F

Sbjct 134 RPEGVVIYGLFSSMLAFVPALGPIAGVLIGEF 165

> SCA10658 putative chloramphenicol and florfenicol resistance

protein (CmlA) [Klebsiella pneumoniae]

Length=404

Score = 38.9 bits (89), Expect = 7e-04, Method: Compositional matrix adjust.

Identities = 45/152 (30%), Positives = 70/152 (46%), Gaps = 14/152 (9%)

Frame = +1

Query 1261 ILSTVALDAVGIGL-IMPVLPGLLRDLVHSNDVTAHYGILLALYALMQFACAPVLGALSD 1437

IL+++A+D I L ++P +PG+L N A + L+LY +M + G LSD

Sbjct 24 ILASLAMD---IYLPVVPAMPGIL------NTTPAMIQLTLSLYMVMLGVGQVIFGPLSD 74

Query 1438 RFGRRPVLL---VSLAGAAVDYAIMATAPFLWVLYigrivagitgatgavagayiaDITD 1608

R GRRP+LL + A++ A +TAP +V + G + A +

Sbjct 75 RIGRRPILLAGATAFVIASLGAAWSSTAP-AFVAFRLLQAVGASAMLVATFATVRDVYAN 133

Query 1609 GDERARHFGFMSACFGFGMVAGPVLGGLMGGF 1704

E +G S+ F GP+ G L+G F

Sbjct 134 RPEGVVIYGLFSSMLAFVPALGPIAGVLIGEF 165

> SBY74064 putative chloramphenicol and florfenicol resistance

protein (CmlA) [Klebsiella pneumoniae]

Length=404

Score = 38.9 bits (89), Expect = 7e-04, Method: Compositional matrix adjust.

Identities = 45/152 (30%), Positives = 70/152 (46%), Gaps = 14/152 (9%)

Frame = +1

Query 1261 ILSTVALDAVGIGL-IMPVLPGLLRDLVHSNDVTAHYGILLALYALMQFACAPVLGALSD 1437

IL+++A+D I L ++P +PG+L N A + L+LY +M + G LSD

Sbjct 24 ILASLAMD---IYLPVVPAMPGIL------NTTPAMIQLTLSLYMVMLGVGQVIFGPLSD 74

Query 1438 RFGRRPVLL---VSLAGAAVDYAIMATAPFLWVLYigrivagitgatgavagayiaDITD 1608

R GRRP+LL + A++ A +TAP +V + G + A +

Sbjct 75 RIGRRPILLAGATAFVIASLGAAWSSTAP-AFVAFRLLQAVGASAMLVATFATVRDVYAN 133

Query 1609 GDERARHFGFMSACFGFGMVAGPVLGGLMGGF 1704

E +G S+ F GP+ G L+G F

Sbjct 134 RPEGVVIYGLFSSMLAFVPALGPIAGVLIGEF 165

> OSJ79165 florfenicol export [Salmonella enterica subsp. enterica

serovar Newport str. SHSN010]

Length=404

Score = 38.9 bits (89), Expect = 7e-04, Method: Compositional matrix adjust.

Identities = 45/152 (30%), Positives = 70/152 (46%), Gaps = 14/152 (9%)

Frame = +1

Query 1261 ILSTVALDAVGIGL-IMPVLPGLLRDLVHSNDVTAHYGILLALYALMQFACAPVLGALSD 1437

IL+++A+D I L ++P +PG+L N A + L+LY +M + G LSD

Sbjct 24 ILASLAMD---IYLPVVPAMPGIL------NTTPAMIQLTLSLYMVMLGVGQVIFGPLSD 74

Query 1438 RFGRRPVLL---VSLAGAAVDYAIMATAPFLWVLYigrivagitgatgavagayiaDITD 1608

R GRRP+LL + A++ A +TAP +V + G + A +

Sbjct 75 RIGRRPILLAGATAFVIASLGAAWSSTAP-AFVAFRLLQAVGASAMLVATFATVRDVYAN 133

Query 1609 GDERARHFGFMSACFGFGMVAGPVLGGLMGGF 1704

E +G S+ F GP+ G L+G F

Sbjct 134 RPEGVVIYGLFSSMLAFVPALGPIAGVLIGEF 165

> OAF88709 Florfenicol export protein [Escherichia coli PCN079]

Length=404

Score = 38.9 bits (89), Expect = 7e-04, Method: Compositional matrix adjust.

Identities = 45/152 (30%), Positives = 70/152 (46%), Gaps = 14/152 (9%)

Frame = +1

Query 1261 ILSTVALDAVGIGL-IMPVLPGLLRDLVHSNDVTAHYGILLALYALMQFACAPVLGALSD 1437

IL+++A+D I L ++P +PG+L N A + L+LY +M + G LSD

Sbjct 24 ILASLAMD---IYLPVVPAMPGIL------NTTPAMIQLTLSLYMVMLGVGQVIFGPLSD 74

Query 1438 RFGRRPVLL---VSLAGAAVDYAIMATAPFLWVLYigrivagitgatgavagayiaDITD 1608

R GRRP+LL + A++ A +TAP +V + G + A +

Sbjct 75 RIGRRPILLAGATAFVIASLGAAWSSTAP-AFVAFRLLQAVGASAMLVATFATVRDVYAN 133

Query 1609 GDERARHFGFMSACFGFGMVAGPVLGGLMGGF 1704

E +G S+ F GP+ G L+G F

Sbjct 134 RPEGVVIYGLFSSMLAFVPALGPIAGVLIGEF 165

> EKF76275 Florfenicol export protein [Klebsiella pneumoniae subsp.

pneumoniae KpQ3]

Length=404

Score = 38.9 bits (89), Expect = 7e-04, Method: Compositional matrix adjust.

Identities = 45/152 (30%), Positives = 70/152 (46%), Gaps = 14/152 (9%)

Frame = +1

Query 1261 ILSTVALDAVGIGL-IMPVLPGLLRDLVHSNDVTAHYGILLALYALMQFACAPVLGALSD 1437

IL+++A+D I L ++P +PG+L N A + L+LY +M + G LSD

Sbjct 24 ILASLAMD---IYLPVVPAMPGIL------NTTPAMIQLTLSLYMVMLGVGQVIFGPLSD 74

Query 1438 RFGRRPVLL---VSLAGAAVDYAIMATAPFLWVLYigrivagitgatgavagayiaDITD 1608

R GRRP+LL + A++ A +TAP +V + G + A +

Sbjct 75 RIGRRPILLAGATAFVIASLGAAWSSTAP-AFVAFRLLQAVGASAMLVATFATVRDVYAN 133

Query 1609 GDERARHFGFMSACFGFGMVAGPVLGGLMGGF 1704

E +G S+ F GP+ G L+G F

Sbjct 134 RPEGVVIYGLFSSMLAFVPALGPIAGVLIGEF 165

> EJE85093 florfenicol exporter [Escherichia coli O111:H11 str.

CVM9455]

Length=404

Score = 38.9 bits (89), Expect = 7e-04, Method: Compositional matrix adjust.

Identities = 45/152 (30%), Positives = 70/152 (46%), Gaps = 14/152 (9%)

Frame = +1

Query 1261 ILSTVALDAVGIGL-IMPVLPGLLRDLVHSNDVTAHYGILLALYALMQFACAPVLGALSD 1437

IL+++A+D I L ++P +PG+L N A + L+LY +M + G LSD

Sbjct 24 ILASLAMD---IYLPVVPAMPGIL------NTTPAMIQLTLSLYMVMLGVGQVIFGPLSD 74

Query 1438 RFGRRPVLL---VSLAGAAVDYAIMATAPFLWVLYigrivagitgatgavagayiaDITD 1608

R GRRP+LL + A++ A +TAP +V + G + A +

Sbjct 75 RIGRRPILLAGATAFVIASLGAAWSSTAP-AFVAFRLLQAVGASAMLVATFATVRDVYAN 133

Query 1609 GDERARHFGFMSACFGFGMVAGPVLGGLMGGF 1704

E +G S+ F GP+ G L+G F

Sbjct 134 RPEGVVIYGLFSSMLAFVPALGPIAGVLIGEF 165

> CCN79991 florfenicol export (plasmid) [Klebsiella pneumoniae]

Length=404

Score = 38.9 bits (89), Expect = 7e-04, Method: Compositional matrix adjust.

Identities = 45/152 (30%), Positives = 70/152 (46%), Gaps = 14/152 (9%)

Frame = +1

Query 1261 ILSTVALDAVGIGL-IMPVLPGLLRDLVHSNDVTAHYGILLALYALMQFACAPVLGALSD 1437

IL+++A+D I L ++P +PG+L N A + L+LY +M + G LSD

Sbjct 24 ILASLAMD---IYLPVVPAMPGIL------NTTPAMIQLTLSLYMVMLGVGQVIFGPLSD 74

Query 1438 RFGRRPVLL---VSLAGAAVDYAIMATAPFLWVLYigrivagitgatgavagayiaDITD 1608

R GRRP+LL + A++ A +TAP +V + G + A +

Sbjct 75 RIGRRPILLAGATAFVIASLGAAWSSTAP-AFVAFRLLQAVGASAMLVATFATVRDVYAN 133

Query 1609 GDERARHFGFMSACFGFGMVAGPVLGGLMGGF 1704

E +G S+ F GP+ G L+G F

Sbjct 134 RPEGVVIYGLFSSMLAFVPALGPIAGVLIGEF 165

> ASF20288 florfenicol exporter [Proteus mirabilis]

Length=404

Score = 38.9 bits (89), Expect = 7e-04, Method: Compositional matrix adjust.

Identities = 45/152 (30%), Positives = 70/152 (46%), Gaps = 14/152 (9%)

Frame = +1

Query 1261 ILSTVALDAVGIGL-IMPVLPGLLRDLVHSNDVTAHYGILLALYALMQFACAPVLGALSD 1437

IL+++A+D I L ++P +PG+L N A + L+LY +M + G LSD

Sbjct 24 ILASLAMD---IYLPVVPAMPGIL------NTTPAMIQLTLSLYMVMLGVGQVIFGPLSD 74

Query 1438 RFGRRPVLL---VSLAGAAVDYAIMATAPFLWVLYigrivagitgatgavagayiaDITD 1608

R GRRP+LL + A++ A +TAP +V + G + A +

Sbjct 75 RIGRRPILLAGATAFVIASLGAAWSSTAP-AFVAFRLLQAVGASAMLVATFATVRDVYAN 133

Query 1609 GDERARHFGFMSACFGFGMVAGPVLGGLMGGF 1704

E +G S+ F GP+ G L+G F

Sbjct 134 RPEGVVIYGLFSSMLAFVPALGPIAGVLIGEF 165

> ASF20233 florfenicol exporter [Proteus mirabilis]

Length=404

Score = 38.9 bits (89), Expect = 7e-04, Method: Compositional matrix adjust.

Identities = 45/152 (30%), Positives = 70/152 (46%), Gaps = 14/152 (9%)

Frame = +1

Query 1261 ILSTVALDAVGIGL-IMPVLPGLLRDLVHSNDVTAHYGILLALYALMQFACAPVLGALSD 1437

IL+++A+D I L ++P +PG+L N A + L+LY +M + G LSD

Sbjct 24 ILASLAMD---IYLPVVPAMPGIL------NTTPAMIQLTLSLYMVMLGVGQVIFGPLSD 74

Query 1438 RFGRRPVLL---VSLAGAAVDYAIMATAPFLWVLYigrivagitgatgavagayiaDITD 1608

R GRRP+LL + A++ A +TAP +V + G + A +

Sbjct 75 RIGRRPILLAGATAFVIASLGAAWSSTAP-AFVAFRLLQAVGASAMLVATFATVRDVYAN 133

Query 1609 GDERARHFGFMSACFGFGMVAGPVLGGLMGGF 1704

E +G S+ F GP+ G L+G F

Sbjct 134 RPEGVVIYGLFSSMLAFVPALGPIAGVLIGEF 165

> AQZ19169 FloR (plasmid) [[Haemophilus] parasuis]

Length=404

Score = 38.9 bits (89), Expect = 7e-04, Method: Compositional matrix adjust.

Identities = 45/152 (30%), Positives = 70/152 (46%), Gaps = 14/152 (9%)

Frame = +1

Query 1261 ILSTVALDAVGIGL-IMPVLPGLLRDLVHSNDVTAHYGILLALYALMQFACAPVLGALSD 1437

IL+++A+D I L ++P +PG+L N A + L+LY +M + G LSD

Sbjct 24 ILASLAMD---IYLPVVPAMPGIL------NTTPAMIQLTLSLYMVMLGVGQVIFGPLSD 74

Query 1438 RFGRRPVLL---VSLAGAAVDYAIMATAPFLWVLYigrivagitgatgavagayiaDITD 1608

R GRRP+LL + A++ A +TAP +V + G + A +

Sbjct 75 RIGRRPILLAGATAFVIASLGAAWSSTAP-AFVAFRLLQAVGASAMLVATFATVRDVYAN 133

Query 1609 GDERARHFGFMSACFGFGMVAGPVLGGLMGGF 1704

E +G S+ F GP+ G L+G F

Sbjct 134 RPEGVVIYGLFSSMLAFVPALGPIAGVLIGEF 165

> ANA09614 florfenicol MFS transporter, FloR (plasmid) [Escherichia

coli]

Length=404

Score = 38.9 bits (89), Expect = 7e-04, Method: Compositional matrix adjust.

Identities = 45/152 (30%), Positives = 70/152 (46%), Gaps = 14/152 (9%)

Frame = +1

Query 1261 ILSTVALDAVGIGL-IMPVLPGLLRDLVHSNDVTAHYGILLALYALMQFACAPVLGALSD 1437

IL+++A+D I L ++P +PG+L N A + L+LY +M + G LSD

Sbjct 24 ILASLAMD---IYLPVVPAMPGIL------NTTPAMIQLTLSLYMVMLGVGQVIFGPLSD 74

Query 1438 RFGRRPVLL---VSLAGAAVDYAIMATAPFLWVLYigrivagitgatgavagayiaDITD 1608

R GRRP+LL + A++ A +TAP +V + G + A +

Sbjct 75 RIGRRPILLAGATAFVIASLGAAWSSTAP-AFVAFRLLQAVGASAMLVATFATVRDVYAN 133

Query 1609 GDERARHFGFMSACFGFGMVAGPVLGGLMGGF 1704

E +G S+ F GP+ G L+G F

Sbjct 134 RPEGVVIYGLFSSMLAFVPALGPIAGVLIGEF 165

> AMP42434 florfenicol resistance protein [uncultured bacterium

IN-12]

Length=404

Score = 38.9 bits (89), Expect = 7e-04, Method: Compositional matrix adjust.

Identities = 45/152 (30%), Positives = 70/152 (46%), Gaps = 14/152 (9%)

Frame = +1

Query 1261 ILSTVALDAVGIGL-IMPVLPGLLRDLVHSNDVTAHYGILLALYALMQFACAPVLGALSD 1437

IL+++A+D I L ++P +PG+L N A + L+LY +M + G LSD

Sbjct 24 ILASLAMD---IYLPVVPAMPGIL------NTTPAMIQLTLSLYMVMLGVGQVIFGPLSD 74

Query 1438 RFGRRPVLL---VSLAGAAVDYAIMATAPFLWVLYigrivagitgatgavagayiaDITD 1608

R GRRP+LL + A++ A +TAP +V + G + A +

Sbjct 75 RIGRRPILLAGATAFVIASLGAAWSSTAP-AFVAFRLLQAVGASAMLVATFATVRDVYAN 133

Query 1609 GDERARHFGFMSACFGFGMVAGPVLGGLMGGF 1704

E +G S+ F GP+ G L+G F

Sbjct 134 RPEGVVIYGLFSSMLAFVPALGPIAGVLIGEF 165

> AMP42401 florfenicol resistance protein [uncultured bacterium

IN-11]

Length=404

Score = 38.9 bits (89), Expect = 7e-04, Method: Compositional matrix adjust.

Identities = 45/152 (30%), Positives = 70/152 (46%), Gaps = 14/152 (9%)

Frame = +1

Query 1261 ILSTVALDAVGIGL-IMPVLPGLLRDLVHSNDVTAHYGILLALYALMQFACAPVLGALSD 1437

IL+++A+D I L ++P +PG+L N A + L+LY +M + G LSD

Sbjct 24 ILASLAMD---IYLPVVPAMPGIL------NTTPAMIQLTLSLYMVMLGVGQVIFGPLSD 74

Query 1438 RFGRRPVLL---VSLAGAAVDYAIMATAPFLWVLYigrivagitgatgavagayiaDITD 1608

R GRRP+LL + A++ A +TAP +V + G + A +

Sbjct 75 RIGRRPILLAGATAFVIASLGAAWSSTAP-AFVAFRLLQAVGASAMLVATFATVRDVYAN 133

Query 1609 GDERARHFGFMSACFGFGMVAGPVLGGLMGGF 1704

E +G S+ F GP+ G L+G F

Sbjct 134 RPEGVVIYGLFSSMLAFVPALGPIAGVLIGEF 165

> ALN43627 FloR (plasmid) [Actinobacillus pleuropneumoniae]

Length=404

Score = 38.9 bits (89), Expect = 7e-04, Method: Compositional matrix adjust.

Identities = 45/152 (30%), Positives = 70/152 (46%), Gaps = 14/152 (9%)

Frame = +1

Query 1261 ILSTVALDAVGIGL-IMPVLPGLLRDLVHSNDVTAHYGILLALYALMQFACAPVLGALSD 1437

IL+++A+D I L ++P +PG+L N A + L+LY +M + G LSD

Sbjct 24 ILASLAMD---IYLPVVPAMPGIL------NTTPAMIQLTLSLYMVMLGVGQVIFGPLSD 74

Query 1438 RFGRRPVLL---VSLAGAAVDYAIMATAPFLWVLYigrivagitgatgavagayiaDITD 1608

R GRRP+LL + A++ A +TAP +V + G + A +

Sbjct 75 RIGRRPILLAGATAFVIASLGAAWSSTAP-AFVAFRLLQAVGASAMLVATFATVRDVYAN 133

Query 1609 GDERARHFGFMSACFGFGMVAGPVLGGLMGGF 1704

E +G S+ F GP+ G L+G F

Sbjct 134 RPEGVVIYGLFSSMLAFVPALGPIAGVLIGEF 165

> AKM21207 FloR (plasmid) [Actinobacillus pleuropneumoniae]

Length=404

Score = 38.9 bits (89), Expect = 7e-04, Method: Compositional matrix adjust.

Identities = 45/152 (30%), Positives = 70/152 (46%), Gaps = 14/152 (9%)

Frame = +1

Query 1261 ILSTVALDAVGIGL-IMPVLPGLLRDLVHSNDVTAHYGILLALYALMQFACAPVLGALSD 1437

IL+++A+D I L ++P +PG+L N A + L+LY +M + G LSD

Sbjct 24 ILASLAMD---IYLPVVPAMPGIL------NTTPAMIQLTLSLYMVMLGVGQVIFGPLSD 74

Query 1438 RFGRRPVLL---VSLAGAAVDYAIMATAPFLWVLYigrivagitgatgavagayiaDITD 1608

R GRRP+LL + A++ A +TAP +V + G + A +

Sbjct 75 RIGRRPILLAGATAFVIASLGAAWSSTAP-AFVAFRLLQAVGASAMLVATFATVRDVYAN 133

Query 1609 GDERARHFGFMSACFGFGMVAGPVLGGLMGGF 1704

E +G S+ F GP+ G L+G F

Sbjct 134 RPEGVVIYGLFSSMLAFVPALGPIAGVLIGEF 165

> AKG90161 floR (plasmid) [Salmonella enterica subsp. enterica

serovar Typhimurium]

Length=404

Score = 38.9 bits (89), Expect = 7e-04, Method: Compositional matrix adjust.

Identities = 45/152 (30%), Positives = 70/152 (46%), Gaps = 14/152 (9%)

Frame = +1

Query 1261 ILSTVALDAVGIGL-IMPVLPGLLRDLVHSNDVTAHYGILLALYALMQFACAPVLGALSD 1437

IL+++A+D I L ++P +PG+L N A + L+LY +M + G LSD

Sbjct 24 ILASLAMD---IYLPVVPAMPGIL------NTTPAMIQLTLSLYMVMLGVGQVIFGPLSD 74

Query 1438 RFGRRPVLL---VSLAGAAVDYAIMATAPFLWVLYigrivagitgatgavagayiaDITD 1608

R GRRP+LL + A++ A +TAP +V + G + A +

Sbjct 75 RIGRRPILLAGATAFVIASLGAAWSSTAP-AFVAFRLLQAVGASAMLVATFATVRDVYAN 133

Query 1609 GDERARHFGFMSACFGFGMVAGPVLGGLMGGF 1704

E +G S+ F GP+ G L+G F

Sbjct 134 RPEGVVIYGLFSSMLAFVPALGPIAGVLIGEF 165

> ACJ64205 florfenicol export protein (plasmid) [Edwardsiella ictaluri]

Length=404

Score = 38.9 bits (89), Expect = 7e-04, Method: Compositional matrix adjust.

Identities = 45/152 (30%), Positives = 70/152 (46%), Gaps = 14/152 (9%)

Frame = +1

Query 1261 ILSTVALDAVGIGL-IMPVLPGLLRDLVHSNDVTAHYGILLALYALMQFACAPVLGALSD 1437

IL+++A+D I L ++P +PG+L N A + L+LY +M + G LSD

Sbjct 24 ILASLAMD---IYLPVVPAMPGIL------NTTPAMIQLTLSLYMVMLGVGQVIFGPLSD 74

Query 1438 RFGRRPVLL---VSLAGAAVDYAIMATAPFLWVLYigrivagitgatgavagayiaDITD 1608

R GRRP+LL + A++ A +TAP +V + G + A +

Sbjct 75 RIGRRPILLAGATAFVIASLGAAWSSTAP-AFVAFRLLQAVGASAMLVATFATVRDVYAN 133

Query 1609 GDERARHFGFMSACFGFGMVAGPVLGGLMGGF 1704

E +G S+ F GP+ G L+G F

Sbjct 134 RPEGVVIYGLFSSMLAFVPALGPIAGVLIGEF 165

> ABO10439 florfenicol export protein [Escherichia coli]

Length=404

Score = 38.9 bits (89), Expect = 7e-04, Method: Compositional matrix adjust.

Identities = 45/152 (30%), Positives = 70/152 (46%), Gaps = 14/152 (9%)

Frame = +1

Query 1261 ILSTVALDAVGIGL-IMPVLPGLLRDLVHSNDVTAHYGILLALYALMQFACAPVLGALSD 1437

IL+++A+D I L ++P +PG+L N A + L+LY +M + G LSD

Sbjct 24 ILASLAMD---IYLPVVPAMPGIL------NTTPAMIQLTLSLYMVMLGVGQVIFGPLSD 74

Query 1438 RFGRRPVLL---VSLAGAAVDYAIMATAPFLWVLYigrivagitgatgavagayiaDITD 1608

R GRRP+LL + A++ A +TAP +V + G + A +

Sbjct 75 RIGRRPILLAGATAFVIASLGAAWSSTAP-AFVAFRLLQAVGASAMLVATFATVRDVYAN 133

Query 1609 GDERARHFGFMSACFGFGMVAGPVLGGLMGGF 1704

E +G S+ F GP+ G L+G F

Sbjct 134 RPEGVVIYGLFSSMLAFVPALGPIAGTLIGEF 165

> ALJ52406 FloR (plasmid) [Escherichia coli]

Length=404

Score = 38.5 bits (88), Expect = 7e-04, Method: Compositional matrix adjust.

Identities = 45/152 (30%), Positives = 70/152 (46%), Gaps = 14/152 (9%)

Frame = +1

Query 1261 ILSTVALDAVGIGL-IMPVLPGLLRDLVHSNDVTAHYGILLALYALMQFACAPVLGALSD 1437

IL+++A+D I L ++P +PG+L N A + L+LY +M + G LSD

Sbjct 24 ILASLAMD---IYLPVVPAMPGIL------NTTPAMIQLTLSLYMVMLGVGQVIFGPLSD 74

Query 1438 RFGRRPVLL---VSLAGAAVDYAIMATAPFLWVLYigrivagitgatgavagayiaDITD 1608

R GRRP+LL + A++ A +TAP +V + G + A +

Sbjct 75 RIGRRPILLAGATAFVIASLGAAWSSTAP-AFVAFRLLQAVGASAMHVATFATVRDVYAN 133

Query 1609 GDERARHFGFMSACFGFGMVAGPVLGGLMGGF 1704

E +G S+ F GP+ G L+G F

Sbjct 134 RPEGVVIYGLFSSMLAFVPALGPIAGVLIGEF 165

> CAJ30495 chloramphenicol/florfenicol exporter (plasmid) [Staphylococcus

aureus]

Length=475

Score = 38.5 bits (88), Expect = 8e-04, Method: Compositional matrix adjust.

Identities = 44/173 (25%), Positives = 85/173 (49%), Gaps = 5/173 (3%)

Frame = +1

Query 1192 RVQSDQRRLVRRPDVKPNRPLIVILS-TVALDAVGIGLIMPVLPGLLRDLVHSNDVTAHY 1368

+ S + +++ R L+++LS +V + A+ L+ PVLP + +DL S +

Sbjct 2 KKDSKSKEMIQSEKRGSTRLLMMVLSLSVLVGAITADLVNPVLPLISKDLEASKSQVSW- 60

Query 1369 GILLALYALMQFACAPVLGALSDRFGRRPVLLVSLAGAAVDYAIMATAPFLWVLYigriv 1548

+++ AL+ P+ G +SD F R + + ++ A + A AP L +L +GR+V

Sbjct 61 --IVSGIALVLAIGVPIYGRISDFFELRKLYIFAIMILASGSLLCAIAPNLPLLVLGRMV 118

Query 1549 agitgatg-avagayiaDITDGDERARHFGFMSACFGFGMVAGPVLGGLMGGF 1704

G + ++ I+ + +R G ++ G G AGP+ GG++G +

Sbjct 119 QGAGMSAIPVLSIIAISKVFPQGKRGGALGIIAGSIGVGTAAGPIFGGVVGQY 171

> CAG29651 florfenicol-chloramphenicol exporter [Staphylococcus

lentus]

Length=475

Score = 38.5 bits (88), Expect = 8e-04, Method: Compositional matrix adjust.

Identities = 44/173 (25%), Positives = 85/173 (49%), Gaps = 5/173 (3%)

Frame = +1

Query 1192 RVQSDQRRLVRRPDVKPNRPLIVILS-TVALDAVGIGLIMPVLPGLLRDLVHSNDVTAHY 1368

+ S + +++ R L+++LS +V + A+ L+ PVLP + +DL S +

Sbjct 2 KKDSKSKEMIQSEKRGSTRLLMMVLSLSVLVGAITADLVNPVLPLISKDLEASKSQVSW- 60

Query 1369 GILLALYALMQFACAPVLGALSDRFGRRPVLLVSLAGAAVDYAIMATAPFLWVLYigriv 1548

+++ AL+ P+ G +SD F R + + ++ A + A AP L +L +GR+V

Sbjct 61 --IVSGIALVLAIGVPIYGRISDFFELRKLYIFAIMILASGSLLCAIAPNLPLLVLGRMV 118

Query 1549 agitgatg-avagayiaDITDGDERARHFGFMSACFGFGMVAGPVLGGLMGGF 1704

G + ++ I+ + +R G ++ G G AGP+ GG++G +

Sbjct 119 QGAGMSAIPVLSIIAISKVFPQGKRGGALGIIAGSIGVGTAAGPIFGGVVGQY 171

> CAD70268 florfenicol-chloramphenicol exporter (plasmid) [Staphylococcus

lentus]

Length=475

Score = 38.5 bits (88), Expect = 8e-04, Method: Compositional matrix adjust.

Identities = 44/173 (25%), Positives = 85/173 (49%), Gaps = 5/173 (3%)

Frame = +1

Query 1192 RVQSDQRRLVRRPDVKPNRPLIVILS-TVALDAVGIGLIMPVLPGLLRDLVHSNDVTAHY 1368

+ S + +++ R L+++LS +V + A+ L+ PVLP + +DL S +

Sbjct 2 KKDSKSKEMIQSEKRGSTRLLMMVLSLSVLVGAITADLVNPVLPLISKDLEASKSQVSW- 60

Query 1369 GILLALYALMQFACAPVLGALSDRFGRRPVLLVSLAGAAVDYAIMATAPFLWVLYigriv 1548

+++ AL+ P+ G +SD F R + + ++ A + A AP L +L +GR+V

Sbjct 61 --IVSGIALVLAIGVPIYGRISDFFELRKLYIFAIMILASGSLLCAIAPNLPLLVLGRMV 118

Query 1549 agitgatg-avagayiaDITDGDERARHFGFMSACFGFGMVAGPVLGGLMGGF 1704

G + ++ I+ + +R G ++ G G AGP+ GG++G +

Sbjct 119 QGAGMSAIPVLSIIAISKVFPQGKRGGALGIIAGSIGVGTAAGPIFGGVVGQY 171

> AMN16506 FexA (plasmid) [Staphylococcus aureus subsp. aureus]

Length=475

Score = 38.5 bits (88), Expect = 8e-04, Method: Compositional matrix adjust.

Identities = 44/173 (25%), Positives = 85/173 (49%), Gaps = 5/173 (3%)

Frame = +1

Query 1192 RVQSDQRRLVRRPDVKPNRPLIVILS-TVALDAVGIGLIMPVLPGLLRDLVHSNDVTAHY 1368

+ S + +++ R L+++LS +V + A+ L+ PVLP + +DL S +

Sbjct 2 KKDSKSKEMIQSEKRGSTRLLMMVLSLSVLVGAITADLVNPVLPLISKDLEASKSQVSW- 60

Query 1369 GILLALYALMQFACAPVLGALSDRFGRRPVLLVSLAGAAVDYAIMATAPFLWVLYigriv 1548

+++ AL+ P+ G +SD F R + + ++ A + A AP L +L +GR+V

Sbjct 61 --IVSGIALVLAIGVPIYGRISDFFELRKLYIFAIMILASGSLLCAIAPNLPLLVLGRMV 118

Query 1549 agitgatg-avagayiaDITDGDERARHFGFMSACFGFGMVAGPVLGGLMGGF 1704

G + ++ I+ + +R G ++ G G AGP+ GG++G +

Sbjct 119 QGAGMSAIPVLSIIAISKVFPQGKRGGALGIIAGSIGVGTAAGPIFGGVVGQY 171

> AGH12818 chloramphenicol/florfenicol exporter (plasmid) [Staphylococcus

haemolyticus]

Length=475

Score = 38.5 bits (88), Expect = 8e-04, Method: Compositional matrix adjust.

Identities = 44/173 (25%), Positives = 85/173 (49%), Gaps = 5/173 (3%)

Frame = +1

Query 1192 RVQSDQRRLVRRPDVKPNRPLIVILS-TVALDAVGIGLIMPVLPGLLRDLVHSNDVTAHY 1368

+ S + +++ R L+++LS +V + A+ L+ PVLP + +DL S +

Sbjct 2 KKDSKSKEMIQSEKRGSTRLLMMVLSLSVLVGAITADLVNPVLPLISKDLEASKSQVSW- 60

Query 1369 GILLALYALMQFACAPVLGALSDRFGRRPVLLVSLAGAAVDYAIMATAPFLWVLYigriv 1548

+++ AL+ P+ G +SD F R + + ++ A + A AP L +L +GR+V

Sbjct 61 --IVSGIALVLAIGVPIYGRISDFFELRKLYIFAIMILASGSLLCAIAPNLPLLVLGRMV 118

Query 1549 agitgatg-avagayiaDITDGDERARHFGFMSACFGFGMVAGPVLGGLMGGF 1704

G + ++ I+ + +R G ++ G G AGP+ GG++G +

Sbjct 119 QGAGMSAIPVLSIIAISKVFPQGKRGGALGIIAGSIGVGTAAGPIFGGVVGQY 171

> WP_032491576 chloramphenicol/florfenicol efflux MFS transporter

FexA [Staphylococcus simulans]

Length=475

Score = 38.5 bits (88), Expect = 8e-04, Method: Compositional matrix adjust.

Identities = 44/173 (25%), Positives = 85/173 (49%), Gaps = 5/173 (3%)

Frame = +1

Query 1192 RVQSDQRRLVRRPDVKPNRPLIVILS-TVALDAVGIGLIMPVLPGLLRDLVHSNDVTAHY 1368

+ S + +++ R L+++LS +V + A+ L+ PVLP + +DL S +

Sbjct 2 KKDSKSKEMIQSEKRGSTRLLMMVLSLSVLVGAITADLVNPVLPLISKDLEASKSQVSW- 60

Query 1369 GILLALYALMQFACAPVLGALSDRFGRRPVLLVSLAGAAVDYAIMATAPFLWVLYigriv 1548

+++ AL+ P+ G +SD F R + + ++ A + A AP L +L +GR+V

Sbjct 61 --IVSGIALVLAIGVPIYGRISDFFELRKLYIFAIMILASGSLLCAIAPNLPLLVLGRMV 118

Query 1549 agitgatg-avagayiaDITDGDERARHFGFMSACFGFGMVAGPVLGGLMGGF 1704

G + ++ I+ + +R G ++ G G AGP+ GG++G +

Sbjct 119 QGAGMSAIPVLSVIAISKVFPQGKRGGALGIIAGSIGVGTAAGPIFGGVVGQY 171

> CAJ31068 chloramphenicol/florfenicol exporter (plasmid) [Staphylococcus

simulans]

Length=475

Score = 38.5 bits (88), Expect = 8e-04, Method: Compositional matrix adjust.

Identities = 44/173 (25%), Positives = 85/173 (49%), Gaps = 5/173 (3%)

Frame = +1

Query 1192 RVQSDQRRLVRRPDVKPNRPLIVILS-TVALDAVGIGLIMPVLPGLLRDLVHSNDVTAHY 1368

+ S + +++ R L+++LS +V + A+ L+ PVLP + +DL S +

Sbjct 2 KKDSKSKEMIQSEKRGSTRLLMMVLSLSVLVGAITADLVNPVLPLISKDLEASKSQVSW- 60

Query 1369 GILLALYALMQFACAPVLGALSDRFGRRPVLLVSLAGAAVDYAIMATAPFLWVLYigriv 1548

+++ AL+ P+ G +SD F R + + ++ A + A AP L +L +GR+V

Sbjct 61 --IVSGIALVLAIGVPIYGRISDFFELRKLYIFAIMILASGSLLCAIAPNLPLLVLGRMV 118

Query 1549 agitgatg-avagayiaDITDGDERARHFGFMSACFGFGMVAGPVLGGLMGGF 1704

G + ++ I+ + +R G ++ G G AGP+ GG++G +

Sbjct 119 QGAGMSAIPVLSVIAISKVFPQGKRGGALGIIAGSIGVGTAAGPIFGGVVGQY 171

> SCA35775 putative chloramphenicol and florfenicol resistance

protein (CmlA) [Klebsiella pneumoniae]

Length=404

Score = 38.1 bits (87), Expect = 0.001, Method: Compositional matrix adjust.

Identities = 27/69 (39%), Positives = 40/69 (58%), Gaps = 10/69 (14%)

Frame = +1

Query 1261 ILSTVALDAVGIGL-IMPVLPGLLRDLVHSNDVTAHYGILLALYALMQFACAPVLGALSD 1437

IL+++A+D I L ++P +PG+L N A + L+LY +M + G LSD

Sbjct 24 ILASLAMD---IYLPVVPAMPGIL------NTTPAMIQLTLSLYMVMLGVGQVIFGPLSD 74

Query 1438 RFGRRPVLL 1464

R GRRP+LL

Sbjct 75 RIGRRPILL 83

> BAX23716 florfenicol/ chloramphenicol export protein FloR [Escherichia

coli]

Length=411

Score = 38.1 bits (87), Expect = 0.001, Method: Compositional matrix adjust.

Identities = 45/153 (29%), Positives = 70/153 (46%), Gaps = 9/153 (6%)

Frame = +1

Query 1261 ILSTVALDAVGIGL-IMPVLPGLLRDLVHS-NDVTAHYGILLALYALMQFACAPVLGALS 1434

IL+++A+D I L ++P +PG+L N A + L+LY +M + G LS

Sbjct 24 ILASLAMD---IYLPVVPAMPGILNTTPGILNTTPAMIQLTLSLYMVMLGVGQVIFGPLS 80

Query 1435 DRFGRRPVLL---VSLAGAAVDYAIMATAPFLWVLYigrivagitgatgavagayiaDIT 1605

DR GRRP+LL + A++ A +TAP +V + G + A

Sbjct 81 DRIGRRPILLAGATAFVIASLGAAWSSTAP-AFVAFRLLQAVGASAMLVATFATVRDVYA 139

Query 1606 DGDERARHFGFMSACFGFGMVAGPVLGGLMGGF 1704

+ E +G S+ F GP+ G L+G F

Sbjct 140 NRPEGVVIYGLFSSMLAFVPALGPIAGTLIGEF 172

> AIM49724 florfenicol exporter (plasmid) [Aeromonas salmonicida

subsp. salmonicida]

Length=404

Score = 38.1 bits (87), Expect = 0.001, Method: Compositional matrix adjust.

Identities = 44/154 (29%), Positives = 69/154 (45%), Gaps = 18/154 (12%)

Frame = +1

Query 1261 ILSTVALDAVGIGLIMPV---LPGLLRDLVHSNDVTAHYGILLALYALMQFACAPVLGAL 1431

IL+++A+D + +PV +PG+L N A + L+LY +M + G L

Sbjct 24 ILASLAMD-----IYLPVVRAMPGIL------NTTPAMIQLTLSLYMVMLGVGQVIFGPL 72

Query 1432 SDRFGRRPVLL---VSLAGAAVDYAIMATAPFLWVLYigrivagitgatgavagayiaDI 1602

SDR GRRP+LL + A++ A +TAP +V + G + A

Sbjct 73 SDRIGRRPILLAGATAFVIASLGAAWSSTAP-AFVAFRLLQAVGASAMLVATFATVRDVY 131

Query 1603 TDGDERARHFGFMSACFGFGMVAGPVLGGLMGGF 1704

+ E +G S+ F GP+ G L+G F

Sbjct 132 ANRPEGVVIYGLFSSMLAFVPALGPIAGALIGEF 165

> ARA90576 chloramphenicol/florfenicol exporter [Staphylococcus

sciuri]

Length=475

Score = 37.7 bits (86), Expect = 0.001, Method: Compositional matrix adjust.

Identities = 43/173 (25%), Positives = 85/173 (49%), Gaps = 5/173 (3%)

Frame = +1

Query 1192 RVQSDQRRLVRRPDVKPNRPLIVILS-TVALDAVGIGLIMPVLPGLLRDLVHSNDVTAHY 1368

+ S + +++ R L+++LS +V + A+ ++ PVLP + +DL S +

Sbjct 2 KKDSKSKEMIQSEKRGSTRLLMMVLSLSVLVGAITSDIVNPVLPLISKDLEASKSQVSW- 60

Query 1369 GILLALYALMQFACAPVLGALSDRFGRRPVLLVSLAGAAVDYAIMATAPFLWVLYigriv 1548

+++ AL+ P+ G +SD F R + + ++ A + A AP L +L +GR+V

Sbjct 61 --IVSGIALVLAIGVPIYGRISDFFELRKLYIFTIMILASGSLLCAIAPNLPLLVLGRMV 118

Query 1549 agitgatg-avagayiaDITDGDERARHFGFMSACFGFGMVAGPVLGGLMGGF 1704

G + ++ I+ + +R G ++ G G AGP+ GG++G +

Sbjct 119 QGAGMSAIPVLSVIAISKVFPQGKRGGALGIIAGSIGVGTAAGPIFGGVVGQY 171

> AQW34658 phenicol exporter protein [Staphylococcus sciuri]

Length=475

Score = 37.7 bits (86), Expect = 0.001, Method: Compositional matrix adjust.

Identities = 43/173 (25%), Positives = 85/173 (49%), Gaps = 5/173 (3%)

Frame = +1

Query 1192 RVQSDQRRLVRRPDVKPNRPLIVILS-TVALDAVGIGLIMPVLPGLLRDLVHSNDVTAHY 1368

+ S + +++ R L+++LS +V + A+ ++ PVLP + +DL S +

Sbjct 2 KKDSKSKEMIQSEKRGSTRLLMMVLSLSVLVGAITSDIVNPVLPLISKDLEASKSQVSW- 60

Query 1369 GILLALYALMQFACAPVLGALSDRFGRRPVLLVSLAGAAVDYAIMATAPFLWVLYigriv 1548

+++ AL+ P+ G +SD F R + + ++ A + A AP L +L +GR+V

Sbjct 61 --IVSGIALVLAIGVPIYGRISDFFELRKLYIFTIMILASGSLLCAIAPNLPLLVLGRMV 118

Query 1549 agitgatg-avagayiaDITDGDERARHFGFMSACFGFGMVAGPVLGGLMGGF 1704

G + ++ I+ + +R G ++ G G AGP+ GG++G +

Sbjct 119 QGAGMSAIPVLSVIAISKVFPQGKRGGALGIIAGSIGVGTAAGPIFGGVVGQY 171

> AMP42360 florfenicol efflux pump [uncultured bacterium IN-10]

Length=404

Score = 37.7 bits (86), Expect = 0.001, Method: Compositional matrix adjust.

Identities = 27/69 (39%), Positives = 40/69 (58%), Gaps = 10/69 (14%)

Frame = +1

Query 1261 ILSTVALDAVGIGL-IMPVLPGLLRDLVHSNDVTAHYGILLALYALMQFACAPVLGALSD 1437

IL+++A+D I L ++P +PG+L N + + L+LY +M + G LSD

Sbjct 24 ILASLAMD---IYLPVVPAMPGIL------NTTPSTIQLTLSLYMVMLGVGQVIFGPLSD 74

Query 1438 RFGRRPVLL 1464

R GRRPVLL

Sbjct 75 RIGRRPVLL 83

> AIU94575 chloramphenicol and florfenicol resistance protein variant

[Stenotrophomonas maltophilia]

Length=404

Score = 37.7 bits (86), Expect = 0.001, Method: Compositional matrix adjust.

Identities = 27/69 (39%), Positives = 40/69 (58%), Gaps = 10/69 (14%)

Frame = +1

Query 1261 ILSTVALDAVGIGL-IMPVLPGLLRDLVHSNDVTAHYGILLALYALMQFACAPVLGALSD 1437

IL+++A+D I L ++P +PG+L N + + L+LY +M + G LSD

Sbjct 24 ILASLAMD---IYLPVVPAMPGIL------NTTPSTIQLTLSLYMVMLGVGQVIFGPLSD 74

Query 1438 RFGRRPVLL 1464

R GRRPVLL

Sbjct 75 RIGRRPVLL 83

> AQW34746 phenicol exporter protein [Staphylococcus sciuri]

Length=475

Score = 37.4 bits (85), Expect = 0.002, Method: Compositional matrix adjust.

Identities = 43/173 (25%), Positives = 85/173 (49%), Gaps = 5/173 (3%)

Frame = +1

Query 1192 RVQSDQRRLVRRPDVKPNRPLIVILS-TVALDAVGIGLIMPVLPGLLRDLVHSNDVTAHY 1368

+ S + +++ R L+++LS +V + A+ ++ PVLP + +DL S +

Sbjct 2 KKDSKSKEMIQSEKRGSTRLLMMVLSLSVLVGAITSDIVNPVLPLISKDLEASKSQVSW- 60

Query 1369 GILLALYALMQFACAPVLGALSDRFGRRPVLLVSLAGAAVDYAIMATAPFLWVLYigriv 1548

+++ AL+ P+ G +SD F R + + ++ A + A AP L +L +GR+V

Sbjct 61 --IVSGIALVLAIGVPIYGRISDFFELRKLYIFAIMILASGSLLCAIAPNLPLLVLGRMV 118

Query 1549 agitgatg-avagayiaDITDGDERARHFGFMSACFGFGMVAGPVLGGLMGGF 1704

G + ++ I+ + +R G ++ G G AGP+ GG++G +

Sbjct 119 QGAGMSAIPVLSVIAISKVFPQGKRGGALGIIAGSIGVGTAAGPIFGGVVGQY 171

> AQW34733 phenicol exporter protein [Staphylococcus sciuri]

Length=475

Score = 37.4 bits (85), Expect = 0.002, Method: Compositional matrix adjust.

Identities = 43/173 (25%), Positives = 85/173 (49%), Gaps = 5/173 (3%)

Frame = +1

Query 1192 RVQSDQRRLVRRPDVKPNRPLIVILS-TVALDAVGIGLIMPVLPGLLRDLVHSNDVTAHY 1368

+ S + +++ R L+++LS +V + A+ ++ PVLP + +DL S +

Sbjct 2 KKDSKSKEMIQSEKRGSTRLLMMVLSLSVLVGAITSDIVNPVLPLISKDLEASKSQVSW- 60

Query 1369 GILLALYALMQFACAPVLGALSDRFGRRPVLLVSLAGAAVDYAIMATAPFLWVLYigriv 1548

+++ AL+ P+ G +SD F R + + ++ A + A AP L +L +GR+V

Sbjct 61 --IVSGIALVLAIGVPIYGRISDFFELRKLYIFAIMILASGSLLCAIAPNLPLLVLGRMV 118

Query 1549 agitgatg-avagayiaDITDGDERARHFGFMSACFGFGMVAGPVLGGLMGGF 1704

G + ++ I+ + +R G ++ G G AGP+ GG++G +

Sbjct 119 QGAGMSAIPVLSVIAISKVFPQGKRGGALGIIAGSIGVGTAAGPIFGGVVGQY 171

> AQW34598 phenicol exporter protein [Staphylococcus sciuri]

Length=475

Score = 37.4 bits (85), Expect = 0.002, Method: Compositional matrix adjust.

Identities = 43/173 (25%), Positives = 85/173 (49%), Gaps = 5/173 (3%)

Frame = +1

Query 1192 RVQSDQRRLVRRPDVKPNRPLIVILS-TVALDAVGIGLIMPVLPGLLRDLVHSNDVTAHY 1368

+ S + +++ R L+++LS +V + A+ ++ PVLP + +DL S +

Sbjct 2 KKDSKSKEMIQSEKRGSTRLLMMVLSLSVLVGAITSDIVNPVLPLISKDLEASKSQVSW- 60

Query 1369 GILLALYALMQFACAPVLGALSDRFGRRPVLLVSLAGAAVDYAIMATAPFLWVLYigriv 1548

+++ AL+ P+ G +SD F R + + ++ A + A AP L +L +GR+V

Sbjct 61 --IVSGIALVLAIGVPIYGRISDFFELRKLYIFAIMILASGSLLCAIAPNLPLLVLGRMV 118

Query 1549 agitgatg-avagayiaDITDGDERARHFGFMSACFGFGMVAGPVLGGLMGGF 1704

G + ++ I+ + +R G ++ G G AGP+ GG++G +

Sbjct 119 QGAGMSAIPVLSVIAISKVFPQGKRGGALGIIAGSIGVGTAAGPIFGGVVGQY 171

> ATB51834 FloR (plasmid) [Actinobacillus indolicus]

Length=404

Score = 36.2 bits (82), Expect = 0.004, Method: Compositional matrix adjust.

Identities = 26/69 (38%), Positives = 40/69 (58%), Gaps = 10/69 (14%)

Frame = +1

Query 1261 ILSTVALDAVGIGL-IMPVLPGLLRDLVHSNDVTAHYGILLALYALMQFACAPVLGALSD 1437

IL+++A+D I L ++P +PG+L N + + L+LY +M + G LSD

Sbjct 24 ILASLAMD---IYLPVVPAMPGVL------NTTPSIIQLTLSLYMVMLGVGQVIFGPLSD 74

Query 1438 RFGRRPVLL 1464

R GRRP+LL

Sbjct 75 RIGRRPILL 83

> XP_002341611 florfenicol exporter, putative [Talaromyces stipitatus

ATCC 10500]

Length=541

Score = 35.8 bits (81), Expect = 0.006, Method: Compositional matrix adjust.

Identities = 37/143 (26%), Positives = 66/143 (46%), Gaps = 4/143 (3%)

Frame = +1

Query 1276 ALDAVGIGLIMPVLPGLLRDLVHSNDVTAHYGILLALYALMQFACAPVLGALSDRFGRRP 1455

A + + P + + DL N T+ + + +Y ++Q + G SD GRR

Sbjct 69 AFSPLSSNIYFPSIDTISHDL---NVNTSLVALTITVYMIVQGIAPSIFGTFSDTCGRRL 125

Query 1456 VLLVSLA-GAAVDYAIMATAPFLWVLYigrivagitgatgavagayiaDITDGDERARHF 1632

+SL A + A+ T+ + ++ + + AG + AT +++ IADI + DER

Sbjct 126 TFAISLTIYTAANLALAFTSNYPMLMVLRGVQAGGSAATISISAGVIADIANPDERGGFM 185

Query 1633 GFMSACFGFGMVAGPVLGGLMGG 1701

G + G GP++GGL+

Sbjct 186 GTNAGVRMTGQAIGPIIGGLLNS 208

> EED24224 florfenicol exporter, putative [Talaromyces stipitatus

ATCC 10500]

Length=541

Score = 35.8 bits (81), Expect = 0.006, Method: Compositional matrix adjust.

Identities = 37/143 (26%), Positives = 66/143 (46%), Gaps = 4/143 (3%)

Frame = +1

Query 1276 ALDAVGIGLIMPVLPGLLRDLVHSNDVTAHYGILLALYALMQFACAPVLGALSDRFGRRP 1455

A + + P + + DL N T+ + + +Y ++Q + G SD GRR

Sbjct 69 AFSPLSSNIYFPSIDTISHDL---NVNTSLVALTITVYMIVQGIAPSIFGTFSDTCGRRL 125

Query 1456 VLLVSLA-GAAVDYAIMATAPFLWVLYigrivagitgatgavagayiaDITDGDERARHF 1632

+SL A + A+ T+ + ++ + + AG + AT +++ IADI + DER

Sbjct 126 TFAISLTIYTAANLALAFTSNYPMLMVLRGVQAGGSAATISISAGVIADIANPDERGGFM 185

Query 1633 GFMSACFGFGMVAGPVLGGLMGG 1701

G + G GP++GGL+

Sbjct 186 GTNAGVRMTGQAIGPIIGGLLNS 208

> KTC83795 florfenicol efflux pump [Legionella brunensis]

Length=396

Score = 35.0 bits (79), Expect = 0.008, Method: Compositional matrix adjust.

Identities = 20/43 (47%), Positives = 27/43 (63%), Gaps = 1/43 (2%)

Frame = +1

Query 1954 FHWDATTIGISLAAFGILHSLAQAMITGPVAARLGERRALMLG 2082

F+ D+TT+ SL F + +LAQ MI GP+A R G RR +L

Sbjct 40 FNTDSTTMQASLYVFMLTVALAQLMI-GPLADRFGRRRVALLS 81

> XP_018148689 florfenicol exporter [Pochonia chlamydosporia 170]

Length=547

Score = 35.4 bits (80), Expect = 0.009, Method: Compositional matrix adjust.

Identities = 37/116 (32%), Positives = 59/116 (51%), Gaps = 7/116 (6%)

Frame = +1

Query 1369 GILLALYALMQFACAPVL-GALSDRFGRRPVLLVSLA---GAAVDYAIMATAPFLWVLYi 1536

+ + +Y +MQ A AP+ G LSD+ GRRPV ++ A GA + A+ L VL

Sbjct 111 NLTVTVYQIMQ-AIAPLFFGDLSDQIGRRPVYALTFAIYLGANIGLALQHNYAALMVL-- 167

Query 1537 grivagitgatgavagayiaDITDGDERARHFGFMSACFGFGMVAGPVLGGLMGGF 1704

+ + + AT A+ A +AD+T ER + + + F PVLGG++ +

Sbjct 168 RALQSTGSSATVAIGSAVVADLTTAAERGGYITAVQSSVMFAPALAPVLGGILTQY 223

> OAQ72606 florfenicol exporter [Pochonia chlamydosporia 170]

Length=547

Score = 35.4 bits (80), Expect = 0.009, Method: Compositional matrix adjust.

Identities = 37/116 (32%), Positives = 59/116 (51%), Gaps = 7/116 (6%)

Frame = +1

Query 1369 GILLALYALMQFACAPVL-GALSDRFGRRPVLLVSLA---GAAVDYAIMATAPFLWVLYi 1536

+ + +Y +MQ A AP+ G LSD+ GRRPV ++ A GA + A+ L VL

Sbjct 111 NLTVTVYQIMQ-AIAPLFFGDLSDQIGRRPVYALTFAIYLGANIGLALQHNYAALMVL-- 167

Query 1537 grivagitgatgavagayiaDITDGDERARHFGFMSACFGFGMVAGPVLGGLMGGF 1704

+ + + AT A+ A +AD+T ER + + + F PVLGG++ +

Sbjct 168 RALQSTGSSATVAIGSAVVADLTTAAERGGYITAVQSSVMFAPALAPVLGGILTQY 223

> AQW34668 chloramphenicol/florfenicol exporter (plasmid) [Staphylococcus

sciuri]

Length=475

Score = 35.0 bits (79), Expect = 0.009, Method: Compositional matrix adjust.

Identities = 40/175 (23%), Positives = 78/175 (45%), Gaps = 9/175 (5%)

Frame = +1

Query 1192 RVQSDQRRLVRRPDVKPNRPLIVILS-TVALDAVGIGLIMPVLPGLLRDLVHSNDVTAHY 1368

+ S + +++ R L+++LS +V + A+ + PVLP + +DL S

Sbjct 2 KKDSKSKEMIQSEKRGSTRLLMMVLSLSVLVGAITADFVNPVLPLISKDLEASKSQVIW- 60

Query 1369 GILLALYALMQFACAPVLGALSDRFGRRPVLLVS---LAGAAVDYAIMATAPFLWVLYig 1539

+++ AL+ P+ G +SD F R + + + LA ++ AI P L +

Sbjct 61 --IVSGIALVLAIGVPIYGRISDFFELRKLYIFAIMILASGSLLCAIATNLPLL--VLGR 116

Query 1540 rivagitgatgavagayiaDITDGDERARHFGFMSACFGFGMVAGPVLGGLMGGF 1704

+ A ++ I+ + +R G ++ G G AGP+ GG++G +

Sbjct 117 MVQGAGMSAIPVLSVIAISKVFPQGKRGGALGIIAGSIGVGTAAGPIFGGVVGQY 171

> AQW34568 chloramphenicol/florfenicol exporter [Staphylococcus

sciuri]

Length=475

Score = 35.0 bits (79), Expect = 0.009, Method: Compositional matrix adjust.

Identities = 40/175 (23%), Positives = 78/175 (45%), Gaps = 9/175 (5%)

Frame = +1

Query 1192 RVQSDQRRLVRRPDVKPNRPLIVILS-TVALDAVGIGLIMPVLPGLLRDLVHSNDVTAHY 1368

+ S + +++ R L+++LS +V + A+ + PVLP + +DL S

Sbjct 2 KKDSKSKEMIQSEKRGSTRLLMMVLSLSVLVGAITADFVNPVLPLISKDLEASKSQVIW- 60

Query 1369 GILLALYALMQFACAPVLGALSDRFGRRPVLLVS---LAGAAVDYAIMATAPFLWVLYig 1539

+++ AL+ P+ G +SD F R + + + LA ++ AI P L +

Sbjct 61 --IVSGIALVLAIGVPIYGRISDFFELRKLYIFAIMILASGSLLCAIATNLPLL--VLGR 116

Query 1540 rivagitgatgavagayiaDITDGDERARHFGFMSACFGFGMVAGPVLGGLMGGF 1704

+ A ++ I+ + +R G ++ G G AGP+ GG++G +

Sbjct 117 MVQGAGMSAIPVLSVIAISKVFPQGKRGGALGIIAGSIGVGTAAGPIFGGVVGQY 171

> XP_002480947 florfenicol exporter, putative [Talaromyces stipitatus

ATCC 10500]

Length=564

Score = 35.0 bits (79), Expect = 0.010, Method: Compositional matrix adjust.

Identities = 46/157 (29%), Positives = 73/157 (46%), Gaps = 14/157 (9%)

Frame = +1

Query 1255 IVILSTVA--LDAVGIGLIMPVLPGLLRDLVHSNDVTAHYGILLALYALMQF-ACAPV-L 1422

IVI ++ A + + P + L +DL S + I L + + M F APV +

Sbjct 102 IVIAASCAGFFSPISSQIYFPAMNTLAKDLSVSISL-----INLTMTSYMIFQGIAPVFI 156

Query 1423 GALSDRFGRRPVLLVSLA---GAAVDYAIMATAPFLWVLYigrivagitgatgavagayi 1593

G +D GRRP + GA V A+ + L+VL + + + T A++ I

Sbjct 157 GDFADNVGRRPAYFLCFVIYLGANVGLALQNSYAALFVL--RCMQSAGSSTTIALSAGVI 214

Query 1594 aDITDGDERARHFGFMSACFGFGMVAGPVLGGLMGGF 1704

AD+ ER + GF++A G GPV+GGL+ +

Sbjct 215 ADVASVAERGSYMGFVTAGSLLGPALGPVIGGLLSQY 251

> EED20513 florfenicol exporter, putative [Talaromyces stipitatus

ATCC 10500]

Length=564

Score = 35.0 bits (79), Expect = 0.010, Method: Compositional matrix adjust.

Identities = 46/157 (29%), Positives = 73/157 (46%), Gaps = 14/157 (9%)

Frame = +1

Query 1255 IVILSTVA--LDAVGIGLIMPVLPGLLRDLVHSNDVTAHYGILLALYALMQF-ACAPV-L 1422

IVI ++ A + + P + L +DL S + I L + + M F APV +

Sbjct 102 IVIAASCAGFFSPISSQIYFPAMNTLAKDLSVSISL-----INLTMTSYMIFQGIAPVFI 156

Query 1423 GALSDRFGRRPVLLVSLA---GAAVDYAIMATAPFLWVLYigrivagitgatgavagayi 1593

G +D GRRP + GA V A+ + L+VL + + + T A++ I

Sbjct 157 GDFADNVGRRPAYFLCFVIYLGANVGLALQNSYAALFVL--RCMQSAGSSTTIALSAGVI 214

Query 1594 aDITDGDERARHFGFMSACFGFGMVAGPVLGGLMGGF 1704

AD+ ER + GF++A G GPV+GGL+ +

Sbjct 215 ADVASVAERGSYMGFVTAGSLLGPALGPVIGGLLSQY 251

> OCL08714 florfenicol exporter [Glonium stellatum]

Length=512

Score = 35.0 bits (79), Expect = 0.012, Method: Compositional matrix adjust.

Identities = 50/190 (26%), Positives = 72/190 (38%), Gaps = 18/190 (9%)

Frame = +1

Query 1180 IKNPRVQSDQRRLVRRPDVKP-NRP------------LIVILSTVAL-DAVGIGLIMPVL 1317

+KNP QS + D P N P L+ I+S L + + P L

Sbjct 1 MKNPSTQSSTPVDPEKTDETPINEPPYHILTPRQKKQLVYIVSLAGLFSPLSSNIYFPAL 60

Query 1318 PGLLRDLVHSNDVTAHYGILLALYALMQFACAPVLGALSDRFGRRPVLLVSLAGAAVDYA 1497

+ RDL S + A + + Y ++Q A +D GRRP+ + + +

Sbjct 61 DTIARDLHVSVSLIA---LTVTSYMILQGIAPSFWSAFADSLGRRPIYISTFIVYIIANI 117

Query 1498 IMATAPFLWVLYigrivagitgatgavaga-yiaDITDGDERARHFGFMSACFGFGMVAG 1674

+A P VL R + + GA IADI ER G G G

Sbjct 118 GLAITPNFPVLMTFRAIQAAGSSATISVGAGVIADIATPAERGGFVGIYGGIRMLGQSVG 177

Query 1675 PVLGGLMGGF 1704

PVLGG++ F

Sbjct 178 PVLGGILAQF 187

> KTD21279 florfenicol efflux pump [Legionella londiniensis]

Length=407

Score = 33.9 bits (76), Expect = 0.023, Method: Compositional matrix adjust.

Identities = 32/133 (24%), Positives = 63/133 (47%), Gaps = 4/133 (3%)

Frame = +1

Query 1300 LIMPVLPGLLRDLVHSNDVTAHYGILLALYALMQFACAPVLGALSDRFGRRPV-LLVSLA 1476

+ +P +PGL S+ + L+++ L V+G LSD FGR+ + LLV+L

Sbjct 32 IYVPAIPGLTHLFAVSDSTML---LTLSIFMLTAGIMQLVVGPLSDSFGRKKIALLVTLI 88

Query 1477 GAAVDYAIMATAPFLWVLYigrivagitgatgavagayiaDITDGDERARHFGFMSACFG 1656

AA + + L++++ I A + + + + D GD+ A+ + +++

Sbjct 89 FAAGCILCASASNALFLIFARMIQAIGSCGMLVLGFSIVRDCFSGDKSAKVYSYLNGIIS 148

Query 1657 FGMVAGPVLGGLM 1695

F + P +GG +

Sbjct 149 FSPMFAPFIGGYL 161

> ACV76847 major facilitator superfamily MFS_1 [Nakamurella multipartita

DSM 44233]

Length=426

Score = 33.9 bits (76), Expect = 0.024, Method: Compositional matrix adjust.

Identities = 34/100 (34%), Positives = 49/100 (49%), Gaps = 8/100 (8%)

Frame = +1

Query 1255 IVILSTVALDAVGIGLIMPVLPGL---LRDLVHSNDVTAHYGILLALYALMQFACAPVLG 1425

++ L +A A + LI P+L L + DLV + + LA + + P+ G

Sbjct 28 VIALYALAFMASCLMLIAPLLVTLALKVGDLVGAEAAPGRLALTLAAGSFLALFANPLFG 87

Query 1426 ALSDR----FG-RRPVLLVSLAGAAVDYAIMATAPFLWVL 1530

LSDR FG RRP +L+ LAG V A +A A L V+

Sbjct 88 KLSDRTTSRFGMRRPWMLLGLAGGTVGIATIAYAQTLIVV 127

> CRL95727 Bicyclomycin resistance protein (Sulfonamide resistance

protein) [Acinetobacter baumannii]

Length=404

Score = 33.5 bits (75), Expect = 0.028, Method: Compositional matrix adjust.

Identities = 25/106 (24%), Positives = 47/106 (44%), Gaps = 6/106 (6%)

Frame = +1

Query 2026 MITGPVAARLGERRALMLGMIADGTGYILLAFATRGWMAFPIMVLLASGG-IGMPALQAM 2202

++ GP++ R+G ++ L G+ + AT W +L A GG +G+ +A

Sbjct 66 LVYGPLSDRIGRKKPLYFGLALYAVASLFCVLATNEWSLIAARILQALGGCVGVVMARAA 125

Query 2203 LSRQVDEERQGQLQGSLAALTSLTSIVGPLLFTAIYAASITTWNGW 2340

+ ++D + Q S+ + L I+ P++ A I W W

Sbjct 126 IRDRLDVQGSAQAFSSMMIVMGLAPILAPMI-----GAWILIWFPW 166

> XP_002487874 florfenicol exporter, putative [Talaromyces stipitatus

ATCC 10500]

Length=988

Score = 33.5 bits (75), Expect = 0.038, Method: Compositional matrix adjust.

Identities = 29/107 (27%), Positives = 50/107 (47%), Gaps = 5/107 (5%)

Frame = +1

Query 1393 LMQFACAPVLGALSDRFGRRPVLLVS---LAGAAVDYAIMATAPFLWVLYigrivagitg 1563

+ Q ++G SDR+GRRP L+ A A + A+ L VL + + +

Sbjct 569 IFQGVSPTIIGGYSDRYGRRPASLLCFTIFAAANIGLALQTNYAALLVL--RCMQSAGSS 626

Query 1564 atgavagayiaDITDGDERARHFGFMSACFGFGMVAGPVLGGLMGGF 1704

T A++ A ++D+ +R + G + G GP++GGL+ F

Sbjct 627 GTTALSSAVVSDLATRQQRGSYIGLAALGSSLGPALGPIIGGLLDHF 673

> EED12220 florfenicol exporter, putative [Talaromyces stipitatus

ATCC 10500]

Length=988

Score = 33.5 bits (75), Expect = 0.038, Method: Compositional matrix adjust.

Identities = 29/107 (27%), Positives = 50/107 (47%), Gaps = 5/107 (5%)

Frame = +1

Query 1393 LMQFACAPVLGALSDRFGRRPVLLVS---LAGAAVDYAIMATAPFLWVLYigrivagitg 1563

+ Q ++G SDR+GRRP L+ A A + A+ L VL + + +

Sbjct 569 IFQGVSPTIIGGYSDRYGRRPASLLCFTIFAAANIGLALQTNYAALLVL--RCMQSAGSS 626

Query 1564 atgavagayiaDITDGDERARHFGFMSACFGFGMVAGPVLGGLMGGF 1704

T A++ A ++D+ +R + G + G GP++GGL+ F

Sbjct 627 GTTALSSAVVSDLATRQQRGSYIGLAALGSSLGPALGPIIGGLLDHF 673

> XP_006670772 florfenicol exporter, putative [Cordyceps militaris

CM01]

Length=523

Score = 33.1 bits (74), Expect = 0.040, Method: Compositional matrix adjust.

Identities = 33/113 (29%), Positives = 53/113 (47%), Gaps = 1/113 (1%)

Frame = +1

Query 1369 GILLALYALMQFACAPVLGALSDRFGRRPVL-LVSLAGAAVDYAIMATAPFLWVLYigri 1545

+ + LY +MQ G LSD+ GRRPV A + A+ F +L++ +

Sbjct 95 NLTVTLYQVMQALSPLFFGDLSDQVGRRPVYAAAFAIYLAANVALAVQRSFGALLFLRAL 154

Query 1546 vagitgatgavagayiaDITDGDERARHFGFMSACFGFGMVAGPVLGGLMGGF 1704

+ + AT A+ A +AD+T ER + + A F PVLGG++ +

Sbjct 155 QSTGSSATVAIGNAVVADLTTPAERGGYITAVQATIMFAPALAPVLGGILTQY 207

> EGX91407 florfenicol exporter, putative [Cordyceps militaris

CM01]

Length=523

Score = 33.1 bits (74), Expect = 0.040, Method: Compositional matrix adjust.

Identities = 33/113 (29%), Positives = 53/113 (47%), Gaps = 1/113 (1%)

Frame = +1

Query 1369 GILLALYALMQFACAPVLGALSDRFGRRPVL-LVSLAGAAVDYAIMATAPFLWVLYigri 1545

+ + LY +MQ G LSD+ GRRPV A + A+ F +L++ +

Sbjct 95 NLTVTLYQVMQALSPLFFGDLSDQVGRRPVYAAAFAIYLAANVALAVQRSFGALLFLRAL 154

Query 1546 vagitgatgavagayiaDITDGDERARHFGFMSACFGFGMVAGPVLGGLMGGF 1704

+ + AT A+ A +AD+T ER + + A F PVLGG++ +

Sbjct 155 QSTGSSATVAIGNAVVADLTTPAERGGYITAVQATIMFAPALAPVLGGILTQY 207

> ASF64231 Bcr/CflA family drug resistance efflux transporter [Salmonella

enterica subsp. enterica serovar Typhimurium]

Length=401

Score = 33.1 bits (74), Expect = 0.042, Method: Compositional matrix adjust.

Identities = 24/83 (29%), Positives = 46/83 (55%), Gaps = 6/83 (7%)

Frame = +1

Query 1234 VKPNRPLIVILSTVA-LDAVGIGLIMPVLPGLLRDL-VHSNDVTAHYGILLALYALMQFA 1407

++P + +V L+ ++ L + + +P + DL + V+A + LA +A+ Q

Sbjct 1 MQPGKGFLVWLAGLSVLGFLATDMYLPAFAAIQADLQTPAAAVSASLSLFLAGFAVAQL- 59

Query 1408 CAPVLGALSDRFGRRPVLLVSLA 1476

+ G LSDR+GR+P+LL+ L+

Sbjct 60 ---LWGPLSDRYGRKPILLLGLS 79

> AEA08384 chloramphenicol transporter [Escherichia coli]

Length=419

Score = 32.7 bits (73), Expect = 0.045, Method: Compositional matrix adjust.

Identities = 41/173 (24%), Positives = 74/173 (43%), Gaps = 14/173 (8%)

Frame = +1

Query 1837 SFRWARGMTVVAALMAVFFIMQLVGQ---VPAALWVIFGEDRFHWDATTIGISLAAFGIL 2007

+F W + L++ F ++ +G +PA V F + A+TI ++L + ++

Sbjct 5 NFSWRYSLAATVLLLSPFDLLASLGMDMYLPA---VPFMPNALGTTASTIQLTLTTYLVM 61

Query 2008 HSLAQAMITGPVAARLGERRALMLGMIADGTGYILLAFATRGWMAFPIMVLLASGGIGMP 2187

Q ++ GP++ RLG R L+ G +A + LA + + + +L A G

Sbjct 62 IGAGQ-LLFGPLSDRLGRRPVLLGGGLAYVVASMGLALTSSAEVFLGLRILQACGASACL 120

Query 2188 ALQAMLSRQV--DEERQGQLQGSLAALTSLTSIVGPLLFTAIYAASITTWNGW 2340

R + E + G L ++ ++ VGPLL A + W GW

Sbjct 121 VSTFATVRDIYAGREESNVIYGILGSMLAMVPAVGPLL-----GALVDMWLGW 168

Score = 31.2 bits (69), Expect = 0.15, Method: Compositional matrix adjust.

Identities = 21/62 (34%), Positives = 32/62 (52%), Gaps = 3/62 (5%)

Frame = +1

Query 1279 LDAVGIGLIMPVLPGLLRDLVHSNDVTAHYGILLALYALMQFACAPVLGALSDRFGRRPV 1458

L ++G+ + +P +P + L + + L Y +M A + G LSDR GRRPV

Sbjct 25 LASLGMDMYLPAVPFMPNAL---GTTASTIQLTLTTYLVMIGAGQLLFGPLSDRLGRRPV 81

Query 1459 LL 1464

LL

Sbjct 82 LL 83

> H845_RS00265_-_25558887_translation chloramphenicol efflux pump

Length=381

Score = 32.7 bits (73), Expect = 0.047, Method: Compositional matrix adjust.

Identities = 38/140 (27%), Positives = 65/140 (46%), Gaps = 9/140 (6%)

Frame = +1

Query 1279 LDAVGIGLIMPVLPGLLRDLVHSNDVTAHYGILLALYALMQFACAPVL-GALSDRFGRRP 1455

+ A+G+ + +PV+P ++ D++ ++ + + + L+ C +L G LSD GRRP

Sbjct 23 MAALGMDVYLPVVP-IMPDVLSTDATLIQFSLT---FYLLVLGCGQLLFGPLSDHIGRRP 78

Query 1456 VLLVS---LAGAAVDYAIMATAPFLWVLYigrivagitgatgavagayiaDITDGDERAR 1626

VL S +G++ A + L++L+ G +GA A E

Sbjct 79 VLFGSALIFSGSSAALACTRSGD-LFLLFRILQAFGASGALVATFATVRDVYAGRREIGV 137

Query 1627 HFGFMSACFGFGMVAGPVLG 1686

+G SAC F GP+LG

Sbjct 138 VYGMFSACLAFVPAFGPILG 157

> AKU20099 chloramphenicol/florfenicol exporter [Enterococcus faecium]

Length=469

Score = 31.6 bits (70), Expect = 0.12, Method: Compositional matrix adjust.

Identities = 11/28 (39%), Positives = 17/28 (61%), Gaps = 0/28 (0%)

Frame = +1

Query 1615 ERARHFGFMSACFGFGMVAGPVLGGLMG 1698

+R R G ++ C G G GP+ GG++G

Sbjct 135 QRGRILGIIAGCIGVGTAGGPIFGGVVG 162

> AEV23046 florfenicol exporter protein,FexB (plasmid) [Enterococcus

faecium]

Length=469

Score = 31.6 bits (70), Expect = 0.12, Method: Compositional matrix adjust.

Identities = 11/28 (39%), Positives = 17/28 (61%), Gaps = 0/28 (0%)

Frame = +1

Query 1615 ERARHFGFMSACFGFGMVAGPVLGGLMG 1698

+R R G ++ C G G GP+ GG++G

Sbjct 135 QRGRILGIIAGCIGVGTAGGPIFGGVVG 162

> AEV23029 florfenicol exporter protein,FexB (plasmid) [Enterococcus

hirae]

Length=469

Score = 31.6 bits (70), Expect = 0.12, Method: Compositional matrix adjust.

Identities = 11/28 (39%), Positives = 17/28 (61%), Gaps = 0/28 (0%)

Frame = +1

Query 1615 ERARHFGFMSACFGFGMVAGPVLGGLMG 1698

+R R G ++ C G G GP+ GG++G

Sbjct 135 QRGRILGIIAGCIGVGTAGGPIFGGVVG 162

> ACI54526 drug resistance transporter, Bcr/CflA subfamily [Rhizobium

leguminosarum bv. trifolii WSM2304]

Length=396

Score = 31.2 bits (69), Expect = 0.16, Method: Compositional matrix adjust.

Identities = 25/68 (37%), Positives = 38/68 (56%), Gaps = 8/68 (12%)

Frame = +1

Query 1261 ILSTVALDAVGIGLIMPVLPGLLRDLVHSNDVTAHYGILLALYALMQFACAPVLGALSDR 1440

IL+++A+D + +PV+P + + L S V + L+LY L+ V G +SD

Sbjct 25 ILASLAMD-----IYLPVVPTMPQALGTSPAVIQ---LTLSLYMLVLGVGQIVFGPISDI 76

Query 1441 FGRRPVLL 1464

GRRPVLL

Sbjct 77 VGRRPVLL 84

> AFH88776 florfenicol export protein, partial (plasmid) [Escherichia

coli]

Length=296

Score = 30.8 bits (68), Expect = 0.18, Method: Compositional matrix adjust.

Identities = 15/29 (52%), Positives = 19/29 (66%), Gaps = 0/29 (0%)

Frame = +1

Query 1378 LALYALMQFACAPVLGALSDRFGRRPVLL 1464

L+LY +M + G LSDR GRRP+LL

Sbjct 2 LSLYMVMLGVGQVIFGPLSDRIGRRPILL 30

> GAT30590 florfenicol exporter [Aspergillus luchuensis]

Length=524

Score = 30.8 bits (68), Expect = 0.19, Method: Compositional matrix adjust.

Identities = 32/128 (25%), Positives = 58/128 (45%), Gaps = 2/128 (2%)

Frame = +1

Query 1327 LRDLVHSNDVTAHYGIL-LALYALMQFACAPVLGALSDRFGRRPVLLVSLAGAAV-DYAI 1500

L D+ S +++ L + +Y ++Q G++SD GRRPV + + V + A+

Sbjct 82 LDDVSKSLNISMSLATLTITVYMIVQGLAPSFWGSMSDATGRRPVFIGTFVVYLVANIAL 141

Query 1501 MATAPFLWVLYigrivagitgatgavagayiaDITDGDERARHFGFMSACFGFGMVAGPV 1680

+ + ++ + A + AT ++ I DIT+ +ER G G GPV

Sbjct 142 AESKNYGELMAFRALQAAGSAATISIGAGVIGDITNSEERGSLVGIFGGVRMLGQGIGPV 201

Query 1681 LGGLMGGF 1704

GG+ +

Sbjct 202 FGGIFTQY 209

> GAA89875 florfenicol exporter [Aspergillus kawachii IFO 4308]

Length=524

Score = 30.8 bits (68), Expect = 0.19, Method: Compositional matrix adjust.

Identities = 32/128 (25%), Positives = 58/128 (45%), Gaps = 2/128 (2%)

Frame = +1

Query 1327 LRDLVHSNDVTAHYGIL-LALYALMQFACAPVLGALSDRFGRRPVLLVSLAGAAV-DYAI 1500

L D+ S +++ L + +Y ++Q G++SD GRRPV + + V + A+

Sbjct 82 LDDVSKSLNISMSLATLTITVYMIVQGLAPSFWGSMSDATGRRPVFIGTFVVYLVANIAL 141

Query 1501 MATAPFLWVLYigrivagitgatgavagayiaDITDGDERARHFGFMSACFGFGMVAGPV 1680

+ + ++ + A + AT ++ I DIT+ +ER G G GPV

Sbjct 142 AESKNYGELMAFRALQAAGSAATISIGAGVIGDITNSEERGSLVGIFGGVRMLGQGIGPV 201

Query 1681 LGGLMGGF 1704

GG+ +

Sbjct 202 FGGIFTQY 209

> GAQ45384 florfenicol exporter [Aspergillus niger]

Length=524

Score = 30.8 bits (68), Expect = 0.19, Method: Compositional matrix adjust.

Identities = 32/128 (25%), Positives = 58/128 (45%), Gaps = 2/128 (2%)

Frame = +1

Query 1327 LRDLVHSNDVTAHYGIL-LALYALMQFACAPVLGALSDRFGRRPVLLVSLAGAAV-DYAI 1500

L D+ S +++ L + +Y ++Q G++SD GRRPV + + V + A+

Sbjct 82 LDDVSKSLNISMSLATLTITVYMIVQGLAPSFWGSMSDATGRRPVFIGTFVVYLVANIAL 141

Query 1501 MATAPFLWVLYigrivagitgatgavagayiaDITDGDERARHFGFMSACFGFGMVAGPV 1680

+ + ++ + A + AT ++ I DIT+ +ER G G GPV

Sbjct 142 AESKNYGELMAFRALQAAGSAATISIGAGVIGDITNSEERGSLVGIFGGVRMLGQGIGPV 201

Query 1681 LGGLMGGF 1704

GG+ +

Sbjct 202 FGGIFTQY 209

> AAZ39943 membrane transport protein, partial [Rhizobium leguminosarum

bv. trifolii TA1]

Length=194

Score = 30.0 bits (66), Expect = 0.22, Method: Compositional matrix adjust.

Identities = 28/69 (41%), Positives = 38/69 (55%), Gaps = 10/69 (14%)

Frame = +1

Query 1261 ILSTVALDAVGIGL-IMPVLPGLLRDLVHSNDVTAHYGILLALYALMQFACAPVLGALSD 1437

IL+++A+D I L I+PV+PG L A + L+LY L+ V G +SD

Sbjct 25 ILASLAMD---IYLPIVPVMPGAL------GTSPAVIQLTLSLYMLVLGVGQMVFGPISD 75

Query 1438 RFGRRPVLL 1464

GRRPVLL

Sbjct 76 IVGRRPVLL 84

> ORY09537 putative florfenicol exporter [Clohesyomyces aquaticus]

Length=552

Score = 30.8 bits (68), Expect = 0.23, Method: Compositional matrix adjust.

Identities = 36/130 (28%), Positives = 57/130 (44%), Gaps = 6/130 (5%)

Frame = +1

Query 1327 LRDLVHSNDVTAH-YGILLALYALMQFACAPVLGALSDRFGRRPVLLVSLA---GAAVDY 1494

L L H V++ + L Y + Q G +D GRRP +V GA +

Sbjct 101 LTTLSHQYGVSSTLMNLTLTSYMIFQGLAPTFFGDFADMEGRRPAYIVGFVIYMGANIGL 160

Query 1495 AIMATAPFLWVLYigrivagitgatgavagayiaDITDGDERARHFGFMSACFGFGMVAG 1674

A+ + L+VL + + + AT A+ +ADI ER G++++ V

Sbjct 161 ALQNSYAALFVL--RCLQSTGSSATIALGNGVVADIATSSERGTWMGYVTSGPMIAPVLA 218

Query 1675 PVLGGLMGGF 1704

PV+GGL+ F

Sbjct 219 PVIGGLLAQF 228

> CAL20780 putative multidrug resistance protein [Yersinia pestis

CO92]

Length=400

Score = 30.0 bits (66), Expect = 0.33, Method: Compositional matrix adjust.

Identities = 32/120 (27%), Positives = 56/120 (47%), Gaps = 6/120 (5%)

Frame = +1

Query 1291 GIGLIMPVLPGLLRDLVHSNDVTAHYGILLALYALMQFACAPVLGALSDRFGRRPVLLVS 1470

GI L +P +P + + L S + +AL+ L+ + G L D++GR+P+ ++

Sbjct 18 GIDLYLPTIPAIAKGLNSSESLIQS---TIALFILVLGIGQLIAGPLVDKYGRKPIAIIG 74

Query 1471 --LAGAAVDYAIMATAPFLWVLYigrivagitgatgavagayiaDITDGDERARHFGFMS 1644

L A +A P ++V + T VA + + D G+E AR FGF++

Sbjct 75 IVLYMLGAAMAALAVDPVMFV-SSRLLQGVSVCCTAVVAFSGVRDRLSGNEAARAFGFLN 133

> KTD48180 florfenicol efflux pump [Legionella quinlivanii]

Length=396

Score = 30.0 bits (66), Expect = 0.34, Method: Compositional matrix adjust.

Identities = 19/43 (44%), Positives = 26/43 (60%), Gaps = 2/43 (5%)

Frame = +1

Query 1954 FHWDATTIGISLAAFGILHSLAQAMITGPVAARLGERRALMLG 2082

FH D T + SL F +L Q +ITGP+A ++G RR + LG

Sbjct 40 FHTDNTIMQASLYVFMFTVALGQ-LITGPLADKVG-RRNIALG 80

> ACS55612 drug resistance transporter, Bcr/CflA subfamily [Rhizobium

leguminosarum bv. trifolii WSM1325]

Length=395

Score = 30.0 bits (66), Expect = 0.35, Method: Compositional matrix adjust.

Identities = 24/68 (35%), Positives = 37/68 (54%), Gaps = 8/68 (12%)

Frame = +1

Query 1261 ILSTVALDAVGIGLIMPVLPGLLRDLVHSNDVTAHYGILLALYALMQFACAPVLGALSDR 1440

IL+++A+D + +P++P + L S V + L+LY L+ V G +SD

Sbjct 25 ILASLAMD-----IYLPIVPVMPEALGTSPAVIQ---LTLSLYMLVLGVGQIVFGPISDI 76

Query 1441 FGRRPVLL 1464

GRRPVLL

Sbjct 77 IGRRPVLL 84

> AFN07605 florfenicol-resistance protein, partial [Escherichia

coli]

Length=183

Score = 28.9 bits (63), Expect = 0.45, Method: Compositional matrix adjust.

Identities = 29/99 (29%), Positives = 43/99 (43%), Gaps = 4/99 (4%)

Frame = +1

Query 1417 VLGALSDRFGRRPVLL---VSLAGAAVDYAIMATAPFLWVLYigrivagitgatgavaga 1587

+ G LSDR GRRP+LL + A++ A +TAP +V + G + A

Sbjct 7 IFGPLSDRIGRRPILLAGATAFVIASLGAAWSSTAP-AFVAFRLLQAVGASAMLVATFAT 65

Query 1588 yiaDITDGDERARHFGFMSACFGFGMVAGPVLGGLMGGF 1704

+ E +G S+ F GP+ G L+G F

Sbjct 66 VRDVYANRPEGVVIYGLFSSMLAFVPALGPIAGVLIGEF 104

> ACU36899 major facilitator superfamily MFS_1 [Actinosynnema mirum

DSM 43827]

Length=421

Score = 29.6 bits (65), Expect = 0.46, Method: Compositional matrix adjust.

Identities = 68/287 (24%), Positives = 117/287 (41%), Gaps = 40/287 (14%)

Frame = +1

Query 1288 VGIGLIMPVLPGLLRDLVHSNDVTAHYGILLALYALMQFACAPVLGALSDR----FGRRP 1455

V +G+ ++P L+ V A YG+L A+ A + G+LSDR FG+R

Sbjct 41 VALGVSTTLIPALMEQ-VDPEAKVALYGVLTAVGAAAGLVANILFGSLSDRTRSRFGKRN 99

Query 1456 --VLLVSLAGAAVDYAIMATAPFLWVLYigrivagitgatgavagayiaDITDGDERARH 1629

+ + L AA A+ T+ F ++ + A A A + D + R

Sbjct 100 GWIAVGGLVSAASLSAMSTTSSFGLLIVLYIGYQIGLNALLAPLYAVLPDRVPTERR--- 156

Query 1630 FGFMSACFGFGMVAGPVLGGLMGGFSPHapffaaaalnglnflTGCFLLPESHK------ 1791

G SA G GM+ G ++G TG ++P +

Sbjct 157 -GLASAVIGLGMLLAQSAGAVIGA------------AFLDEIRTGMAIMPWAIAVTALVF 203

Query 1792 ---GERRPLRREALNPLASFRWARGMTVVAA---LMAVF-FIMQLVGQVPAALWVIFG-E 1947

+ RP E + AR V A L+A+F + L+ A L+ +F +

Sbjct 204 AVFAKDRPNLDEPREAFSLVELARTFKVPADRDYLLALFGRLTLLLAYFSATLYQLFILQ 263

Query 1948 DRFHWDATTIGISLAAFGILHSLAQ---AMITGPVAARLGERRALML 2079

D DA + ++A G++ ++A +I+GP++ R+G R+ L++

Sbjct 264 DYIKLDAAGVAGTVALAGVIMAVASGIGTLISGPLSDRIGRRKPLII 310

> XP_022383730 florfenicol exporter [Aspergillus bombycis]

Length=516

Score = 29.3 bits (64), Expect = 0.64, Method: Compositional matrix adjust.

Identities = 46/196 (23%), Positives = 79/196 (40%), Gaps = 18/196 (9%)

Frame = +1

Query 1141 ITDKHNMSTNL--SVIKNPRVQSDQRRLVRRPDVKPNRPLIVILSTVAL-DAVGIGLIMP 1311

+ D+ S +L S +++ QS + R + ++ I+S A+ + + P

Sbjct 17 VEDERQPSDSLGTSQLEDQVAQSKPHHIFSR---RKKLQMVCIVSMAAIFSPLSSNIYFP 73

Query 1312 VLPGLLRDLVHSNDVTAHYGILLALYALMQFACAPVLGALSDRFGRRPVLLVSLAGAAVD 1491

L + R L S + + + +Y ++Q G++SD GRRPV + G +

Sbjct 74 ALGEVSRSLNVSMSLAT---LTVTIYMIVQGITPTFWGSISDATGRRPVFI----GTFIV 126

Query 1492 YAIMATAPFLWVLYigrivagitgatgavagay-----iaDITDGDERARHFGFMSACFG 1656

Y I + A + Y + A G+ A I DIT ER G

Sbjct 127 YMIASIALAVSTNYGELMAFRALQAAGSAATISIGAGVIGDITTSAERGSLVGIFGGVRM 186

Query 1657 FGMVAGPVLGGLMGGF 1704

G GPV+GG++ +

Sbjct 187 LGQGIGPVIGGILTQY 202

> OGM40013 florfenicol exporter [Aspergillus bombycis]

Length=516

Score = 29.3 bits (64), Expect = 0.64, Method: Compositional matrix adjust.

Identities = 46/196 (23%), Positives = 79/196 (40%), Gaps = 18/196 (9%)

Frame = +1

Query 1141 ITDKHNMSTNL--SVIKNPRVQSDQRRLVRRPDVKPNRPLIVILSTVAL-DAVGIGLIMP 1311

+ D+ S +L S +++ QS + R + ++ I+S A+ + + P

Sbjct 17 VEDERQPSDSLGTSQLEDQVAQSKPHHIFSR---RKKLQMVCIVSMAAIFSPLSSNIYFP 73

Query 1312 VLPGLLRDLVHSNDVTAHYGILLALYALMQFACAPVLGALSDRFGRRPVLLVSLAGAAVD 1491

L + R L S + + + +Y ++Q G++SD GRRPV + G +

Sbjct 74 ALGEVSRSLNVSMSLAT---LTVTIYMIVQGITPTFWGSISDATGRRPVFI----GTFIV 126

Query 1492 YAIMATAPFLWVLYigrivagitgatgavagay-----iaDITDGDERARHFGFMSACFG 1656

Y I + A + Y + A G+ A I DIT ER G

Sbjct 127 YMIASIALAVSTNYGELMAFRALQAAGSAATISIGAGVIGDITTSAERGSLVGIFGGVRM 186

Query 1657 FGMVAGPVLGGLMGGF 1704

G GPV+GG++ +

Sbjct 187 LGQGIGPVIGGILTQY 202

> EKU30462 putative chloramphenicol and florfenicol resistance

protein (CmlA) [Alcaligenes sp. HPC1271]

Length=381

Score = 29.3 bits (64), Expect = 0.64, Method: Compositional matrix adjust.

Identities = 13/20 (65%), Positives = 14/20 (70%), Gaps = 0/20 (0%)

Frame = +1

Query 1417 VLGALSDRFGRRPVLLVSLA 1476

+ G LSDR GRRPVLL A

Sbjct 50 IFGPLSDRIGRRPVLLTGAA 69

> mdfA_-_945448_translation mdfA: multidrug efflux system protein

Length=410

Score = 28.9 bits (63), Expect = 0.80, Method: Compositional matrix adjust.

Identities = 12/16 (75%), Positives = 14/16 (88%), Gaps = 0/16 (0%)

Frame = +1

Query 1417 VLGALSDRFGRRPVLL 1464

+LG LSDR GRRPV+L

Sbjct 71 LLGPLSDRIGRRPVML 86

Lambda K H a alpha

0.318 0.134 0.401 0.792 4.96

Gapped

Lambda K H a alpha sigma

0.267 0.0410 0.140 1.90 42.6 43.6

Effective search space used: 333988234

**Query=** NODE_6_length_3876_cov_14.3766

Length=3876

***** No hits found *****

Lambda K H a alpha

0.318 0.134 0.401 0.792 4.96

Gapped

Lambda K H a alpha sigma

0.267 0.0410 0.140 1.90 42.6 43.6

Effective search space used: 245182928

**Query=** NODE_7_length_3309_cov_20.3762

Length=3309

***** No hits found *****

Lambda K H a alpha

0.318 0.134 0.401 0.792 4.96

Gapped

Lambda K H a alpha sigma

0.267 0.0410 0.140 1.90 42.6 43.6

Effective search space used: 207741360

**Query=** NODE_8_length_2573_cov_0.985691

Length=2573

***** No hits found *****

Lambda K H a alpha

0.318 0.134 0.401 0.792 4.96

Gapped

Lambda K H a alpha sigma

0.267 0.0410 0.140 1.90 42.6 43.6

Effective search space used: 159135872

**Query=** NODE_9_length_2440_cov_21.7056

Length=2440

***** No hits found *****

Lambda K H a alpha

0.318 0.134 0.401 0.792 4.96

Gapped

Lambda K H a alpha sigma

0.267 0.0410 0.140 1.90 42.6 43.6

Effective search space used: 150112704

**Query=** NODE_10_length_1816_cov_21.8917

Length=1816

***** No hits found *****

Lambda K H a alpha

0.318 0.134 0.401 0.792 4.96

Gapped

Lambda K H a alpha sigma

0.267 0.0410 0.140 1.90 42.6 43.6

Effective search space used: 109182806

**Query=** NODE_11_length_1694_cov_14.3931

Length=1694

***** No hits found *****

Lambda K H a alpha

0.318 0.134 0.401 0.792 4.96

Gapped

Lambda K H a alpha sigma

0.267 0.0410 0.140 1.90 42.6 43.6

Effective search space used: 100688508

**Query=** NODE_12_length_1101_cov_3.1037

Length=1101

***** No hits found *****

Lambda K H a alpha

0.318 0.134 0.401 0.792 4.96

Gapped

Lambda K H a alpha sigma

0.267 0.0410 0.140 1.90 42.6 43.6

Effective search space used: 61525898

**Query=** NODE_13_length_1063_cov_0.538462

Length=1063

***** No hits found *****

Lambda K H a alpha

0.318 0.134 0.401 0.792 4.96

Gapped

Lambda K H a alpha sigma

0.267 0.0410 0.140 1.90 42.6 43.6

Effective search space used: 58796080

**Query=** NODE_14_length_988_cov_0.528455

Length=988

***** No hits found *****

Lambda K H a alpha

0.318 0.134 0.401 0.792 4.96

Gapped

Lambda K H a alpha sigma

0.267 0.0410 0.140 1.90 42.6 43.6

Effective search space used: 53936128

**Query=** NODE_15_length_946_cov_0.445665

Length=946

Score E

Sequences producing significant alignments: (Bits) Value

AQW34720 copper-transporting P-type ATPase B [Staphylococcus sc... [30.8](file:///Users/JMBNew/Desktop/Articles%20en%20Cours/Article%20Diane/Bb_42-1_output-blast_named.html#BL_ORD_ID:271) 0.029

> AQW34720 copper-transporting P-type ATPase B [Staphylococcus

sciuri]

Length=701

Score = 30.8 bits (68), Expect = 0.029, Method: Compositional matrix adjust.

Identities = 17/50 (34%), Positives = 22/50 (44%), Gaps = 0/50 (0%)

Frame = +3

Query 156 WGAGLWPAGIASWAPDLPSFGFTESPARDAYAYTFDNITRTMDRFTEQLK 305

WGAG + A L S G SPA A + I ++ FT +LK

Sbjct 652 WGAGYNVIAVPLAAGILASIGLILSPAVGAILMSLSTIIVAINAFTLKLK 701

Lambda K H a alpha

0.318 0.134 0.401 0.792 4.96

Gapped

Lambda K H a alpha sigma

0.267 0.0410 0.140 1.90 42.6 43.6

Effective search space used: 50986496

**Query=** NODE_16_length_939_cov_0.375616

Length=939

***** No hits found *****

Lambda K H a alpha

0.318 0.134 0.401 0.792 4.96

Gapped

Lambda K H a alpha sigma

0.267 0.0410 0.140 1.90 42.6 43.6

Effective search space used: 50565120

**Query=** NODE_17_length_901_cov_0.466408

Length=901

***** No hits found *****

Lambda K H a alpha

0.318 0.134 0.401 0.792 4.96

Gapped

Lambda K H a alpha sigma

0.267 0.0410 0.140 1.90 42.6 43.6

Effective search space used: 47826176

**Query=** NODE_18_length_891_cov_0.341623

Length=891

***** No hits found *****

Lambda K H a alpha

0.318 0.134 0.401 0.792 4.96

Gapped

Lambda K H a alpha sigma

0.267 0.0410 0.140 1.90 42.6 43.6

Effective search space used: 47194112

**Query=** NODE_19_length_862_cov_0.431293

Length=862

***** No hits found *****

Lambda K H a alpha

0.318 0.134 0.401 0.792 4.96

Gapped

Lambda K H a alpha sigma

0.267 0.0410 0.140 1.90 42.6 43.6

Effective search space used: 45448850

**Query=** NODE_20_length_852_cov_0.423448

Length=852

***** No hits found *****

Lambda K H a alpha

0.318 0.134 0.401 0.792 4.96

Gapped

Lambda K H a alpha sigma

0.267 0.0410 0.140 1.90 42.6 43.6

Effective search space used: 44814680

**Query=** NODE_21_length_845_cov_0.71727

Length=845

***** No hits found *****

Lambda K H a alpha

0.318 0.134 0.401 0.792 4.96

Gapped

Lambda K H a alpha sigma

0.267 0.0410 0.140 1.90 42.6 43.6

Effective search space used: 44180510

**Query=** NODE_22_length_843_cov_3.45391

Length=843

***** No hits found *****

Lambda K H a alpha

0.318 0.134 0.401 0.792 4.96

Gapped

Lambda K H a alpha sigma

0.267 0.0410 0.140 1.90 42.6 43.6

Effective search space used: 44180510

**Query=** NODE_23_length_832_cov_0.453901

Length=832

***** No hits found *****

Lambda K H a alpha

0.318 0.134 0.401 0.792 4.96

Gapped

Lambda K H a alpha sigma

0.267 0.0410 0.140 1.90 42.6 43.6

Effective search space used: 43334950

**Query=** NODE_24_length_831_cov_0.457386

Length=831

***** No hits found *****

Lambda K H a alpha

0.318 0.134 0.401 0.792 4.96

Gapped

Lambda K H a alpha sigma

0.267 0.0410 0.140 1.90 42.6 43.6

Effective search space used: 43334950

**Query=** NODE_25_length_798_cov_0.687034

Length=798

***** No hits found *****

Lambda K H a alpha

0.318 0.134 0.401 0.792 4.96

Gapped

Lambda K H a alpha sigma

0.267 0.0410 0.140 1.90 42.6 43.6

Effective search space used: 41009660

**Query=** NODE_26_length_790_cov_0.434389

Length=790

***** No hits found *****

Lambda K H a alpha

0.318 0.134 0.401 0.792 4.96

Gapped

Lambda K H a alpha sigma

0.267 0.0410 0.140 1.90 42.6 43.6

Effective search space used: 40721664

**Query=** NODE_27_length_788_cov_0.397882

Length=788

***** No hits found *****

Lambda K H a alpha

0.318 0.134 0.401 0.792 4.96

Gapped

Lambda K H a alpha sigma

0.267 0.0410 0.140 1.90 42.6 43.6

Effective search space used: 40509572

**Query=** NODE_28_length_787_cov_0.366667

Length=787

***** No hits found *****

Lambda K H a alpha

0.318 0.134 0.401 0.792 4.96

Gapped

Lambda K H a alpha sigma

0.267 0.0410 0.140 1.90 42.6 43.6

Effective search space used: 40509572

**Query=** NODE_29_length_778_cov_0.568356

Length=778

***** No hits found *****

Lambda K H a alpha

0.318 0.134 0.401 0.792 4.96

Gapped

Lambda K H a alpha sigma

0.267 0.0410 0.140 1.90 42.6 43.6

Effective search space used: 39873296

**Query=** NODE_30_length_766_cov_0.161189

Length=766

***** No hits found *****

Lambda K H a alpha

0.318 0.134 0.401 0.792 4.96

Gapped

Lambda K H a alpha sigma

0.267 0.0410 0.140 1.90 42.6 43.6

Effective search space used: 39024928

**Query=** NODE_31_length_760_cov_0.265403

Length=760

***** No hits found *****

Lambda K H a alpha

0.318 0.134 0.401 0.792 4.96

Gapped

Lambda K H a alpha sigma

0.267 0.0410 0.140 1.90 42.6 43.6

Effective search space used: 38600744

**Query=** NODE_32_length_758_cov_0.400951

Length=758

***** No hits found *****

Lambda K H a alpha

0.318 0.134 0.401 0.792 4.96

Gapped

Lambda K H a alpha sigma

0.267 0.0410 0.140 1.90 42.6 43.6

Effective search space used: 38388652

**Query=** NODE_33_length_757_cov_0.377778

Length=757

***** No hits found *****

Lambda K H a alpha

0.318 0.134 0.401 0.792 4.96

Gapped

Lambda K H a alpha sigma

0.267 0.0410 0.140 1.90 42.6 43.6

Effective search space used: 38388652

**Query=** NODE_34_length_754_cov_0.671451

Length=754

***** No hits found *****

Lambda K H a alpha

0.318 0.134 0.401 0.792 4.96

Gapped

Lambda K H a alpha sigma

0.267 0.0410 0.140 1.90 42.6 43.6

Effective search space used: 38176560

**Query=** NODE_35_length_741_cov_0.319218

Length=741

***** No hits found *****

Lambda K H a alpha

0.318 0.134 0.401 0.792 4.96

Gapped

Lambda K H a alpha sigma

0.267 0.0410 0.140 1.90 42.6 43.6

Effective search space used: 37328192

**Query=** NODE_36_length_739_cov_0.534314

Length=739

Score E

Sequences producing significant alignments: (Bits) Value

ABO50235 23S rRNA m(2)A-2503 methyltransferase [Desulfotomaculu... [25.8](file:///Users/JMBNew/Desktop/Articles%20en%20Cours/Article%20Diane/Bb_42-1_output-blast_named.html#BL_ORD_ID:31) 0.64

> ABO50235 23S rRNA m(2)A-2503 methyltransferase [Desulfotomaculum

reducens MI-1]

Length=350

Score = 25.8 bits (55), Expect = 0.64, Method: Compositional matrix adjust.

Identities = 12/25 (48%), Positives = 13/25 (52%), Gaps = 0/25 (0%)

Frame = +3

Query 240 GCRGACTFHASTARWWMRR*SPGRA 314

GCR C F AST +R SPG

Sbjct 117 GCRMGCLFCASTINGLVRNLSPGEI 141

Lambda K H a alpha

0.318 0.134 0.401 0.792 4.96

Gapped

Lambda K H a alpha sigma

0.267 0.0410 0.140 1.90 42.6 43.6

Effective search space used: 37116100

**Query=** NODE_37_length_739_cov_0.495098

Length=739

***** No hits found *****

Lambda K H a alpha

0.318 0.134 0.401 0.792 4.96

Gapped

Lambda K H a alpha sigma

0.267 0.0410 0.140 1.90 42.6 43.6

Effective search space used: 37116100

**Query=** NODE_38_length_735_cov_0.503289

Length=735

***** No hits found *****

Lambda K H a alpha

0.318 0.134 0.401 0.792 4.96

Gapped

Lambda K H a alpha sigma

0.267 0.0410 0.140 1.90 42.6 43.6

Effective search space used: 36904008

**Query=** NODE_39_length_732_cov_0.557025

Length=732

***** No hits found *****

Lambda K H a alpha

0.318 0.134 0.401 0.792 4.96

Gapped

Lambda K H a alpha sigma

0.267 0.0410 0.140 1.90 42.6 43.6

Effective search space used: 36691916

**Query=** NODE_40_length_725_cov_0.493311

Length=725

***** No hits found *****

Lambda K H a alpha

0.318 0.134 0.401 0.792 4.96

Gapped

Lambda K H a alpha sigma

0.267 0.0410 0.140 1.90 42.6 43.6

Effective search space used: 36055640

**Query=** NODE_41_length_720_cov_0.387858

Length=720

***** No hits found *****

Lambda K H a alpha

0.318 0.134 0.401 0.792 4.96

Gapped

Lambda K H a alpha sigma

0.267 0.0410 0.140 1.90 42.6 43.6

Effective search space used: 35843548

**Query=** NODE_42_length_718_cov_0.461929

Length=718

***** No hits found *****

Lambda K H a alpha

0.318 0.134 0.401 0.792 4.96

Gapped

Lambda K H a alpha sigma

0.267 0.0410 0.140 1.90 42.6 43.6

Effective search space used: 35631456

**Query=** NODE_43_length_718_cov_0.519459

Length=718

***** No hits found *****

Lambda K H a alpha

0.318 0.134 0.401 0.792 4.96

Gapped

Lambda K H a alpha sigma

0.267 0.0410 0.140 1.90 42.6 43.6

Effective search space used: 35631456

**Query=** NODE_44_length_713_cov_0.515358

Length=713

***** No hits found *****

Lambda K H a alpha

0.318 0.134 0.401 0.792 4.96

Gapped

Lambda K H a alpha sigma

0.267 0.0410 0.140 1.90 42.6 43.6

Effective search space used: 35536598

**Query=** NODE_45_length_712_cov_0.439316

Length=712

***** No hits found *****

Lambda K H a alpha

0.318 0.134 0.401 0.792 4.96

Gapped

Lambda K H a alpha sigma

0.267 0.0410 0.140 1.90 42.6 43.6

Effective search space used: 35536598

**Query=** NODE_46_length_711_cov_0.482877

Length=711

***** No hits found *****

Lambda K H a alpha

0.318 0.134 0.401 0.792 4.96

Gapped

Lambda K H a alpha sigma

0.267 0.0410 0.140 1.90 42.6 43.6

Effective search space used: 35536598

**Query=** NODE_47_length_711_cov_0.883562

Length=711

***** No hits found *****

Lambda K H a alpha

0.318 0.134 0.401 0.792 4.96

Gapped

Lambda K H a alpha sigma

0.267 0.0410 0.140 1.90 42.6 43.6

Effective search space used: 35536598

**Query=** NODE_48_length_708_cov_0.452668

Length=708

***** No hits found *****

Lambda K H a alpha

0.318 0.134 0.401 0.792 4.96

Gapped

Lambda K H a alpha sigma

0.267 0.0410 0.140 1.90 42.6 43.6

Effective search space used: 35323804

**Query=** NODE_49_length_705_cov_0.543253

Length=705

***** No hits found *****

Lambda K H a alpha

0.318 0.134 0.401 0.792 4.96

Gapped

Lambda K H a alpha sigma

0.267 0.0410 0.140 1.90 42.6 43.6

Effective search space used: 35111010

**Query=** NODE_50_length_704_cov_0.615251

Length=704

***** No hits found *****

Lambda K H a alpha

0.318 0.134 0.401 0.792 4.96

Gapped

Lambda K H a alpha sigma

0.267 0.0410 0.140 1.90 42.6 43.6

Effective search space used: 34898216

**Query=** NODE_51_length_703_cov_0.3125

Length=703

***** No hits found *****

Lambda K H a alpha

0.318 0.134 0.401 0.792 4.96

Gapped

Lambda K H a alpha sigma

0.267 0.0410 0.140 1.90 42.6 43.6

Effective search space used: 34898216

**Query=** NODE_52_length_702_cov_0.737391

Length=702

***** No hits found *****

Lambda K H a alpha

0.318 0.134 0.401 0.792 4.96

Gapped

Lambda K H a alpha sigma

0.267 0.0410 0.140 1.90 42.6 43.6

Effective search space used: 34898216

**Query=** NODE_53_length_696_cov_0.655536

Length=696

***** No hits found *****

Lambda K H a alpha

0.318 0.134 0.401 0.792 4.96

Gapped

Lambda K H a alpha sigma

0.267 0.0410 0.140 1.90 42.6 43.6

Effective search space used: 34472628

**Query=** NODE_54_length_687_cov_0.369643

Length=687

***** No hits found *****

Lambda K H a alpha

0.318 0.134 0.401 0.792 4.96

Gapped

Lambda K H a alpha sigma

0.267 0.0410 0.140 1.90 42.6 43.6

Effective search space used: 33834246

**Query=** NODE_55_length_684_cov_0.624776

Length=684

***** No hits found *****

Lambda K H a alpha

0.318 0.134 0.401 0.792 4.96

Gapped

Lambda K H a alpha sigma

0.267 0.0410 0.140 1.90 42.6 43.6

Effective search space used: 33621452

**Query=** NODE_56_length_681_cov_1.14982

Length=681

***** No hits found *****

Lambda K H a alpha

0.318 0.134 0.401 0.792 4.96

Gapped

Lambda K H a alpha sigma

0.267 0.0410 0.140 1.90 42.6 43.6

Effective search space used: 33408658

**Query=** NODE_57_length_680_cov_0.576854

Length=680

***** No hits found *****

Lambda K H a alpha

0.318 0.134 0.401 0.792 4.96

Gapped

Lambda K H a alpha sigma

0.267 0.0410 0.140 1.90 42.6 43.6

Effective search space used: 33195864

**Query=** NODE_58_length_672_cov_0.469725

Length=672

***** No hits found *****

Lambda K H a alpha

0.318 0.134 0.401 0.792 4.96

Gapped

Lambda K H a alpha sigma

0.267 0.0410 0.140 1.90 42.6 43.6

Effective search space used: 32770276

**Query=** NODE_59_length_667_cov_0.488889

Length=667

***** No hits found *****

Lambda K H a alpha

0.318 0.134 0.401 0.792 4.96

Gapped

Lambda K H a alpha sigma

0.267 0.0410 0.140 1.90 42.6 43.6

Effective search space used: 32344688

**Query=** NODE_60_length_666_cov_0.64564

Length=666

***** No hits found *****

Lambda K H a alpha

0.318 0.134 0.401 0.792 4.96

Gapped

Lambda K H a alpha sigma

0.267 0.0410 0.140 1.90 42.6 43.6

Effective search space used: 32344688

**Query=** NODE_61_length_664_cov_0.346369

Length=664

***** No hits found *****

Lambda K H a alpha

0.318 0.134 0.401 0.792 4.96

Gapped

Lambda K H a alpha sigma

0.267 0.0410 0.140 1.90 42.6 43.6

Effective search space used: 32131894

**Query=** NODE_62_length_660_cov_0.981238

Length=660

***** No hits found *****

Lambda K H a alpha

0.318 0.134 0.401 0.792 4.96

Gapped

Lambda K H a alpha sigma

0.267 0.0410 0.140 1.90 42.6 43.6

Effective search space used: 31919100

**Query=** NODE_63_length_660_cov_0.393996

Length=660

***** No hits found *****

Lambda K H a alpha

0.318 0.134 0.401 0.792 4.96

Gapped

Lambda K H a alpha sigma

0.267 0.0410 0.140 1.90 42.6 43.6

Effective search space used: 31919100

**Query=** NODE_64_length_659_cov_6.01692

Length=659

***** No hits found *****

Lambda K H a alpha

0.318 0.134 0.401 0.792 4.96

Gapped

Lambda K H a alpha sigma

0.267 0.0410 0.140 1.90 42.6 43.6

Effective search space used: 31706306

**Query=** NODE_65_length_656_cov_0.287335

Length=656

***** No hits found *****

Lambda K H a alpha

0.318 0.134 0.401 0.792 4.96

Gapped

Lambda K H a alpha sigma

0.267 0.0410 0.140 1.90 42.6 43.6

Effective search space used: 31493512

**Query=** NODE_66_length_654_cov_0.489564

Length=654

***** No hits found *****

Lambda K H a alpha

0.318 0.134 0.401 0.792 4.96

Gapped

Lambda K H a alpha sigma

0.267 0.0410 0.140 1.90 42.6 43.6

Effective search space used: 31493512

**Query=** NODE_67_length_654_cov_0.370019

Length=654

***** No hits found *****

Lambda K H a alpha

0.318 0.134 0.401 0.792 4.96

Gapped

Lambda K H a alpha sigma

0.267 0.0410 0.140 1.90 42.6 43.6

Effective search space used: 31493512

**Query=** NODE_68_length_648_cov_0.443378

Length=648

Score E

Sequences producing significant alignments: (Bits) Value

XP_002487874 florfenicol exporter, putative [Talaromyces stipit... [27.3](file:///Users/JMBNew/Desktop/Articles%20en%20Cours/Article%20Diane/Bb_42-1_output-blast_named.html#BL_ORD_ID:585) 0.20

EED12220 florfenicol exporter, putative [Talaromyces stipitatus... [27.3](file:///Users/JMBNew/Desktop/Articles%20en%20Cours/Article%20Diane/Bb_42-1_output-blast_named.html#BL_ORD_ID:422) 0.20

> XP_002487874 florfenicol exporter, putative [Talaromyces stipitatus

ATCC 10500]

Length=988

Score = 27.3 bits (59), Expect = 0.20, Method: Compositional matrix adjust.

Identities = 14/61 (23%), Positives = 28/61 (46%), Gaps = 2/61 (3%)

Frame = +3

Query 216 WTTMANGADWIFCLVRTDAEAKPQAGISLLLIDMKTPGITVRPVSY--TPPTLPTILPVY 389

WT G W+ + + +++ G LL++D +T++ + Y + L I P +

Sbjct 246 WTNNDIGYRWLVDVFDKETKSQASRGWRLLILDGHGSHVTMKFIEYCDSNRILLAIFPAH 305

Query 390 A 392

A

Sbjct 306 A 306

> EED12220 florfenicol exporter, putative [Talaromyces stipitatus

ATCC 10500]

Length=988

Score = 27.3 bits (59), Expect = 0.20, Method: Compositional matrix adjust.

Identities = 14/61 (23%), Positives = 28/61 (46%), Gaps = 2/61 (3%)

Frame = +3

Query 216 WTTMANGADWIFCLVRTDAEAKPQAGISLLLIDMKTPGITVRPVSY--TPPTLPTILPVY 389

WT G W+ + + +++ G LL++D +T++ + Y + L I P +

Sbjct 246 WTNNDIGYRWLVDVFDKETKSQASRGWRLLILDGHGSHVTMKFIEYCDSNRILLAIFPAH 305

Query 390 A 392

A

Sbjct 306 A 306

Lambda K H a alpha

0.318 0.134 0.401 0.792 4.96

Gapped

Lambda K H a alpha sigma

0.267 0.0410 0.140 1.90 42.6 43.6

Effective search space used: 31067924

**Query=** NODE_69_length_645_cov_0.567568

Length=645

***** No hits found *****

Lambda K H a alpha

0.318 0.134 0.401 0.792 4.96

Gapped

Lambda K H a alpha sigma

0.267 0.0410 0.140 1.90 42.6 43.6

Effective search space used: 31170416

**Query=** NODE_70_length_637_cov_0.584314

Length=637

***** No hits found *****

Lambda K H a alpha

0.318 0.134 0.401 0.792 4.96

Gapped

Lambda K H a alpha sigma

0.267 0.0410 0.140 1.90 42.6 43.6

Effective search space used: 30529928

**Query=** NODE_71_length_636_cov_0.667976

Length=636

***** No hits found *****

Lambda K H a alpha

0.318 0.134 0.401 0.792 4.96

Gapped

Lambda K H a alpha sigma

0.267 0.0410 0.140 1.90 42.6 43.6

Effective search space used: 30529928

**Query=** NODE_72_length_632_cov_0.582178

Length=632

***** No hits found *****

Lambda K H a alpha

0.318 0.134 0.401 0.792 4.96

Gapped

Lambda K H a alpha sigma

0.267 0.0410 0.140 1.90 42.6 43.6

Effective search space used: 30102936

**Query=** NODE_73_length_632_cov_0.617822

Length=632

***** No hits found *****

Lambda K H a alpha

0.318 0.134 0.401 0.792 4.96

Gapped

Lambda K H a alpha sigma

0.267 0.0410 0.140 1.90 42.6 43.6

Effective search space used: 30102936

**Query=** NODE_74_length_630_cov_0.534791

Length=630

***** No hits found *****

Lambda K H a alpha

0.318 0.134 0.401 0.792 4.96

Gapped

Lambda K H a alpha sigma

0.267 0.0410 0.140 1.90 42.6 43.6

Effective search space used: 30102936

**Query=** NODE_75_length_630_cov_0.616302

Length=630

***** No hits found *****

Lambda K H a alpha

0.318 0.134 0.401 0.792 4.96

Gapped

Lambda K H a alpha sigma

0.267 0.0410 0.140 1.90 42.6 43.6

Effective search space used: 30102936

**Query=** NODE_76_length_629_cov_0.503984

Length=629

***** No hits found *****

Lambda K H a alpha

0.318 0.134 0.401 0.792 4.96

Gapped

Lambda K H a alpha sigma

0.267 0.0410 0.140 1.90 42.6 43.6

Effective search space used: 29889440

**Query=** NODE_77_length_625_cov_0.471888

Length=625

***** No hits found *****

Lambda K H a alpha

0.318 0.134 0.401 0.792 4.96

Gapped

Lambda K H a alpha sigma

0.267 0.0410 0.140 1.90 42.6 43.6

Effective search space used: 29675944

**Query=** NODE_78_length_624_cov_0.488934

Length=624

***** No hits found *****

Lambda K H a alpha

0.318 0.134 0.401 0.792 4.96

Gapped

Lambda K H a alpha sigma

0.267 0.0410 0.140 1.90 42.6 43.6

Effective search space used: 29675944

**Query=** NODE_79_length_623_cov_0.290323

Length=623

***** No hits found *****

Lambda K H a alpha

0.318 0.134 0.401 0.792 4.96

Gapped

Lambda K H a alpha sigma

0.267 0.0410 0.140 1.90 42.6 43.6

Effective search space used: 29462448

**Query=** NODE_80_length_621_cov_0.576923

Length=621

***** No hits found *****

Lambda K H a alpha

0.318 0.134 0.401 0.792 4.96

Gapped

Lambda K H a alpha sigma

0.267 0.0410 0.140 1.90 42.6 43.6

Effective search space used: 29462448

**Query=** NODE_81_length_617_cov_0.6

Length=617

***** No hits found *****

Lambda K H a alpha

0.318 0.134 0.401 0.792 4.96

Gapped

Lambda K H a alpha sigma

0.267 0.0410 0.140 1.90 42.6 43.6

Effective search space used: 29035456

**Query=** NODE_82_length_616_cov_0.251534

Length=616

***** No hits found *****

Lambda K H a alpha

0.318 0.134 0.401 0.792 4.96

Gapped

Lambda K H a alpha sigma

0.267 0.0410 0.140 1.90 42.6 43.6

Effective search space used: 29035456

**Query=** NODE_83_length_614_cov_0.367556

Length=614

***** No hits found *****

Lambda K H a alpha

0.318 0.134 0.401 0.792 4.96

Gapped

Lambda K H a alpha sigma

0.267 0.0410 0.140 1.90 42.6 43.6

Effective search space used: 28821960

**Query=** NODE_84_length_612_cov_0.579381

Length=612

***** No hits found *****

Lambda K H a alpha

0.318 0.134 0.401 0.792 4.96

Gapped

Lambda K H a alpha sigma

0.267 0.0410 0.140 1.90 42.6 43.6

Effective search space used: 28821960

**Query=** NODE_85_length_611_cov_0.38843

Length=611

***** No hits found *****

Lambda K H a alpha

0.318 0.134 0.401 0.792 4.96

Gapped

Lambda K H a alpha sigma

0.267 0.0410 0.140 1.90 42.6 43.6

Effective search space used: 28608464

**Query=** NODE_86_length_609_cov_0.516598

Length=609

***** No hits found *****

Lambda K H a alpha

0.318 0.134 0.401 0.792 4.96

Gapped

Lambda K H a alpha sigma

0.267 0.0410 0.140 1.90 42.6 43.6

Effective search space used: 28608464

**Query=** NODE_87_length_609_cov_0.60166

Length=609

***** No hits found *****

Lambda K H a alpha

0.318 0.134 0.401 0.792 4.96

Gapped

Lambda K H a alpha sigma

0.267 0.0410 0.140 1.90 42.6 43.6

Effective search space used: 28608464

**Query=** NODE_88_length_608_cov_0.351351

Length=608

***** No hits found *****

Lambda K H a alpha

0.318 0.134 0.401 0.792 4.96

Gapped

Lambda K H a alpha sigma

0.267 0.0410 0.140 1.90 42.6 43.6

Effective search space used: 28394968

**Query=** NODE_89_length_607_cov_0.635417

Length=607

***** No hits found *****

Lambda K H a alpha

0.318 0.134 0.401 0.792 4.96

Gapped

Lambda K H a alpha sigma

0.267 0.0410 0.140 1.90 42.6 43.6

Effective search space used: 28394968

**Query=** NODE_90_length_603_cov_0.579832

Length=603

***** No hits found *****

Lambda K H a alpha

0.318 0.134 0.401 0.792 4.96

Gapped

Lambda K H a alpha sigma

0.267 0.0410 0.140 1.90 42.6 43.6

Effective search space used: 28181472

**Query=** NODE_91_length_601_cov_0.578059

Length=601

***** No hits found *****

Lambda K H a alpha

0.318 0.134 0.401 0.792 4.96

Gapped

Lambda K H a alpha sigma

0.267 0.0410 0.140 1.90 42.6 43.6

Effective search space used: 27967976

**Query=** NODE_92_length_599_cov_0.59322

Length=599

***** No hits found *****

Lambda K H a alpha

0.318 0.134 0.401 0.792 4.96

Gapped

Lambda K H a alpha sigma

0.267 0.0410 0.140 1.90 42.6 43.6

Effective search space used: 27754480

**Query=** NODE_93_length_598_cov_0.700637

Length=598

***** No hits found *****

Lambda K H a alpha

0.318 0.134 0.401 0.792 4.96

Gapped

Lambda K H a alpha sigma

0.267 0.0410 0.140 1.90 42.6 43.6

Effective search space used: 27754480

**Query=** NODE_94_length_598_cov_0.477707

Length=598

***** No hits found *****

Lambda K H a alpha

0.318 0.134 0.401 0.792 4.96

Gapped

Lambda K H a alpha sigma

0.267 0.0410 0.140 1.90 42.6 43.6

Effective search space used: 27754480

**Query=** NODE_95_length_595_cov_0.209402

Length=595

***** No hits found *****

Lambda K H a alpha

0.318 0.134 0.401 0.792 4.96

Gapped

Lambda K H a alpha sigma

0.267 0.0410 0.140 1.90 42.6 43.6

Effective search space used: 27540984

**Query=** NODE_96_length_593_cov_0.746781

Length=593

***** No hits found *****

Lambda K H a alpha

0.318 0.134 0.401 0.792 4.96

Gapped

Lambda K H a alpha sigma

0.267 0.0410 0.140 1.90 42.6 43.6

Effective search space used: 27327488

**Query=** NODE_97_length_592_cov_0.72043

Length=592

***** No hits found *****

Lambda K H a alpha

0.318 0.134 0.401 0.792 4.96

Gapped

Lambda K H a alpha sigma

0.267 0.0410 0.140 1.90 42.6 43.6

Effective search space used: 27327488

**Query=** NODE_98_length_589_cov_0.67316

Length=589

***** No hits found *****

Lambda K H a alpha

0.318 0.134 0.401 0.792 4.96

Gapped

Lambda K H a alpha sigma

0.267 0.0410 0.140 1.90 42.6 43.6

Effective search space used: 27113992

**Query=** NODE_99_length_588_cov_0.362256

Length=588

***** No hits found *****

Lambda K H a alpha

0.318 0.134 0.401 0.792 4.96

Gapped

Lambda K H a alpha sigma

0.267 0.0410 0.140 1.90 42.6 43.6

Effective search space used: 27113992

**Query=** NODE_100_length_588_cov_0.509761

Length=588

***** No hits found *****

Lambda K H a alpha

0.318 0.134 0.401 0.792 4.96

Gapped

Lambda K H a alpha sigma

0.267 0.0410 0.140 1.90 42.6 43.6

Effective search space used: 27113992

**Query=** NODE_101_length_585_cov_0.451965

Length=585

***** No hits found *****

Lambda K H a alpha

0.318 0.134 0.401 0.792 4.96

Gapped

Lambda K H a alpha sigma

0.267 0.0410 0.140 1.90 42.6 43.6

Effective search space used: 26900496

**Query=** NODE_102_length_581_cov_0.577093

Length=581

***** No hits found *****

Lambda K H a alpha

0.318 0.134 0.401 0.792 4.96

Gapped

Lambda K H a alpha sigma

0.267 0.0410 0.140 1.90 42.6 43.6

Effective search space used: 26774750

**Query=** NODE_103_length_575_cov_0.258929

Length=575

***** No hits found *****

Lambda K H a alpha

0.318 0.134 0.401 0.792 4.96

Gapped

Lambda K H a alpha sigma

0.267 0.0410 0.140 1.90 42.6 43.6

Effective search space used: 26346354

**Query=** NODE_104_length_574_cov_0.494407

Length=574

***** No hits found *****

Lambda K H a alpha

0.318 0.134 0.401 0.792 4.96

Gapped

Lambda K H a alpha sigma

0.267 0.0410 0.140 1.90 42.6 43.6

Effective search space used: 26346354

**Query=** NODE_105_length_572_cov_0.649438

Length=572

***** No hits found *****

Lambda K H a alpha

0.318 0.134 0.401 0.792 4.96

Gapped

Lambda K H a alpha sigma

0.267 0.0410 0.140 1.90 42.6 43.6

Effective search space used: 26132156

**Query=** NODE_106_length_572_cov_1.70112

Length=572

***** No hits found *****

Lambda K H a alpha

0.318 0.134 0.401 0.792 4.96

Gapped

Lambda K H a alpha sigma

0.267 0.0410 0.140 1.90 42.6 43.6

Effective search space used: 26132156

**Query=** NODE_107_length_572_cov_0.550562

Length=572

***** No hits found *****

Lambda K H a alpha

0.318 0.134 0.401 0.792 4.96

Gapped

Lambda K H a alpha sigma

0.267 0.0410 0.140 1.90 42.6 43.6

Effective search space used: 26132156

**Query=** NODE_108_length_567_cov_0.745455

Length=567

***** No hits found *****

Lambda K H a alpha

0.318 0.134 0.401 0.792 4.96

Gapped

Lambda K H a alpha sigma

0.267 0.0410 0.140 1.90 42.6 43.6

Effective search space used: 25917958

**Query=** NODE_109_length_566_cov_0.715262

Length=566

***** No hits found *****

Lambda K H a alpha

0.318 0.134 0.401 0.792 4.96

Gapped

Lambda K H a alpha sigma

0.267 0.0410 0.140 1.90 42.6 43.6

Effective search space used: 25703760

**Query=** NODE_110_length_566_cov_0.346241

Length=566

***** No hits found *****

Lambda K H a alpha

0.318 0.134 0.401 0.792 4.96

Gapped

Lambda K H a alpha sigma

0.267 0.0410 0.140 1.90 42.6 43.6

Effective search space used: 25703760

**Query=** NODE_111_length_563_cov_0.688073

Length=563

***** No hits found *****

Lambda K H a alpha

0.318 0.134 0.401 0.792 4.96

Gapped

Lambda K H a alpha sigma

0.267 0.0410 0.140 1.90 42.6 43.6

Effective search space used: 25489562

**Query=** NODE_112_length_562_cov_0.652874

Length=562

***** No hits found *****

Lambda K H a alpha

0.318 0.134 0.401 0.792 4.96

Gapped

Lambda K H a alpha sigma

0.267 0.0410 0.140 1.90 42.6 43.6

Effective search space used: 25489562

**Query=** NODE_113_length_562_cov_0.531034

Length=562

***** No hits found *****

Lambda K H a alpha

0.318 0.134 0.401 0.792 4.96

Gapped

Lambda K H a alpha sigma

0.267 0.0410 0.140 1.90 42.6 43.6

Effective search space used: 25489562

**Query=** NODE_114_length_561_cov_0.476959

Length=561

***** No hits found *****

Lambda K H a alpha

0.318 0.134 0.401 0.792 4.96

Gapped

Lambda K H a alpha sigma

0.267 0.0410 0.140 1.90 42.6 43.6

Effective search space used: 25489562

**Query=** NODE_115_length_560_cov_0.766744

Length=560

***** No hits found *****

Lambda K H a alpha

0.318 0.134 0.401 0.792 4.96

Gapped

Lambda K H a alpha sigma

0.267 0.0410 0.140 1.90 42.6 43.6

Effective search space used: 25275364

**Query=** NODE_116_length_560_cov_0.644342

Length=560

***** No hits found *****

Lambda K H a alpha

0.318 0.134 0.401 0.792 4.96

Gapped

Lambda K H a alpha sigma

0.267 0.0410 0.140 1.90 42.6 43.6

Effective search space used: 25275364

**Query=** NODE_117_length_560_cov_0.752887

Length=560

***** No hits found *****

Lambda K H a alpha

0.318 0.134 0.401 0.792 4.96

Gapped

Lambda K H a alpha sigma

0.267 0.0410 0.140 1.90 42.6 43.6

Effective search space used: 25275364

**Query=** NODE_118_length_559_cov_0.587963

Length=559

***** No hits found *****

Lambda K H a alpha

0.318 0.134 0.401 0.792 4.96

Gapped

Lambda K H a alpha sigma

0.267 0.0410 0.140 1.90 42.6 43.6

Effective search space used: 25275364

**Query=** NODE_119_length_559_cov_0.75463

Length=559

***** No hits found *****

Lambda K H a alpha

0.318 0.134 0.401 0.792 4.96

Gapped

Lambda K H a alpha sigma

0.267 0.0410 0.140 1.90 42.6 43.6

Effective search space used: 25275364

**Query=** NODE_120_length_559_cov_0.333333

Length=559

***** No hits found *****

Lambda K H a alpha

0.318 0.134 0.401 0.792 4.96

Gapped

Lambda K H a alpha sigma

0.267 0.0410 0.140 1.90 42.6 43.6

Effective search space used: 25275364

**Query=** NODE_121_length_558_cov_0.440835

Length=558

***** No hits found *****

Lambda K H a alpha

0.318 0.134 0.401 0.792 4.96

Gapped

Lambda K H a alpha sigma

0.267 0.0410 0.140 1.90 42.6 43.6

Effective search space used: 25275364

**Query=** NODE_122_length_556_cov_0.424242

Length=556

***** No hits found *****

Lambda K H a alpha

0.318 0.134 0.401 0.792 4.96

Gapped

Lambda K H a alpha sigma

0.267 0.0410 0.140 1.90 42.6 43.6

Effective search space used: 25061166

**Query=** NODE_123_length_555_cov_0.495327

Length=555

***** No hits found *****

Lambda K H a alpha

0.318 0.134 0.401 0.792 4.96

Gapped

Lambda K H a alpha sigma

0.267 0.0410 0.140 1.90 42.6 43.6

Effective search space used: 25061166

**Query=** NODE_124_length_555_cov_0.371495

Length=555

***** No hits found *****

Lambda K H a alpha

0.318 0.134 0.401 0.792 4.96

Gapped

Lambda K H a alpha sigma

0.267 0.0410 0.140 1.90 42.6 43.6

Effective search space used: 25061166

**Query=** NODE_125_length_554_cov_0.606557

Length=554

***** No hits found *****

Lambda K H a alpha

0.318 0.134 0.401 0.792 4.96

Gapped

Lambda K H a alpha sigma

0.267 0.0410 0.140 1.90 42.6 43.6

Effective search space used: 24846968

**Query=** NODE_126_length_554_cov_0.75644

Length=554

***** No hits found *****

Lambda K H a alpha

0.318 0.134 0.401 0.792 4.96

Gapped

Lambda K H a alpha sigma

0.267 0.0410 0.140 1.90 42.6 43.6

Effective search space used: 24846968

**Query=** NODE_127_length_554_cov_0.29274

Length=554

***** No hits found *****

Lambda K H a alpha

0.318 0.134 0.401 0.792 4.96

Gapped

Lambda K H a alpha sigma

0.267 0.0410 0.140 1.90 42.6 43.6

Effective search space used: 24846968

**Query=** NODE_128_length_551_cov_0.389151

Length=551

***** No hits found *****

Lambda K H a alpha

0.318 0.134 0.401 0.792 4.96

Gapped

Lambda K H a alpha sigma

0.267 0.0410 0.140 1.90 42.6 43.6

Effective search space used: 24632770

**Query=** NODE_129_length_551_cov_0.575472

Length=551

***** No hits found *****

Lambda K H a alpha

0.318 0.134 0.401 0.792 4.96

Gapped

Lambda K H a alpha sigma

0.267 0.0410 0.140 1.90 42.6 43.6

Effective search space used: 24632770

**Query=** NODE_130_length_550_cov_0.801418

Length=550

***** No hits found *****

Lambda K H a alpha

0.318 0.134 0.401 0.792 4.96

Gapped

Lambda K H a alpha sigma

0.267 0.0410 0.140 1.90 42.6 43.6

Effective search space used: 24632770

**Query=** NODE_131_length_550_cov_0.782506

Length=550

***** No hits found *****

Lambda K H a alpha

0.318 0.134 0.401 0.792 4.96

Gapped

Lambda K H a alpha sigma

0.267 0.0410 0.140 1.90 42.6 43.6

Effective search space used: 24632770

**Query=** NODE_132_length_549_cov_0.7891

Length=549

***** No hits found *****

Lambda K H a alpha

0.318 0.134 0.401 0.792 4.96

Gapped

Lambda K H a alpha sigma

0.267 0.0410 0.140 1.90 42.6 43.6

Effective search space used: 24632770

**Query=** NODE_133_length_547_cov_0.442857

Length=547

***** No hits found *****

Lambda K H a alpha

0.318 0.134 0.401 0.792 4.96

Gapped

Lambda K H a alpha sigma

0.267 0.0410 0.140 1.90 42.6 43.6

Effective search space used: 24418572

**Query=** NODE_134_length_544_cov_0.35012

Length=544

***** No hits found *****

Lambda K H a alpha

0.318 0.134 0.401 0.792 4.96

Gapped

Lambda K H a alpha sigma

0.267 0.0410 0.140 1.90 42.6 43.6

Effective search space used: 24204374

**Query=** NODE_135_length_541_cov_0.676329

Length=541

***** No hits found *****

Lambda K H a alpha

0.318 0.134 0.401 0.792 4.96

Gapped

Lambda K H a alpha sigma

0.267 0.0410 0.140 1.90 42.6 43.6

Effective search space used: 23990176

**Query=** NODE_136_length_540_cov_0.690073

Length=540

***** No hits found *****

Lambda K H a alpha

0.318 0.134 0.401 0.792 4.96

Gapped

Lambda K H a alpha sigma

0.267 0.0410 0.140 1.90 42.6 43.6

Effective search space used: 23990176

**Query=** NODE_137_length_540_cov_0.539952

Length=540

***** No hits found *****

Lambda K H a alpha

0.317 0.136 0.468 0.792 4.96

Gapped

Lambda K H a alpha sigma

0.267 0.0410 0.140 1.90 42.6 43.6

Effective search space used: 23990176

**Query=** NODE_138_length_539_cov_0.497573

Length=539

Score E

Sequences producing significant alignments: (Bits) Value

ARA90579 phenicol and oxazolidinone resistance protein [Staphyl... [25.4](file:///Users/JMBNew/Desktop/Articles%20en%20Cours/Article%20Diane/Bb_42-1_output-blast_named.html#BL_ORD_ID:288) 0.57

AQW34744 phenicol and oxazolidinone resistance protein [Staphyl... [25.4](file:///Users/JMBNew/Desktop/Articles%20en%20Cours/Article%20Diane/Bb_42-1_output-blast_named.html#BL_ORD_ID:278) 0.57

AQW34730 phenicol and oxazolidinone resistance protein [Staphyl... [25.4](file:///Users/JMBNew/Desktop/Articles%20en%20Cours/Article%20Diane/Bb_42-1_output-blast_named.html#BL_ORD_ID:274) 0.57

AQW34667 ABC transporter ATP-binding protein (plasmid) [Staphyl... [25.4](file:///Users/JMBNew/Desktop/Articles%20en%20Cours/Article%20Diane/Bb_42-1_output-blast_named.html#BL_ORD_ID:259) 0.57

AQW34661 phenicol and oxazolidinone resistance protein [Staphyl... [25.4](file:///Users/JMBNew/Desktop/Articles%20en%20Cours/Article%20Diane/Bb_42-1_output-blast_named.html#BL_ORD_ID:256) 0.57

AQW34617 OptrA (plasmid) [Staphylococcus sciuri] [25.4](file:///Users/JMBNew/Desktop/Articles%20en%20Cours/Article%20Diane/Bb_42-1_output-blast_named.html#BL_ORD_ID:249) 0.57

AQW34612 phenicol and oxazolidinone resistance protein [Staphyl... [25.4](file:///Users/JMBNew/Desktop/Articles%20en%20Cours/Article%20Diane/Bb_42-1_output-blast_named.html#BL_ORD_ID:246) 0.57

AQW34601 phenicol and oxazolidinone resistance protein [Staphyl... [25.4](file:///Users/JMBNew/Desktop/Articles%20en%20Cours/Article%20Diane/Bb_42-1_output-blast_named.html#BL_ORD_ID:245) 0.57

AQW34567 ABC transporter ATP-binding protein [Staphylococcus sc... [25.4](file:///Users/JMBNew/Desktop/Articles%20en%20Cours/Article%20Diane/Bb_42-1_output-blast_named.html#BL_ORD_ID:238) 0.57

> ARA90579 phenicol and oxazolidinone resistance protein [Staphylococcus

sciuri]

Length=655

Score = 25.4 bits (54), Expect = 0.57, Method: Composition-based stats.

Identities = 11/19 (58%), Positives = 11/19 (58%), Gaps = 0/19 (0%)

Frame = -1

Query 200 FSRGSMTAIVGPNGAGKST 144

RG IVG NG GKST

Sbjct 368 LERGQKLGIVGSNGIGKST 386

> AQW34744 phenicol and oxazolidinone resistance protein [Staphylococcus

sciuri]

Length=655

Score = 25.4 bits (54), Expect = 0.57, Method: Composition-based stats.

Identities = 11/19 (58%), Positives = 11/19 (58%), Gaps = 0/19 (0%)

Frame = -1

Query 200 FSRGSMTAIVGPNGAGKST 144

RG IVG NG GKST

Sbjct 368 LERGQKLGIVGSNGIGKST 386

> AQW34730 phenicol and oxazolidinone resistance protein [Staphylococcus

sciuri]

Length=655

Score = 25.4 bits (54), Expect = 0.57, Method: Composition-based stats.

Identities = 11/19 (58%), Positives = 11/19 (58%), Gaps = 0/19 (0%)

Frame = -1

Query 200 FSRGSMTAIVGPNGAGKST 144

RG IVG NG GKST

Sbjct 368 LERGQKLGIVGSNGIGKST 386

> AQW34667 ABC transporter ATP-binding protein (plasmid) [Staphylococcus

sciuri]

Length=655

Score = 25.4 bits (54), Expect = 0.57, Method: Composition-based stats.

Identities = 11/19 (58%), Positives = 11/19 (58%), Gaps = 0/19 (0%)

Frame = -1

Query 200 FSRGSMTAIVGPNGAGKST 144

RG IVG NG GKST

Sbjct 368 LERGQKLGIVGSNGIGKST 386

> AQW34661 phenicol and oxazolidinone resistance protein [Staphylococcus

sciuri]

Length=655

Score = 25.4 bits (54), Expect = 0.57, Method: Composition-based stats.

Identities = 11/19 (58%), Positives = 11/19 (58%), Gaps = 0/19 (0%)

Frame = -1

Query 200 FSRGSMTAIVGPNGAGKST 144

RG IVG NG GKST

Sbjct 368 LERGQKLGIVGSNGIGKST 386

> AQW34617 OptrA (plasmid) [Staphylococcus sciuri]

Length=655

Score = 25.4 bits (54), Expect = 0.57, Method: Composition-based stats.

Identities = 11/19 (58%), Positives = 11/19 (58%), Gaps = 0/19 (0%)

Frame = -1

Query 200 FSRGSMTAIVGPNGAGKST 144

RG IVG NG GKST

Sbjct 368 LERGQKLGIVGSNGIGKST 386

> AQW34612 phenicol and oxazolidinone resistance protein [Staphylococcus

sciuri]

Length=655

Score = 25.4 bits (54), Expect = 0.57, Method: Composition-based stats.

Identities = 11/19 (58%), Positives = 11/19 (58%), Gaps = 0/19 (0%)

Frame = -1

Query 200 FSRGSMTAIVGPNGAGKST 144

RG IVG NG GKST

Sbjct 368 LERGQKLGIVGSNGIGKST 386

> AQW34601 phenicol and oxazolidinone resistance protein [Staphylococcus

sciuri]

Length=655

Score = 25.4 bits (54), Expect = 0.57, Method: Composition-based stats.

Identities = 11/19 (58%), Positives = 11/19 (58%), Gaps = 0/19 (0%)

Frame = -1

Query 200 FSRGSMTAIVGPNGAGKST 144

RG IVG NG GKST

Sbjct 368 LERGQKLGIVGSNGIGKST 386

> AQW34567 ABC transporter ATP-binding protein [Staphylococcus

sciuri]

Length=655

Score = 25.4 bits (54), Expect = 0.57, Method: Composition-based stats.

Identities = 11/19 (58%), Positives = 11/19 (58%), Gaps = 0/19 (0%)

Frame = -1

Query 200 FSRGSMTAIVGPNGAGKST 144

RG IVG NG GKST

Sbjct 368 LERGQKLGIVGSNGIGKST 386

Lambda K H a alpha

0.318 0.134 0.401 0.792 4.96

Gapped

Lambda K H a alpha sigma

0.267 0.0410 0.140 1.90 42.6 43.6

Effective search space used: 23775978

**Query=** NODE_139_length_538_cov_0.744526

Length=538

Score E

Sequences producing significant alignments: (Bits) Value

AEO37498 esterase EstDL136 [uncultured bacterium pDL136] [25.0](file:///Users/JMBNew/Desktop/Articles%20en%20Cours/Article%20Diane/Bb_42-1_output-blast_named.html#BL_ORD_ID:82) 0.80

>tr|G3CR02|G3CR02_estDL136 estDL136 [25.0](file:///Users/JMBNew/Desktop/Articles%20en%20Cours/Article%20Diane/Bb_42-1_output-blast_named.html#BL_ORD_ID:0) 0.80

> AEO37498 esterase EstDL136 [uncultured bacterium pDL136]

Length=310

Score = 25.0 bits (53), Expect = 0.80, Method: Compositional matrix adjust.

Identities = 10/20 (50%), Positives = 14/20 (70%), Gaps = 0/20 (0%)

Frame = +2

Query 299 PLHRHSESLAQLNGQHPAPE 358

PL+ H E+L Q+ Q PAP+

Sbjct 2 PLNPHVEALLQMMAQMPAPD 21

> >tr|G3CR02|G3CR02_estDL136 estDL136

Length=310

Score = 25.0 bits (53), Expect = 0.80, Method: Compositional matrix adjust.

Identities = 10/20 (50%), Positives = 14/20 (70%), Gaps = 0/20 (0%)

Frame = +2

Query 299 PLHRHSESLAQLNGQHPAPE 358

PL+ H E+L Q+ Q PAP+

Sbjct 2 PLNPHVEALLQMMAQMPAPD 21

Lambda K H a alpha

0.318 0.134 0.401 0.792 4.96

Gapped

Lambda K H a alpha sigma

0.267 0.0410 0.140 1.90 42.6 43.6

Effective search space used: 23775978

**Query=** NODE_140_length_537_cov_1.69024

Length=537

***** No hits found *****

Lambda K H a alpha

0.318 0.134 0.401 0.792 4.96

Gapped

Lambda K H a alpha sigma

0.267 0.0410 0.140 1.90 42.6 43.6

Effective search space used: 23775978

**Query=** NODE_141_length_536_cov_0.481663

Length=536

***** No hits found *****

Lambda K H a alpha

0.318 0.134 0.401 0.792 4.96

Gapped

Lambda K H a alpha sigma

0.267 0.0410 0.140 1.90 42.6 43.6

Effective search space used: 23561780

**Query=** NODE_142_length_535_cov_0.502451

Length=535

***** No hits found *****

Lambda K H a alpha

0.318 0.134 0.401 0.792 4.96

Gapped

Lambda K H a alpha sigma

0.267 0.0410 0.140 1.90 42.6 43.6

Effective search space used: 23561780

**Query=** NODE_143_length_535_cov_0.5

Length=535

***** No hits found *****

Lambda K H a alpha

0.318 0.134 0.401 0.792 4.96

Gapped

Lambda K H a alpha sigma

0.267 0.0410 0.140 1.90 42.6 43.6

Effective search space used: 23561780

**Query=** NODE_144_length_535_cov_0.448529

Length=535

***** No hits found *****

Lambda K H a alpha

0.318 0.134 0.401 0.792 4.96

Gapped

Lambda K H a alpha sigma

0.267 0.0410 0.140 1.90 42.6 43.6

Effective search space used: 23561780

**Query=** NODE_145_length_534_cov_0.739558

Length=534

***** No hits found *****

Lambda K H a alpha

0.318 0.134 0.401 0.792 4.96

Gapped

Lambda K H a alpha sigma

0.267 0.0410 0.140 1.90 42.6 43.6

Effective search space used: 23561780

**Query=** NODE_146_length_534_cov_0.638821

Length=534

***** No hits found *****

Lambda K H a alpha

0.318 0.134 0.401 0.792 4.96

Gapped

Lambda K H a alpha sigma

0.267 0.0410 0.140 1.90 42.6 43.6

Effective search space used: 23561780

**Query=** NODE_147_length_534_cov_0.476658

Length=534

***** No hits found *****

Lambda K H a alpha

0.318 0.134 0.401 0.792 4.96

Gapped

Lambda K H a alpha sigma

0.267 0.0410 0.140 1.90 42.6 43.6

Effective search space used: 23561780

**Query=** NODE_148_length_534_cov_0.211302

Length=534

***** No hits found *****

Lambda K H a alpha

0.318 0.134 0.401 0.792 4.96

Gapped

Lambda K H a alpha sigma

0.267 0.0410 0.140 1.90 42.6 43.6

Effective search space used: 23561780

**Query=** NODE_149_length_533_cov_0.593596

Length=533

***** No hits found *****

Lambda K H a alpha

0.318 0.134 0.401 0.792 4.96

Gapped

Lambda K H a alpha sigma

0.267 0.0410 0.140 1.90 42.6 43.6

Effective search space used: 23347582

**Query=** NODE_150_length_533_cov_0.743842

Length=533

***** No hits found *****

Lambda K H a alpha

0.318 0.134 0.401 0.792 4.96

Gapped

Lambda K H a alpha sigma

0.267 0.0410 0.140 1.90 42.6 43.6

Effective search space used: 23347582

**Query=** NODE_151_length_531_cov_0.601485

Length=531

***** No hits found *****

Lambda K H a alpha

0.318 0.134 0.401 0.792 4.96

Gapped

Lambda K H a alpha sigma

0.267 0.0410 0.140 1.90 42.6 43.6

Effective search space used: 23347582

**Query=** NODE_152_length_530_cov_0.243176

Length=530

***** No hits found *****

Lambda K H a alpha

0.318 0.134 0.401 0.792 4.96

Gapped

Lambda K H a alpha sigma

0.267 0.0410 0.140 1.90 42.6 43.6

Effective search space used: 23424100

**Query=** NODE_153_length_529_cov_0.4801

Length=529

***** No hits found *****

Lambda K H a alpha

0.318 0.134 0.401 0.792 4.96

Gapped

Lambda K H a alpha sigma

0.267 0.0410 0.140 1.90 42.6 43.6

Effective search space used: 23424100

**Query=** NODE_154_length_528_cov_0.700748

Length=528

***** No hits found *****

Lambda K H a alpha

0.318 0.134 0.401 0.792 4.96

Gapped

Lambda K H a alpha sigma

0.267 0.0410 0.140 1.90 42.6 43.6

Effective search space used: 23424100

**Query=** NODE_155_length_528_cov_0.703242

Length=528

***** No hits found *****

Lambda K H a alpha

0.318 0.134 0.401 0.792 4.96

Gapped

Lambda K H a alpha sigma

0.267 0.0410 0.140 1.90 42.6 43.6

Effective search space used: 23424100

**Query=** NODE_156_length_528_cov_0.65586

Length=528

***** No hits found *****

Lambda K H a alpha

0.318 0.134 0.401 0.792 4.96

Gapped

Lambda K H a alpha sigma

0.267 0.0410 0.140 1.90 42.6 43.6

Effective search space used: 23424100

**Query=** NODE_157_length_525_cov_0.432161

Length=525

***** No hits found *****

Lambda K H a alpha

0.318 0.134 0.401 0.792 4.96

Gapped

Lambda K H a alpha sigma

0.267 0.0410 0.140 1.90 42.6 43.6

Effective search space used: 23209200

**Query=** NODE_158_length_524_cov_0.43073

Length=524

***** No hits found *****

Lambda K H a alpha

0.318 0.134 0.401 0.792 4.96

Gapped

Lambda K H a alpha sigma

0.267 0.0410 0.140 1.90 42.6 43.6

Effective search space used: 22994300

**Query=** NODE_159_length_524_cov_0.506297

Length=524

***** No hits found *****

Lambda K H a alpha

0.318 0.134 0.401 0.792 4.96

Gapped

Lambda K H a alpha sigma

0.267 0.0410 0.140 1.90 42.6 43.6

Effective search space used: 22994300

**Query=** NODE_160_length_523_cov_0.431818

Length=523

***** No hits found *****

Lambda K H a alpha

0.318 0.134 0.401 0.792 4.96

Gapped

Lambda K H a alpha sigma

0.267 0.0410 0.140 1.90 42.6 43.6

Effective search space used: 22994300

**Query=** NODE_161_length_523_cov_0.762626

Length=523

***** No hits found *****

Lambda K H a alpha

0.318 0.134 0.401 0.792 4.96

Gapped

Lambda K H a alpha sigma

0.267 0.0410 0.140 1.90 42.6 43.6

Effective search space used: 22994300

**Query=** NODE_162_length_522_cov_0.675949

Length=522

***** No hits found *****

Lambda K H a alpha

0.318 0.134 0.401 0.792 4.96

Gapped

Lambda K H a alpha sigma

0.267 0.0410 0.140 1.90 42.6 43.6

Effective search space used: 22994300

**Query=** NODE_163_length_521_cov_0.733503

Length=521

***** No hits found *****

Lambda K H a alpha

0.318 0.134 0.401 0.792 4.96

Gapped

Lambda K H a alpha sigma

0.267 0.0410 0.140 1.90 42.6 43.6

Effective search space used: 22779400

**Query=** NODE_164_length_521_cov_0.687817

Length=521

***** No hits found *****

Lambda K H a alpha

0.318 0.134 0.401 0.792 4.96

Gapped

Lambda K H a alpha sigma

0.267 0.0410 0.140 1.90 42.6 43.6

Effective search space used: 22779400

**Query=** NODE_165_length_520_cov_0.531807

Length=520

***** No hits found *****

Lambda K H a alpha

0.318 0.134 0.401 0.792 4.96

Gapped

Lambda K H a alpha sigma

0.267 0.0410 0.140 1.90 42.6 43.6

Effective search space used: 22779400

**Query=** NODE_166_length_514_cov_0.731266

Length=514

***** No hits found *****

Lambda K H a alpha

0.318 0.134 0.401 0.792 4.96

Gapped

Lambda K H a alpha sigma

0.267 0.0410 0.140 1.90 42.6 43.6

Effective search space used: 22349600

**Query=** NODE_167_length_514_cov_0.736434

Length=514

***** No hits found *****

Lambda K H a alpha

0.318 0.134 0.401 0.792 4.96

Gapped

Lambda K H a alpha sigma

0.267 0.0410 0.140 1.90 42.6 43.6

Effective search space used: 22349600

**Query=** NODE_168_length_513_cov_0.375648

Length=513

***** No hits found *****

Lambda K H a alpha

0.318 0.134 0.401 0.792 4.96

Gapped

Lambda K H a alpha sigma

0.267 0.0410 0.140 1.90 42.6 43.6

Effective search space used: 22349600

**Query=** NODE_169_length_511_cov_0

Length=511

***** No hits found *****

Lambda K H a alpha

0.318 0.134 0.401 0.792 4.96

Gapped

Lambda K H a alpha sigma

0.267 0.0410 0.140 1.90 42.6 43.6

Effective search space used: 22134700

**Query=** NODE_170_length_511_cov_0.664062

Length=511

***** No hits found *****

Lambda K H a alpha

0.318 0.134 0.401 0.792 4.96

Gapped

Lambda K H a alpha sigma

0.267 0.0410 0.140 1.90 42.6 43.6

Effective search space used: 22134700

**Query=** NODE_171_length_511_cov_0.723958

Length=511

***** No hits found *****

Lambda K H a alpha

0.318 0.134 0.401 0.792 4.96

Gapped

Lambda K H a alpha sigma

0.267 0.0410 0.140 1.90 42.6 43.6

Effective search space used: 22134700

**Query=** NODE_172_length_510_cov_0.809399

Length=510

***** No hits found *****

Lambda K H a alpha

0.318 0.134 0.401 0.792 4.96

Gapped

Lambda K H a alpha sigma

0.267 0.0410 0.140 1.90 42.6 43.6

Effective search space used: 22134700

**Query=** NODE_173_length_508_cov_0.133858

Length=508

***** No hits found *****

Lambda K H a alpha

0.318 0.134 0.401 0.792 4.96

Gapped

Lambda K H a alpha sigma

0.267 0.0410 0.140 1.90 42.6 43.6

Effective search space used: 21919800

**Query=** NODE_174_length_507_cov_0.784211

Length=507

***** No hits found *****

Lambda K H a alpha

0.318 0.134 0.401 0.792 4.96

Gapped

Lambda K H a alpha sigma

0.267 0.0410 0.140 1.90 42.6 43.6

Effective search space used: 21919800

**Query=** NODE_175_length_507_cov_0.742105

Length=507

***** No hits found *****

Lambda K H a alpha

0.318 0.134 0.401 0.792 4.96

Gapped

Lambda K H a alpha sigma

0.267 0.0410 0.140 1.90 42.6 43.6

Effective search space used: 21919800

**Query=** NODE_176_length_507_cov_0.810526

Length=507

Score E

Sequences producing significant alignments: (Bits) Value

XP_002341612 florfenicol exporter, putative [Talaromyces stipit... [26.2](file:///Users/JMBNew/Desktop/Articles%20en%20Cours/Article%20Diane/Bb_42-1_output-blast_named.html#BL_ORD_ID:580) 0.31

EED24225 florfenicol exporter, putative [Talaromyces stipitatus... [26.2](file:///Users/JMBNew/Desktop/Articles%20en%20Cours/Article%20Diane/Bb_42-1_output-blast_named.html#BL_ORD_ID:426) 0.31

> XP_002341612 florfenicol exporter, putative [Talaromyces stipitatus

ATCC 10500]

Length=393

Score = 26.2 bits (56), Expect = 0.31, Method: Compositional matrix adjust.

Identities = 15/52 (29%), Positives = 26/52 (50%), Gaps = 9/52 (17%)

Frame = -3

Query 277 SRVVVATVFVFQPDTADESTTVLTVPVSN------YHFPPPQ---NTQKTPN 149

S +V++ + +F P+T +VP+S Y+F PP+ N Q + N

Sbjct 75 SVMVLSALLIFLPETQRSKAGNGSVPLSGFQKPLIYYFKPPKAWANYQSSGN 126

> EED24225 florfenicol exporter, putative [Talaromyces stipitatus

ATCC 10500]

Length=393

Score = 26.2 bits (56), Expect = 0.31, Method: Compositional matrix adjust.

Identities = 15/52 (29%), Positives = 26/52 (50%), Gaps = 9/52 (17%)

Frame = -3

Query 277 SRVVVATVFVFQPDTADESTTVLTVPVSN------YHFPPPQ---NTQKTPN 149

S +V++ + +F P+T +VP+S Y+F PP+ N Q + N

Sbjct 75 SVMVLSALLIFLPETQRSKAGNGSVPLSGFQKPLIYYFKPPKAWANYQSSGN 126

Lambda K H a alpha

0.318 0.134 0.401 0.792 4.96

Gapped

Lambda K H a alpha sigma

0.267 0.0410 0.140 1.90 42.6 43.6

Effective search space used: 21919800

**Query=** NODE_177_length_507_cov_0.481579

Length=507

***** No hits found *****

Lambda K H a alpha

0.318 0.134 0.401 0.792 4.96

Gapped

Lambda K H a alpha sigma

0.267 0.0410 0.140 1.90 42.6 43.6

Effective search space used: 21919800

**Query=** NODE_178_length_504_cov_0.66313

Length=504

***** No hits found *****

Lambda K H a alpha

0.318 0.134 0.401 0.792 4.96

Gapped

Lambda K H a alpha sigma

0.267 0.0410 0.140 1.90 42.6 43.6

Effective search space used: 21704900

**Query=** NODE_179_length_504_cov_0.718833

Length=504

***** No hits found *****

Lambda K H a alpha

0.318 0.134 0.401 0.792 4.96

Gapped

Lambda K H a alpha sigma

0.267 0.0410 0.140 1.90 42.6 43.6

Effective search space used: 21704900

**Query=** NODE_180_length_504_cov_0.718833

Length=504

***** No hits found *****

Lambda K H a alpha

0.318 0.134 0.401 0.792 4.96

Gapped

Lambda K H a alpha sigma

0.267 0.0410 0.140 1.90 42.6 43.6

Effective search space used: 21704900

**Query=** NODE_181_length_504_cov_0.214854

Length=504

***** No hits found *****

Lambda K H a alpha

0.318 0.134 0.401 0.792 4.96

Gapped

Lambda K H a alpha sigma

0.267 0.0410 0.140 1.90 42.6 43.6

Effective search space used: 21704900

**Query=** NODE_182_length_503_cov_0.43617

Length=503

***** No hits found *****

Lambda K H a alpha

0.318 0.134 0.401 0.792 4.96

Gapped

Lambda K H a alpha sigma

0.267 0.0410 0.140 1.90 42.6 43.6

Effective search space used: 21490000

**Query=** NODE_183_length_502_cov_0.674667

Length=502

***** No hits found *****

Lambda K H a alpha

0.318 0.134 0.401 0.792 4.96

Gapped

Lambda K H a alpha sigma

0.267 0.0410 0.140 1.90 42.6 43.6

Effective search space used: 21490000

**Query=** NODE_184_length_501_cov_0.748663

Length=501

***** No hits found *****

Lambda K H a alpha

0.318 0.134 0.401 0.792 4.96

Gapped

Lambda K H a alpha sigma

0.267 0.0410 0.140 1.90 42.6 43.6

Effective search space used: 21490000

**Query=** NODE_185_length_501_cov_0.743316

Length=501

***** No hits found *****

Lambda K H a alpha

0.318 0.134 0.401 0.792 4.96

Gapped

Lambda K H a alpha sigma

0.267 0.0410 0.140 1.90 42.6 43.6

Effective search space used: 21490000

**Query=** NODE_186_length_500_cov_0.747989

Length=500

***** No hits found *****

Lambda K H a alpha

0.318 0.134 0.401 0.792 4.96

Gapped

Lambda K H a alpha sigma

0.267 0.0410 0.140 1.90 42.6 43.6

Effective search space used: 21275100

**Query=** NODE_187_length_500_cov_0.351206

Length=500

***** No hits found *****

Lambda K H a alpha

0.316 0.137 0.455 0.792 4.96

Gapped

Lambda K H a alpha sigma

0.267 0.0410 0.140 1.90 42.6 43.6

Effective search space used: 21275100

**Query=** NODE_188_length_498_cov_0.692722

Length=498

Score E

Sequences producing significant alignments: (Bits) Value

ABO50235 23S rRNA m(2)A-2503 methyltransferase [Desulfotomaculu... [25.8](file:///Users/JMBNew/Desktop/Articles%20en%20Cours/Article%20Diane/Bb_42-1_output-blast_named.html#BL_ORD_ID:31) 0.36

> ABO50235 23S rRNA m(2)A-2503 methyltransferase [Desulfotomaculum

reducens MI-1]

Length=350

Score = 25.8 bits (55), Expect = 0.36, Method: Compositional matrix adjust.

Identities = 13/37 (35%), Positives = 17/37 (46%), Gaps = 0/37 (0%)

Frame = -1

Query 252 FCASCVAGCQDRRSPGRAYHRPDGLGADLRSRDHHGV 142

FCAS + G SPG Y + G+ + R H V

Sbjct 124 FCASTINGLVRNLSPGEIYDQVLGIQRETGERVSHIV 160

Lambda K H a alpha

0.318 0.134 0.401 0.792 4.96

Gapped

Lambda K H a alpha sigma

0.267 0.0410 0.140 1.90 42.6 43.6

Effective search space used: 21275100

**Query=** NODE_189_length_497_cov_0.591892

Length=497

***** No hits found *****

Lambda K H a alpha

0.318 0.134 0.401 0.792 4.96

Gapped

Lambda K H a alpha sigma

0.267 0.0410 0.140 1.90 42.6 43.6

Effective search space used: 21060200

**Query=** NODE_190_length_497_cov_0.343243

Length=497

***** No hits found *****

Lambda K H a alpha

0.318 0.134 0.401 0.792 4.96

Gapped

Lambda K H a alpha sigma

0.267 0.0410 0.140 1.90 42.6 43.6

Effective search space used: 21060200

**Query=** NODE_191_length_497_cov_0.462162

Length=497

Score E

Sequences producing significant alignments: (Bits) Value

VDAG_10167_-_20711630_translation chloramphenicol resistance pr... [25.0](file:///Users/JMBNew/Desktop/Articles%20en%20Cours/Article%20Diane/Bb_42-1_output-blast_named.html#BL_ORD_ID:697) 0.67

> VDAG_10167_-_20711630_translation chloramphenicol resistance

protein

Length=497

Score = 25.0 bits (53), Expect = 0.67, Method: Composition-based stats.

Identities = 8/17 (47%), Positives = 11/17 (65%), Gaps = 0/17 (0%)

Frame = -2

Query 322 PPPLFSPPHTAPQKKTE 272

PPPL PH PQ++ +

Sbjct 12 PPPLGPIPHNDPQRRRQ 28

Lambda K H a alpha

0.318 0.134 0.401 0.792 4.96

Gapped

Lambda K H a alpha sigma

0.267 0.0410 0.140 1.90 42.6 43.6

Effective search space used: 21060200

**Query=** NODE_192_length_495_cov_0.970109

Length=495

***** No hits found *****

Lambda K H a alpha

0.318 0.134 0.401 0.792 4.96

Gapped

Lambda K H a alpha sigma

0.267 0.0410 0.140 1.90 42.6 43.6

Effective search space used: 21060200

**Query=** NODE_193_length_495_cov_0.3125

Length=495

***** No hits found *****

Lambda K H a alpha

0.318 0.134 0.401 0.792 4.96

Gapped

Lambda K H a alpha sigma

0.267 0.0410 0.140 1.90 42.6 43.6

Effective search space used: 21060200

**Query=** NODE_194_length_493_cov_0.704918

Length=493

***** No hits found *****

Lambda K H a alpha

0.318 0.134 0.401 0.792 4.96

Gapped

Lambda K H a alpha sigma

0.267 0.0410 0.140 1.90 42.6 43.6

Effective search space used: 20845300

**Query=** NODE_195_length_493_cov_0.0409836

Length=493

***** No hits found *****

Lambda K H a alpha

0.318 0.134 0.401 0.792 4.96

Gapped

Lambda K H a alpha sigma

0.267 0.0410 0.140 1.90 42.6 43.6

Effective search space used: 20845300

**Query=** NODE_196_length_492_cov_0.69589

Length=492

***** No hits found *****

Lambda K H a alpha

0.318 0.134 0.401 0.792 4.96

Gapped

Lambda K H a alpha sigma

0.267 0.0410 0.140 1.90 42.6 43.6

Effective search space used: 20845300

**Query=** NODE_197_length_492_cov_0.449315

Length=492

***** No hits found *****

Lambda K H a alpha

0.318 0.134 0.401 0.792 4.96

Gapped

Lambda K H a alpha sigma

0.267 0.0410 0.140 1.90 42.6 43.6

Effective search space used: 20845300

**Query=** NODE_198_length_492_cov_0.427397

Length=492

***** No hits found *****

Lambda K H a alpha

0.318 0.134 0.401 0.792 4.96

Gapped

Lambda K H a alpha sigma

0.267 0.0410 0.140 1.90 42.6 43.6

Effective search space used: 20845300

**Query=** NODE_199_length_492_cov_0.591781

Length=492

***** No hits found *****

Lambda K H a alpha

0.318 0.134 0.401 0.792 4.96

Gapped

Lambda K H a alpha sigma

0.267 0.0410 0.140 1.90 42.6 43.6

Effective search space used: 20845300

**Query=** NODE_200_length_491_cov_0.706044

Length=491

***** No hits found *****

Lambda K H a alpha

0.318 0.134 0.401 0.792 4.96

Gapped

Lambda K H a alpha sigma

0.267 0.0410 0.140 1.90 42.6 43.6

Effective search space used: 20630400

**Query=** NODE_201_length_491_cov_0.587912

Length=491

***** No hits found *****

Lambda K H a alpha

0.318 0.134 0.401 0.792 4.96

Gapped

Lambda K H a alpha sigma

0.267 0.0410 0.140 1.90 42.6 43.6

Effective search space used: 20630400

**Query=** NODE_202_length_491_cov_0.156593

Length=491

***** No hits found *****

Lambda K H a alpha

0.318 0.134 0.401 0.792 4.96

Gapped

Lambda K H a alpha sigma

0.267 0.0410 0.140 1.90 42.6 43.6

Effective search space used: 20630400

**Query=** NODE_203_length_490_cov_3.9697

Length=490

***** No hits found *****

Lambda K H a alpha

0.318 0.134 0.401 0.792 4.96

Gapped

Lambda K H a alpha sigma

0.267 0.0410 0.140 1.90 42.6 43.6

Effective search space used: 20630400

**Query=** NODE_204_length_490_cov_0.46832

Length=490

***** No hits found *****

Lambda K H a alpha

0.318 0.134 0.401 0.792 4.96

Gapped

Lambda K H a alpha sigma

0.267 0.0410 0.140 1.90 42.6 43.6

Effective search space used: 20630400

**Query=** NODE_205_length_490_cov_0.429752

Length=490

***** No hits found *****

Lambda K H a alpha

0.318 0.134 0.401 0.792 4.96

Gapped

Lambda K H a alpha sigma

0.267 0.0410 0.140 1.90 42.6 43.6

Effective search space used: 20630400

**Query=** NODE_206_length_489_cov_0.229282

Length=489

***** No hits found *****

Lambda K H a alpha

0.318 0.134 0.401 0.792 4.96

Gapped

Lambda K H a alpha sigma

0.267 0.0410 0.140 1.90 42.6 43.6

Effective search space used: 20630400

**Query=** NODE_207_length_489_cov_0.756906

Length=489

***** No hits found *****

Lambda K H a alpha

0.318 0.134 0.401 0.792 4.96

Gapped

Lambda K H a alpha sigma

0.267 0.0410 0.140 1.90 42.6 43.6

Effective search space used: 20630400

**Query=** NODE_208_length_487_cov_0.577778

Length=487

***** No hits found *****

Lambda K H a alpha

0.318 0.134 0.401 0.792 4.96

Gapped

Lambda K H a alpha sigma

0.267 0.0410 0.140 1.90 42.6 43.6

Effective search space used: 20415500

**Query=** NODE_209_length_486_cov_0.713092

Length=486

***** No hits found *****

Lambda K H a alpha

0.318 0.134 0.401 0.792 4.96

Gapped

Lambda K H a alpha sigma

0.267 0.0410 0.140 1.90 42.6 43.6

Effective search space used: 20415500

**Query=** NODE_210_length_486_cov_0.462396

Length=486

***** No hits found *****

Lambda K H a alpha

0.318 0.134 0.401 0.792 4.96

Gapped

Lambda K H a alpha sigma

0.267 0.0410 0.140 1.90 42.6 43.6

Effective search space used: 20415500

**Query=** NODE_211_length_486_cov_0.267409

Length=486

***** No hits found *****

Lambda K H a alpha

0.318 0.134 0.401 0.792 4.96

Gapped

Lambda K H a alpha sigma

0.267 0.0410 0.140 1.90 42.6 43.6

Effective search space used: 20415500

**Query=** NODE_212_length_485_cov_0.346369

Length=485

***** No hits found *****

Lambda K H a alpha

0.318 0.134 0.401 0.792 4.96

Gapped

Lambda K H a alpha sigma

0.267 0.0410 0.140 1.90 42.6 43.6

Effective search space used: 20482190

**Query=** NODE_213_length_485_cov_0.402235

Length=485

***** No hits found *****

Lambda K H a alpha

0.318 0.134 0.401 0.792 4.96

Gapped

Lambda K H a alpha sigma

0.267 0.0410 0.140 1.90 42.6 43.6

Effective search space used: 20482190

**Query=** NODE_214_length_482_cov_0.735211

Length=482

***** No hits found *****

Lambda K H a alpha

0.318 0.134 0.401 0.792 4.96

Gapped

Lambda K H a alpha sigma

0.267 0.0410 0.140 1.90 42.6 43.6

Effective search space used: 20266588

**Query=** NODE_215_length_481_cov_0.720339

Length=481

***** No hits found *****

Lambda K H a alpha

0.318 0.134 0.401 0.792 4.96

Gapped

Lambda K H a alpha sigma

0.267 0.0410 0.140 1.90 42.6 43.6

Effective search space used: 20266588

**Query=** NODE_216_length_481_cov_0.847458

Length=481

***** No hits found *****

Lambda K H a alpha

0.318 0.134 0.401 0.792 4.96

Gapped

Lambda K H a alpha sigma

0.267 0.0410 0.140 1.90 42.6 43.6

Effective search space used: 20266588

**Query=** NODE_217_length_481_cov_0.59887

Length=481

***** No hits found *****

Lambda K H a alpha

0.318 0.134 0.401 0.792 4.96

Gapped

Lambda K H a alpha sigma

0.267 0.0410 0.140 1.90 42.6 43.6

Effective search space used: 20266588

**Query=** NODE_218_length_481_cov_0.700565

Length=481

***** No hits found *****

Lambda K H a alpha

0.318 0.134 0.401 0.792 4.96

Gapped

Lambda K H a alpha sigma

0.267 0.0410 0.140 1.90 42.6 43.6

Effective search space used: 20266588

**Query=** NODE_219_length_480_cov_0.498584

Length=480

***** No hits found *****

Lambda K H a alpha

0.318 0.134 0.401 0.792 4.96

Gapped

Lambda K H a alpha sigma

0.267 0.0410 0.140 1.90 42.6 43.6

Effective search space used: 20266588

**Query=** NODE_220_length_478_cov_0.729345

Length=478

***** No hits found *****

Lambda K H a alpha

0.318 0.134 0.401 0.792 4.96

Gapped

Lambda K H a alpha sigma

0.267 0.0410 0.140 1.90 42.6 43.6

Effective search space used: 20050986

**Query=** NODE_221_length_478_cov_0.700855

Length=478

***** No hits found *****

Lambda K H a alpha

0.318 0.134 0.401 0.792 4.96

Gapped

Lambda K H a alpha sigma

0.267 0.0410 0.140 1.90 42.6 43.6

Effective search space used: 20050986

**Query=** NODE_222_length_478_cov_0.700855

Length=478

***** No hits found *****

Lambda K H a alpha

0.318 0.134 0.401 0.792 4.96

Gapped

Lambda K H a alpha sigma

0.267 0.0410 0.140 1.90 42.6 43.6

Effective search space used: 20050986

**Query=** NODE_223_length_478_cov_0.424501

Length=478

***** No hits found *****

Lambda K H a alpha

0.318 0.134 0.401 0.792 4.96

Gapped

Lambda K H a alpha sigma

0.267 0.0410 0.140 1.90 42.6 43.6

Effective search space used: 20050986

**Query=** NODE_224_length_477_cov_0.702857

Length=477

***** No hits found *****

Lambda K H a alpha

0.318 0.134 0.401 0.792 4.96

Gapped

Lambda K H a alpha sigma

0.267 0.0410 0.140 1.90 42.6 43.6

Effective search space used: 20050986

**Query=** NODE_225_length_477_cov_0.8

Length=477

***** No hits found *****

Lambda K H a alpha

0.318 0.134 0.401 0.792 4.96

Gapped

Lambda K H a alpha sigma

0.267 0.0410 0.140 1.90 42.6 43.6

Effective search space used: 20050986

**Query=** NODE_226_length_476_cov_0.730659

Length=476

***** No hits found *****

Lambda K H a alpha

0.318 0.134 0.401 0.792 4.96

Gapped

Lambda K H a alpha sigma

0.267 0.0410 0.140 1.90 42.6 43.6

Effective search space used: 19835384

**Query=** NODE_227_length_475_cov_0.706897

Length=475

***** No hits found *****

Lambda K H a alpha

0.318 0.134 0.401 0.792 4.96

Gapped

Lambda K H a alpha sigma

0.267 0.0410 0.140 1.90 42.6 43.6

Effective search space used: 19835384

**Query=** NODE_228_length_475_cov_0.606322

Length=475

***** No hits found *****

Lambda K H a alpha

0.318 0.134 0.401 0.792 4.96

Gapped

Lambda K H a alpha sigma

0.267 0.0410 0.140 1.90 42.6 43.6

Effective search space used: 19835384

**Query=** NODE_229_length_474_cov_0.740634

Length=474

***** No hits found *****

Lambda K H a alpha

0.318 0.134 0.401 0.792 4.96

Gapped

Lambda K H a alpha sigma

0.267 0.0410 0.140 1.90 42.6 43.6

Effective search space used: 19835384

**Query=** NODE_230_length_474_cov_0.694524

Length=474

***** No hits found *****

Lambda K H a alpha

0.318 0.134 0.401 0.792 4.96

Gapped

Lambda K H a alpha sigma

0.267 0.0410 0.140 1.90 42.6 43.6

Effective search space used: 19835384

**Query=** NODE_231_length_473_cov_0.734104

Length=473

Score E

Sequences producing significant alignments: (Bits) Value

EFO31790 florfenicol resistance protein [Roseibium sp. TrichSKD4] [24.6](file:///Users/JMBNew/Desktop/Articles%20en%20Cours/Article%20Diane/Bb_42-1_output-blast_named.html#BL_ORD_ID:436) 0.79

> EFO31790 florfenicol resistance protein [Roseibium sp. TrichSKD4]

Length=282

Score = 24.6 bits (52), Expect = 0.79, Method: Compositional matrix adjust.

Identities = 13/32 (41%), Positives = 17/32 (53%), Gaps = 1/32 (3%)

Frame = +1

Query 337 NSAHAFHGAGIGPALEH-TQARARLYARTKTK 429

N HA+H IG L+H AR +Y + K K

Sbjct 2 NYRHAYHVGNIGDVLKHAVLARLIVYLQRKDK 33

Lambda K H a alpha

0.318 0.134 0.401 0.792 4.96

Gapped

Lambda K H a alpha sigma

0.267 0.0410 0.140 1.90 42.6 43.6

Effective search space used: 19619782

**Query=** NODE_232_length_473_cov_0.728324

Length=473

***** No hits found *****

Lambda K H a alpha

0.318 0.134 0.401 0.792 4.96

Gapped

Lambda K H a alpha sigma

0.267 0.0410 0.140 1.90 42.6 43.6

Effective search space used: 19619782

**Query=** NODE_233_length_473_cov_0.705202

Length=473

***** No hits found *****

Lambda K H a alpha

0.318 0.134 0.401 0.792 4.96

Gapped

Lambda K H a alpha sigma

0.267 0.0410 0.140 1.90 42.6 43.6

Effective search space used: 19619782

**Query=** NODE_234_length_473_cov_0.713873

Length=473

***** No hits found *****

Lambda K H a alpha

0.318 0.134 0.401 0.792 4.96

Gapped

Lambda K H a alpha sigma

0.267 0.0410 0.140 1.90 42.6 43.6

Effective search space used: 19619782

**Query=** NODE_235_length_472_cov_0.710145

Length=472

***** No hits found *****

Lambda K H a alpha

0.318 0.134 0.401 0.792 4.96

Gapped

Lambda K H a alpha sigma

0.267 0.0410 0.140 1.90 42.6 43.6

Effective search space used: 19619782

**Query=** NODE_236_length_472_cov_0.695652

Length=472

***** No hits found *****

Lambda K H a alpha

0.318 0.134 0.401 0.792 4.96

Gapped

Lambda K H a alpha sigma

0.267 0.0410 0.140 1.90 42.6 43.6

Effective search space used: 19619782

**Query=** NODE_237_length_471_cov_0.476744

Length=471

***** No hits found *****

Lambda K H a alpha

0.318 0.134 0.401 0.792 4.96

Gapped

Lambda K H a alpha sigma

0.267 0.0410 0.140 1.90 42.6 43.6

Effective search space used: 19619782

**Query=** NODE_238_length_471_cov_0.726744

Length=471

***** No hits found *****

Lambda K H a alpha

0.318 0.134 0.401 0.792 4.96

Gapped

Lambda K H a alpha sigma

0.267 0.0410 0.140 1.90 42.6 43.6

Effective search space used: 19619782

**Query=** NODE_239_length_471_cov_0.755814

Length=471

***** No hits found *****

Lambda K H a alpha

0.318 0.134 0.401 0.792 4.96

Gapped

Lambda K H a alpha sigma

0.267 0.0410 0.140 1.90 42.6 43.6

Effective search space used: 19619782

**Query=** NODE_240_length_469_cov_0.736842

Length=469

***** No hits found *****

Lambda K H a alpha

0.318 0.134 0.401 0.792 4.96

Gapped

Lambda K H a alpha sigma

0.267 0.0410 0.140 1.90 42.6 43.6

Effective search space used: 19404180

**Query=** NODE_241_length_469_cov_0.725146

Length=469

***** No hits found *****

Lambda K H a alpha

0.318 0.134 0.401 0.792 4.96

Gapped

Lambda K H a alpha sigma

0.267 0.0410 0.140 1.90 42.6 43.6

Effective search space used: 19404180

**Query=** NODE_242_length_469_cov_0.725146

Length=469

***** No hits found *****

Lambda K H a alpha

0.318 0.134 0.401 0.792 4.96

Gapped

Lambda K H a alpha sigma

0.267 0.0410 0.140 1.90 42.6 43.6

Effective search space used: 19404180

**Query=** NODE_243_length_468_cov_0.360704

Length=468

***** No hits found *****

Lambda K H a alpha

0.318 0.134 0.401 0.792 4.96

Gapped

Lambda K H a alpha sigma

0.267 0.0410 0.140 1.90 42.6 43.6

Effective search space used: 19404180

**Query=** NODE_244_length_468_cov_0.777126

Length=468

***** No hits found *****

Lambda K H a alpha

0.318 0.134 0.401 0.792 4.96

Gapped

Lambda K H a alpha sigma

0.267 0.0410 0.140 1.90 42.6 43.6

Effective search space used: 19404180

**Query=** NODE_245_length_468_cov_0.416422

Length=468

***** No hits found *****

Lambda K H a alpha

0.318 0.134 0.401 0.792 4.96

Gapped

Lambda K H a alpha sigma

0.267 0.0410 0.140 1.90 42.6 43.6

Effective search space used: 19404180

**Query=** NODE_246_length_467_cov_0.723529

Length=467

***** No hits found *****

Lambda K H a alpha

0.318 0.134 0.401 0.792 4.96

Gapped

Lambda K H a alpha sigma

0.267 0.0410 0.140 1.90 42.6 43.6

Effective search space used: 19188578

**Query=** NODE_247_length_467_cov_0.714706

Length=467

***** No hits found *****

Lambda K H a alpha

0.318 0.134 0.401 0.792 4.96

Gapped

Lambda K H a alpha sigma

0.267 0.0410 0.140 1.90 42.6 43.6

Effective search space used: 19188578

**Query=** NODE_248_length_466_cov_0.690265

Length=466

***** No hits found *****

Lambda K H a alpha

0.318 0.134 0.401 0.792 4.96

Gapped

Lambda K H a alpha sigma

0.267 0.0410 0.140 1.90 42.6 43.6

Effective search space used: 19188578

**Query=** NODE_249_length_466_cov_0.787611

Length=466

***** No hits found *****

Lambda K H a alpha

0.318 0.134 0.401 0.792 4.96

Gapped

Lambda K H a alpha sigma

0.267 0.0410 0.140 1.90 42.6 43.6

Effective search space used: 19188578

**Query=** NODE_250_length_465_cov_0.704142

Length=465

***** No hits found *****

Lambda K H a alpha

0.318 0.134 0.401 0.792 4.96

Gapped

Lambda K H a alpha sigma

0.267 0.0410 0.140 1.90 42.6 43.6

Effective search space used: 19188578

**Query=** NODE_251_length_465_cov_0.328402

Length=465

***** No hits found *****

Lambda K H a alpha

0.318 0.134 0.401 0.792 4.96

Gapped

Lambda K H a alpha sigma

0.267 0.0410 0.140 1.90 42.6 43.6

Effective search space used: 19188578

**Query=** NODE_252_length_464_cov_0.480712

Length=464

***** No hits found *****

Lambda K H a alpha

0.318 0.134 0.401 0.792 4.96

Gapped

Lambda K H a alpha sigma

0.267 0.0410 0.140 1.90 42.6 43.6

Effective search space used: 18972976

**Query=** NODE_253_length_464_cov_0.74184

Length=464

***** No hits found *****

Lambda K H a alpha

0.318 0.134 0.401 0.792 4.96

Gapped

Lambda K H a alpha sigma

0.267 0.0410 0.140 1.90 42.6 43.6

Effective search space used: 18972976

**Query=** NODE_254_length_464_cov_0.204748

Length=464

***** No hits found *****

Lambda K H a alpha

0.318 0.134 0.401 0.792 4.96

Gapped

Lambda K H a alpha sigma

0.267 0.0410 0.140 1.90 42.6 43.6

Effective search space used: 18972976

**Query=** NODE_255_length_464_cov_0.617211

Length=464

***** No hits found *****

Lambda K H a alpha

0.318 0.134 0.401 0.792 4.96

Gapped

Lambda K H a alpha sigma

0.267 0.0410 0.140 1.90 42.6 43.6

Effective search space used: 18972976

**Query=** NODE_256_length_464_cov_0.249258

Length=464

***** No hits found *****

Lambda K H a alpha

0.318 0.134 0.401 0.792 4.96

Gapped

Lambda K H a alpha sigma

0.267 0.0410 0.140 1.90 42.6 43.6

Effective search space used: 18972976

**Query=** NODE_257_length_463_cov_0.755952

Length=463

Score E

Sequences producing significant alignments: (Bits) Value

AEA08384 chloramphenicol transporter [Escherichia coli] [26.2](file:///Users/JMBNew/Desktop/Articles%20en%20Cours/Article%20Diane/Bb_42-1_output-blast_named.html#BL_ORD_ID:70) 0.27

XP_022383730 florfenicol exporter [Aspergillus bombycis] [25.4](file:///Users/JMBNew/Desktop/Articles%20en%20Cours/Article%20Diane/Bb_42-1_output-blast_named.html#BL_ORD_ID:595) 0.49

OGM40013 florfenicol exporter [Aspergillus bombycis] [25.4](file:///Users/JMBNew/Desktop/Articles%20en%20Cours/Article%20Diane/Bb_42-1_output-blast_named.html#BL_ORD_ID:511) 0.49

> AEA08384 chloramphenicol transporter [Escherichia coli]

Length=419

Score = 26.2 bits (56), Expect = 0.27, Method: Compositional matrix adjust.

Identities = 11/21 (52%), Positives = 14/21 (67%), Gaps = 0/21 (0%)

Frame = +3

Query 321 PRRVAWPVSYSPLTMATILHG 383

PR AWPV LT+AT++ G

Sbjct 367 PRNTAWPVVVYCLTLATVVLG 387

> XP_022383730 florfenicol exporter [Aspergillus bombycis]

Length=516

Score = 25.4 bits (54), Expect = 0.49, Method: Compositional matrix adjust.

Identities = 19/73 (26%), Positives = 29/73 (40%), Gaps = 14/73 (19%)

Frame = +3

Query 192 HVATRNPTRRHGLRFPERQRL*RRPGARLGCDQAILGVADGREPRRVAWPVSYSPLT--- 362

H+A R G+ P +G AI G G++ ++ W ++PLT

Sbjct 233 HIAGNGTVRLRGIHKPFLYVF-------IGQKGAITGADPGQKKPKLTWRAIFAPLTFLV 285

Query 363 ----MATILHGSI 389

T+L GSI

Sbjct 286 EKDIFVTLLFGSI 298

> OGM40013 florfenicol exporter [Aspergillus bombycis]

Length=516

Score = 25.4 bits (54), Expect = 0.49, Method: Compositional matrix adjust.

Identities = 19/73 (26%), Positives = 29/73 (40%), Gaps = 14/73 (19%)

Frame = +3

Query 192 HVATRNPTRRHGLRFPERQRL*RRPGARLGCDQAILGVADGREPRRVAWPVSYSPLT--- 362

H+A R G+ P +G AI G G++ ++ W ++PLT

Sbjct 233 HIAGNGTVRLRGIHKPFLYVF-------IGQKGAITGADPGQKKPKLTWRAIFAPLTFLV 285

Query 363 ----MATILHGSI 389

T+L GSI

Sbjct 286 EKDIFVTLLFGSI 298

Lambda K H a alpha

0.318 0.134 0.401 0.792 4.96

Gapped

Lambda K H a alpha sigma

0.267 0.0410 0.140 1.90 42.6 43.6

Effective search space used: 18972976

**Query=** NODE_258_length_463_cov_0.720238

Length=463

Score E

Sequences producing significant alignments: (Bits) Value

AAZ39943 membrane transport protein, partial [Rhizobium legumin... [25.4](file:///Users/JMBNew/Desktop/Articles%20en%20Cours/Article%20Diane/Bb_42-1_output-blast_named.html#BL_ORD_ID:22) 0.36

ACS55612 drug resistance transporter, Bcr/CflA subfamily [Rhizo... [25.0](file:///Users/JMBNew/Desktop/Articles%20en%20Cours/Article%20Diane/Bb_42-1_output-blast_named.html#BL_ORD_ID:56) 0.62

> AAZ39943 membrane transport protein, partial [Rhizobium leguminosarum

bv. trifolii TA1]

Length=194

Score = 25.4 bits (54), Expect = 0.36, Method: Compositional matrix adjust.

Identities = 14/40 (35%), Positives = 22/40 (55%), Gaps = 1/40 (3%)

Frame = +3

Query 96 GPIESVCRHLRRRYDAVGGLIDVFSIMRDMAAGRPQRPSL 215

PI V R L + A L+ F+ +RD+ AGRP+ ++

Sbjct 103 APIFVVLR-LLQAVGASATLVATFATVRDVYAGRPESSTI 141

> ACS55612 drug resistance transporter, Bcr/CflA subfamily [Rhizobium

leguminosarum bv. trifolii WSM1325]

Length=395

Score = 25.0 bits (53), Expect = 0.62, Method: Compositional matrix adjust.

Identities = 13/39 (33%), Positives = 22/39 (56%), Gaps = 1/39 (3%)

Frame = +3

Query 99 PIESVCRHLRRRYDAVGGLIDVFSIMRDMAAGRPQRPSL 215

P+ V R L + A L+ F+ +RD+ AGRP+ ++

Sbjct 104 PVFVVLR-LLQAVGASATLVATFATVRDVYAGRPESSTI 141

Lambda K H a alpha

0.318 0.134 0.401 0.792 4.96

Gapped

Lambda K H a alpha sigma

0.267 0.0410 0.140 1.90 42.6 43.6

Effective search space used: 18972976

**Query=** NODE_259_length_463_cov_0.720238

Length=463

***** No hits found *****

Lambda K H a alpha

0.318 0.134 0.401 0.792 4.96

Gapped

Lambda K H a alpha sigma

0.267 0.0410 0.140 1.90 42.6 43.6

Effective search space used: 18972976

**Query=** NODE_260_length_461_cov_0.538922

Length=461

***** No hits found *****

Lambda K H a alpha

0.318 0.134 0.401 0.792 4.96

Gapped

Lambda K H a alpha sigma

0.267 0.0410 0.140 1.90 42.6 43.6

Effective search space used: 18757374

**Query=** NODE_261_length_461_cov_0.688623

Length=461

***** No hits found *****

Lambda K H a alpha

0.318 0.134 0.401 0.792 4.96

Gapped

Lambda K H a alpha sigma

0.267 0.0410 0.140 1.90 42.6 43.6

Effective search space used: 18757374

**Query=** NODE_262_length_461_cov_0.733533

Length=461

***** No hits found *****

Lambda K H a alpha

0.318 0.134 0.401 0.792 4.96

Gapped

Lambda K H a alpha sigma

0.267 0.0410 0.140 1.90 42.6 43.6

Effective search space used: 18757374

**Query=** NODE_263_length_461_cov_0.781437

Length=461

***** No hits found *****

Lambda K H a alpha

0.318 0.134 0.401 0.792 4.96

Gapped

Lambda K H a alpha sigma

0.267 0.0410 0.140 1.90 42.6 43.6

Effective search space used: 18757374

**Query=** NODE_264_length_460_cov_0.600601

Length=460

***** No hits found *****

Lambda K H a alpha

0.318 0.134 0.401 0.792 4.96

Gapped

Lambda K H a alpha sigma

0.267 0.0410 0.140 1.90 42.6 43.6

Effective search space used: 18757374

**Query=** NODE_265_length_459_cov_0.496988

Length=459

***** No hits found *****

Lambda K H a alpha

0.318 0.134 0.401 0.792 4.96

Gapped

Lambda K H a alpha sigma

0.267 0.0410 0.140 1.90 42.6 43.6

Effective search space used: 18757374

**Query=** NODE_266_length_459_cov_0.707831

Length=459

***** No hits found *****

Lambda K H a alpha

0.318 0.134 0.401 0.792 4.96

Gapped

Lambda K H a alpha sigma

0.267 0.0410 0.140 1.90 42.6 43.6

Effective search space used: 18757374

**Query=** NODE_267_length_459_cov_0.518072

Length=459

***** No hits found *****

Lambda K H a alpha

0.318 0.134 0.401 0.792 4.96

Gapped

Lambda K H a alpha sigma

0.267 0.0410 0.140 1.90 42.6 43.6

Effective search space used: 18757374

**Query=** NODE_268_length_458_cov_0.353474

Length=458

Score E

Sequences producing significant alignments: (Bits) Value

EED10097 radical SAM enzyme, Cfr family [Thermus aquaticus Y51M... [26.9](file:///Users/JMBNew/Desktop/Articles%20en%20Cours/Article%20Diane/Bb_42-1_output-blast_named.html#BL_ORD_ID:419) 0.13

AAS81916 florfenicol resistance protein [Thermus thermophilus H... [26.6](file:///Users/JMBNew/Desktop/Articles%20en%20Cours/Article%20Diane/Bb_42-1_output-blast_named.html#BL_ORD_ID:16) 0.16

> EED10097 radical SAM enzyme, Cfr family [Thermus aquaticus Y51MC23]

Length=349

Score = 26.9 bits (58), Expect = 0.13, Method: Compositional matrix adjust.

Identities = 13/47 (28%), Positives = 23/47 (49%), Gaps = 12/47 (26%)

Frame = -1

Query 341 LGLRCGMRDHTPDSKARRAAATAWSISACEQAVTSARNWPVAGLMAA 201

+G+R + H PD + RR + + +A +P+A +MAA

Sbjct 201 VGVRLALSLHAPDDETRR------------KIIPTAHRYPIAEIMAA 235

> AAS81916 florfenicol resistance protein [Thermus thermophilus

HB27]

Length=355

Score = 26.6 bits (57), Expect = 0.16, Method: Compositional matrix adjust.

Identities = 13/47 (28%), Positives = 22/47 (47%), Gaps = 12/47 (26%)

Frame = -1

Query 341 LGLRCGMRDHTPDSKARRAAATAWSISACEQAVTSARNWPVAGLMAA 201

LG+R + H PD + RR + + +A +P+A +M A

Sbjct 207 LGVRLALSLHAPDDETRR------------KIIPTAHRYPIAEIMEA 241

Lambda K H a alpha

0.318 0.134 0.401 0.792 4.96

Gapped

Lambda K H a alpha sigma

0.267 0.0410 0.140 1.90 42.6 43.6

Effective search space used: 18541772

**Query=** NODE_269_length_458_cov_0.253776

Length=458

***** No hits found *****

Lambda K H a alpha

0.318 0.134 0.401 0.792 4.96

Gapped

Lambda K H a alpha sigma

0.267 0.0410 0.140 1.90 42.6 43.6

Effective search space used: 18541772

**Query=** NODE_270_length_457_cov_0.40303

Length=457

***** No hits found *****

Lambda K H a alpha

0.318 0.134 0.401 0.792 4.96

Gapped

Lambda K H a alpha sigma

0.267 0.0410 0.140 1.90 42.6 43.6

Effective search space used: 18541772

**Query=** NODE_271_length_457_cov_0.363636

Length=457

***** No hits found *****

Lambda K H a alpha

0.318 0.134 0.401 0.792 4.96

Gapped

Lambda K H a alpha sigma

0.267 0.0410 0.140 1.90 42.6 43.6

Effective search space used: 18541772

**Query=** NODE_272_length_455_cov_0.710366

Length=455

Score E

Sequences producing significant alignments: (Bits) Value

YP_002286764 dihydropteroate synthase (plasmid) [Pasteurella mu... [26.2](file:///Users/JMBNew/Desktop/Articles%20en%20Cours/Article%20Diane/Bb_42-1_output-blast_named.html#BL_ORD_ID:607) 0.24

CAQ77167 dihydropteroate synthase (plasmid) [Pasteurella multoc... [26.2](file:///Users/JMBNew/Desktop/Articles%20en%20Cours/Article%20Diane/Bb_42-1_output-blast_named.html#BL_ORD_ID:347) 0.24

YP_512237 type 2 dihydropteroate synthase (plasmid) [Biberstein... [25.4](file:///Users/JMBNew/Desktop/Articles%20en%20Cours/Article%20Diane/Bb_42-1_output-blast_named.html#BL_ORD_ID:599) 0.40

CAJ65908 type 2 dihydropteroate synthase (plasmid) [Bibersteini... [25.4](file:///Users/JMBNew/Desktop/Articles%20en%20Cours/Article%20Diane/Bb_42-1_output-blast_named.html#BL_ORD_ID:336) 0.40

> YP_002286764 dihydropteroate synthase (plasmid) [Pasteurella

multocida]

Length=271

Score = 26.2 bits (56), Expect = 0.24, Method: Compositional matrix adjust.

Identities = 24/78 (31%), Positives = 36/78 (46%), Gaps = 6/78 (8%)

Frame = -2

Query 259 IRSGAVPVSPFRDQPSTADYLMCRLVS---DI*KYQEVNFYTILQKPSILQLSKIDSVQI 89

+++ +PVS QP+T Y + R V+ DI + + FY L K S +L + SVQ

Sbjct 75 LKADGIPVSLDSYQPATQAYALSRGVAYLNDIRGFPDAAFYPQLAKSSA-KLVVMHSVQ- 132

Query 88 INNLKFYYYISMSDIEDH 35

+ DI DH

Sbjct 133 -DGQADRREAPAGDIMDH 149

> CAQ77167 dihydropteroate synthase (plasmid) [Pasteurella multocida]

Length=271

Score = 26.2 bits (56), Expect = 0.24, Method: Compositional matrix adjust.

Identities = 24/78 (31%), Positives = 36/78 (46%), Gaps = 6/78 (8%)

Frame = -2

Query 259 IRSGAVPVSPFRDQPSTADYLMCRLVS---DI*KYQEVNFYTILQKPSILQLSKIDSVQI 89

+++ +PVS QP+T Y + R V+ DI + + FY L K S +L + SVQ

Sbjct 75 LKADGIPVSLDSYQPATQAYALSRGVAYLNDIRGFPDAAFYPQLAKSSA-KLVVMHSVQ- 132

Query 88 INNLKFYYYISMSDIEDH 35

+ DI DH

Sbjct 133 -DGQADRREAPAGDIMDH 149

> YP_512237 type 2 dihydropteroate synthase (plasmid) [Bibersteinia

trehalosi]

Length=272

Score = 25.4 bits (54), Expect = 0.40, Method: Compositional matrix adjust.

Identities = 20/59 (34%), Positives = 31/59 (53%), Gaps = 4/59 (7%)

Frame = -2

Query 259 IRSGAVPVSPFRDQPSTADYLMCRLVS---DI*KYQEVNFYTILQKPSILQLSKIDSVQ 92

+++ +PVS QP+T Y + R V+ DI + + FY L K S +L + SVQ

Sbjct 75 LKADGIPVSLDSYQPATQAYALSRGVAYLNDIRGFPDAAFYPQLAKSSA-KLVVMHSVQ 132

> CAJ65908 type 2 dihydropteroate synthase (plasmid) [Bibersteinia

trehalosi]

Length=272

Score = 25.4 bits (54), Expect = 0.40, Method: Compositional matrix adjust.

Identities = 20/59 (34%), Positives = 31/59 (53%), Gaps = 4/59 (7%)

Frame = -2

Query 259 IRSGAVPVSPFRDQPSTADYLMCRLVS---DI*KYQEVNFYTILQKPSILQLSKIDSVQ 92

+++ +PVS QP+T Y + R V+ DI + + FY L K S +L + SVQ

Sbjct 75 LKADGIPVSLDSYQPATQAYALSRGVAYLNDIRGFPDAAFYPQLAKSSA-KLVVMHSVQ 132

Lambda K H a alpha

0.318 0.134 0.401 0.792 4.96

Gapped

Lambda K H a alpha sigma

0.267 0.0410 0.140 1.90 42.6 43.6

Effective search space used: 18326170

**Query=** NODE_273_length_455_cov_0.713415

Length=455

***** No hits found *****

Lambda K H a alpha

0.318 0.134 0.401 0.792 4.96

Gapped

Lambda K H a alpha sigma

0.267 0.0410 0.140 1.90 42.6 43.6

Effective search space used: 18326170

**Query=** NODE_274_length_455_cov_0.679878

Length=455

Score E

Sequences producing significant alignments: (Bits) Value

JK_RS07220_-_3432187_translation chloramphenicol resistance pro... [26.2](file:///Users/JMBNew/Desktop/Articles%20en%20Cours/Article%20Diane/Bb_42-1_output-blast_named.html#BL_ORD_ID:677) 0.24

> JK_RS07220_-_3432187_translation chloramphenicol resistance protein

Length=391

Score = 26.2 bits (56), Expect = 0.24, Method: Compositional matrix adjust.

Identities = 8/16 (50%), Positives = 10/16 (63%), Gaps = 0/16 (0%)

Frame = +2

Query 62 TSNGWRRSFWRKPRLC 109

T+ GWR +FW LC

Sbjct 152 TALGWRTTFWAIAILC 167

Lambda K H a alpha

0.315 0.128 0.396 0.792 4.96

Gapped

Lambda K H a alpha sigma

0.267 0.0410 0.140 1.90 42.6 43.6

Effective search space used: 18326170

**Query=** NODE_275_length_455_cov_0.307927

Length=455

***** No hits found *****

Lambda K H a alpha

0.318 0.134 0.401 0.792 4.96

Gapped

Lambda K H a alpha sigma

0.267 0.0410 0.140 1.90 42.6 43.6

Effective search space used: 18326170

**Query=** NODE_276_length_454_cov_0.675841

Length=454

***** No hits found *****

Lambda K H a alpha

0.318 0.134 0.401 0.792 4.96

Gapped

Lambda K H a alpha sigma

0.267 0.0410 0.140 1.90 42.6 43.6

Effective search space used: 18326170

**Query=** NODE_277_length_454_cov_0.678899

Length=454

***** No hits found *****

Lambda K H a alpha

0.318 0.134 0.401 0.792 4.96

Gapped

Lambda K H a alpha sigma

0.267 0.0410 0.140 1.90 42.6 43.6

Effective search space used: 18326170

**Query=** NODE_278_length_453_cov_0.754601

Length=453

***** No hits found *****

Lambda K H a alpha

0.318 0.134 0.401 0.792 4.96

Gapped

Lambda K H a alpha sigma

0.267 0.0410 0.140 1.90 42.6 43.6

Effective search space used: 18326170

**Query=** NODE_279_length_453_cov_0.726994

Length=453

***** No hits found *****

Lambda K H a alpha

0.318 0.134 0.401 0.792 4.96

Gapped

Lambda K H a alpha sigma

0.267 0.0410 0.140 1.90 42.6 43.6

Effective search space used: 18326170

**Query=** NODE_280_length_453_cov_0.506135

Length=453

***** No hits found *****

Lambda K H a alpha

0.318 0.134 0.401 0.792 4.96

Gapped

Lambda K H a alpha sigma

0.267 0.0410 0.140 1.90 42.6 43.6

Effective search space used: 18326170

**Query=** NODE_281_length_452_cov_0.504615

Length=452

***** No hits found *****

Lambda K H a alpha

0.318 0.134 0.401 0.792 4.96

Gapped

Lambda K H a alpha sigma

0.267 0.0410 0.140 1.90 42.6 43.6

Effective search space used: 18110568

**Query=** NODE_282_length_451_cov_0.756173

Length=451

***** No hits found *****

Lambda K H a alpha

0.318 0.134 0.401 0.792 4.96

Gapped

Lambda K H a alpha sigma

0.267 0.0410 0.140 1.90 42.6 43.6

Effective search space used: 18110568

**Query=** NODE_283_length_451_cov_0.391975

Length=451

***** No hits found *****

Lambda K H a alpha

0.318 0.134 0.401 0.792 4.96

Gapped

Lambda K H a alpha sigma

0.267 0.0410 0.140 1.90 42.6 43.6

Effective search space used: 18110568

**Query=** NODE_284_length_450_cov_0.331269

Length=450

***** No hits found *****

Lambda K H a alpha

0.318 0.134 0.401 0.792 4.96

Gapped

Lambda K H a alpha sigma

0.267 0.0410 0.140 1.90 42.6 43.6

Effective search space used: 18110568

**Query=** NODE_285_length_450_cov_0.767802

Length=450

Score E

Sequences producing significant alignments: (Bits) Value

ORY09537 putative florfenicol exporter [Clohesyomyces aquaticus] [24.6](file:///Users/JMBNew/Desktop/Articles%20en%20Cours/Article%20Diane/Bb_42-1_output-blast_named.html#BL_ORD_ID:514) 0.85

> ORY09537 putative florfenicol exporter [Clohesyomyces aquaticus]

Length=552

Score = 24.6 bits (52), Expect = 0.85, Method: Composition-based stats.

Identities = 14/32 (44%), Positives = 17/32 (53%), Gaps = 0/32 (0%)

Frame = +2

Query 233 VAYIGACTGAKLDDLRAAAQVCRCLLYTSYAA 328

V Y+GA G L + AA V RCL T +A

Sbjct 151 VIYMGANIGLALQNSYAALFVLRCLQSTGSSA 182

Lambda K H a alpha

0.318 0.134 0.401 0.792 4.96

Gapped

Lambda K H a alpha sigma

0.267 0.0410 0.140 1.90 42.6 43.6

Effective search space used: 18110568

**Query=** NODE_286_length_449_cov_0.416149

Length=449

***** No hits found *****

Lambda K H a alpha

0.318 0.134 0.401 0.792 4.96

Gapped

Lambda K H a alpha sigma

0.267 0.0410 0.140 1.90 42.6 43.6

Effective search space used: 17894966

**Query=** NODE_287_length_448_cov_0.794393

Length=448

***** No hits found *****

Lambda K H a alpha

0.318 0.134 0.401 0.792 4.96

Gapped

Lambda K H a alpha sigma

0.267 0.0410 0.140 1.90 42.6 43.6

Effective search space used: 17894966

**Query=** NODE_288_length_448_cov_0.713396

Length=448

***** No hits found *****

Lambda K H a alpha

0.318 0.134 0.401 0.792 4.96

Gapped

Lambda K H a alpha sigma

0.267 0.0410 0.140 1.90 42.6 43.6

Effective search space used: 17894966

**Query=** NODE_289_length_448_cov_0.672897

Length=448

***** No hits found *****

Lambda K H a alpha

0.318 0.134 0.401 0.792 4.96

Gapped

Lambda K H a alpha sigma

0.267 0.0410 0.140 1.90 42.6 43.6

Effective search space used: 17894966

**Query=** NODE_290_length_448_cov_0.838006

Length=448

***** No hits found *****

Lambda K H a alpha

0.318 0.134 0.401 0.792 4.96

Gapped

Lambda K H a alpha sigma

0.267 0.0410 0.140 1.90 42.6 43.6

Effective search space used: 17894966

**Query=** NODE_291_length_448_cov_0.669782

Length=448

***** No hits found *****

Lambda K H a alpha

0.318 0.134 0.401 0.792 4.96

Gapped

Lambda K H a alpha sigma

0.267 0.0410 0.140 1.90 42.6 43.6

Effective search space used: 17894966

**Query=** NODE_292_length_447_cov_0.784375

Length=447

Score E

Sequences producing significant alignments: (Bits) Value

EJU08814 florfenicol resistance protein [Fusobacterium hwasooki... [26.9](file:///Users/JMBNew/Desktop/Articles%20en%20Cours/Article%20Diane/Bb_42-1_output-blast_named.html#BL_ORD_ID:444) 0.11

NP_603423 florfenicol resistance protein [Fusobacterium nucleat... [26.9](file:///Users/JMBNew/Desktop/Articles%20en%20Cours/Article%20Diane/Bb_42-1_output-blast_named.html#BL_ORD_ID:504) 0.12

AAL94722 Florfenicol resistance protein [Fusobacterium nucleatu... [26.9](file:///Users/JMBNew/Desktop/Articles%20en%20Cours/Article%20Diane/Bb_42-1_output-blast_named.html#BL_ORD_ID:6) 0.12

EJG09268 florfenicol resistance protein [Fusobacterium nucleatu... [25.8](file:///Users/JMBNew/Desktop/Articles%20en%20Cours/Article%20Diane/Bb_42-1_output-blast_named.html#BL_ORD_ID:443) 0.34

EMP16199 florfenicol resistance protein [Fusobacterium nucleatu... [25.4](file:///Users/JMBNew/Desktop/Articles%20en%20Cours/Article%20Diane/Bb_42-1_output-blast_named.html#BL_ORD_ID:456) 0.35

> EJU08814 florfenicol resistance protein [Fusobacterium hwasookii

ChDC F128]

Length=358

Score = 26.9 bits (58), Expect = 0.11, Method: Compositional matrix adjust.

Identities = 13/41 (32%), Positives = 19/41 (46%), Gaps = 0/41 (0%)

Frame = -1

Query 366 SGGGWVSTCCLRRPISCSRGTRSMMRRSWPPGCASAASWCA 244

GG + T LR S ++ R+ + S GC S+CA

Sbjct 89 EDGGTIETVLLRHKDSKNKEIRNTLCVSSQVGCPVKCSFCA 129

> NP_603423 florfenicol resistance protein [Fusobacterium nucleatum

subsp. nucleatum ATCC 25586]

Length=358

Score = 26.9 bits (58), Expect = 0.12, Method: Compositional matrix adjust.

Identities = 13/41 (32%), Positives = 19/41 (46%), Gaps = 0/41 (0%)

Frame = -1

Query 366 SGGGWVSTCCLRRPISCSRGTRSMMRRSWPPGCASAASWCA 244

GG + T LR S ++ R+ + S GC S+CA

Sbjct 89 EDGGTIETVLLRHKDSKNKEIRNTLCVSSQVGCPVKCSFCA 129

> AAL94722 Florfenicol resistance protein [Fusobacterium nucleatum

subsp. nucleatum ATCC 25586]

Length=358

Score = 26.9 bits (58), Expect = 0.12, Method: Compositional matrix adjust.

Identities = 13/41 (32%), Positives = 19/41 (46%), Gaps = 0/41 (0%)

Frame = -1

Query 366 SGGGWVSTCCLRRPISCSRGTRSMMRRSWPPGCASAASWCA 244

GG + T LR S ++ R+ + S GC S+CA

Sbjct 89 EDGGTIETVLLRHKDSKNKEIRNTLCVSSQVGCPVKCSFCA 129

> EJG09268 florfenicol resistance protein [Fusobacterium nucleatum

subsp. fusiforme ATCC 51190]

Length=358

Score = 25.8 bits (55), Expect = 0.34, Method: Compositional matrix adjust.

Identities = 13/38 (34%), Positives = 18/38 (47%), Gaps = 0/38 (0%)

Frame = -1

Query 357 GWVSTCCLRRPISCSRGTRSMMRRSWPPGCASAASWCA 244

G + T LR S +R R+ + S GC S+CA

Sbjct 92 GTIETVLLRHKDSKNREIRNTLCVSSQVGCPVKCSFCA 129

> EMP16199 florfenicol resistance protein [Fusobacterium nucleatum

CC53]

Length=358

Score = 25.4 bits (54), Expect = 0.35, Method: Compositional matrix adjust.

Identities = 13/38 (34%), Positives = 18/38 (47%), Gaps = 0/38 (0%)

Frame = -1

Query 357 GWVSTCCLRRPISCSRGTRSMMRRSWPPGCASAASWCA 244

G + T LR S +R R+ + S GC S+CA

Sbjct 92 GTIETVLLRHKDSKNREIRNTLCVSSQVGCPVKCSFCA 129

Lambda K H a alpha

0.318 0.134 0.401 0.792 4.96

Gapped

Lambda K H a alpha sigma

0.267 0.0410 0.140 1.90 42.6 43.6

Effective search space used: 17894966

**Query=** NODE_293_length_447_cov_0.35625

Length=447

***** No hits found *****

Lambda K H a alpha

0.318 0.134 0.401 0.792 4.96

Gapped

Lambda K H a alpha sigma

0.267 0.0410 0.140 1.90 42.6 43.6

Effective search space used: 17894966

**Query=** NODE_294_length_445_cov_0.808176

Length=445

***** No hits found *****

Lambda K H a alpha

0.318 0.134 0.401 0.792 4.96

Gapped

Lambda K H a alpha sigma

0.267 0.0410 0.140 1.90 42.6 43.6

Effective search space used: 17679364

**Query=** NODE_295_length_445_cov_0.666667

Length=445

Score E

Sequences producing significant alignments: (Bits) Value

CBJ12806 putative florfenicol efflux pump-like protein [Legione... [30.0](file:///Users/JMBNew/Desktop/Articles%20en%20Cours/Article%20Diane/Bb_42-1_output-blast_named.html#BL_ORD_ID:350) 0.011

> CBJ12806 putative florfenicol efflux pump-like protein [Legionella

longbeachae NSW150]

Length=386

Score = 30.0 bits (66), Expect = 0.011, Method: Compositional matrix adjust.

Identities = 21/58 (36%), Positives = 28/58 (48%), Gaps = 6/58 (10%)

Frame = -1

Query 334 LVGVQWLLWPPHPLMASIPALVTP*SRAPVSPG*RDAGPKRPHPL--VPEAPAGEGGE 167

LVG+ W PHPL+ +IP L S + ++ G + P P P A GEG E

Sbjct 330 LVGILITQWTPHPLILAIPVL----SLSTLALIKMKKGERAPDPCDQTPNANLGEGIE 383

Lambda K H a alpha

0.318 0.134 0.401 0.792 4.96

Gapped

Lambda K H a alpha sigma

0.267 0.0410 0.140 1.90 42.6 43.6

Effective search space used: 17679364

**Query=** NODE_296_length_445_cov_0.220126

Length=445

***** No hits found *****

Lambda K H a alpha

0.318 0.134 0.401 0.792 4.96

Gapped

Lambda K H a alpha sigma

0.267 0.0410 0.140 1.90 42.6 43.6

Effective search space used: 17679364

**Query=** NODE_297_length_445_cov_0.462264

Length=445

***** No hits found *****

Lambda K H a alpha

0.318 0.134 0.401 0.792 4.96

Gapped

Lambda K H a alpha sigma

0.267 0.0410 0.140 1.90 42.6 43.6

Effective search space used: 17679364

**Query=** NODE_298_length_444_cov_0.81388

Length=444

***** No hits found *****

Lambda K H a alpha

0.318 0.134 0.401 0.792 4.96

Gapped

Lambda K H a alpha sigma

0.267 0.0410 0.140 1.90 42.6 43.6

Effective search space used: 17679364

**Query=** NODE_299_length_444_cov_0.772871

Length=444

***** No hits found *****

Lambda K H a alpha

0.318 0.134 0.401 0.792 4.96

Gapped

Lambda K H a alpha sigma

0.267 0.0410 0.140 1.90 42.6 43.6

Effective search space used: 17679364

**Query=** NODE_300_length_444_cov_0.731861

Length=444

***** No hits found *****

Lambda K H a alpha

0.318 0.134 0.401 0.792 4.96

Gapped

Lambda K H a alpha sigma

0.267 0.0410 0.140 1.90 42.6 43.6

Effective search space used: 17679364

**Query=** NODE_301_length_444_cov_0.365931

Length=444

***** No hits found *****

Lambda K H a alpha

0.318 0.134 0.401 0.792 4.96

Gapped

Lambda K H a alpha sigma

0.267 0.0410 0.140 1.90 42.6 43.6

Effective search space used: 17679364

**Query=** NODE_302_length_443_cov_0.702532

Length=443

***** No hits found *****

Lambda K H a alpha

0.318 0.134 0.401 0.792 4.96

Gapped

Lambda K H a alpha sigma

0.267 0.0410 0.140 1.90 42.6 43.6

Effective search space used: 17736928

**Query=** NODE_303_length_442_cov_0.857143

Length=442

***** No hits found *****

Lambda K H a alpha

0.318 0.134 0.401 0.792 4.96

Gapped

Lambda K H a alpha sigma

0.267 0.0410 0.140 1.90 42.6 43.6

Effective search space used: 17736928

**Query=** NODE_304_length_441_cov_0.780255

Length=441

***** No hits found *****

Lambda K H a alpha

0.318 0.134 0.401 0.792 4.96

Gapped

Lambda K H a alpha sigma

0.267 0.0410 0.140 1.90 42.6 43.6

Effective search space used: 17736928

**Query=** NODE_305_length_440_cov_0.801917

Length=440

***** No hits found *****

Lambda K H a alpha

0.318 0.134 0.401 0.792 4.96

Gapped

Lambda K H a alpha sigma

0.267 0.0410 0.140 1.90 42.6 43.6

Effective search space used: 17520624

**Query=** NODE_306_length_440_cov_0.638978

Length=440

***** No hits found *****

Lambda K H a alpha

0.318 0.134 0.401 0.792 4.96

Gapped

Lambda K H a alpha sigma

0.267 0.0410 0.140 1.90 42.6 43.6

Effective search space used: 17520624

**Query=** NODE_307_length_440_cov_0.693291

Length=440

***** No hits found *****

Lambda K H a alpha

0.318 0.134 0.401 0.792 4.96

Gapped

Lambda K H a alpha sigma

0.267 0.0410 0.140 1.90 42.6 43.6

Effective search space used: 17520624

**Query=** NODE_308_length_440_cov_0.635783

Length=440

***** No hits found *****

Lambda K H a alpha

0.318 0.134 0.401 0.792 4.96

Gapped

Lambda K H a alpha sigma

0.267 0.0410 0.140 1.90 42.6 43.6

Effective search space used: 17520624

**Query=** NODE_309_length_440_cov_0.664537

Length=440

***** No hits found *****

Lambda K H a alpha

0.318 0.134 0.401 0.792 4.96

Gapped

Lambda K H a alpha sigma

0.267 0.0410 0.140 1.90 42.6 43.6

Effective search space used: 17520624

**Query=** NODE_310_length_438_cov_0.559486

Length=438

***** No hits found *****

Lambda K H a alpha

0.318 0.134 0.401 0.792 4.96

Gapped

Lambda K H a alpha sigma

0.267 0.0410 0.140 1.90 42.6 43.6

Effective search space used: 17520624

**Query=** NODE_311_length_438_cov_0.794212

Length=438

***** No hits found *****

Lambda K H a alpha

0.318 0.134 0.401 0.792 4.96

Gapped

Lambda K H a alpha sigma

0.267 0.0410 0.140 1.90 42.6 43.6

Effective search space used: 17520624

**Query=** NODE_312_length_438_cov_0.652733

Length=438

***** No hits found *****

Lambda K H a alpha

0.318 0.134 0.401 0.792 4.96

Gapped

Lambda K H a alpha sigma

0.267 0.0410 0.140 1.90 42.6 43.6

Effective search space used: 17520624

**Query=** NODE_313_length_438_cov_7.33119

Length=438

***** No hits found *****

Lambda K H a alpha

0.318 0.134 0.401 0.792 4.96

Gapped

Lambda K H a alpha sigma

0.267 0.0410 0.140 1.90 42.6 43.6

Effective search space used: 17520624

**Query=** NODE_314_length_437_cov_0.658065

Length=437

***** No hits found *****

Lambda K H a alpha

0.318 0.134 0.401 0.792 4.96

Gapped

Lambda K H a alpha sigma

0.267 0.0410 0.140 1.90 42.6 43.6

Effective search space used: 17304320

**Query=** NODE_315_length_436_cov_0.540453

Length=436

***** No hits found *****

Lambda K H a alpha

0.318 0.134 0.401 0.792 4.96

Gapped

Lambda K H a alpha sigma

0.267 0.0410 0.140 1.90 42.6 43.6

Effective search space used: 17304320

**Query=** NODE_316_length_436_cov_0.660194

Length=436

***** No hits found *****

Lambda K H a alpha

0.318 0.134 0.401 0.792 4.96

Gapped

Lambda K H a alpha sigma

0.267 0.0410 0.140 1.90 42.6 43.6

Effective search space used: 17304320

**Query=** NODE_317_length_436_cov_0.326861

Length=436

***** No hits found *****

Lambda K H a alpha

0.318 0.134 0.401 0.792 4.96

Gapped

Lambda K H a alpha sigma

0.267 0.0410 0.140 1.90 42.6 43.6

Effective search space used: 17304320

**Query=** NODE_318_length_436_cov_0.550162

Length=436

***** No hits found *****

Lambda K H a alpha

0.318 0.134 0.401 0.792 4.96

Gapped

Lambda K H a alpha sigma

0.267 0.0410 0.140 1.90 42.6 43.6

Effective search space used: 17304320

**Query=** NODE_319_length_435_cov_0.493506

Length=435

***** No hits found *****

Lambda K H a alpha

0.318 0.134 0.401 0.792 4.96

Gapped

Lambda K H a alpha sigma

0.267 0.0410 0.140 1.90 42.6 43.6

Effective search space used: 17304320

**Query=** NODE_320_length_435_cov_0.711039

Length=435

***** No hits found *****

Lambda K H a alpha

0.318 0.134 0.401 0.792 4.96

Gapped

Lambda K H a alpha sigma

0.267 0.0410 0.140 1.90 42.6 43.6

Effective search space used: 17304320

**Query=** NODE_321_length_435_cov_0.655844

Length=435

***** No hits found *****

Lambda K H a alpha

0.318 0.134 0.401 0.792 4.96

Gapped

Lambda K H a alpha sigma

0.267 0.0410 0.140 1.90 42.6 43.6

Effective search space used: 17304320

**Query=** NODE_322_length_434_cov_0.778502

Length=434

***** No hits found *****

Lambda K H a alpha

0.318 0.134 0.401 0.792 4.96

Gapped

Lambda K H a alpha sigma

0.267 0.0410 0.140 1.90 42.6 43.6

Effective search space used: 17088016

**Query=** NODE_323_length_433_cov_0.663399

Length=433

***** No hits found *****

Lambda K H a alpha

0.318 0.134 0.401 0.792 4.96

Gapped

Lambda K H a alpha sigma

0.267 0.0410 0.140 1.90 42.6 43.6

Effective search space used: 17088016

**Query=** NODE_324_length_432_cov_0.819672

Length=432

***** No hits found *****

Lambda K H a alpha

0.318 0.134 0.401 0.792 4.96

Gapped

Lambda K H a alpha sigma

0.267 0.0410 0.140 1.90 42.6 43.6

Effective search space used: 17088016

**Query=** NODE_325_length_431_cov_0.651316

Length=431

***** No hits found *****

Lambda K H a alpha

0.318 0.134 0.401 0.792 4.96

Gapped

Lambda K H a alpha sigma

0.267 0.0410 0.140 1.90 42.6 43.6

Effective search space used: 16871712

**Query=** NODE_326_length_431_cov_0.680921

Length=431

***** No hits found *****

Lambda K H a alpha

0.318 0.134 0.401 0.792 4.96

Gapped

Lambda K H a alpha sigma

0.267 0.0410 0.140 1.90 42.6 43.6

Effective search space used: 16871712

**Query=** NODE_327_length_431_cov_0.289474

Length=431

***** No hits found *****

Lambda K H a alpha

0.318 0.134 0.401 0.792 4.96

Gapped

Lambda K H a alpha sigma

0.267 0.0410 0.140 1.90 42.6 43.6

Effective search space used: 16871712

**Query=** NODE_328_length_430_cov_0.653465

Length=430

***** No hits found *****

Lambda K H a alpha

0.318 0.134 0.401 0.792 4.96

Gapped

Lambda K H a alpha sigma

0.267 0.0410 0.140 1.90 42.6 43.6

Effective search space used: 16871712

**Query=** NODE_329_length_429_cov_0.738411

Length=429

Score E

Sequences producing significant alignments: (Bits) Value

XP_002488834 florfenicol exporter, putative [Talaromyces stipit... [25.0](file:///Users/JMBNew/Desktop/Articles%20en%20Cours/Article%20Diane/Bb_42-1_output-blast_named.html#BL_ORD_ID:587) 0.53

XP_002488833 florfenicol exporter, putative [Talaromyces stipit... [25.0](file:///Users/JMBNew/Desktop/Articles%20en%20Cours/Article%20Diane/Bb_42-1_output-blast_named.html#BL_ORD_ID:586) 0.53

EED11424 florfenicol exporter, putative [Talaromyces stipitatus... [25.0](file:///Users/JMBNew/Desktop/Articles%20en%20Cours/Article%20Diane/Bb_42-1_output-blast_named.html#BL_ORD_ID:421) 0.53

EED11423 florfenicol exporter, putative [Talaromyces stipitatus... [25.0](file:///Users/JMBNew/Desktop/Articles%20en%20Cours/Article%20Diane/Bb_42-1_output-blast_named.html#BL_ORD_ID:420) 0.53

> XP_002488834 florfenicol exporter, putative [Talaromyces stipitatus

ATCC 10500]

Length=478

Score = 25.0 bits (53), Expect = 0.53, Method: Composition-based stats.

Identities = 14/26 (54%), Positives = 15/26 (58%), Gaps = 0/26 (0%)

Frame = +3

Query 276 GGRSLAIRLVDIGIPAPVIQAGIGHR 353

GG LA+ V IG PAPVI G R

Sbjct 439 GGSLLALVAVCIGFPAPVIIWIYGRR 464

> XP_002488833 florfenicol exporter, putative [Talaromyces stipitatus

ATCC 10500]

Length=478

Score = 25.0 bits (53), Expect = 0.53, Method: Composition-based stats.

Identities = 14/26 (54%), Positives = 15/26 (58%), Gaps = 0/26 (0%)

Frame = +3

Query 276 GGRSLAIRLVDIGIPAPVIQAGIGHR 353

GG LA+ V IG PAPVI G R

Sbjct 439 GGSLLALVAVCIGFPAPVIIWIYGRR 464

> EED11424 florfenicol exporter, putative [Talaromyces stipitatus

ATCC 10500]

Length=478

Score = 25.0 bits (53), Expect = 0.53, Method: Composition-based stats.

Identities = 14/26 (54%), Positives = 15/26 (58%), Gaps = 0/26 (0%)

Frame = +3

Query 276 GGRSLAIRLVDIGIPAPVIQAGIGHR 353

GG LA+ V IG PAPVI G R

Sbjct 439 GGSLLALVAVCIGFPAPVIIWIYGRR 464

> EED11423 florfenicol exporter, putative [Talaromyces stipitatus

ATCC 10500]

Length=478

Score = 25.0 bits (53), Expect = 0.53, Method: Composition-based stats.

Identities = 14/26 (54%), Positives = 15/26 (58%), Gaps = 0/26 (0%)

Frame = +3

Query 276 GGRSLAIRLVDIGIPAPVIQAGIGHR 353

GG LA+ V IG PAPVI G R

Sbjct 439 GGSLLALVAVCIGFPAPVIIWIYGRR 464

Lambda K H a alpha

0.318 0.134 0.401 0.792 4.96

Gapped

Lambda K H a alpha sigma

0.267 0.0410 0.140 1.90 42.6 43.6

Effective search space used: 16871712

**Query=** NODE_330_length_429_cov_0.483444

Length=429

***** No hits found *****

Lambda K H a alpha

0.318 0.134 0.401 0.792 4.96

Gapped

Lambda K H a alpha sigma

0.267 0.0410 0.140 1.90 42.6 43.6

Effective search space used: 16871712

**Query=** NODE_331_length_128_cov_19547

Length=128

***** No hits found *****

Effective search space used: 0

Database: BdD_Prot-name.fasta

Posted date: Nov 10, 2017 10:32 AM

Number of letters in database: 261,934

Number of sequences in database: 702

Matrix: BLOSUM62

Gap Penalties: Existence: 11, Extension: 1

Neighboring words threshold: 12

Window for multiple hits: 40
