# Supplementary material for: Polyamino-Isoprenyl Derivatives as Antibiotic Adjuvants and Motility Inhibitors for Bordetella bronchiseptica Porcine Pulmonary Infection Treatment
Source: Front Microbiol. 2019 Aug 13;10:1771. doi: 10.3389/fmicb.2019.01771 (PMC6700233; doi:10.3389/fmicb.2019.01771)
Supplement: Supplementary file 4 [file Table_4.DOCX]

**Supplementary Material 3: DNA Sequence Analysis of SR11-14 Strain**

**BLASTX 2.6.0+**

[**Reference**](https://www.ncbi.nlm.nih.gov/entrez/query.fcgi?db=PubMed&cmd=R%0aetrieve&list_uids=9254694&dopt=Citation)**:**

Stephen F. Altschul, Thomas L. Madden, Alejandro A. Schäffer,

Jinghui Zhang, Zheng Zhang, Webb Miller, and David J. Lipman (1997),

"Gapped BLAST and PSI-BLAST: a new generation of protein database

search programs", Nucleic Acids Res. 25:3389-3402.

Database: BdD_Prot-name.fasta

702 sequences; 261,934 total letters

**Query=** NODE_1_length_15587_cov_29.2634

Length=15587

Score E

Sequences producing significant alignments: (Bits) Value

YP_002286764 dihydropteroate synthase (plasmid) [Pasteurella mu... [493](file:///Users/JMBNew/Desktop/Articles%20en%20Cours/Article%20Diane/Bb_SR-11-14_output-blast_named.html#BL_ORD_ID:607) 6e-161

CAQ77167 dihydropteroate synthase (plasmid) [Pasteurella multoc... [493](file:///Users/JMBNew/Desktop/Articles%20en%20Cours/Article%20Diane/Bb_SR-11-14_output-blast_named.html#BL_ORD_ID:347) 6e-161

YP_512237 type 2 dihydropteroate synthase (plasmid) [Biberstein... [471](file:///Users/JMBNew/Desktop/Articles%20en%20Cours/Article%20Diane/Bb_SR-11-14_output-blast_named.html#BL_ORD_ID:599) 6e-153

CAJ65908 type 2 dihydropteroate synthase (plasmid) [Bibersteini... [471](file:///Users/JMBNew/Desktop/Articles%20en%20Cours/Article%20Diane/Bb_SR-11-14_output-blast_named.html#BL_ORD_ID:336) 6e-153

> YP_002286764 dihydropteroate synthase (plasmid) [Pasteurella

multocida]

Length=271

Score = 493 bits (1270), Expect = 6e-161, Method: Compositional matrix adjust.

Identities = 271/271 (100%), Positives = 271/271 (100%), Gaps = 0/271 (0%)

Frame = -2

Query 15232 MNKSLIIFGIVNITSDSFSDGGRYLAPDAAIAQARKLMAEGADVIDLGPASSNPDAAPVS 15053

MNKSLIIFGIVNITSDSFSDGGRYLAPDAAIAQARKLMAEGADVIDLGPASSNPDAAPVS

Sbjct 1 MNKSLIIFGIVNITSDSFSDGGRYLAPDAAIAQARKLMAEGADVIDLGPASSNPDAAPVS 60

Query 15052 SDTEIARIAPVLDALKADGIPVSLDSYQPATQAYALSRGVAYLNDIRGFPDAAFYPQLAK 14873

SDTEIARIAPVLDALKADGIPVSLDSYQPATQAYALSRGVAYLNDIRGFPDAAFYPQLAK

Sbjct 61 SDTEIARIAPVLDALKADGIPVSLDSYQPATQAYALSRGVAYLNDIRGFPDAAFYPQLAK 120

Query 14872 SSAKLVVMHSVQDGQADRREAPAGDIMDHIAAFFDARIAALTGAGIKRNRLVLDPGMGFF 14693

SSAKLVVMHSVQDGQADRREAPAGDIMDHIAAFFDARIAALTGAGIKRNRLVLDPGMGFF

Sbjct 121 SSAKLVVMHSVQDGQADRREAPAGDIMDHIAAFFDARIAALTGAGIKRNRLVLDPGMGFF 180

Query 14692 LGAAPETSLSVLARFDELRLRFDLPVLLSVSRKSFLRALTGRGPGDVgaatlaaelaaaa 14513

LGAAPETSLSVLARFDELRLRFDLPVLLSVSRKSFLRALTGRGPGDVGAATLAAELAAAA

Sbjct 181 LGAAPETSLSVLARFDELRLRFDLPVLLSVSRKSFLRALTGRGPGDVGAATLAAELAAAA 240

Query 14512 ggADFIRTHEPRPLRDGLAVLAALKETARIR 14420

GGADFIRTHEPRPLRDGLAVLAALKETARIR

Sbjct 241 GGADFIRTHEPRPLRDGLAVLAALKETARIR 271

> CAQ77167 dihydropteroate synthase (plasmid) [Pasteurella multocida]

Length=271

Score = 493 bits (1270), Expect = 6e-161, Method: Compositional matrix adjust.

Identities = 271/271 (100%), Positives = 271/271 (100%), Gaps = 0/271 (0%)

Frame = -2

Query 15232 MNKSLIIFGIVNITSDSFSDGGRYLAPDAAIAQARKLMAEGADVIDLGPASSNPDAAPVS 15053

MNKSLIIFGIVNITSDSFSDGGRYLAPDAAIAQARKLMAEGADVIDLGPASSNPDAAPVS

Sbjct 1 MNKSLIIFGIVNITSDSFSDGGRYLAPDAAIAQARKLMAEGADVIDLGPASSNPDAAPVS 60

Query 15052 SDTEIARIAPVLDALKADGIPVSLDSYQPATQAYALSRGVAYLNDIRGFPDAAFYPQLAK 14873

SDTEIARIAPVLDALKADGIPVSLDSYQPATQAYALSRGVAYLNDIRGFPDAAFYPQLAK

Sbjct 61 SDTEIARIAPVLDALKADGIPVSLDSYQPATQAYALSRGVAYLNDIRGFPDAAFYPQLAK 120

Query 14872 SSAKLVVMHSVQDGQADRREAPAGDIMDHIAAFFDARIAALTGAGIKRNRLVLDPGMGFF 14693

SSAKLVVMHSVQDGQADRREAPAGDIMDHIAAFFDARIAALTGAGIKRNRLVLDPGMGFF

Sbjct 121 SSAKLVVMHSVQDGQADRREAPAGDIMDHIAAFFDARIAALTGAGIKRNRLVLDPGMGFF 180

Query 14692 LGAAPETSLSVLARFDELRLRFDLPVLLSVSRKSFLRALTGRGPGDVgaatlaaelaaaa 14513

LGAAPETSLSVLARFDELRLRFDLPVLLSVSRKSFLRALTGRGPGDVGAATLAAELAAAA

Sbjct 181 LGAAPETSLSVLARFDELRLRFDLPVLLSVSRKSFLRALTGRGPGDVGAATLAAELAAAA 240

Query 14512 ggADFIRTHEPRPLRDGLAVLAALKETARIR 14420

GGADFIRTHEPRPLRDGLAVLAALKETARIR

Sbjct 241 GGADFIRTHEPRPLRDGLAVLAALKETARIR 271

> YP_512237 type 2 dihydropteroate synthase (plasmid) [Bibersteinia

trehalosi]

Length=272

Score = 471 bits (1211), Expect = 6e-153, Method: Compositional matrix adjust.

Identities = 263/272 (97%), Positives = 263/272 (97%), Gaps = 1/272 (0%)

Frame = -2

Query 15232 MNKSLIIFGIVNITSDSFSDGGRYLAPDAAIAQARKLMAEGADVIDLGPASSNPDAAPVS 15053

MNKSLIIFGIVNITSDSFSDGGRYLAPDAAIAQARKLMAEGADVIDLGPASSNPDAAPVS

Sbjct 1 MNKSLIIFGIVNITSDSFSDGGRYLAPDAAIAQARKLMAEGADVIDLGPASSNPDAAPVS 60

Query 15052 SDTEIARIAPVLDALKADGIPVSLDSYQPATQAYALSRGVAYLNDIRGFPDAAFYPQLAK 14873

SDTEIARIAPVLDALKADGIPVSLDSYQPATQAYALSRGVAYLNDIRGFPDAAFYPQLAK

Sbjct 61 SDTEIARIAPVLDALKADGIPVSLDSYQPATQAYALSRGVAYLNDIRGFPDAAFYPQLAK 120

Query 14872 SSAKLVVMHSVQDGQA-DRREAPAGDIMDHIAAFFDARIAALTGAGIKRNRLVLDPGMGF 14696

SSAKLVVMHSVQDGQA R P MDHIAAFFDARIAALTGAGIKRNRLVLDPGMGF

Sbjct 121 SSAKLVVMHSVQDGQARSARGHPLATFMDHIAAFFDARIAALTGAGIKRNRLVLDPGMGF 180

Query 14695 FLGAAPETSLSVLARFDELRLRFDLPVLLSVSRKSFLRALTGRGPGDVgaatlaaelaaa 14516

FLGAAPETSLSVLARFDELRLRFDLPVLLSVSRKSFLRALTGRGPGDVGAATLAAELAAA

Sbjct 181 FLGAAPETSLSVLARFDELRLRFDLPVLLSVSRKSFLRALTGRGPGDVGAATLAAELAAA 240

Query 14515 aggADFIRTHEPRPLRDGLAVLAALKETARIR 14420

AGGADFIRTHEPRPLRDGLAVLAALKETARIR

Sbjct 241 AGGADFIRTHEPRPLRDGLAVLAALKETARIR 272

> CAJ65908 type 2 dihydropteroate synthase (plasmid) [Bibersteinia

trehalosi]

Length=272

Score = 471 bits (1211), Expect = 6e-153, Method: Compositional matrix adjust.

Identities = 263/272 (97%), Positives = 263/272 (97%), Gaps = 1/272 (0%)

Frame = -2

Query 15232 MNKSLIIFGIVNITSDSFSDGGRYLAPDAAIAQARKLMAEGADVIDLGPASSNPDAAPVS 15053

MNKSLIIFGIVNITSDSFSDGGRYLAPDAAIAQARKLMAEGADVIDLGPASSNPDAAPVS

Sbjct 1 MNKSLIIFGIVNITSDSFSDGGRYLAPDAAIAQARKLMAEGADVIDLGPASSNPDAAPVS 60

Query 15052 SDTEIARIAPVLDALKADGIPVSLDSYQPATQAYALSRGVAYLNDIRGFPDAAFYPQLAK 14873

SDTEIARIAPVLDALKADGIPVSLDSYQPATQAYALSRGVAYLNDIRGFPDAAFYPQLAK

Sbjct 61 SDTEIARIAPVLDALKADGIPVSLDSYQPATQAYALSRGVAYLNDIRGFPDAAFYPQLAK 120

Query 14872 SSAKLVVMHSVQDGQA-DRREAPAGDIMDHIAAFFDARIAALTGAGIKRNRLVLDPGMGF 14696

SSAKLVVMHSVQDGQA R P MDHIAAFFDARIAALTGAGIKRNRLVLDPGMGF

Sbjct 121 SSAKLVVMHSVQDGQARSARGHPLATFMDHIAAFFDARIAALTGAGIKRNRLVLDPGMGF 180

Query 14695 FLGAAPETSLSVLARFDELRLRFDLPVLLSVSRKSFLRALTGRGPGDVgaatlaaelaaa 14516

FLGAAPETSLSVLARFDELRLRFDLPVLLSVSRKSFLRALTGRGPGDVGAATLAAELAAA

Sbjct 181 FLGAAPETSLSVLARFDELRLRFDLPVLLSVSRKSFLRALTGRGPGDVGAATLAAELAAA 240

Query 14515 aggADFIRTHEPRPLRDGLAVLAALKETARIR 14420

AGGADFIRTHEPRPLRDGLAVLAALKETARIR

Sbjct 241 AGGADFIRTHEPRPLRDGLAVLAALKETARIR 272

Lambda K H a alpha

0.318 0.134 0.401 0.792 4.96

Gapped

Lambda K H a alpha sigma

0.267 0.0410 0.140 1.90 42.6 43.6

Effective search space used: 999520546

**Query=** NODE_2_length_2591_cov_29.4662

Length=2591

***** No hits found *****

Lambda K H a alpha

0.318 0.134 0.401 0.792 4.96

Gapped

Lambda K H a alpha sigma

0.267 0.0410 0.140 1.90 42.6 43.6

Effective search space used: 160366304

**Query=** NODE_3_length_1945_cov_27.3491

Length=1945

***** No hits found *****

Lambda K H a alpha

0.318 0.134 0.401 0.792 4.96

Gapped

Lambda K H a alpha sigma

0.267 0.0410 0.140 1.90 42.6 43.6

Effective search space used: 117484844

**Query=** NODE_4_length_1057_cov_1.32331

Length=1057

***** No hits found *****

Lambda K H a alpha

0.318 0.134 0.401 0.792 4.96

Gapped

Lambda K H a alpha sigma

0.267 0.0410 0.140 1.90 42.6 43.6

Effective search space used: 58376108

**Query=** NODE_5_length_954_cov_0.436518

Length=954

***** No hits found *****

Lambda K H a alpha

0.318 0.134 0.401 0.792 4.96

Gapped

Lambda K H a alpha sigma

0.267 0.0410 0.140 1.90 42.6 43.6

Effective search space used: 51618560

**Query=** NODE_6_length_925_cov_0.79625

Length=925

***** No hits found *****

Lambda K H a alpha

0.318 0.134 0.401 0.792 4.96

Gapped

Lambda K H a alpha sigma

0.267 0.0410 0.140 1.90 42.6 43.6

Effective search space used: 49511680

**Query=** NODE_7_length_916_cov_0.457649

Length=916

***** No hits found *****

Lambda K H a alpha

0.318 0.134 0.401 0.792 4.96

Gapped

Lambda K H a alpha sigma

0.267 0.0410 0.140 1.90 42.6 43.6

Effective search space used: 48879616

**Query=** NODE_8_length_897_cov_0.901299

Length=897

***** No hits found *****

Lambda K H a alpha

0.318 0.134 0.401 0.792 4.96

Gapped

Lambda K H a alpha sigma

0.267 0.0410 0.140 1.90 42.6 43.6

Effective search space used: 47615488

**Query=** NODE_9_length_891_cov_0.629581

Length=891

***** No hits found *****

Lambda K H a alpha

0.318 0.134 0.401 0.792 4.96

Gapped

Lambda K H a alpha sigma

0.267 0.0410 0.140 1.90 42.6 43.6

Effective search space used: 47194112

**Query=** NODE_10_length_832_cov_0.479433

Length=832

***** No hits found *****

Lambda K H a alpha

0.318 0.134 0.401 0.792 4.96

Gapped

Lambda K H a alpha sigma

0.267 0.0410 0.140 1.90 42.6 43.6

Effective search space used: 43334950

**Query=** NODE_11_length_802_cov_1.69185

Length=802

***** No hits found *****

Lambda K H a alpha

0.318 0.134 0.401 0.792 4.96

Gapped

Lambda K H a alpha sigma

0.267 0.0410 0.140 1.90 42.6 43.6

Effective search space used: 41221050

**Query=** NODE_12_length_798_cov_18.8361

Length=798

***** No hits found *****

Lambda K H a alpha

0.318 0.134 0.401 0.792 4.96

Gapped

Lambda K H a alpha sigma

0.267 0.0410 0.140 1.90 42.6 43.6

Effective search space used: 41009660

**Query=** NODE_13_length_783_cov_0.608232

Length=783

***** No hits found *****

Lambda K H a alpha

0.318 0.134 0.401 0.792 4.96

Gapped

Lambda K H a alpha sigma

0.267 0.0410 0.140 1.90 42.6 43.6

Effective search space used: 40297480

**Query=** NODE_14_length_782_cov_0.630534

Length=782

***** No hits found *****

Lambda K H a alpha

0.318 0.134 0.401 0.792 4.96

Gapped

Lambda K H a alpha sigma

0.267 0.0410 0.140 1.90 42.6 43.6

Effective search space used: 40085388

**Query=** NODE_15_length_780_cov_0.385321

Length=780

***** No hits found *****

Lambda K H a alpha

0.318 0.134 0.401 0.792 4.96

Gapped

Lambda K H a alpha sigma

0.267 0.0410 0.140 1.90 42.6 43.6

Effective search space used: 40085388

**Query=** NODE_16_length_770_cov_0.454264

Length=770

***** No hits found *****

Lambda K H a alpha

0.318 0.134 0.401 0.792 4.96

Gapped

Lambda K H a alpha sigma

0.267 0.0410 0.140 1.90 42.6 43.6

Effective search space used: 39237020

**Query=** NODE_17_length_756_cov_0.698262

Length=756

***** No hits found *****

Lambda K H a alpha

0.318 0.134 0.401 0.792 4.96

Gapped

Lambda K H a alpha sigma

0.267 0.0410 0.140 1.90 42.6 43.6

Effective search space used: 38388652

**Query=** NODE_18_length_749_cov_0.4791

Length=749

***** No hits found *****

Lambda K H a alpha

0.318 0.134 0.401 0.792 4.96

Gapped

Lambda K H a alpha sigma

0.267 0.0410 0.140 1.90 42.6 43.6

Effective search space used: 37752376

**Query=** NODE_19_length_744_cov_0.491935

Length=744

***** No hits found *****

Lambda K H a alpha

0.318 0.134 0.401 0.792 4.96

Gapped

Lambda K H a alpha sigma

0.267 0.0410 0.140 1.90 42.6 43.6

Effective search space used: 37540284

**Query=** NODE_20_length_738_cov_0.561375

Length=738

***** No hits found *****

Lambda K H a alpha

0.316 0.135 0.468 0.792 4.96

Gapped

Lambda K H a alpha sigma

0.267 0.0410 0.140 1.90 42.6 43.6

Effective search space used: 37116100

**Query=** NODE_21_length_733_cov_0.592775

Length=733

***** No hits found *****

Lambda K H a alpha

0.318 0.134 0.401 0.792 4.96

Gapped

Lambda K H a alpha sigma

0.267 0.0410 0.140 1.90 42.6 43.6

Effective search space used: 36691916

**Query=** NODE_22_length_717_cov_0.54223

Length=717

***** No hits found *****

Lambda K H a alpha

0.318 0.134 0.401 0.792 4.96

Gapped

Lambda K H a alpha sigma

0.267 0.0410 0.140 1.90 42.6 43.6

Effective search space used: 35631456

**Query=** NODE_23_length_714_cov_0.551959

Length=714

***** No hits found *****

Lambda K H a alpha

0.318 0.134 0.401 0.792 4.96

Gapped

Lambda K H a alpha sigma

0.267 0.0410 0.140 1.90 42.6 43.6

Effective search space used: 35749392

**Query=** NODE_24_length_712_cov_1.58462

Length=712

***** No hits found *****

Lambda K H a alpha

0.318 0.134 0.401 0.792 4.96

Gapped

Lambda K H a alpha sigma

0.267 0.0410 0.140 1.90 42.6 43.6

Effective search space used: 35536598

**Query=** NODE_25_length_711_cov_0.430769

Length=711

***** No hits found *****

Lambda K H a alpha

0.318 0.134 0.401 0.792 4.96

Gapped

Lambda K H a alpha sigma

0.267 0.0410 0.140 1.90 42.6 43.6

Effective search space used: 35536598

**Query=** NODE_26_length_710_cov_0.756849

Length=710

***** No hits found *****

Lambda K H a alpha

0.318 0.134 0.401 0.792 4.96

Gapped

Lambda K H a alpha sigma

0.267 0.0410 0.140 1.90 42.6 43.6

Effective search space used: 35323804

**Query=** NODE_27_length_706_cov_0.284974

Length=706

***** No hits found *****

Lambda K H a alpha

0.318 0.134 0.401 0.792 4.96

Gapped

Lambda K H a alpha sigma

0.267 0.0410 0.140 1.90 42.6 43.6

Effective search space used: 35111010

**Query=** NODE_28_length_702_cov_0.519031

Length=702

***** No hits found *****

Lambda K H a alpha

0.318 0.134 0.401 0.792 4.96

Gapped

Lambda K H a alpha sigma

0.267 0.0410 0.140 1.90 42.6 43.6

Effective search space used: 34898216

**Query=** NODE_29_length_703_cov_7.37088

Length=703

***** No hits found *****

Lambda K H a alpha

0.318 0.134 0.401 0.792 4.96

Gapped

Lambda K H a alpha sigma

0.267 0.0410 0.140 1.90 42.6 43.6

Effective search space used: 34898216

**Query=** NODE_30_length_703_cov_0.584055

Length=703

***** No hits found *****

Lambda K H a alpha

0.316 0.135 0.455 0.792 4.96

Gapped

Lambda K H a alpha sigma

0.267 0.0410 0.140 1.90 42.6 43.6

Effective search space used: 34898216

**Query=** NODE_31_length_690_cov_9.75844

Length=690

***** No hits found *****

Lambda K H a alpha

0.318 0.134 0.401 0.792 4.96

Gapped

Lambda K H a alpha sigma

0.267 0.0410 0.140 1.90 42.6 43.6

Effective search space used: 34047040

**Query=** NODE_32_length_687_cov_0.524064

Length=687

***** No hits found *****

Lambda K H a alpha

0.318 0.134 0.401 0.792 4.96

Gapped

Lambda K H a alpha sigma

0.267 0.0410 0.140 1.90 42.6 43.6

Effective search space used: 33834246

**Query=** NODE_33_length_687_cov_0.583929

Length=687

***** No hits found *****

Lambda K H a alpha

0.318 0.134 0.401 0.792 4.96

Gapped

Lambda K H a alpha sigma

0.267 0.0410 0.140 1.90 42.6 43.6

Effective search space used: 33834246

**Query=** NODE_34_length_687_cov_0.596429

Length=687

***** No hits found *****

Lambda K H a alpha

0.318 0.134 0.401 0.792 4.96

Gapped

Lambda K H a alpha sigma

0.267 0.0410 0.140 1.90 42.6 43.6

Effective search space used: 33834246

**Query=** NODE_35_length_687_cov_0.416071

Length=687

***** No hits found *****

Lambda K H a alpha

0.318 0.134 0.401 0.792 4.96

Gapped

Lambda K H a alpha sigma

0.267 0.0410 0.140 1.90 42.6 43.6

Effective search space used: 33834246

**Query=** NODE_36_length_685_cov_3.94991

Length=685

***** No hits found *****

Lambda K H a alpha

0.318 0.134 0.401 0.792 4.96

Gapped

Lambda K H a alpha sigma

0.267 0.0410 0.140 1.90 42.6 43.6

Effective search space used: 33621452

**Query=** NODE_37_length_681_cov_0.416968

Length=681

***** No hits found *****

Lambda K H a alpha

0.318 0.134 0.401 0.792 4.96

Gapped

Lambda K H a alpha sigma

0.267 0.0410 0.140 1.90 42.6 43.6

Effective search space used: 33408658

**Query=** NODE_38_length_675_cov_0.565455

Length=675

***** No hits found *****

Lambda K H a alpha

0.318 0.134 0.401 0.792 4.96

Gapped

Lambda K H a alpha sigma

0.267 0.0410 0.140 1.90 42.6 43.6

Effective search space used: 32983070

**Query=** NODE_39_length_675_cov_0.54927

Length=675

***** No hits found *****

Lambda K H a alpha

0.318 0.134 0.401 0.792 4.96

Gapped

Lambda K H a alpha sigma

0.267 0.0410 0.140 1.90 42.6 43.6

Effective search space used: 32983070

**Query=** NODE_40_length_675_cov_0.596715

Length=675

***** No hits found *****

Lambda K H a alpha

0.318 0.134 0.401 0.792 4.96

Gapped

Lambda K H a alpha sigma

0.267 0.0410 0.140 1.90 42.6 43.6

Effective search space used: 32983070

**Query=** NODE_41_length_671_cov_0.25641

Length=671

***** No hits found *****

Lambda K H a alpha

0.318 0.134 0.401 0.792 4.96

Gapped

Lambda K H a alpha sigma

0.267 0.0410 0.140 1.90 42.6 43.6

Effective search space used: 32557482

**Query=** NODE_42_length_672_cov_0.579817

Length=672

Score E

Sequences producing significant alignments: (Bits) Value

KXA23970 23S rRNA methyltransferase [Fusobacterium nucleatum] [39.7](file:///Users/JMBNew/Desktop/Articles%20en%20Cours/Article%20Diane/Bb_SR-11-14_output-blast_named.html#BL_ORD_ID:503) 2e-05

EJU08814 florfenicol resistance protein [Fusobacterium hwasooki... [39.7](file:///Users/JMBNew/Desktop/Articles%20en%20Cours/Article%20Diane/Bb_SR-11-14_output-blast_named.html#BL_ORD_ID:444) 2e-05

NP_603423 florfenicol resistance protein [Fusobacterium nucleat... [39.7](file:///Users/JMBNew/Desktop/Articles%20en%20Cours/Article%20Diane/Bb_SR-11-14_output-blast_named.html#BL_ORD_ID:504) 2e-05

AAL94722 Florfenicol resistance protein [Fusobacterium nucleatu... [39.7](file:///Users/JMBNew/Desktop/Articles%20en%20Cours/Article%20Diane/Bb_SR-11-14_output-blast_named.html#BL_ORD_ID:6) 2e-05

EFE87464 23S rRNA m2A2503 methyltransferase [Fusobacterium peri... [38.9](file:///Users/JMBNew/Desktop/Articles%20en%20Cours/Article%20Diane/Bb_SR-11-14_output-blast_named.html#BL_ORD_ID:429) 3e-05

EHI75907 23S rRNA m2A2503 methyltransferase [Fusobacterium sp. ... [38.5](file:///Users/JMBNew/Desktop/Articles%20en%20Cours/Article%20Diane/Bb_SR-11-14_output-blast_named.html#BL_ORD_ID:440) 4e-05

EJG09268 florfenicol resistance protein [Fusobacterium nucleatu... [38.5](file:///Users/JMBNew/Desktop/Articles%20en%20Cours/Article%20Diane/Bb_SR-11-14_output-blast_named.html#BL_ORD_ID:443) 5e-05

EMP16199 florfenicol resistance protein [Fusobacterium nucleatu... [38.5](file:///Users/JMBNew/Desktop/Articles%20en%20Cours/Article%20Diane/Bb_SR-11-14_output-blast_named.html#BL_ORD_ID:456) 5e-05

ACF11531 radical SAM enzyme, Cfr family [Chlorobaculum parvum N... [38.5](file:///Users/JMBNew/Desktop/Articles%20en%20Cours/Article%20Diane/Bb_SR-11-14_output-blast_named.html#BL_ORD_ID:46) 5e-05

YP_001388569 chloramphenicol/florfenicol resistance protein [Cl... [38.5](file:///Users/JMBNew/Desktop/Articles%20en%20Cours/Article%20Diane/Bb_SR-11-14_output-blast_named.html#BL_ORD_ID:605) 5e-05

YP_001255356 chloramphenicol/florfenicol resistance protein [Cl... [38.5](file:///Users/JMBNew/Desktop/Articles%20en%20Cours/Article%20Diane/Bb_SR-11-14_output-blast_named.html#BL_ORD_ID:604) 5e-05

WP_011987126 23S rRNA (adenine(2503)-C(8))-methyltransferase Cf... [38.5](file:///Users/JMBNew/Desktop/Articles%20en%20Cours/Article%20Diane/Bb_SR-11-14_output-blast_named.html#BL_ORD_ID:550) 5e-05

OSA98255 23S rRNA (adenine(2503)-C(8))-methyltransferase Cfr [C... [38.5](file:///Users/JMBNew/Desktop/Articles%20en%20Cours/Article%20Diane/Bb_SR-11-14_output-blast_named.html#BL_ORD_ID:517) 5e-05

KON11466 chloramphenicol/florfenicol resistance protein [Clostr... [38.5](file:///Users/JMBNew/Desktop/Articles%20en%20Cours/Article%20Diane/Bb_SR-11-14_output-blast_named.html#BL_ORD_ID:481) 5e-05

EPS49069 chloramphenicol/florfenicol resistance protein [Clostr... [38.5](file:///Users/JMBNew/Desktop/Articles%20en%20Cours/Article%20Diane/Bb_SR-11-14_output-blast_named.html#BL_ORD_ID:459) 5e-05

CFR_CLOBH RecName: Full=Ribosomal RNA large subunit methyltrans... [38.5](file:///Users/JMBNew/Desktop/Articles%20en%20Cours/Article%20Diane/Bb_SR-11-14_output-blast_named.html#BL_ORD_ID:403) 5e-05

CFR_CLOB1 RecName: Full=Ribosomal RNA large subunit methyltrans... [38.5](file:///Users/JMBNew/Desktop/Articles%20en%20Cours/Article%20Diane/Bb_SR-11-14_output-blast_named.html#BL_ORD_ID:402) 5e-05

CAL84423 putative florfenicol resistance protein [Clostridium b... [38.5](file:///Users/JMBNew/Desktop/Articles%20en%20Cours/Article%20Diane/Bb_SR-11-14_output-blast_named.html#BL_ORD_ID:345) 5e-05

ABS37379 florfenicol/chloramphenicol resistance protein [Clostr... [38.5](file:///Users/JMBNew/Desktop/Articles%20en%20Cours/Article%20Diane/Bb_SR-11-14_output-blast_named.html#BL_ORD_ID:39) 5e-05

ABS35021 florfenicol/chloramphenicol resistance protein [Clostr... [38.5](file:///Users/JMBNew/Desktop/Articles%20en%20Cours/Article%20Diane/Bb_SR-11-14_output-blast_named.html#BL_ORD_ID:38) 5e-05

EYD70257 florfenicol resistance protein [Fusobacterium necropho... [38.1](file:///Users/JMBNew/Desktop/Articles%20en%20Cours/Article%20Diane/Bb_SR-11-14_output-blast_named.html#BL_ORD_ID:464) 6e-05

KXA13869 23S rRNA methyltransferase [Fusobacterium equinum] [38.1](file:///Users/JMBNew/Desktop/Articles%20en%20Cours/Article%20Diane/Bb_SR-11-14_output-blast_named.html#BL_ORD_ID:502) 6e-05

WP_061310144 Cfr family 23S rRNA (adenine(2503)-C(8))-methyltra... [37.7](file:///Users/JMBNew/Desktop/Articles%20en%20Cours/Article%20Diane/Bb_SR-11-14_output-blast_named.html#BL_ORD_ID:568) 8e-05

WP_045540636 Cfr family 23S rRNA (adenine(2503)-C(8))-methyltra... [37.7](file:///Users/JMBNew/Desktop/Articles%20en%20Cours/Article%20Diane/Bb_SR-11-14_output-blast_named.html#BL_ORD_ID:566) 8e-05

KEI76823 chloramphenicol/florfenicol resistance protein [Clostr... [37.7](file:///Users/JMBNew/Desktop/Articles%20en%20Cours/Article%20Diane/Bb_SR-11-14_output-blast_named.html#BL_ORD_ID:474) 8e-05

BAQ14476 putative chloramphenicol/florfenicol resistance protei... [37.7](file:///Users/JMBNew/Desktop/Articles%20en%20Cours/Article%20Diane/Bb_SR-11-14_output-blast_named.html#BL_ORD_ID:319) 8e-05

WP_041350656 Cfr family 23S rRNA (adenine(2503)-C(8))-methyltra... [37.7](file:///Users/JMBNew/Desktop/Articles%20en%20Cours/Article%20Diane/Bb_SR-11-14_output-blast_named.html#BL_ORD_ID:565) 8e-05

WP_015957497 23S rRNA (adenine(2503)-C(8))-methyltransferase Cf... [37.7](file:///Users/JMBNew/Desktop/Articles%20en%20Cours/Article%20Diane/Bb_SR-11-14_output-blast_named.html#BL_ORD_ID:559) 8e-05

WP_012341861 23S rRNA (adenine(2503)-C(8))-methyltransferase Cf... [37.7](file:///Users/JMBNew/Desktop/Articles%20en%20Cours/Article%20Diane/Bb_SR-11-14_output-blast_named.html#BL_ORD_ID:551) 8e-05

KIN79793 chloramphenicol/florfenicol resistance protein [Clostr... [37.7](file:///Users/JMBNew/Desktop/Articles%20en%20Cours/Article%20Diane/Bb_SR-11-14_output-blast_named.html#BL_ORD_ID:478) 8e-05

CFR_CLOBM RecName: Full=Ribosomal RNA large subunit methyltrans... [37.7](file:///Users/JMBNew/Desktop/Articles%20en%20Cours/Article%20Diane/Bb_SR-11-14_output-blast_named.html#BL_ORD_ID:405) 8e-05

CFR_CLOBK RecName: Full=Ribosomal RNA large subunit methyltrans... [37.7](file:///Users/JMBNew/Desktop/Articles%20en%20Cours/Article%20Diane/Bb_SR-11-14_output-blast_named.html#BL_ORD_ID:404) 8e-05

ACA53662 florfenicol/chloramphenicol resistance protein [Clostr... [37.7](file:///Users/JMBNew/Desktop/Articles%20en%20Cours/Article%20Diane/Bb_SR-11-14_output-blast_named.html#BL_ORD_ID:43) 8e-05

ACA43818 florfenicol/chloramphenicol resistance protein [Clostr... [37.7](file:///Users/JMBNew/Desktop/Articles%20en%20Cours/Article%20Diane/Bb_SR-11-14_output-blast_named.html#BL_ORD_ID:42) 8e-05

WP_076178522 Cfr family 23S rRNA (adenine(2503)-C(8))-methyltra... [37.7](file:///Users/JMBNew/Desktop/Articles%20en%20Cours/Article%20Diane/Bb_SR-11-14_output-blast_named.html#BL_ORD_ID:571) 8e-05

WP_024932864 Cfr family 23S rRNA (adenine(2503)-C(8))-methyltra... [37.7](file:///Users/JMBNew/Desktop/Articles%20en%20Cours/Article%20Diane/Bb_SR-11-14_output-blast_named.html#BL_ORD_ID:560) 8e-05

WP_014521578 Cfr family 23S rRNA (adenine(2503)-C(8))-methyltra... [37.7](file:///Users/JMBNew/Desktop/Articles%20en%20Cours/Article%20Diane/Bb_SR-11-14_output-blast_named.html#BL_ORD_ID:557) 8e-05

OSB14185 23S rRNA (adenine(2503)-C(8))-methyltransferase Cfr [C... [37.7](file:///Users/JMBNew/Desktop/Articles%20en%20Cours/Article%20Diane/Bb_SR-11-14_output-blast_named.html#BL_ORD_ID:518) 8e-05

OSA80008 23S rRNA (adenine(2503)-C(8))-methyltransferase Cfr [C... [37.7](file:///Users/JMBNew/Desktop/Articles%20en%20Cours/Article%20Diane/Bb_SR-11-14_output-blast_named.html#BL_ORD_ID:516) 8e-05

OSA71862 23S rRNA (adenine(2503)-C(8))-methyltransferase Cfr [C... [37.7](file:///Users/JMBNew/Desktop/Articles%20en%20Cours/Article%20Diane/Bb_SR-11-14_output-blast_named.html#BL_ORD_ID:515) 8e-05

OPD29848 23S rRNA (adenine(2503)-C8)-methyltransferase [Clostri... [37.7](file:///Users/JMBNew/Desktop/Articles%20en%20Cours/Article%20Diane/Bb_SR-11-14_output-blast_named.html#BL_ORD_ID:513) 8e-05

KOR51488 23S rRNA (adenine(2503)-C8)-methyltransferase [Clostri... [37.7](file:///Users/JMBNew/Desktop/Articles%20en%20Cours/Article%20Diane/Bb_SR-11-14_output-blast_named.html#BL_ORD_ID:482) 8e-05

KEI96147 chloramphenicol/florfenicol resistance protein [Clostr... [37.7](file:///Users/JMBNew/Desktop/Articles%20en%20Cours/Article%20Diane/Bb_SR-11-14_output-blast_named.html#BL_ORD_ID:477) 8e-05

KEI90501 chloramphenicol/florfenicol resistance protein [Clostr... [37.7](file:///Users/JMBNew/Desktop/Articles%20en%20Cours/Article%20Diane/Bb_SR-11-14_output-blast_named.html#BL_ORD_ID:476) 8e-05

KEI79595 chloramphenicol/florfenicol resistance protein [Clostr... [37.7](file:///Users/JMBNew/Desktop/Articles%20en%20Cours/Article%20Diane/Bb_SR-11-14_output-blast_named.html#BL_ORD_ID:475) 8e-05

CBZ04747 putative florfenicol resistance protein [Clostridium b... [37.7](file:///Users/JMBNew/Desktop/Articles%20en%20Cours/Article%20Diane/Bb_SR-11-14_output-blast_named.html#BL_ORD_ID:353) 8e-05

APH18681 23S rRNA (adenine(2503)-C(8))-methyltransferase Cfr [C... [37.7](file:///Users/JMBNew/Desktop/Articles%20en%20Cours/Article%20Diane/Bb_SR-11-14_output-blast_named.html#BL_ORD_ID:237) 8e-05

YP_009023638 chloramphenicol-florfenicol resistance protein, CF... [37.7](file:///Users/JMBNew/Desktop/Articles%20en%20Cours/Article%20Diane/Bb_SR-11-14_output-blast_named.html#BL_ORD_ID:641) 8e-05

YP_003896025 chloramphenicol-florfenicol resistance protein, CF... [37.7](file:///Users/JMBNew/Desktop/Articles%20en%20Cours/Article%20Diane/Bb_SR-11-14_output-blast_named.html#BL_ORD_ID:615) 8e-05

WP_013330753 Cfr family 23S rRNA (adenine(2503)-C(8))-methyltra... [37.7](file:///Users/JMBNew/Desktop/Articles%20en%20Cours/Article%20Diane/Bb_SR-11-14_output-blast_named.html#BL_ORD_ID:554) 8e-05

ALI92641 Cfr (plasmid) [Enterococcus faecalis] [37.7](file:///Users/JMBNew/Desktop/Articles%20en%20Cours/Article%20Diane/Bb_SR-11-14_output-blast_named.html#BL_ORD_ID:215) 8e-05

AIA99220 chloramphenicol-florfenicol resistance protein, CFR (p... [37.7](file:///Users/JMBNew/Desktop/Articles%20en%20Cours/Article%20Diane/Bb_SR-11-14_output-blast_named.html#BL_ORD_ID:161) 8e-05

AHN52249 chloramphenicol-florfenicol resistance protein, CFR (p... [37.7](file:///Users/JMBNew/Desktop/Articles%20en%20Cours/Article%20Diane/Bb_SR-11-14_output-blast_named.html#BL_ORD_ID:160) 8e-05

ADN34770 chloramphenicol-florfenicol resistance protein, CFR (p... [37.7](file:///Users/JMBNew/Desktop/Articles%20en%20Cours/Article%20Diane/Bb_SR-11-14_output-blast_named.html#BL_ORD_ID:66) 8e-05

ARX61578 23S ribosomal RNA methyltransferase (plasmid) [Escheri... [37.7](file:///Users/JMBNew/Desktop/Articles%20en%20Cours/Article%20Diane/Bb_SR-11-14_output-blast_named.html#BL_ORD_ID:293) 8e-05

ARX61545 florfenicol/chloramphenicol resistance (plasmid) [Esch... [37.7](file:///Users/JMBNew/Desktop/Articles%20en%20Cours/Article%20Diane/Bb_SR-11-14_output-blast_named.html#BL_ORD_ID:292) 8e-05

ALJ52404 Cfr (plasmid) [Escherichia coli] [37.7](file:///Users/JMBNew/Desktop/Articles%20en%20Cours/Article%20Diane/Bb_SR-11-14_output-blast_named.html#BL_ORD_ID:217) 8e-05

AIY22804 florfenicol/chloramphenicol resistance protein (plasmi... [37.7](file:///Users/JMBNew/Desktop/Articles%20en%20Cours/Article%20Diane/Bb_SR-11-14_output-blast_named.html#BL_ORD_ID:183) 8e-05

WP_030037108 Cfr family 23S rRNA (adenine(2503)-C(8))-methyltra... [37.7](file:///Users/JMBNew/Desktop/Articles%20en%20Cours/Article%20Diane/Bb_SR-11-14_output-blast_named.html#BL_ORD_ID:561) 8e-05

WP_003405508 23S rRNA (adenine(2503)-C(8))-methyltransferase Cf... [37.7](file:///Users/JMBNew/Desktop/Articles%20en%20Cours/Article%20Diane/Bb_SR-11-14_output-blast_named.html#BL_ORD_ID:543) 8e-05

GAE03833 chloramphenicol/florfenicol resistance protein [Clostr... [37.7](file:///Users/JMBNew/Desktop/Articles%20en%20Cours/Article%20Diane/Bb_SR-11-14_output-blast_named.html#BL_ORD_ID:468) 8e-05

EKX78206 chloramphenicol/florfenicol resistance protein [Clostr... [37.7](file:///Users/JMBNew/Desktop/Articles%20en%20Cours/Article%20Diane/Bb_SR-11-14_output-blast_named.html#BL_ORD_ID:454) 8e-05

cfr_-_17035810_translation cfr: florfenicol/chloramphenicol res... [37.7](file:///Users/JMBNew/Desktop/Articles%20en%20Cours/Article%20Diane/Bb_SR-11-14_output-blast_named.html#BL_ORD_ID:643) 8e-05

YP_008575034 florfenicol/chloramphenicol resistance protein (pl... [37.7](file:///Users/JMBNew/Desktop/Articles%20en%20Cours/Article%20Diane/Bb_SR-11-14_output-blast_named.html#BL_ORD_ID:638) 8e-05

YP_007988851 23S rRNA methylase (plasmid) [Staphylococcus aureus] [37.7](file:///Users/JMBNew/Desktop/Articles%20en%20Cours/Article%20Diane/Bb_SR-11-14_output-blast_named.html#BL_ORD_ID:634) 8e-05

YP_007878372 23S rRNA methylase (plasmid) [Staphylococcus aureus] [37.7](file:///Users/JMBNew/Desktop/Articles%20en%20Cours/Article%20Diane/Bb_SR-11-14_output-blast_named.html#BL_ORD_ID:633) 8e-05

YP_004888091 23S rRNA methylase (plasmid) [Staphylococcus arlet... [37.7](file:///Users/JMBNew/Desktop/Articles%20en%20Cours/Article%20Diane/Bb_SR-11-14_output-blast_named.html#BL_ORD_ID:624) 8e-05

YP_003927883 rRNA methylase (plasmid) [Bacillus sp. BS-02] [37.7](file:///Users/JMBNew/Desktop/Articles%20en%20Cours/Article%20Diane/Bb_SR-11-14_output-blast_named.html#BL_ORD_ID:616) 8e-05

WP_032491462 23S rRNA (adenine(2503)-C(8))-methyltransferase Cf... [37.7](file:///Users/JMBNew/Desktop/Articles%20en%20Cours/Article%20Diane/Bb_SR-11-14_output-blast_named.html#BL_ORD_ID:562) 8e-05

WP_001010505 MULTISPECIES: 23S rRNA (adenine(2503)-C(8))-methyl... [37.7](file:///Users/JMBNew/Desktop/Articles%20en%20Cours/Article%20Diane/Bb_SR-11-14_output-blast_named.html#BL_ORD_ID:539) 8e-05

NP_899167 florfenicol/chloramphenicol resistance protein (plasm... [37.7](file:///Users/JMBNew/Desktop/Articles%20en%20Cours/Article%20Diane/Bb_SR-11-14_output-blast_named.html#BL_ORD_ID:505) 8e-05

EXP53712 ribosomal RNA large subunit methyltransferase Cfr [Sta... [37.7](file:///Users/JMBNew/Desktop/Articles%20en%20Cours/Article%20Diane/Bb_SR-11-14_output-blast_named.html#BL_ORD_ID:463) 8e-05

EXO68354 ribosomal RNA large subunit methyltransferase Cfr [Sta... [37.7](file:///Users/JMBNew/Desktop/Articles%20en%20Cours/Article%20Diane/Bb_SR-11-14_output-blast_named.html#BL_ORD_ID:462) 8e-05

CFR_STAWA RecName: Full=Ribosomal RNA large subunit methyltrans... [37.7](file:///Users/JMBNew/Desktop/Articles%20en%20Cours/Article%20Diane/Bb_SR-11-14_output-blast_named.html#BL_ORD_ID:408) 8e-05

CFR_STASC RecName: Full=Ribosomal RNA large subunit methyltrans... [37.7](file:///Users/JMBNew/Desktop/Articles%20en%20Cours/Article%20Diane/Bb_SR-11-14_output-blast_named.html#BL_ORD_ID:407) 8e-05

CFR_STAAU RecName: Full=Ribosomal RNA large subunit methyltrans... [37.7](file:///Users/JMBNew/Desktop/Articles%20en%20Cours/Article%20Diane/Bb_SR-11-14_output-blast_named.html#BL_ORD_ID:406) 8e-05

CBW44219 rRNA methylase (plasmid) [Staphylococcus aureus] [37.7](file:///Users/JMBNew/Desktop/Articles%20en%20Cours/Article%20Diane/Bb_SR-11-14_output-blast_named.html#BL_ORD_ID:352) 8e-05

CBN88266 rRNA methylase [Staphylococcus aureus] [37.7](file:///Users/JMBNew/Desktop/Articles%20en%20Cours/Article%20Diane/Bb_SR-11-14_output-blast_named.html#BL_ORD_ID:351) 8e-05

CAL64019 rRNA methylase (plasmid) [Staphylococcus warneri] [37.7](file:///Users/JMBNew/Desktop/Articles%20en%20Cours/Article%20Diane/Bb_SR-11-14_output-blast_named.html#BL_ORD_ID:344) 8e-05

CAJ30491 23S rRNA methylase (plasmid) [Staphylococcus aureus] [37.7](file:///Users/JMBNew/Desktop/Articles%20en%20Cours/Article%20Diane/Bb_SR-11-14_output-blast_named.html#BL_ORD_ID:331) 8e-05

CAI56203 methyltransferase (plasmid) [Staphylococcus aureus] [37.7](file:///Users/JMBNew/Desktop/Articles%20en%20Cours/Article%20Diane/Bb_SR-11-14_output-blast_named.html#BL_ORD_ID:330) 8e-05

CAE18142 florfenicol/chloramphenicol resistance protein (plasmi... [37.7](file:///Users/JMBNew/Desktop/Articles%20en%20Cours/Article%20Diane/Bb_SR-11-14_output-blast_named.html#BL_ORD_ID:326) 8e-05

CAC04525 florfenicol resistance protein (plasmid) [Staphylococc... [37.7](file:///Users/JMBNew/Desktop/Articles%20en%20Cours/Article%20Diane/Bb_SR-11-14_output-blast_named.html#BL_ORD_ID:323) 8e-05

ARQ19363 chloramphenicol-florfenicol resistance protein (plasmi... [37.7](file:///Users/JMBNew/Desktop/Articles%20en%20Cours/Article%20Diane/Bb_SR-11-14_output-blast_named.html#BL_ORD_ID:290) 8e-05

ARA73644 23S ribosomal RNA methyltransferase Cfr (plasmid) [Sta... [37.7](file:///Users/JMBNew/Desktop/Articles%20en%20Cours/Article%20Diane/Bb_SR-11-14_output-blast_named.html#BL_ORD_ID:286) 8e-05

AQZ36678 23S rRNA methylase (plasmid) [Staphylococcus sciuri] [37.7](file:///Users/JMBNew/Desktop/Articles%20en%20Cours/Article%20Diane/Bb_SR-11-14_output-blast_named.html#BL_ORD_ID:285) 8e-05

AQW34743 23S rRNA methylase [Staphylococcus sciuri] [37.7](file:///Users/JMBNew/Desktop/Articles%20en%20Cours/Article%20Diane/Bb_SR-11-14_output-blast_named.html#BL_ORD_ID:277) 8e-05

AQW34729 23S rRNA methylase [Staphylococcus sciuri] [37.7](file:///Users/JMBNew/Desktop/Articles%20en%20Cours/Article%20Diane/Bb_SR-11-14_output-blast_named.html#BL_ORD_ID:273) 8e-05

AQW34711 23S rRNA methylase [Staphylococcus sciuri] [37.7](file:///Users/JMBNew/Desktop/Articles%20en%20Cours/Article%20Diane/Bb_SR-11-14_output-blast_named.html#BL_ORD_ID:269) 8e-05

AQW34676 23S rRNA methylase (plasmid) [Staphylococcus sciuri] [37.7](file:///Users/JMBNew/Desktop/Articles%20en%20Cours/Article%20Diane/Bb_SR-11-14_output-blast_named.html#BL_ORD_ID:263) 8e-05

AQW34623 23S rRNA methylase (plasmid) [Staphylococcus sciuri] [37.7](file:///Users/JMBNew/Desktop/Articles%20en%20Cours/Article%20Diane/Bb_SR-11-14_output-blast_named.html#BL_ORD_ID:252) 8e-05

AQW34576 23S rRNA methylase [Staphylococcus sciuri] [37.7](file:///Users/JMBNew/Desktop/Articles%20en%20Cours/Article%20Diane/Bb_SR-11-14_output-blast_named.html#BL_ORD_ID:242) 8e-05

AMN16502 Cfr (plasmid) [Staphylococcus aureus subsp. aureus] [37.7](file:///Users/JMBNew/Desktop/Articles%20en%20Cours/Article%20Diane/Bb_SR-11-14_output-blast_named.html#BL_ORD_ID:226) 8e-05

AMN16461 chloramphenicol-florfenicol resistance protein CFR (pl... [37.7](file:///Users/JMBNew/Desktop/Articles%20en%20Cours/Article%20Diane/Bb_SR-11-14_output-blast_named.html#BL_ORD_ID:225) 8e-05

ALI92801 23S rRNA methylase (plasmid) [Staphylococcus sciuri] [37.7](file:///Users/JMBNew/Desktop/Articles%20en%20Cours/Article%20Diane/Bb_SR-11-14_output-blast_named.html#BL_ORD_ID:216) 8e-05

ALF95990 rRNA methyltransferase (plasmid) [Staphylococcus aureus] [37.7](file:///Users/JMBNew/Desktop/Articles%20en%20Cours/Article%20Diane/Bb_SR-11-14_output-blast_named.html#BL_ORD_ID:210) 8e-05

ALF95987 rRNA methyltransferase (plasmid) [Staphylococcus epide... [37.7](file:///Users/JMBNew/Desktop/Articles%20en%20Cours/Article%20Diane/Bb_SR-11-14_output-blast_named.html#BL_ORD_ID:209) 8e-05

ALE30190 23S rRNA methylase (plasmid) [Staphylococcus simulans] [37.7](file:///Users/JMBNew/Desktop/Articles%20en%20Cours/Article%20Diane/Bb_SR-11-14_output-blast_named.html#BL_ORD_ID:208) 8e-05

AKL80218 23S rRNA methylase (plasmid) [Staphylococcus xylosus] [37.7](file:///Users/JMBNew/Desktop/Articles%20en%20Cours/Article%20Diane/Bb_SR-11-14_output-blast_named.html#BL_ORD_ID:196) 8e-05

AKE50930 23S rRNA methylase [Staphylococcus aureus] [37.7](file:///Users/JMBNew/Desktop/Articles%20en%20Cours/Article%20Diane/Bb_SR-11-14_output-blast_named.html#BL_ORD_ID:192) 8e-05

AJW29170 rRNA methyltransferase (plasmid) [Staphylococcus epide... [37.7](file:///Users/JMBNew/Desktop/Articles%20en%20Cours/Article%20Diane/Bb_SR-11-14_output-blast_named.html#BL_ORD_ID:191) 8e-05

AJW29120 rRNA methyltransferase (plasmid) [Staphylococcus epide... [37.7](file:///Users/JMBNew/Desktop/Articles%20en%20Cours/Article%20Diane/Bb_SR-11-14_output-blast_named.html#BL_ORD_ID:190) 8e-05

AJQ17306 Cfr (plasmid) [Escherichia coli] [37.7](file:///Users/JMBNew/Desktop/Articles%20en%20Cours/Article%20Diane/Bb_SR-11-14_output-blast_named.html#BL_ORD_ID:188) 8e-05

AJM87289 23S rRNA methylase (plasmid) [Staphylococcus aureus] [37.7](file:///Users/JMBNew/Desktop/Articles%20en%20Cours/Article%20Diane/Bb_SR-11-14_output-blast_named.html#BL_ORD_ID:187) 8e-05

AJF83635 florfenicol/chloramphenicol resistance protein (plasmi... [37.7](file:///Users/JMBNew/Desktop/Articles%20en%20Cours/Article%20Diane/Bb_SR-11-14_output-blast_named.html#BL_ORD_ID:186) 8e-05

AJF83598 florfenicol/chloramphenicol resistance protein (plasmi... [37.7](file:///Users/JMBNew/Desktop/Articles%20en%20Cours/Article%20Diane/Bb_SR-11-14_output-blast_named.html#BL_ORD_ID:185) 8e-05

AIT41446 Cfr (plasmid) [Staphylococcus aureus] [37.7](file:///Users/JMBNew/Desktop/Articles%20en%20Cours/Article%20Diane/Bb_SR-11-14_output-blast_named.html#BL_ORD_ID:176) 8e-05

AIT38258 23S rRNA methyltransferase [Staphylococcus cohnii] [37.7](file:///Users/JMBNew/Desktop/Articles%20en%20Cours/Article%20Diane/Bb_SR-11-14_output-blast_named.html#BL_ORD_ID:175) 8e-05

AIL01330 Cfr [Staphylococcus aureus] [37.7](file:///Users/JMBNew/Desktop/Articles%20en%20Cours/Article%20Diane/Bb_SR-11-14_output-blast_named.html#BL_ORD_ID:171) 8e-05

AIL01327 Cfr [Staphylococcus aureus] [37.7](file:///Users/JMBNew/Desktop/Articles%20en%20Cours/Article%20Diane/Bb_SR-11-14_output-blast_named.html#BL_ORD_ID:170) 8e-05

AIL01324 Cfr [Staphylococcus aureus] [37.7](file:///Users/JMBNew/Desktop/Articles%20en%20Cours/Article%20Diane/Bb_SR-11-14_output-blast_named.html#BL_ORD_ID:169) 8e-05

AIG88478 rRNA methylase Cfr (plasmid) [Escherichia coli] [37.7](file:///Users/JMBNew/Desktop/Articles%20en%20Cours/Article%20Diane/Bb_SR-11-14_output-blast_named.html#BL_ORD_ID:168) 8e-05

AIG88475 rRNA methylase Cfr (plasmid) [Escherichia coli] [37.7](file:///Users/JMBNew/Desktop/Articles%20en%20Cours/Article%20Diane/Bb_SR-11-14_output-blast_named.html#BL_ORD_ID:167) 8e-05

AIG88472 rRNA methylase Cfr (plasmid) [Escherichia coli] [37.7](file:///Users/JMBNew/Desktop/Articles%20en%20Cours/Article%20Diane/Bb_SR-11-14_output-blast_named.html#BL_ORD_ID:166) 8e-05

AIG88468 rRNA methylase Cfr (plasmid) [Escherichia coli] [37.7](file:///Users/JMBNew/Desktop/Articles%20en%20Cours/Article%20Diane/Bb_SR-11-14_output-blast_named.html#BL_ORD_ID:165) 8e-05

AIA99243 chloramphenicol-florfenicol resistance protein, CFR [E... [37.7](file:///Users/JMBNew/Desktop/Articles%20en%20Cours/Article%20Diane/Bb_SR-11-14_output-blast_named.html#BL_ORD_ID:163) 8e-05

AIA99231 chloramphenicol-florfenicol resistance protein, CFR [E... [37.7](file:///Users/JMBNew/Desktop/Articles%20en%20Cours/Article%20Diane/Bb_SR-11-14_output-blast_named.html#BL_ORD_ID:162) 8e-05

AHL28472 23S rRNA methylase (plasmid) [Staphylococcus simulans] [37.7](file:///Users/JMBNew/Desktop/Articles%20en%20Cours/Article%20Diane/Bb_SR-11-14_output-blast_named.html#BL_ORD_ID:159) 8e-05

AHL28467 23S rRNA methylase (plasmid) [Staphylococcus simulans] [37.7](file:///Users/JMBNew/Desktop/Articles%20en%20Cours/Article%20Diane/Bb_SR-11-14_output-blast_named.html#BL_ORD_ID:158) 8e-05

AHL28458 23S rRNA methylase (plasmid) [Staphylococcus simulans] [37.7](file:///Users/JMBNew/Desktop/Articles%20en%20Cours/Article%20Diane/Bb_SR-11-14_output-blast_named.html#BL_ORD_ID:157) 8e-05

AHJ80329 23S rRNA methylase (plasmid) [Staphylococcus aureus] [37.7](file:///Users/JMBNew/Desktop/Articles%20en%20Cours/Article%20Diane/Bb_SR-11-14_output-blast_named.html#BL_ORD_ID:156) 8e-05

AHI16978 23S rRNA methylase (plasmid) [Staphylococcus equorum] [37.7](file:///Users/JMBNew/Desktop/Articles%20en%20Cours/Article%20Diane/Bb_SR-11-14_output-blast_named.html#BL_ORD_ID:155) 8e-05

AHI16972 23S rRNA methylase (plasmid) [Staphylococcus sciuri] [37.7](file:///Users/JMBNew/Desktop/Articles%20en%20Cours/Article%20Diane/Bb_SR-11-14_output-blast_named.html#BL_ORD_ID:154) 8e-05

AHB87388 23S rRNA methylase (plasmid) [Staphylococcus rostri] [37.7](file:///Users/JMBNew/Desktop/Articles%20en%20Cours/Article%20Diane/Bb_SR-11-14_output-blast_named.html#BL_ORD_ID:149) 8e-05

AGZ63429 23S rRNA methylase [Staphylococcus lentus] [37.7](file:///Users/JMBNew/Desktop/Articles%20en%20Cours/Article%20Diane/Bb_SR-11-14_output-blast_named.html#BL_ORD_ID:146) 8e-05

AGZ63423 florfenicol/chloramphenicol resistance protein [Staphy... [37.7](file:///Users/JMBNew/Desktop/Articles%20en%20Cours/Article%20Diane/Bb_SR-11-14_output-blast_named.html#BL_ORD_ID:145) 8e-05

AGW01079 florfenicol/chloramphenicol resistance protein (plasmi... [37.7](file:///Users/JMBNew/Desktop/Articles%20en%20Cours/Article%20Diane/Bb_SR-11-14_output-blast_named.html#BL_ORD_ID:143) 8e-05

AGR88944 florfenicol/chloramphenicol resistance protein (plasmi... [37.7](file:///Users/JMBNew/Desktop/Articles%20en%20Cours/Article%20Diane/Bb_SR-11-14_output-blast_named.html#BL_ORD_ID:140) 8e-05

AGR88927 23S rRNA methylase (plasmid) [Staphylococcus lentus] [37.7](file:///Users/JMBNew/Desktop/Articles%20en%20Cours/Article%20Diane/Bb_SR-11-14_output-blast_named.html#BL_ORD_ID:139) 8e-05

AGR88915 florfenicol/chloramphenicol resistance protein [Staphy... [37.7](file:///Users/JMBNew/Desktop/Articles%20en%20Cours/Article%20Diane/Bb_SR-11-14_output-blast_named.html#BL_ORD_ID:138) 8e-05

AGQ80867 23S rRNA methylase (plasmid) [Staphylococcus cohnii] [37.7](file:///Users/JMBNew/Desktop/Articles%20en%20Cours/Article%20Diane/Bb_SR-11-14_output-blast_named.html#BL_ORD_ID:137) 8e-05

AGQ80838 23S rRNA methyltransferase (plasmid) [Staphylococcus c... [37.7](file:///Users/JMBNew/Desktop/Articles%20en%20Cours/Article%20Diane/Bb_SR-11-14_output-blast_named.html#BL_ORD_ID:136) 8e-05

AGO02198 florfenicol/chloramphenicol resistance protein (plasmi... [37.7](file:///Users/JMBNew/Desktop/Articles%20en%20Cours/Article%20Diane/Bb_SR-11-14_output-blast_named.html#BL_ORD_ID:131) 8e-05

AGL42330 23S rRNA methylase (plasmid) [Staphylococcus aureus] [37.7](file:///Users/JMBNew/Desktop/Articles%20en%20Cours/Article%20Diane/Bb_SR-11-14_output-blast_named.html#BL_ORD_ID:130) 8e-05

AGJ70604 23S rRNA methylase (plasmid) [Staphylococcus epidermidis] [37.7](file:///Users/JMBNew/Desktop/Articles%20en%20Cours/Article%20Diane/Bb_SR-11-14_output-blast_named.html#BL_ORD_ID:127) 8e-05

AGH72126 chloramphenicol-florfenicol resistance protein (plasmi... [37.7](file:///Users/JMBNew/Desktop/Articles%20en%20Cours/Article%20Diane/Bb_SR-11-14_output-blast_named.html#BL_ORD_ID:126) 8e-05

AGH12814 23S rRNA methylase (plasmid) [Staphylococcus haemolyti... [37.7](file:///Users/JMBNew/Desktop/Articles%20en%20Cours/Article%20Diane/Bb_SR-11-14_output-blast_named.html#BL_ORD_ID:123) 8e-05

AFW17901 rRNA methylase (plasmid) [Enterococcus faecalis] [37.7](file:///Users/JMBNew/Desktop/Articles%20en%20Cours/Article%20Diane/Bb_SR-11-14_output-blast_named.html#BL_ORD_ID:121) 8e-05

AFW17874 rRNA methylase (plasmid) [Enterococcus thailandicus] [37.7](file:///Users/JMBNew/Desktop/Articles%20en%20Cours/Article%20Diane/Bb_SR-11-14_output-blast_named.html#BL_ORD_ID:120) 8e-05

AFO64605 23S rRNA methylase (plasmid) [Jeotgalicoccus pinnipedi... [37.7](file:///Users/JMBNew/Desktop/Articles%20en%20Cours/Article%20Diane/Bb_SR-11-14_output-blast_named.html#BL_ORD_ID:117) 8e-05

AFJ49143 florfenicol/chloramphenicol resistance protein (plasmi... [37.7](file:///Users/JMBNew/Desktop/Articles%20en%20Cours/Article%20Diane/Bb_SR-11-14_output-blast_named.html#BL_ORD_ID:114) 8e-05

AFH78457 florfenicol/chloramphenicol resistance protein (plasmi... [37.7](file:///Users/JMBNew/Desktop/Articles%20en%20Cours/Article%20Diane/Bb_SR-11-14_output-blast_named.html#BL_ORD_ID:112) 8e-05

AFG17053 Cfr (plasmid) [Enterococcus faecalis] [37.7](file:///Users/JMBNew/Desktop/Articles%20en%20Cours/Article%20Diane/Bb_SR-11-14_output-blast_named.html#BL_ORD_ID:108) 8e-05

AEX20241 23S rRNA methylase (plasmid) [Staphylococcus cohnii] [37.7](file:///Users/JMBNew/Desktop/Articles%20en%20Cours/Article%20Diane/Bb_SR-11-14_output-blast_named.html#BL_ORD_ID:100) 8e-05

AEX07579 23S rRNA methylase (plasmid) [Staphylococcus cohnii] [37.7](file:///Users/JMBNew/Desktop/Articles%20en%20Cours/Article%20Diane/Bb_SR-11-14_output-blast_named.html#BL_ORD_ID:98) 8e-05

AEX01232 rRNA methylase [Staphylococcus cohnii] [37.7](file:///Users/JMBNew/Desktop/Articles%20en%20Cours/Article%20Diane/Bb_SR-11-14_output-blast_named.html#BL_ORD_ID:97) 8e-05

AEX01231 rRNA methylase [Staphylococcus haemolyticus] [37.7](file:///Users/JMBNew/Desktop/Articles%20en%20Cours/Article%20Diane/Bb_SR-11-14_output-blast_named.html#BL_ORD_ID:96) 8e-05

AEW23132 Cfr (plasmid) [Staphylococcus aureus] [37.7](file:///Users/JMBNew/Desktop/Articles%20en%20Cours/Article%20Diane/Bb_SR-11-14_output-blast_named.html#BL_ORD_ID:93) 8e-05

AET37217 Cfr [Staphylococcus aureus] [37.7](file:///Users/JMBNew/Desktop/Articles%20en%20Cours/Article%20Diane/Bb_SR-11-14_output-blast_named.html#BL_ORD_ID:89) 8e-05

AER50875 rRNA methylase [Proteus vulgaris] [37.7](file:///Users/JMBNew/Desktop/Articles%20en%20Cours/Article%20Diane/Bb_SR-11-14_output-blast_named.html#BL_ORD_ID:87) 8e-05

AEP69239 23S rRNA methylase (plasmid) [Staphylococcus arlettae] [37.7](file:///Users/JMBNew/Desktop/Articles%20en%20Cours/Article%20Diane/Bb_SR-11-14_output-blast_named.html#BL_ORD_ID:85) 8e-05

AEP69232 23S rRNA methylase (plasmid) [Staphylococcus saprophyt... [37.7](file:///Users/JMBNew/Desktop/Articles%20en%20Cours/Article%20Diane/Bb_SR-11-14_output-blast_named.html#BL_ORD_ID:84) 8e-05

AEP69220 23S rRNA methylase (plasmid) [Staphylococcus cohnii] [37.7](file:///Users/JMBNew/Desktop/Articles%20en%20Cours/Article%20Diane/Bb_SR-11-14_output-blast_named.html#BL_ORD_ID:83) 8e-05

ADN44269 rRNA methylase (plasmid) [Bacillus sp. BS-02] [37.7](file:///Users/JMBNew/Desktop/Articles%20en%20Cours/Article%20Diane/Bb_SR-11-14_output-blast_named.html#BL_ORD_ID:67) 8e-05

ACC77590 23S rRNA methylase (plasmid) [Staphylococcus aureus] [37.7](file:///Users/JMBNew/Desktop/Articles%20en%20Cours/Article%20Diane/Bb_SR-11-14_output-blast_named.html#BL_ORD_ID:44) 8e-05

ABQ00063 Cfr [Staphylococcus aureus] [37.7](file:///Users/JMBNew/Desktop/Articles%20en%20Cours/Article%20Diane/Bb_SR-11-14_output-blast_named.html#BL_ORD_ID:33) 8e-05

WP_003494750 Cfr family 23S rRNA (adenine(2503)-C(8))-methyltra... [37.7](file:///Users/JMBNew/Desktop/Articles%20en%20Cours/Article%20Diane/Bb_SR-11-14_output-blast_named.html#BL_ORD_ID:544) 8e-05

EHN14160 chloramphenicol/florfenicol resistance protein [Clostr... [37.7](file:///Users/JMBNew/Desktop/Articles%20en%20Cours/Article%20Diane/Bb_SR-11-14_output-blast_named.html#BL_ORD_ID:441) 8e-05

ALG88812 florfenicol/chloramphenicol resistance protein (plasmi... [37.7](file:///Users/JMBNew/Desktop/Articles%20en%20Cours/Article%20Diane/Bb_SR-11-14_output-blast_named.html#BL_ORD_ID:212) 8e-05

ALG88797 florfenicol/chloramphenicol resistance protein [Escher... [37.7](file:///Users/JMBNew/Desktop/Articles%20en%20Cours/Article%20Diane/Bb_SR-11-14_output-blast_named.html#BL_ORD_ID:211) 8e-05

AFR11502 rRNA methylase (plasmid) [Staphylococcus capitis] [37.7](file:///Users/JMBNew/Desktop/Articles%20en%20Cours/Article%20Diane/Bb_SR-11-14_output-blast_named.html#BL_ORD_ID:118) 8e-05

cfr_-_8918998_translation cfr: rRNA methylase [37.7](file:///Users/JMBNew/Desktop/Articles%20en%20Cours/Article%20Diane/Bb_SR-11-14_output-blast_named.html#BL_ORD_ID:642) 8e-05

YP_003533077 rRNA methylase (plasmid) [Bacillus sp. BS-01] [37.7](file:///Users/JMBNew/Desktop/Articles%20en%20Cours/Article%20Diane/Bb_SR-11-14_output-blast_named.html#BL_ORD_ID:614) 8e-05

WP_013034930 Cfr family 23S rRNA (adenine(2503)-C(8))-methyltra... [37.7](file:///Users/JMBNew/Desktop/Articles%20en%20Cours/Article%20Diane/Bb_SR-11-14_output-blast_named.html#BL_ORD_ID:553) 8e-05

ADD91311 rRNA methylase (plasmid) [Bacillus sp. BS-01] [37.7](file:///Users/JMBNew/Desktop/Articles%20en%20Cours/Article%20Diane/Bb_SR-11-14_output-blast_named.html#BL_ORD_ID:63) 8e-05

ARQ19305 chloramphenicol-florfenicol resistance protein (plasmi... [37.7](file:///Users/JMBNew/Desktop/Articles%20en%20Cours/Article%20Diane/Bb_SR-11-14_output-blast_named.html#BL_ORD_ID:289) 8e-05

YP_006958115 rRNA methylase (plasmid) [Staphylococcus aureus] [37.7](file:///Users/JMBNew/Desktop/Articles%20en%20Cours/Article%20Diane/Bb_SR-11-14_output-blast_named.html#BL_ORD_ID:629) 8e-05

WP_001835153 Cfr family 23S rRNA (adenine(2503)-C(8))-methyltra... [37.7](file:///Users/JMBNew/Desktop/Articles%20en%20Cours/Article%20Diane/Bb_SR-11-14_output-blast_named.html#BL_ORD_ID:540) 8e-05

EJU86205 23S rRNA methyltransferase [Enterococcus faecalis 599] [37.7](file:///Users/JMBNew/Desktop/Articles%20en%20Cours/Article%20Diane/Bb_SR-11-14_output-blast_named.html#BL_ORD_ID:446) 8e-05

EJU83061 23S rRNA methyltransferase [Staphylococcus aureus subs... [37.7](file:///Users/JMBNew/Desktop/Articles%20en%20Cours/Article%20Diane/Bb_SR-11-14_output-blast_named.html#BL_ORD_ID:445) 8e-05

AKJ75143 rRNA methylase (plasmid) [Staphylococcus epidermidis] [37.7](file:///Users/JMBNew/Desktop/Articles%20en%20Cours/Article%20Diane/Bb_SR-11-14_output-blast_named.html#BL_ORD_ID:195) 8e-05

AGB06240 Cfr methyltransferase [Staphylococcus epidermidis] [37.7](file:///Users/JMBNew/Desktop/Articles%20en%20Cours/Article%20Diane/Bb_SR-11-14_output-blast_named.html#BL_ORD_ID:122) 8e-05

AFW17854 rRNA methylase (plasmid) [Enterococcus thailandicus] [37.7](file:///Users/JMBNew/Desktop/Articles%20en%20Cours/Article%20Diane/Bb_SR-11-14_output-blast_named.html#BL_ORD_ID:119) 8e-05

AFF18416 rRNA methylase (plasmid) [Staphylococcus aureus] [37.7](file:///Users/JMBNew/Desktop/Articles%20en%20Cours/Article%20Diane/Bb_SR-11-14_output-blast_named.html#BL_ORD_ID:107) 8e-05

WP_002349981 23S rRNA (adenine(2503)-C(8))-methyltransferase Cf... [37.4](file:///Users/JMBNew/Desktop/Articles%20en%20Cours/Article%20Diane/Bb_SR-11-14_output-blast_named.html#BL_ORD_ID:541) 1e-04

EJX57584 23S rRNA methyltransferase [Enterococcus faecium R497] [37.4](file:///Users/JMBNew/Desktop/Articles%20en%20Cours/Article%20Diane/Bb_SR-11-14_output-blast_named.html#BL_ORD_ID:447) 1e-04

AKV84429 chloramphenicol/ fluorphenicol resistance protein [Ent... [37.4](file:///Users/JMBNew/Desktop/Articles%20en%20Cours/Article%20Diane/Bb_SR-11-14_output-blast_named.html#BL_ORD_ID:206) 1e-04

AIX48090 23S methyltransferase [Clostridioides difficile] [37.4](file:///Users/JMBNew/Desktop/Articles%20en%20Cours/Article%20Diane/Bb_SR-11-14_output-blast_named.html#BL_ORD_ID:181) 1e-04

WP_002405682 MULTISPECIES: Cfr family 23S rRNA (adenine(2503)-C... [37.4](file:///Users/JMBNew/Desktop/Articles%20en%20Cours/Article%20Diane/Bb_SR-11-14_output-blast_named.html#BL_ORD_ID:542) 1e-04

EQF87020 ribosomal RNA large subunit methyltransferase N [Clost... [37.4](file:///Users/JMBNew/Desktop/Articles%20en%20Cours/Article%20Diane/Bb_SR-11-14_output-blast_named.html#BL_ORD_ID:460) 1e-04

EOI92877 ribosomal RNA large subunit methyltransferase Cfr [Ent... [37.4](file:///Users/JMBNew/Desktop/Articles%20en%20Cours/Article%20Diane/Bb_SR-11-14_output-blast_named.html#BL_ORD_ID:458) 1e-04

EOI25076 ribosomal RNA large subunit methyltransferase Cfr [Ent... [37.4](file:///Users/JMBNew/Desktop/Articles%20en%20Cours/Article%20Diane/Bb_SR-11-14_output-blast_named.html#BL_ORD_ID:457) 1e-04

EFQ16578 23S rRNA m2A2503 methyltransferase [Enterococcus faeca... [37.4](file:///Users/JMBNew/Desktop/Articles%20en%20Cours/Article%20Diane/Bb_SR-11-14_output-blast_named.html#BL_ORD_ID:437) 1e-04

CDF47262 chloramphenicol/fluorfenicol resistance protein, radic... [37.4](file:///Users/JMBNew/Desktop/Articles%20en%20Cours/Article%20Diane/Bb_SR-11-14_output-blast_named.html#BL_ORD_ID:372) 1e-04

AIX48091 23S methyltransferase [Clostridioides difficile] [37.4](file:///Users/JMBNew/Desktop/Articles%20en%20Cours/Article%20Diane/Bb_SR-11-14_output-blast_named.html#BL_ORD_ID:182) 1e-04

WP_053594538 Cfr family 23S rRNA (adenine(2503)-C(8))-methyltra... [37.0](file:///Users/JMBNew/Desktop/Articles%20en%20Cours/Article%20Diane/Bb_SR-11-14_output-blast_named.html#BL_ORD_ID:567) 1e-04

KOS62985 23S rRNA (adenine(2503)-C8)-methyltransferase [Lysinib... [37.0](file:///Users/JMBNew/Desktop/Articles%20en%20Cours/Article%20Diane/Bb_SR-11-14_output-blast_named.html#BL_ORD_ID:483) 1e-04

WP_094423516 Cfr family 23S rRNA (adenine(2503)-C(8))-methyltra... [36.6](file:///Users/JMBNew/Desktop/Articles%20en%20Cours/Article%20Diane/Bb_SR-11-14_output-blast_named.html#BL_ORD_ID:573) 2e-04

AST94603 23S rRNA (adenine(2503)-C(8))-methyltransferase Cfr [B... [36.6](file:///Users/JMBNew/Desktop/Articles%20en%20Cours/Article%20Diane/Bb_SR-11-14_output-blast_named.html#BL_ORD_ID:309) 2e-04

WP_095294851 Cfr family 23S rRNA (adenine(2503)-C(8))-methyltra... [36.6](file:///Users/JMBNew/Desktop/Articles%20en%20Cours/Article%20Diane/Bb_SR-11-14_output-blast_named.html#BL_ORD_ID:574) 2e-04

WP_011245929 MULTISPECIES: 23S rRNA (adenine(2503)-C(8))-methyl... [36.6](file:///Users/JMBNew/Desktop/Articles%20en%20Cours/Article%20Diane/Bb_SR-11-14_output-blast_named.html#BL_ORD_ID:547) 2e-04

CFR_BACSK RecName: Full=Ribosomal RNA large subunit methyltrans... [36.6](file:///Users/JMBNew/Desktop/Articles%20en%20Cours/Article%20Diane/Bb_SR-11-14_output-blast_named.html#BL_ORD_ID:401) 2e-04

BAD63613 florfenicol/chloramphenicol resistance protein [Bacill... [36.6](file:///Users/JMBNew/Desktop/Articles%20en%20Cours/Article%20Diane/Bb_SR-11-14_output-blast_named.html#BL_ORD_ID:313) 2e-04

ALA51754 Ribosomal RNA large subunit methyltransferase N [Bacil... [36.6](file:///Users/JMBNew/Desktop/Articles%20en%20Cours/Article%20Diane/Bb_SR-11-14_output-blast_named.html#BL_ORD_ID:207) 2e-04

WP_063608639 Cfr family 23S rRNA (adenine(2503)-C(8))-methyltra... [36.6](file:///Users/JMBNew/Desktop/Articles%20en%20Cours/Article%20Diane/Bb_SR-11-14_output-blast_named.html#BL_ORD_ID:569) 2e-04

KKI87196 chloramphenicol/florfenicol resistance protein [Bacill... [36.6](file:///Users/JMBNew/Desktop/Articles%20en%20Cours/Article%20Diane/Bb_SR-11-14_output-blast_named.html#BL_ORD_ID:480) 2e-04

WP_095327308 Cfr family 23S rRNA (adenine(2503)-C(8))-methyltra... [36.6](file:///Users/JMBNew/Desktop/Articles%20en%20Cours/Article%20Diane/Bb_SR-11-14_output-blast_named.html#BL_ORD_ID:577) 2e-04

WP_095319205 Cfr family 23S rRNA (adenine(2503)-C(8))-methyltra... [36.2](file:///Users/JMBNew/Desktop/Articles%20en%20Cours/Article%20Diane/Bb_SR-11-14_output-blast_named.html#BL_ORD_ID:576) 2e-04

AAN87489 florfenicol resistance protein, partial [Heliobacillus... [35.4](file:///Users/JMBNew/Desktop/Articles%20en%20Cours/Article%20Diane/Bb_SR-11-14_output-blast_named.html#BL_ORD_ID:10) 5e-04

WP_090913837 Cfr family 23S rRNA (adenine(2503)-C(8))-methyltra... [35.0](file:///Users/JMBNew/Desktop/Articles%20en%20Cours/Article%20Diane/Bb_SR-11-14_output-blast_named.html#BL_ORD_ID:572) 5e-04

SEM59396 23S rRNA (adenine-C8)-methyltransferase [Paenibacillus... [35.0](file:///Users/JMBNew/Desktop/Articles%20en%20Cours/Article%20Diane/Bb_SR-11-14_output-blast_named.html#BL_ORD_ID:534) 5e-04

WP_096773940 Cfr family 23S rRNA (adenine(2503)-C(8))-methyltra... [35.0](file:///Users/JMBNew/Desktop/Articles%20en%20Cours/Article%20Diane/Bb_SR-11-14_output-blast_named.html#BL_ORD_ID:578) 5e-04

WP_036660664 Cfr family 23S rRNA (adenine(2503)-C(8))-methyltra... [35.0](file:///Users/JMBNew/Desktop/Articles%20en%20Cours/Article%20Diane/Bb_SR-11-14_output-blast_named.html#BL_ORD_ID:564) 5e-04

WP_015735625 Cfr family 23S rRNA (adenine(2503)-C(8))-methyltra... [35.0](file:///Users/JMBNew/Desktop/Articles%20en%20Cours/Article%20Diane/Bb_SR-11-14_output-blast_named.html#BL_ORD_ID:558) 5e-04

ETT66883 chloramphenicol/florfenicol resistance protein [Paenib... [35.0](file:///Users/JMBNew/Desktop/Articles%20en%20Cours/Article%20Diane/Bb_SR-11-14_output-blast_named.html#BL_ORD_ID:461) 5e-04

ACX65640 radical SAM enzyme, Cfr family [Paenibacillus sp. Y412... [35.0](file:///Users/JMBNew/Desktop/Articles%20en%20Cours/Article%20Diane/Bb_SR-11-14_output-blast_named.html#BL_ORD_ID:61) 5e-04

WP_009595405 Cfr family 23S rRNA (adenine(2503)-C(8))-methyltra... [35.0](file:///Users/JMBNew/Desktop/Articles%20en%20Cours/Article%20Diane/Bb_SR-11-14_output-blast_named.html#BL_ORD_ID:546) 6e-04

EGG31816 23S rRNA m2A2503 methyltransferase [Paenibacillus sp. ... [35.0](file:///Users/JMBNew/Desktop/Articles%20en%20Cours/Article%20Diane/Bb_SR-11-14_output-blast_named.html#BL_ORD_ID:438) 6e-04

XP_009692050 florfenicol resistance protein [Theileria oriental... [34.7](file:///Users/JMBNew/Desktop/Articles%20en%20Cours/Article%20Diane/Bb_SR-11-14_output-blast_named.html#BL_ORD_ID:589) 8e-04

BAM41749 florfenicol resistance protein [Theileria orientalis s... [34.7](file:///Users/JMBNew/Desktop/Articles%20en%20Cours/Article%20Diane/Bb_SR-11-14_output-blast_named.html#BL_ORD_ID:317) 8e-04

WP_004442525 Cfr family 23S rRNA (adenine(2503)-C(8))-methyltra... [34.7](file:///Users/JMBNew/Desktop/Articles%20en%20Cours/Article%20Diane/Bb_SR-11-14_output-blast_named.html#BL_ORD_ID:545) 8e-04

EDT84436 florfenicol/chloramphenicol resistance protein [Clostr... [34.7](file:///Users/JMBNew/Desktop/Articles%20en%20Cours/Article%20Diane/Bb_SR-11-14_output-blast_named.html#BL_ORD_ID:417) 8e-04

AJE11066 23S rRNA methyltransferase [Clostridium botulinum CDC_... [34.7](file:///Users/JMBNew/Desktop/Articles%20en%20Cours/Article%20Diane/Bb_SR-11-14_output-blast_named.html#BL_ORD_ID:184) 8e-04

ACQ52150 florfenicol/chloramphenicol resistance protein [Clostr... [34.7](file:///Users/JMBNew/Desktop/Articles%20en%20Cours/Article%20Diane/Bb_SR-11-14_output-blast_named.html#BL_ORD_ID:52) 8e-04

ADO82581 23S rRNA m(2)A-2503 methyltransferase [Ilyobacter poly... [34.7](file:///Users/JMBNew/Desktop/Articles%20en%20Cours/Article%20Diane/Bb_SR-11-14_output-blast_named.html#BL_ORD_ID:68) 9e-04

WP_095305967 Cfr family 23S rRNA (adenine(2503)-C(8))-methyltra... [34.3](file:///Users/JMBNew/Desktop/Articles%20en%20Cours/Article%20Diane/Bb_SR-11-14_output-blast_named.html#BL_ORD_ID:575) 0.001

ABX42587 radical SAM enzyme, Cfr family [Lachnoclostridium phyt... [33.9](file:///Users/JMBNew/Desktop/Articles%20en%20Cours/Article%20Diane/Bb_SR-11-14_output-blast_named.html#BL_ORD_ID:40) 0.001

AAM72416 florfenicol resistance protein, putative [Chlorobium t... [33.9](file:///Users/JMBNew/Desktop/Articles%20en%20Cours/Article%20Diane/Bb_SR-11-14_output-blast_named.html#BL_ORD_ID:8) 0.001

CEP78658 23S rRNA (adenine(2503)-C(8))-methyltransferase [Deflu... [33.5](file:///Users/JMBNew/Desktop/Articles%20en%20Cours/Article%20Diane/Bb_SR-11-14_output-blast_named.html#BL_ORD_ID:400) 0.002

AHG94326 chloramphenicol florfenicol resistance protein, partia... [31.6](file:///Users/JMBNew/Desktop/Articles%20en%20Cours/Article%20Diane/Bb_SR-11-14_output-blast_named.html#BL_ORD_ID:153) 0.007

AAL53048 florfenicol resistance protein [Brucella melitensis bv... [31.2](file:///Users/JMBNew/Desktop/Articles%20en%20Cours/Article%20Diane/Bb_SR-11-14_output-blast_named.html#BL_ORD_ID:4) 0.011

ACE04355 radical SAM enzyme, Cfr family [Chlorobium phaeobacter... [30.8](file:///Users/JMBNew/Desktop/Articles%20en%20Cours/Article%20Diane/Bb_SR-11-14_output-blast_named.html#BL_ORD_ID:45) 0.014

ABS12821 radical SAM enzyme, Cfr family [Ochrobactrum anthropi ... [30.4](file:///Users/JMBNew/Desktop/Articles%20en%20Cours/Article%20Diane/Bb_SR-11-14_output-blast_named.html#BL_ORD_ID:37) 0.019

EED10097 radical SAM enzyme, Cfr family [Thermus aquaticus Y51M... [30.0](file:///Users/JMBNew/Desktop/Articles%20en%20Cours/Article%20Diane/Bb_SR-11-14_output-blast_named.html#BL_ORD_ID:419) 0.022

ACV39069 radical SAM enzyme, Cfr family [Leptotrichia buccalis ... [29.6](file:///Users/JMBNew/Desktop/Articles%20en%20Cours/Article%20Diane/Bb_SR-11-14_output-blast_named.html#BL_ORD_ID:59) 0.035

AAS81916 florfenicol resistance protein [Thermus thermophilus H... [29.3](file:///Users/JMBNew/Desktop/Articles%20en%20Cours/Article%20Diane/Bb_SR-11-14_output-blast_named.html#BL_ORD_ID:16) 0.048

AEE96185 23S rRNA m(2)A-2503 methyltransferase [Mahella austral... [28.5](file:///Users/JMBNew/Desktop/Articles%20en%20Cours/Article%20Diane/Bb_SR-11-14_output-blast_named.html#BL_ORD_ID:71) 0.077

AIL54054 23S methyltransferase, partial [Clostridioides difficile] [28.1](file:///Users/JMBNew/Desktop/Articles%20en%20Cours/Article%20Diane/Bb_SR-11-14_output-blast_named.html#BL_ORD_ID:172) 0.083

BAD68852 florfenicol resistance protein-like [Oryza sativa Japo... [27.7](file:///Users/JMBNew/Desktop/Articles%20en%20Cours/Article%20Diane/Bb_SR-11-14_output-blast_named.html#BL_ORD_ID:314) 0.11

AAT87417 Florfenicol resistance protein [Streptococcus pyogenes... [27.3](file:///Users/JMBNew/Desktop/Articles%20en%20Cours/Article%20Diane/Bb_SR-11-14_output-blast_named.html#BL_ORD_ID:18) 0.16

ABO50235 23S rRNA m(2)A-2503 methyltransferase [Desulfotomaculu... [26.6](file:///Users/JMBNew/Desktop/Articles%20en%20Cours/Article%20Diane/Bb_SR-11-14_output-blast_named.html#BL_ORD_ID:31) 0.36

AAO35792 florfenicol resistance protein [Clostridium tetani E88] [25.8](file:///Users/JMBNew/Desktop/Articles%20en%20Cours/Article%20Diane/Bb_SR-11-14_output-blast_named.html#BL_ORD_ID:11) 0.53

> KXA23970 23S rRNA methyltransferase [Fusobacterium nucleatum]

Length=357

Score = 39.7 bits (91), Expect = 2e-05, Method: Compositional matrix adjust.

Identities = 17/31 (55%), Positives = 19/31 (61%), Gaps = 0/31 (0%)

Frame = +2

Query 449 SPRKERAEPWCISSQAGCVVNCRFCSTGQPG 541

S KE C+SSQ GC V C FC+TGQ G

Sbjct 104 SKNKEIRNTLCVSSQVGCPVKCSFCATGQSG 134

> EJU08814 florfenicol resistance protein [Fusobacterium hwasookii

ChDC F128]

Length=358

Score = 39.7 bits (91), Expect = 2e-05, Method: Compositional matrix adjust.

Identities = 17/31 (55%), Positives = 19/31 (61%), Gaps = 0/31 (0%)

Frame = +2

Query 449 SPRKERAEPWCISSQAGCVVNCRFCSTGQPG 541

S KE C+SSQ GC V C FC+TGQ G

Sbjct 104 SKNKEIRNTLCVSSQVGCPVKCSFCATGQSG 134

> NP_603423 florfenicol resistance protein [Fusobacterium nucleatum

subsp. nucleatum ATCC 25586]

Length=358

Score = 39.7 bits (91), Expect = 2e-05, Method: Compositional matrix adjust.

Identities = 17/31 (55%), Positives = 19/31 (61%), Gaps = 0/31 (0%)

Frame = +2

Query 449 SPRKERAEPWCISSQAGCVVNCRFCSTGQPG 541

S KE C+SSQ GC V C FC+TGQ G

Sbjct 104 SKNKEIRNTLCVSSQVGCPVKCSFCATGQSG 134

> AAL94722 Florfenicol resistance protein [Fusobacterium nucleatum

subsp. nucleatum ATCC 25586]

Length=358

Score = 39.7 bits (91), Expect = 2e-05, Method: Compositional matrix adjust.

Identities = 17/31 (55%), Positives = 19/31 (61%), Gaps = 0/31 (0%)

Frame = +2

Query 449 SPRKERAEPWCISSQAGCVVNCRFCSTGQPG 541

S KE C+SSQ GC V C FC+TGQ G

Sbjct 104 SKNKEIRNTLCVSSQVGCPVKCSFCATGQSG 134

> EFE87464 23S rRNA m2A2503 methyltransferase [Fusobacterium periodonticum

ATCC 33693]

Length=358

Score = 38.9 bits (89), Expect = 3e-05, Method: Compositional matrix adjust.

Identities = 17/31 (55%), Positives = 19/31 (61%), Gaps = 0/31 (0%)

Frame = +2

Query 449 SPRKERAEPWCISSQAGCVVNCRFCSTGQPG 541

S KE C+SSQ GC V C FC+TGQ G

Sbjct 104 SKNKEIRNTLCVSSQVGCPVKCSFCATGQGG 134

> EHI75907 23S rRNA m2A2503 methyltransferase [Fusobacterium sp.

oral taxon 370 str. F0437]

Length=358

Score = 38.5 bits (88), Expect = 4e-05, Method: Compositional matrix adjust.

Identities = 16/31 (52%), Positives = 19/31 (61%), Gaps = 0/31 (0%)

Frame = +2

Query 449 SPRKERAEPWCISSQAGCVVNCRFCSTGQPG 541

S KE C+SSQ GC + C FC+TGQ G

Sbjct 104 SKNKEIRNTLCVSSQVGCPIKCSFCATGQGG 134

> EJG09268 florfenicol resistance protein [Fusobacterium nucleatum

subsp. fusiforme ATCC 51190]

Length=358

Score = 38.5 bits (88), Expect = 5e-05, Method: Compositional matrix adjust.

Identities = 16/31 (52%), Positives = 19/31 (61%), Gaps = 0/31 (0%)

Frame = +2

Query 449 SPRKERAEPWCISSQAGCVVNCRFCSTGQPG 541

S +E C+SSQ GC V C FC+TGQ G

Sbjct 104 SKNREIRNTLCVSSQVGCPVKCSFCATGQSG 134

> EMP16199 florfenicol resistance protein [Fusobacterium nucleatum

CC53]

Length=358

Score = 38.5 bits (88), Expect = 5e-05, Method: Compositional matrix adjust.

Identities = 16/31 (52%), Positives = 19/31 (61%), Gaps = 0/31 (0%)

Frame = +2

Query 449 SPRKERAEPWCISSQAGCVVNCRFCSTGQPG 541

S +E C+SSQ GC V C FC+TGQ G

Sbjct 104 SKNREIRNTLCVSSQVGCPVKCSFCATGQSG 134

> ACF11531 radical SAM enzyme, Cfr family [Chlorobaculum parvum

NCIB 8327]

Length=374

Score = 38.5 bits (88), Expect = 5e-05, Method: Compositional matrix adjust.

Identities = 18/38 (47%), Positives = 22/38 (58%), Gaps = 1/38 (3%)

Frame = +2

Query 452 PRKERAEPWCISSQAGCVVNCRFCSTGQPGLQPHTCRP 565

P +ER CISSQ GC + C FC+TGQ G + P

Sbjct 119 PSEERMTA-CISSQVGCALRCTFCATGQMGFKRDLVAP 155

> YP_001388569 chloramphenicol/florfenicol resistance protein [Clostridium

botulinum A str. Hall]

Length=344

Score = 38.5 bits (88), Expect = 5e-05, Method: Compositional matrix adjust.

Identities = 15/26 (58%), Positives = 18/26 (69%), Gaps = 0/26 (0%)

Frame = +2

Query 470 EPWCISSQAGCVVNCRFCSTGQPGLQ 547

E +CISSQ GC CRFC+TG G +

Sbjct 101 ESFCISSQCGCSFGCRFCATGSAGFK 126

> YP_001255356 chloramphenicol/florfenicol resistance protein [Clostridium

botulinum A str. ATCC 3502]

Length=344

Score = 38.5 bits (88), Expect = 5e-05, Method: Compositional matrix adjust.

Identities = 15/26 (58%), Positives = 18/26 (69%), Gaps = 0/26 (0%)

Frame = +2

Query 470 EPWCISSQAGCVVNCRFCSTGQPGLQ 547

E +CISSQ GC CRFC+TG G +

Sbjct 101 ESFCISSQCGCSFGCRFCATGSAGFK 126

> WP_011987126 23S rRNA (adenine(2503)-C(8))-methyltransferase

Cfr [Clostridium botulinum]

Length=344

Score = 38.5 bits (88), Expect = 5e-05, Method: Compositional matrix adjust.

Identities = 15/26 (58%), Positives = 18/26 (69%), Gaps = 0/26 (0%)

Frame = +2

Query 470 EPWCISSQAGCVVNCRFCSTGQPGLQ 547

E +CISSQ GC CRFC+TG G +

Sbjct 101 ESFCISSQCGCSFGCRFCATGSAGFK 126

> OSA98255 23S rRNA (adenine(2503)-C(8))-methyltransferase Cfr

[Clostridium botulinum]

Length=344

Score = 38.5 bits (88), Expect = 5e-05, Method: Compositional matrix adjust.

Identities = 15/26 (58%), Positives = 18/26 (69%), Gaps = 0/26 (0%)

Frame = +2

Query 470 EPWCISSQAGCVVNCRFCSTGQPGLQ 547

E +CISSQ GC CRFC+TG G +

Sbjct 101 ESFCISSQCGCSFGCRFCATGSAGFK 126

> KON11466 chloramphenicol/florfenicol resistance protein [Clostridium

botulinum]

Length=344

Score = 38.5 bits (88), Expect = 5e-05, Method: Compositional matrix adjust.

Identities = 15/26 (58%), Positives = 18/26 (69%), Gaps = 0/26 (0%)

Frame = +2

Query 470 EPWCISSQAGCVVNCRFCSTGQPGLQ 547

E +CISSQ GC CRFC+TG G +

Sbjct 101 ESFCISSQCGCSFGCRFCATGSAGFK 126

> EPS49069 chloramphenicol/florfenicol resistance protein [Clostridium

botulinum CFSAN002369]

Length=344

Score = 38.5 bits (88), Expect = 5e-05, Method: Compositional matrix adjust.

Identities = 15/26 (58%), Positives = 18/26 (69%), Gaps = 0/26 (0%)

Frame = +2

Query 470 EPWCISSQAGCVVNCRFCSTGQPGLQ 547

E +CISSQ GC CRFC+TG G +

Sbjct 101 ESFCISSQCGCSFGCRFCATGSAGFK 126

> CFR_CLOBH RecName: Full=Ribosomal RNA large subunit methyltransferase

Cfr; AltName: Full=23S rRNA (adenine(2503)-C(8))-methyltransferase;

AltName: Full=23S rRNA m8A2503 methyltransferase

Length=344

Score = 38.5 bits (88), Expect = 5e-05, Method: Compositional matrix adjust.

Identities = 15/26 (58%), Positives = 18/26 (69%), Gaps = 0/26 (0%)

Frame = +2

Query 470 EPWCISSQAGCVVNCRFCSTGQPGLQ 547

E +CISSQ GC CRFC+TG G +

Sbjct 101 ESFCISSQCGCSFGCRFCATGSAGFK 126

> CFR_CLOB1 RecName: Full=Ribosomal RNA large subunit methyltransferase

Cfr; AltName: Full=23S rRNA (adenine(2503)-C(8))-methyltransferase;

AltName: Full=23S rRNA m8A2503 methyltransferase

Length=344

Score = 38.5 bits (88), Expect = 5e-05, Method: Compositional matrix adjust.

Identities = 15/26 (58%), Positives = 18/26 (69%), Gaps = 0/26 (0%)

Frame = +2

Query 470 EPWCISSQAGCVVNCRFCSTGQPGLQ 547

E +CISSQ GC CRFC+TG G +

Sbjct 101 ESFCISSQCGCSFGCRFCATGSAGFK 126

> CAL84423 putative florfenicol resistance protein [Clostridium

botulinum A str. ATCC 3502]

Length=344

Score = 38.5 bits (88), Expect = 5e-05, Method: Compositional matrix adjust.

Identities = 15/26 (58%), Positives = 18/26 (69%), Gaps = 0/26 (0%)

Frame = +2

Query 470 EPWCISSQAGCVVNCRFCSTGQPGLQ 547

E +CISSQ GC CRFC+TG G +

Sbjct 101 ESFCISSQCGCSFGCRFCATGSAGFK 126

> ABS37379 florfenicol/chloramphenicol resistance protein [Clostridium

botulinum A str. Hall]

Length=344

Score = 38.5 bits (88), Expect = 5e-05, Method: Compositional matrix adjust.

Identities = 15/26 (58%), Positives = 18/26 (69%), Gaps = 0/26 (0%)

Frame = +2

Query 470 EPWCISSQAGCVVNCRFCSTGQPGLQ 547

E +CISSQ GC CRFC+TG G +

Sbjct 101 ESFCISSQCGCSFGCRFCATGSAGFK 126

> ABS35021 florfenicol/chloramphenicol resistance protein [Clostridium

botulinum A str. ATCC 19397]

Length=344

Score = 38.5 bits (88), Expect = 5e-05, Method: Compositional matrix adjust.

Identities = 15/26 (58%), Positives = 18/26 (69%), Gaps = 0/26 (0%)

Frame = +2

Query 470 EPWCISSQAGCVVNCRFCSTGQPGLQ 547

E +CISSQ GC CRFC+TG G +

Sbjct 101 ESFCISSQCGCSFGCRFCATGSAGFK 126

> EYD70257 florfenicol resistance protein [Fusobacterium necrophorum

subsp. funduliforme B35]

Length=349

Score = 38.1 bits (87), Expect = 6e-05, Method: Compositional matrix adjust.

Identities = 17/44 (39%), Positives = 26/44 (59%), Gaps = 1/44 (2%)

Frame = +2

Query 458 KERAEPWCISSQAGCVVNCRFCSTGQPGLQPHTCRPAKHVAQLW 589

+++ CISSQ GC V C FC+TGQ G R ++ + Q++

Sbjct 100 RDQRNTLCISSQVGCPVKCTFCATGQDGF-VRNLRVSEILNQVY 142

> KXA13869 23S rRNA methyltransferase [Fusobacterium equinum]

Length=349

Score = 38.1 bits (87), Expect = 6e-05, Method: Compositional matrix adjust.

Identities = 17/44 (39%), Positives = 26/44 (59%), Gaps = 1/44 (2%)

Frame = +2

Query 458 KERAEPWCISSQAGCVVNCRFCSTGQPGLQPHTCRPAKHVAQLW 589

+++ CISSQ GC V C FC+TGQ G R ++ + Q++

Sbjct 100 RDQRNTLCISSQVGCPVKCSFCATGQDGF-VRNLRVSEILNQVY 142

> WP_061310144 Cfr family 23S rRNA (adenine(2503)-C(8))-methyltransferase

[Clostridium botulinum]

Length=344

Score = 37.7 bits (86), Expect = 8e-05, Method: Compositional matrix adjust.

Identities = 15/26 (58%), Positives = 18/26 (69%), Gaps = 0/26 (0%)

Frame = +2

Query 470 EPWCISSQAGCVVNCRFCSTGQPGLQ 547

E +CISSQ GC CRFC+TG G +

Sbjct 101 ESFCISSQCGCGFGCRFCATGSAGFK 126

> WP_045540636 Cfr family 23S rRNA (adenine(2503)-C(8))-methyltransferase

[Clostridium botulinum]

Length=344

Score = 37.7 bits (86), Expect = 8e-05, Method: Compositional matrix adjust.

Identities = 15/26 (58%), Positives = 18/26 (69%), Gaps = 0/26 (0%)

Frame = +2

Query 470 EPWCISSQAGCVVNCRFCSTGQPGLQ 547

E +CISSQ GC CRFC+TG G +

Sbjct 101 ESFCISSQCGCGFGCRFCATGSAGFK 126

> KEI76823 chloramphenicol/florfenicol resistance protein [Clostridium

botulinum B2 128]

Length=344

Score = 37.7 bits (86), Expect = 8e-05, Method: Compositional matrix adjust.

Identities = 15/26 (58%), Positives = 18/26 (69%), Gaps = 0/26 (0%)

Frame = +2

Query 470 EPWCISSQAGCVVNCRFCSTGQPGLQ 547

E +CISSQ GC CRFC+TG G +

Sbjct 101 ESFCISSQCGCGFGCRFCATGSAGFK 126

> BAQ14476 putative chloramphenicol/florfenicol resistance protein

[Clostridium botulinum]

Length=344

Score = 37.7 bits (86), Expect = 8e-05, Method: Compositional matrix adjust.

Identities = 15/26 (58%), Positives = 18/26 (69%), Gaps = 0/26 (0%)

Frame = +2

Query 470 EPWCISSQAGCVVNCRFCSTGQPGLQ 547

E +CISSQ GC CRFC+TG G +

Sbjct 101 ESFCISSQCGCGFGCRFCATGSAGFK 126

> WP_041350656 Cfr family 23S rRNA (adenine(2503)-C(8))-methyltransferase

[Clostridium botulinum]

Length=344

Score = 37.7 bits (86), Expect = 8e-05, Method: Compositional matrix adjust.

Identities = 15/26 (58%), Positives = 18/26 (69%), Gaps = 0/26 (0%)

Frame = +2

Query 470 EPWCISSQAGCVVNCRFCSTGQPGLQ 547

E +CISSQ GC CRFC+TG G +

Sbjct 101 ESFCISSQCGCGFGCRFCATGSAGFK 126

> WP_015957497 23S rRNA (adenine(2503)-C(8))-methyltransferase

Cfr [Clostridium botulinum]

Length=344

Score = 37.7 bits (86), Expect = 8e-05, Method: Compositional matrix adjust.

Identities = 15/26 (58%), Positives = 18/26 (69%), Gaps = 0/26 (0%)

Frame = +2

Query 470 EPWCISSQAGCVVNCRFCSTGQPGLQ 547

E +CISSQ GC CRFC+TG G +

Sbjct 101 ESFCISSQCGCGFGCRFCATGSAGFK 126

> WP_012341861 23S rRNA (adenine(2503)-C(8))-methyltransferase

Cfr [Clostridium botulinum]

Length=344

Score = 37.7 bits (86), Expect = 8e-05, Method: Compositional matrix adjust.

Identities = 15/26 (58%), Positives = 18/26 (69%), Gaps = 0/26 (0%)

Frame = +2

Query 470 EPWCISSQAGCVVNCRFCSTGQPGLQ 547

E +CISSQ GC CRFC+TG G +

Sbjct 101 ESFCISSQCGCGFGCRFCATGSAGFK 126

> KIN79793 chloramphenicol/florfenicol resistance protein [Clostridium

botulinum]

Length=344

Score = 37.7 bits (86), Expect = 8e-05, Method: Compositional matrix adjust.

Identities = 15/26 (58%), Positives = 18/26 (69%), Gaps = 0/26 (0%)

Frame = +2

Query 470 EPWCISSQAGCVVNCRFCSTGQPGLQ 547

E +CISSQ GC CRFC+TG G +

Sbjct 101 ESFCISSQCGCGFGCRFCATGSAGFK 126

> CFR_CLOBM RecName: Full=Ribosomal RNA large subunit methyltransferase

Cfr; AltName: Full=23S rRNA (adenine(2503)-C(8))-methyltransferase;

AltName: Full=23S rRNA m8A2503 methyltransferase

Length=344

Score = 37.7 bits (86), Expect = 8e-05, Method: Compositional matrix adjust.

Identities = 15/26 (58%), Positives = 18/26 (69%), Gaps = 0/26 (0%)

Frame = +2

Query 470 EPWCISSQAGCVVNCRFCSTGQPGLQ 547

E +CISSQ GC CRFC+TG G +

Sbjct 101 ESFCISSQCGCGFGCRFCATGSAGFK 126

> CFR_CLOBK RecName: Full=Ribosomal RNA large subunit methyltransferase

Cfr; AltName: Full=23S rRNA (adenine(2503)-C(8))-methyltransferase;

AltName: Full=23S rRNA m8A2503 methyltransferase

Length=344

Score = 37.7 bits (86), Expect = 8e-05, Method: Compositional matrix adjust.

Identities = 15/26 (58%), Positives = 18/26 (69%), Gaps = 0/26 (0%)

Frame = +2

Query 470 EPWCISSQAGCVVNCRFCSTGQPGLQ 547

E +CISSQ GC CRFC+TG G +

Sbjct 101 ESFCISSQCGCGFGCRFCATGSAGFK 126

> ACA53662 florfenicol/chloramphenicol resistance protein [Clostridium

botulinum A3 str. Loch Maree]

Length=344

Score = 37.7 bits (86), Expect = 8e-05, Method: Compositional matrix adjust.

Identities = 15/26 (58%), Positives = 18/26 (69%), Gaps = 0/26 (0%)

Frame = +2

Query 470 EPWCISSQAGCVVNCRFCSTGQPGLQ 547

E +CISSQ GC CRFC+TG G +

Sbjct 101 ESFCISSQCGCGFGCRFCATGSAGFK 126

> ACA43818 florfenicol/chloramphenicol resistance protein [Clostridium

botulinum B1 str. Okra]

Length=344

Score = 37.7 bits (86), Expect = 8e-05, Method: Compositional matrix adjust.

Identities = 15/26 (58%), Positives = 18/26 (69%), Gaps = 0/26 (0%)

Frame = +2

Query 470 EPWCISSQAGCVVNCRFCSTGQPGLQ 547

E +CISSQ GC CRFC+TG G +

Sbjct 101 ESFCISSQCGCGFGCRFCATGSAGFK 126

> WP_076178522 Cfr family 23S rRNA (adenine(2503)-C(8))-methyltransferase

[Clostridium botulinum]

Length=344

Score = 37.7 bits (86), Expect = 8e-05, Method: Compositional matrix adjust.

Identities = 15/26 (58%), Positives = 18/26 (69%), Gaps = 0/26 (0%)

Frame = +2

Query 470 EPWCISSQAGCVVNCRFCSTGQPGLQ 547

E +CISSQ GC CRFC+TG G +

Sbjct 101 ESFCISSQCGCGFGCRFCATGSAGFK 126

> WP_024932864 Cfr family 23S rRNA (adenine(2503)-C(8))-methyltransferase

[Clostridium botulinum]

Length=344

Score = 37.7 bits (86), Expect = 8e-05, Method: Compositional matrix adjust.

Identities = 15/26 (58%), Positives = 18/26 (69%), Gaps = 0/26 (0%)

Frame = +2

Query 470 EPWCISSQAGCVVNCRFCSTGQPGLQ 547

E +CISSQ GC CRFC+TG G +

Sbjct 101 ESFCISSQCGCGFGCRFCATGSAGFK 126

> WP_014521578 Cfr family 23S rRNA (adenine(2503)-C(8))-methyltransferase

[Clostridium botulinum]

Length=344

Score = 37.7 bits (86), Expect = 8e-05, Method: Compositional matrix adjust.

Identities = 15/26 (58%), Positives = 18/26 (69%), Gaps = 0/26 (0%)

Frame = +2

Query 470 EPWCISSQAGCVVNCRFCSTGQPGLQ 547

E +CISSQ GC CRFC+TG G +

Sbjct 101 ESFCISSQCGCGFGCRFCATGSAGFK 126

> OSB14185 23S rRNA (adenine(2503)-C(8))-methyltransferase Cfr

[Clostridium botulinum]

Length=344

Score = 37.7 bits (86), Expect = 8e-05, Method: Compositional matrix adjust.

Identities = 15/26 (58%), Positives = 18/26 (69%), Gaps = 0/26 (0%)

Frame = +2

Query 470 EPWCISSQAGCVVNCRFCSTGQPGLQ 547

E +CISSQ GC CRFC+TG G +

Sbjct 101 ESFCISSQCGCGFGCRFCATGSAGFK 126

> OSA80008 23S rRNA (adenine(2503)-C(8))-methyltransferase Cfr

[Clostridium botulinum]

Length=344

Score = 37.7 bits (86), Expect = 8e-05, Method: Compositional matrix adjust.

Identities = 15/26 (58%), Positives = 18/26 (69%), Gaps = 0/26 (0%)

Frame = +2

Query 470 EPWCISSQAGCVVNCRFCSTGQPGLQ 547

E +CISSQ GC CRFC+TG G +

Sbjct 101 ESFCISSQCGCGFGCRFCATGSAGFK 126

> OSA71862 23S rRNA (adenine(2503)-C(8))-methyltransferase Cfr

[Clostridium botulinum]

Length=344

Score = 37.7 bits (86), Expect = 8e-05, Method: Compositional matrix adjust.

Identities = 15/26 (58%), Positives = 18/26 (69%), Gaps = 0/26 (0%)

Frame = +2

Query 470 EPWCISSQAGCVVNCRFCSTGQPGLQ 547

E +CISSQ GC CRFC+TG G +

Sbjct 101 ESFCISSQCGCGFGCRFCATGSAGFK 126

> OPD29848 23S rRNA (adenine(2503)-C8)-methyltransferase [Clostridium

botulinum]

Length=344

Score = 37.7 bits (86), Expect = 8e-05, Method: Compositional matrix adjust.

Identities = 15/26 (58%), Positives = 18/26 (69%), Gaps = 0/26 (0%)

Frame = +2

Query 470 EPWCISSQAGCVVNCRFCSTGQPGLQ 547

E +CISSQ GC CRFC+TG G +

Sbjct 101 ESFCISSQCGCGFGCRFCATGSAGFK 126

> KOR51488 23S rRNA (adenine(2503)-C8)-methyltransferase [Clostridium

botulinum]

Length=344

Score = 37.7 bits (86), Expect = 8e-05, Method: Compositional matrix adjust.

Identities = 15/26 (58%), Positives = 18/26 (69%), Gaps = 0/26 (0%)

Frame = +2

Query 470 EPWCISSQAGCVVNCRFCSTGQPGLQ 547

E +CISSQ GC CRFC+TG G +

Sbjct 101 ESFCISSQCGCGFGCRFCATGSAGFK 126

> KEI96147 chloramphenicol/florfenicol resistance protein [Clostridium

botulinum F 357]

Length=344

Score = 37.7 bits (86), Expect = 8e-05, Method: Compositional matrix adjust.

Identities = 15/26 (58%), Positives = 18/26 (69%), Gaps = 0/26 (0%)

Frame = +2

Query 470 EPWCISSQAGCVVNCRFCSTGQPGLQ 547

E +CISSQ GC CRFC+TG G +

Sbjct 101 ESFCISSQCGCGFGCRFCATGSAGFK 126

> KEI90501 chloramphenicol/florfenicol resistance protein [Clostridium

botulinum B2 433]

Length=344

Score = 37.7 bits (86), Expect = 8e-05, Method: Compositional matrix adjust.

Identities = 15/26 (58%), Positives = 18/26 (69%), Gaps = 0/26 (0%)

Frame = +2

Query 470 EPWCISSQAGCVVNCRFCSTGQPGLQ 547

E +CISSQ GC CRFC+TG G +

Sbjct 101 ESFCISSQCGCGFGCRFCATGSAGFK 126

> KEI79595 chloramphenicol/florfenicol resistance protein [Clostridium

botulinum A2 117]

Length=344

Score = 37.7 bits (86), Expect = 8e-05, Method: Compositional matrix adjust.

Identities = 15/26 (58%), Positives = 18/26 (69%), Gaps = 0/26 (0%)

Frame = +2

Query 470 EPWCISSQAGCVVNCRFCSTGQPGLQ 547

E +CISSQ GC CRFC+TG G +

Sbjct 101 ESFCISSQCGCGFGCRFCATGSAGFK 126

> CBZ04747 putative florfenicol resistance protein [Clostridium

botulinum H04402 065]

Length=344

Score = 37.7 bits (86), Expect = 8e-05, Method: Compositional matrix adjust.

Identities = 15/26 (58%), Positives = 18/26 (69%), Gaps = 0/26 (0%)

Frame = +2

Query 470 EPWCISSQAGCVVNCRFCSTGQPGLQ 547

E +CISSQ GC CRFC+TG G +

Sbjct 101 ESFCISSQCGCGFGCRFCATGSAGFK 126

> APH18681 23S rRNA (adenine(2503)-C(8))-methyltransferase Cfr

[Clostridium botulinum]

Length=344

Score = 37.7 bits (86), Expect = 8e-05, Method: Compositional matrix adjust.

Identities = 15/26 (58%), Positives = 18/26 (69%), Gaps = 0/26 (0%)

Frame = +2

Query 470 EPWCISSQAGCVVNCRFCSTGQPGLQ 547

E +CISSQ GC CRFC+TG G +

Sbjct 101 ESFCISSQCGCGFGCRFCATGSAGFK 126

> YP_009023638 chloramphenicol-florfenicol resistance protein,

CFR (plasmid) [Enterococcus faecalis]

Length=349

Score = 37.7 bits (86), Expect = 8e-05, Method: Compositional matrix adjust.

Identities = 15/28 (54%), Positives = 20/28 (71%), Gaps = 0/28 (0%)

Frame = +2

Query 470 EPWCISSQAGCVVNCRFCSTGQPGLQPH 553

E +CISSQ GC C+FC+TG GL+ +

Sbjct 102 ESFCISSQCGCNFGCKFCATGNIGLKKN 129

> YP_003896025 chloramphenicol-florfenicol resistance protein,

CFR (plasmid) [Enterococcus faecalis]

Length=349

Score = 37.7 bits (86), Expect = 8e-05, Method: Compositional matrix adjust.

Identities = 15/28 (54%), Positives = 20/28 (71%), Gaps = 0/28 (0%)

Frame = +2

Query 470 EPWCISSQAGCVVNCRFCSTGQPGLQPH 553

E +CISSQ GC C+FC+TG GL+ +

Sbjct 102 ESFCISSQCGCNFGCKFCATGNIGLKKN 129

> WP_013330753 Cfr family 23S rRNA (adenine(2503)-C(8))-methyltransferase

[Enterococcus faecalis]

Length=349

Score = 37.7 bits (86), Expect = 8e-05, Method: Compositional matrix adjust.

Identities = 15/28 (54%), Positives = 20/28 (71%), Gaps = 0/28 (0%)

Frame = +2

Query 470 EPWCISSQAGCVVNCRFCSTGQPGLQPH 553

E +CISSQ GC C+FC+TG GL+ +

Sbjct 102 ESFCISSQCGCNFGCKFCATGNIGLKKN 129

> ALI92641 Cfr (plasmid) [Enterococcus faecalis]

Length=349

Score = 37.7 bits (86), Expect = 8e-05, Method: Compositional matrix adjust.

Identities = 15/28 (54%), Positives = 20/28 (71%), Gaps = 0/28 (0%)

Frame = +2

Query 470 EPWCISSQAGCVVNCRFCSTGQPGLQPH 553

E +CISSQ GC C+FC+TG GL+ +

Sbjct 102 ESFCISSQCGCNFGCKFCATGNIGLKKN 129

> AIA99220 chloramphenicol-florfenicol resistance protein, CFR

(plasmid) [Enterococcus casseliflavus]

Length=349

Score = 37.7 bits (86), Expect = 8e-05, Method: Compositional matrix adjust.

Identities = 15/28 (54%), Positives = 20/28 (71%), Gaps = 0/28 (0%)

Frame = +2

Query 470 EPWCISSQAGCVVNCRFCSTGQPGLQPH 553

E +CISSQ GC C+FC+TG GL+ +

Sbjct 102 ESFCISSQCGCNFGCKFCATGNIGLKKN 129

> AHN52249 chloramphenicol-florfenicol resistance protein, CFR

(plasmid) [Enterococcus faecalis]

Length=349

Score = 37.7 bits (86), Expect = 8e-05, Method: Compositional matrix adjust.

Identities = 15/28 (54%), Positives = 20/28 (71%), Gaps = 0/28 (0%)

Frame = +2

Query 470 EPWCISSQAGCVVNCRFCSTGQPGLQPH 553

E +CISSQ GC C+FC+TG GL+ +

Sbjct 102 ESFCISSQCGCNFGCKFCATGNIGLKKN 129

> ADN34770 chloramphenicol-florfenicol resistance protein, CFR

(plasmid) [Enterococcus faecalis]

Length=349

Score = 37.7 bits (86), Expect = 8e-05, Method: Compositional matrix adjust.

Identities = 15/28 (54%), Positives = 20/28 (71%), Gaps = 0/28 (0%)

Frame = +2

Query 470 EPWCISSQAGCVVNCRFCSTGQPGLQPH 553

E +CISSQ GC C+FC+TG GL+ +

Sbjct 102 ESFCISSQCGCNFGCKFCATGNIGLKKN 129

> ARX61578 23S ribosomal RNA methyltransferase (plasmid) [Escherichia

coli]

Length=349

Score = 37.7 bits (86), Expect = 8e-05, Method: Compositional matrix adjust.

Identities = 15/28 (54%), Positives = 20/28 (71%), Gaps = 0/28 (0%)

Frame = +2

Query 470 EPWCISSQAGCVVNCRFCSTGQPGLQPH 553

E +CISSQ GC C+FC+TG GL+ +

Sbjct 102 ESFCISSQCGCNFGCKFCATGDIGLKKN 129

> ARX61545 florfenicol/chloramphenicol resistance (plasmid) [Escherichia

coli]

Length=349

Score = 37.7 bits (86), Expect = 8e-05, Method: Compositional matrix adjust.

Identities = 15/28 (54%), Positives = 20/28 (71%), Gaps = 0/28 (0%)

Frame = +2

Query 470 EPWCISSQAGCVVNCRFCSTGQPGLQPH 553

E +CISSQ GC C+FC+TG GL+ +

Sbjct 102 ESFCISSQCGCNFGCKFCATGDIGLKKN 129

> ALJ52404 Cfr (plasmid) [Escherichia coli]

Length=349

Score = 37.7 bits (86), Expect = 8e-05, Method: Compositional matrix adjust.

Identities = 15/28 (54%), Positives = 20/28 (71%), Gaps = 0/28 (0%)

Frame = +2

Query 470 EPWCISSQAGCVVNCRFCSTGQPGLQPH 553

E +CISSQ GC C+FC+TG GL+ +

Sbjct 102 ESFCISSQCGCNFGCKFCATGDIGLKKN 129

> AIY22804 florfenicol/chloramphenicol resistance protein (plasmid)

[Escherichia coli]

Length=349

Score = 37.7 bits (86), Expect = 8e-05, Method: Compositional matrix adjust.

Identities = 15/28 (54%), Positives = 20/28 (71%), Gaps = 0/28 (0%)

Frame = +2

Query 470 EPWCISSQAGCVVNCRFCSTGQPGLQPH 553

E +CISSQ GC C+FC+TG GL+ +

Sbjct 102 ESFCISSQCGCNFGCKFCATGDIGLKKN 129

> WP_030037108 Cfr family 23S rRNA (adenine(2503)-C(8))-methyltransferase

[Clostridium botulinum]

Length=344

Score = 37.7 bits (86), Expect = 8e-05, Method: Compositional matrix adjust.

Identities = 15/26 (58%), Positives = 18/26 (69%), Gaps = 0/26 (0%)

Frame = +2

Query 470 EPWCISSQAGCVVNCRFCSTGQPGLQ 547

E +CISSQ GC CRFC+TG G +

Sbjct 101 ESFCISSQCGCGFGCRFCATGSAGFK 126

> WP_003405508 23S rRNA (adenine(2503)-C(8))-methyltransferase

Cfr [Clostridium botulinum]

Length=344

Score = 37.7 bits (86), Expect = 8e-05, Method: Compositional matrix adjust.

Identities = 15/26 (58%), Positives = 18/26 (69%), Gaps = 0/26 (0%)

Frame = +2

Query 470 EPWCISSQAGCVVNCRFCSTGQPGLQ 547

E +CISSQ GC CRFC+TG G +

Sbjct 101 ESFCISSQCGCGFGCRFCATGSAGFK 126

> GAE03833 chloramphenicol/florfenicol resistance protein [Clostridium

botulinum B str. Osaka05]

Length=344

Score = 37.7 bits (86), Expect = 8e-05, Method: Compositional matrix adjust.

Identities = 15/26 (58%), Positives = 18/26 (69%), Gaps = 0/26 (0%)

Frame = +2

Query 470 EPWCISSQAGCVVNCRFCSTGQPGLQ 547

E +CISSQ GC CRFC+TG G +

Sbjct 101 ESFCISSQCGCGFGCRFCATGSAGFK 126

> EKX78206 chloramphenicol/florfenicol resistance protein [Clostridium

botulinum CFSAN001628]

Length=344

Score = 37.7 bits (86), Expect = 8e-05, Method: Compositional matrix adjust.

Identities = 15/26 (58%), Positives = 18/26 (69%), Gaps = 0/26 (0%)

Frame = +2

Query 470 EPWCISSQAGCVVNCRFCSTGQPGLQ 547

E +CISSQ GC CRFC+TG G +

Sbjct 101 ESFCISSQCGCGFGCRFCATGSAGFK 126

> cfr_-_17035810_translation cfr: florfenicol/chloramphenicol resistance

protein

Length=349

Score = 37.7 bits (86), Expect = 8e-05, Method: Compositional matrix adjust.

Identities = 15/28 (54%), Positives = 20/28 (71%), Gaps = 0/28 (0%)

Frame = +2

Query 470 EPWCISSQAGCVVNCRFCSTGQPGLQPH 553

E +CISSQ GC C+FC+TG GL+ +

Sbjct 102 ESFCISSQCGCNFGCKFCATGDIGLKKN 129

> YP_008575034 florfenicol/chloramphenicol resistance protein (plasmid)

[Escherichia coli]

Length=349

Score = 37.7 bits (86), Expect = 8e-05, Method: Compositional matrix adjust.

Identities = 15/28 (54%), Positives = 20/28 (71%), Gaps = 0/28 (0%)

Frame = +2

Query 470 EPWCISSQAGCVVNCRFCSTGQPGLQPH 553

E +CISSQ GC C+FC+TG GL+ +

Sbjct 102 ESFCISSQCGCNFGCKFCATGDIGLKKN 129

> YP_007988851 23S rRNA methylase (plasmid) [Staphylococcus aureus]

Length=349

Score = 37.7 bits (86), Expect = 8e-05, Method: Compositional matrix adjust.

Identities = 15/28 (54%), Positives = 20/28 (71%), Gaps = 0/28 (0%)

Frame = +2

Query 470 EPWCISSQAGCVVNCRFCSTGQPGLQPH 553

E +CISSQ GC C+FC+TG GL+ +

Sbjct 102 ESFCISSQCGCNFGCKFCATGDIGLKKN 129

> YP_007878372 23S rRNA methylase (plasmid) [Staphylococcus aureus]

Length=349

Score = 37.7 bits (86), Expect = 8e-05, Method: Compositional matrix adjust.

Identities = 15/28 (54%), Positives = 20/28 (71%), Gaps = 0/28 (0%)

Frame = +2

Query 470 EPWCISSQAGCVVNCRFCSTGQPGLQPH 553

E +CISSQ GC C+FC+TG GL+ +

Sbjct 102 ESFCISSQCGCNFGCKFCATGDIGLKKN 129

> YP_004888091 23S rRNA methylase (plasmid) [Staphylococcus arlettae]

Length=349

Score = 37.7 bits (86), Expect = 8e-05, Method: Compositional matrix adjust.

Identities = 15/28 (54%), Positives = 20/28 (71%), Gaps = 0/28 (0%)

Frame = +2

Query 470 EPWCISSQAGCVVNCRFCSTGQPGLQPH 553

E +CISSQ GC C+FC+TG GL+ +

Sbjct 102 ESFCISSQCGCNFGCKFCATGDIGLKKN 129

> YP_003927883 rRNA methylase (plasmid) [Bacillus sp. BS-02]

Length=349

Score = 37.7 bits (86), Expect = 8e-05, Method: Compositional matrix adjust.

Identities = 15/28 (54%), Positives = 20/28 (71%), Gaps = 0/28 (0%)

Frame = +2

Query 470 EPWCISSQAGCVVNCRFCSTGQPGLQPH 553

E +CISSQ GC C+FC+TG GL+ +

Sbjct 102 ESFCISSQCGCNFGCKFCATGDIGLKKN 129

> WP_032491462 23S rRNA (adenine(2503)-C(8))-methyltransferase

Cfr [Staphylococcus aureus]

Length=349

Score = 37.7 bits (86), Expect = 8e-05, Method: Compositional matrix adjust.

Identities = 15/28 (54%), Positives = 20/28 (71%), Gaps = 0/28 (0%)

Frame = +2

Query 470 EPWCISSQAGCVVNCRFCSTGQPGLQPH 553

E +CISSQ GC C+FC+TG GL+ +

Sbjct 102 ESFCISSQCGCNFGCKFCATGDIGLKKN 129

> WP_001010505 MULTISPECIES: 23S rRNA (adenine(2503)-C(8))-methyltransferase

Cfr [Bacteria]

Length=349

Score = 37.7 bits (86), Expect = 8e-05, Method: Compositional matrix adjust.

Identities = 15/28 (54%), Positives = 20/28 (71%), Gaps = 0/28 (0%)

Frame = +2

Query 470 EPWCISSQAGCVVNCRFCSTGQPGLQPH 553

E +CISSQ GC C+FC+TG GL+ +

Sbjct 102 ESFCISSQCGCNFGCKFCATGDIGLKKN 129

> NP_899167 florfenicol/chloramphenicol resistance protein (plasmid)

[Staphylococcus sciuri]

Length=349

Score = 37.7 bits (86), Expect = 8e-05, Method: Compositional matrix adjust.

Identities = 15/28 (54%), Positives = 20/28 (71%), Gaps = 0/28 (0%)

Frame = +2

Query 470 EPWCISSQAGCVVNCRFCSTGQPGLQPH 553

E +CISSQ GC C+FC+TG GL+ +

Sbjct 102 ESFCISSQCGCNFGCKFCATGDIGLKKN 129

> EXP53712 ribosomal RNA large subunit methyltransferase Cfr [Staphylococcus

aureus W12583]

Length=349

Score = 37.7 bits (86), Expect = 8e-05, Method: Compositional matrix adjust.

Identities = 15/28 (54%), Positives = 20/28 (71%), Gaps = 0/28 (0%)

Frame = +2

Query 470 EPWCISSQAGCVVNCRFCSTGQPGLQPH 553

E +CISSQ GC C+FC+TG GL+ +

Sbjct 102 ESFCISSQCGCNFGCKFCATGDIGLKKN 129

> EXO68354 ribosomal RNA large subunit methyltransferase Cfr [Staphylococcus

aureus W12586]

Length=349

Score = 37.7 bits (86), Expect = 8e-05, Method: Compositional matrix adjust.

Identities = 15/28 (54%), Positives = 20/28 (71%), Gaps = 0/28 (0%)

Frame = +2

Query 470 EPWCISSQAGCVVNCRFCSTGQPGLQPH 553

E +CISSQ GC C+FC+TG GL+ +

Sbjct 102 ESFCISSQCGCNFGCKFCATGDIGLKKN 129

> CFR_STAWA RecName: Full=Ribosomal RNA large subunit methyltransferase

Cfr; AltName: Full=23S rRNA (adenine(2503)-C(8))-methyltransferase;

AltName: Full=23S rRNA m8A2503 methyltransferase

Length=349

Score = 37.7 bits (86), Expect = 8e-05, Method: Compositional matrix adjust.

Identities = 15/28 (54%), Positives = 20/28 (71%), Gaps = 0/28 (0%)

Frame = +2

Query 470 EPWCISSQAGCVVNCRFCSTGQPGLQPH 553

E +CISSQ GC C+FC+TG GL+ +

Sbjct 102 ESFCISSQCGCNFGCKFCATGDIGLKKN 129

> CFR_STASC RecName: Full=Ribosomal RNA large subunit methyltransferase

Cfr; AltName: Full=23S rRNA (adenine(2503)-C(8))-methyltransferase;

AltName: Full=23S rRNA m8A2503 methyltransferase

Length=349

Score = 37.7 bits (86), Expect = 8e-05, Method: Compositional matrix adjust.

Identities = 15/28 (54%), Positives = 20/28 (71%), Gaps = 0/28 (0%)

Frame = +2

Query 470 EPWCISSQAGCVVNCRFCSTGQPGLQPH 553

E +CISSQ GC C+FC+TG GL+ +

Sbjct 102 ESFCISSQCGCNFGCKFCATGDIGLKKN 129

> CFR_STAAU RecName: Full=Ribosomal RNA large subunit methyltransferase

Cfr; AltName: Full=23S rRNA (adenine(2503)-C(8))-methyltransferase;

AltName: Full=23S rRNA m8A2503 methyltransferase

Length=349

Score = 37.7 bits (86), Expect = 8e-05, Method: Compositional matrix adjust.

Identities = 15/28 (54%), Positives = 20/28 (71%), Gaps = 0/28 (0%)

Frame = +2

Query 470 EPWCISSQAGCVVNCRFCSTGQPGLQPH 553

E +CISSQ GC C+FC+TG GL+ +

Sbjct 102 ESFCISSQCGCNFGCKFCATGDIGLKKN 129

> CBW44219 rRNA methylase (plasmid) [Staphylococcus aureus]

Length=349

Score = 37.7 bits (86), Expect = 8e-05, Method: Compositional matrix adjust.

Identities = 15/28 (54%), Positives = 20/28 (71%), Gaps = 0/28 (0%)

Frame = +2

Query 470 EPWCISSQAGCVVNCRFCSTGQPGLQPH 553

E +CISSQ GC C+FC+TG GL+ +

Sbjct 102 ESFCISSQCGCNFGCKFCATGDIGLKKN 129

> CBN88266 rRNA methylase [Staphylococcus aureus]

Length=349

Score = 37.7 bits (86), Expect = 8e-05, Method: Compositional matrix adjust.

Identities = 15/28 (54%), Positives = 20/28 (71%), Gaps = 0/28 (0%)

Frame = +2

Query 470 EPWCISSQAGCVVNCRFCSTGQPGLQPH 553

E +CISSQ GC C+FC+TG GL+ +

Sbjct 102 ESFCISSQCGCNFGCKFCATGDIGLKKN 129

> CAL64019 rRNA methylase (plasmid) [Staphylococcus warneri]

Length=349

Score = 37.7 bits (86), Expect = 8e-05, Method: Compositional matrix adjust.

Identities = 15/28 (54%), Positives = 20/28 (71%), Gaps = 0/28 (0%)

Frame = +2

Query 470 EPWCISSQAGCVVNCRFCSTGQPGLQPH 553

E +CISSQ GC C+FC+TG GL+ +

Sbjct 102 ESFCISSQCGCNFGCKFCATGDIGLKKN 129

> CAJ30491 23S rRNA methylase (plasmid) [Staphylococcus aureus]

Length=349

Score = 37.7 bits (86), Expect = 8e-05, Method: Compositional matrix adjust.

Identities = 15/28 (54%), Positives = 20/28 (71%), Gaps = 0/28 (0%)

Frame = +2

Query 470 EPWCISSQAGCVVNCRFCSTGQPGLQPH 553

E +CISSQ GC C+FC+TG GL+ +

Sbjct 102 ESFCISSQCGCNFGCKFCATGDIGLKKN 129

> CAI56203 methyltransferase (plasmid) [Staphylococcus aureus]

Length=349

Score = 37.7 bits (86), Expect = 8e-05, Method: Compositional matrix adjust.

Identities = 15/28 (54%), Positives = 20/28 (71%), Gaps = 0/28 (0%)

Frame = +2

Query 470 EPWCISSQAGCVVNCRFCSTGQPGLQPH 553

E +CISSQ GC C+FC+TG GL+ +

Sbjct 102 ESFCISSQCGCNFGCKFCATGDIGLKKN 129

> CAE18142 florfenicol/chloramphenicol resistance protein (plasmid)

[Staphylococcus sciuri]

Length=349

Score = 37.7 bits (86), Expect = 8e-05, Method: Compositional matrix adjust.

Identities = 15/28 (54%), Positives = 20/28 (71%), Gaps = 0/28 (0%)

Frame = +2

Query 470 EPWCISSQAGCVVNCRFCSTGQPGLQPH 553

E +CISSQ GC C+FC+TG GL+ +

Sbjct 102 ESFCISSQCGCNFGCKFCATGDIGLKKN 129

> CAC04525 florfenicol resistance protein (plasmid) [Staphylococcus

sciuri]

Length=349

Score = 37.7 bits (86), Expect = 8e-05, Method: Compositional matrix adjust.

Identities = 15/28 (54%), Positives = 20/28 (71%), Gaps = 0/28 (0%)

Frame = +2

Query 470 EPWCISSQAGCVVNCRFCSTGQPGLQPH 553

E +CISSQ GC C+FC+TG GL+ +

Sbjct 102 ESFCISSQCGCNFGCKFCATGDIGLKKN 129

> ARQ19363 chloramphenicol-florfenicol resistance protein (plasmid)

[Staphylococcus epidermidis]

Length=349

Score = 37.7 bits (86), Expect = 8e-05, Method: Compositional matrix adjust.

Identities = 15/28 (54%), Positives = 20/28 (71%), Gaps = 0/28 (0%)

Frame = +2

Query 470 EPWCISSQAGCVVNCRFCSTGQPGLQPH 553

E +CISSQ GC C+FC+TG GL+ +

Sbjct 102 ESFCISSQCGCNFGCKFCATGDIGLKKN 129

> ARA73644 23S ribosomal RNA methyltransferase Cfr (plasmid) [Staphylococcus

epidermidis]

Length=349

Score = 37.7 bits (86), Expect = 8e-05, Method: Compositional matrix adjust.

Identities = 15/28 (54%), Positives = 20/28 (71%), Gaps = 0/28 (0%)

Frame = +2

Query 470 EPWCISSQAGCVVNCRFCSTGQPGLQPH 553

E +CISSQ GC C+FC+TG GL+ +

Sbjct 102 ESFCISSQCGCNFGCKFCATGDIGLKKN 129

> AQZ36678 23S rRNA methylase (plasmid) [Staphylococcus sciuri]

Length=349

Score = 37.7 bits (86), Expect = 8e-05, Method: Compositional matrix adjust.

Identities = 15/28 (54%), Positives = 20/28 (71%), Gaps = 0/28 (0%)

Frame = +2

Query 470 EPWCISSQAGCVVNCRFCSTGQPGLQPH 553

E +CISSQ GC C+FC+TG GL+ +

Sbjct 102 ESFCISSQCGCNFGCKFCATGDIGLKKN 129

> AQW34743 23S rRNA methylase [Staphylococcus sciuri]

Length=349

Score = 37.7 bits (86), Expect = 8e-05, Method: Compositional matrix adjust.

Identities = 15/28 (54%), Positives = 20/28 (71%), Gaps = 0/28 (0%)

Frame = +2

Query 470 EPWCISSQAGCVVNCRFCSTGQPGLQPH 553

E +CISSQ GC C+FC+TG GL+ +

Sbjct 102 ESFCISSQCGCNFGCKFCATGDIGLKKN 129

> AQW34729 23S rRNA methylase [Staphylococcus sciuri]

Length=349

Score = 37.7 bits (86), Expect = 8e-05, Method: Compositional matrix adjust.

Identities = 15/28 (54%), Positives = 20/28 (71%), Gaps = 0/28 (0%)

Frame = +2

Query 470 EPWCISSQAGCVVNCRFCSTGQPGLQPH 553

E +CISSQ GC C+FC+TG GL+ +

Sbjct 102 ESFCISSQCGCNFGCKFCATGDIGLKKN 129

> AQW34711 23S rRNA methylase [Staphylococcus sciuri]

Length=349

Score = 37.7 bits (86), Expect = 8e-05, Method: Compositional matrix adjust.

Identities = 15/28 (54%), Positives = 20/28 (71%), Gaps = 0/28 (0%)

Frame = +2

Query 470 EPWCISSQAGCVVNCRFCSTGQPGLQPH 553

E +CISSQ GC C+FC+TG GL+ +

Sbjct 102 ESFCISSQCGCNFGCKFCATGDIGLKKN 129

> AQW34676 23S rRNA methylase (plasmid) [Staphylococcus sciuri]

Length=349

Score = 37.7 bits (86), Expect = 8e-05, Method: Compositional matrix adjust.

Identities = 15/28 (54%), Positives = 20/28 (71%), Gaps = 0/28 (0%)

Frame = +2

Query 470 EPWCISSQAGCVVNCRFCSTGQPGLQPH 553

E +CISSQ GC C+FC+TG GL+ +

Sbjct 102 ESFCISSQCGCNFGCKFCATGDIGLKKN 129

> AQW34623 23S rRNA methylase (plasmid) [Staphylococcus sciuri]

Length=349

Score = 37.7 bits (86), Expect = 8e-05, Method: Compositional matrix adjust.

Identities = 15/28 (54%), Positives = 20/28 (71%), Gaps = 0/28 (0%)

Frame = +2

Query 470 EPWCISSQAGCVVNCRFCSTGQPGLQPH 553

E +CISSQ GC C+FC+TG GL+ +

Sbjct 102 ESFCISSQCGCNFGCKFCATGDIGLKKN 129

> AQW34576 23S rRNA methylase [Staphylococcus sciuri]

Length=349

Score = 37.7 bits (86), Expect = 8e-05, Method: Compositional matrix adjust.

Identities = 15/28 (54%), Positives = 20/28 (71%), Gaps = 0/28 (0%)

Frame = +2

Query 470 EPWCISSQAGCVVNCRFCSTGQPGLQPH 553

E +CISSQ GC C+FC+TG GL+ +

Sbjct 102 ESFCISSQCGCNFGCKFCATGDIGLKKN 129

> AMN16502 Cfr (plasmid) [Staphylococcus aureus subsp. aureus]

Length=349

Score = 37.7 bits (86), Expect = 8e-05, Method: Compositional matrix adjust.

Identities = 15/28 (54%), Positives = 20/28 (71%), Gaps = 0/28 (0%)

Frame = +2

Query 470 EPWCISSQAGCVVNCRFCSTGQPGLQPH 553

E +CISSQ GC C+FC+TG GL+ +

Sbjct 102 ESFCISSQCGCNFGCKFCATGDIGLKKN 129

> AMN16461 chloramphenicol-florfenicol resistance protein CFR (plasmid)

[Staphylococcus aureus subsp. aureus]

Length=349

Score = 37.7 bits (86), Expect = 8e-05, Method: Compositional matrix adjust.

Identities = 15/28 (54%), Positives = 20/28 (71%), Gaps = 0/28 (0%)

Frame = +2

Query 470 EPWCISSQAGCVVNCRFCSTGQPGLQPH 553

E +CISSQ GC C+FC+TG GL+ +

Sbjct 102 ESFCISSQCGCNFGCKFCATGDIGLKKN 129

> ALI92801 23S rRNA methylase (plasmid) [Staphylococcus sciuri]

Length=349

Score = 37.7 bits (86), Expect = 8e-05, Method: Compositional matrix adjust.

Identities = 15/28 (54%), Positives = 20/28 (71%), Gaps = 0/28 (0%)

Frame = +2

Query 470 EPWCISSQAGCVVNCRFCSTGQPGLQPH 553

E +CISSQ GC C+FC+TG GL+ +

Sbjct 102 ESFCISSQCGCNFGCKFCATGDIGLKKN 129

> ALF95990 rRNA methyltransferase (plasmid) [Staphylococcus aureus]

Length=349

Score = 37.7 bits (86), Expect = 8e-05, Method: Compositional matrix adjust.

Identities = 15/28 (54%), Positives = 20/28 (71%), Gaps = 0/28 (0%)

Frame = +2

Query 470 EPWCISSQAGCVVNCRFCSTGQPGLQPH 553

E +CISSQ GC C+FC+TG GL+ +

Sbjct 102 ESFCISSQCGCNFGCKFCATGDIGLKKN 129

> ALF95987 rRNA methyltransferase (plasmid) [Staphylococcus epidermidis]

Length=349

Score = 37.7 bits (86), Expect = 8e-05, Method: Compositional matrix adjust.

Identities = 15/28 (54%), Positives = 20/28 (71%), Gaps = 0/28 (0%)

Frame = +2

Query 470 EPWCISSQAGCVVNCRFCSTGQPGLQPH 553

E +CISSQ GC C+FC+TG GL+ +

Sbjct 102 ESFCISSQCGCNFGCKFCATGDIGLKKN 129

> ALE30190 23S rRNA methylase (plasmid) [Staphylococcus simulans]

Length=349

Score = 37.7 bits (86), Expect = 8e-05, Method: Compositional matrix adjust.

Identities = 15/28 (54%), Positives = 20/28 (71%), Gaps = 0/28 (0%)

Frame = +2

Query 470 EPWCISSQAGCVVNCRFCSTGQPGLQPH 553

E +CISSQ GC C+FC+TG GL+ +

Sbjct 102 ESFCISSQCGCNFGCKFCATGDIGLKKN 129

> AKL80218 23S rRNA methylase (plasmid) [Staphylococcus xylosus]

Length=349

Score = 37.7 bits (86), Expect = 8e-05, Method: Compositional matrix adjust.

Identities = 15/28 (54%), Positives = 20/28 (71%), Gaps = 0/28 (0%)

Frame = +2

Query 470 EPWCISSQAGCVVNCRFCSTGQPGLQPH 553

E +CISSQ GC C+FC+TG GL+ +

Sbjct 102 ESFCISSQCGCNFGCKFCATGDIGLKKN 129

> AKE50930 23S rRNA methylase [Staphylococcus aureus]

Length=349

Score = 37.7 bits (86), Expect = 8e-05, Method: Compositional matrix adjust.

Identities = 15/28 (54%), Positives = 20/28 (71%), Gaps = 0/28 (0%)

Frame = +2

Query 470 EPWCISSQAGCVVNCRFCSTGQPGLQPH 553

E +CISSQ GC C+FC+TG GL+ +

Sbjct 102 ESFCISSQCGCNFGCKFCATGDIGLKKN 129

> AJW29170 rRNA methyltransferase (plasmid) [Staphylococcus epidermidis]

Length=349

Score = 37.7 bits (86), Expect = 8e-05, Method: Compositional matrix adjust.

Identities = 15/28 (54%), Positives = 20/28 (71%), Gaps = 0/28 (0%)

Frame = +2

Query 470 EPWCISSQAGCVVNCRFCSTGQPGLQPH 553

E +CISSQ GC C+FC+TG GL+ +

Sbjct 102 ESFCISSQCGCNFGCKFCATGDIGLKKN 129

> AJW29120 rRNA methyltransferase (plasmid) [Staphylococcus epidermidis]

Length=349

Score = 37.7 bits (86), Expect = 8e-05, Method: Compositional matrix adjust.

Identities = 15/28 (54%), Positives = 20/28 (71%), Gaps = 0/28 (0%)

Frame = +2

Query 470 EPWCISSQAGCVVNCRFCSTGQPGLQPH 553

E +CISSQ GC C+FC+TG GL+ +

Sbjct 102 ESFCISSQCGCNFGCKFCATGDIGLKKN 129

> AJQ17306 Cfr (plasmid) [Escherichia coli]

Length=349

Score = 37.7 bits (86), Expect = 8e-05, Method: Compositional matrix adjust.

Identities = 15/28 (54%), Positives = 20/28 (71%), Gaps = 0/28 (0%)

Frame = +2

Query 470 EPWCISSQAGCVVNCRFCSTGQPGLQPH 553

E +CISSQ GC C+FC+TG GL+ +

Sbjct 102 ESFCISSQCGCNFGCKFCATGDIGLKKN 129

> AJM87289 23S rRNA methylase (plasmid) [Staphylococcus aureus]

Length=349

Score = 37.7 bits (86), Expect = 8e-05, Method: Compositional matrix adjust.

Identities = 15/28 (54%), Positives = 20/28 (71%), Gaps = 0/28 (0%)

Frame = +2

Query 470 EPWCISSQAGCVVNCRFCSTGQPGLQPH 553

E +CISSQ GC C+FC+TG GL+ +

Sbjct 102 ESFCISSQCGCNFGCKFCATGDIGLKKN 129

> AJF83635 florfenicol/chloramphenicol resistance protein (plasmid)

[Escherichia coli]

Length=349

Score = 37.7 bits (86), Expect = 8e-05, Method: Compositional matrix adjust.

Identities = 15/28 (54%), Positives = 20/28 (71%), Gaps = 0/28 (0%)

Frame = +2

Query 470 EPWCISSQAGCVVNCRFCSTGQPGLQPH 553

E +CISSQ GC C+FC+TG GL+ +

Sbjct 102 ESFCISSQCGCNFGCKFCATGDIGLKKN 129

> AJF83598 florfenicol/chloramphenicol resistance protein (plasmid)

[Escherichia coli]

Length=349

Score = 37.7 bits (86), Expect = 8e-05, Method: Compositional matrix adjust.

Identities = 15/28 (54%), Positives = 20/28 (71%), Gaps = 0/28 (0%)

Frame = +2

Query 470 EPWCISSQAGCVVNCRFCSTGQPGLQPH 553

E +CISSQ GC C+FC+TG GL+ +

Sbjct 102 ESFCISSQCGCNFGCKFCATGDIGLKKN 129

> AIT41446 Cfr (plasmid) [Staphylococcus aureus]

Length=349

Score = 37.7 bits (86), Expect = 8e-05, Method: Compositional matrix adjust.

Identities = 15/28 (54%), Positives = 20/28 (71%), Gaps = 0/28 (0%)

Frame = +2

Query 470 EPWCISSQAGCVVNCRFCSTGQPGLQPH 553

E +CISSQ GC C+FC+TG GL+ +

Sbjct 102 ESFCISSQCGCNFGCKFCATGDIGLKKN 129

> AIT38258 23S rRNA methyltransferase [Staphylococcus cohnii]

Length=349

Score = 37.7 bits (86), Expect = 8e-05, Method: Compositional matrix adjust.

Identities = 15/28 (54%), Positives = 20/28 (71%), Gaps = 0/28 (0%)

Frame = +2

Query 470 EPWCISSQAGCVVNCRFCSTGQPGLQPH 553

E +CISSQ GC C+FC+TG GL+ +

Sbjct 102 ESFCISSQCGCNFGCKFCATGDIGLKKN 129

> AIL01330 Cfr [Staphylococcus aureus]

Length=349

Score = 37.7 bits (86), Expect = 8e-05, Method: Compositional matrix adjust.

Identities = 15/28 (54%), Positives = 20/28 (71%), Gaps = 0/28 (0%)

Frame = +2

Query 470 EPWCISSQAGCVVNCRFCSTGQPGLQPH 553

E +CISSQ GC C+FC+TG GL+ +

Sbjct 102 ESFCISSQCGCNFGCKFCATGDIGLKKN 129

> AIL01327 Cfr [Staphylococcus aureus]

Length=349

Score = 37.7 bits (86), Expect = 8e-05, Method: Compositional matrix adjust.

Identities = 15/28 (54%), Positives = 20/28 (71%), Gaps = 0/28 (0%)

Frame = +2

Query 470 EPWCISSQAGCVVNCRFCSTGQPGLQPH 553

E +CISSQ GC C+FC+TG GL+ +

Sbjct 102 ESFCISSQCGCNFGCKFCATGDIGLKKN 129

> AIL01324 Cfr [Staphylococcus aureus]

Length=349

Score = 37.7 bits (86), Expect = 8e-05, Method: Compositional matrix adjust.

Identities = 15/28 (54%), Positives = 20/28 (71%), Gaps = 0/28 (0%)

Frame = +2

Query 470 EPWCISSQAGCVVNCRFCSTGQPGLQPH 553

E +CISSQ GC C+FC+TG GL+ +

Sbjct 102 ESFCISSQCGCNFGCKFCATGDIGLKKN 129

> AIG88478 rRNA methylase Cfr (plasmid) [Escherichia coli]

Length=349

Score = 37.7 bits (86), Expect = 8e-05, Method: Compositional matrix adjust.

Identities = 15/28 (54%), Positives = 20/28 (71%), Gaps = 0/28 (0%)

Frame = +2

Query 470 EPWCISSQAGCVVNCRFCSTGQPGLQPH 553

E +CISSQ GC C+FC+TG GL+ +

Sbjct 102 ESFCISSQCGCNFGCKFCATGDIGLKKN 129

> AIG88475 rRNA methylase Cfr (plasmid) [Escherichia coli]

Length=349

Score = 37.7 bits (86), Expect = 8e-05, Method: Compositional matrix adjust.

Identities = 15/28 (54%), Positives = 20/28 (71%), Gaps = 0/28 (0%)

Frame = +2

Query 470 EPWCISSQAGCVVNCRFCSTGQPGLQPH 553

E +CISSQ GC C+FC+TG GL+ +

Sbjct 102 ESFCISSQCGCNFGCKFCATGDIGLKKN 129

> AIG88472 rRNA methylase Cfr (plasmid) [Escherichia coli]

Length=349

Score = 37.7 bits (86), Expect = 8e-05, Method: Compositional matrix adjust.

Identities = 15/28 (54%), Positives = 20/28 (71%), Gaps = 0/28 (0%)

Frame = +2

Query 470 EPWCISSQAGCVVNCRFCSTGQPGLQPH 553

E +CISSQ GC C+FC+TG GL+ +

Sbjct 102 ESFCISSQCGCNFGCKFCATGDIGLKKN 129

> AIG88468 rRNA methylase Cfr (plasmid) [Escherichia coli]

Length=349

Score = 37.7 bits (86), Expect = 8e-05, Method: Compositional matrix adjust.

Identities = 15/28 (54%), Positives = 20/28 (71%), Gaps = 0/28 (0%)

Frame = +2

Query 470 EPWCISSQAGCVVNCRFCSTGQPGLQPH 553

E +CISSQ GC C+FC+TG GL+ +

Sbjct 102 ESFCISSQCGCNFGCKFCATGDIGLKKN 129

> AIA99243 chloramphenicol-florfenicol resistance protein, CFR

[Enterococcus casseliflavus]

Length=349

Score = 37.7 bits (86), Expect = 8e-05, Method: Compositional matrix adjust.

Identities = 15/28 (54%), Positives = 20/28 (71%), Gaps = 0/28 (0%)

Frame = +2

Query 470 EPWCISSQAGCVVNCRFCSTGQPGLQPH 553

E +CISSQ GC C+FC+TG GL+ +

Sbjct 102 ESFCISSQCGCNFGCKFCATGDIGLKKN 129

> AIA99231 chloramphenicol-florfenicol resistance protein, CFR

[Enterococcus casseliflavus]

Length=349

Score = 37.7 bits (86), Expect = 8e-05, Method: Compositional matrix adjust.

Identities = 15/28 (54%), Positives = 20/28 (71%), Gaps = 0/28 (0%)

Frame = +2

Query 470 EPWCISSQAGCVVNCRFCSTGQPGLQPH 553

E +CISSQ GC C+FC+TG GL+ +

Sbjct 102 ESFCISSQCGCNFGCKFCATGDIGLKKN 129

> AHL28472 23S rRNA methylase (plasmid) [Staphylococcus simulans]

Length=349

Score = 37.7 bits (86), Expect = 8e-05, Method: Compositional matrix adjust.

Identities = 15/28 (54%), Positives = 20/28 (71%), Gaps = 0/28 (0%)

Frame = +2

Query 470 EPWCISSQAGCVVNCRFCSTGQPGLQPH 553

E +CISSQ GC C+FC+TG GL+ +

Sbjct 102 ESFCISSQCGCNFGCKFCATGDIGLKKN 129

> AHL28467 23S rRNA methylase (plasmid) [Staphylococcus simulans]

Length=349

Score = 37.7 bits (86), Expect = 8e-05, Method: Compositional matrix adjust.

Identities = 15/28 (54%), Positives = 20/28 (71%), Gaps = 0/28 (0%)

Frame = +2

Query 470 EPWCISSQAGCVVNCRFCSTGQPGLQPH 553

E +CISSQ GC C+FC+TG GL+ +

Sbjct 102 ESFCISSQCGCNFGCKFCATGDIGLKKN 129

> AHL28458 23S rRNA methylase (plasmid) [Staphylococcus simulans]

Length=349

Score = 37.7 bits (86), Expect = 8e-05, Method: Compositional matrix adjust.

Identities = 15/28 (54%), Positives = 20/28 (71%), Gaps = 0/28 (0%)

Frame = +2

Query 470 EPWCISSQAGCVVNCRFCSTGQPGLQPH 553

E +CISSQ GC C+FC+TG GL+ +

Sbjct 102 ESFCISSQCGCNFGCKFCATGDIGLKKN 129

> AHJ80329 23S rRNA methylase (plasmid) [Staphylococcus aureus]

Length=349

Score = 37.7 bits (86), Expect = 8e-05, Method: Compositional matrix adjust.

Identities = 15/28 (54%), Positives = 20/28 (71%), Gaps = 0/28 (0%)

Frame = +2

Query 470 EPWCISSQAGCVVNCRFCSTGQPGLQPH 553

E +CISSQ GC C+FC+TG GL+ +

Sbjct 102 ESFCISSQCGCNFGCKFCATGDIGLKKN 129

> AHI16978 23S rRNA methylase (plasmid) [Staphylococcus equorum]

Length=349

Score = 37.7 bits (86), Expect = 8e-05, Method: Compositional matrix adjust.

Identities = 15/28 (54%), Positives = 20/28 (71%), Gaps = 0/28 (0%)

Frame = +2

Query 470 EPWCISSQAGCVVNCRFCSTGQPGLQPH 553

E +CISSQ GC C+FC+TG GL+ +

Sbjct 102 ESFCISSQCGCNFGCKFCATGDIGLKKN 129

> AHI16972 23S rRNA methylase (plasmid) [Staphylococcus sciuri]

Length=349

Score = 37.7 bits (86), Expect = 8e-05, Method: Compositional matrix adjust.

Identities = 15/28 (54%), Positives = 20/28 (71%), Gaps = 0/28 (0%)

Frame = +2

Query 470 EPWCISSQAGCVVNCRFCSTGQPGLQPH 553

E +CISSQ GC C+FC+TG GL+ +

Sbjct 102 ESFCISSQCGCNFGCKFCATGDIGLKKN 129

> AHB87388 23S rRNA methylase (plasmid) [Staphylococcus rostri]

Length=349

Score = 37.7 bits (86), Expect = 8e-05, Method: Compositional matrix adjust.

Identities = 15/28 (54%), Positives = 20/28 (71%), Gaps = 0/28 (0%)

Frame = +2

Query 470 EPWCISSQAGCVVNCRFCSTGQPGLQPH 553

E +CISSQ GC C+FC+TG GL+ +

Sbjct 102 ESFCISSQCGCNFGCKFCATGDIGLKKN 129

> AGZ63429 23S rRNA methylase [Staphylococcus lentus]

Length=349

Score = 37.7 bits (86), Expect = 8e-05, Method: Compositional matrix adjust.

Identities = 15/28 (54%), Positives = 20/28 (71%), Gaps = 0/28 (0%)

Frame = +2

Query 470 EPWCISSQAGCVVNCRFCSTGQPGLQPH 553

E +CISSQ GC C+FC+TG GL+ +

Sbjct 102 ESFCISSQCGCNFGCKFCATGDIGLKKN 129

> AGZ63423 florfenicol/chloramphenicol resistance protein [Staphylococcus

lentus]

Length=349

Score = 37.7 bits (86), Expect = 8e-05, Method: Compositional matrix adjust.

Identities = 15/28 (54%), Positives = 20/28 (71%), Gaps = 0/28 (0%)

Frame = +2

Query 470 EPWCISSQAGCVVNCRFCSTGQPGLQPH 553

E +CISSQ GC C+FC+TG GL+ +

Sbjct 102 ESFCISSQCGCNFGCKFCATGDIGLKKN 129

> AGW01079 florfenicol/chloramphenicol resistance protein (plasmid)

[Escherichia coli]

Length=349

Score = 37.7 bits (86), Expect = 8e-05, Method: Compositional matrix adjust.

Identities = 15/28 (54%), Positives = 20/28 (71%), Gaps = 0/28 (0%)

Frame = +2

Query 470 EPWCISSQAGCVVNCRFCSTGQPGLQPH 553

E +CISSQ GC C+FC+TG GL+ +

Sbjct 102 ESFCISSQCGCNFGCKFCATGDIGLKKN 129

> AGR88944 florfenicol/chloramphenicol resistance protein (plasmid)

[Staphylococcus sciuri]

Length=349

Score = 37.7 bits (86), Expect = 8e-05, Method: Compositional matrix adjust.

Identities = 15/28 (54%), Positives = 20/28 (71%), Gaps = 0/28 (0%)

Frame = +2

Query 470 EPWCISSQAGCVVNCRFCSTGQPGLQPH 553

E +CISSQ GC C+FC+TG GL+ +

Sbjct 102 ESFCISSQCGCNFGCKFCATGDIGLKKN 129

> AGR88927 23S rRNA methylase (plasmid) [Staphylococcus lentus]

Length=349

Score = 37.7 bits (86), Expect = 8e-05, Method: Compositional matrix adjust.

Identities = 15/28 (54%), Positives = 20/28 (71%), Gaps = 0/28 (0%)

Frame = +2

Query 470 EPWCISSQAGCVVNCRFCSTGQPGLQPH 553

E +CISSQ GC C+FC+TG GL+ +

Sbjct 102 ESFCISSQCGCNFGCKFCATGDIGLKKN 129

> AGR88915 florfenicol/chloramphenicol resistance protein [Staphylococcus

lentus]

Length=349

Score = 37.7 bits (86), Expect = 8e-05, Method: Compositional matrix adjust.

Identities = 15/28 (54%), Positives = 20/28 (71%), Gaps = 0/28 (0%)

Frame = +2

Query 470 EPWCISSQAGCVVNCRFCSTGQPGLQPH 553

E +CISSQ GC C+FC+TG GL+ +

Sbjct 102 ESFCISSQCGCNFGCKFCATGDIGLKKN 129

> AGQ80867 23S rRNA methylase (plasmid) [Staphylococcus cohnii]

Length=349

Score = 37.7 bits (86), Expect = 8e-05, Method: Compositional matrix adjust.

Identities = 15/28 (54%), Positives = 20/28 (71%), Gaps = 0/28 (0%)

Frame = +2

Query 470 EPWCISSQAGCVVNCRFCSTGQPGLQPH 553

E +CISSQ GC C+FC+TG GL+ +

Sbjct 102 ESFCISSQCGCNFGCKFCATGDIGLKKN 129

> AGQ80838 23S rRNA methyltransferase (plasmid) [Staphylococcus

cohnii]

Length=349

Score = 37.7 bits (86), Expect = 8e-05, Method: Compositional matrix adjust.

Identities = 15/28 (54%), Positives = 20/28 (71%), Gaps = 0/28 (0%)

Frame = +2

Query 470 EPWCISSQAGCVVNCRFCSTGQPGLQPH 553

E +CISSQ GC C+FC+TG GL+ +

Sbjct 102 ESFCISSQCGCNFGCKFCATGDIGLKKN 129

> AGO02198 florfenicol/chloramphenicol resistance protein (plasmid)

[Streptococcus suis]

Length=349

Score = 37.7 bits (86), Expect = 8e-05, Method: Compositional matrix adjust.

Identities = 15/28 (54%), Positives = 20/28 (71%), Gaps = 0/28 (0%)

Frame = +2

Query 470 EPWCISSQAGCVVNCRFCSTGQPGLQPH 553

E +CISSQ GC C+FC+TG GL+ +

Sbjct 102 ESFCISSQCGCNFGCKFCATGDIGLKKN 129

> AGL42330 23S rRNA methylase (plasmid) [Staphylococcus aureus]

Length=349

Score = 37.7 bits (86), Expect = 8e-05, Method: Compositional matrix adjust.

Identities = 15/28 (54%), Positives = 20/28 (71%), Gaps = 0/28 (0%)

Frame = +2

Query 470 EPWCISSQAGCVVNCRFCSTGQPGLQPH 553

E +CISSQ GC C+FC+TG GL+ +

Sbjct 102 ESFCISSQCGCNFGCKFCATGDIGLKKN 129

> AGJ70604 23S rRNA methylase (plasmid) [Staphylococcus epidermidis]

Length=349

Score = 37.7 bits (86), Expect = 8e-05, Method: Compositional matrix adjust.

Identities = 15/28 (54%), Positives = 20/28 (71%), Gaps = 0/28 (0%)

Frame = +2

Query 470 EPWCISSQAGCVVNCRFCSTGQPGLQPH 553

E +CISSQ GC C+FC+TG GL+ +

Sbjct 102 ESFCISSQCGCNFGCKFCATGDIGLKKN 129

> AGH72126 chloramphenicol-florfenicol resistance protein (plasmid)

[Streptococcus suis]

Length=349

Score = 37.7 bits (86), Expect = 8e-05, Method: Compositional matrix adjust.

Identities = 15/28 (54%), Positives = 20/28 (71%), Gaps = 0/28 (0%)

Frame = +2

Query 470 EPWCISSQAGCVVNCRFCSTGQPGLQPH 553

E +CISSQ GC C+FC+TG GL+ +

Sbjct 102 ESFCISSQCGCNFGCKFCATGDIGLKKN 129

> AGH12814 23S rRNA methylase (plasmid) [Staphylococcus haemolyticus]

Length=349

Score = 37.7 bits (86), Expect = 8e-05, Method: Compositional matrix adjust.

Identities = 15/28 (54%), Positives = 20/28 (71%), Gaps = 0/28 (0%)

Frame = +2

Query 470 EPWCISSQAGCVVNCRFCSTGQPGLQPH 553

E +CISSQ GC C+FC+TG GL+ +

Sbjct 102 ESFCISSQCGCNFGCKFCATGDIGLKKN 129

> AFW17901 rRNA methylase (plasmid) [Enterococcus faecalis]

Length=349

Score = 37.7 bits (86), Expect = 8e-05, Method: Compositional matrix adjust.

Identities = 15/28 (54%), Positives = 20/28 (71%), Gaps = 0/28 (0%)

Frame = +2

Query 470 EPWCISSQAGCVVNCRFCSTGQPGLQPH 553

E +CISSQ GC C+FC+TG GL+ +

Sbjct 102 ESFCISSQCGCNFGCKFCATGDIGLKKN 129

> AFW17874 rRNA methylase (plasmid) [Enterococcus thailandicus]

Length=349

Score = 37.7 bits (86), Expect = 8e-05, Method: Compositional matrix adjust.

Identities = 15/28 (54%), Positives = 20/28 (71%), Gaps = 0/28 (0%)

Frame = +2

Query 470 EPWCISSQAGCVVNCRFCSTGQPGLQPH 553

E +CISSQ GC C+FC+TG GL+ +

Sbjct 102 ESFCISSQCGCNFGCKFCATGDIGLKKN 129

> AFO64605 23S rRNA methylase (plasmid) [Jeotgalicoccus pinnipedialis]

Length=349

Score = 37.7 bits (86), Expect = 8e-05, Method: Compositional matrix adjust.

Identities = 15/28 (54%), Positives = 20/28 (71%), Gaps = 0/28 (0%)

Frame = +2

Query 470 EPWCISSQAGCVVNCRFCSTGQPGLQPH 553

E +CISSQ GC C+FC+TG GL+ +

Sbjct 102 ESFCISSQCGCNFGCKFCATGDIGLKKN 129

> AFJ49143 florfenicol/chloramphenicol resistance protein (plasmid)

[Bacillus sp. pBS-03]

Length=349

Score = 37.7 bits (86), Expect = 8e-05, Method: Compositional matrix adjust.

Identities = 15/28 (54%), Positives = 20/28 (71%), Gaps = 0/28 (0%)

Frame = +2

Query 470 EPWCISSQAGCVVNCRFCSTGQPGLQPH 553

E +CISSQ GC C+FC+TG GL+ +

Sbjct 102 ESFCISSQCGCNFGCKFCATGDIGLKKN 129

> AFH78457 florfenicol/chloramphenicol resistance protein (plasmid)

[Escherichia coli]

Length=349

Score = 37.7 bits (86), Expect = 8e-05, Method: Compositional matrix adjust.

Identities = 15/28 (54%), Positives = 20/28 (71%), Gaps = 0/28 (0%)

Frame = +2

Query 470 EPWCISSQAGCVVNCRFCSTGQPGLQPH 553

E +CISSQ GC C+FC+TG GL+ +

Sbjct 102 ESFCISSQCGCNFGCKFCATGDIGLKKN 129

> AFG17053 Cfr (plasmid) [Enterococcus faecalis]

Length=349

Score = 37.7 bits (86), Expect = 8e-05, Method: Compositional matrix adjust.

Identities = 15/28 (54%), Positives = 20/28 (71%), Gaps = 0/28 (0%)

Frame = +2

Query 470 EPWCISSQAGCVVNCRFCSTGQPGLQPH 553

E +CISSQ GC C+FC+TG GL+ +

Sbjct 102 ESFCISSQCGCNFGCKFCATGDIGLKKN 129

> AEX20241 23S rRNA methylase (plasmid) [Staphylococcus cohnii]

Length=349

Score = 37.7 bits (86), Expect = 8e-05, Method: Compositional matrix adjust.

Identities = 15/28 (54%), Positives = 20/28 (71%), Gaps = 0/28 (0%)

Frame = +2

Query 470 EPWCISSQAGCVVNCRFCSTGQPGLQPH 553

E +CISSQ GC C+FC+TG GL+ +

Sbjct 102 ESFCISSQCGCNFGCKFCATGDIGLKKN 129

> AEX07579 23S rRNA methylase (plasmid) [Staphylococcus cohnii]

Length=349

Score = 37.7 bits (86), Expect = 8e-05, Method: Compositional matrix adjust.

Identities = 15/28 (54%), Positives = 20/28 (71%), Gaps = 0/28 (0%)

Frame = +2

Query 470 EPWCISSQAGCVVNCRFCSTGQPGLQPH 553

E +CISSQ GC C+FC+TG GL+ +

Sbjct 102 ESFCISSQCGCNFGCKFCATGDIGLKKN 129

> AEX01232 rRNA methylase [Staphylococcus cohnii]

Length=349

Score = 37.7 bits (86), Expect = 8e-05, Method: Compositional matrix adjust.

Identities = 15/28 (54%), Positives = 20/28 (71%), Gaps = 0/28 (0%)

Frame = +2

Query 470 EPWCISSQAGCVVNCRFCSTGQPGLQPH 553

E +CISSQ GC C+FC+TG GL+ +

Sbjct 102 ESFCISSQCGCNFGCKFCATGDIGLKKN 129

> AEX01231 rRNA methylase [Staphylococcus haemolyticus]

Length=349

Score = 37.7 bits (86), Expect = 8e-05, Method: Compositional matrix adjust.

Identities = 15/28 (54%), Positives = 20/28 (71%), Gaps = 0/28 (0%)

Frame = +2

Query 470 EPWCISSQAGCVVNCRFCSTGQPGLQPH 553

E +CISSQ GC C+FC+TG GL+ +

Sbjct 102 ESFCISSQCGCNFGCKFCATGDIGLKKN 129

> AEW23132 Cfr (plasmid) [Staphylococcus aureus]

Length=349

Score = 37.7 bits (86), Expect = 8e-05, Method: Compositional matrix adjust.

Identities = 15/28 (54%), Positives = 20/28 (71%), Gaps = 0/28 (0%)

Frame = +2

Query 470 EPWCISSQAGCVVNCRFCSTGQPGLQPH 553

E +CISSQ GC C+FC+TG GL+ +

Sbjct 102 ESFCISSQCGCNFGCKFCATGDIGLKKN 129

> AET37217 Cfr [Staphylococcus aureus]

Length=349

Score = 37.7 bits (86), Expect = 8e-05, Method: Compositional matrix adjust.

Identities = 15/28 (54%), Positives = 20/28 (71%), Gaps = 0/28 (0%)

Frame = +2

Query 470 EPWCISSQAGCVVNCRFCSTGQPGLQPH 553

E +CISSQ GC C+FC+TG GL+ +

Sbjct 102 ESFCISSQCGCNFGCKFCATGDIGLKKN 129

> AER50875 rRNA methylase [Proteus vulgaris]

Length=349

Score = 37.7 bits (86), Expect = 8e-05, Method: Compositional matrix adjust.

Identities = 15/28 (54%), Positives = 20/28 (71%), Gaps = 0/28 (0%)

Frame = +2

Query 470 EPWCISSQAGCVVNCRFCSTGQPGLQPH 553

E +CISSQ GC C+FC+TG GL+ +

Sbjct 102 ESFCISSQCGCNFGCKFCATGDIGLKKN 129

> AEP69239 23S rRNA methylase (plasmid) [Staphylococcus arlettae]

Length=349

Score = 37.7 bits (86), Expect = 8e-05, Method: Compositional matrix adjust.

Identities = 15/28 (54%), Positives = 20/28 (71%), Gaps = 0/28 (0%)

Frame = +2

Query 470 EPWCISSQAGCVVNCRFCSTGQPGLQPH 553

E +CISSQ GC C+FC+TG GL+ +

Sbjct 102 ESFCISSQCGCNFGCKFCATGDIGLKKN 129

> AEP69232 23S rRNA methylase (plasmid) [Staphylococcus saprophyticus]

Length=349

Score = 37.7 bits (86), Expect = 8e-05, Method: Compositional matrix adjust.

Identities = 15/28 (54%), Positives = 20/28 (71%), Gaps = 0/28 (0%)

Frame = +2

Query 470 EPWCISSQAGCVVNCRFCSTGQPGLQPH 553

E +CISSQ GC C+FC+TG GL+ +

Sbjct 102 ESFCISSQCGCNFGCKFCATGDIGLKKN 129

> AEP69220 23S rRNA methylase (plasmid) [Staphylococcus cohnii]

Length=349

Score = 37.7 bits (86), Expect = 8e-05, Method: Compositional matrix adjust.

Identities = 15/28 (54%), Positives = 20/28 (71%), Gaps = 0/28 (0%)

Frame = +2

Query 470 EPWCISSQAGCVVNCRFCSTGQPGLQPH 553

E +CISSQ GC C+FC+TG GL+ +

Sbjct 102 ESFCISSQCGCNFGCKFCATGDIGLKKN 129

> ADN44269 rRNA methylase (plasmid) [Bacillus sp. BS-02]

Length=349

Score = 37.7 bits (86), Expect = 8e-05, Method: Compositional matrix adjust.

Identities = 15/28 (54%), Positives = 20/28 (71%), Gaps = 0/28 (0%)

Frame = +2

Query 470 EPWCISSQAGCVVNCRFCSTGQPGLQPH 553

E +CISSQ GC C+FC+TG GL+ +

Sbjct 102 ESFCISSQCGCNFGCKFCATGDIGLKKN 129

> ACC77590 23S rRNA methylase (plasmid) [Staphylococcus aureus]

Length=349

Score = 37.7 bits (86), Expect = 8e-05, Method: Compositional matrix adjust.

Identities = 15/28 (54%), Positives = 20/28 (71%), Gaps = 0/28 (0%)

Frame = +2

Query 470 EPWCISSQAGCVVNCRFCSTGQPGLQPH 553

E +CISSQ GC C+FC+TG GL+ +

Sbjct 102 ESFCISSQCGCNFGCKFCATGDIGLKKN 129

> ABQ00063 Cfr [Staphylococcus aureus]

Length=349

Score = 37.7 bits (86), Expect = 8e-05, Method: Compositional matrix adjust.

Identities = 15/28 (54%), Positives = 20/28 (71%), Gaps = 0/28 (0%)

Frame = +2

Query 470 EPWCISSQAGCVVNCRFCSTGQPGLQPH 553

E +CISSQ GC C+FC+TG GL+ +

Sbjct 102 ESFCISSQCGCNFGCKFCATGDIGLKKN 129

> WP_003494750 Cfr family 23S rRNA (adenine(2503)-C(8))-methyltransferase

[Clostridium sporogenes]

Length=344

Score = 37.7 bits (86), Expect = 8e-05, Method: Compositional matrix adjust.

Identities = 15/26 (58%), Positives = 18/26 (69%), Gaps = 0/26 (0%)

Frame = +2

Query 470 EPWCISSQAGCVVNCRFCSTGQPGLQ 547

E +CISSQ GC CRFC+TG G +

Sbjct 101 ESFCISSQCGCGFGCRFCATGSAGFK 126

> EHN14160 chloramphenicol/florfenicol resistance protein [Clostridium

sporogenes PA 3679]

Length=344

Score = 37.7 bits (86), Expect = 8e-05, Method: Compositional matrix adjust.

Identities = 15/26 (58%), Positives = 18/26 (69%), Gaps = 0/26 (0%)

Frame = +2

Query 470 EPWCISSQAGCVVNCRFCSTGQPGLQ 547

E +CISSQ GC CRFC+TG G +

Sbjct 101 ESFCISSQCGCGFGCRFCATGSAGFK 126

> ALG88812 florfenicol/chloramphenicol resistance protein (plasmid)

[Escherichia coli]

Length=352

Score = 37.7 bits (86), Expect = 8e-05, Method: Compositional matrix adjust.

Identities = 15/28 (54%), Positives = 20/28 (71%), Gaps = 0/28 (0%)

Frame = +2

Query 470 EPWCISSQAGCVVNCRFCSTGQPGLQPH 553

E +CISSQ GC C+FC+TG GL+ +

Sbjct 105 ESFCISSQCGCNFGCKFCATGDIGLKKN 132

> ALG88797 florfenicol/chloramphenicol resistance protein [Escherichia

coli]

Length=352

Score = 37.7 bits (86), Expect = 8e-05, Method: Compositional matrix adjust.

Identities = 15/28 (54%), Positives = 20/28 (71%), Gaps = 0/28 (0%)

Frame = +2

Query 470 EPWCISSQAGCVVNCRFCSTGQPGLQPH 553

E +CISSQ GC C+FC+TG GL+ +

Sbjct 105 ESFCISSQCGCNFGCKFCATGDIGLKKN 132

> AFR11502 rRNA methylase (plasmid) [Staphylococcus capitis]

Length=352

Score = 37.7 bits (86), Expect = 8e-05, Method: Compositional matrix adjust.

Identities = 15/28 (54%), Positives = 20/28 (71%), Gaps = 0/28 (0%)

Frame = +2

Query 470 EPWCISSQAGCVVNCRFCSTGQPGLQPH 553

E +CISSQ GC C+FC+TG GL+ +

Sbjct 105 ESFCISSQCGCNFGCKFCATGDIGLKKN 132

> cfr_-_8918998_translation cfr: rRNA methylase

Length=349

Score = 37.7 bits (86), Expect = 8e-05, Method: Compositional matrix adjust.

Identities = 15/28 (54%), Positives = 20/28 (71%), Gaps = 0/28 (0%)

Frame = +2

Query 470 EPWCISSQAGCVVNCRFCSTGQPGLQPH 553

E +CISSQ GC C+FC+TG GL+ +

Sbjct 102 ESFCISSQCGCNFGCKFCATGDIGLKKN 129

> YP_003533077 rRNA methylase (plasmid) [Bacillus sp. BS-01]

Length=349

Score = 37.7 bits (86), Expect = 8e-05, Method: Compositional matrix adjust.

Identities = 15/28 (54%), Positives = 20/28 (71%), Gaps = 0/28 (0%)

Frame = +2

Query 470 EPWCISSQAGCVVNCRFCSTGQPGLQPH 553

E +CISSQ GC C+FC+TG GL+ +

Sbjct 102 ESFCISSQCGCNFGCKFCATGDIGLKKN 129

> WP_013034930 Cfr family 23S rRNA (adenine(2503)-C(8))-methyltransferase

[Bacillus sp. BS-01]

Length=349

Score = 37.7 bits (86), Expect = 8e-05, Method: Compositional matrix adjust.

Identities = 15/28 (54%), Positives = 20/28 (71%), Gaps = 0/28 (0%)

Frame = +2

Query 470 EPWCISSQAGCVVNCRFCSTGQPGLQPH 553

E +CISSQ GC C+FC+TG GL+ +

Sbjct 102 ESFCISSQCGCNFGCKFCATGDIGLKKN 129

> ADD91311 rRNA methylase (plasmid) [Bacillus sp. BS-01]

Length=349

Score = 37.7 bits (86), Expect = 8e-05, Method: Compositional matrix adjust.

Identities = 15/28 (54%), Positives = 20/28 (71%), Gaps = 0/28 (0%)

Frame = +2

Query 470 EPWCISSQAGCVVNCRFCSTGQPGLQPH 553

E +CISSQ GC C+FC+TG GL+ +

Sbjct 102 ESFCISSQCGCNFGCKFCATGDIGLKKN 129

> ARQ19305 chloramphenicol-florfenicol resistance protein (plasmid)

[Enterococcus faecium]

Length=374

Score = 37.7 bits (86), Expect = 8e-05, Method: Compositional matrix adjust.

Identities = 15/28 (54%), Positives = 20/28 (71%), Gaps = 0/28 (0%)

Frame = +2

Query 470 EPWCISSQAGCVVNCRFCSTGQPGLQPH 553

E +CISSQ GC C+FC+TG GL+ +

Sbjct 127 ESFCISSQCGCNFGCKFCATGDIGLKKN 154

> YP_006958115 rRNA methylase (plasmid) [Staphylococcus aureus]

Length=352

Score = 37.7 bits (86), Expect = 8e-05, Method: Compositional matrix adjust.

Identities = 15/28 (54%), Positives = 20/28 (71%), Gaps = 0/28 (0%)

Frame = +2

Query 470 EPWCISSQAGCVVNCRFCSTGQPGLQPH 553

E +CISSQ GC C+FC+TG GL+ +

Sbjct 105 ESFCISSQCGCNFGCKFCATGDIGLKKN 132

> WP_001835153 Cfr family 23S rRNA (adenine(2503)-C(8))-methyltransferase

[Staphylococcus aureus]

Length=352

Score = 37.7 bits (86), Expect = 8e-05, Method: Compositional matrix adjust.

Identities = 15/28 (54%), Positives = 20/28 (71%), Gaps = 0/28 (0%)

Frame = +2

Query 470 EPWCISSQAGCVVNCRFCSTGQPGLQPH 553

E +CISSQ GC C+FC+TG GL+ +

Sbjct 105 ESFCISSQCGCNFGCKFCATGDIGLKKN 132

> EJU86205 23S rRNA methyltransferase [Enterococcus faecalis 599]

Length=352

Score = 37.7 bits (86), Expect = 8e-05, Method: Compositional matrix adjust.

Identities = 15/28 (54%), Positives = 20/28 (71%), Gaps = 0/28 (0%)

Frame = +2

Query 470 EPWCISSQAGCVVNCRFCSTGQPGLQPH 553

E +CISSQ GC C+FC+TG GL+ +

Sbjct 105 ESFCISSQCGCNFGCKFCATGDIGLKKN 132

> EJU83061 23S rRNA methyltransferase [Staphylococcus aureus subsp.

aureus CM05]

Length=352

Score = 37.7 bits (86), Expect = 8e-05, Method: Compositional matrix adjust.

Identities = 15/28 (54%), Positives = 20/28 (71%), Gaps = 0/28 (0%)

Frame = +2

Query 470 EPWCISSQAGCVVNCRFCSTGQPGLQPH 553

E +CISSQ GC C+FC+TG GL+ +

Sbjct 105 ESFCISSQCGCNFGCKFCATGDIGLKKN 132

> AKJ75143 rRNA methylase (plasmid) [Staphylococcus epidermidis]

Length=352

Score = 37.7 bits (86), Expect = 8e-05, Method: Compositional matrix adjust.

Identities = 15/28 (54%), Positives = 20/28 (71%), Gaps = 0/28 (0%)

Frame = +2

Query 470 EPWCISSQAGCVVNCRFCSTGQPGLQPH 553

E +CISSQ GC C+FC+TG GL+ +

Sbjct 105 ESFCISSQCGCNFGCKFCATGDIGLKKN 132

> AGB06240 Cfr methyltransferase [Staphylococcus epidermidis]

Length=352

Score = 37.7 bits (86), Expect = 8e-05, Method: Compositional matrix adjust.

Identities = 15/28 (54%), Positives = 20/28 (71%), Gaps = 0/28 (0%)

Frame = +2

Query 470 EPWCISSQAGCVVNCRFCSTGQPGLQPH 553

E +CISSQ GC C+FC+TG GL+ +

Sbjct 105 ESFCISSQCGCNFGCKFCATGDIGLKKN 132

> AFW17854 rRNA methylase (plasmid) [Enterococcus thailandicus]

Length=352

Score = 37.7 bits (86), Expect = 8e-05, Method: Compositional matrix adjust.

Identities = 15/28 (54%), Positives = 20/28 (71%), Gaps = 0/28 (0%)

Frame = +2

Query 470 EPWCISSQAGCVVNCRFCSTGQPGLQPH 553

E +CISSQ GC C+FC+TG GL+ +

Sbjct 105 ESFCISSQCGCNFGCKFCATGDIGLKKN 132

> AFF18416 rRNA methylase (plasmid) [Staphylococcus aureus]

Length=352

Score = 37.7 bits (86), Expect = 8e-05, Method: Compositional matrix adjust.

Identities = 15/28 (54%), Positives = 20/28 (71%), Gaps = 0/28 (0%)

Frame = +2

Query 470 EPWCISSQAGCVVNCRFCSTGQPGLQPH 553

E +CISSQ GC C+FC+TG GL+ +

Sbjct 105 ESFCISSQCGCNFGCKFCATGDIGLKKN 132

> WP_002349981 23S rRNA (adenine(2503)-C(8))-methyltransferase

Cfr(B) [Enterococcus faecium]

Length=349

Score = 37.4 bits (85), Expect = 1e-04, Method: Compositional matrix adjust.

Identities = 15/26 (58%), Positives = 19/26 (73%), Gaps = 0/26 (0%)

Frame = +2

Query 470 EPWCISSQAGCVVNCRFCSTGQPGLQ 547

E +CISSQ GC C+FC+TG GL+

Sbjct 100 ESFCISSQCGCNFGCKFCATGDIGLK 125

> EJX57584 23S rRNA methyltransferase [Enterococcus faecium R497]

Length=349

Score = 37.4 bits (85), Expect = 1e-04, Method: Compositional matrix adjust.

Identities = 15/26 (58%), Positives = 19/26 (73%), Gaps = 0/26 (0%)

Frame = +2

Query 470 EPWCISSQAGCVVNCRFCSTGQPGLQ 547

E +CISSQ GC C+FC+TG GL+

Sbjct 100 ESFCISSQCGCNFGCKFCATGDIGLK 125

> AKV84429 chloramphenicol/ fluorphenicol resistance protein [Enterococcus

faecium]

Length=349

Score = 37.4 bits (85), Expect = 1e-04, Method: Compositional matrix adjust.

Identities = 15/26 (58%), Positives = 19/26 (73%), Gaps = 0/26 (0%)

Frame = +2

Query 470 EPWCISSQAGCVVNCRFCSTGQPGLQ 547

E +CISSQ GC C+FC+TG GL+

Sbjct 100 ESFCISSQCGCNFGCKFCATGDIGLK 125

> AIX48090 23S methyltransferase [Clostridioides difficile]

Length=349

Score = 37.4 bits (85), Expect = 1e-04, Method: Compositional matrix adjust.

Identities = 15/26 (58%), Positives = 19/26 (73%), Gaps = 0/26 (0%)

Frame = +2

Query 470 EPWCISSQAGCVVNCRFCSTGQPGLQ 547

E +CISSQ GC C+FC+TG GL+

Sbjct 100 ESFCISSQCGCNFGCKFCATGDIGLK 125

> WP_002405682 MULTISPECIES: Cfr family 23S rRNA (adenine(2503)-C(8))-methyltransferase

[Firmicutes]

Length=349

Score = 37.4 bits (85), Expect = 1e-04, Method: Compositional matrix adjust.

Identities = 15/26 (58%), Positives = 19/26 (73%), Gaps = 0/26 (0%)

Frame = +2

Query 470 EPWCISSQAGCVVNCRFCSTGQPGLQ 547

E +CISSQ GC C+FC+TG GL+

Sbjct 100 ESFCISSQCGCNFGCKFCATGDIGLK 125

> EQF87020 ribosomal RNA large subunit methyltransferase N [Clostridioides

difficile 824]

Length=349

Score = 37.4 bits (85), Expect = 1e-04, Method: Compositional matrix adjust.

Identities = 15/26 (58%), Positives = 19/26 (73%), Gaps = 0/26 (0%)

Frame = +2

Query 470 EPWCISSQAGCVVNCRFCSTGQPGLQ 547

E +CISSQ GC C+FC+TG GL+

Sbjct 100 ESFCISSQCGCNFGCKFCATGDIGLK 125

> EOI92877 ribosomal RNA large subunit methyltransferase Cfr [Enterococcus

faecalis EnGen0311]

Length=349

Score = 37.4 bits (85), Expect = 1e-04, Method: Compositional matrix adjust.

Identities = 15/26 (58%), Positives = 19/26 (73%), Gaps = 0/26 (0%)

Frame = +2

Query 470 EPWCISSQAGCVVNCRFCSTGQPGLQ 547

E +CISSQ GC C+FC+TG GL+

Sbjct 100 ESFCISSQCGCNFGCKFCATGDIGLK 125

> EOI25076 ribosomal RNA large subunit methyltransferase Cfr [Enterococcus

faecalis EnGen0251]

Length=349

Score = 37.4 bits (85), Expect = 1e-04, Method: Compositional matrix adjust.

Identities = 15/26 (58%), Positives = 19/26 (73%), Gaps = 0/26 (0%)

Frame = +2

Query 470 EPWCISSQAGCVVNCRFCSTGQPGLQ 547

E +CISSQ GC C+FC+TG GL+

Sbjct 100 ESFCISSQCGCNFGCKFCATGDIGLK 125

> EFQ16578 23S rRNA m2A2503 methyltransferase [Enterococcus faecalis

EnGen0311]

Length=349

Score = 37.4 bits (85), Expect = 1e-04, Method: Compositional matrix adjust.

Identities = 15/26 (58%), Positives = 19/26 (73%), Gaps = 0/26 (0%)

Frame = +2

Query 470 EPWCISSQAGCVVNCRFCSTGQPGLQ 547

E +CISSQ GC C+FC+TG GL+

Sbjct 100 ESFCISSQCGCNFGCKFCATGDIGLK 125

> CDF47262 chloramphenicol/fluorfenicol resistance protein, radical

SAM enzyme, cfr family [Clostridioides difficile]

Length=349

Score = 37.4 bits (85), Expect = 1e-04, Method: Compositional matrix adjust.

Identities = 15/26 (58%), Positives = 19/26 (73%), Gaps = 0/26 (0%)

Frame = +2

Query 470 EPWCISSQAGCVVNCRFCSTGQPGLQ 547

E +CISSQ GC C+FC+TG GL+

Sbjct 100 ESFCISSQCGCNFGCKFCATGDIGLK 125

> AIX48091 23S methyltransferase [Clostridioides difficile]

Length=349

Score = 37.4 bits (85), Expect = 1e-04, Method: Compositional matrix adjust.

Identities = 15/26 (58%), Positives = 19/26 (73%), Gaps = 0/26 (0%)

Frame = +2

Query 470 EPWCISSQAGCVVNCRFCSTGQPGLQ 547

E +CISSQ GC C+FC+TG GL+

Sbjct 100 ESFCISSQCGCNFGCKFCATGDIGLK 125

> WP_053594538 Cfr family 23S rRNA (adenine(2503)-C(8))-methyltransferase

[Lysinibacillus sp. FJAT-14222]

Length=350

Score = 37.0 bits (84), Expect = 1e-04, Method: Compositional matrix adjust.

Identities = 15/26 (58%), Positives = 19/26 (73%), Gaps = 0/26 (0%)

Frame = +2

Query 470 EPWCISSQAGCVVNCRFCSTGQPGLQ 547

E +CISSQ GC C+FC+TG GL+

Sbjct 100 ESFCISSQCGCNFGCKFCATGDLGLK 125

> KOS62985 23S rRNA (adenine(2503)-C8)-methyltransferase [Lysinibacillus

sp. FJAT-14222]

Length=350

Score = 37.0 bits (84), Expect = 1e-04, Method: Compositional matrix adjust.

Identities = 15/26 (58%), Positives = 19/26 (73%), Gaps = 0/26 (0%)

Frame = +2

Query 470 EPWCISSQAGCVVNCRFCSTGQPGLQ 547

E +CISSQ GC C+FC+TG GL+

Sbjct 100 ESFCISSQCGCNFGCKFCATGDLGLK 125

> WP_094423516 Cfr family 23S rRNA (adenine(2503)-C(8))-methyltransferase

[Bacillus clausii]

Length=350

Score = 36.6 bits (83), Expect = 2e-04, Method: Compositional matrix adjust.

Identities = 16/26 (62%), Positives = 19/26 (73%), Gaps = 0/26 (0%)

Frame = +2

Query 470 EPWCISSQAGCVVNCRFCSTGQPGLQ 547

E +CISSQ GC + C FCSTG GL+

Sbjct 103 ESFCISSQCGCGLGCTFCSTGAIGLK 128

> AST94603 23S rRNA (adenine(2503)-C(8))-methyltransferase Cfr

[Bacillus clausii]

Length=350

Score = 36.6 bits (83), Expect = 2e-04, Method: Compositional matrix adjust.

Identities = 16/26 (62%), Positives = 19/26 (73%), Gaps = 0/26 (0%)

Frame = +2

Query 470 EPWCISSQAGCVVNCRFCSTGQPGLQ 547

E +CISSQ GC + C FCSTG GL+

Sbjct 103 ESFCISSQCGCGLGCTFCSTGAIGLK 128

> WP_095294851 Cfr family 23S rRNA (adenine(2503)-C(8))-methyltransferase

[Bacillus clausii]

Length=350

Score = 36.6 bits (83), Expect = 2e-04, Method: Compositional matrix adjust.

Identities = 16/26 (62%), Positives = 19/26 (73%), Gaps = 0/26 (0%)

Frame = +2

Query 470 EPWCISSQAGCVVNCRFCSTGQPGLQ 547

E +CISSQ GC + C FCSTG GL+

Sbjct 103 ESFCISSQCGCGLGCTFCSTGAIGLK 128

> WP_011245929 MULTISPECIES: 23S rRNA (adenine(2503)-C(8))-methyltransferase

Cfr [Bacillus]

Length=350

Score = 36.6 bits (83), Expect = 2e-04, Method: Compositional matrix adjust.

Identities = 16/26 (62%), Positives = 19/26 (73%), Gaps = 0/26 (0%)

Frame = +2

Query 470 EPWCISSQAGCVVNCRFCSTGQPGLQ 547

E +CISSQ GC + C FCSTG GL+

Sbjct 103 ESFCISSQCGCGLGCTFCSTGAIGLK 128

> CFR_BACSK RecName: Full=Ribosomal RNA large subunit methyltransferase

Cfr; AltName: Full=23S rRNA (adenine(2503)-C(8))-methyltransferase;

AltName: Full=23S rRNA m8A2503 methyltransferase

Length=350

Score = 36.6 bits (83), Expect = 2e-04, Method: Compositional matrix adjust.

Identities = 16/26 (62%), Positives = 19/26 (73%), Gaps = 0/26 (0%)

Frame = +2

Query 470 EPWCISSQAGCVVNCRFCSTGQPGLQ 547

E +CISSQ GC + C FCSTG GL+

Sbjct 103 ESFCISSQCGCGLGCTFCSTGAIGLK 128

> BAD63613 florfenicol/chloramphenicol resistance protein [Bacillus

clausii KSM-K16]

Length=350

Score = 36.6 bits (83), Expect = 2e-04, Method: Compositional matrix adjust.

Identities = 16/26 (62%), Positives = 19/26 (73%), Gaps = 0/26 (0%)

Frame = +2

Query 470 EPWCISSQAGCVVNCRFCSTGQPGLQ 547

E +CISSQ GC + C FCSTG GL+

Sbjct 103 ESFCISSQCGCGLGCTFCSTGAIGLK 128

> ALA51754 Ribosomal RNA large subunit methyltransferase N [Bacillus

clausii]

Length=350

Score = 36.6 bits (83), Expect = 2e-04, Method: Compositional matrix adjust.

Identities = 16/26 (62%), Positives = 19/26 (73%), Gaps = 0/26 (0%)

Frame = +2

Query 470 EPWCISSQAGCVVNCRFCSTGQPGLQ 547

E +CISSQ GC + C FCSTG GL+

Sbjct 103 ESFCISSQCGCGLGCTFCSTGAIGLK 128

> WP_063608639 Cfr family 23S rRNA (adenine(2503)-C(8))-methyltransferase

[Bacillus clausii]

Length=350

Score = 36.6 bits (83), Expect = 2e-04, Method: Compositional matrix adjust.

Identities = 16/26 (62%), Positives = 19/26 (73%), Gaps = 0/26 (0%)

Frame = +2

Query 470 EPWCISSQAGCVVNCRFCSTGQPGLQ 547

E +CISSQ GC + C FCSTG GL+

Sbjct 103 ESFCISSQCGCGLGCTFCSTGAIGLK 128

> KKI87196 chloramphenicol/florfenicol resistance protein [Bacillus

clausii]

Length=350

Score = 36.6 bits (83), Expect = 2e-04, Method: Compositional matrix adjust.

Identities = 16/26 (62%), Positives = 19/26 (73%), Gaps = 0/26 (0%)

Frame = +2

Query 470 EPWCISSQAGCVVNCRFCSTGQPGLQ 547

E +CISSQ GC + C FCSTG GL+

Sbjct 103 ESFCISSQCGCGLGCTFCSTGAIGLK 128

> WP_095327308 Cfr family 23S rRNA (adenine(2503)-C(8))-methyltransferase

[Bacillus clausii]

Length=350

Score = 36.6 bits (83), Expect = 2e-04, Method: Compositional matrix adjust.

Identities = 16/26 (62%), Positives = 19/26 (73%), Gaps = 0/26 (0%)

Frame = +2

Query 470 EPWCISSQAGCVVNCRFCSTGQPGLQ 547

E +CISSQ GC + C FCSTG GL+

Sbjct 103 ESFCISSQCGCGLGCTFCSTGAIGLK 128

> WP_095319205 Cfr family 23S rRNA (adenine(2503)-C(8))-methyltransferase

[Bacillus clausii]

Length=350

Score = 36.2 bits (82), Expect = 2e-04, Method: Compositional matrix adjust.

Identities = 16/26 (62%), Positives = 19/26 (73%), Gaps = 0/26 (0%)

Frame = +2

Query 470 EPWCISSQAGCVVNCRFCSTGQPGLQ 547

E +CISSQ GC + C FCSTG GL+

Sbjct 103 ESFCISSQCGCGLGCAFCSTGAIGLK 128

> AAN87489 florfenicol resistance protein, partial [Heliobacillus

mobilis]

Length=360

Score = 35.4 bits (80), Expect = 5e-04, Method: Compositional matrix adjust.

Identities = 13/23 (57%), Positives = 19/23 (83%), Gaps = 0/23 (0%)

Frame = +2

Query 479 CISSQAGCVVNCRFCSTGQPGLQ 547

C+S+QAGC + C+FC+TGQ G +

Sbjct 114 CLSTQAGCPLGCKFCATGQQGFR 136

> WP_090913837 Cfr family 23S rRNA (adenine(2503)-C(8))-methyltransferase

[Paenibacillus sp. cl141a]

Length=346

Score = 35.0 bits (79), Expect = 5e-04, Method: Compositional matrix adjust.

Identities = 15/26 (58%), Positives = 18/26 (69%), Gaps = 0/26 (0%)

Frame = +2

Query 470 EPWCISSQAGCVVNCRFCSTGQPGLQ 547

E +CISSQ GC C FC+TG GL+

Sbjct 100 ESYCISSQCGCGFGCTFCATGTLGLK 125

> SEM59396 23S rRNA (adenine-C8)-methyltransferase [Paenibacillus

sp. cl141a]

Length=346

Score = 35.0 bits (79), Expect = 5e-04, Method: Compositional matrix adjust.

Identities = 15/26 (58%), Positives = 18/26 (69%), Gaps = 0/26 (0%)

Frame = +2

Query 470 EPWCISSQAGCVVNCRFCSTGQPGLQ 547

E +CISSQ GC C FC+TG GL+

Sbjct 100 ESYCISSQCGCGFGCTFCATGTLGLK 125

> WP_096773940 Cfr family 23S rRNA (adenine(2503)-C(8))-methyltransferase

[Paenibacillus lautus]

Length=346

Score = 35.0 bits (79), Expect = 5e-04, Method: Compositional matrix adjust.

Identities = 15/26 (58%), Positives = 18/26 (69%), Gaps = 0/26 (0%)

Frame = +2

Query 470 EPWCISSQAGCVVNCRFCSTGQPGLQ 547

E +CISSQ GC C FC+TG GL+

Sbjct 100 ESYCISSQCGCGFGCTFCATGTLGLK 125

> WP_036660664 Cfr family 23S rRNA (adenine(2503)-C(8))-methyltransferase

[Paenibacillus sp. FSL H8-457]

Length=346

Score = 35.0 bits (79), Expect = 5e-04, Method: Compositional matrix adjust.

Identities = 15/26 (58%), Positives = 18/26 (69%), Gaps = 0/26 (0%)

Frame = +2

Query 470 EPWCISSQAGCVVNCRFCSTGQPGLQ 547

E +CISSQ GC C FC+TG GL+

Sbjct 100 ESYCISSQCGCGFGCTFCATGTLGLK 125

> WP_015735625 Cfr family 23S rRNA (adenine(2503)-C(8))-methyltransferase

[Paenibacillus sp. Y412MC10]

Length=346

Score = 35.0 bits (79), Expect = 5e-04, Method: Compositional matrix adjust.

Identities = 15/26 (58%), Positives = 18/26 (69%), Gaps = 0/26 (0%)

Frame = +2

Query 470 EPWCISSQAGCVVNCRFCSTGQPGLQ 547

E +CISSQ GC C FC+TG GL+

Sbjct 100 ESYCISSQCGCGFGCTFCATGTLGLK 125

> ETT66883 chloramphenicol/florfenicol resistance protein [Paenibacillus

sp. FSL H8-457]

Length=346

Score = 35.0 bits (79), Expect = 5e-04, Method: Compositional matrix adjust.

Identities = 15/26 (58%), Positives = 18/26 (69%), Gaps = 0/26 (0%)

Frame = +2

Query 470 EPWCISSQAGCVVNCRFCSTGQPGLQ 547

E +CISSQ GC C FC+TG GL+

Sbjct 100 ESYCISSQCGCGFGCTFCATGTLGLK 125

> ACX65640 radical SAM enzyme, Cfr family [Paenibacillus sp. Y412MC10]

Length=346

Score = 35.0 bits (79), Expect = 5e-04, Method: Compositional matrix adjust.

Identities = 15/26 (58%), Positives = 18/26 (69%), Gaps = 0/26 (0%)

Frame = +2

Query 470 EPWCISSQAGCVVNCRFCSTGQPGLQ 547

E +CISSQ GC C FC+TG GL+

Sbjct 100 ESYCISSQCGCGFGCTFCATGTLGLK 125

> WP_009595405 Cfr family 23S rRNA (adenine(2503)-C(8))-methyltransferase

[Paenibacillus sp. HGF5]

Length=346

Score = 35.0 bits (79), Expect = 6e-04, Method: Compositional matrix adjust.

Identities = 15/26 (58%), Positives = 18/26 (69%), Gaps = 0/26 (0%)

Frame = +2

Query 470 EPWCISSQAGCVVNCRFCSTGQPGLQ 547

E +CISSQ GC C FC+TG GL+

Sbjct 100 ESYCISSQCGCGFGCTFCATGTLGLK 125

> EGG31816 23S rRNA m2A2503 methyltransferase [Paenibacillus sp.

HGF5]

Length=346

Score = 35.0 bits (79), Expect = 6e-04, Method: Compositional matrix adjust.

Identities = 15/26 (58%), Positives = 18/26 (69%), Gaps = 0/26 (0%)

Frame = +2

Query 470 EPWCISSQAGCVVNCRFCSTGQPGLQ 547

E +CISSQ GC C FC+TG GL+

Sbjct 100 ESYCISSQCGCGFGCTFCATGTLGLK 125

> XP_009692050 florfenicol resistance protein [Theileria orientalis

strain Shintoku]

Length=286

Score = 34.7 bits (78), Expect = 8e-04, Method: Compositional matrix adjust.

Identities = 14/23 (61%), Positives = 18/23 (78%), Gaps = 0/23 (0%)

Frame = +2

Query 479 CISSQAGCVVNCRFCSTGQPGLQ 547

CISSQ GC +C FC+TG+ GL+

Sbjct 102 CISSQVGCSYSCSFCATGKIGLK 124

> BAM41749 florfenicol resistance protein [Theileria orientalis

strain Shintoku]

Length=286

Score = 34.7 bits (78), Expect = 8e-04, Method: Compositional matrix adjust.

Identities = 14/23 (61%), Positives = 18/23 (78%), Gaps = 0/23 (0%)

Frame = +2

Query 479 CISSQAGCVVNCRFCSTGQPGLQ 547

CISSQ GC +C FC+TG+ GL+

Sbjct 102 CISSQVGCSYSCSFCATGKIGLK 124

> WP_004442525 Cfr family 23S rRNA (adenine(2503)-C(8))-methyltransferase

[Clostridium botulinum]

Length=344

Score = 34.7 bits (78), Expect = 8e-04, Method: Compositional matrix adjust.

Identities = 14/26 (54%), Positives = 17/26 (65%), Gaps = 0/26 (0%)

Frame = +2

Query 470 EPWCISSQAGCVVNCRFCSTGQPGLQ 547

E +CIS Q GC CRFC+TG G +

Sbjct 101 ESFCISCQCGCSFGCRFCATGSVGFK 126

> EDT84436 florfenicol/chloramphenicol resistance protein [Clostridium

botulinum Bf]

Length=344

Score = 34.7 bits (78), Expect = 8e-04, Method: Compositional matrix adjust.

Identities = 14/26 (54%), Positives = 17/26 (65%), Gaps = 0/26 (0%)

Frame = +2

Query 470 EPWCISSQAGCVVNCRFCSTGQPGLQ 547

E +CIS Q GC CRFC+TG G +

Sbjct 101 ESFCISCQCGCSFGCRFCATGSVGFK 126

> AJE11066 23S rRNA methyltransferase [Clostridium botulinum CDC_1436]

Length=344

Score = 34.7 bits (78), Expect = 8e-04, Method: Compositional matrix adjust.

Identities = 14/26 (54%), Positives = 17/26 (65%), Gaps = 0/26 (0%)

Frame = +2

Query 470 EPWCISSQAGCVVNCRFCSTGQPGLQ 547

E +CIS Q GC CRFC+TG G +

Sbjct 101 ESFCISCQCGCSFGCRFCATGSVGFK 126

> ACQ52150 florfenicol/chloramphenicol resistance protein [Clostridium

botulinum Ba4 str. 657]

Length=344

Score = 34.7 bits (78), Expect = 8e-04, Method: Compositional matrix adjust.

Identities = 14/26 (54%), Positives = 17/26 (65%), Gaps = 0/26 (0%)

Frame = +2

Query 470 EPWCISSQAGCVVNCRFCSTGQPGLQ 547

E +CIS Q GC CRFC+TG G +

Sbjct 101 ESFCISCQCGCSFGCRFCATGSVGFK 126

> ADO82581 23S rRNA m(2)A-2503 methyltransferase [Ilyobacter polytropus

DSM 2926]

Length=349

Score = 34.7 bits (78), Expect = 9e-04, Method: Compositional matrix adjust.

Identities = 17/29 (59%), Positives = 18/29 (62%), Gaps = 1/29 (3%)

Frame = +2

Query 458 KERAEPWCISSQAGCVVNCRFCSTGQPGL 544

KER CISSQ GC V C FC+TG G

Sbjct 101 KER-NTLCISSQVGCPVKCSFCATGLDGF 128

> WP_095305967 Cfr family 23S rRNA (adenine(2503)-C(8))-methyltransferase

[Bacillus clausii]

Length=350

Score = 34.3 bits (77), Expect = 0.001, Method: Compositional matrix adjust.

Identities = 15/26 (58%), Positives = 18/26 (69%), Gaps = 0/26 (0%)

Frame = +2

Query 470 EPWCISSQAGCVVNCRFCSTGQPGLQ 547

E +CISSQ GC + C FCS G GL+

Sbjct 103 ESFCISSQCGCGLGCTFCSAGAIGLK 128

> ABX42587 radical SAM enzyme, Cfr family [Lachnoclostridium phytofermentans

ISDg]

Length=344

Score = 33.9 bits (76), Expect = 0.001, Method: Compositional matrix adjust.

Identities = 14/24 (58%), Positives = 17/24 (71%), Gaps = 0/24 (0%)

Frame = +2

Query 470 EPWCISSQAGCVVNCRFCSTGQPG 541

E +CISSQ GC C+FC+TG G

Sbjct 99 ESFCISSQCGCGFGCQFCATGTLG 122

> AAM72416 florfenicol resistance protein, putative [Chlorobium

tepidum TLS]

Length=374

Score = 33.9 bits (76), Expect = 0.001, Method: Compositional matrix adjust.

Identities = 16/32 (50%), Positives = 20/32 (63%), Gaps = 1/32 (3%)

Frame = +2

Query 452 PRKERAEPWCISSQAGCVVNCRFCSTGQPGLQ 547

P +ER CISSQ GC + C FC+TG G +

Sbjct 124 PSEERI-TACISSQIGCPLRCTFCATGHMGFR 154

> CEP78658 23S rRNA (adenine(2503)-C(8))-methyltransferase [Defluviitoga

tunisiensis]

Length=346

Score = 33.5 bits (75), Expect = 0.002, Method: Compositional matrix adjust.

Identities = 13/22 (59%), Positives = 16/22 (73%), Gaps = 0/22 (0%)

Frame = +2

Query 479 CISSQAGCVVNCRFCSTGQPGL 544

CIS+Q GC + C FCSTG+ G

Sbjct 107 CISTQVGCPLKCAFCSTGKSGF 128

> AHG94326 chloramphenicol florfenicol resistance protein, partial

[Staphylococcus haemolyticus]

Length=234

Score = 31.6 bits (70), Expect = 0.007, Method: Compositional matrix adjust.

Identities = 13/22 (59%), Positives = 16/22 (73%), Gaps = 0/22 (0%)

Frame = +2

Query 482 ISSQAGCVVNCRFCSTGQPGLQ 547

ISSQ GC C+FC+TG GL+

Sbjct 1 ISSQCGCNFGCKFCATGDIGLK 22

> AAL53048 florfenicol resistance protein [Brucella melitensis

bv. 1 str. 16M]

Length=411

Score = 31.2 bits (69), Expect = 0.011, Method: Compositional matrix adjust.

Identities = 12/18 (67%), Positives = 13/18 (72%), Gaps = 0/18 (0%)

Frame = +2

Query 479 CISSQAGCVVNCRFCSTG 532

CISSQ GC + C FC TG

Sbjct 138 CISSQVGCTLTCSFCHTG 155

> ACE04355 radical SAM enzyme, Cfr family [Chlorobium phaeobacteroides

BS1]

Length=362

Score = 30.8 bits (68), Expect = 0.014, Method: Compositional matrix adjust.

Identities = 12/22 (55%), Positives = 15/22 (68%), Gaps = 0/22 (0%)

Frame = +2

Query 479 CISSQAGCVVNCRFCSTGQPGL 544

C+SSQ GC + C FC+TG G

Sbjct 112 CVSSQVGCPLRCTFCATGYMGF 133

> ABS12821 radical SAM enzyme, Cfr family [Ochrobactrum anthropi

ATCC 49188]

Length=411

Score = 30.4 bits (67), Expect = 0.019, Method: Compositional matrix adjust.

Identities = 11/18 (61%), Positives = 13/18 (72%), Gaps = 0/18 (0%)

Frame = +2

Query 479 CISSQAGCVVNCRFCSTG 532

C+SSQ GC + C FC TG

Sbjct 138 CVSSQVGCTLTCSFCHTG 155

> EED10097 radical SAM enzyme, Cfr family [Thermus aquaticus Y51MC23]

Length=349

Score = 30.0 bits (66), Expect = 0.022, Method: Compositional matrix adjust.

Identities = 16/46 (35%), Positives = 22/46 (48%), Gaps = 4/46 (9%)

Frame = +2

Query 407 VFDGRPGK*P*NPFSPRKERAEPWCISSQAGCVVNCRFCSTGQPGL 544

+ DG+ + P+ RK C+SS GC C FC+TG G

Sbjct 76 LLDGKKTEAVYMPYKNRKT----VCLSSMVGCPAGCTFCATGALGF 117

> ACV39069 radical SAM enzyme, Cfr family [Leptotrichia buccalis

C-1013-b]

Length=372

Score = 29.6 bits (65), Expect = 0.035, Method: Compositional matrix adjust.

Identities = 10/18 (56%), Positives = 14/18 (78%), Gaps = 0/18 (0%)

Frame = +2

Query 479 CISSQAGCVVNCRFCSTG 532

C+SSQ GC++ C FC+T

Sbjct 125 CVSSQIGCLIGCDFCATA 142

> AAS81916 florfenicol resistance protein [Thermus thermophilus

HB27]

Length=355

Score = 29.3 bits (64), Expect = 0.048, Method: Compositional matrix adjust.

Identities = 15/46 (33%), Positives = 22/46 (48%), Gaps = 4/46 (9%)

Frame = +2

Query 407 VFDGRPGK*P*NPFSPRKERAEPWCISSQAGCVVNCRFCSTGQPGL 544

+ DG+ + P+ RK C+S+ GC C FC+TG G

Sbjct 82 LLDGKKTEAVYMPYENRKT----VCLSTMVGCPAGCTFCATGALGF 123

> AEE96185 23S rRNA m(2)A-2503 methyltransferase [Mahella australiensis

50-1 BON]

Length=344

Score = 28.5 bits (62), Expect = 0.077, Method: Compositional matrix adjust.

Identities = 11/34 (32%), Positives = 19/34 (56%), Gaps = 0/34 (0%)

Frame = +2

Query 461 ERAEPWCISSQAGCVVNCRFCSTGQPGLQPHTCR 562

E C+SSQ GC + C FC++ G++ + +

Sbjct 100 EYGNSVCVSSQVGCRMGCAFCASTIKGIKRNLTK 133

> AIL54054 23S methyltransferase, partial [Clostridioides difficile]

Length=216

Score = 28.1 bits (61), Expect = 0.083, Method: Compositional matrix adjust.

Identities = 11/20 (55%), Positives = 14/20 (70%), Gaps = 0/20 (0%)

Frame = +2

Query 488 SQAGCVVNCRFCSTGQPGLQ 547

SQ GC C+FC+TG GL+

Sbjct 1 SQCGCNFGCKFCATGDIGLK 20

> BAD68852 florfenicol resistance protein-like [Oryza sativa Japonica

Group]

Length=247

Score = 27.7 bits (60), Expect = 0.11, Method: Compositional matrix adjust.

Identities = 14/34 (41%), Positives = 21/34 (62%), Gaps = 1/34 (3%)

Frame = +2

Query 506 VNCRFCSTGQPGLQPHTCRPAKHVAQLWWACLLY 607

+NC+FC TG+ GL+ H A+ V Q +A L+

Sbjct 1 MNCQFCFTGRMGLRKHLS-TAEIVEQAVFARRLF 33

> AAT87417 Florfenicol resistance protein [Streptococcus pyogenes

MGAS10394]

Length=359

Score = 27.3 bits (59), Expect = 0.16, Method: Compositional matrix adjust.

Identities = 9/26 (35%), Positives = 16/26 (62%), Gaps = 0/26 (0%)

Frame = +2

Query 455 RKERAEPWCISSQAGCVVNCRFCSTG 532

R+ C+++Q GC + C FC++G

Sbjct 96 RQHYGHSVCVTTQVGCNIGCTFCASG 121

> ABO50235 23S rRNA m(2)A-2503 methyltransferase [Desulfotomaculum

reducens MI-1]

Length=350

Score = 26.6 bits (57), Expect = 0.36, Method: Compositional matrix adjust.

Identities = 10/22 (45%), Positives = 15/22 (68%), Gaps = 0/22 (0%)

Frame = +2

Query 479 CISSQAGCVVNCRFCSTGQPGL 544

C+S+Q GC + C FC++ GL

Sbjct 111 CVSTQVGCRMGCLFCASTINGL 132

> AAO35792 florfenicol resistance protein [Clostridium tetani E88]

Length=349

Score = 25.8 bits (55), Expect = 0.53, Method: Compositional matrix adjust.

Identities = 9/22 (41%), Positives = 15/22 (68%), Gaps = 0/22 (0%)

Frame = +2

Query 479 CISSQAGCVVNCRFCSTGQPGL 544

C+S+Q GC + C FC++ G+

Sbjct 106 CVSTQVGCKMGCTFCASTIGGI 127

Lambda K H a alpha

0.318 0.134 0.401 0.792 4.96

Gapped

Lambda K H a alpha sigma

0.267 0.0410 0.140 1.90 42.6 43.6

Effective search space used: 32770276

**Query=** NODE_43_length_664_cov_0.597765

Length=664

***** No hits found *****

Lambda K H a alpha

0.318 0.134 0.401 0.792 4.96

Gapped

Lambda K H a alpha sigma

0.267 0.0410 0.140 1.90 42.6 43.6

Effective search space used: 32131894

**Query=** NODE_44_length_662_cov_0.626866

Length=662

Score E

Sequences producing significant alignments: (Bits) Value

YP_004810315 putative multi-drug ABC transporter permease/ATPas... [56.6](file:///Users/JMBNew/Desktop/Articles%20en%20Cours/Article%20Diane/Bb_SR-11-14_output-blast_named.html#BL_ORD_ID:623) 4e-11

AEM66524 putative multi-drug ABC transporter permease/ATPase (p... [56.6](file:///Users/JMBNew/Desktop/Articles%20en%20Cours/Article%20Diane/Bb_SR-11-14_output-blast_named.html#BL_ORD_ID:74) 4e-11

YP_004800000 putative multi-drug ABC transporter permease/ATPas... [56.6](file:///Users/JMBNew/Desktop/Articles%20en%20Cours/Article%20Diane/Bb_SR-11-14_output-blast_named.html#BL_ORD_ID:619) 4e-11

AEM66534 putative multi-drug ABC transporter permease/ATPase (p... [56.6](file:///Users/JMBNew/Desktop/Articles%20en%20Cours/Article%20Diane/Bb_SR-11-14_output-blast_named.html#BL_ORD_ID:79) 4e-11

AQW34662 multidrug ABC transporter ATP-binding protein [Staphyl... [52.8](file:///Users/JMBNew/Desktop/Articles%20en%20Cours/Article%20Diane/Bb_SR-11-14_output-blast_named.html#BL_ORD_ID:257) 1e-09

AQW34613 multidrug ABC transporter ATP-binding protein [Staphyl... [52.8](file:///Users/JMBNew/Desktop/Articles%20en%20Cours/Article%20Diane/Bb_SR-11-14_output-blast_named.html#BL_ORD_ID:247) 1e-09

AQW34663 ABC transporter ATP-binding protein [Staphylococcus sc... [45.4](file:///Users/JMBNew/Desktop/Articles%20en%20Cours/Article%20Diane/Bb_SR-11-14_output-blast_named.html#BL_ORD_ID:258) 3e-07

AQW34614 ABC transporter ATP-binding protein [Staphylococcus sc... [45.4](file:///Users/JMBNew/Desktop/Articles%20en%20Cours/Article%20Diane/Bb_SR-11-14_output-blast_named.html#BL_ORD_ID:248) 3e-07

AKU20095 putative ABC transporter [Enterococcus faecium] [42.7](file:///Users/JMBNew/Desktop/Articles%20en%20Cours/Article%20Diane/Bb_SR-11-14_output-blast_named.html#BL_ORD_ID:204) 2e-06

ARA90579 phenicol and oxazolidinone resistance protein [Staphyl... [41.2](file:///Users/JMBNew/Desktop/Articles%20en%20Cours/Article%20Diane/Bb_SR-11-14_output-blast_named.html#BL_ORD_ID:288) 7e-06

AQW34744 phenicol and oxazolidinone resistance protein [Staphyl... [41.2](file:///Users/JMBNew/Desktop/Articles%20en%20Cours/Article%20Diane/Bb_SR-11-14_output-blast_named.html#BL_ORD_ID:278) 7e-06

AQW34730 phenicol and oxazolidinone resistance protein [Staphyl... [41.2](file:///Users/JMBNew/Desktop/Articles%20en%20Cours/Article%20Diane/Bb_SR-11-14_output-blast_named.html#BL_ORD_ID:274) 7e-06

AQW34661 phenicol and oxazolidinone resistance protein [Staphyl... [41.2](file:///Users/JMBNew/Desktop/Articles%20en%20Cours/Article%20Diane/Bb_SR-11-14_output-blast_named.html#BL_ORD_ID:256) 7e-06

AQW34601 phenicol and oxazolidinone resistance protein [Staphyl... [41.2](file:///Users/JMBNew/Desktop/Articles%20en%20Cours/Article%20Diane/Bb_SR-11-14_output-blast_named.html#BL_ORD_ID:245) 7e-06

AQW34667 ABC transporter ATP-binding protein (plasmid) [Staphyl... [41.2](file:///Users/JMBNew/Desktop/Articles%20en%20Cours/Article%20Diane/Bb_SR-11-14_output-blast_named.html#BL_ORD_ID:259) 7e-06

AQW34617 OptrA (plasmid) [Staphylococcus sciuri] [41.2](file:///Users/JMBNew/Desktop/Articles%20en%20Cours/Article%20Diane/Bb_SR-11-14_output-blast_named.html#BL_ORD_ID:249) 7e-06

AQW34567 ABC transporter ATP-binding protein [Staphylococcus sc... [41.2](file:///Users/JMBNew/Desktop/Articles%20en%20Cours/Article%20Diane/Bb_SR-11-14_output-blast_named.html#BL_ORD_ID:238) 7e-06

AQW34612 phenicol and oxazolidinone resistance protein [Staphyl... [41.2](file:///Users/JMBNew/Desktop/Articles%20en%20Cours/Article%20Diane/Bb_SR-11-14_output-blast_named.html#BL_ORD_ID:246) 7e-06

> YP_004810315 putative multi-drug ABC transporter permease/ATPase

(plasmid) [Riemerella anatipestifer]

Length=373

Score = 56.6 bits (135), Expect = 4e-11, Method: Compositional matrix adjust.

Identities = 26/58 (45%), Positives = 38/58 (66%), Gaps = 0/58 (0%)

Frame = -1

Query 392 RPGNSRDQLSGGQQQRVAIARSLCMTPKIMLFDEPTSALDPEMVKEVLDTVTTLAHGR 219

+ G S + +SGGQ+QR+ IAR++ P+ +LFDE TSALD E K + D + + GR

Sbjct 262 KIGASGNGISGGQKQRILIARAVYKNPQFILFDEATSALDAENEKIIHDNLQSFFKGR 319

> AEM66524 putative multi-drug ABC transporter permease/ATPase

(plasmid) [Riemerella anatipestifer]

Length=373

Score = 56.6 bits (135), Expect = 4e-11, Method: Compositional matrix adjust.

Identities = 26/58 (45%), Positives = 38/58 (66%), Gaps = 0/58 (0%)

Frame = -1

Query 392 RPGNSRDQLSGGQQQRVAIARSLCMTPKIMLFDEPTSALDPEMVKEVLDTVTTLAHGR 219

+ G S + +SGGQ+QR+ IAR++ P+ +LFDE TSALD E K + D + + GR

Sbjct 262 KIGASGNGISGGQKQRILIARAVYKNPQFILFDEATSALDAENEKIIHDNLQSFFKGR 319

> YP_004800000 putative multi-drug ABC transporter permease/ATPase

(plasmid) [Riemerella anatipestifer]

Length=373

Score = 56.6 bits (135), Expect = 4e-11, Method: Compositional matrix adjust.

Identities = 26/58 (45%), Positives = 38/58 (66%), Gaps = 0/58 (0%)

Frame = -1

Query 392 RPGNSRDQLSGGQQQRVAIARSLCMTPKIMLFDEPTSALDPEMVKEVLDTVTTLAHGR 219

+ G S + +SGGQ+QR+ IAR++ P+ +LFDE TSALD E K + D + + GR

Sbjct 262 KIGASGNGISGGQKQRILIARAVYKNPQFILFDEATSALDAENEKIIHDNLQSFFKGR 319

> AEM66534 putative multi-drug ABC transporter permease/ATPase

(plasmid) [Riemerella anatipestifer]

Length=373

Score = 56.6 bits (135), Expect = 4e-11, Method: Compositional matrix adjust.

Identities = 26/58 (45%), Positives = 38/58 (66%), Gaps = 0/58 (0%)

Frame = -1

Query 392 RPGNSRDQLSGGQQQRVAIARSLCMTPKIMLFDEPTSALDPEMVKEVLDTVTTLAHGR 219

+ G S + +SGGQ+QR+ IAR++ P+ +LFDE TSALD E K + D + + GR

Sbjct 262 KIGASGNGISGGQKQRILIARAVYKNPQFILFDEATSALDAENEKIIHDNLQSFFKGR 319

> AQW34662 multidrug ABC transporter ATP-binding protein [Staphylococcus

sciuri]

Length=579

Score = 52.8 bits (125), Expect = 1e-09, Method: Compositional matrix adjust.

Identities = 21/50 (42%), Positives = 35/50 (70%), Gaps = 0/50 (0%)

Frame = -1

Query 368 LSGGQQQRVAIARSLCMTPKIMLFDEPTSALDPEMVKEVLDTVTTLAHGR 219

+SGGQ+QR+AIAR+L M PKI++ D+ SA+D + K+++ + G+

Sbjct 473 ISGGQKQRIAIARALIMDPKILILDDALSAVDAKTEKKIIQNIQKYRQGK 522

> AQW34613 multidrug ABC transporter ATP-binding protein [Staphylococcus

sciuri]

Length=579

Score = 52.8 bits (125), Expect = 1e-09, Method: Compositional matrix adjust.

Identities = 21/50 (42%), Positives = 35/50 (70%), Gaps = 0/50 (0%)

Frame = -1

Query 368 LSGGQQQRVAIARSLCMTPKIMLFDEPTSALDPEMVKEVLDTVTTLAHGR 219

+SGGQ+QR+AIAR+L M PKI++ D+ SA+D + K+++ + G+

Sbjct 473 ISGGQKQRIAIARALIMDPKILILDDALSAVDAKTEKKIIQNIQKYRQGK 522

> AQW34663 ABC transporter ATP-binding protein [Staphylococcus

sciuri]

Length=582

Score = 45.4 bits (106), Expect = 3e-07, Method: Compositional matrix adjust.

Identities = 20/50 (40%), Positives = 33/50 (66%), Gaps = 0/50 (0%)

Frame = -1

Query 368 LSGGQQQRVAIARSLCMTPKIMLFDEPTSALDPEMVKEVLDTVTTLAHGR 219

LS GQ+Q ++ AR+L P+I++ DE TS++D E + + +T L+ GR

Sbjct 480 LSSGQRQIISFARALAFDPEILILDEATSSIDSETEEIIQKAMTVLSKGR 529

> AQW34614 ABC transporter ATP-binding protein [Staphylococcus

sciuri]

Length=582

Score = 45.4 bits (106), Expect = 3e-07, Method: Compositional matrix adjust.

Identities = 20/50 (40%), Positives = 33/50 (66%), Gaps = 0/50 (0%)

Frame = -1

Query 368 LSGGQQQRVAIARSLCMTPKIMLFDEPTSALDPEMVKEVLDTVTTLAHGR 219

LS GQ+Q ++ AR+L P+I++ DE TS++D E + + +T L+ GR

Sbjct 480 LSSGQRQIISFARALAFDPEILILDEATSSIDSETEEIIQKAMTVLSKGR 529

> AKU20095 putative ABC transporter [Enterococcus faecium]

Length=542

Score = 42.7 bits (99), Expect = 2e-06, Method: Compositional matrix adjust.

Identities = 18/33 (55%), Positives = 25/33 (76%), Gaps = 0/33 (0%)

Frame = -1

Query 371 QLSGGQQQRVAIARSLCMTPKIMLFDEPTSALD 273

+LSGGQ+ ++A AR L P+I+L DEPT+ LD

Sbjct 158 ELSGGQKSKMAFARLLYSKPEILLLDEPTNHLD 190

Score = 37.0 bits (84), Expect = 2e-04, Method: Compositional matrix adjust.

Identities = 16/37 (43%), Positives = 24/37 (65%), Gaps = 0/37 (0%)

Frame = -1

Query 368 LSGGQQQRVAIARSLCMTPKIMLFDEPTSALDPEMVK 258

LS G++ RVA+ + L +++ DEPT+ LDPE K

Sbjct 446 LSPGEKARVALCKILLQKANLLILDEPTNHLDPETQK 482

> ARA90579 phenicol and oxazolidinone resistance protein [Staphylococcus

sciuri]

Length=655

Score = 41.2 bits (95), Expect = 7e-06, Method: Compositional matrix adjust.

Identities = 19/58 (33%), Positives = 31/58 (53%), Gaps = 7/58 (12%)

Frame = -1

Query 371 QLSGGQQQRVAIARSLCMTPKIMLFDEPTSALDPEMV-------KEVLDTVTTLAHGR 219

+ SGGQ+ ++A + L P I+L DEPT+ LD E + + T+ ++H R

Sbjct 182 EFSGGQRTKIAFIKILLTKPDILLLDEPTNHLDIETIQWLESYLRSYKSTLVIISHDR 239

Score = 32.0 bits (71), Expect = 0.008, Method: Compositional matrix adjust.

Identities = 19/65 (29%), Positives = 36/65 (55%), Gaps = 10/65 (15%)

Frame = -1

Query 392 RPGNSRDQLSGGQQQRVAIARSLCMTPKIMLFDEPTSALD---PEMVKEVL----DTVTT 234

RP +S LSGG++ R+ + + L +++ DEPT+ +D E ++ +L T+

Sbjct 458 RPVSS---LSGGEKVRLTLCKLLYKRTNVLILDEPTNHMDIIGKENLENILCSYQGTIIF 514

Query 233 LAHGR 219

++H R

Sbjct 515 VSHDR 519

> AQW34744 phenicol and oxazolidinone resistance protein [Staphylococcus

sciuri]

Length=655

Score = 41.2 bits (95), Expect = 7e-06, Method: Compositional matrix adjust.

Identities = 19/58 (33%), Positives = 31/58 (53%), Gaps = 7/58 (12%)

Frame = -1

Query 371 QLSGGQQQRVAIARSLCMTPKIMLFDEPTSALDPEMV-------KEVLDTVTTLAHGR 219

+ SGGQ+ ++A + L P I+L DEPT+ LD E + + T+ ++H R

Sbjct 182 EFSGGQRTKIAFIKILLTKPDILLLDEPTNHLDIETIQWLESYLRSYKSTLVIISHDR 239

Score = 32.0 bits (71), Expect = 0.008, Method: Compositional matrix adjust.

Identities = 19/65 (29%), Positives = 36/65 (55%), Gaps = 10/65 (15%)

Frame = -1

Query 392 RPGNSRDQLSGGQQQRVAIARSLCMTPKIMLFDEPTSALD---PEMVKEVL----DTVTT 234

RP +S LSGG++ R+ + + L +++ DEPT+ +D E ++ +L T+

Sbjct 458 RPVSS---LSGGEKVRLTLCKLLYKRTNVLILDEPTNHMDIIGKENLENILCSYQGTIIF 514

Query 233 LAHGR 219

++H R

Sbjct 515 VSHDR 519

> AQW34730 phenicol and oxazolidinone resistance protein [Staphylococcus

sciuri]

Length=655

Score = 41.2 bits (95), Expect = 7e-06, Method: Compositional matrix adjust.

Identities = 19/58 (33%), Positives = 31/58 (53%), Gaps = 7/58 (12%)

Frame = -1

Query 371 QLSGGQQQRVAIARSLCMTPKIMLFDEPTSALDPEMV-------KEVLDTVTTLAHGR 219

+ SGGQ+ ++A + L P I+L DEPT+ LD E + + T+ ++H R

Sbjct 182 EFSGGQRTKIAFIKILLTKPDILLLDEPTNHLDIETIQWLESYLRSYKSTLVIISHDR 239

Score = 32.0 bits (71), Expect = 0.008, Method: Compositional matrix adjust.

Identities = 19/65 (29%), Positives = 36/65 (55%), Gaps = 10/65 (15%)

Frame = -1

Query 392 RPGNSRDQLSGGQQQRVAIARSLCMTPKIMLFDEPTSALD---PEMVKEVL----DTVTT 234

RP +S LSGG++ R+ + + L +++ DEPT+ +D E ++ +L T+

Sbjct 458 RPVSS---LSGGEKVRLTLCKLLYKRTNVLILDEPTNHMDIIGKENLENILCSYQGTIIF 514

Query 233 LAHGR 219

++H R

Sbjct 515 VSHDR 519

> AQW34661 phenicol and oxazolidinone resistance protein [Staphylococcus

sciuri]

Length=655

Score = 41.2 bits (95), Expect = 7e-06, Method: Compositional matrix adjust.

Identities = 19/58 (33%), Positives = 31/58 (53%), Gaps = 7/58 (12%)

Frame = -1

Query 371 QLSGGQQQRVAIARSLCMTPKIMLFDEPTSALDPEMV-------KEVLDTVTTLAHGR 219

+ SGGQ+ ++A + L P I+L DEPT+ LD E + + T+ ++H R

Sbjct 182 EFSGGQRTKIAFIKILLTKPDILLLDEPTNHLDIETIQWLESYLRSYKSTLVIISHDR 239

Score = 32.0 bits (71), Expect = 0.008, Method: Compositional matrix adjust.

Identities = 19/65 (29%), Positives = 36/65 (55%), Gaps = 10/65 (15%)

Frame = -1

Query 392 RPGNSRDQLSGGQQQRVAIARSLCMTPKIMLFDEPTSALD---PEMVKEVL----DTVTT 234

RP +S LSGG++ R+ + + L +++ DEPT+ +D E ++ +L T+

Sbjct 458 RPVSS---LSGGEKVRLTLCKLLYKRTNVLILDEPTNHMDIIGKENLENILCSYQGTIIF 514

Query 233 LAHGR 219

++H R

Sbjct 515 VSHDR 519

> AQW34601 phenicol and oxazolidinone resistance protein [Staphylococcus

sciuri]

Length=655

Score = 41.2 bits (95), Expect = 7e-06, Method: Compositional matrix adjust.

Identities = 19/58 (33%), Positives = 31/58 (53%), Gaps = 7/58 (12%)

Frame = -1

Query 371 QLSGGQQQRVAIARSLCMTPKIMLFDEPTSALDPEMV-------KEVLDTVTTLAHGR 219

+ SGGQ+ ++A + L P I+L DEPT+ LD E + + T+ ++H R

Sbjct 182 EFSGGQRTKIAFIKILLTKPDILLLDEPTNHLDIETIQWLESYLRSYKSTLVIISHDR 239

Score = 32.0 bits (71), Expect = 0.008, Method: Compositional matrix adjust.

Identities = 19/65 (29%), Positives = 36/65 (55%), Gaps = 10/65 (15%)

Frame = -1

Query 392 RPGNSRDQLSGGQQQRVAIARSLCMTPKIMLFDEPTSALD---PEMVKEVL----DTVTT 234

RP +S LSGG++ R+ + + L +++ DEPT+ +D E ++ +L T+

Sbjct 458 RPVSS---LSGGEKVRLTLCKLLYKRTNVLILDEPTNHMDIIGKENLENILCSYQGTIIF 514

Query 233 LAHGR 219

++H R

Sbjct 515 VSHDR 519

> AQW34667 ABC transporter ATP-binding protein (plasmid) [Staphylococcus

sciuri]

Length=655

Score = 41.2 bits (95), Expect = 7e-06, Method: Compositional matrix adjust.

Identities = 19/58 (33%), Positives = 31/58 (53%), Gaps = 7/58 (12%)

Frame = -1

Query 371 QLSGGQQQRVAIARSLCMTPKIMLFDEPTSALDPEMV-------KEVLDTVTTLAHGR 219

+ SGGQ+ ++A + L P I+L DEPT+ LD E + + T+ ++H R

Sbjct 182 EFSGGQRTKIAFIKILLTKPDILLLDEPTNHLDIETIQWLESYLRSYKSTLVIISHDR 239

Score = 32.0 bits (71), Expect = 0.008, Method: Compositional matrix adjust.

Identities = 19/65 (29%), Positives = 36/65 (55%), Gaps = 10/65 (15%)

Frame = -1

Query 392 RPGNSRDQLSGGQQQRVAIARSLCMTPKIMLFDEPTSALD---PEMVKEVL----DTVTT 234

RP +S LSGG++ R+ + + L +++ DEPT+ +D E ++ +L T+

Sbjct 458 RPVSS---LSGGEKVRLTLCKLLYKRTNVLILDEPTNHMDIIGKENLENILCSYQGTIIF 514

Query 233 LAHGR 219

++H R

Sbjct 515 VSHDR 519

> AQW34617 OptrA (plasmid) [Staphylococcus sciuri]

Length=655

Score = 41.2 bits (95), Expect = 7e-06, Method: Compositional matrix adjust.

Identities = 19/58 (33%), Positives = 31/58 (53%), Gaps = 7/58 (12%)

Frame = -1

Query 371 QLSGGQQQRVAIARSLCMTPKIMLFDEPTSALDPEMV-------KEVLDTVTTLAHGR 219

+ SGGQ+ ++A + L P I+L DEPT+ LD E + + T+ ++H R

Sbjct 182 EFSGGQRTKIAFIKILLTKPDILLLDEPTNHLDIETIQWLESYLRSYKSTLVIISHDR 239

Score = 32.0 bits (71), Expect = 0.008, Method: Compositional matrix adjust.

Identities = 19/65 (29%), Positives = 36/65 (55%), Gaps = 10/65 (15%)

Frame = -1

Query 392 RPGNSRDQLSGGQQQRVAIARSLCMTPKIMLFDEPTSALD---PEMVKEVL----DTVTT 234

RP +S LSGG++ R+ + + L +++ DEPT+ +D E ++ +L T+

Sbjct 458 RPVSS---LSGGEKVRLTLCKLLYKRTNVLILDEPTNHMDIIGKENLENILCSYQGTIIF 514

Query 233 LAHGR 219

++H R

Sbjct 515 VSHDR 519

> AQW34567 ABC transporter ATP-binding protein [Staphylococcus

sciuri]

Length=655

Score = 41.2 bits (95), Expect = 7e-06, Method: Compositional matrix adjust.

Identities = 19/58 (33%), Positives = 31/58 (53%), Gaps = 7/58 (12%)

Frame = -1

Query 371 QLSGGQQQRVAIARSLCMTPKIMLFDEPTSALDPEMV-------KEVLDTVTTLAHGR 219

+ SGGQ+ ++A + L P I+L DEPT+ LD E + + T+ ++H R

Sbjct 182 EFSGGQRTKIAFIKILLTKPDILLLDEPTNHLDIETIQWLESYLRSYKSTLVIISHDR 239

Score = 32.0 bits (71), Expect = 0.008, Method: Compositional matrix adjust.

Identities = 19/65 (29%), Positives = 36/65 (55%), Gaps = 10/65 (15%)

Frame = -1

Query 392 RPGNSRDQLSGGQQQRVAIARSLCMTPKIMLFDEPTSALD---PEMVKEVL----DTVTT 234

RP +S LSGG++ R+ + + L +++ DEPT+ +D E ++ +L T+

Sbjct 458 RPVSS---LSGGEKVRLTLCKLLYKRTNVLILDEPTNHMDIIGKENLENILCSYQGTIIF 514

Query 233 LAHGR 219

++H R

Sbjct 515 VSHDR 519

> AQW34612 phenicol and oxazolidinone resistance protein [Staphylococcus

sciuri]

Length=655

Score = 41.2 bits (95), Expect = 7e-06, Method: Compositional matrix adjust.

Identities = 19/58 (33%), Positives = 31/58 (53%), Gaps = 7/58 (12%)

Frame = -1

Query 371 QLSGGQQQRVAIARSLCMTPKIMLFDEPTSALDPEMV-------KEVLDTVTTLAHGR 219

+ SGGQ+ ++A + L P I+L DEPT+ LD E + + T+ ++H R

Sbjct 182 EFSGGQRTKIAFIKILLTKPDILLLDEPTNHLDIETIQWLESYLRSYKSTLVIISHDR 239

Score = 32.0 bits (71), Expect = 0.008, Method: Compositional matrix adjust.

Identities = 19/65 (29%), Positives = 36/65 (55%), Gaps = 10/65 (15%)

Frame = -1

Query 392 RPGNSRDQLSGGQQQRVAIARSLCMTPKIMLFDEPTSALD---PEMVKEVL----DTVTT 234

RP +S LSGG++ R+ + + L +++ DEPT+ +D E ++ +L T+

Sbjct 458 RPVSS---LSGGEKVRLTLCKLLYKRTNVLILDEPTNHMDIIGKENLENILCSYQGTIIF 514

Query 233 LAHGR 219

++H R

Sbjct 515 VSHDR 519

Lambda K H a alpha

0.318 0.134 0.401 0.792 4.96

Gapped

Lambda K H a alpha sigma

0.267 0.0410 0.140 1.90 42.6 43.6

Effective search space used: 31919100

**Query=** NODE_45_length_661_cov_3.24486

Length=661

***** No hits found *****

Lambda K H a alpha

0.318 0.134 0.401 0.792 4.96

Gapped

Lambda K H a alpha sigma

0.267 0.0410 0.140 1.90 42.6 43.6

Effective search space used: 31919100

**Query=** NODE_46_length_656_cov_0.427495

Length=656

***** No hits found *****

Lambda K H a alpha

0.318 0.134 0.401 0.792 4.96

Gapped

Lambda K H a alpha sigma

0.267 0.0410 0.140 1.90 42.6 43.6

Effective search space used: 31493512

**Query=** NODE_47_length_656_cov_0.276836

Length=656

***** No hits found *****

Lambda K H a alpha

0.318 0.134 0.401 0.792 4.96

Gapped

Lambda K H a alpha sigma

0.267 0.0410 0.140 1.90 42.6 43.6

Effective search space used: 31493512

**Query=** NODE_48_length_655_cov_0.625709

Length=655

***** No hits found *****

Lambda K H a alpha

0.318 0.134 0.401 0.792 4.96

Gapped

Lambda K H a alpha sigma

0.267 0.0410 0.140 1.90 42.6 43.6

Effective search space used: 31493512

**Query=** NODE_49_length_653_cov_0.612167

Length=653

***** No hits found *****

Lambda K H a alpha

0.318 0.134 0.401 0.792 4.96

Gapped

Lambda K H a alpha sigma

0.267 0.0410 0.140 1.90 42.6 43.6

Effective search space used: 31280718

**Query=** NODE_50_length_652_cov_0.641905

Length=652

***** No hits found *****

Lambda K H a alpha

0.318 0.134 0.401 0.792 4.96

Gapped

Lambda K H a alpha sigma

0.267 0.0410 0.140 1.90 42.6 43.6

Effective search space used: 31280718

**Query=** NODE_51_length_644_cov_0.574468

Length=644

***** No hits found *****

Lambda K H a alpha

0.318 0.134 0.401 0.792 4.96

Gapped

Lambda K H a alpha sigma

0.267 0.0410 0.140 1.90 42.6 43.6

Effective search space used: 30956920

**Query=** NODE_52_length_641_cov_0.54845

Length=641

***** No hits found *****

Lambda K H a alpha

0.318 0.134 0.401 0.792 4.96

Gapped

Lambda K H a alpha sigma

0.267 0.0410 0.140 1.90 42.6 43.6

Effective search space used: 30743424

**Query=** NODE_53_length_642_cov_0.500971

Length=642

***** No hits found *****

Lambda K H a alpha

0.318 0.134 0.401 0.792 4.96

Gapped

Lambda K H a alpha sigma

0.267 0.0410 0.140 1.90 42.6 43.6

Effective search space used: 30956920

**Query=** NODE_54_length_640_cov_0.610136

Length=640

Score E

Sequences producing significant alignments: (Bits) Value

CDT99921 putative FLORFENICOL EXPORTER [Vibrio diabolicus] [25.4](file:///Users/JMBNew/Desktop/Articles%20en%20Cours/Article%20Diane/Bb_SR-11-14_output-blast_named.html#BL_ORD_ID:394) 0.73

> CDT99921 putative FLORFENICOL EXPORTER [Vibrio diabolicus]

Length=397

Score = 25.4 bits (54), Expect = 0.73, Method: Compositional matrix adjust.

Identities = 12/29 (41%), Positives = 17/29 (59%), Gaps = 0/29 (0%)

Frame = +1

Query 346 RRTALGFGLPVLATNLGRAFCLPPANAPA 432

+ TAL F LP+ +++G AF L A A

Sbjct 299 QNTALSFMLPIFMSSVGFAFILGAAAGKA 327

Lambda K H a alpha

0.318 0.134 0.401 0.792 4.96

Gapped

Lambda K H a alpha sigma

0.267 0.0410 0.140 1.90 42.6 43.6

Effective search space used: 30743424

**Query=** NODE_55_length_639_cov_0.549708

Length=639

Score E

Sequences producing significant alignments: (Bits) Value

ARA90579 phenicol and oxazolidinone resistance protein [Staphyl... [26.9](file:///Users/JMBNew/Desktop/Articles%20en%20Cours/Article%20Diane/Bb_SR-11-14_output-blast_named.html#BL_ORD_ID:288) 0.24

AQW34744 phenicol and oxazolidinone resistance protein [Staphyl... [26.9](file:///Users/JMBNew/Desktop/Articles%20en%20Cours/Article%20Diane/Bb_SR-11-14_output-blast_named.html#BL_ORD_ID:278) 0.24

AQW34730 phenicol and oxazolidinone resistance protein [Staphyl... [26.9](file:///Users/JMBNew/Desktop/Articles%20en%20Cours/Article%20Diane/Bb_SR-11-14_output-blast_named.html#BL_ORD_ID:274) 0.24

AQW34667 ABC transporter ATP-binding protein (plasmid) [Staphyl... [26.9](file:///Users/JMBNew/Desktop/Articles%20en%20Cours/Article%20Diane/Bb_SR-11-14_output-blast_named.html#BL_ORD_ID:259) 0.24

AQW34661 phenicol and oxazolidinone resistance protein [Staphyl... [26.9](file:///Users/JMBNew/Desktop/Articles%20en%20Cours/Article%20Diane/Bb_SR-11-14_output-blast_named.html#BL_ORD_ID:256) 0.24

AQW34617 OptrA (plasmid) [Staphylococcus sciuri] [26.9](file:///Users/JMBNew/Desktop/Articles%20en%20Cours/Article%20Diane/Bb_SR-11-14_output-blast_named.html#BL_ORD_ID:249) 0.24

AQW34612 phenicol and oxazolidinone resistance protein [Staphyl... [26.9](file:///Users/JMBNew/Desktop/Articles%20en%20Cours/Article%20Diane/Bb_SR-11-14_output-blast_named.html#BL_ORD_ID:246) 0.24

AQW34601 phenicol and oxazolidinone resistance protein [Staphyl... [26.9](file:///Users/JMBNew/Desktop/Articles%20en%20Cours/Article%20Diane/Bb_SR-11-14_output-blast_named.html#BL_ORD_ID:245) 0.24

AQW34567 ABC transporter ATP-binding protein [Staphylococcus sc... [26.9](file:///Users/JMBNew/Desktop/Articles%20en%20Cours/Article%20Diane/Bb_SR-11-14_output-blast_named.html#BL_ORD_ID:238) 0.24

AKU20095 putative ABC transporter [Enterococcus faecium] [26.6](file:///Users/JMBNew/Desktop/Articles%20en%20Cours/Article%20Diane/Bb_SR-11-14_output-blast_named.html#BL_ORD_ID:204) 0.29

AQW34614 ABC transporter ATP-binding protein [Staphylococcus sc... [25.0](file:///Users/JMBNew/Desktop/Articles%20en%20Cours/Article%20Diane/Bb_SR-11-14_output-blast_named.html#BL_ORD_ID:248) 0.97

AQW34663 ABC transporter ATP-binding protein [Staphylococcus sc... [25.0](file:///Users/JMBNew/Desktop/Articles%20en%20Cours/Article%20Diane/Bb_SR-11-14_output-blast_named.html#BL_ORD_ID:258) 0.98

> ARA90579 phenicol and oxazolidinone resistance protein [Staphylococcus

sciuri]

Length=655

Score = 26.9 bits (58), Expect = 0.24, Method: Composition-based stats.

Identities = 9/24 (38%), Positives = 16/24 (67%), Gaps = 0/24 (0%)

Frame = -1

Query 366 GRRKLVALAACLMSRPSVAILDEP 295

G+R +A L+++P + +LDEP

Sbjct 186 GQRTKIAFIKILLTKPDILLLDEP 209

Score = 25.4 bits (54), Expect = 0.90, Method: Composition-based stats.

Identities = 16/35 (46%), Positives = 20/35 (57%), Gaps = 2/35 (6%)

Frame = -1

Query 396 VYRDPASCLMGRRKL-VALAACLMSRPSVAILDEP 295

V+R P S L G K+ + L L R +V ILDEP

Sbjct 456 VFR-PVSSLSGGEKVRLTLCKLLYKRTNVLILDEP 489

> AQW34744 phenicol and oxazolidinone resistance protein [Staphylococcus

sciuri]

Length=655

Score = 26.9 bits (58), Expect = 0.24, Method: Composition-based stats.

Identities = 9/24 (38%), Positives = 16/24 (67%), Gaps = 0/24 (0%)

Frame = -1

Query 366 GRRKLVALAACLMSRPSVAILDEP 295

G+R +A L+++P + +LDEP

Sbjct 186 GQRTKIAFIKILLTKPDILLLDEP 209

Score = 25.4 bits (54), Expect = 0.90, Method: Composition-based stats.

Identities = 16/35 (46%), Positives = 20/35 (57%), Gaps = 2/35 (6%)

Frame = -1

Query 396 VYRDPASCLMGRRKL-VALAACLMSRPSVAILDEP 295

V+R P S L G K+ + L L R +V ILDEP

Sbjct 456 VFR-PVSSLSGGEKVRLTLCKLLYKRTNVLILDEP 489

> AQW34730 phenicol and oxazolidinone resistance protein [Staphylococcus

sciuri]

Length=655

Score = 26.9 bits (58), Expect = 0.24, Method: Composition-based stats.

Identities = 9/24 (38%), Positives = 16/24 (67%), Gaps = 0/24 (0%)

Frame = -1

Query 366 GRRKLVALAACLMSRPSVAILDEP 295

G+R +A L+++P + +LDEP

Sbjct 186 GQRTKIAFIKILLTKPDILLLDEP 209

Score = 25.4 bits (54), Expect = 0.90, Method: Composition-based stats.

Identities = 16/35 (46%), Positives = 20/35 (57%), Gaps = 2/35 (6%)

Frame = -1

Query 396 VYRDPASCLMGRRKL-VALAACLMSRPSVAILDEP 295

V+R P S L G K+ + L L R +V ILDEP

Sbjct 456 VFR-PVSSLSGGEKVRLTLCKLLYKRTNVLILDEP 489

> AQW34667 ABC transporter ATP-binding protein (plasmid) [Staphylococcus

sciuri]

Length=655

Score = 26.9 bits (58), Expect = 0.24, Method: Composition-based stats.

Identities = 9/24 (38%), Positives = 16/24 (67%), Gaps = 0/24 (0%)

Frame = -1

Query 366 GRRKLVALAACLMSRPSVAILDEP 295

G+R +A L+++P + +LDEP

Sbjct 186 GQRTKIAFIKILLTKPDILLLDEP 209

Score = 25.4 bits (54), Expect = 0.90, Method: Composition-based stats.

Identities = 16/35 (46%), Positives = 20/35 (57%), Gaps = 2/35 (6%)

Frame = -1

Query 396 VYRDPASCLMGRRKL-VALAACLMSRPSVAILDEP 295

V+R P S L G K+ + L L R +V ILDEP

Sbjct 456 VFR-PVSSLSGGEKVRLTLCKLLYKRTNVLILDEP 489

> AQW34661 phenicol and oxazolidinone resistance protein [Staphylococcus

sciuri]

Length=655

Score = 26.9 bits (58), Expect = 0.24, Method: Composition-based stats.

Identities = 9/24 (38%), Positives = 16/24 (67%), Gaps = 0/24 (0%)

Frame = -1

Query 366 GRRKLVALAACLMSRPSVAILDEP 295

G+R +A L+++P + +LDEP

Sbjct 186 GQRTKIAFIKILLTKPDILLLDEP 209

Score = 25.4 bits (54), Expect = 0.90, Method: Composition-based stats.

Identities = 16/35 (46%), Positives = 20/35 (57%), Gaps = 2/35 (6%)

Frame = -1

Query 396 VYRDPASCLMGRRKL-VALAACLMSRPSVAILDEP 295

V+R P S L G K+ + L L R +V ILDEP

Sbjct 456 VFR-PVSSLSGGEKVRLTLCKLLYKRTNVLILDEP 489

> AQW34617 OptrA (plasmid) [Staphylococcus sciuri]

Length=655

Score = 26.9 bits (58), Expect = 0.24, Method: Composition-based stats.

Identities = 9/24 (38%), Positives = 16/24 (67%), Gaps = 0/24 (0%)

Frame = -1

Query 366 GRRKLVALAACLMSRPSVAILDEP 295

G+R +A L+++P + +LDEP

Sbjct 186 GQRTKIAFIKILLTKPDILLLDEP 209

Score = 25.4 bits (54), Expect = 0.90, Method: Composition-based stats.

Identities = 16/35 (46%), Positives = 20/35 (57%), Gaps = 2/35 (6%)

Frame = -1

Query 396 VYRDPASCLMGRRKL-VALAACLMSRPSVAILDEP 295

V+R P S L G K+ + L L R +V ILDEP

Sbjct 456 VFR-PVSSLSGGEKVRLTLCKLLYKRTNVLILDEP 489

> AQW34612 phenicol and oxazolidinone resistance protein [Staphylococcus

sciuri]

Length=655

Score = 26.9 bits (58), Expect = 0.24, Method: Composition-based stats.

Identities = 9/24 (38%), Positives = 16/24 (67%), Gaps = 0/24 (0%)

Frame = -1

Query 366 GRRKLVALAACLMSRPSVAILDEP 295

G+R +A L+++P + +LDEP

Sbjct 186 GQRTKIAFIKILLTKPDILLLDEP 209

Score = 25.4 bits (54), Expect = 0.90, Method: Composition-based stats.

Identities = 16/35 (46%), Positives = 20/35 (57%), Gaps = 2/35 (6%)

Frame = -1

Query 396 VYRDPASCLMGRRKL-VALAACLMSRPSVAILDEP 295

V+R P S L G K+ + L L R +V ILDEP

Sbjct 456 VFR-PVSSLSGGEKVRLTLCKLLYKRTNVLILDEP 489

> AQW34601 phenicol and oxazolidinone resistance protein [Staphylococcus

sciuri]

Length=655

Score = 26.9 bits (58), Expect = 0.24, Method: Composition-based stats.

Identities = 9/24 (38%), Positives = 16/24 (67%), Gaps = 0/24 (0%)

Frame = -1

Query 366 GRRKLVALAACLMSRPSVAILDEP 295

G+R +A L+++P + +LDEP

Sbjct 186 GQRTKIAFIKILLTKPDILLLDEP 209

Score = 25.4 bits (54), Expect = 0.90, Method: Composition-based stats.

Identities = 16/35 (46%), Positives = 20/35 (57%), Gaps = 2/35 (6%)

Frame = -1

Query 396 VYRDPASCLMGRRKL-VALAACLMSRPSVAILDEP 295

V+R P S L G K+ + L L R +V ILDEP

Sbjct 456 VFR-PVSSLSGGEKVRLTLCKLLYKRTNVLILDEP 489

> AQW34567 ABC transporter ATP-binding protein [Staphylococcus

sciuri]

Length=655

Score = 26.9 bits (58), Expect = 0.24, Method: Composition-based stats.

Identities = 9/24 (38%), Positives = 16/24 (67%), Gaps = 0/24 (0%)

Frame = -1

Query 366 GRRKLVALAACLMSRPSVAILDEP 295

G+R +A L+++P + +LDEP

Sbjct 186 GQRTKIAFIKILLTKPDILLLDEP 209

Score = 25.4 bits (54), Expect = 0.90, Method: Composition-based stats.

Identities = 16/35 (46%), Positives = 20/35 (57%), Gaps = 2/35 (6%)

Frame = -1

Query 396 VYRDPASCLMGRRKL-VALAACLMSRPSVAILDEP 295

V+R P S L G K+ + L L R +V ILDEP

Sbjct 456 VFR-PVSSLSGGEKVRLTLCKLLYKRTNVLILDEP 489

> AKU20095 putative ABC transporter [Enterococcus faecium]

Length=542

Score = 26.6 bits (57), Expect = 0.29, Method: Compositional matrix adjust.

Identities = 10/24 (42%), Positives = 16/24 (67%), Gaps = 0/24 (0%)

Frame = -1

Query 366 GRRKLVALAACLMSRPSVAILDEP 295

G++ +A A L S+P + +LDEP

Sbjct 162 GQKSKMAFARLLYSKPEILLLDEP 185

> AQW34614 ABC transporter ATP-binding protein [Staphylococcus

sciuri]

Length=582

Score = 25.0 bits (53), Expect = 0.97, Method: Compositional matrix adjust.

Identities = 9/23 (39%), Positives = 15/23 (65%), Gaps = 0/23 (0%)

Frame = -1

Query 366 GRRKLVALAACLMSRPSVAILDE 298

G+R++++ A L P + ILDE

Sbjct 483 GQRQIISFARALAFDPEILILDE 505

> AQW34663 ABC transporter ATP-binding protein [Staphylococcus

sciuri]

Length=582

Score = 25.0 bits (53), Expect = 0.98, Method: Compositional matrix adjust.

Identities = 9/23 (39%), Positives = 15/23 (65%), Gaps = 0/23 (0%)

Frame = -1

Query 366 GRRKLVALAACLMSRPSVAILDE 298

G+R++++ A L P + ILDE

Sbjct 483 GQRQIISFARALAFDPEILILDE 505

Lambda K H a alpha

0.318 0.134 0.401 0.792 4.96

Gapped

Lambda K H a alpha sigma

0.267 0.0410 0.140 1.90 42.6 43.6

Effective search space used: 30743424

**Query=** NODE_56_length_640_cov_0.590643

Length=640

***** No hits found *****

Lambda K H a alpha

0.318 0.134 0.401 0.792 4.96

Gapped

Lambda K H a alpha sigma

0.267 0.0410 0.140 1.90 42.6 43.6

Effective search space used: 30743424

**Query=** NODE_57_length_637_cov_0.64775

Length=637

***** No hits found *****

Lambda K H a alpha

0.318 0.134 0.401 0.792 4.96

Gapped

Lambda K H a alpha sigma

0.267 0.0410 0.140 1.90 42.6 43.6

Effective search space used: 30529928

**Query=** NODE_58_length_637_cov_2.49509

Length=637

***** No hits found *****

Lambda K H a alpha

0.318 0.134 0.401 0.792 4.96

Gapped

Lambda K H a alpha sigma

0.267 0.0410 0.140 1.90 42.6 43.6

Effective search space used: 30529928

**Query=** NODE_59_length_635_cov_0.167323

Length=635

***** No hits found *****

Lambda K H a alpha

0.318 0.134 0.401 0.792 4.96

Gapped

Lambda K H a alpha sigma

0.267 0.0410 0.140 1.90 42.6 43.6

Effective search space used: 30316432

**Query=** NODE_60_length_632_cov_0.952569

Length=632

Score E

Sequences producing significant alignments: (Bits) Value

AAM72416 florfenicol resistance protein, putative [Chlorobium t... [25.0](file:///Users/JMBNew/Desktop/Articles%20en%20Cours/Article%20Diane/Bb_SR-11-14_output-blast_named.html#BL_ORD_ID:8) 0.85

> AAM72416 florfenicol resistance protein, putative [Chlorobium

tepidum TLS]

Length=374

Score = 25.0 bits (53), Expect = 0.85, Method: Compositional matrix adjust.

Identities = 14/43 (33%), Positives = 18/43 (42%), Gaps = 0/43 (0%)

Frame = -1

Query 239 RSGRAASRAGQVHHAVFV*QTCRAVVASGTGKSENFILCSNWV 111

R G A RA Q+H ++ Q R S K L S W+

Sbjct 39 RLGEPAYRANQLHRWLYSNQALRFEEMSTLSKQLRQKLASEWI 81

Lambda K H a alpha

0.318 0.134 0.401 0.792 4.96

Gapped

Lambda K H a alpha sigma

0.267 0.0410 0.140 1.90 42.6 43.6

Effective search space used: 30102936

**Query=** NODE_61_length_633_cov_0.387352

Length=633

***** No hits found *****

Lambda K H a alpha

0.318 0.134 0.401 0.792 4.96

Gapped

Lambda K H a alpha sigma

0.267 0.0410 0.140 1.90 42.6 43.6

Effective search space used: 30316432

**Query=** NODE_62_length_629_cov_6.84661

Length=629

***** No hits found *****

Lambda K H a alpha

0.318 0.134 0.401 0.792 4.96

Gapped

Lambda K H a alpha sigma

0.267 0.0410 0.140 1.90 42.6 43.6

Effective search space used: 29889440

**Query=** NODE_63_length_628_cov_0.437126

Length=628

***** No hits found *****

Lambda K H a alpha

0.318 0.134 0.401 0.792 4.96

Gapped

Lambda K H a alpha sigma

0.267 0.0410 0.140 1.90 42.6 43.6

Effective search space used: 29889440

**Query=** NODE_64_length_623_cov_0.566

Length=623

***** No hits found *****

Lambda K H a alpha

0.318 0.134 0.401 0.792 4.96

Gapped

Lambda K H a alpha sigma

0.267 0.0410 0.140 1.90 42.6 43.6

Effective search space used: 29462448

**Query=** NODE_65_length_627_cov_1.04

Length=627

***** No hits found *****

Lambda K H a alpha

0.318 0.134 0.401 0.792 4.96

Gapped

Lambda K H a alpha sigma

0.267 0.0410 0.140 1.90 42.6 43.6

Effective search space used: 29889440

**Query=** NODE_66_length_623_cov_0.483871

Length=623

***** No hits found *****

Lambda K H a alpha

0.318 0.134 0.401 0.792 4.96

Gapped

Lambda K H a alpha sigma

0.267 0.0410 0.140 1.90 42.6 43.6

Effective search space used: 29462448

**Query=** NODE_67_length_619_cov_0.684848

Length=619

***** No hits found *****

Lambda K H a alpha

0.318 0.134 0.401 0.792 4.96

Gapped

Lambda K H a alpha sigma

0.267 0.0410 0.140 1.90 42.6 43.6

Effective search space used: 29248952

**Query=** NODE_68_length_621_cov_0.323232

Length=621

***** No hits found *****

Lambda K H a alpha

0.318 0.134 0.401 0.792 4.96

Gapped

Lambda K H a alpha sigma

0.267 0.0410 0.140 1.90 42.6 43.6

Effective search space used: 29462448

**Query=** NODE_69_length_616_cov_3.92229

Length=616

***** No hits found *****

Lambda K H a alpha

0.318 0.134 0.401 0.792 4.96

Gapped

Lambda K H a alpha sigma

0.267 0.0410 0.140 1.90 42.6 43.6

Effective search space used: 29035456

**Query=** NODE_70_length_615_cov_0.312883

Length=615

***** No hits found *****

Lambda K H a alpha

0.318 0.134 0.401 0.792 4.96

Gapped

Lambda K H a alpha sigma

0.267 0.0410 0.140 1.90 42.6 43.6

Effective search space used: 29035456

**Query=** NODE_71_length_614_cov_0.320329

Length=614

***** No hits found *****

Lambda K H a alpha

0.318 0.134 0.401 0.792 4.96

Gapped

Lambda K H a alpha sigma

0.267 0.0410 0.140 1.90 42.6 43.6

Effective search space used: 28821960

**Query=** NODE_72_length_613_cov_0.615226

Length=613

***** No hits found *****

Lambda K H a alpha

0.318 0.134 0.401 0.792 4.96

Gapped

Lambda K H a alpha sigma

0.267 0.0410 0.140 1.90 42.6 43.6

Effective search space used: 28821960

**Query=** NODE_73_length_613_cov_0.442387

Length=613

***** No hits found *****

Lambda K H a alpha

0.318 0.134 0.401 0.792 4.96

Gapped

Lambda K H a alpha sigma

0.267 0.0410 0.140 1.90 42.6 43.6

Effective search space used: 28821960

**Query=** NODE_74_length_612_cov_0.424742

Length=612

***** No hits found *****

Lambda K H a alpha

0.318 0.134 0.401 0.792 4.96

Gapped

Lambda K H a alpha sigma

0.267 0.0410 0.140 1.90 42.6 43.6

Effective search space used: 28821960

**Query=** NODE_75_length_607_cov_2.38462

Length=607

***** No hits found *****

Lambda K H a alpha

0.318 0.134 0.401 0.792 4.96

Gapped

Lambda K H a alpha sigma

0.267 0.0410 0.140 1.90 42.6 43.6

Effective search space used: 28394968

**Query=** NODE_76_length_607_cov_0.685417

Length=607

***** No hits found *****

Lambda K H a alpha

0.318 0.134 0.401 0.792 4.96

Gapped

Lambda K H a alpha sigma

0.267 0.0410 0.140 1.90 42.6 43.6

Effective search space used: 28394968

**Query=** NODE_77_length_605_cov_0.351464

Length=605

***** No hits found *****

Lambda K H a alpha

0.318 0.134 0.401 0.792 4.96

Gapped

Lambda K H a alpha sigma

0.267 0.0410 0.140 1.90 42.6 43.6

Effective search space used: 28181472

**Query=** NODE_78_length_605_cov_0.627615

Length=605

***** No hits found *****

Lambda K H a alpha

0.318 0.134 0.401 0.792 4.96

Gapped

Lambda K H a alpha sigma

0.267 0.0410 0.140 1.90 42.6 43.6

Effective search space used: 28181472

**Query=** NODE_79_length_605_cov_0.458159

Length=605

***** No hits found *****

Lambda K H a alpha

0.318 0.134 0.401 0.792 4.96

Gapped

Lambda K H a alpha sigma

0.267 0.0410 0.140 1.90 42.6 43.6

Effective search space used: 28181472

**Query=** NODE_80_length_601_cov_0.734177

Length=601

***** No hits found *****

Lambda K H a alpha

0.318 0.134 0.401 0.792 4.96

Gapped

Lambda K H a alpha sigma

0.267 0.0410 0.140 1.90 42.6 43.6

Effective search space used: 27967976

**Query=** NODE_81_length_595_cov_0.495763

Length=595

***** No hits found *****

Lambda K H a alpha

0.318 0.134 0.401 0.792 4.96

Gapped

Lambda K H a alpha sigma

0.267 0.0410 0.140 1.90 42.6 43.6

Effective search space used: 27540984

**Query=** NODE_82_length_599_cov_0.633475

Length=599

***** No hits found *****

Lambda K H a alpha

0.318 0.134 0.401 0.792 4.96

Gapped

Lambda K H a alpha sigma

0.267 0.0410 0.140 1.90 42.6 43.6

Effective search space used: 27754480

**Query=** NODE_83_length_596_cov_0.405117

Length=596

***** No hits found *****

Lambda K H a alpha

0.318 0.134 0.401 0.792 4.96

Gapped

Lambda K H a alpha sigma

0.267 0.0410 0.140 1.90 42.6 43.6

Effective search space used: 27540984

**Query=** NODE_84_length_594_cov_0.717949

Length=594

Score E

Sequences producing significant alignments: (Bits) Value

AAS81916 florfenicol resistance protein [Thermus thermophilus H... [25.4](file:///Users/JMBNew/Desktop/Articles%20en%20Cours/Article%20Diane/Bb_SR-11-14_output-blast_named.html#BL_ORD_ID:16) 0.65

> AAS81916 florfenicol resistance protein [Thermus thermophilus

HB27]

Length=355

Score = 25.4 bits (54), Expect = 0.65, Method: Compositional matrix adjust.

Identities = 11/30 (37%), Positives = 15/30 (50%), Gaps = 0/30 (0%)

Frame = -3

Query 256 VEILPFNPLPGPPDTRAPRVCGLCFKKNKK 167

V ++PFNP G P P+ L F + K

Sbjct 284 VNLIPFNPWEGAPVAGTPKAGVLAFAEELK 313

Lambda K H a alpha

0.318 0.134 0.401 0.792 4.96

Gapped

Lambda K H a alpha sigma

0.267 0.0410 0.140 1.90 42.6 43.6

Effective search space used: 27540984

**Query=** NODE_85_length_594_cov_0.508547

Length=594

***** No hits found *****

Lambda K H a alpha

0.318 0.134 0.401 0.792 4.96

Gapped

Lambda K H a alpha sigma

0.267 0.0410 0.140 1.90 42.6 43.6

Effective search space used: 27540984

**Query=** NODE_86_length_595_cov_0.40812

Length=595

***** No hits found *****

Lambda K H a alpha

0.318 0.134 0.401 0.792 4.96

Gapped

Lambda K H a alpha sigma

0.267 0.0410 0.140 1.90 42.6 43.6

Effective search space used: 27540984

**Query=** NODE_87_length_593_cov_0.487124

Length=593

***** No hits found *****

Lambda K H a alpha

0.318 0.134 0.401 0.792 4.96

Gapped

Lambda K H a alpha sigma

0.267 0.0410 0.140 1.90 42.6 43.6

Effective search space used: 27327488

**Query=** NODE_88_length_592_cov_0.729032

Length=592

***** No hits found *****

Lambda K H a alpha

0.318 0.134 0.401 0.792 4.96

Gapped

Lambda K H a alpha sigma

0.267 0.0410 0.140 1.90 42.6 43.6

Effective search space used: 27327488

**Query=** NODE_89_length_592_cov_0.634409

Length=592

***** No hits found *****

Lambda K H a alpha

0.318 0.134 0.401 0.792 4.96

Gapped

Lambda K H a alpha sigma

0.267 0.0410 0.140 1.90 42.6 43.6

Effective search space used: 27327488

**Query=** NODE_90_length_590_cov_0.7473

Length=590

***** No hits found *****

Lambda K H a alpha

0.318 0.134 0.401 0.792 4.96

Gapped

Lambda K H a alpha sigma

0.267 0.0410 0.140 1.90 42.6 43.6

Effective search space used: 27113992

**Query=** NODE_91_length_590_cov_1.0216

Length=590

***** No hits found *****

Lambda K H a alpha

0.318 0.134 0.401 0.792 4.96

Gapped

Lambda K H a alpha sigma

0.267 0.0410 0.140 1.90 42.6 43.6

Effective search space used: 27113992

**Query=** NODE_92_length_584_cov_0.515284

Length=584

***** No hits found *****

Lambda K H a alpha

0.318 0.134 0.401 0.792 4.96

Gapped

Lambda K H a alpha sigma

0.267 0.0410 0.140 1.90 42.6 43.6

Effective search space used: 26988948

**Query=** NODE_93_length_584_cov_0.7593

Length=584

***** No hits found *****

Lambda K H a alpha

0.318 0.134 0.401 0.792 4.96

Gapped

Lambda K H a alpha sigma

0.267 0.0410 0.140 1.90 42.6 43.6

Effective search space used: 26988948

**Query=** NODE_94_length_583_cov_0.760965

Length=583

***** No hits found *****

Lambda K H a alpha

0.318 0.134 0.401 0.792 4.96

Gapped

Lambda K H a alpha sigma

0.267 0.0410 0.140 1.90 42.6 43.6

Effective search space used: 26988948

**Query=** NODE_95_length_582_cov_0.564835

Length=582

***** No hits found *****

Lambda K H a alpha

0.318 0.134 0.401 0.792 4.96

Gapped

Lambda K H a alpha sigma

0.267 0.0410 0.140 1.90 42.6 43.6

Effective search space used: 26988948

**Query=** NODE_96_length_578_cov_0.595133

Length=578

***** No hits found *****

Lambda K H a alpha

0.318 0.134 0.401 0.792 4.96

Gapped

Lambda K H a alpha sigma

0.267 0.0410 0.140 1.90 42.6 43.6

Effective search space used: 26560552

**Query=** NODE_97_length_578_cov_0.696231

Length=578

***** No hits found *****

Lambda K H a alpha

0.318 0.134 0.401 0.792 4.96

Gapped

Lambda K H a alpha sigma

0.267 0.0410 0.140 1.90 42.6 43.6

Effective search space used: 26560552

**Query=** NODE_98_length_577_cov_0.764444

Length=577

***** No hits found *****

Lambda K H a alpha

0.318 0.134 0.401 0.792 4.96

Gapped

Lambda K H a alpha sigma

0.267 0.0410 0.140 1.90 42.6 43.6

Effective search space used: 26560552

**Query=** NODE_99_length_577_cov_0.408889

Length=577

***** No hits found *****

Lambda K H a alpha

0.318 0.134 0.401 0.792 4.96

Gapped

Lambda K H a alpha sigma

0.267 0.0410 0.140 1.90 42.6 43.6

Effective search space used: 26560552

**Query=** NODE_100_length_575_cov_0.754464

Length=575

***** No hits found *****

Lambda K H a alpha

0.318 0.134 0.401 0.792 4.96

Gapped

Lambda K H a alpha sigma

0.267 0.0410 0.140 1.90 42.6 43.6

Effective search space used: 26346354

**Query=** NODE_101_length_575_cov_0.399554

Length=575

***** No hits found *****

Lambda K H a alpha

0.318 0.134 0.401 0.792 4.96

Gapped

Lambda K H a alpha sigma

0.267 0.0410 0.140 1.90 42.6 43.6

Effective search space used: 26346354

**Query=** NODE_102_length_573_cov_0.607623

Length=573

Score E

Sequences producing significant alignments: (Bits) Value

RHE_RS08010_-_24301006_translation chloramphenicol efflux pump [25.0](file:///Users/JMBNew/Desktop/Articles%20en%20Cours/Article%20Diane/Bb_SR-11-14_output-blast_named.html#BL_ORD_ID:695) 0.83

> RHE_RS08010_-_24301006_translation chloramphenicol efflux pump

Length=397

Score = 25.0 bits (53), Expect = 0.83, Method: Compositional matrix adjust.

Identities = 14/32 (44%), Positives = 19/32 (59%), Gaps = 1/32 (3%)

Frame = +1

Query 319 MPRNIGGLEVGYRRQGLDDEADAIPAVQLGDE 414

MP + GG+E G RQ DDE D P + +G+

Sbjct 206 MPPH-GGIENGDDRQQADDEKDGSPPIVVGNH 236

Lambda K H a alpha

0.318 0.134 0.401 0.792 4.96

Gapped

Lambda K H a alpha sigma

0.267 0.0410 0.140 1.90 42.6 43.6

Effective search space used: 26346354

**Query=** NODE_103_length_572_cov_0.737079

Length=572

***** No hits found *****

Lambda K H a alpha

0.318 0.134 0.401 0.792 4.96

Gapped

Lambda K H a alpha sigma

0.267 0.0410 0.140 1.90 42.6 43.6

Effective search space used: 26132156

**Query=** NODE_104_length_571_cov_0.685393

Length=571

***** No hits found *****

Lambda K H a alpha

0.318 0.134 0.401 0.792 4.96

Gapped

Lambda K H a alpha sigma

0.267 0.0410 0.140 1.90 42.6 43.6

Effective search space used: 26132156

**Query=** NODE_105_length_572_cov_0.642697

Length=572

***** No hits found *****

Lambda K H a alpha

0.318 0.134 0.401 0.792 4.96

Gapped

Lambda K H a alpha sigma

0.267 0.0410 0.140 1.90 42.6 43.6

Effective search space used: 26132156

**Query=** NODE_106_length_570_cov_0.453933

Length=570

***** No hits found *****

Lambda K H a alpha

0.318 0.134 0.401 0.792 4.96

Gapped

Lambda K H a alpha sigma

0.267 0.0410 0.140 1.90 42.6 43.6

Effective search space used: 26132156

**Query=** NODE_107_length_571_cov_0.536036

Length=571

***** No hits found *****

Lambda K H a alpha

0.318 0.134 0.401 0.792 4.96

Gapped

Lambda K H a alpha sigma

0.267 0.0410 0.140 1.90 42.6 43.6

Effective search space used: 26132156

**Query=** NODE_108_length_570_cov_0.647856

Length=570

***** No hits found *****

Lambda K H a alpha

0.318 0.134 0.401 0.792 4.96

Gapped

Lambda K H a alpha sigma

0.267 0.0410 0.140 1.90 42.6 43.6

Effective search space used: 26132156

**Query=** NODE_109_length_570_cov_0.525959

Length=570

***** No hits found *****

Lambda K H a alpha

0.318 0.134 0.401 0.792 4.96

Gapped

Lambda K H a alpha sigma

0.267 0.0410 0.140 1.90 42.6 43.6

Effective search space used: 26132156

**Query=** NODE_110_length_567_cov_0.678733

Length=567

***** No hits found *****

Lambda K H a alpha

0.318 0.134 0.401 0.792 4.96

Gapped

Lambda K H a alpha sigma

0.267 0.0410 0.140 1.90 42.6 43.6

Effective search space used: 25917958

**Query=** NODE_111_length_568_cov_0.755656

Length=568

***** No hits found *****

Lambda K H a alpha

0.318 0.134 0.401 0.792 4.96

Gapped

Lambda K H a alpha sigma

0.267 0.0410 0.140 1.90 42.6 43.6

Effective search space used: 25917958

**Query=** NODE_112_length_569_cov_3.16063

Length=569

***** No hits found *****

Lambda K H a alpha

0.318 0.134 0.401 0.792 4.96

Gapped

Lambda K H a alpha sigma

0.267 0.0410 0.140 1.90 42.6 43.6

Effective search space used: 25917958

**Query=** NODE_113_length_568_cov_0.591837

Length=568

***** No hits found *****

Lambda K H a alpha

0.318 0.134 0.401 0.792 4.96

Gapped

Lambda K H a alpha sigma

0.267 0.0410 0.140 1.90 42.6 43.6

Effective search space used: 25917958

**Query=** NODE_114_length_566_cov_0.775

Length=566

***** No hits found *****

Lambda K H a alpha

0.318 0.134 0.401 0.792 4.96

Gapped

Lambda K H a alpha sigma

0.267 0.0410 0.140 1.90 42.6 43.6

Effective search space used: 25703760

**Query=** NODE_115_length_566_cov_0.785877

Length=566

***** No hits found *****

Lambda K H a alpha

0.318 0.134 0.401 0.792 4.96

Gapped

Lambda K H a alpha sigma

0.267 0.0410 0.140 1.90 42.6 43.6

Effective search space used: 25703760

**Query=** NODE_116_length_566_cov_0.767654

Length=566

***** No hits found *****

Lambda K H a alpha

0.318 0.134 0.401 0.792 4.96

Gapped

Lambda K H a alpha sigma

0.267 0.0410 0.140 1.90 42.6 43.6

Effective search space used: 25703760

**Query=** NODE_117_length_566_cov_0.76082

Length=566

***** No hits found *****

Lambda K H a alpha

0.318 0.134 0.401 0.792 4.96

Gapped

Lambda K H a alpha sigma

0.267 0.0410 0.140 1.90 42.6 43.6

Effective search space used: 25703760

**Query=** NODE_118_length_562_cov_0.506849

Length=562

***** No hits found *****

Lambda K H a alpha

0.318 0.134 0.401 0.792 4.96

Gapped

Lambda K H a alpha sigma

0.267 0.0410 0.140 1.90 42.6 43.6

Effective search space used: 25489562

**Query=** NODE_119_length_563_cov_0.540046

Length=563

***** No hits found *****

Lambda K H a alpha

0.318 0.134 0.401 0.792 4.96

Gapped

Lambda K H a alpha sigma

0.267 0.0410 0.140 1.90 42.6 43.6

Effective search space used: 25489562

**Query=** NODE_120_length_563_cov_0.763761

Length=563

***** No hits found *****

Lambda K H a alpha

0.318 0.134 0.401 0.792 4.96

Gapped

Lambda K H a alpha sigma

0.267 0.0410 0.140 1.90 42.6 43.6

Effective search space used: 25489562

**Query=** NODE_121_length_563_cov_0.759174

Length=563

***** No hits found *****

Lambda K H a alpha

0.317 0.133 0.431 0.792 4.96

Gapped

Lambda K H a alpha sigma

0.267 0.0410 0.140 1.90 42.6 43.6

Effective search space used: 25489562

**Query=** NODE_122_length_561_cov_0.548165

Length=561

Score E

Sequences producing significant alignments: (Bits) Value

OV14_RS04905_-_26221503_translation chloramphenicol efflux MFS ... [25.4](file:///Users/JMBNew/Desktop/Articles%20en%20Cours/Article%20Diane/Bb_SR-11-14_output-blast_named.html#BL_ORD_ID:692) 0.68

> OV14_RS04905_-_26221503_translation chloramphenicol efflux MFS

transporter

Length=382

Score = 25.4 bits (54), Expect = 0.68, Method: Compositional matrix adjust.

Identities = 12/29 (41%), Positives = 15/29 (52%), Gaps = 0/29 (0%)

Frame = -3

Query 319 DRAQGRRGGHQRPGMGHAGHALHPLCASL 233

DRA+GR G R GHA P+C +

Sbjct 244 DRAEGRHEGEHRAEEAVDGHAFGPVCIDI 272

Lambda K H a alpha

0.318 0.134 0.401 0.792 4.96

Gapped

Lambda K H a alpha sigma

0.267 0.0410 0.140 1.90 42.6 43.6

Effective search space used: 25489562

**Query=** NODE_123_length_562_cov_0.76092

Length=562

***** No hits found *****

Lambda K H a alpha

0.318 0.134 0.401 0.792 4.96

Gapped

Lambda K H a alpha sigma

0.267 0.0410 0.140 1.90 42.6 43.6

Effective search space used: 25489562

**Query=** NODE_124_length_562_cov_0.756322

Length=562

***** No hits found *****

Lambda K H a alpha

0.318 0.134 0.401 0.792 4.96

Gapped

Lambda K H a alpha sigma

0.267 0.0410 0.140 1.90 42.6 43.6

Effective search space used: 25489562

**Query=** NODE_125_length_562_cov_0.531034

Length=562

***** No hits found *****

Lambda K H a alpha

0.318 0.134 0.401 0.792 4.96

Gapped

Lambda K H a alpha sigma

0.267 0.0410 0.140 1.90 42.6 43.6

Effective search space used: 25489562

**Query=** NODE_126_length_562_cov_0.512644

Length=562

***** No hits found *****

Lambda K H a alpha

0.318 0.134 0.401 0.792 4.96

Gapped

Lambda K H a alpha sigma

0.267 0.0410 0.140 1.90 42.6 43.6

Effective search space used: 25489562

**Query=** NODE_127_length_561_cov_0.75576

Length=561

***** No hits found *****

Lambda K H a alpha

0.318 0.134 0.401 0.792 4.96

Gapped

Lambda K H a alpha sigma

0.267 0.0410 0.140 1.90 42.6 43.6

Effective search space used: 25489562

**Query=** NODE_128_length_560_cov_0.707373

Length=560

***** No hits found *****

Lambda K H a alpha

0.318 0.134 0.401 0.792 4.96

Gapped

Lambda K H a alpha sigma

0.267 0.0410 0.140 1.90 42.6 43.6

Effective search space used: 25275364

**Query=** NODE_129_length_560_cov_0.757506

Length=560

***** No hits found *****

Lambda K H a alpha

0.318 0.134 0.401 0.792 4.96

Gapped

Lambda K H a alpha sigma

0.267 0.0410 0.140 1.90 42.6 43.6

Effective search space used: 25275364

**Query=** NODE_130_length_560_cov_0.993072

Length=560

***** No hits found *****

Lambda K H a alpha

0.318 0.134 0.401 0.792 4.96

Gapped

Lambda K H a alpha sigma

0.267 0.0410 0.140 1.90 42.6 43.6

Effective search space used: 25275364

**Query=** NODE_131_length_559_cov_2.51501

Length=559

***** No hits found *****

Lambda K H a alpha

0.318 0.134 0.401 0.792 4.96

Gapped

Lambda K H a alpha sigma

0.267 0.0410 0.140 1.90 42.6 43.6

Effective search space used: 25275364

**Query=** NODE_132_length_559_cov_0.780093

Length=559

***** No hits found *****

Lambda K H a alpha

0.318 0.134 0.401 0.792 4.96

Gapped

Lambda K H a alpha sigma

0.267 0.0410 0.140 1.90 42.6 43.6

Effective search space used: 25275364

**Query=** NODE_133_length_559_cov_0.756944

Length=559

***** No hits found *****

Lambda K H a alpha

0.318 0.134 0.401 0.792 4.96

Gapped

Lambda K H a alpha sigma

0.267 0.0410 0.140 1.90 42.6 43.6

Effective search space used: 25275364

**Query=** NODE_134_length_559_cov_0.840278

Length=559

***** No hits found *****

Lambda K H a alpha

0.318 0.134 0.401 0.792 4.96

Gapped

Lambda K H a alpha sigma

0.267 0.0410 0.140 1.90 42.6 43.6

Effective search space used: 25275364

**Query=** NODE_135_length_558_cov_0.758701

Length=558

***** No hits found *****

Lambda K H a alpha

0.318 0.134 0.401 0.792 4.96

Gapped

Lambda K H a alpha sigma

0.267 0.0410 0.140 1.90 42.6 43.6

Effective search space used: 25275364

**Query=** NODE_136_length_555_cov_0.757576

Length=555

***** No hits found *****

Lambda K H a alpha

0.318 0.134 0.401 0.792 4.96

Gapped

Lambda K H a alpha sigma

0.267 0.0410 0.140 1.90 42.6 43.6

Effective search space used: 25061166

**Query=** NODE_137_length_556_cov_0.773893

Length=556

***** No hits found *****

Lambda K H a alpha

0.318 0.134 0.401 0.792 4.96

Gapped

Lambda K H a alpha sigma

0.267 0.0410 0.140 1.90 42.6 43.6

Effective search space used: 25061166

**Query=** NODE_138_length_556_cov_0.664336

Length=556

***** No hits found *****

Lambda K H a alpha

0.318 0.134 0.401 0.792 4.96

Gapped

Lambda K H a alpha sigma

0.267 0.0410 0.140 1.90 42.6 43.6

Effective search space used: 25061166

**Query=** NODE_139_length_555_cov_0.566434

Length=555

***** No hits found *****

Lambda K H a alpha

0.318 0.134 0.401 0.792 4.96

Gapped

Lambda K H a alpha sigma

0.267 0.0410 0.140 1.90 42.6 43.6

Effective search space used: 25061166

**Query=** NODE_140_length_555_cov_0.757009

Length=555

***** No hits found *****

Lambda K H a alpha

0.318 0.134 0.401 0.792 4.96

Gapped

Lambda K H a alpha sigma

0.267 0.0410 0.140 1.90 42.6 43.6

Effective search space used: 25061166

**Query=** NODE_141_length_555_cov_0.553738

Length=555

***** No hits found *****

Lambda K H a alpha

0.316 0.130 0.410 0.792 4.96

Gapped

Lambda K H a alpha sigma

0.267 0.0410 0.140 1.90 42.6 43.6

Effective search space used: 25061166

**Query=** NODE_142_length_554_cov_0.599532

Length=554

***** No hits found *****

Lambda K H a alpha

0.318 0.134 0.401 0.792 4.96

Gapped

Lambda K H a alpha sigma

0.267 0.0410 0.140 1.90 42.6 43.6

Effective search space used: 24846968

**Query=** NODE_143_length_554_cov_0.388759

Length=554

***** No hits found *****

Lambda K H a alpha

0.318 0.134 0.401 0.792 4.96

Gapped

Lambda K H a alpha sigma

0.267 0.0410 0.140 1.90 42.6 43.6

Effective search space used: 24846968

**Query=** NODE_144_length_554_cov_5.5644

Length=554

***** No hits found *****

Lambda K H a alpha

0.318 0.134 0.401 0.792 4.96

Gapped

Lambda K H a alpha sigma

0.267 0.0410 0.140 1.90 42.6 43.6

Effective search space used: 24846968

**Query=** NODE_145_length_554_cov_0.751756

Length=554

***** No hits found *****

Lambda K H a alpha

0.318 0.134 0.401 0.792 4.96

Gapped

Lambda K H a alpha sigma

0.267 0.0410 0.140 1.90 42.6 43.6

Effective search space used: 24846968

**Query=** NODE_146_length_551_cov_0.687059

Length=551

***** No hits found *****

Lambda K H a alpha

0.318 0.134 0.401 0.792 4.96

Gapped

Lambda K H a alpha sigma

0.267 0.0410 0.140 1.90 42.6 43.6

Effective search space used: 24632770

**Query=** NODE_147_length_552_cov_1.18588

Length=552

***** No hits found *****

Lambda K H a alpha

0.318 0.134 0.401 0.792 4.96

Gapped

Lambda K H a alpha sigma

0.267 0.0410 0.140 1.90 42.6 43.6

Effective search space used: 24846968

**Query=** NODE_148_length_552_cov_0.437647

Length=552

***** No hits found *****

Lambda K H a alpha

0.318 0.134 0.401 0.792 4.96

Gapped

Lambda K H a alpha sigma

0.267 0.0410 0.140 1.90 42.6 43.6

Effective search space used: 24846968

**Query=** NODE_149_length_550_cov_1.70047

Length=550

***** No hits found *****

Lambda K H a alpha

0.318 0.134 0.401 0.792 4.96

Gapped

Lambda K H a alpha sigma

0.267 0.0410 0.140 1.90 42.6 43.6

Effective search space used: 24632770

**Query=** NODE_150_length_550_cov_0.77305

Length=550

***** No hits found *****

Lambda K H a alpha

0.318 0.134 0.401 0.792 4.96

Gapped

Lambda K H a alpha sigma

0.267 0.0410 0.140 1.90 42.6 43.6

Effective search space used: 24632770

**Query=** NODE_151_length_547_cov_0.441805

Length=547

***** No hits found *****

Lambda K H a alpha

0.318 0.134 0.401 0.792 4.96

Gapped

Lambda K H a alpha sigma

0.267 0.0410 0.140 1.90 42.6 43.6

Effective search space used: 24418572

**Query=** NODE_152_length_544_cov_0.678571

Length=544

***** No hits found *****

Lambda K H a alpha

0.318 0.134 0.401 0.792 4.96

Gapped

Lambda K H a alpha sigma

0.267 0.0410 0.140 1.90 42.6 43.6

Effective search space used: 24204374

**Query=** NODE_153_length_547_cov_0.533333

Length=547

***** No hits found *****

Lambda K H a alpha

0.318 0.134 0.401 0.792 4.96

Gapped

Lambda K H a alpha sigma

0.267 0.0410 0.140 1.90 42.6 43.6

Effective search space used: 24418572

**Query=** NODE_154_length_546_cov_0.271429

Length=546

***** No hits found *****

Lambda K H a alpha

0.318 0.134 0.401 0.792 4.96

Gapped

Lambda K H a alpha sigma

0.267 0.0410 0.140 1.90 42.6 43.6

Effective search space used: 24418572

**Query=** NODE_155_length_546_cov_0.577566

Length=546

***** No hits found *****

Lambda K H a alpha

0.318 0.134 0.401 0.792 4.96

Gapped

Lambda K H a alpha sigma

0.267 0.0410 0.140 1.90 42.6 43.6

Effective search space used: 24418572

**Query=** NODE_156_length_546_cov_0.711217

Length=546

***** No hits found *****

Lambda K H a alpha

0.318 0.134 0.401 0.792 4.96

Gapped

Lambda K H a alpha sigma

0.267 0.0410 0.140 1.90 42.6 43.6

Effective search space used: 24418572

**Query=** NODE_157_length_546_cov_0.637232

Length=546

***** No hits found *****

Lambda K H a alpha

0.318 0.134 0.401 0.792 4.96

Gapped

Lambda K H a alpha sigma

0.267 0.0410 0.140 1.90 42.6 43.6

Effective search space used: 24418572

**Query=** NODE_158_length_545_cov_0.392344

Length=545

***** No hits found *****

Lambda K H a alpha

0.318 0.134 0.401 0.792 4.96

Gapped

Lambda K H a alpha sigma

0.267 0.0410 0.140 1.90 42.6 43.6

Effective search space used: 24204374

**Query=** NODE_159_length_545_cov_0.497608

Length=545

***** No hits found *****

Lambda K H a alpha

0.318 0.134 0.401 0.792 4.96

Gapped

Lambda K H a alpha sigma

0.267 0.0410 0.140 1.90 42.6 43.6

Effective search space used: 24204374

**Query=** NODE_160_length_544_cov_0.59952

Length=544

***** No hits found *****

Lambda K H a alpha

0.318 0.134 0.401 0.792 4.96

Gapped

Lambda K H a alpha sigma

0.267 0.0410 0.140 1.90 42.6 43.6

Effective search space used: 24204374

**Query=** NODE_161_length_544_cov_0.805755

Length=544

***** No hits found *****

Lambda K H a alpha

0.318 0.134 0.401 0.792 4.96

Gapped

Lambda K H a alpha sigma

0.267 0.0410 0.140 1.90 42.6 43.6

Effective search space used: 24204374

**Query=** NODE_162_length_543_cov_0.495192

Length=543

***** No hits found *****

Lambda K H a alpha

0.318 0.134 0.401 0.792 4.96

Gapped

Lambda K H a alpha sigma

0.267 0.0410 0.140 1.90 42.6 43.6

Effective search space used: 24204374

**Query=** NODE_163_length_543_cov_0.487981

Length=543

***** No hits found *****

Lambda K H a alpha

0.318 0.134 0.401 0.792 4.96

Gapped

Lambda K H a alpha sigma

0.267 0.0410 0.140 1.90 42.6 43.6

Effective search space used: 24204374

**Query=** NODE_164_length_542_cov_0.472289

Length=542

***** No hits found *****

Lambda K H a alpha

0.318 0.134 0.401 0.792 4.96

Gapped

Lambda K H a alpha sigma

0.267 0.0410 0.140 1.90 42.6 43.6

Effective search space used: 23990176

**Query=** NODE_165_length_540_cov_0.777778

Length=540

***** No hits found *****

Lambda K H a alpha

0.318 0.134 0.401 0.792 4.96

Gapped

Lambda K H a alpha sigma

0.267 0.0410 0.140 1.90 42.6 43.6

Effective search space used: 23990176

**Query=** NODE_166_length_539_cov_0.868932

Length=539

***** No hits found *****

Lambda K H a alpha

0.318 0.134 0.401 0.792 4.96

Gapped

Lambda K H a alpha sigma

0.267 0.0410 0.140 1.90 42.6 43.6

Effective search space used: 23775978

**Query=** NODE_167_length_537_cov_0.827251

Length=537

***** No hits found *****

Lambda K H a alpha

0.318 0.134 0.401 0.792 4.96

Gapped

Lambda K H a alpha sigma

0.267 0.0410 0.140 1.90 42.6 43.6

Effective search space used: 23775978

**Query=** NODE_168_length_538_cov_0.350365

Length=538

***** No hits found *****

Lambda K H a alpha

0.318 0.134 0.401 0.792 4.96

Gapped

Lambda K H a alpha sigma

0.267 0.0410 0.140 1.90 42.6 43.6

Effective search space used: 23775978

**Query=** NODE_169_length_535_cov_0.816626

Length=535

***** No hits found *****

Lambda K H a alpha

0.318 0.134 0.401 0.792 4.96

Gapped

Lambda K H a alpha sigma

0.267 0.0410 0.140 1.90 42.6 43.6

Effective search space used: 23561780

**Query=** NODE_170_length_533_cov_0.736453

Length=533

***** No hits found *****

Lambda K H a alpha

0.318 0.134 0.401 0.792 4.96

Gapped

Lambda K H a alpha sigma

0.267 0.0410 0.140 1.90 42.6 43.6

Effective search space used: 23347582

**Query=** NODE_171_length_533_cov_0.583744

Length=533

***** No hits found *****

Lambda K H a alpha

0.318 0.134 0.401 0.792 4.96

Gapped

Lambda K H a alpha sigma

0.267 0.0410 0.140 1.90 42.6 43.6

Effective search space used: 23347582

**Query=** NODE_172_length_528_cov_0.435644

Length=528

***** No hits found *****

Lambda K H a alpha

0.318 0.134 0.401 0.792 4.96

Gapped

Lambda K H a alpha sigma

0.267 0.0410 0.140 1.90 42.6 43.6

Effective search space used: 23424100

**Query=** NODE_173_length_529_cov_0.655087

Length=529

***** No hits found *****

Lambda K H a alpha

0.318 0.134 0.401 0.792 4.96

Gapped

Lambda K H a alpha sigma

0.267 0.0410 0.140 1.90 42.6 43.6

Effective search space used: 23424100

**Query=** NODE_174_length_530_cov_0.590571

Length=530

***** No hits found *****

Lambda K H a alpha

0.318 0.134 0.401 0.792 4.96

Gapped

Lambda K H a alpha sigma

0.267 0.0410 0.140 1.90 42.6 43.6

Effective search space used: 23424100

**Query=** NODE_175_length_529_cov_0.230769

Length=529

***** No hits found *****

Lambda K H a alpha

0.318 0.134 0.401 0.792 4.96

Gapped

Lambda K H a alpha sigma

0.267 0.0410 0.140 1.90 42.6 43.6

Effective search space used: 23424100

**Query=** NODE_176_length_529_cov_0.246269

Length=529

***** No hits found *****

Lambda K H a alpha

0.318 0.134 0.401 0.792 4.96

Gapped

Lambda K H a alpha sigma

0.267 0.0410 0.140 1.90 42.6 43.6

Effective search space used: 23424100

**Query=** NODE_177_length_528_cov_0.63591

Length=528

***** No hits found *****

Lambda K H a alpha

0.318 0.134 0.401 0.792 4.96

Gapped

Lambda K H a alpha sigma

0.267 0.0410 0.140 1.90 42.6 43.6

Effective search space used: 23424100

**Query=** NODE_178_length_528_cov_0.86783

Length=528

***** No hits found *****

Lambda K H a alpha

0.318 0.134 0.401 0.792 4.96

Gapped

Lambda K H a alpha sigma

0.267 0.0410 0.140 1.90 42.6 43.6

Effective search space used: 23424100

**Query=** NODE_179_length_528_cov_0.705736

Length=528

***** No hits found *****

Lambda K H a alpha

0.318 0.134 0.401 0.792 4.96

Gapped

Lambda K H a alpha sigma

0.267 0.0410 0.140 1.90 42.6 43.6

Effective search space used: 23424100

**Query=** NODE_180_length_528_cov_0.738155

Length=528

***** No hits found *****

Lambda K H a alpha

0.318 0.134 0.401 0.792 4.96

Gapped

Lambda K H a alpha sigma

0.267 0.0410 0.140 1.90 42.6 43.6

Effective search space used: 23424100

**Query=** NODE_181_length_527_cov_0.763092

Length=527

***** No hits found *****

Lambda K H a alpha

0.318 0.134 0.401 0.792 4.96

Gapped

Lambda K H a alpha sigma

0.267 0.0410 0.140 1.90 42.6 43.6

Effective search space used: 23209200

**Query=** NODE_182_length_527_cov_0.8125

Length=527

***** No hits found *****

Lambda K H a alpha

0.318 0.134 0.401 0.792 4.96

Gapped

Lambda K H a alpha sigma

0.267 0.0410 0.140 1.90 42.6 43.6

Effective search space used: 23209200

**Query=** NODE_183_length_525_cov_0.699248

Length=525

***** No hits found *****

Lambda K H a alpha

0.318 0.134 0.401 0.792 4.96

Gapped

Lambda K H a alpha sigma

0.267 0.0410 0.140 1.90 42.6 43.6

Effective search space used: 23209200

**Query=** NODE_184_length_524_cov_0.684211

Length=524

***** No hits found *****

Lambda K H a alpha

0.318 0.134 0.401 0.792 4.96

Gapped

Lambda K H a alpha sigma

0.267 0.0410 0.140 1.90 42.6 43.6

Effective search space used: 22994300

**Query=** NODE_185_length_525_cov_0.561404

Length=525

***** No hits found *****

Lambda K H a alpha

0.318 0.134 0.401 0.792 4.96

Gapped

Lambda K H a alpha sigma

0.267 0.0410 0.140 1.90 42.6 43.6

Effective search space used: 23209200

**Query=** NODE_186_length_526_cov_0.606516

Length=526

***** No hits found *****

Lambda K H a alpha

0.318 0.134 0.401 0.792 4.96

Gapped

Lambda K H a alpha sigma

0.267 0.0410 0.140 1.90 42.6 43.6

Effective search space used: 23209200

**Query=** NODE_187_length_523_cov_1.32161

Length=523

***** No hits found *****

Lambda K H a alpha

0.317 0.139 0.455 0.792 4.96

Gapped

Lambda K H a alpha sigma

0.267 0.0410 0.140 1.90 42.6 43.6

Effective search space used: 22994300

**Query=** NODE_188_length_522_cov_0.78392

Length=522

Score E

Sequences producing significant alignments: (Bits) Value

EKU30462 putative chloramphenicol and florfenicol resistance pr... [25.0](file:///Users/JMBNew/Desktop/Articles%20en%20Cours/Article%20Diane/Bb_SR-11-14_output-blast_named.html#BL_ORD_ID:449) 0.79

> EKU30462 putative chloramphenicol and florfenicol resistance

protein (CmlA) [Alcaligenes sp. HPC1271]

Length=381

Score = 25.0 bits (53), Expect = 0.79, Method: Compositional matrix adjust.

Identities = 10/21 (48%), Positives = 15/21 (71%), Gaps = 0/21 (0%)

Frame = +2

Query 164 VARRGGWAAMMVRMMAIGLAA 226

+AR GGW A+ + + A+GL A

Sbjct 144 IARMGGWQAIFLSLAAMGLIA 164

Lambda K H a alpha

0.318 0.134 0.401 0.792 4.96

Gapped

Lambda K H a alpha sigma

0.267 0.0410 0.140 1.90 42.6 43.6

Effective search space used: 22994300

**Query=** NODE_189_length_525_cov_0.748744

Length=525

Score E

Sequences producing significant alignments: (Bits) Value

MRAD2831_RS42670_-_6138192_translation chloramphenicol resistan... [27.3](file:///Users/JMBNew/Desktop/Articles%20en%20Cours/Article%20Diane/Bb_SR-11-14_output-blast_named.html#BL_ORD_ID:688) 0.10

> MRAD2831_RS42670_-_6138192_translation chloramphenicol resistance

permease RarD

Length=299

Score = 27.3 bits (59), Expect = 0.10, Method: Compositional matrix adjust.

Identities = 13/27 (48%), Positives = 14/27 (52%), Gaps = 0/27 (0%)

Frame = -2

Query 263 PLRAPAWRRTGARHPSLSRDGGAPPGV 183

PLR R GA HP + RD AP V

Sbjct 264 PLRQDVVRGVGAEHPEMHRDQEAPAQV 290

Lambda K H a alpha

0.318 0.134 0.401 0.792 4.96

Gapped

Lambda K H a alpha sigma

0.267 0.0410 0.140 1.90 42.6 43.6

Effective search space used: 23209200

**Query=** NODE_190_length_525_cov_0.733668

Length=525

***** No hits found *****

Lambda K H a alpha

0.318 0.134 0.401 0.792 4.96

Gapped

Lambda K H a alpha sigma

0.267 0.0410 0.140 1.90 42.6 43.6

Effective search space used: 23209200

**Query=** NODE_191_length_523_cov_0.438287

Length=523

***** No hits found *****

Lambda K H a alpha

0.318 0.134 0.401 0.792 4.96

Gapped

Lambda K H a alpha sigma

0.267 0.0410 0.140 1.90 42.6 43.6

Effective search space used: 22994300

**Query=** NODE_192_length_522_cov_0.755051

Length=522

***** No hits found *****

Lambda K H a alpha

0.318 0.134 0.401 0.792 4.96

Gapped

Lambda K H a alpha sigma

0.267 0.0410 0.140 1.90 42.6 43.6

Effective search space used: 22994300

**Query=** NODE_193_length_523_cov_0.467172

Length=523

***** No hits found *****

Lambda K H a alpha

0.316 0.132 0.401 0.792 4.96

Gapped

Lambda K H a alpha sigma

0.267 0.0410 0.140 1.90 42.6 43.6

Effective search space used: 22994300

**Query=** NODE_194_length_523_cov_0.817259

Length=523

***** No hits found *****

Lambda K H a alpha

0.318 0.134 0.401 0.792 4.96

Gapped

Lambda K H a alpha sigma

0.267 0.0410 0.140 1.90 42.6 43.6

Effective search space used: 22994300

**Query=** NODE_195_length_521_cov_0.345178

Length=521

***** No hits found *****

Lambda K H a alpha

0.318 0.134 0.401 0.792 4.96

Gapped

Lambda K H a alpha sigma

0.267 0.0410 0.140 1.90 42.6 43.6

Effective search space used: 22779400

**Query=** NODE_196_length_520_cov_0.801527

Length=520

***** No hits found *****

Lambda K H a alpha

0.318 0.134 0.401 0.792 4.96

Gapped

Lambda K H a alpha sigma

0.267 0.0410 0.140 1.90 42.6 43.6

Effective search space used: 22779400

**Query=** NODE_197_length_519_cov_0.431122

Length=519

***** No hits found *****

Lambda K H a alpha

0.318 0.134 0.401 0.792 4.96

Gapped

Lambda K H a alpha sigma

0.267 0.0410 0.140 1.90 42.6 43.6

Effective search space used: 22779400

**Query=** NODE_198_length_515_cov_0.741026

Length=515

***** No hits found *****

Lambda K H a alpha

0.318 0.134 0.401 0.792 4.96

Gapped

Lambda K H a alpha sigma

0.267 0.0410 0.140 1.90 42.6 43.6

Effective search space used: 22349600

**Query=** NODE_199_length_516_cov_0.632391

Length=516

***** No hits found *****

Lambda K H a alpha

0.318 0.134 0.401 0.792 4.96

Gapped

Lambda K H a alpha sigma

0.267 0.0410 0.140 1.90 42.6 43.6

Effective search space used: 22564500

**Query=** NODE_200_length_516_cov_0.40617

Length=516

***** No hits found *****

Lambda K H a alpha

0.318 0.134 0.401 0.792 4.96

Gapped

Lambda K H a alpha sigma

0.267 0.0410 0.140 1.90 42.6 43.6

Effective search space used: 22564500

**Query=** NODE_201_length_514_cov_0.51671

Length=514

***** No hits found *****

Lambda K H a alpha

0.318 0.134 0.401 0.792 4.96

Gapped

Lambda K H a alpha sigma

0.267 0.0410 0.140 1.90 42.6 43.6

Effective search space used: 22349600

**Query=** NODE_202_length_511_cov_0.4677

Length=511

***** No hits found *****

Lambda K H a alpha

0.318 0.134 0.401 0.792 4.96

Gapped

Lambda K H a alpha sigma

0.267 0.0410 0.140 1.90 42.6 43.6

Effective search space used: 22134700

**Query=** NODE_203_length_514_cov_0.702842

Length=514

***** No hits found *****

Lambda K H a alpha

0.318 0.134 0.401 0.792 4.96

Gapped

Lambda K H a alpha sigma

0.267 0.0410 0.140 1.90 42.6 43.6

Effective search space used: 22349600

**Query=** NODE_204_length_510_cov_0.810881

Length=510

***** No hits found *****

Lambda K H a alpha

0.318 0.134 0.401 0.792 4.96

Gapped

Lambda K H a alpha sigma

0.267 0.0410 0.140 1.90 42.6 43.6

Effective search space used: 22134700

**Query=** NODE_205_length_512_cov_0.406736

Length=512

***** No hits found *****

Lambda K H a alpha

0.318 0.134 0.401 0.792 4.96

Gapped

Lambda K H a alpha sigma

0.267 0.0410 0.140 1.90 42.6 43.6

Effective search space used: 22134700

**Query=** NODE_206_length_512_cov_0.644156

Length=512

***** No hits found *****

Lambda K H a alpha

0.318 0.134 0.401 0.792 4.96

Gapped

Lambda K H a alpha sigma

0.267 0.0410 0.140 1.90 42.6 43.6

Effective search space used: 22134700

**Query=** NODE_207_length_512_cov_0.587013

Length=512

***** No hits found *****

Lambda K H a alpha

0.318 0.134 0.401 0.792 4.96

Gapped

Lambda K H a alpha sigma

0.267 0.0410 0.140 1.90 42.6 43.6

Effective search space used: 22134700

**Query=** NODE_208_length_509_cov_0.736979

Length=509

***** No hits found *****

Lambda K H a alpha

0.318 0.134 0.401 0.792 4.96

Gapped

Lambda K H a alpha sigma

0.267 0.0410 0.140 1.90 42.6 43.6

Effective search space used: 21919800

**Query=** NODE_209_length_509_cov_0.62141

Length=509

***** No hits found *****

Lambda K H a alpha

0.318 0.134 0.401 0.792 4.96

Gapped

Lambda K H a alpha sigma

0.267 0.0410 0.140 1.90 42.6 43.6

Effective search space used: 21919800

**Query=** NODE_210_length_506_cov_1.02362

Length=506

***** No hits found *****

Lambda K H a alpha

0.318 0.134 0.401 0.792 4.96

Gapped

Lambda K H a alpha sigma

0.267 0.0410 0.140 1.90 42.6 43.6

Effective search space used: 21704900

**Query=** NODE_211_length_506_cov_0.514436

Length=506

***** No hits found *****

Lambda K H a alpha

0.318 0.134 0.401 0.792 4.96

Gapped

Lambda K H a alpha sigma

0.267 0.0410 0.140 1.90 42.6 43.6

Effective search space used: 21704900

**Query=** NODE_212_length_508_cov_0.433071

Length=508

***** No hits found *****

Lambda K H a alpha

0.318 0.134 0.401 0.792 4.96

Gapped

Lambda K H a alpha sigma

0.267 0.0410 0.140 1.90 42.6 43.6

Effective search space used: 21919800

**Query=** NODE_213_length_507_cov_0.444737

Length=507

***** No hits found *****

Lambda K H a alpha

0.318 0.134 0.401 0.792 4.96

Gapped

Lambda K H a alpha sigma

0.267 0.0410 0.140 1.90 42.6 43.6

Effective search space used: 21919800

**Query=** NODE_214_length_507_cov_1.15

Length=507

***** No hits found *****

Lambda K H a alpha

0.318 0.134 0.401 0.792 4.96

Gapped

Lambda K H a alpha sigma

0.267 0.0410 0.140 1.90 42.6 43.6

Effective search space used: 21919800

**Query=** NODE_215_length_507_cov_0.726316

Length=507

***** No hits found *****

Lambda K H a alpha

0.318 0.134 0.401 0.792 4.96

Gapped

Lambda K H a alpha sigma

0.267 0.0410 0.140 1.90 42.6 43.6

Effective search space used: 21919800

**Query=** NODE_216_length_505_cov_0.931579

Length=505

***** No hits found *****

Lambda K H a alpha

0.314 0.132 0.402 0.792 4.96

Gapped

Lambda K H a alpha sigma

0.267 0.0410 0.140 1.90 42.6 43.6

Effective search space used: 21704900

**Query=** NODE_217_length_508_cov_0.5

Length=508

Score E

Sequences producing significant alignments: (Bits) Value

XP_002487874 florfenicol exporter, putative [Talaromyces stipit... [25.8](file:///Users/JMBNew/Desktop/Articles%20en%20Cours/Article%20Diane/Bb_SR-11-14_output-blast_named.html#BL_ORD_ID:585) 0.46

EED12220 florfenicol exporter, putative [Talaromyces stipitatus... [25.8](file:///Users/JMBNew/Desktop/Articles%20en%20Cours/Article%20Diane/Bb_SR-11-14_output-blast_named.html#BL_ORD_ID:422) 0.46

> XP_002487874 florfenicol exporter, putative [Talaromyces stipitatus

ATCC 10500]

Length=988

Score = 25.8 bits (55), Expect = 0.46, Method: Composition-based stats.

Identities = 18/64 (28%), Positives = 31/64 (48%), Gaps = 7/64 (11%)

Frame = +1

Query 19 EKAAQAYTGAGGDAVVEGLPHRRRQAGIGLGGFLVEGVLAGGAAIQPAVQATLAGLASET 198

+ A + T A AVV L R+++ G ++ G+ A G+++ PA+ + GL

Sbjct 621 QSAGSSGTTALSSAVVSDLATRQQR-----GSYI--GLAALGSSLGPALGPIIGGLLDHF 673

Query 199 AAWR 210

WR

Sbjct 674 LGWR 677

> EED12220 florfenicol exporter, putative [Talaromyces stipitatus

ATCC 10500]

Length=988

Score = 25.8 bits (55), Expect = 0.46, Method: Composition-based stats.

Identities = 18/64 (28%), Positives = 31/64 (48%), Gaps = 7/64 (11%)

Frame = +1

Query 19 EKAAQAYTGAGGDAVVEGLPHRRRQAGIGLGGFLVEGVLAGGAAIQPAVQATLAGLASET 198

+ A + T A AVV L R+++ G ++ G+ A G+++ PA+ + GL

Sbjct 621 QSAGSSGTTALSSAVVSDLATRQQR-----GSYI--GLAALGSSLGPALGPIIGGLLDHF 673

Query 199 AAWR 210

WR

Sbjct 674 LGWR 677

Lambda K H a alpha

0.314 0.131 0.373 0.792 4.96

Gapped

Lambda K H a alpha sigma

0.267 0.0410 0.140 1.90 42.6 43.6

Effective search space used: 21919800

**Query=** NODE_218_length_506_cov_0.443272

Length=506

***** No hits found *****

Lambda K H a alpha

0.318 0.134 0.401 0.792 4.96

Gapped

Lambda K H a alpha sigma

0.267 0.0410 0.140 1.90 42.6 43.6

Effective search space used: 21704900

**Query=** NODE_219_length_505_cov_0.201058

Length=505

***** No hits found *****

Lambda K H a alpha

0.318 0.134 0.401 0.792 4.96

Gapped

Lambda K H a alpha sigma

0.267 0.0410 0.140 1.90 42.6 43.6

Effective search space used: 21704900

**Query=** NODE_220_length_504_cov_0.721485

Length=504

Score E

Sequences producing significant alignments: (Bits) Value

DJ39_RS02575_-_25228845_translation chloramphenicol resistance ... [26.2](file:///Users/JMBNew/Desktop/Articles%20en%20Cours/Article%20Diane/Bb_SR-11-14_output-blast_named.html#BL_ORD_ID:647) 0.26

> DJ39_RS02575_-_25228845_translation chloramphenicol resistance

permease RarD

Length=296

Score = 26.2 bits (56), Expect = 0.26, Method: Compositional matrix adjust.

Identities = 16/33 (48%), Positives = 19/33 (58%), Gaps = 1/33 (3%)

Frame = -2

Query 371 PGFR-QAAGFFVGHEAGLHAGAREKPIGPGQVA 276

PG Q AG+ VG+E +H G RE P G Q A

Sbjct 87 PGIHAQMAGWTVGNEKQVHGGHRE*PQGFDQHA 119

Lambda K H a alpha

0.318 0.134 0.401 0.792 4.96

Gapped

Lambda K H a alpha sigma

0.267 0.0410 0.140 1.90 42.6 43.6

Effective search space used: 21704900

**Query=** NODE_221_length_499_cov_0.462766

Length=499

***** No hits found *****

Lambda K H a alpha

0.318 0.134 0.401 0.792 4.96

Gapped

Lambda K H a alpha sigma

0.267 0.0410 0.140 1.90 42.6 43.6

Effective search space used: 21275100

**Query=** NODE_222_length_502_cov_0.744681

Length=502

***** No hits found *****

Lambda K H a alpha

0.317 0.130 0.390 0.792 4.96

Gapped

Lambda K H a alpha sigma

0.267 0.0410 0.140 1.90 42.6 43.6

Effective search space used: 21490000

**Query=** NODE_223_length_503_cov_0.448

Length=503

***** No hits found *****

Lambda K H a alpha

0.318 0.134 0.401 0.792 4.96

Gapped

Lambda K H a alpha sigma

0.267 0.0410 0.140 1.90 42.6 43.6

Effective search space used: 21490000

**Query=** NODE_224_length_499_cov_0.747989

Length=499

***** No hits found *****

Lambda K H a alpha

0.318 0.134 0.401 0.792 4.96

Gapped

Lambda K H a alpha sigma

0.267 0.0410 0.140 1.90 42.6 43.6

Effective search space used: 21275100

**Query=** NODE_225_length_499_cov_0.509383

Length=499

***** No hits found *****

Lambda K H a alpha

0.318 0.134 0.401 0.792 4.96

Gapped

Lambda K H a alpha sigma

0.267 0.0410 0.140 1.90 42.6 43.6

Effective search space used: 21275100

**Query=** NODE_226_length_499_cov_0.674731

Length=499

***** No hits found *****

Lambda K H a alpha

0.318 0.134 0.401 0.792 4.96

Gapped

Lambda K H a alpha sigma

0.267 0.0410 0.140 1.90 42.6 43.6

Effective search space used: 21275100

**Query=** NODE_227_length_499_cov_0.72043

Length=499

***** No hits found *****

Lambda K H a alpha

0.318 0.134 0.401 0.792 4.96

Gapped

Lambda K H a alpha sigma

0.267 0.0410 0.140 1.90 42.6 43.6

Effective search space used: 21275100

**Query=** NODE_228_length_499_cov_0.379032

Length=499

***** No hits found *****

Lambda K H a alpha

0.318 0.134 0.401 0.792 4.96

Gapped

Lambda K H a alpha sigma

0.267 0.0410 0.140 1.90 42.6 43.6

Effective search space used: 21275100

**Query=** NODE_229_length_498_cov_0.681941

Length=498

***** No hits found *****

Lambda K H a alpha

0.318 0.134 0.401 0.792 4.96

Gapped

Lambda K H a alpha sigma

0.267 0.0410 0.140 1.90 42.6 43.6

Effective search space used: 21275100

**Query=** NODE_230_length_496_cov_0.439353

Length=496

Score E

Sequences producing significant alignments: (Bits) Value

lpg0177_-_19831744_translation chloramphenicol resistance protein [25.4](file:///Users/JMBNew/Desktop/Articles%20en%20Cours/Article%20Diane/Bb_SR-11-14_output-blast_named.html#BL_ORD_ID:681) 0.44

> lpg0177_-_19831744_translation chloramphenicol resistance protein

Length=382

Score = 25.4 bits (54), Expect = 0.44, Method: Compositional matrix adjust.

Identities = 12/30 (40%), Positives = 17/30 (57%), Gaps = 3/30 (10%)

Frame = +3

Query 336 TPACRAIWVSWPRPSARSR---RTWTATCA 416

TP C +I + + RPSA+ R W+A A

Sbjct 109 TPLCISIAIHFTRPSAKGRILSFIWSANSA 138

Lambda K H a alpha

0.318 0.134 0.401 0.792 4.96

Gapped

Lambda K H a alpha sigma

0.267 0.0410 0.140 1.90 42.6 43.6

Effective search space used: 21060200

**Query=** NODE_231_length_497_cov_0.719677

Length=497

***** No hits found *****

Lambda K H a alpha

0.318 0.134 0.401 0.792 4.96

Gapped

Lambda K H a alpha sigma

0.267 0.0410 0.140 1.90 42.6 43.6

Effective search space used: 21060200

**Query=** NODE_232_length_498_cov_0.420485

Length=498

***** No hits found *****

Lambda K H a alpha

0.318 0.134 0.401 0.792 4.96

Gapped

Lambda K H a alpha sigma

0.267 0.0410 0.140 1.90 42.6 43.6

Effective search space used: 21275100

**Query=** NODE_233_length_496_cov_0.680217

Length=496

***** No hits found *****

Lambda K H a alpha

0.318 0.134 0.401 0.792 4.96

Gapped

Lambda K H a alpha sigma

0.267 0.0410 0.140 1.90 42.6 43.6

Effective search space used: 21060200

**Query=** NODE_234_length_494_cov_0.682927

Length=494

***** No hits found *****

Lambda K H a alpha

0.318 0.134 0.401 0.792 4.96

Gapped

Lambda K H a alpha sigma

0.267 0.0410 0.140 1.90 42.6 43.6

Effective search space used: 20845300

**Query=** NODE_235_length_496_cov_0.723577

Length=496

***** No hits found *****

Lambda K H a alpha

0.318 0.134 0.401 0.792 4.96

Gapped

Lambda K H a alpha sigma

0.267 0.0410 0.140 1.90 42.6 43.6

Effective search space used: 21060200

**Query=** NODE_236_length_496_cov_0.704607

Length=496

***** No hits found *****

Lambda K H a alpha

0.318 0.134 0.401 0.792 4.96

Gapped

Lambda K H a alpha sigma

0.267 0.0410 0.140 1.90 42.6 43.6

Effective search space used: 21060200

**Query=** NODE_237_length_493_cov_0.75

Length=493

***** No hits found *****

Lambda K H a alpha

0.318 0.134 0.401 0.792 4.96

Gapped

Lambda K H a alpha sigma

0.267 0.0410 0.140 1.90 42.6 43.6

Effective search space used: 20845300

**Query=** NODE_238_length_495_cov_0.896739

Length=495

***** No hits found *****

Lambda K H a alpha

0.318 0.134 0.401 0.792 4.96

Gapped

Lambda K H a alpha sigma

0.267 0.0410 0.140 1.90 42.6 43.6

Effective search space used: 21060200

**Query=** NODE_239_length_495_cov_0.434783

Length=495

Score E

Sequences producing significant alignments: (Bits) Value

YP_111179 transport/efflux protein [Burkholderia pseudomallei K... [25.4](file:///Users/JMBNew/Desktop/Articles%20en%20Cours/Article%20Diane/Bb_SR-11-14_output-blast_named.html#BL_ORD_ID:597) 0.43

CAH38634 putative transport/efflux protein [Burkholderia pseudo... [25.4](file:///Users/JMBNew/Desktop/Articles%20en%20Cours/Article%20Diane/Bb_SR-11-14_output-blast_named.html#BL_ORD_ID:328) 0.43

> YP_111179 transport/efflux protein [Burkholderia pseudomallei

K96243]

Length=416

Score = 25.4 bits (54), Expect = 0.43, Method: Compositional matrix adjust.

Identities = 17/50 (34%), Positives = 25/50 (50%), Gaps = 4/50 (8%)

Frame = +1

Query 160 NGLRRQAPVES--MDGIWNVVGS--APRPTENLGRPRLRGPATVTRGTGG 297

NGL + A + + G+ + GS APR ++ +G P L G A GG

Sbjct 244 NGLTQNAFSAAFFLCGLTEIAGSLLAPRISKRIGHPALTGLACACCALGG 293

> CAH38634 putative transport/efflux protein [Burkholderia pseudomallei

K96243]

Length=416

Score = 25.4 bits (54), Expect = 0.43, Method: Compositional matrix adjust.

Identities = 17/50 (34%), Positives = 25/50 (50%), Gaps = 4/50 (8%)

Frame = +1

Query 160 NGLRRQAPVES--MDGIWNVVGS--APRPTENLGRPRLRGPATVTRGTGG 297

NGL + A + + G+ + GS APR ++ +G P L G A GG

Sbjct 244 NGLTQNAFSAAFFLCGLTEIAGSLLAPRISKRIGHPALTGLACACCALGG 293

Lambda K H a alpha

0.318 0.134 0.401 0.792 4.96

Gapped

Lambda K H a alpha sigma

0.267 0.0410 0.140 1.90 42.6 43.6

Effective search space used: 21060200

**Query=** NODE_240_length_492_cov_0.50411

Length=492

***** No hits found *****

Lambda K H a alpha

0.318 0.134 0.401 0.792 4.96

Gapped

Lambda K H a alpha sigma

0.267 0.0410 0.140 1.90 42.6 43.6

Effective search space used: 20845300

**Query=** NODE_241_length_491_cov_0.695055

Length=491

***** No hits found *****

Lambda K H a alpha

0.318 0.134 0.401 0.792 4.96

Gapped

Lambda K H a alpha sigma

0.267 0.0410 0.140 1.90 42.6 43.6

Effective search space used: 20630400

**Query=** NODE_242_length_491_cov_0.711538

Length=491

***** No hits found *****

Lambda K H a alpha

0.318 0.134 0.401 0.792 4.96

Gapped

Lambda K H a alpha sigma

0.267 0.0410 0.140 1.90 42.6 43.6

Effective search space used: 20630400

**Query=** NODE_243_length_490_cov_0.498623

Length=490

Score E

Sequences producing significant alignments: (Bits) Value

XP_018179360 florfenicol exporter [Purpureocillium lilacinum] [25.0](file:///Users/JMBNew/Desktop/Articles%20en%20Cours/Article%20Diane/Bb_SR-11-14_output-blast_named.html#BL_ORD_ID:594) 0.78

OAQ90641 florfenicol exporter [Purpureocillium lilacinum] [25.0](file:///Users/JMBNew/Desktop/Articles%20en%20Cours/Article%20Diane/Bb_SR-11-14_output-blast_named.html#BL_ORD_ID:509) 0.78

OAQ83863 florfenicol exporter [Purpureocillium lilacinum] [24.6](file:///Users/JMBNew/Desktop/Articles%20en%20Cours/Article%20Diane/Bb_SR-11-14_output-blast_named.html#BL_ORD_ID:508) 0.79

> XP_018179360 florfenicol exporter [Purpureocillium lilacinum]

Length=564

Score = 25.0 bits (53), Expect = 0.78, Method: Compositional matrix adjust.

Identities = 9/14 (64%), Positives = 10/14 (71%), Gaps = 0/14 (0%)

Frame = -2

Query 354 RGTPVERARRDLTW 313

R P+ERAR DL W

Sbjct 398 RKFPIERARLDLMW 411

> OAQ90641 florfenicol exporter [Purpureocillium lilacinum]

Length=564

Score = 25.0 bits (53), Expect = 0.78, Method: Compositional matrix adjust.

Identities = 9/14 (64%), Positives = 10/14 (71%), Gaps = 0/14 (0%)

Frame = -2

Query 354 RGTPVERARRDLTW 313

R P+ERAR DL W

Sbjct 398 RKFPIERARLDLMW 411

> OAQ83863 florfenicol exporter [Purpureocillium lilacinum]

Length=539

Score = 24.6 bits (52), Expect = 0.79, Method: Compositional matrix adjust.

Identities = 9/14 (64%), Positives = 10/14 (71%), Gaps = 0/14 (0%)

Frame = -2

Query 354 RGTPVERARRDLTW 313

R P+ERAR DL W

Sbjct 398 RKFPIERARLDLMW 411

Lambda K H a alpha

0.318 0.134 0.401 0.792 4.96

Gapped

Lambda K H a alpha sigma

0.267 0.0410 0.140 1.90 42.6 43.6

Effective search space used: 20630400

**Query=** NODE_244_length_489_cov_0.701657

Length=489

***** No hits found *****

Lambda K H a alpha

0.318 0.134 0.401 0.792 4.96

Gapped

Lambda K H a alpha sigma

0.267 0.0410 0.140 1.90 42.6 43.6

Effective search space used: 20630400

**Query=** NODE_245_length_489_cov_0.535912

Length=489

***** No hits found *****

Lambda K H a alpha

0.318 0.134 0.401 0.792 4.96

Gapped

Lambda K H a alpha sigma

0.267 0.0410 0.140 1.90 42.6 43.6

Effective search space used: 20630400

**Query=** NODE_246_length_487_cov_1.2133

Length=487

***** No hits found *****

Lambda K H a alpha

0.318 0.134 0.401 0.792 4.96

Gapped

Lambda K H a alpha sigma

0.267 0.0410 0.140 1.90 42.6 43.6

Effective search space used: 20415500

**Query=** NODE_247_length_488_cov_0.598338

Length=488

***** No hits found *****

Lambda K H a alpha

0.314 0.133 0.393 0.792 4.96

Gapped

Lambda K H a alpha sigma

0.267 0.0410 0.140 1.90 42.6 43.6

Effective search space used: 20415500

**Query=** NODE_248_length_487_cov_0.713889

Length=487

***** No hits found *****

Lambda K H a alpha

0.318 0.134 0.401 0.792 4.96

Gapped

Lambda K H a alpha sigma

0.267 0.0410 0.140 1.90 42.6 43.6

Effective search space used: 20415500

**Query=** NODE_249_length_487_cov_0.569444

Length=487

***** No hits found *****

Lambda K H a alpha

0.318 0.134 0.401 0.792 4.96

Gapped

Lambda K H a alpha sigma

0.267 0.0410 0.140 1.90 42.6 43.6

Effective search space used: 20415500

**Query=** NODE_250_length_487_cov_0.713889

Length=487

***** No hits found *****

Lambda K H a alpha

0.318 0.134 0.401 0.792 4.96

Gapped

Lambda K H a alpha sigma

0.267 0.0410 0.140 1.90 42.6 43.6

Effective search space used: 20415500

**Query=** NODE_251_length_487_cov_0.897222

Length=487

***** No hits found *****

Lambda K H a alpha

0.318 0.134 0.401 0.792 4.96

Gapped

Lambda K H a alpha sigma

0.267 0.0410 0.140 1.90 42.6 43.6

Effective search space used: 20415500

**Query=** NODE_252_length_487_cov_0.811111

Length=487

***** No hits found *****

Lambda K H a alpha

0.318 0.134 0.401 0.792 4.96

Gapped

Lambda K H a alpha sigma

0.267 0.0410 0.140 1.90 42.6 43.6

Effective search space used: 20415500

**Query=** NODE_253_length_486_cov_0.548747

Length=486

***** No hits found *****

Lambda K H a alpha

0.318 0.134 0.401 0.792 4.96

Gapped

Lambda K H a alpha sigma

0.267 0.0410 0.140 1.90 42.6 43.6

Effective search space used: 20415500

**Query=** NODE_254_length_483_cov_0.719888

Length=483

***** No hits found *****

Lambda K H a alpha

0.318 0.134 0.401 0.792 4.96

Gapped

Lambda K H a alpha sigma

0.267 0.0410 0.140 1.90 42.6 43.6

Effective search space used: 20482190

**Query=** NODE_255_length_484_cov_0.540616

Length=484

***** No hits found *****

Lambda K H a alpha

0.318 0.134 0.401 0.792 4.96

Gapped

Lambda K H a alpha sigma

0.267 0.0410 0.140 1.90 42.6 43.6

Effective search space used: 20482190

**Query=** NODE_256_length_484_cov_0.327731

Length=484

***** No hits found *****

Lambda K H a alpha

0.318 0.134 0.401 0.792 4.96

Gapped

Lambda K H a alpha sigma

0.267 0.0410 0.140 1.90 42.6 43.6

Effective search space used: 20482190

**Query=** NODE_257_length_483_cov_0.47191

Length=483

***** No hits found *****

Lambda K H a alpha

0.318 0.134 0.401 0.792 4.96

Gapped

Lambda K H a alpha sigma

0.267 0.0410 0.140 1.90 42.6 43.6

Effective search space used: 20482190

**Query=** NODE_258_length_482_cov_0.701408

Length=482

***** No hits found *****

Lambda K H a alpha

0.318 0.134 0.401 0.792 4.96

Gapped

Lambda K H a alpha sigma

0.267 0.0410 0.140 1.90 42.6 43.6

Effective search space used: 20266588

**Query=** NODE_259_length_482_cov_0.667606

Length=482

***** No hits found *****

Lambda K H a alpha

0.318 0.134 0.401 0.792 4.96

Gapped

Lambda K H a alpha sigma

0.267 0.0410 0.140 1.90 42.6 43.6

Effective search space used: 20266588

**Query=** NODE_260_length_481_cov_0.771831

Length=481

***** No hits found *****

Lambda K H a alpha

0.318 0.134 0.401 0.792 4.96

Gapped

Lambda K H a alpha sigma

0.267 0.0410 0.140 1.90 42.6 43.6

Effective search space used: 20266588

**Query=** NODE_261_length_482_cov_0.84507

Length=482

***** No hits found *****

Lambda K H a alpha

0.318 0.134 0.401 0.792 4.96

Gapped

Lambda K H a alpha sigma

0.267 0.0410 0.140 1.90 42.6 43.6

Effective search space used: 20266588

**Query=** NODE_262_length_482_cov_0.701408

Length=482

***** No hits found *****

Lambda K H a alpha

0.318 0.134 0.401 0.792 4.96

Gapped

Lambda K H a alpha sigma

0.267 0.0410 0.140 1.90 42.6 43.6

Effective search space used: 20266588

**Query=** NODE_263_length_482_cov_0.709859

Length=482

***** No hits found *****

Lambda K H a alpha

0.318 0.134 0.401 0.792 4.96

Gapped

Lambda K H a alpha sigma

0.267 0.0410 0.140 1.90 42.6 43.6

Effective search space used: 20266588

**Query=** NODE_264_length_481_cov_0.663842

Length=481

***** No hits found *****

Lambda K H a alpha

0.318 0.134 0.401 0.792 4.96

Gapped

Lambda K H a alpha sigma

0.267 0.0410 0.140 1.90 42.6 43.6

Effective search space used: 20266588

**Query=** NODE_265_length_479_cov_0.417614

Length=479

***** No hits found *****

Lambda K H a alpha

0.318 0.134 0.401 0.792 4.96

Gapped

Lambda K H a alpha sigma

0.267 0.0410 0.140 1.90 42.6 43.6

Effective search space used: 20050986

**Query=** NODE_266_length_477_cov_0.7151

Length=477

***** No hits found *****

Lambda K H a alpha

0.318 0.134 0.401 0.792 4.96

Gapped

Lambda K H a alpha sigma

0.267 0.0410 0.140 1.90 42.6 43.6

Effective search space used: 20050986

**Query=** NODE_267_length_477_cov_0.703704

Length=477

***** No hits found *****

Lambda K H a alpha

0.318 0.134 0.401 0.792 4.96

Gapped

Lambda K H a alpha sigma

0.267 0.0410 0.140 1.90 42.6 43.6

Effective search space used: 20050986

**Query=** NODE_268_length_476_cov_0.277143

Length=476

***** No hits found *****

Lambda K H a alpha

0.318 0.134 0.401 0.792 4.96

Gapped

Lambda K H a alpha sigma

0.267 0.0410 0.140 1.90 42.6 43.6

Effective search space used: 19835384

**Query=** NODE_269_length_477_cov_3.67714

Length=477

***** No hits found *****

Lambda K H a alpha

0.318 0.134 0.401 0.792 4.96

Gapped

Lambda K H a alpha sigma

0.267 0.0410 0.140 1.90 42.6 43.6

Effective search space used: 20050986

**Query=** NODE_270_length_475_cov_0.636103

Length=475

Score E

Sequences producing significant alignments: (Bits) Value

AQW34720 copper-transporting P-type ATPase B [Staphylococcus sc... [27.3](file:///Users/JMBNew/Desktop/Articles%20en%20Cours/Article%20Diane/Bb_SR-11-14_output-blast_named.html#BL_ORD_ID:271) 0.11

> AQW34720 copper-transporting P-type ATPase B [Staphylococcus

sciuri]

Length=701

Score = 27.3 bits (59), Expect = 0.11, Method: Composition-based stats.

Identities = 9/27 (33%), Positives = 16/27 (59%), Gaps = 0/27 (0%)

Frame = +1

Query 148 SEVFFFYAKRPYTSAAQGTLGLRKRGM 228

+ + FFY +P+ S A+ + +K GM

Sbjct 97 ATILFFYGGKPFLSGAKDEISTKKPGM 123

Lambda K H a alpha

0.318 0.134 0.401 0.792 4.96

Gapped

Lambda K H a alpha sigma

0.267 0.0410 0.140 1.90 42.6 43.6

Effective search space used: 19835384

**Query=** NODE_271_length_475_cov_0.701149

Length=475

***** No hits found *****

Lambda K H a alpha

0.318 0.134 0.401 0.792 4.96

Gapped

Lambda K H a alpha sigma

0.267 0.0410 0.140 1.90 42.6 43.6

Effective search space used: 19835384

**Query=** NODE_272_length_474_cov_0.360231

Length=474

***** No hits found *****

Lambda K H a alpha

0.318 0.134 0.401 0.792 4.96

Gapped

Lambda K H a alpha sigma

0.267 0.0410 0.140 1.90 42.6 43.6

Effective search space used: 19835384

**Query=** NODE_273_length_474_cov_0.685879

Length=474

***** No hits found *****

Lambda K H a alpha

0.318 0.134 0.401 0.792 4.96

Gapped

Lambda K H a alpha sigma

0.267 0.0410 0.140 1.90 42.6 43.6

Effective search space used: 19835384

**Query=** NODE_274_length_473_cov_0.398844

Length=473

***** No hits found *****

Lambda K H a alpha

0.318 0.134 0.401 0.792 4.96

Gapped

Lambda K H a alpha sigma

0.267 0.0410 0.140 1.90 42.6 43.6

Effective search space used: 19619782

**Query=** NODE_275_length_471_cov_0.55942

Length=471

***** No hits found *****

Lambda K H a alpha

0.318 0.134 0.401 0.792 4.96

Gapped

Lambda K H a alpha sigma

0.267 0.0410 0.140 1.90 42.6 43.6

Effective search space used: 19619782

**Query=** NODE_276_length_472_cov_0.730435

Length=472

***** No hits found *****

Lambda K H a alpha

0.318 0.134 0.401 0.792 4.96

Gapped

Lambda K H a alpha sigma

0.267 0.0410 0.140 1.90 42.6 43.6

Effective search space used: 19619782

**Query=** NODE_277_length_472_cov_0.698551

Length=472

***** No hits found *****

Lambda K H a alpha

0.318 0.134 0.401 0.792 4.96

Gapped

Lambda K H a alpha sigma

0.267 0.0410 0.140 1.90 42.6 43.6

Effective search space used: 19619782

**Query=** NODE_278_length_472_cov_0.608696

Length=472

***** No hits found *****

Lambda K H a alpha

0.318 0.134 0.401 0.792 4.96

Gapped

Lambda K H a alpha sigma

0.267 0.0410 0.140 1.90 42.6 43.6

Effective search space used: 19619782

**Query=** NODE_279_length_471_cov_0.755814

Length=471

***** No hits found *****

Lambda K H a alpha

0.318 0.134 0.401 0.792 4.96

Gapped

Lambda K H a alpha sigma

0.267 0.0410 0.140 1.90 42.6 43.6

Effective search space used: 19619782

**Query=** NODE_280_length_471_cov_0.427326

Length=471

***** No hits found *****

Lambda K H a alpha

0.318 0.134 0.401 0.792 4.96

Gapped

Lambda K H a alpha sigma

0.267 0.0410 0.140 1.90 42.6 43.6

Effective search space used: 19619782

**Query=** NODE_281_length_470_cov_0.74344

Length=470

***** No hits found *****

Lambda K H a alpha

0.318 0.134 0.401 0.792 4.96

Gapped

Lambda K H a alpha sigma

0.267 0.0410 0.140 1.90 42.6 43.6

Effective search space used: 19404180

**Query=** NODE_282_length_470_cov_0.655977

Length=470

***** No hits found *****

Lambda K H a alpha

0.318 0.134 0.401 0.792 4.96

Gapped

Lambda K H a alpha sigma

0.267 0.0410 0.140 1.90 42.6 43.6

Effective search space used: 19404180

**Query=** NODE_283_length_470_cov_0.393586

Length=470

***** No hits found *****

Lambda K H a alpha

0.318 0.134 0.401 0.792 4.96

Gapped

Lambda K H a alpha sigma

0.267 0.0410 0.140 1.90 42.6 43.6

Effective search space used: 19404180

**Query=** NODE_284_length_468_cov_0.739766

Length=468

***** No hits found *****

Lambda K H a alpha

0.318 0.134 0.401 0.792 4.96

Gapped

Lambda K H a alpha sigma

0.267 0.0410 0.140 1.90 42.6 43.6

Effective search space used: 19404180

**Query=** NODE_285_length_467_cov_0.727273

Length=467

Score E

Sequences producing significant alignments: (Bits) Value

floR_-_12372839_translation floR: efflux transporter [25.4](file:///Users/JMBNew/Desktop/Articles%20en%20Cours/Article%20Diane/Bb_SR-11-14_output-blast_named.html#BL_ORD_ID:661) 0.52

lpg0177_-_19831744_translation chloramphenicol resistance protein [24.6](file:///Users/JMBNew/Desktop/Articles%20en%20Cours/Article%20Diane/Bb_SR-11-14_output-blast_named.html#BL_ORD_ID:681) 0.69

> floR_-_12372839_translation floR: efflux transporter

Length=428

Score = 25.4 bits (54), Expect = 0.52, Method: Compositional matrix adjust.

Identities = 9/18 (50%), Positives = 13/18 (72%), Gaps = 0/18 (0%)

Frame = -2

Query 253 STVARLALAWCTQQGSLG 200

S++A +AL WC QG+L

Sbjct 122 SSIAVIALFWCITQGALN 139

> lpg0177_-_19831744_translation chloramphenicol resistance protein

Length=382

Score = 24.6 bits (52), Expect = 0.69, Method: Compositional matrix adjust.

Identities = 11/23 (48%), Positives = 15/23 (65%), Gaps = 0/23 (0%)

Frame = -1

Query 380 LQVSGLWCFRSEQGVGVTSYVTL 312

L V G+ CF + +G+ SYVTL

Sbjct 202 LSVIGITCFTALASLGLYSYVTL 224

Lambda K H a alpha

0.318 0.134 0.401 0.792 4.96

Gapped

Lambda K H a alpha sigma

0.267 0.0410 0.140 1.90 42.6 43.6

Effective search space used: 19188578

**Query=** NODE_286_length_468_cov_0.727273

Length=468

***** No hits found *****

Lambda K H a alpha

0.318 0.134 0.401 0.792 4.96

Gapped

Lambda K H a alpha sigma

0.267 0.0410 0.140 1.90 42.6 43.6

Effective search space used: 19404180

**Query=** NODE_287_length_467_cov_0.711765

Length=467

Score E

Sequences producing significant alignments: (Bits) Value

ACS55612 drug resistance transporter, Bcr/CflA subfamily [Rhizo... [25.0](file:///Users/JMBNew/Desktop/Articles%20en%20Cours/Article%20Diane/Bb_SR-11-14_output-blast_named.html#BL_ORD_ID:56) 0.62

> ACS55612 drug resistance transporter, Bcr/CflA subfamily [Rhizobium

leguminosarum bv. trifolii WSM1325]

Length=395

Score = 25.0 bits (53), Expect = 0.62, Method: Compositional matrix adjust.

Identities = 11/35 (31%), Positives = 20/35 (57%), Gaps = 0/35 (0%)

Frame = -2

Query 313 CAQAMNIASQSSFMVCLAICETAWALILPSSSTSL 209

C Q++ + +F V L ++AW L+ S+T+L

Sbjct 347 CVQSIIVGFAGTFFVILLGGDSAWPLVGYVSATAL 381

Lambda K H a alpha

0.318 0.134 0.401 0.792 4.96

Gapped

Lambda K H a alpha sigma

0.267 0.0410 0.140 1.90 42.6 43.6

Effective search space used: 19188578

**Query=** NODE_288_length_467_cov_0.75

Length=467

***** No hits found *****

Lambda K H a alpha

0.318 0.134 0.401 0.792 4.96

Gapped

Lambda K H a alpha sigma

0.267 0.0410 0.140 1.90 42.6 43.6

Effective search space used: 19188578

**Query=** NODE_289_length_467_cov_3.06471

Length=467

***** No hits found *****

Lambda K H a alpha

0.318 0.134 0.401 0.792 4.96

Gapped

Lambda K H a alpha sigma

0.267 0.0410 0.140 1.90 42.6 43.6

Effective search space used: 19188578

**Query=** NODE_290_length_467_cov_0.734513

Length=467

***** No hits found *****

Lambda K H a alpha

0.318 0.134 0.401 0.792 4.96

Gapped

Lambda K H a alpha sigma

0.267 0.0410 0.140 1.90 42.6 43.6

Effective search space used: 19188578

**Query=** NODE_291_length_466_cov_0.528024

Length=466

Score E

Sequences producing significant alignments: (Bits) Value

GAM36585 florfenicol exporter [Talaromyces cellulolyticus] [25.4](file:///Users/JMBNew/Desktop/Articles%20en%20Cours/Article%20Diane/Bb_SR-11-14_output-blast_named.html#BL_ORD_ID:470) 0.46

> GAM36585 florfenicol exporter [Talaromyces cellulolyticus]

Length=292

Score = 25.4 bits (54), Expect = 0.46, Method: Compositional matrix adjust.

Identities = 16/48 (33%), Positives = 22/48 (46%), Gaps = 10/48 (21%)

Frame = +1

Query 283 KK*NEQDSAAARDTYQQ----------PV*FQEEKIVILFYNLQKYMG 396

+K NE AA R+T + P+ FQ+E +IL Y Y G

Sbjct 39 RKKNENRHAAKRETMSKKQRPNLLSSIPILFQKESFLILIYGAFVYSG 86

Lambda K H a alpha

0.318 0.134 0.401 0.792 4.96

Gapped

Lambda K H a alpha sigma

0.267 0.0410 0.140 1.90 42.6 43.6

Effective search space used: 19188578

**Query=** NODE_292_length_466_cov_0.687316

Length=466

***** No hits found *****

Lambda K H a alpha

0.318 0.134 0.401 0.792 4.96

Gapped

Lambda K H a alpha sigma

0.267 0.0410 0.140 1.90 42.6 43.6

Effective search space used: 19188578

**Query=** NODE_293_length_466_cov_0.507375

Length=466

Score E

Sequences producing significant alignments: (Bits) Value

AQW34662 multidrug ABC transporter ATP-binding protein [Staphyl... [25.4](file:///Users/JMBNew/Desktop/Articles%20en%20Cours/Article%20Diane/Bb_SR-11-14_output-blast_named.html#BL_ORD_ID:257) 0.43

AQW34613 multidrug ABC transporter ATP-binding protein [Staphyl... [25.4](file:///Users/JMBNew/Desktop/Articles%20en%20Cours/Article%20Diane/Bb_SR-11-14_output-blast_named.html#BL_ORD_ID:247) 0.45

> AQW34662 multidrug ABC transporter ATP-binding protein [Staphylococcus

sciuri]

Length=579

Score = 25.4 bits (54), Expect = 0.43, Method: Composition-based stats.

Identities = 11/27 (41%), Positives = 18/27 (67%), Gaps = 0/27 (0%)

Frame = -1

Query 169 ALEHADRGYVMESGEITLSGPAREMLH 89

++HA++ VM+ GEIT SG ++L

Sbjct 533 GVKHANQIVVMDHGEITESGTHDQLLQ 559

> AQW34613 multidrug ABC transporter ATP-binding protein [Staphylococcus

sciuri]

Length=579

Score = 25.4 bits (54), Expect = 0.45, Method: Composition-based stats.

Identities = 11/27 (41%), Positives = 18/27 (67%), Gaps = 0/27 (0%)

Frame = -1

Query 169 ALEHADRGYVMESGEITLSGPAREMLH 89

++HA++ VM+ GEIT SG ++L

Sbjct 533 GVKHANQIVVMDHGEITESGTHDQLLQ 559

Lambda K H a alpha

0.318 0.134 0.401 0.792 4.96

Gapped

Lambda K H a alpha sigma

0.267 0.0410 0.140 1.90 42.6 43.6

Effective search space used: 19188578

**Query=** NODE_294_length_465_cov_0.721893

Length=465

***** No hits found *****

Lambda K H a alpha

0.318 0.134 0.401 0.792 4.96

Gapped

Lambda K H a alpha sigma

0.267 0.0410 0.140 1.90 42.6 43.6

Effective search space used: 19188578

**Query=** NODE_295_length_465_cov_0.559172

Length=465

Score E

Sequences producing significant alignments: (Bits) Value

EEA95839 florfenicol resistance protein [Pseudovibrio sp. JE062] [24.6](file:///Users/JMBNew/Desktop/Articles%20en%20Cours/Article%20Diane/Bb_SR-11-14_output-blast_named.html#BL_ORD_ID:418) 0.85

> EEA95839 florfenicol resistance protein [Pseudovibrio sp. JE062]

Length=284

Score = 24.6 bits (52), Expect = 0.85, Method: Compositional matrix adjust.

Identities = 9/21 (43%), Positives = 14/21 (67%), Gaps = 0/21 (0%)

Frame = -1

Query 123 AGVRRGVEADPGAGQVGEDAP 61

AG+R V+ + AGQ+ +D P

Sbjct 218 AGIRNAVKLELSAGQISDDRP 238

Lambda K H a alpha

0.318 0.134 0.401 0.792 4.96

Gapped

Lambda K H a alpha sigma

0.267 0.0410 0.140 1.90 42.6 43.6

Effective search space used: 19188578

**Query=** NODE_296_length_464_cov_0.51632

Length=464

***** No hits found *****

Lambda K H a alpha

0.318 0.134 0.401 0.792 4.96

Gapped

Lambda K H a alpha sigma

0.267 0.0410 0.140 1.90 42.6 43.6

Effective search space used: 18972976

**Query=** NODE_297_length_464_cov_0.540059

Length=464

***** No hits found *****

Lambda K H a alpha

0.318 0.134 0.401 0.792 4.96

Gapped

Lambda K H a alpha sigma

0.267 0.0410 0.140 1.90 42.6 43.6

Effective search space used: 18972976

**Query=** NODE_298_length_463_cov_0.744807

Length=463

Score E

Sequences producing significant alignments: (Bits) Value

XP_002480947 florfenicol exporter, putative [Talaromyces stipit... [24.3](file:///Users/JMBNew/Desktop/Articles%20en%20Cours/Article%20Diane/Bb_SR-11-14_output-blast_named.html#BL_ORD_ID:583) 0.068

EED20513 florfenicol exporter, putative [Talaromyces stipitatus... [24.3](file:///Users/JMBNew/Desktop/Articles%20en%20Cours/Article%20Diane/Bb_SR-11-14_output-blast_named.html#BL_ORD_ID:424) 0.068

> XP_002480947 florfenicol exporter, putative [Talaromyces stipitatus

ATCC 10500]

Length=564

Score = 24.3 bits (51), Expect(2) = 0.068, Method: Compositional matrix adjust.

Identities = 14/41 (34%), Positives = 21/41 (51%), Gaps = 0/41 (0%)

Frame = +3

Query 261 NGTTPAKGVRWTQRGGLAARRRPPTAILGDTASLPPSPRPV 383

NG+ P + G L ARR I+ ++LPP+PR +

Sbjct 286 NGSIPPPTWNMSLIGYLEARRVSERDIIAAESTLPPTPRKL 326

Score = 21.9 bits (45), Expect(2) = 0.068, Method: Composition-based stats.

Identities = 9/14 (64%), Positives = 9/14 (64%), Gaps = 0/14 (0%)

Frame = +1

Query 364 PRPRGLSFKQHLPT 405

P PR LSF LPT

Sbjct 321 PTPRKLSFPNPLPT 334

> EED20513 florfenicol exporter, putative [Talaromyces stipitatus

ATCC 10500]

Length=564

Score = 24.3 bits (51), Expect(2) = 0.068, Method: Compositional matrix adjust.

Identities = 14/41 (34%), Positives = 21/41 (51%), Gaps = 0/41 (0%)

Frame = +3

Query 261 NGTTPAKGVRWTQRGGLAARRRPPTAILGDTASLPPSPRPV 383

NG+ P + G L ARR I+ ++LPP+PR +

Sbjct 286 NGSIPPPTWNMSLIGYLEARRVSERDIIAAESTLPPTPRKL 326

Score = 21.9 bits (45), Expect(2) = 0.068, Method: Composition-based stats.

Identities = 9/14 (64%), Positives = 9/14 (64%), Gaps = 0/14 (0%)

Frame = +1

Query 364 PRPRGLSFKQHLPT 405

P PR LSF LPT

Sbjct 321 PTPRKLSFPNPLPT 334

Lambda K H a alpha

0.318 0.134 0.401 0.792 4.96

Gapped

Lambda K H a alpha sigma

0.267 0.0410 0.140 1.90 42.6 43.6

Effective search space used: 18972976

**Query=** NODE_299_length_463_cov_0.6875

Length=463

***** No hits found *****

Lambda K H a alpha

0.318 0.134 0.401 0.792 4.96

Gapped

Lambda K H a alpha sigma

0.267 0.0410 0.140 1.90 42.6 43.6

Effective search space used: 18972976

**Query=** NODE_300_length_462_cov_1.24702

Length=462

***** No hits found *****

Lambda K H a alpha

0.318 0.134 0.401 0.792 4.96

Gapped

Lambda K H a alpha sigma

0.267 0.0410 0.140 1.90 42.6 43.6

Effective search space used: 18972976

**Query=** NODE_301_length_463_cov_0.684524

Length=463

***** No hits found *****

Lambda K H a alpha

0.318 0.134 0.401 0.792 4.96

Gapped

Lambda K H a alpha sigma

0.267 0.0410 0.140 1.90 42.6 43.6

Effective search space used: 18972976

**Query=** NODE_302_length_462_cov_0.749254

Length=462

***** No hits found *****

Lambda K H a alpha

0.318 0.134 0.401 0.792 4.96

Gapped

Lambda K H a alpha sigma

0.267 0.0410 0.140 1.90 42.6 43.6

Effective search space used: 18972976

**Query=** NODE_303_length_461_cov_0.586826

Length=461

***** No hits found *****

Lambda K H a alpha

0.318 0.134 0.401 0.792 4.96

Gapped

Lambda K H a alpha sigma

0.267 0.0410 0.140 1.90 42.6 43.6

Effective search space used: 18757374

**Query=** NODE_304_length_460_cov_0.718563

Length=460

***** No hits found *****

Lambda K H a alpha

0.318 0.134 0.401 0.792 4.96

Gapped

Lambda K H a alpha sigma

0.267 0.0410 0.140 1.90 42.6 43.6

Effective search space used: 18757374

**Query=** NODE_305_length_459_cov_0.353293

Length=459

***** No hits found *****

Lambda K H a alpha

0.318 0.134 0.401 0.792 4.96

Gapped

Lambda K H a alpha sigma

0.267 0.0410 0.140 1.90 42.6 43.6

Effective search space used: 18757374

**Query=** NODE_306_length_459_cov_0.57958

Length=459

***** No hits found *****

Lambda K H a alpha

0.318 0.134 0.401 0.792 4.96

Gapped

Lambda K H a alpha sigma

0.267 0.0410 0.140 1.90 42.6 43.6

Effective search space used: 18757374

**Query=** NODE_307_length_460_cov_0.618619

Length=460

***** No hits found *****

Lambda K H a alpha

0.318 0.134 0.401 0.792 4.96

Gapped

Lambda K H a alpha sigma

0.267 0.0410 0.140 1.90 42.6 43.6

Effective search space used: 18757374

**Query=** NODE_308_length_460_cov_0.591592

Length=460

***** No hits found *****

Lambda K H a alpha

0.318 0.134 0.401 0.792 4.96

Gapped

Lambda K H a alpha sigma

0.267 0.0410 0.140 1.90 42.6 43.6

Effective search space used: 18757374

**Query=** NODE_309_length_459_cov_0.3003

Length=459

***** No hits found *****

Lambda K H a alpha

0.318 0.134 0.401 0.792 4.96

Gapped

Lambda K H a alpha sigma

0.267 0.0410 0.140 1.90 42.6 43.6

Effective search space used: 18757374

**Query=** NODE_310_length_456_cov_0.48048

Length=456

***** No hits found *****

Lambda K H a alpha

0.318 0.134 0.401 0.792 4.96

Gapped

Lambda K H a alpha sigma

0.267 0.0410 0.140 1.90 42.6 43.6

Effective search space used: 18541772

**Query=** NODE_311_length_458_cov_0.740964

Length=458

***** No hits found *****

Lambda K H a alpha

0.318 0.134 0.401 0.792 4.96

Gapped

Lambda K H a alpha sigma

0.267 0.0410 0.140 1.90 42.6 43.6

Effective search space used: 18541772

**Query=** NODE_312_length_458_cov_0.707831

Length=458

***** No hits found *****

Lambda K H a alpha

0.318 0.134 0.401 0.792 4.96

Gapped

Lambda K H a alpha sigma

0.267 0.0410 0.140 1.90 42.6 43.6

Effective search space used: 18541772

**Query=** NODE_313_length_458_cov_0.692771

Length=458

***** No hits found *****

Lambda K H a alpha

0.318 0.134 0.401 0.792 4.96

Gapped

Lambda K H a alpha sigma

0.267 0.0410 0.140 1.90 42.6 43.6

Effective search space used: 18541772

**Query=** NODE_314_length_459_cov_0.680723

Length=459

***** No hits found *****

Lambda K H a alpha

0.318 0.134 0.401 0.792 4.96

Gapped

Lambda K H a alpha sigma

0.267 0.0410 0.140 1.90 42.6 43.6

Effective search space used: 18757374

**Query=** NODE_315_length_459_cov_0.972892

Length=459

***** No hits found *****

Lambda K H a alpha

0.318 0.134 0.401 0.792 4.96

Gapped

Lambda K H a alpha sigma

0.267 0.0410 0.140 1.90 42.6 43.6

Effective search space used: 18757374

**Query=** NODE_316_length_459_cov_0.906627

Length=459

***** No hits found *****

Lambda K H a alpha

0.318 0.134 0.401 0.792 4.96

Gapped

Lambda K H a alpha sigma

0.267 0.0410 0.140 1.90 42.6 43.6

Effective search space used: 18757374

**Query=** NODE_317_length_456_cov_0.592145

Length=456

Score E

Sequences producing significant alignments: (Bits) Value

XP_018179360 florfenicol exporter [Purpureocillium lilacinum] [25.8](file:///Users/JMBNew/Desktop/Articles%20en%20Cours/Article%20Diane/Bb_SR-11-14_output-blast_named.html#BL_ORD_ID:594) 0.36

OAQ90641 florfenicol exporter [Purpureocillium lilacinum] [25.8](file:///Users/JMBNew/Desktop/Articles%20en%20Cours/Article%20Diane/Bb_SR-11-14_output-blast_named.html#BL_ORD_ID:509) 0.36

OAQ83863 florfenicol exporter [Purpureocillium lilacinum] [25.4](file:///Users/JMBNew/Desktop/Articles%20en%20Cours/Article%20Diane/Bb_SR-11-14_output-blast_named.html#BL_ORD_ID:508) 0.39

> XP_018179360 florfenicol exporter [Purpureocillium lilacinum]

Length=564

Score = 25.8 bits (55), Expect = 0.36, Method: Composition-based stats.

Identities = 13/36 (36%), Positives = 17/36 (47%), Gaps = 2/36 (6%)

Frame = +3

Query 150 PPEVLRSRGGAGMWLK--RDDQHNPHHPPFPPGETR 251

PP V S + W + RD + NP PP P + R

Sbjct 260 PPSVNTSVTSSIQWRRKRRDLESNPDAPPLPEPKKR 295

> OAQ90641 florfenicol exporter [Purpureocillium lilacinum]

Length=564

Score = 25.8 bits (55), Expect = 0.36, Method: Composition-based stats.

Identities = 13/36 (36%), Positives = 17/36 (47%), Gaps = 2/36 (6%)

Frame = +3

Query 150 PPEVLRSRGGAGMWLK--RDDQHNPHHPPFPPGETR 251

PP V S + W + RD + NP PP P + R

Sbjct 260 PPSVNTSVTSSIQWRRKRRDLESNPDAPPLPEPKKR 295

> OAQ83863 florfenicol exporter [Purpureocillium lilacinum]

Length=539

Score = 25.4 bits (54), Expect = 0.39, Method: Composition-based stats.

Identities = 13/36 (36%), Positives = 17/36 (47%), Gaps = 2/36 (6%)

Frame = +3

Query 150 PPEVLRSRGGAGMWLK--RDDQHNPHHPPFPPGETR 251

PP V S + W + RD + NP PP P + R

Sbjct 260 PPSVNTSVTSSIQWRRKRRDLESNPDAPPLPEPKKR 295

Lambda K H a alpha

0.318 0.134 0.401 0.792 4.96

Gapped

Lambda K H a alpha sigma

0.267 0.0410 0.140 1.90 42.6 43.6

Effective search space used: 18541772

**Query=** NODE_318_length_458_cov_0.761329

Length=458

***** No hits found *****

Lambda K H a alpha

0.318 0.134 0.401 0.792 4.96

Gapped

Lambda K H a alpha sigma

0.267 0.0410 0.140 1.90 42.6 43.6

Effective search space used: 18541772

**Query=** NODE_319_length_458_cov_0.685801

Length=458

***** No hits found *****

Lambda K H a alpha

0.318 0.134 0.401 0.792 4.96

Gapped

Lambda K H a alpha sigma

0.267 0.0410 0.140 1.90 42.6 43.6

Effective search space used: 18541772

**Query=** NODE_320_length_456_cov_0.250755

Length=456

***** No hits found *****

Lambda K H a alpha

0.318 0.134 0.401 0.792 4.96

Gapped

Lambda K H a alpha sigma

0.267 0.0410 0.140 1.90 42.6 43.6

Effective search space used: 18541772

**Query=** NODE_321_length_454_cov_0.512121

Length=454

***** No hits found *****

Lambda K H a alpha

0.318 0.134 0.401 0.792 4.96

Gapped

Lambda K H a alpha sigma

0.267 0.0410 0.140 1.90 42.6 43.6

Effective search space used: 18326170

**Query=** NODE_322_length_457_cov_0.366667

Length=457

***** No hits found *****

Lambda K H a alpha

0.318 0.134 0.401 0.792 4.96

Gapped

Lambda K H a alpha sigma

0.267 0.0410 0.140 1.90 42.6 43.6

Effective search space used: 18541772

**Query=** NODE_323_length_457_cov_0.624242

Length=457

***** No hits found *****

Lambda K H a alpha

0.318 0.134 0.401 0.792 4.96

Gapped

Lambda K H a alpha sigma

0.267 0.0410 0.140 1.90 42.6 43.6

Effective search space used: 18541772

**Query=** NODE_324_length_457_cov_0.518182

Length=457

***** No hits found *****

Lambda K H a alpha

0.318 0.134 0.401 0.792 4.96

Gapped

Lambda K H a alpha sigma

0.267 0.0410 0.140 1.90 42.6 43.6

Effective search space used: 18541772

**Query=** NODE_325_length_456_cov_0.79697

Length=456

***** No hits found *****

Lambda K H a alpha

0.318 0.134 0.401 0.792 4.96

Gapped

Lambda K H a alpha sigma

0.267 0.0410 0.140 1.90 42.6 43.6

Effective search space used: 18541772

**Query=** NODE_326_length_457_cov_0.678788

Length=457

***** No hits found *****

Lambda K H a alpha

0.318 0.134 0.401 0.792 4.96

Gapped

Lambda K H a alpha sigma

0.267 0.0410 0.140 1.90 42.6 43.6

Effective search space used: 18541772

**Query=** NODE_327_length_457_cov_0.275758

Length=457

***** No hits found *****

Lambda K H a alpha

0.318 0.134 0.401 0.792 4.96

Gapped

Lambda K H a alpha sigma

0.267 0.0410 0.140 1.90 42.6 43.6

Effective search space used: 18541772

**Query=** NODE_328_length_456_cov_0.759878

Length=456

***** No hits found *****

Lambda K H a alpha

0.318 0.134 0.401 0.792 4.96

Gapped

Lambda K H a alpha sigma

0.267 0.0410 0.140 1.90 42.6 43.6

Effective search space used: 18541772

**Query=** NODE_329_length_454_cov_0.714286

Length=454

***** No hits found *****

Lambda K H a alpha

0.318 0.134 0.401 0.792 4.96

Gapped

Lambda K H a alpha sigma

0.267 0.0410 0.140 1.90 42.6 43.6

Effective search space used: 18326170

**Query=** NODE_330_length_456_cov_0.443769

Length=456

Score E

Sequences producing significant alignments: (Bits) Value

OV14_RS04905_-_26221503_translation chloramphenicol efflux MFS ... [25.0](file:///Users/JMBNew/Desktop/Articles%20en%20Cours/Article%20Diane/Bb_SR-11-14_output-blast_named.html#BL_ORD_ID:692) 0.50

> OV14_RS04905_-_26221503_translation chloramphenicol efflux MFS

transporter

Length=382

Score = 25.0 bits (53), Expect = 0.50, Method: Compositional matrix adjust.

Identities = 20/64 (31%), Positives = 24/64 (38%), Gaps = 1/64 (2%)

Frame = -3

Query 256 TPHRAPHTPSRRSW*-VADDAQHRTPQHGLFPPSTGNGVTLRAHRAPTEGFYDVLFKGQP 80

PHR P RR +ADD QHR P G H A + D KG+

Sbjct 86 DPHRQDEGPERRCGDALADDEQHRAEDEQRHPAGKAAGDAPERHEAFGKTGRDDHHKGRR 145

Query 79 FRFQ 68

+ Q

Sbjct 146 RKDQ 149

Lambda K H a alpha

0.318 0.134 0.401 0.792 4.96

Gapped

Lambda K H a alpha sigma

0.267 0.0410 0.140 1.90 42.6 43.6

Effective search space used: 18541772

**Query=** NODE_331_length_456_cov_0.428571

Length=456

***** No hits found *****

Lambda K H a alpha

0.318 0.134 0.401 0.792 4.96

Gapped

Lambda K H a alpha sigma

0.267 0.0410 0.140 1.90 42.6 43.6

Effective search space used: 18541772

**Query=** NODE_332_length_455_cov_0.527439

Length=455

***** No hits found *****

Lambda K H a alpha

0.318 0.134 0.401 0.792 4.96

Gapped

Lambda K H a alpha sigma

0.267 0.0410 0.140 1.90 42.6 43.6

Effective search space used: 18326170

**Query=** NODE_333_length_455_cov_0.698171

Length=455

***** No hits found *****

Lambda K H a alpha

0.318 0.134 0.401 0.792 4.96

Gapped

Lambda K H a alpha sigma

0.267 0.0410 0.140 1.90 42.6 43.6

Effective search space used: 18326170

**Query=** NODE_334_length_455_cov_0.637195

Length=455

***** No hits found *****

Lambda K H a alpha

0.318 0.134 0.401 0.792 4.96

Gapped

Lambda K H a alpha sigma

0.267 0.0410 0.140 1.90 42.6 43.6

Effective search space used: 18326170

**Query=** NODE_335_length_454_cov_0.412844

Length=454

***** No hits found *****

Lambda K H a alpha

0.318 0.134 0.401 0.792 4.96

Gapped

Lambda K H a alpha sigma

0.267 0.0410 0.140 1.90 42.6 43.6

Effective search space used: 18326170

**Query=** NODE_336_length_454_cov_0.675841

Length=454

***** No hits found *****

Lambda K H a alpha

0.318 0.134 0.401 0.792 4.96

Gapped

Lambda K H a alpha sigma

0.267 0.0410 0.140 1.90 42.6 43.6

Effective search space used: 18326170

**Query=** NODE_337_length_454_cov_0.681957

Length=454

Score E

Sequences producing significant alignments: (Bits) Value

ADB74682 major facilitator superfamily MFS_1 [Geodermatophilus ... [26.2](file:///Users/JMBNew/Desktop/Articles%20en%20Cours/Article%20Diane/Bb_SR-11-14_output-blast_named.html#BL_ORD_ID:62) 0.25

> ADB74682 major facilitator superfamily MFS_1 [Geodermatophilus

obscurus DSM 43160]

Length=425

Score = 26.2 bits (56), Expect = 0.25, Method: Compositional matrix adjust.

Identities = 12/34 (35%), Positives = 20/34 (59%), Gaps = 0/34 (0%)

Frame = -3

Query 269 PVIVHVALREHARGGGDMSPRCHHAVTGGGPSLS 168

PV+V +AL+ ++ G + +P VTG G L+

Sbjct 46 PVLVTLALKINSLVGSEQAPNSLALVTGTGSLLA 79

Lambda K H a alpha

0.318 0.134 0.401 0.792 4.96

Gapped

Lambda K H a alpha sigma

0.267 0.0410 0.140 1.90 42.6 43.6

Effective search space used: 18326170

**Query=** NODE_338_length_454_cov_0.513761

Length=454

***** No hits found *****

Lambda K H a alpha

0.318 0.134 0.401 0.792 4.96

Gapped

Lambda K H a alpha sigma

0.267 0.0410 0.140 1.90 42.6 43.6

Effective search space used: 18326170

**Query=** NODE_339_length_458_cov_0.782209

Length=458

***** No hits found *****

Lambda K H a alpha

0.318 0.134 0.401 0.792 4.96

Gapped

Lambda K H a alpha sigma

0.267 0.0410 0.140 1.90 42.6 43.6

Effective search space used: 18541772

**Query=** NODE_340_length_453_cov_0.503067

Length=453

***** No hits found *****

Lambda K H a alpha

0.318 0.134 0.401 0.792 4.96

Gapped

Lambda K H a alpha sigma

0.267 0.0410 0.140 1.90 42.6 43.6

Effective search space used: 18326170

**Query=** NODE_341_length_451_cov_0.676923

Length=451

***** No hits found *****

Lambda K H a alpha

0.318 0.134 0.401 0.792 4.96

Gapped

Lambda K H a alpha sigma

0.267 0.0410 0.140 1.90 42.6 43.6

Effective search space used: 18110568

**Query=** NODE_342_length_451_cov_0.658462

Length=451

***** No hits found *****

Lambda K H a alpha

0.318 0.134 0.401 0.792 4.96

Gapped

Lambda K H a alpha sigma

0.267 0.0410 0.140 1.90 42.6 43.6

Effective search space used: 18110568

**Query=** NODE_343_length_452_cov_0.415385

Length=452

***** No hits found *****

Lambda K H a alpha

0.318 0.134 0.401 0.792 4.96

Gapped

Lambda K H a alpha sigma

0.267 0.0410 0.140 1.90 42.6 43.6

Effective search space used: 18110568

**Query=** NODE_344_length_451_cov_0.42284

Length=451

***** No hits found *****

Lambda K H a alpha

0.318 0.134 0.401 0.792 4.96

Gapped

Lambda K H a alpha sigma

0.267 0.0410 0.140 1.90 42.6 43.6

Effective search space used: 18110568

**Query=** NODE_345_length_450_cov_0.773994

Length=450

***** No hits found *****

Lambda K H a alpha

0.318 0.134 0.401 0.792 4.96

Gapped

Lambda K H a alpha sigma

0.267 0.0410 0.140 1.90 42.6 43.6

Effective search space used: 18110568

**Query=** NODE_346_length_448_cov_0.467492

Length=448

***** No hits found *****

Lambda K H a alpha

0.318 0.134 0.401 0.792 4.96

Gapped

Lambda K H a alpha sigma

0.267 0.0410 0.140 1.90 42.6 43.6

Effective search space used: 17894966

**Query=** NODE_347_length_450_cov_0.588235

Length=450

***** No hits found *****

Lambda K H a alpha

0.318 0.134 0.401 0.792 4.96

Gapped

Lambda K H a alpha sigma

0.267 0.0410 0.140 1.90 42.6 43.6

Effective search space used: 18110568

**Query=** NODE_348_length_448_cov_0.721362

Length=448

***** No hits found *****

Lambda K H a alpha

0.318 0.134 0.401 0.792 4.96

Gapped

Lambda K H a alpha sigma

0.267 0.0410 0.140 1.90 42.6 43.6

Effective search space used: 17894966

**Query=** NODE_349_length_449_cov_0.78882

Length=449

***** No hits found *****

Lambda K H a alpha

0.318 0.134 0.401 0.792 4.96

Gapped

Lambda K H a alpha sigma

0.267 0.0410 0.140 1.90 42.6 43.6

Effective search space used: 17894966

**Query=** NODE_350_length_448_cov_0.71028

Length=448

***** No hits found *****

Lambda K H a alpha

0.318 0.134 0.401 0.792 4.96

Gapped

Lambda K H a alpha sigma

0.267 0.0410 0.140 1.90 42.6 43.6

Effective search space used: 17894966

**Query=** NODE_351_length_448_cov_0.563863

Length=448

***** No hits found *****

Lambda K H a alpha

0.318 0.134 0.401 0.792 4.96

Gapped

Lambda K H a alpha sigma

0.267 0.0410 0.140 1.90 42.6 43.6

Effective search space used: 17894966

**Query=** NODE_352_length_448_cov_0.576324

Length=448

***** No hits found *****

Lambda K H a alpha

0.318 0.134 0.401 0.792 4.96

Gapped

Lambda K H a alpha sigma

0.267 0.0410 0.140 1.90 42.6 43.6

Effective search space used: 17894966

**Query=** NODE_353_length_448_cov_0.52648

Length=448

***** No hits found *****

Lambda K H a alpha

0.316 0.131 0.430 0.792 4.96

Gapped

Lambda K H a alpha sigma

0.267 0.0410 0.140 1.90 42.6 43.6

Effective search space used: 17894966

**Query=** NODE_354_length_448_cov_0.679128

Length=448

***** No hits found *****

Lambda K H a alpha

0.318 0.134 0.401 0.792 4.96

Gapped

Lambda K H a alpha sigma

0.267 0.0410 0.140 1.90 42.6 43.6

Effective search space used: 17894966

**Query=** NODE_355_length_448_cov_0.386293

Length=448

***** No hits found *****

Lambda K H a alpha

0.318 0.134 0.401 0.792 4.96

Gapped

Lambda K H a alpha sigma

0.267 0.0410 0.140 1.90 42.6 43.6

Effective search space used: 17894966

**Query=** NODE_356_length_447_cov_0.29375

Length=447

***** No hits found *****

Lambda K H a alpha

0.318 0.134 0.401 0.792 4.96

Gapped

Lambda K H a alpha sigma

0.267 0.0410 0.140 1.90 42.6 43.6

Effective search space used: 17894966

**Query=** NODE_357_length_447_cov_0.6125

Length=447

Score E

Sequences producing significant alignments: (Bits) Value

ARA90579 phenicol and oxazolidinone resistance protein [Staphyl... [39.3](file:///Users/JMBNew/Desktop/Articles%20en%20Cours/Article%20Diane/Bb_SR-11-14_output-blast_named.html#BL_ORD_ID:288) 1e-05

AQW34744 phenicol and oxazolidinone resistance protein [Staphyl... [39.3](file:///Users/JMBNew/Desktop/Articles%20en%20Cours/Article%20Diane/Bb_SR-11-14_output-blast_named.html#BL_ORD_ID:278) 1e-05

AQW34730 phenicol and oxazolidinone resistance protein [Staphyl... [39.3](file:///Users/JMBNew/Desktop/Articles%20en%20Cours/Article%20Diane/Bb_SR-11-14_output-blast_named.html#BL_ORD_ID:274) 1e-05

AQW34667 ABC transporter ATP-binding protein (plasmid) [Staphyl... [39.3](file:///Users/JMBNew/Desktop/Articles%20en%20Cours/Article%20Diane/Bb_SR-11-14_output-blast_named.html#BL_ORD_ID:259) 1e-05

AQW34661 phenicol and oxazolidinone resistance protein [Staphyl... [39.3](file:///Users/JMBNew/Desktop/Articles%20en%20Cours/Article%20Diane/Bb_SR-11-14_output-blast_named.html#BL_ORD_ID:256) 1e-05

AQW34617 OptrA (plasmid) [Staphylococcus sciuri] [39.3](file:///Users/JMBNew/Desktop/Articles%20en%20Cours/Article%20Diane/Bb_SR-11-14_output-blast_named.html#BL_ORD_ID:249) 1e-05

AQW34612 phenicol and oxazolidinone resistance protein [Staphyl... [39.3](file:///Users/JMBNew/Desktop/Articles%20en%20Cours/Article%20Diane/Bb_SR-11-14_output-blast_named.html#BL_ORD_ID:246) 1e-05

AQW34601 phenicol and oxazolidinone resistance protein [Staphyl... [39.3](file:///Users/JMBNew/Desktop/Articles%20en%20Cours/Article%20Diane/Bb_SR-11-14_output-blast_named.html#BL_ORD_ID:245) 1e-05

AQW34567 ABC transporter ATP-binding protein [Staphylococcus sc... [39.3](file:///Users/JMBNew/Desktop/Articles%20en%20Cours/Article%20Diane/Bb_SR-11-14_output-blast_named.html#BL_ORD_ID:238) 1e-05

AKU20095 putative ABC transporter [Enterococcus faecium] [38.5](file:///Users/JMBNew/Desktop/Articles%20en%20Cours/Article%20Diane/Bb_SR-11-14_output-blast_named.html#BL_ORD_ID:204) 2e-05

AQW34663 ABC transporter ATP-binding protein [Staphylococcus sc... [38.5](file:///Users/JMBNew/Desktop/Articles%20en%20Cours/Article%20Diane/Bb_SR-11-14_output-blast_named.html#BL_ORD_ID:258) 2e-05

AQW34614 ABC transporter ATP-binding protein [Staphylococcus sc... [37.0](file:///Users/JMBNew/Desktop/Articles%20en%20Cours/Article%20Diane/Bb_SR-11-14_output-blast_named.html#BL_ORD_ID:248) 6e-05

AQW34662 multidrug ABC transporter ATP-binding protein [Staphyl... [33.9](file:///Users/JMBNew/Desktop/Articles%20en%20Cours/Article%20Diane/Bb_SR-11-14_output-blast_named.html#BL_ORD_ID:257) 6e-04

AQW34613 multidrug ABC transporter ATP-binding protein [Staphyl... [33.9](file:///Users/JMBNew/Desktop/Articles%20en%20Cours/Article%20Diane/Bb_SR-11-14_output-blast_named.html#BL_ORD_ID:247) 6e-04

YP_004800000 putative multi-drug ABC transporter permease/ATPas... [32.3](file:///Users/JMBNew/Desktop/Articles%20en%20Cours/Article%20Diane/Bb_SR-11-14_output-blast_named.html#BL_ORD_ID:619) 0.002

AEM66534 putative multi-drug ABC transporter permease/ATPase (p... [32.3](file:///Users/JMBNew/Desktop/Articles%20en%20Cours/Article%20Diane/Bb_SR-11-14_output-blast_named.html#BL_ORD_ID:79) 0.002

YP_004810315 putative multi-drug ABC transporter permease/ATPas... [32.3](file:///Users/JMBNew/Desktop/Articles%20en%20Cours/Article%20Diane/Bb_SR-11-14_output-blast_named.html#BL_ORD_ID:623) 0.002

AEM66524 putative multi-drug ABC transporter permease/ATPase (p... [32.3](file:///Users/JMBNew/Desktop/Articles%20en%20Cours/Article%20Diane/Bb_SR-11-14_output-blast_named.html#BL_ORD_ID:74) 0.002

CEP78658 23S rRNA (adenine(2503)-C(8))-methyltransferase [Deflu... [26.2](file:///Users/JMBNew/Desktop/Articles%20en%20Cours/Article%20Diane/Bb_SR-11-14_output-blast_named.html#BL_ORD_ID:400) 0.22

> ARA90579 phenicol and oxazolidinone resistance protein [Staphylococcus

sciuri]

Length=655

Score = 39.3 bits (90), Expect = 1e-05, Method: Composition-based stats.

Identities = 18/64 (28%), Positives = 35/64 (55%), Gaps = 1/64 (2%)

Frame = +1

Query 169 AEQSAELAGQPAREAAQAMLRRVGLGERLHHYPRT-LSGGEQQRVSLARAFVVQPDLLFA 345

+E+ L G ++ + M+R +G E P + SGG++ +++ + + +PD+L

Sbjct 147 SERYMALGGLTYQKEYETMIRSMGFTEADDKKPISEFSGGQRTKIAFIKILLTKPDILLL 206

Query 346 DEPT 357

DEPT

Sbjct 207 DEPT 210

Score = 29.6 bits (65), Expect = 0.017, Method: Composition-based stats.

Identities = 12/29 (41%), Positives = 20/29 (69%), Gaps = 0/29 (0%)

Frame = +1

Query 271 TLSGGEQQRVSLARAFVVQPDLLFADEPT 357

+LSGGE+ R++L + + ++L DEPT

Sbjct 462 SLSGGEKVRLTLCKLLYKRTNVLILDEPT 490

> AQW34744 phenicol and oxazolidinone resistance protein [Staphylococcus

sciuri]

Length=655

Score = 39.3 bits (90), Expect = 1e-05, Method: Composition-based stats.

Identities = 18/64 (28%), Positives = 35/64 (55%), Gaps = 1/64 (2%)

Frame = +1

Query 169 AEQSAELAGQPAREAAQAMLRRVGLGERLHHYPRT-LSGGEQQRVSLARAFVVQPDLLFA 345

+E+ L G ++ + M+R +G E P + SGG++ +++ + + +PD+L

Sbjct 147 SERYMALGGLTYQKEYETMIRSMGFTEADDKKPISEFSGGQRTKIAFIKILLTKPDILLL 206

Query 346 DEPT 357

DEPT

Sbjct 207 DEPT 210

Score = 29.6 bits (65), Expect = 0.017, Method: Composition-based stats.

Identities = 12/29 (41%), Positives = 20/29 (69%), Gaps = 0/29 (0%)

Frame = +1

Query 271 TLSGGEQQRVSLARAFVVQPDLLFADEPT 357

+LSGGE+ R++L + + ++L DEPT

Sbjct 462 SLSGGEKVRLTLCKLLYKRTNVLILDEPT 490

> AQW34730 phenicol and oxazolidinone resistance protein [Staphylococcus

sciuri]

Length=655

Score = 39.3 bits (90), Expect = 1e-05, Method: Composition-based stats.

Identities = 18/64 (28%), Positives = 35/64 (55%), Gaps = 1/64 (2%)

Frame = +1

Query 169 AEQSAELAGQPAREAAQAMLRRVGLGERLHHYPRT-LSGGEQQRVSLARAFVVQPDLLFA 345

+E+ L G ++ + M+R +G E P + SGG++ +++ + + +PD+L

Sbjct 147 SERYMALGGLTYQKEYETMIRSMGFTEADDKKPISEFSGGQRTKIAFIKILLTKPDILLL 206

Query 346 DEPT 357

DEPT

Sbjct 207 DEPT 210

Score = 29.6 bits (65), Expect = 0.017, Method: Composition-based stats.

Identities = 12/29 (41%), Positives = 20/29 (69%), Gaps = 0/29 (0%)

Frame = +1

Query 271 TLSGGEQQRVSLARAFVVQPDLLFADEPT 357

+LSGGE+ R++L + + ++L DEPT

Sbjct 462 SLSGGEKVRLTLCKLLYKRTNVLILDEPT 490

> AQW34667 ABC transporter ATP-binding protein (plasmid) [Staphylococcus

sciuri]

Length=655

Score = 39.3 bits (90), Expect = 1e-05, Method: Composition-based stats.

Identities = 18/64 (28%), Positives = 35/64 (55%), Gaps = 1/64 (2%)

Frame = +1

Query 169 AEQSAELAGQPAREAAQAMLRRVGLGERLHHYPRT-LSGGEQQRVSLARAFVVQPDLLFA 345

+E+ L G ++ + M+R +G E P + SGG++ +++ + + +PD+L

Sbjct 147 SERYMALGGLTYQKEYETMIRSMGFTEADDKKPISEFSGGQRTKIAFIKILLTKPDILLL 206

Query 346 DEPT 357

DEPT

Sbjct 207 DEPT 210

Score = 29.6 bits (65), Expect = 0.017, Method: Composition-based stats.

Identities = 12/29 (41%), Positives = 20/29 (69%), Gaps = 0/29 (0%)

Frame = +1

Query 271 TLSGGEQQRVSLARAFVVQPDLLFADEPT 357

+LSGGE+ R++L + + ++L DEPT

Sbjct 462 SLSGGEKVRLTLCKLLYKRTNVLILDEPT 490

> AQW34661 phenicol and oxazolidinone resistance protein [Staphylococcus

sciuri]

Length=655

Score = 39.3 bits (90), Expect = 1e-05, Method: Composition-based stats.

Identities = 18/64 (28%), Positives = 35/64 (55%), Gaps = 1/64 (2%)

Frame = +1

Query 169 AEQSAELAGQPAREAAQAMLRRVGLGERLHHYPRT-LSGGEQQRVSLARAFVVQPDLLFA 345

+E+ L G ++ + M+R +G E P + SGG++ +++ + + +PD+L

Sbjct 147 SERYMALGGLTYQKEYETMIRSMGFTEADDKKPISEFSGGQRTKIAFIKILLTKPDILLL 206

Query 346 DEPT 357

DEPT

Sbjct 207 DEPT 210

Score = 29.6 bits (65), Expect = 0.017, Method: Composition-based stats.

Identities = 12/29 (41%), Positives = 20/29 (69%), Gaps = 0/29 (0%)

Frame = +1

Query 271 TLSGGEQQRVSLARAFVVQPDLLFADEPT 357

+LSGGE+ R++L + + ++L DEPT

Sbjct 462 SLSGGEKVRLTLCKLLYKRTNVLILDEPT 490

> AQW34617 OptrA (plasmid) [Staphylococcus sciuri]

Length=655

Score = 39.3 bits (90), Expect = 1e-05, Method: Composition-based stats.

Identities = 18/64 (28%), Positives = 35/64 (55%), Gaps = 1/64 (2%)

Frame = +1

Query 169 AEQSAELAGQPAREAAQAMLRRVGLGERLHHYPRT-LSGGEQQRVSLARAFVVQPDLLFA 345

+E+ L G ++ + M+R +G E P + SGG++ +++ + + +PD+L

Sbjct 147 SERYMALGGLTYQKEYETMIRSMGFTEADDKKPISEFSGGQRTKIAFIKILLTKPDILLL 206

Query 346 DEPT 357

DEPT

Sbjct 207 DEPT 210

Score = 29.6 bits (65), Expect = 0.017, Method: Composition-based stats.

Identities = 12/29 (41%), Positives = 20/29 (69%), Gaps = 0/29 (0%)

Frame = +1

Query 271 TLSGGEQQRVSLARAFVVQPDLLFADEPT 357

+LSGGE+ R++L + + ++L DEPT

Sbjct 462 SLSGGEKVRLTLCKLLYKRTNVLILDEPT 490

> AQW34612 phenicol and oxazolidinone resistance protein [Staphylococcus

sciuri]

Length=655

Score = 39.3 bits (90), Expect = 1e-05, Method: Composition-based stats.

Identities = 18/64 (28%), Positives = 35/64 (55%), Gaps = 1/64 (2%)

Frame = +1

Query 169 AEQSAELAGQPAREAAQAMLRRVGLGERLHHYPRT-LSGGEQQRVSLARAFVVQPDLLFA 345

+E+ L G ++ + M+R +G E P + SGG++ +++ + + +PD+L

Sbjct 147 SERYMALGGLTYQKEYETMIRSMGFTEADDKKPISEFSGGQRTKIAFIKILLTKPDILLL 206

Query 346 DEPT 357

DEPT

Sbjct 207 DEPT 210

Score = 29.6 bits (65), Expect = 0.017, Method: Composition-based stats.

Identities = 12/29 (41%), Positives = 20/29 (69%), Gaps = 0/29 (0%)

Frame = +1

Query 271 TLSGGEQQRVSLARAFVVQPDLLFADEPT 357

+LSGGE+ R++L + + ++L DEPT

Sbjct 462 SLSGGEKVRLTLCKLLYKRTNVLILDEPT 490

> AQW34601 phenicol and oxazolidinone resistance protein [Staphylococcus

sciuri]

Length=655

Score = 39.3 bits (90), Expect = 1e-05, Method: Composition-based stats.

Identities = 18/64 (28%), Positives = 35/64 (55%), Gaps = 1/64 (2%)

Frame = +1

Query 169 AEQSAELAGQPAREAAQAMLRRVGLGERLHHYPRT-LSGGEQQRVSLARAFVVQPDLLFA 345

+E+ L G ++ + M+R +G E P + SGG++ +++ + + +PD+L

Sbjct 147 SERYMALGGLTYQKEYETMIRSMGFTEADDKKPISEFSGGQRTKIAFIKILLTKPDILLL 206

Query 346 DEPT 357

DEPT

Sbjct 207 DEPT 210

Score = 29.6 bits (65), Expect = 0.017, Method: Composition-based stats.

Identities = 12/29 (41%), Positives = 20/29 (69%), Gaps = 0/29 (0%)

Frame = +1

Query 271 TLSGGEQQRVSLARAFVVQPDLLFADEPT 357

+LSGGE+ R++L + + ++L DEPT

Sbjct 462 SLSGGEKVRLTLCKLLYKRTNVLILDEPT 490

> AQW34567 ABC transporter ATP-binding protein [Staphylococcus

sciuri]

Length=655

Score = 39.3 bits (90), Expect = 1e-05, Method: Composition-based stats.

Identities = 18/64 (28%), Positives = 35/64 (55%), Gaps = 1/64 (2%)

Frame = +1

Query 169 AEQSAELAGQPAREAAQAMLRRVGLGERLHHYPRT-LSGGEQQRVSLARAFVVQPDLLFA 345

+E+ L G ++ + M+R +G E P + SGG++ +++ + + +PD+L

Sbjct 147 SERYMALGGLTYQKEYETMIRSMGFTEADDKKPISEFSGGQRTKIAFIKILLTKPDILLL 206

Query 346 DEPT 357

DEPT

Sbjct 207 DEPT 210

Score = 29.6 bits (65), Expect = 0.017, Method: Composition-based stats.

Identities = 12/29 (41%), Positives = 20/29 (69%), Gaps = 0/29 (0%)

Frame = +1

Query 271 TLSGGEQQRVSLARAFVVQPDLLFADEPT 357

+LSGGE+ R++L + + ++L DEPT

Sbjct 462 SLSGGEKVRLTLCKLLYKRTNVLILDEPT 490

> AKU20095 putative ABC transporter [Enterococcus faecium]

Length=542

Score = 38.5 bits (88), Expect = 2e-05, Method: Composition-based stats.

Identities = 16/43 (37%), Positives = 28/43 (65%), Gaps = 1/43 (2%)

Frame = +1

Query 232 RVGLGERLHHYP-RTLSGGEQQRVSLARAFVVQPDLLFADEPT 357

++ + L+H P R LSGG++ +++ AR +P++L DEPT

Sbjct 144 KMSIDAELYHRPMRELSGGQKSKMAFARLLYSKPEILLLDEPT 186

Score = 32.3 bits (72), Expect = 0.002, Method: Composition-based stats.

Identities = 17/39 (44%), Positives = 23/39 (59%), Gaps = 4/39 (10%)

Frame = +1

Query 274 LSGGEQQRVSLARAFVVQPDLLFADEPTPVSFTHLTPAT 390

LS GE+ RV+L + + + +LL DEPT HL P T

Sbjct 446 LSPGEKARVALCKILLQKANLLILDEPT----NHLDPET 480

> AQW34663 ABC transporter ATP-binding protein [Staphylococcus

sciuri]

Length=582

Score = 38.5 bits (88), Expect = 2e-05, Method: Composition-based stats.

Identities = 23/62 (37%), Positives = 29/62 (47%), Gaps = 11/62 (18%)

Frame = +1

Query 205 REAAQAMLRRVGLGERLHHYP-----------RTLSGGEQQRVSLARAFVVQPDLLFADE 351

RE A L VG L H+ RTLS G++Q +S ARA P++L DE

Sbjct 446 REKAVESLNAVGGDRVLEHFKKGIDEPVIERGRTLSSGQRQIISFARALAFDPEILILDE 505

Query 352 PT 357

T

Sbjct 506 AT 507

> AQW34614 ABC transporter ATP-binding protein [Staphylococcus

sciuri]

Length=582

Score = 37.0 bits (84), Expect = 6e-05, Method: Composition-based stats.

Identities = 17/40 (43%), Positives = 24/40 (60%), Gaps = 0/40 (0%)

Frame = +1

Query 238 GLGERLHHYPRTLSGGEQQRVSLARAFVVQPDLLFADEPT 357

G+ E + RTLS G++Q +S ARA P++L DE T

Sbjct 468 GIDEPVIERGRTLSSGQRQIISFARALAFDPEILILDEAT 507

> AQW34662 multidrug ABC transporter ATP-binding protein [Staphylococcus

sciuri]

Length=579

Score = 33.9 bits (76), Expect = 6e-04, Method: Composition-based stats.

Identities = 11/26 (42%), Positives = 21/26 (81%), Gaps = 0/26 (0%)

Frame = +1

Query 274 LSGGEQQRVSLARAFVVQPDLLFADE 351

+SGG++QR+++ARA ++ P +L D+

Sbjct 473 ISGGQKQRIAIARALIMDPKILILDD 498

> AQW34613 multidrug ABC transporter ATP-binding protein [Staphylococcus

sciuri]

Length=579

Score = 33.9 bits (76), Expect = 6e-04, Method: Composition-based stats.

Identities = 11/26 (42%), Positives = 21/26 (81%), Gaps = 0/26 (0%)

Frame = +1

Query 274 LSGGEQQRVSLARAFVVQPDLLFADE 351

+SGG++QR+++ARA ++ P +L D+

Sbjct 473 ISGGQKQRIAIARALIMDPKILILDD 498

> YP_004800000 putative multi-drug ABC transporter permease/ATPase

(plasmid) [Riemerella anatipestifer]

Length=373

Score = 32.3 bits (72), Expect = 0.002, Method: Compositional matrix adjust.

Identities = 14/41 (34%), Positives = 23/41 (56%), Gaps = 0/41 (0%)

Frame = +1

Query 235 VGLGERLHHYPRTLSGGEQQRVSLARAFVVQPDLLFADEPT 357

+GL ++ +SGG++QR+ +ARA P + DE T

Sbjct 257 LGLNTKIGASGNGISGGQKQRILIARAVYKNPQFILFDEAT 297

> AEM66534 putative multi-drug ABC transporter permease/ATPase

(plasmid) [Riemerella anatipestifer]

Length=373

Score = 32.3 bits (72), Expect = 0.002, Method: Compositional matrix adjust.

Identities = 14/41 (34%), Positives = 23/41 (56%), Gaps = 0/41 (0%)

Frame = +1

Query 235 VGLGERLHHYPRTLSGGEQQRVSLARAFVVQPDLLFADEPT 357

+GL ++ +SGG++QR+ +ARA P + DE T

Sbjct 257 LGLNTKIGASGNGISGGQKQRILIARAVYKNPQFILFDEAT 297

> YP_004810315 putative multi-drug ABC transporter permease/ATPase

(plasmid) [Riemerella anatipestifer]

Length=373

Score = 32.3 bits (72), Expect = 0.002, Method: Compositional matrix adjust.

Identities = 14/41 (34%), Positives = 23/41 (56%), Gaps = 0/41 (0%)

Frame = +1

Query 235 VGLGERLHHYPRTLSGGEQQRVSLARAFVVQPDLLFADEPT 357

+GL ++ +SGG++QR+ +ARA P + DE T

Sbjct 257 LGLNTKIGASGNGISGGQKQRILIARAVYKNPQFILFDEAT 297

> AEM66524 putative multi-drug ABC transporter permease/ATPase

(plasmid) [Riemerella anatipestifer]

Length=373

Score = 32.3 bits (72), Expect = 0.002, Method: Compositional matrix adjust.

Identities = 14/41 (34%), Positives = 23/41 (56%), Gaps = 0/41 (0%)

Frame = +1

Query 235 VGLGERLHHYPRTLSGGEQQRVSLARAFVVQPDLLFADEPT 357

+GL ++ +SGG++QR+ +ARA P + DE T

Sbjct 257 LGLNTKIGASGNGISGGQKQRILIARAVYKNPQFILFDEAT 297

> CEP78658 23S rRNA (adenine(2503)-C(8))-methyltransferase [Defluviitoga

tunisiensis]

Length=346

Score = 26.2 bits (56), Expect = 0.22, Method: Compositional matrix adjust.

Identities = 9/21 (43%), Positives = 13/21 (62%), Gaps = 0/21 (0%)

Frame = -2

Query 230 RSIACAASRAGWPASSALCSA 168

R+IAC +++ G P A CS

Sbjct 103 RTIACISTQVGCPLKCAFCST 123

Lambda K H a alpha

0.318 0.134 0.401 0.792 4.96

Gapped

Lambda K H a alpha sigma

0.267 0.0410 0.140 1.90 42.6 43.6

Effective search space used: 17894966

**Query=** NODE_358_length_447_cov_0.715625

Length=447

***** No hits found *****

Lambda K H a alpha

0.318 0.134 0.401 0.792 4.96

Gapped

Lambda K H a alpha sigma

0.267 0.0410 0.140 1.90 42.6 43.6

Effective search space used: 17894966

**Query=** NODE_359_length_446_cov_0.5125

Length=446

***** No hits found *****

Lambda K H a alpha

0.318 0.134 0.401 0.792 4.96

Gapped

Lambda K H a alpha sigma

0.267 0.0410 0.140 1.90 42.6 43.6

Effective search space used: 17679364

**Query=** NODE_360_length_447_cov_0.74375

Length=447

***** No hits found *****

Lambda K H a alpha

0.318 0.134 0.401 0.792 4.96

Gapped

Lambda K H a alpha sigma

0.267 0.0410 0.140 1.90 42.6 43.6

Effective search space used: 17894966

**Query=** NODE_361_length_447_cov_0.478125

Length=447

***** No hits found *****

Lambda K H a alpha

0.318 0.134 0.401 0.792 4.96

Gapped

Lambda K H a alpha sigma

0.267 0.0410 0.140 1.90 42.6 43.6

Effective search space used: 17894966

**Query=** NODE_362_length_446_cov_0.498433

Length=446

***** No hits found *****

Lambda K H a alpha

0.318 0.134 0.401 0.792 4.96

Gapped

Lambda K H a alpha sigma

0.267 0.0410 0.140 1.90 42.6 43.6

Effective search space used: 17679364

**Query=** NODE_363_length_445_cov_0.54717

Length=445

Score E

Sequences producing significant alignments: (Bits) Value

ACU36899 major facilitator superfamily MFS_1 [Actinosynnema mir... [24.3](file:///Users/JMBNew/Desktop/Articles%20en%20Cours/Article%20Diane/Bb_SR-11-14_output-blast_named.html#BL_ORD_ID:57) 0.91

> ACU36899 major facilitator superfamily MFS_1 [Actinosynnema mirum

DSM 43827]

Length=421

Score = 24.3 bits (51), Expect = 0.91, Method: Compositional matrix adjust.

Identities = 15/44 (34%), Positives = 20/44 (45%), Gaps = 0/44 (0%)

Frame = -1

Query 316 SVTGVSGGLGALMWCKGTGVTACVCPQWNLLPRTVPAERPCMAK 185

S T G L L G+ A + P + +LP VP ER +A

Sbjct 117 STTSSFGLLIVLYIGYQIGLNALLAPLYAVLPDRVPTERRGLAS 160

Lambda K H a alpha

0.318 0.134 0.401 0.792 4.96

Gapped

Lambda K H a alpha sigma

0.267 0.0410 0.140 1.90 42.6 43.6

Effective search space used: 17679364

**Query=** NODE_364_length_445_cov_0.732704

Length=445

***** No hits found *****

Lambda K H a alpha

0.318 0.134 0.401 0.792 4.96

Gapped

Lambda K H a alpha sigma

0.267 0.0410 0.140 1.90 42.6 43.6

Effective search space used: 17679364

**Query=** NODE_365_length_445_cov_0.742138

Length=445

***** No hits found *****

Lambda K H a alpha

0.318 0.134 0.401 0.792 4.96

Gapped

Lambda K H a alpha sigma

0.267 0.0410 0.140 1.90 42.6 43.6

Effective search space used: 17679364

**Query=** NODE_366_length_445_cov_0.924528

Length=445

***** No hits found *****

Lambda K H a alpha

0.318 0.134 0.401 0.792 4.96

Gapped

Lambda K H a alpha sigma

0.267 0.0410 0.140 1.90 42.6 43.6

Effective search space used: 17679364

**Query=** NODE_367_length_445_cov_0.710692

Length=445

***** No hits found *****

Lambda K H a alpha

0.318 0.134 0.401 0.792 4.96

Gapped

Lambda K H a alpha sigma

0.267 0.0410 0.140 1.90 42.6 43.6

Effective search space used: 17679364

**Query=** NODE_368_length_445_cov_0.704403

Length=445

***** No hits found *****

Lambda K H a alpha

0.318 0.134 0.401 0.792 4.96

Gapped

Lambda K H a alpha sigma

0.267 0.0410 0.140 1.90 42.6 43.6

Effective search space used: 17679364

**Query=** NODE_369_length_444_cov_0.665615

Length=444

***** No hits found *****

Lambda K H a alpha

0.318 0.134 0.401 0.792 4.96

Gapped

Lambda K H a alpha sigma

0.267 0.0410 0.140 1.90 42.6 43.6

Effective search space used: 17679364

**Query=** NODE_370_length_444_cov_0.665615

Length=444

Score E

Sequences producing significant alignments: (Bits) Value

AAO35792 florfenicol resistance protein [Clostridium tetani E88] [25.4](file:///Users/JMBNew/Desktop/Articles%20en%20Cours/Article%20Diane/Bb_SR-11-14_output-blast_named.html#BL_ORD_ID:11) 0.44

> AAO35792 florfenicol resistance protein [Clostridium tetani E88]

Length=349

Score = 25.4 bits (54), Expect = 0.44, Method: Compositional matrix adjust.

Identities = 10/34 (29%), Positives = 16/34 (47%), Gaps = 0/34 (0%)

Frame = -3

Query 421 TAAGGVLRRASHAGPTGQGVGGLSHVGARVGNAL 320

+ GG++R SH GQ + G R+ N +

Sbjct 122 STIGGIVRSLSHGEILGQVLKAQEETGERISNIV 155

Lambda K H a alpha

0.318 0.134 0.401 0.792 4.96

Gapped

Lambda K H a alpha sigma

0.267 0.0410 0.140 1.90 42.6 43.6

Effective search space used: 17679364

**Query=** NODE_371_length_444_cov_0.542587

Length=444

***** No hits found *****

Lambda K H a alpha

0.318 0.134 0.401 0.792 4.96

Gapped

Lambda K H a alpha sigma

0.267 0.0410 0.140 1.90 42.6 43.6

Effective search space used: 17679364

**Query=** NODE_372_length_444_cov_0.403785

Length=444

***** No hits found *****

Lambda K H a alpha

0.318 0.134 0.401 0.792 4.96

Gapped

Lambda K H a alpha sigma

0.267 0.0410 0.140 1.90 42.6 43.6

Effective search space used: 17679364

**Query=** NODE_373_length_443_cov_0.705696

Length=443

***** No hits found *****

Lambda K H a alpha

0.318 0.134 0.401 0.792 4.96

Gapped

Lambda K H a alpha sigma

0.267 0.0410 0.140 1.90 42.6 43.6

Effective search space used: 17736928

**Query=** NODE_374_length_442_cov_0.655063

Length=442

***** No hits found *****

Lambda K H a alpha

0.318 0.134 0.401 0.792 4.96

Gapped

Lambda K H a alpha sigma

0.267 0.0410 0.140 1.90 42.6 43.6

Effective search space used: 17736928

**Query=** NODE_375_length_443_cov_0.664557

Length=443

***** No hits found *****

Lambda K H a alpha

0.318 0.134 0.401 0.792 4.96

Gapped

Lambda K H a alpha sigma

0.267 0.0410 0.140 1.90 42.6 43.6

Effective search space used: 17736928

**Query=** NODE_376_length_442_cov_0.755556

Length=442

***** No hits found *****

Lambda K H a alpha

0.318 0.134 0.401 0.792 4.96

Gapped

Lambda K H a alpha sigma

0.267 0.0410 0.140 1.90 42.6 43.6

Effective search space used: 17736928

**Query=** NODE_377_length_442_cov_0.688889

Length=442

***** No hits found *****

Lambda K H a alpha

0.318 0.134 0.401 0.792 4.96

Gapped

Lambda K H a alpha sigma

0.267 0.0410 0.140 1.90 42.6 43.6

Effective search space used: 17736928

**Query=** NODE_378_length_441_cov_0.796178

Length=441

Score E

Sequences producing significant alignments: (Bits) Value

XP_002488834 florfenicol exporter, putative [Talaromyces stipit... [24.3](file:///Users/JMBNew/Desktop/Articles%20en%20Cours/Article%20Diane/Bb_SR-11-14_output-blast_named.html#BL_ORD_ID:587) 0.91

XP_002488833 florfenicol exporter, putative [Talaromyces stipit... [24.3](file:///Users/JMBNew/Desktop/Articles%20en%20Cours/Article%20Diane/Bb_SR-11-14_output-blast_named.html#BL_ORD_ID:586) 0.91

EED11424 florfenicol exporter, putative [Talaromyces stipitatus... [24.3](file:///Users/JMBNew/Desktop/Articles%20en%20Cours/Article%20Diane/Bb_SR-11-14_output-blast_named.html#BL_ORD_ID:421) 0.91

EED11423 florfenicol exporter, putative [Talaromyces stipitatus... [24.3](file:///Users/JMBNew/Desktop/Articles%20en%20Cours/Article%20Diane/Bb_SR-11-14_output-blast_named.html#BL_ORD_ID:420) 0.91

> XP_002488834 florfenicol exporter, putative [Talaromyces stipitatus

ATCC 10500]

Length=478

Score = 24.3 bits (51), Expect = 0.91, Method: Composition-based stats.

Identities = 14/26 (54%), Positives = 15/26 (58%), Gaps = 0/26 (0%)

Frame = +1

Query 97 GGRSLAIRLVDIGIPAPVIQAGIGHR 174

GG LA+ V IG PAPVI G R

Sbjct 439 GGSLLALVAVCIGFPAPVIIWIYGRR 464

> XP_002488833 florfenicol exporter, putative [Talaromyces stipitatus

ATCC 10500]

Length=478

Score = 24.3 bits (51), Expect = 0.91, Method: Composition-based stats.

Identities = 14/26 (54%), Positives = 15/26 (58%), Gaps = 0/26 (0%)

Frame = +1

Query 97 GGRSLAIRLVDIGIPAPVIQAGIGHR 174

GG LA+ V IG PAPVI G R

Sbjct 439 GGSLLALVAVCIGFPAPVIIWIYGRR 464

> EED11424 florfenicol exporter, putative [Talaromyces stipitatus

ATCC 10500]

Length=478

Score = 24.3 bits (51), Expect = 0.91, Method: Composition-based stats.

Identities = 14/26 (54%), Positives = 15/26 (58%), Gaps = 0/26 (0%)

Frame = +1

Query 97 GGRSLAIRLVDIGIPAPVIQAGIGHR 174

GG LA+ V IG PAPVI G R

Sbjct 439 GGSLLALVAVCIGFPAPVIIWIYGRR 464

> EED11423 florfenicol exporter, putative [Talaromyces stipitatus

ATCC 10500]

Length=478

Score = 24.3 bits (51), Expect = 0.91, Method: Composition-based stats.

Identities = 14/26 (54%), Positives = 15/26 (58%), Gaps = 0/26 (0%)

Frame = +1

Query 97 GGRSLAIRLVDIGIPAPVIQAGIGHR 174

GG LA+ V IG PAPVI G R

Sbjct 439 GGSLLALVAVCIGFPAPVIIWIYGRR 464

Lambda K H a alpha

0.318 0.134 0.401 0.792 4.96

Gapped

Lambda K H a alpha sigma

0.267 0.0410 0.140 1.90 42.6 43.6

Effective search space used: 17736928

**Query=** NODE_379_length_441_cov_0.665605

Length=441

***** No hits found *****

Lambda K H a alpha

0.318 0.134 0.401 0.792 4.96

Gapped

Lambda K H a alpha sigma

0.267 0.0410 0.140 1.90 42.6 43.6

Effective search space used: 17736928

**Query=** NODE_380_length_440_cov_0.565495

Length=440

***** No hits found *****

Lambda K H a alpha

0.318 0.134 0.401 0.792 4.96

Gapped

Lambda K H a alpha sigma

0.267 0.0410 0.140 1.90 42.6 43.6

Effective search space used: 17520624

**Query=** NODE_381_length_439_cov_0.269231

Length=439

***** No hits found *****

Lambda K H a alpha

0.318 0.134 0.401 0.792 4.96

Gapped

Lambda K H a alpha sigma

0.267 0.0410 0.140 1.90 42.6 43.6

Effective search space used: 17520624

**Query=** NODE_382_length_439_cov_0.657051

Length=439

***** No hits found *****

Lambda K H a alpha

0.318 0.134 0.401 0.792 4.96

Gapped

Lambda K H a alpha sigma

0.267 0.0410 0.140 1.90 42.6 43.6

Effective search space used: 17520624

**Query=** NODE_383_length_438_cov_0.662379

Length=438

***** No hits found *****

Lambda K H a alpha

0.318 0.134 0.401 0.792 4.96

Gapped

Lambda K H a alpha sigma

0.267 0.0410 0.140 1.90 42.6 43.6

Effective search space used: 17520624

**Query=** NODE_384_length_435_cov_0.554839

Length=435

Score E

Sequences producing significant alignments: (Bits) Value

XP_002480947 florfenicol exporter, putative [Talaromyces stipit... [24.6](file:///Users/JMBNew/Desktop/Articles%20en%20Cours/Article%20Diane/Bb_SR-11-14_output-blast_named.html#BL_ORD_ID:583) 0.83

EED20513 florfenicol exporter, putative [Talaromyces stipitatus... [24.6](file:///Users/JMBNew/Desktop/Articles%20en%20Cours/Article%20Diane/Bb_SR-11-14_output-blast_named.html#BL_ORD_ID:424) 0.83

> XP_002480947 florfenicol exporter, putative [Talaromyces stipitatus

ATCC 10500]

Length=564

Score = 24.6 bits (52), Expect = 0.83, Method: Composition-based stats.

Identities = 11/25 (44%), Positives = 15/25 (60%), Gaps = 1/25 (4%)

Frame = +3

Query 360 AQHTIPSPTPTQAHAPRPTPTTLLV 434

A+ T+P PTP + P P PT +V

Sbjct 315 AESTLP-PTPRKLSFPNPLPTLAIV 338

> EED20513 florfenicol exporter, putative [Talaromyces stipitatus

ATCC 10500]

Length=564

Score = 24.6 bits (52), Expect = 0.83, Method: Composition-based stats.

Identities = 11/25 (44%), Positives = 15/25 (60%), Gaps = 1/25 (4%)

Frame = +3

Query 360 AQHTIPSPTPTQAHAPRPTPTTLLV 434

A+ T+P PTP + P P PT +V

Sbjct 315 AESTLP-PTPRKLSFPNPLPTLAIV 338

Lambda K H a alpha

0.318 0.134 0.401 0.792 4.96

Gapped

Lambda K H a alpha sigma

0.267 0.0410 0.140 1.90 42.6 43.6

Effective search space used: 17304320

**Query=** NODE_385_length_437_cov_1.00645

Length=437

***** No hits found *****

Lambda K H a alpha

0.318 0.134 0.401 0.792 4.96

Gapped

Lambda K H a alpha sigma

0.267 0.0410 0.140 1.90 42.6 43.6

Effective search space used: 17304320

**Query=** NODE_386_length_437_cov_0.616129

Length=437

Score E

Sequences producing significant alignments: (Bits) Value

AQW34614 ABC transporter ATP-binding protein [Staphylococcus sc... [31.6](file:///Users/JMBNew/Desktop/Articles%20en%20Cours/Article%20Diane/Bb_SR-11-14_output-blast_named.html#BL_ORD_ID:248) 0.003

AQW34663 ABC transporter ATP-binding protein [Staphylococcus sc... [31.6](file:///Users/JMBNew/Desktop/Articles%20en%20Cours/Article%20Diane/Bb_SR-11-14_output-blast_named.html#BL_ORD_ID:258) 0.004

YP_004810315 putative multi-drug ABC transporter permease/ATPas... [30.8](file:///Users/JMBNew/Desktop/Articles%20en%20Cours/Article%20Diane/Bb_SR-11-14_output-blast_named.html#BL_ORD_ID:623) 0.005

AEM66524 putative multi-drug ABC transporter permease/ATPase (p... [30.8](file:///Users/JMBNew/Desktop/Articles%20en%20Cours/Article%20Diane/Bb_SR-11-14_output-blast_named.html#BL_ORD_ID:74) 0.005

YP_004800000 putative multi-drug ABC transporter permease/ATPas... [30.8](file:///Users/JMBNew/Desktop/Articles%20en%20Cours/Article%20Diane/Bb_SR-11-14_output-blast_named.html#BL_ORD_ID:619) 0.005

AEM66534 putative multi-drug ABC transporter permease/ATPase (p... [30.8](file:///Users/JMBNew/Desktop/Articles%20en%20Cours/Article%20Diane/Bb_SR-11-14_output-blast_named.html#BL_ORD_ID:79) 0.005

ARA90579 phenicol and oxazolidinone resistance protein [Staphyl... [27.7](file:///Users/JMBNew/Desktop/Articles%20en%20Cours/Article%20Diane/Bb_SR-11-14_output-blast_named.html#BL_ORD_ID:288) 0.071

AQW34744 phenicol and oxazolidinone resistance protein [Staphyl... [27.7](file:///Users/JMBNew/Desktop/Articles%20en%20Cours/Article%20Diane/Bb_SR-11-14_output-blast_named.html#BL_ORD_ID:278) 0.071

AQW34730 phenicol and oxazolidinone resistance protein [Staphyl... [27.7](file:///Users/JMBNew/Desktop/Articles%20en%20Cours/Article%20Diane/Bb_SR-11-14_output-blast_named.html#BL_ORD_ID:274) 0.071

AQW34667 ABC transporter ATP-binding protein (plasmid) [Staphyl... [27.7](file:///Users/JMBNew/Desktop/Articles%20en%20Cours/Article%20Diane/Bb_SR-11-14_output-blast_named.html#BL_ORD_ID:259) 0.071

AQW34661 phenicol and oxazolidinone resistance protein [Staphyl... [27.7](file:///Users/JMBNew/Desktop/Articles%20en%20Cours/Article%20Diane/Bb_SR-11-14_output-blast_named.html#BL_ORD_ID:256) 0.071

AQW34617 OptrA (plasmid) [Staphylococcus sciuri] [27.7](file:///Users/JMBNew/Desktop/Articles%20en%20Cours/Article%20Diane/Bb_SR-11-14_output-blast_named.html#BL_ORD_ID:249) 0.071

AQW34612 phenicol and oxazolidinone resistance protein [Staphyl... [27.7](file:///Users/JMBNew/Desktop/Articles%20en%20Cours/Article%20Diane/Bb_SR-11-14_output-blast_named.html#BL_ORD_ID:246) 0.071

AQW34601 phenicol and oxazolidinone resistance protein [Staphyl... [27.7](file:///Users/JMBNew/Desktop/Articles%20en%20Cours/Article%20Diane/Bb_SR-11-14_output-blast_named.html#BL_ORD_ID:245) 0.071

AQW34567 ABC transporter ATP-binding protein [Staphylococcus sc... [27.7](file:///Users/JMBNew/Desktop/Articles%20en%20Cours/Article%20Diane/Bb_SR-11-14_output-blast_named.html#BL_ORD_ID:238) 0.071

AQW34662 multidrug ABC transporter ATP-binding protein [Staphyl... [27.3](file:///Users/JMBNew/Desktop/Articles%20en%20Cours/Article%20Diane/Bb_SR-11-14_output-blast_named.html#BL_ORD_ID:257) 0.090

AQW34613 multidrug ABC transporter ATP-binding protein [Staphyl... [27.3](file:///Users/JMBNew/Desktop/Articles%20en%20Cours/Article%20Diane/Bb_SR-11-14_output-blast_named.html#BL_ORD_ID:247) 0.090

AKU20095 putative ABC transporter [Enterococcus faecium] [25.4](file:///Users/JMBNew/Desktop/Articles%20en%20Cours/Article%20Diane/Bb_SR-11-14_output-blast_named.html#BL_ORD_ID:204) 0.38

> AQW34614 ABC transporter ATP-binding protein [Staphylococcus

sciuri]

Length=582

Score = 31.6 bits (70), Expect = 0.003, Method: Composition-based stats.

Identities = 12/37 (32%), Positives = 25/37 (68%), Gaps = 0/37 (0%)

Frame = -1

Query 239 QRGEVVAVIGPSGSGKSTFLRCLNHLEIIDEGSIEVE 129

++G+ +A++G +GSGKS+ + L +G+IE++

Sbjct 367 EKGQTIALVGHTGSGKSSIMNLLFRFYDPTDGTIEID 403

> AQW34663 ABC transporter ATP-binding protein [Staphylococcus

sciuri]

Length=582

Score = 31.6 bits (70), Expect = 0.004, Method: Composition-based stats.

Identities = 13/37 (35%), Positives = 24/37 (65%), Gaps = 0/37 (0%)

Frame = -1

Query 236 RGEVVAVIGPSGSGKSTFLRCLNHLEIIDEGSIEVEG 126

+G+ +A++G +GSGKS+ + L G+IE++G

Sbjct 368 KGQTIALVGHTGSGKSSIMNLLFRFYDPTVGTIEIDG 404

> YP_004810315 putative multi-drug ABC transporter permease/ATPase

(plasmid) [Riemerella anatipestifer]

Length=373

Score = 30.8 bits (68), Expect = 0.005, Method: Compositional matrix adjust.

Identities = 15/40 (38%), Positives = 25/40 (63%), Gaps = 2/40 (5%)

Frame = -1

Query 245 PRQRGEVVAVIGPSGSGKSTFLRCLNHLEIIDEGSIEVEG 126

P+++ V A++G SGSGK+T L+ L +G I++ G

Sbjct 157 PKEK--VTAIVGASGSGKTTLLKILLKFYEPTQGKIKING 194

> AEM66524 putative multi-drug ABC transporter permease/ATPase

(plasmid) [Riemerella anatipestifer]

Length=373

Score = 30.8 bits (68), Expect = 0.005, Method: Compositional matrix adjust.

Identities = 15/40 (38%), Positives = 25/40 (63%), Gaps = 2/40 (5%)

Frame = -1

Query 245 PRQRGEVVAVIGPSGSGKSTFLRCLNHLEIIDEGSIEVEG 126

P+++ V A++G SGSGK+T L+ L +G I++ G

Sbjct 157 PKEK--VTAIVGASGSGKTTLLKILLKFYEPTQGKIKING 194

> YP_004800000 putative multi-drug ABC transporter permease/ATPase

(plasmid) [Riemerella anatipestifer]

Length=373

Score = 30.8 bits (68), Expect = 0.005, Method: Compositional matrix adjust.

Identities = 15/40 (38%), Positives = 25/40 (63%), Gaps = 2/40 (5%)

Frame = -1

Query 245 PRQRGEVVAVIGPSGSGKSTFLRCLNHLEIIDEGSIEVEG 126

P+++ V A++G SGSGK+T L+ L +G I++ G

Sbjct 157 PKEK--VTAIVGASGSGKTTLLKILLKFYEPTQGKIKING 194

> AEM66534 putative multi-drug ABC transporter permease/ATPase

(plasmid) [Riemerella anatipestifer]

Length=373

Score = 30.8 bits (68), Expect = 0.005, Method: Compositional matrix adjust.

Identities = 15/40 (38%), Positives = 25/40 (63%), Gaps = 2/40 (5%)

Frame = -1

Query 245 PRQRGEVVAVIGPSGSGKSTFLRCLNHLEIIDEGSIEVEG 126

P+++ V A++G SGSGK+T L+ L +G I++ G

Sbjct 157 PKEK--VTAIVGASGSGKTTLLKILLKFYEPTQGKIKING 194

> ARA90579 phenicol and oxazolidinone resistance protein [Staphylococcus

sciuri]

Length=655

Score = 27.7 bits (60), Expect = 0.071, Method: Composition-based stats.

Identities = 10/23 (43%), Positives = 17/23 (74%), Gaps = 0/23 (0%)

Frame = -1

Query 239 QRGEVVAVIGPSGSGKSTFLRCL 171

+RG+ + ++G +G GKST L+ L

Sbjct 369 ERGQKLGIVGSNGIGKSTLLKTL 391

Score = 26.2 bits (56), Expect = 0.24, Method: Composition-based stats.

Identities = 12/38 (32%), Positives = 24/38 (63%), Gaps = 0/38 (0%)

Frame = -1

Query 230 EVVAVIGPSGSGKSTFLRCLNHLEIIDEGSIEVEGEAL 117

E +A++G +G GK+T L+ + ++EG+ E E + +

Sbjct 47 EKIAIVGRNGCGKTTLLKAIIGEIELEEGTGESEFQVI 84

> AQW34744 phenicol and oxazolidinone resistance protein [Staphylococcus

sciuri]

Length=655

Score = 27.7 bits (60), Expect = 0.071, Method: Composition-based stats.

Identities = 10/23 (43%), Positives = 17/23 (74%), Gaps = 0/23 (0%)

Frame = -1

Query 239 QRGEVVAVIGPSGSGKSTFLRCL 171

+RG+ + ++G +G GKST L+ L

Sbjct 369 ERGQKLGIVGSNGIGKSTLLKTL 391

Score = 26.2 bits (56), Expect = 0.24, Method: Composition-based stats.

Identities = 12/38 (32%), Positives = 24/38 (63%), Gaps = 0/38 (0%)

Frame = -1

Query 230 EVVAVIGPSGSGKSTFLRCLNHLEIIDEGSIEVEGEAL 117

E +A++G +G GK+T L+ + ++EG+ E E + +

Sbjct 47 EKIAIVGRNGCGKTTLLKAIIGEIELEEGTGESEFQVI 84

> AQW34730 phenicol and oxazolidinone resistance protein [Staphylococcus

sciuri]

Length=655

Score = 27.7 bits (60), Expect = 0.071, Method: Composition-based stats.

Identities = 10/23 (43%), Positives = 17/23 (74%), Gaps = 0/23 (0%)

Frame = -1

Query 239 QRGEVVAVIGPSGSGKSTFLRCL 171

+RG+ + ++G +G GKST L+ L

Sbjct 369 ERGQKLGIVGSNGIGKSTLLKTL 391

Score = 26.2 bits (56), Expect = 0.24, Method: Composition-based stats.

Identities = 12/38 (32%), Positives = 24/38 (63%), Gaps = 0/38 (0%)

Frame = -1

Query 230 EVVAVIGPSGSGKSTFLRCLNHLEIIDEGSIEVEGEAL 117

E +A++G +G GK+T L+ + ++EG+ E E + +

Sbjct 47 EKIAIVGRNGCGKTTLLKAIIGEIELEEGTGESEFQVI 84

> AQW34667 ABC transporter ATP-binding protein (plasmid) [Staphylococcus

sciuri]

Length=655

Score = 27.7 bits (60), Expect = 0.071, Method: Composition-based stats.

Identities = 10/23 (43%), Positives = 17/23 (74%), Gaps = 0/23 (0%)

Frame = -1

Query 239 QRGEVVAVIGPSGSGKSTFLRCL 171

+RG+ + ++G +G GKST L+ L

Sbjct 369 ERGQKLGIVGSNGIGKSTLLKTL 391

Score = 26.2 bits (56), Expect = 0.24, Method: Composition-based stats.

Identities = 12/38 (32%), Positives = 24/38 (63%), Gaps = 0/38 (0%)

Frame = -1

Query 230 EVVAVIGPSGSGKSTFLRCLNHLEIIDEGSIEVEGEAL 117

E +A++G +G GK+T L+ + ++EG+ E E + +

Sbjct 47 EKIAIVGRNGCGKTTLLKAIIGEIELEEGTGESEFQVI 84

> AQW34661 phenicol and oxazolidinone resistance protein [Staphylococcus

sciuri]

Length=655

Score = 27.7 bits (60), Expect = 0.071, Method: Composition-based stats.

Identities = 10/23 (43%), Positives = 17/23 (74%), Gaps = 0/23 (0%)

Frame = -1

Query 239 QRGEVVAVIGPSGSGKSTFLRCL 171

+RG+ + ++G +G GKST L+ L

Sbjct 369 ERGQKLGIVGSNGIGKSTLLKTL 391

Score = 26.2 bits (56), Expect = 0.24, Method: Composition-based stats.

Identities = 12/38 (32%), Positives = 24/38 (63%), Gaps = 0/38 (0%)

Frame = -1

Query 230 EVVAVIGPSGSGKSTFLRCLNHLEIIDEGSIEVEGEAL 117

E +A++G +G GK+T L+ + ++EG+ E E + +

Sbjct 47 EKIAIVGRNGCGKTTLLKAIIGEIELEEGTGESEFQVI 84

> AQW34617 OptrA (plasmid) [Staphylococcus sciuri]

Length=655

Score = 27.7 bits (60), Expect = 0.071, Method: Composition-based stats.

Identities = 10/23 (43%), Positives = 17/23 (74%), Gaps = 0/23 (0%)

Frame = -1

Query 239 QRGEVVAVIGPSGSGKSTFLRCL 171

+RG+ + ++G +G GKST L+ L

Sbjct 369 ERGQKLGIVGSNGIGKSTLLKTL 391

Score = 26.2 bits (56), Expect = 0.24, Method: Composition-based stats.

Identities = 12/38 (32%), Positives = 24/38 (63%), Gaps = 0/38 (0%)

Frame = -1

Query 230 EVVAVIGPSGSGKSTFLRCLNHLEIIDEGSIEVEGEAL 117

E +A++G +G GK+T L+ + ++EG+ E E + +

Sbjct 47 EKIAIVGRNGCGKTTLLKAIIGEIELEEGTGESEFQVI 84

> AQW34612 phenicol and oxazolidinone resistance protein [Staphylococcus

sciuri]

Length=655

Score = 27.7 bits (60), Expect = 0.071, Method: Composition-based stats.

Identities = 10/23 (43%), Positives = 17/23 (74%), Gaps = 0/23 (0%)

Frame = -1

Query 239 QRGEVVAVIGPSGSGKSTFLRCL 171

+RG+ + ++G +G GKST L+ L

Sbjct 369 ERGQKLGIVGSNGIGKSTLLKTL 391

Score = 26.2 bits (56), Expect = 0.24, Method: Composition-based stats.

Identities = 12/38 (32%), Positives = 24/38 (63%), Gaps = 0/38 (0%)

Frame = -1

Query 230 EVVAVIGPSGSGKSTFLRCLNHLEIIDEGSIEVEGEAL 117

E +A++G +G GK+T L+ + ++EG+ E E + +

Sbjct 47 EKIAIVGRNGCGKTTLLKAIIGEIELEEGTGESEFQVI 84

> AQW34601 phenicol and oxazolidinone resistance protein [Staphylococcus

sciuri]

Length=655

Score = 27.7 bits (60), Expect = 0.071, Method: Composition-based stats.

Identities = 10/23 (43%), Positives = 17/23 (74%), Gaps = 0/23 (0%)

Frame = -1

Query 239 QRGEVVAVIGPSGSGKSTFLRCL 171

+RG+ + ++G +G GKST L+ L

Sbjct 369 ERGQKLGIVGSNGIGKSTLLKTL 391

Score = 26.2 bits (56), Expect = 0.24, Method: Composition-based stats.

Identities = 12/38 (32%), Positives = 24/38 (63%), Gaps = 0/38 (0%)

Frame = -1

Query 230 EVVAVIGPSGSGKSTFLRCLNHLEIIDEGSIEVEGEAL 117

E +A++G +G GK+T L+ + ++EG+ E E + +

Sbjct 47 EKIAIVGRNGCGKTTLLKAIIGEIELEEGTGESEFQVI 84

> AQW34567 ABC transporter ATP-binding protein [Staphylococcus

sciuri]

Length=655

Score = 27.7 bits (60), Expect = 0.071, Method: Composition-based stats.

Identities = 10/23 (43%), Positives = 17/23 (74%), Gaps = 0/23 (0%)

Frame = -1

Query 239 QRGEVVAVIGPSGSGKSTFLRCL 171

+RG+ + ++G +G GKST L+ L

Sbjct 369 ERGQKLGIVGSNGIGKSTLLKTL 391

Score = 26.2 bits (56), Expect = 0.24, Method: Composition-based stats.

Identities = 12/38 (32%), Positives = 24/38 (63%), Gaps = 0/38 (0%)

Frame = -1

Query 230 EVVAVIGPSGSGKSTFLRCLNHLEIIDEGSIEVEGEAL 117

E +A++G +G GK+T L+ + ++EG+ E E + +

Sbjct 47 EKIAIVGRNGCGKTTLLKAIIGEIELEEGTGESEFQVI 84

> AQW34662 multidrug ABC transporter ATP-binding protein [Staphylococcus

sciuri]

Length=579

Score = 27.3 bits (59), Expect = 0.090, Method: Composition-based stats.

Identities = 8/20 (40%), Positives = 16/20 (80%), Gaps = 0/20 (0%)

Frame = -1

Query 236 RGEVVAVIGPSGSGKSTFLR 177

+GE + ++G +GSGK+T ++

Sbjct 361 KGETLGIVGATGSGKTTLIK 380

> AQW34613 multidrug ABC transporter ATP-binding protein [Staphylococcus

sciuri]

Length=579

Score = 27.3 bits (59), Expect = 0.090, Method: Composition-based stats.

Identities = 8/20 (40%), Positives = 16/20 (80%), Gaps = 0/20 (0%)

Frame = -1

Query 236 RGEVVAVIGPSGSGKSTFLR 177

+GE + ++G +GSGK+T ++

Sbjct 361 KGETLGIVGATGSGKTTLIK 380

> AKU20095 putative ABC transporter [Enterococcus faecium]

Length=542

Score = 25.4 bits (54), Expect = 0.38, Method: Composition-based stats.

Identities = 13/31 (42%), Positives = 18/31 (58%), Gaps = 0/31 (0%)

Frame = -1

Query 230 EVVAVIGPSGSGKSTFLRCLNHLEIIDEGSI 138

E V+G +G GKST L+ + + DEG I

Sbjct 357 ERFLVVGENGVGKSTLLKLMMGILSPDEGCI 387

Score = 25.0 bits (53), Expect = 0.55, Method: Composition-based stats.

Identities = 13/30 (43%), Positives = 20/30 (67%), Gaps = 2/30 (7%)

Frame = -1

Query 224 VAVIGPSGSGKSTFLR-CLNHLEIIDEGSI 138

V ++G +G+GK+T R L LE +D GS+

Sbjct 29 VGIVGVNGAGKTTLFRLLLGELE-LDNGSL 57

Lambda K H a alpha

0.318 0.134 0.401 0.792 4.96

Gapped

Lambda K H a alpha sigma

0.267 0.0410 0.140 1.90 42.6 43.6

Effective search space used: 17304320

**Query=** NODE_387_length_437_cov_0.667742

Length=437

***** No hits found *****

Lambda K H a alpha

0.318 0.134 0.401 0.792 4.96

Gapped

Lambda K H a alpha sigma

0.267 0.0410 0.140 1.90 42.6 43.6

Effective search space used: 17304320

**Query=** NODE_388_length_437_cov_0.664516

Length=437

***** No hits found *****

Lambda K H a alpha

0.318 0.134 0.401 0.792 4.96

Gapped

Lambda K H a alpha sigma

0.267 0.0410 0.140 1.90 42.6 43.6

Effective search space used: 17304320

**Query=** NODE_389_length_437_cov_0.309677

Length=437

***** No hits found *****

Lambda K H a alpha

0.318 0.134 0.401 0.792 4.96

Gapped

Lambda K H a alpha sigma

0.267 0.0410 0.140 1.90 42.6 43.6

Effective search space used: 17304320

**Query=** NODE_390_length_437_cov_0.716129

Length=437

***** No hits found *****

Lambda K H a alpha

0.318 0.134 0.401 0.792 4.96

Gapped

Lambda K H a alpha sigma

0.267 0.0410 0.140 1.90 42.6 43.6

Effective search space used: 17304320

**Query=** NODE_391_length_437_cov_0.429032

Length=437

***** No hits found *****

Lambda K H a alpha

0.318 0.134 0.401 0.792 4.96

Gapped

Lambda K H a alpha sigma

0.267 0.0410 0.140 1.90 42.6 43.6

Effective search space used: 17304320

**Query=** NODE_392_length_436_cov_0.23301

Length=436

***** No hits found *****

Lambda K H a alpha

0.308 0.119 0.299 0.792 4.96

Gapped

Lambda K H a alpha sigma

0.267 0.0410 0.140 1.90 42.6 43.6

Effective search space used: 17304320

**Query=** NODE_393_length_436_cov_0.79288

Length=436

***** No hits found *****

Lambda K H a alpha

0.318 0.134 0.401 0.792 4.96

Gapped

Lambda K H a alpha sigma

0.267 0.0410 0.140 1.90 42.6 43.6

Effective search space used: 17304320

**Query=** NODE_394_length_436_cov_0.721683

Length=436

***** No hits found *****

Lambda K H a alpha

0.318 0.134 0.401 0.792 4.96

Gapped

Lambda K H a alpha sigma

0.267 0.0410 0.140 1.90 42.6 43.6

Effective search space used: 17304320

**Query=** NODE_395_length_435_cov_1.22006

Length=435

***** No hits found *****

Lambda K H a alpha

0.318 0.134 0.401 0.792 4.96

Gapped

Lambda K H a alpha sigma

0.267 0.0410 0.140 1.90 42.6 43.6

Effective search space used: 17304320

**Query=** NODE_396_length_436_cov_0.453074

Length=436

***** No hits found *****

Lambda K H a alpha

0.318 0.134 0.401 0.792 4.96

Gapped

Lambda K H a alpha sigma

0.267 0.0410 0.140 1.90 42.6 43.6

Effective search space used: 17304320

**Query=** NODE_397_length_435_cov_2.46429

Length=435

***** No hits found *****

Lambda K H a alpha

0.318 0.134 0.401 0.792 4.96

Gapped

Lambda K H a alpha sigma

0.267 0.0410 0.140 1.90 42.6 43.6

Effective search space used: 17304320

**Query=** NODE_398_length_434_cov_0.295455

Length=434

***** No hits found *****

Lambda K H a alpha

0.318 0.134 0.401 0.792 4.96

Gapped

Lambda K H a alpha sigma

0.267 0.0410 0.140 1.90 42.6 43.6

Effective search space used: 17088016

**Query=** NODE_399_length_434_cov_0.729642

Length=434

***** No hits found *****

Lambda K H a alpha

0.318 0.134 0.401 0.792 4.96

Gapped

Lambda K H a alpha sigma

0.267 0.0410 0.140 1.90 42.6 43.6

Effective search space used: 17088016

**Query=** NODE_400_length_433_cov_0.823529

Length=433

***** No hits found *****

Lambda K H a alpha

0.318 0.134 0.401 0.792 4.96

Gapped

Lambda K H a alpha sigma

0.267 0.0410 0.140 1.90 42.6 43.6

Effective search space used: 17088016

**Query=** NODE_401_length_433_cov_0.849673

Length=433

***** No hits found *****

Lambda K H a alpha

0.318 0.134 0.401 0.792 4.96

Gapped

Lambda K H a alpha sigma

0.267 0.0410 0.140 1.90 42.6 43.6

Effective search space used: 17088016

**Query=** NODE_402_length_432_cov_0.653595

Length=432

***** No hits found *****

Lambda K H a alpha

0.318 0.134 0.401 0.792 4.96

Gapped

Lambda K H a alpha sigma

0.267 0.0410 0.140 1.90 42.6 43.6

Effective search space used: 17088016

**Query=** NODE_403_length_432_cov_0.72459

Length=432

***** No hits found *****

Lambda K H a alpha

0.318 0.134 0.401 0.792 4.96

Gapped

Lambda K H a alpha sigma

0.267 0.0410 0.140 1.90 42.6 43.6

Effective search space used: 17088016

**Query=** NODE_404_length_432_cov_0.495082

Length=432

Score E

Sequences producing significant alignments: (Bits) Value

KTD48180 florfenicol efflux pump [Legionella quinlivanii] [26.2](file:///Users/JMBNew/Desktop/Articles%20en%20Cours/Article%20Diane/Bb_SR-11-14_output-blast_named.html#BL_ORD_ID:497) 0.24

> KTD48180 florfenicol efflux pump [Legionella quinlivanii]

Length=396

Score = 26.2 bits (56), Expect = 0.24, Method: Compositional matrix adjust.

Identities = 10/17 (59%), Positives = 11/17 (65%), Gaps = 0/17 (0%)

Frame = +3

Query 330 KPPHPAISAPNGWEKPR 380

+ P P ISA NGWE R

Sbjct 190 QFPKPKISAANGWEAMR 206

Lambda K H a alpha

0.318 0.134 0.401 0.792 4.96

Gapped

Lambda K H a alpha sigma

0.267 0.0410 0.140 1.90 42.6 43.6

Effective search space used: 17088016

**Query=** NODE_405_length_432_cov_0.695082

Length=432

***** No hits found *****

Lambda K H a alpha

0.318 0.134 0.401 0.792 4.96

Gapped

Lambda K H a alpha sigma

0.267 0.0410 0.140 1.90 42.6 43.6

Effective search space used: 17088016

**Query=** NODE_406_length_431_cov_0.690789

Length=431

***** No hits found *****

Lambda K H a alpha

0.318 0.134 0.401 0.792 4.96

Gapped

Lambda K H a alpha sigma

0.267 0.0410 0.140 1.90 42.6 43.6

Effective search space used: 16871712

**Query=** NODE_407_length_430_cov_0.697368

Length=430

***** No hits found *****

Lambda K H a alpha

0.318 0.134 0.401 0.792 4.96

Gapped

Lambda K H a alpha sigma

0.267 0.0410 0.140 1.90 42.6 43.6

Effective search space used: 16871712

**Query=** NODE_408_length_431_cov_0.480263

Length=431

***** No hits found *****

Lambda K H a alpha

0.318 0.134 0.401 0.792 4.96

Gapped

Lambda K H a alpha sigma

0.267 0.0410 0.140 1.90 42.6 43.6

Effective search space used: 16871712

**Query=** NODE_409_length_429_cov_0.653465

Length=429

***** No hits found *****

Lambda K H a alpha

0.318 0.134 0.401 0.792 4.96

Gapped

Lambda K H a alpha sigma

0.267 0.0410 0.140 1.90 42.6 43.6

Effective search space used: 16871712

**Query=** NODE_410_length_430_cov_0.79538

Length=430

***** No hits found *****

Lambda K H a alpha

0.318 0.134 0.401 0.792 4.96

Gapped

Lambda K H a alpha sigma

0.267 0.0410 0.140 1.90 42.6 43.6

Effective search space used: 16871712

**Query=** NODE_411_length_430_cov_0.524752

Length=430

***** No hits found *****

Lambda K H a alpha

0.318 0.134 0.401 0.792 4.96

Gapped

Lambda K H a alpha sigma

0.267 0.0410 0.140 1.90 42.6 43.6

Effective search space used: 16871712

**Query=** NODE_412_length_430_cov_0.587459

Length=430

***** No hits found *****

Lambda K H a alpha

0.318 0.134 0.401 0.792 4.96

Gapped

Lambda K H a alpha sigma

0.267 0.0410 0.140 1.90 42.6 43.6

Effective search space used: 16871712

**Query=** NODE_413_length_428_cov_0.625828

Length=428

***** No hits found *****

Lambda K H a alpha

0.318 0.134 0.401 0.792 4.96

Gapped

Lambda K H a alpha sigma

0.267 0.0410 0.140 1.90 42.6 43.6

Effective search space used: 16655408

**Query=** NODE_414_length_428_cov_0.433775

Length=428

Score E

Sequences producing significant alignments: (Bits) Value

CDN53419 Florfenicol resistance protein [Neorhizobium galegae b... [25.4](file:///Users/JMBNew/Desktop/Articles%20en%20Cours/Article%20Diane/Bb_SR-11-14_output-blast_named.html#BL_ORD_ID:374) 0.40

CDN47273 Florfenicol resistance protein [Neorhizobium galegae b... [25.0](file:///Users/JMBNew/Desktop/Articles%20en%20Cours/Article%20Diane/Bb_SR-11-14_output-blast_named.html#BL_ORD_ID:373) 0.42

> CDN53419 Florfenicol resistance protein [Neorhizobium galegae

bv. officinalis bv. officinalis str. HAMBI 1141]

Length=292

Score = 25.4 bits (54), Expect = 0.40, Method: Compositional matrix adjust.

Identities = 18/52 (35%), Positives = 27/52 (52%), Gaps = 4/52 (8%)

Frame = +3

Query 84 GIGLGHL----AQGVGHVRIFLEAVVDACLPARRADLVHLEALVLRDVGHIG 227

GIGL L AQ G R + +++A LPA+ ADL+ +R++ G

Sbjct 43 GIGLYDLSSDEAQKTGEWRDGIGRLMEAELPAKVADLLEPYLTAVRELNPTG 94

> CDN47273 Florfenicol resistance protein [Neorhizobium galegae

bv. orientalis str. HAMBI 540]

Length=292

Score = 25.0 bits (53), Expect = 0.42, Method: Compositional matrix adjust.

Identities = 18/52 (35%), Positives = 27/52 (52%), Gaps = 4/52 (8%)

Frame = +3

Query 84 GIGLGHL----AQGVGHVRIFLEAVVDACLPARRADLVHLEALVLRDVGHIG 227

GIGL L AQ G R + +++A LPA+ ADL+ +R++ G

Sbjct 43 GIGLYDLSSDEAQKTGEWRDGIGRLMEAELPAKVADLLEPYLTAVRELNPTG 94

Lambda K H a alpha

0.318 0.134 0.401 0.792 4.96

Gapped

Lambda K H a alpha sigma

0.267 0.0410 0.140 1.90 42.6 43.6

Effective search space used: 16655408

**Query=** NODE_415_length_136_cov_6.11111

Length=136

***** No hits found *****

Effective search space used: 0

**Query=** NODE_416_length_128_cov_37365

Length=128

***** No hits found *****

Effective search space used: 0

Database: BdD_Prot-name.fasta

Posted date: Nov 10, 2017 10:32 AM

Number of letters in database: 261,934

Number of sequences in database: 702

Matrix: BLOSUM62

Gap Penalties: Existence: 11, Extension: 1

Neighboring words threshold: 12

Window for multiple hits: 40
